# Integrative analysis of SEPN1 in glioma: Prognostic roles, functional implications, and potential therapeutic interventions

**DOI:** 10.1371/journal.pone.0318501

**Published:** 2025-02-07

**Authors:** Zisong Wang, Danwen Wang, Xuanyu Wang, Yihang Xu, Yunhe Yuan, Yuxin Chen, Zhiqiang Li, Xiaoping Liu

**Affiliations:** 1 Department of Pathology, Zhongnan Hospital of Wuhan University, Wuhan, Hubei Province, China; 2 Department of Radiology, Zhongnan Hospital of Wuhan University, Wuhan, Hubei Province, China; 3 Department of Neurosurgery, Zhongnan Hospital of Wuhan University, Wuhan, Hubei Province, China; Al-Azhar University / King Khalid University, EGYPT

## Abstract

**Background:**

SEPN1, a selenoprotein involved in redox regulation and endoplasmic reticulum stress response, has an unclear role in cancer. This study aims to investigate the expression, prognostic significance, and tumor microenvironment (TME) relevance of SEPN1 across pan-cancer, with a particular focus on glioma.

**Methods:**

We analyzed SEPN1 expression and prognosis using the TCGA pan-cancer cohort. SEPN1 in glioma was further examined using data from TCGA, CGGA, GEO, and ZN-GC cohorts, along with survival analysis, single-cell RNA sequencing analysis, and enrichment analysis. We developed an SEPN1-related risk score (SRS) based on SEPN1-related long non-coding RNAs and validated its prognostic value. Drug sensitivity data and connectivity map analysis identified potential anti-glioma drugs based on the SRS.

**Results:**

We found that SEPN1 was significantly upregulated in glioma, associated with poor prognosis, functioned as an independent risk factor, and predominantly expressed in malignant glioma cells. Enrichment analysis indicated the involvement of SEPN1 in immune-related processes and signaling pathways. Suppressing SEPN1 in glioblastoma cells inhibited proliferation and induced G2/M arrest and apoptosis. The SRS demonstrated strong prognostic value and correlated with enhanced immune infiltration in the glioma TME. Potential anti-glioma drugs were identified based on the SRS.

**Conclusions:**

SEPN1 emerges as a novel biomarker and therapeutic target in glioma, providing a basis for future development of targeted therapies.

## Introduction

Glioma, the most common primary tumor of the central nervous system, poses significant treatment challenges due to its high invasiveness and poor prognosis, with an average incidence of about 6 cases per 100,000 people annually and high-grade glioma exhibiting a median survival of about 18 months [1–3]. Despite advancements in surgery, radiotherapy, and chemotherapy, the overall survival (OS) rate of glioma patients remains very low [4]. The heterogeneity and complexity of the glioma tumor microenvironment (TME) further contribute to treatment resistance and tumor recurrence [5]. Therefore, identifying new biomarkers and therapeutic targets is crucial for improving the prognosis of glioma patients.

Selenium (Se) is an essential trace element for humans, playing a critical role in regulating immune function and oxidative stress [6]. Se is vital for the brain, and during Se depletion, the brain maintains its Se levels at the expense of other tissues, as Se deficiency can lead to irreversible brain damage [7]. In humans, the nutritional functions of Se are carried out by 25 selenoproteins, with selenocysteine at their active centers [8]. Selenoproteins are involved in antioxidant reactions, thus potentially protecting normal cells from oxidative stress; on the other hand, when normal cells transform into tumor cells, selenoproteins in tumors may adapt their functions to support the malignant phenotype [9].

One member of the selenoprotein family, SEPN1, also known as selenoprotein N (SELENON), is a glycoprotein located in the endoplasmic reticulum [10], essential for endoplasmic reticulum homeostasis and protection against oxidative stress [11]. Given that oxidative stress responses are often dysregulated in tumors [12], SEPN1 may play a role in cancer cell proliferation by regulating oxidative stress responses. However, the role of SEPN1 in cancer, particularly in glioma, remains largely unexplored. This study aims to comprehensively investigate the roles of SEPN1 in glioma, providing new insights for the understanding of glioma biology.

In the present study, we used the The Cancer Genome Atlas (TCGA) Pan-Cancer cohort to analyze the expression patterns and prognostic significance of SEPN1 across various types of cancers. We then focused on the roles of SEPN1 in glioma using multi-omics data from TCGA, the Chinese Glioma Genome Atlas (CGGA), Gene Expression Omnibus (GEO), and an independent glioma cohort from Zhongnan Hospital of Wuhan University (ZN-Glioma). We found that SEPN1 was upregulated in glioma and identified as an independent prognostic factor for glioma patients. Single-cell RNA sequencing (ScRNA-seq) analysis revealed that SEPN1 was primarily expressed in malignant cells of glioma. Functional enrichment analyses indicated that SEPN1 was involved in multiple biological processes and signaling pathways in glioma, including immune-related processes, cell cycle regulation, and apoptosis. Cell experiments confirmed that SEPN1 promoted glioma cell proliferation. Furthermore, based on SEPN1-related long non-coding RNAs (lncRNAs), we developed and validated an SEPN1-related risk score (SRS) and associated nomogram, and identified potential anti-glioma drugs based on SRS. Finally, the characteristics of the selenoprotein family across pan-cancer types were also explored. Our findings provide new insights into the role of SEPN1 in glioma and highlight the potential of SEPN1 as a prognostic biomarker and therapeutic target for glioma, laying the foundation for future research and translational applications.

## Materials and methods

### Glioma cohorts

To comprehensively analyze the role of SEPN1 in cancer, we obtained the pan-cancer gene expression matrix and tumor annotation files from the National Cancer Institute’s Genomic Data Commons (GDC) platform. Patient survival data were obtained from the University of California Santa Cruz (UCSC) Xena database [13]. Specifically focusing on glioma, we incorporated RNA sequencing (RNA-seq) and clinical information from four glioma-specific cohorts: TCGA-LGG [14], CGGA-693, CGGA-325 [15], and GSE16011 [16], accessed through the R package “CuratedCancerPrognosisData” [17]. Additionally, tumor tissue samples and corresponding clinical data from 42 glioma patients were collected at Zhongnan Hospital of Wuhan University, establishing the ZN-Glioma cohort. The protocol of the study was approved by the Committee of Ethics from Zhongnan Hospital of Wuhan University with a waiver of informed consent (Ethics No. 2024029K).

### Expression, prognosis, and tumor microenvironment (TME) characteristics of SEPN1 in pan-cancer

We began our analyses by extracting SEPN1 expression data from the TCGA TARGET GTEx cohort in the UCSC Xena database using the Sangerbox platform [18]. Data were log_2_(x+1) transformed for normalization before assessing expression differences between cancerous and adjacent non-cancerous samples using non-paired Wilcoxon Rank Sum and Signed Rank Tests. Univariate Cox proportional hazards regression models (CoxPHs) and Kaplan-Meier (KM) curve analyses were performed to evaluate the prognostic significance of SEPN1 expression in pan-cancer patients in terms of overall survival (OS), disease-specific survival (DSS), and disease-free interval (DFI) using the R package “survival”. SEPN1 expression was treated as a continuous variable in the CoxPHs, while patients were stratified based on the median expression level for KM models. A forest plot was used to visualize the impact of SEPN1 expression on OS across different cancer types. The TME was then explored using the ESTIMATE algorithm [19], which calculated immune, stromal, and ESTIMATE scores, and then correlations between SEPN1 expression and the three scores were assessed using Spearman’s correlation. Additionally, the relationship between SEPN1 expression and immune cell infiltration was investigated using the “Immune” module from the TIMER2.0 database [20], which incorporates data processed by six advanced algorithms: TIMER [21], xCell [22], MCP-counter [23], CIBERSORT [24], EPIC [25], and quanTIseq [26].

### Tissue microarrays (TMAs) construction and immunohistochemistry (IHC) staining

We constructed TMAs for the ZN-Glioma cohort using 42 human glioma samples. The retrospective data for this study were accessed on March 5, 2024. The authors had access to information that could identify individual participants during the data collection phase. After data collection, all identifiable information was anonymized to protect participant privacy. Samples were embedded, sectioned, and subjected to hematoxylin and eosin (H&E) staining. The TMAs were prepared through a series of steps including a 2-hour heat treatment at 62°C, deparaffinization in xylene, and rehydration through graded alcohols. High-temperature antigen retrieval was conducted using EDTA buffer (pH 7.4) at 120°C, followed by natural cooling. Endogenous peroxidase activity was suppressed by incubating the slides with 0.3% hydrogen peroxide in methanol for 30 minutes, followed by washes in phosphate buffered solution (PBS). Blocking was performed at room temperature using goat serum for 1 hour before applying the primary antibody, SEPN1 rabbit polyclonal antibody (Proteintech Wuhan Sanying), diluted according to instructions and applied overnight at 4°C for no more than 16 hours. Secondary antibodies were added the next day, and slides were incubated for 40 minutes in a humidified chamber. Color development was initiated with diaminobenzidine (DAB) staining, followed by counterstaining with hematoxylin and bluing in ammonia water. The TMAs were digitally scanned using a Hamamatsu NanoZoomer XR slide scanner. Finally, the analysis and quantification of positivity rates were performed with QuPath software (version 0.4.3) using its default parameters [27]. In detail, the regions of interest (ROIs) for each tissue core were automatically segmented which were then manually reviewed to exclude regions with artifacts, necrosis, or poor staining quality. Cell detection was performed using adaptive thresholding to segment nuclei and classify cells as positive or negative based on the intensity of DAB staining within nuclear or cytoplasmic. The positivity rate was calculated as the percentage of positively stained cells relative to the total number of cells within the finalized ROI.

### Association of SEPN1 with prognosis in glioma patients

Patients were categorized into high and low SEPN1 expression groups based on the median expression levels across four distinct cohorts: TCGA-LGG, CGGA-693, CGGA-325, and GSE16011. The R package “table1” was used to analyze the demographic and clinical differences between the groups, such as age, gender, histological type, grade, and stage. Survival outcomes were compared using KM curves generated using the R package “survminer”, and the restricted mean survival time (RMST) was computed using the R package “survRM2”. Initial analyses incorporated SEPN1 expression and relevant clinical data into univariate CoxPHs, followed by refinement using stepwise regression to develop multivariate CoxPHs that better captured the factors influencing prognosis. The robustness of our findings was confirmed through parallel analyses within the ZN-Glioma cohort, which reinforced the initial observations and provided a more comprehensive understanding of the role of SEPN1 in the prognosis of glioma patients.

### Expression characteristics of SEPN1 in single cells from glioma

To further investigate the cellular heterogeneity of glioma and the specific role of SEPN1, we utilized the TISCH2 database [28] to analyze its expression across various cell clusters. The analysis was based on the GSE131928 cohort [29], which employed both 10× Genomics and Smart-seq2 technologies for scRNA-seq. This approach provided insights into the expression patterns of SEPN1 at the single-cell level within glioma.

### Functional enrichment analyses of SEPN1 in glioma

To elucidate the functional role of SEPN1 in glioma, we identified differentially expressed genes (DEGs) between high and low SEPN1 expression groups across the TCGA-LGG, CGGA-693, CGGA-325, and GSE16011 cohorts using the R package “limma” [30]. Significant DEGs were selected based on an absolute log_2_ fold change (log_2_FC) greater than 2.0 and an adjusted p-value below 0.05. Subsequently, gene ontology (GO) analyses [31] for biological process (BP) and pathway analyses based on the Kyoto Encyclopedia of Genes and Genomes (KEGG) [32] were conducted using the R package “clusterProfiler” [33], with significance determined by Fisher’s exact test with p-values less than 0.05. We further assessed gene set enrichment across all samples by integrating 255 published characteristic gene sets sourced from the R package “IOBR” [34] and applying single sample gene set enrichment analysis (ssGSEA) using the R package “GSVA” [35]. Spearman’s correlation coefficients were calculated to visualize the associations of these biomarkers with SEPN1 expression, and significant associations (absolute rho > 0.3 and p-value < 0.05) were presented in heatmaps.

### Correlations between SEPN1 and immune checkpoints (ICs) in glioma

Cancer cells manipulate ICs to avoid immune attack, and monoclonal antibodies that inhibit this pathway have become an effective therapeutic strategy for cancer [36]. Therefore, we analyzed the associations between the expression of SPEN1 and common ICs across the TCGA-LGG, CGGA-693, CGGA-325, and GSE16011 cohorts.

### Cell culture and siRNA transfection

The U251 and U87 cell lines were obtained from the Cell Bank of the Chinese Academy of Sciences. The siRNAs targeted SEPN1 were synthesized from Tsingke (Wuhan, China), and the sequences (siSEPN1-1, siSEPN1-2, siSEPN1-3) are listed below: siSEPN1-1: 5′- AAGCTAACAGGGTCTTGTTCTGT-3′; siSEPN1-2: 5′-TTGTGTTTGAGGAGATCAAGTGG-3′; siSEPN1-3: 5′-GGCCATGTACCCCTTCAAGAAGG-3′; The siRNAs were transfected into the two glioma cell lines via Lipofectamine 3000 reagent.

### RNA extraction and qRT‒PCR

The RNA extraction assay was conducted by the RNeasy mini kit (Qiagen), which was carried out under 4°C. Afterwards, 1 μg RNA underwent reverse transcription to cDNA. Next, qRT-PCR was conducted with guidelines provided by the PCR Mix manufacturer. We conducted the qRT-PCR assays following the cycling program: 95°C for 3 min, then 94°C for 30 s plus 55°C for 30 s, and followed 72°C for 50 s (30 cycles). The primer sequences were showed below: SEPN1: 5′-CCTGACCCTAGCGAGGAGAC-3′; 3′-GGCTGTCCAGTTTCGGAGG-5′; GAPDH: 5′-TGTGGGCATCAATGGATTTGG-3′; 3′-ACACCATGTATTCCGGGTCAAT-5′.

### MTT assay

The glioma cells were evenly seeded into a 96-well plate, with approximately 3000 cells per well and 100 μL of medium per well. The cells are cultured in various time points in a CO_2_ incubator. Then, 20 μL of 0.5% MTT solution was added to each well, with incubating for 5 hours. Next, 150 μL of DMSO was added to every well. Then, the absorbance of each well at OD 490/570nm was measured using a microplate reader.

### Cell cycle and apoptosis assay

For cell cycle assay, the cells were collected and then washed once with PBS. After that, 1 μL of DNA dye and 10 μL of permeabilization reagent were added to the collected cells. Gently pipette to mix the cell suspension and incubate it at room temperature in the dark for 20 minutes. Then, we detected the cell cycle status through the flow cytometer. For cell apoptosis assay, the cells were collected using EDTA-free trypsin, and then centrifuged at 1300 rpm for 5 minutes. Subsequently, 5 μL of Annexin V-FITC and 10 μL of PI were added to the cells. The fixed cells were incubated at room temperature in the dark for 20 minutes, and then detect apoptosis status using a flow cytometer.

### Clone formation assay

The U251 and U87 glioma cells transfected with siRNAs were digested with trypsin and dispersed into single cell. Each experimental group was seeded with 500 cells per well in a 6-well plate. The cells were cultured for 14 days or until the majority of single clones contained more than 50 cells. Medium was changed every 3 days, and cell status was observed. Afterward, cells were washed once with PBS and fixed for 15 minutes. Then, an appropriate amount of Giemsa staining solution was applied for 10–30 minutes. Then, the staining solution was slowly washed away with running water, and the cells were air-dried. Images were taken using a camera.

### Development and validation of SEPN1-related risk score (SRS) and nomogram

To develop an SRS, we began by extracting long non-coding RNA (lncRNA) expression matrix from the TCGA-LGG cohort and calculating the Pearson’s correlation coefficients between SEPN1 expression and lncRNA expression. SEPN1-related lncRNAs were selected based on a correlation threshold of absolute r > 0.3 and p-value < 0.05. The TCGA-LGG cohort was stratified based on patient OS for division into training and validation sets, with a 7:3 ratio. In the training set, univariate CoxPHs identified significant lncRNAs associated with patient survival. The least absolute shrinkage and selection operator (LASSO), provided by the R package “glmnet” [37], refined our selection and identified the best candidate significant lncRNAs with optimal predictive power. The SRS for each patient was computed using the formula: SRS = ∑ [Expr(i) * Coef(i)], where Expr referred to the expression of each lncRNA (i) and Coef referred to the corresponding coefficients. Patients were classified into high and low SRS groups based on the median SRS level. Then, distribution plots of SRS, survival time and survival status, and KM curves were generated for the training set, validation set, and the entire TCGA-LGG cohort. RMST analyses and univariate and multivariate CoxPHs assessed the prognostic significance of the SRS in the TCGA-LGG cohort. The robustness and advantages of the SRS were validated in additional cohorts (CGGA-693, CGGA-325, and GSE16011) using the intersected set of SRS genes, and corresponding KM, RMST, and univariate and multivariate CoxPHs analyses were performed. We also explored the association between the SRS and pan-cancer prognosis across The Cancer Genome Atlas (TCGA) using univariate CoxPHs. To enhance the clinical applicability of our findings, we incorporated the SRS with clinical data to construct a predictive nomogram for the TCGA-LGG cohort and generated calibration curves to demonstrate the accuracy of the nomogram in predicting 1-year, 3-year, and 5-year OS probabilities.

### Association of SRS with glioma immunity and potential anti-glioma drugs screened based on SRS

To understand the relationship between the SRS and glioma immunity, we categorized patients into high and low SRS groups based on median SRS levels in the TCGA-LGG, CGGA-693, CGGA-325, and GSE16011 cohorts. Immune-related heatmaps were generated, illustrating immune and stromal scores computed using the ESTIMATE algorithm and scores for various immune cells derived through gene set variation analysis (GSVA) using curated immune signatures including 22 CIBERSORT signatures and 2 MCPcounter signatures [38]. For drug sensitivity predictions, we utilized the R package “pRRophetic” [39] to estimate half maximal inhibitory concentrations (IC50s) by constructing ridge regression models using gene expression profiles from the Genomics of Drug Sensitivity in Cancer (GDSC) database and expression data from our study cohorts. Drugs with significant associations to SRS (absolute Spearman’s rho > 0.3 and p-value < 0.05) were identified as potentially relevant therapeutics. To explore the functional connections among small molecules, genes, and disease states, we employed the Connectivity Map (CMap) [40] based on the eXtreme Sum (XSum) method [41] for its greater likelihood of meaningful results [42]. After grouping by median SRS levels, we identified significant DEGs and selected the top 300 upregulated and downregulated genes for XSum analyses in the four cohorts. The results were normalized from -1 to 1 and sorted in ascending order, with the top 5 drugs highlighted that showed the strongest potential for anti-glioma.

### Investigating the pan-cancer characteristics of selenoprotein family

SELENOF, SELENOH, SELENOI, SELENOK, SELENOM, SELENON (SEPN1), SELENOO, SELENOP, SELENOS, SELENOT, SELENOV, SELENOW, TXNRD1, TXNRD2, TXNRD3, GPX1, GPX2, GPX3, GPX4, GPX6, DIO1, DIO2, DIO3, MSRB1, and SEPHS2 are 25 selenoprotein genes in the human genome, which are critical for human health [43, 44]. To understand their roles in cancer, we investigated the association of selenoprotein family expression, prognosis, and CNV in pan-cancer. First, we calculated the expression patterns of selenoprotein families in tumor tissues and adjacent normal controls using data from the TCGA pan-cancer atlas. Next, we employed univariate CoxPHs to estimate the correlations between the expression levels of members of selenoprotein family and survival of patients in the pan-cancer cohort. Since CNVs can lead to genomic and molecular phenotypic heterogeneity affecting cancer development and progression [45], we also calculated the amplification and deletion rates of the selenoprotein family in pan-cancer using TCGA RNA-seq data and CNV data analyzed using GISTIC2.

### Statistical analysis

All statistical analyses and data visualization were performed using R software (version 4.3.1) and GraphPad Prism (version 8.0). The Wilcoxon test was used for comparisons between two groups, and the Kruskal-Wallis test for comparisons between multiple groups. Spearman’s and Pearson’s correlation were used to evaluate correlations between variables. The Chi-square test was used to assess the clinicopathological features of the patients. Survival analyses were performed using KM curves, and differences in OS were analyzed using the log-rank test and RMST method. Univariate and multivariate CoxPHs were constructed to describe the prognostic roles of genes, and 95% confidence intervals (CIs) were provided. All the cellular experiments were repeated for independently three times, and differences between two groups were analyzed using unpaired Student’s t-test while analysis among more than two groups was performed using one-way analysis of variance (one-way ANOVA) or two-way analysis of variance (two-way ANOVA). Statistical significance is expressed as follows: NS indicates no statistical significance, *p-value < 0.05, ** p-value < 0.01, *** p-value < 0.001.

## Results

### Pan-cancer analyses reveals that SEPN1 is significantly associated with the prognosis and TME of glioma

**Fig 1:**
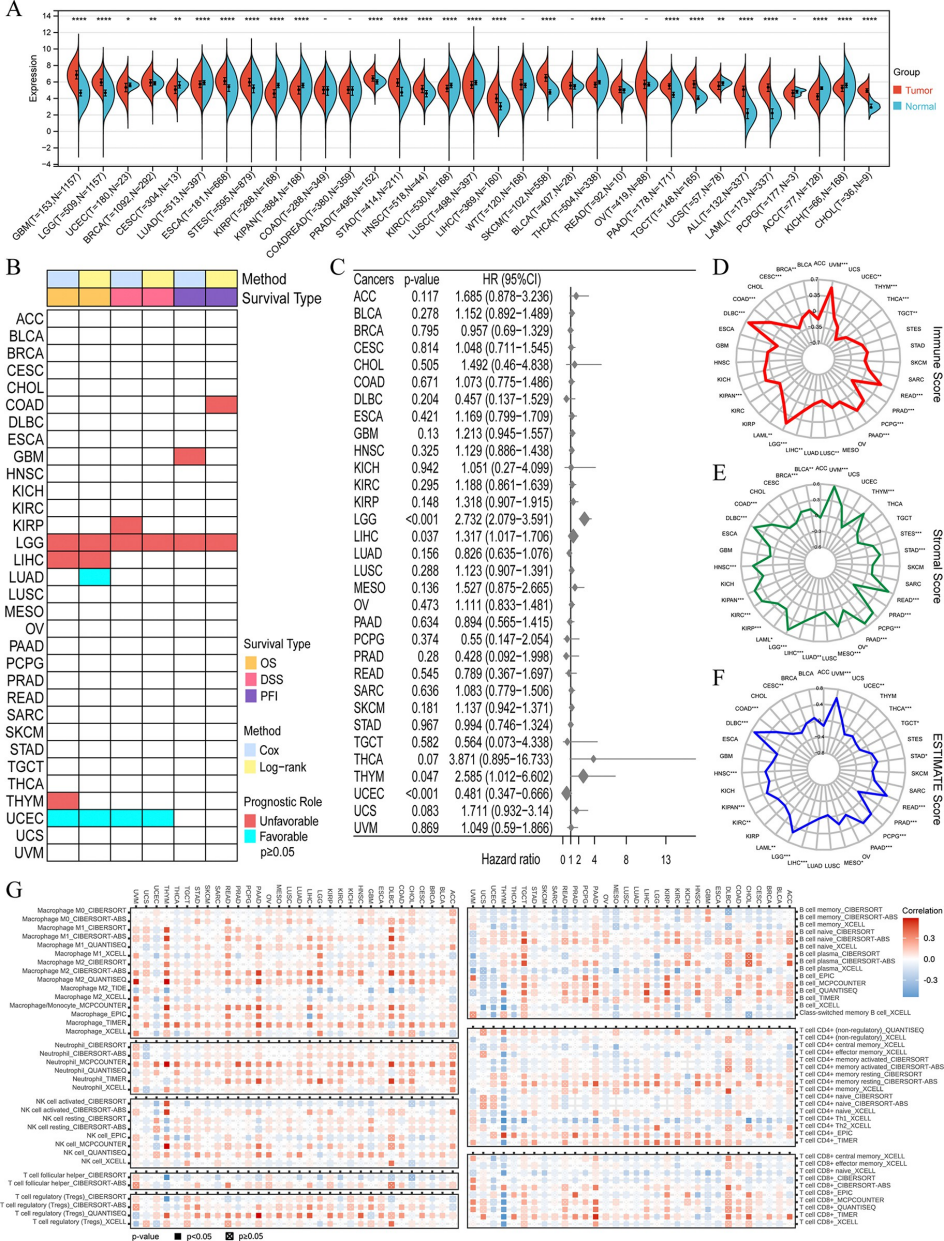
Pan-cancer expression characteristics of SEPN1 and its association with the TME. (A) Differential expression of SEPN1 in cancer and adjacent normal tissues across pan-cancer. (B) Prognostic impact of SEPN1 on OS, DSS, and PFI in pan-cancer. (C) Forest plot of SEPN1’s effect on OS in pan-cancer based on CoxPHs. (D-F) Correlations of SEPN1 expression with immune, stromal, and ESTIMATE scores in pan-cancer. (G) Correlations of SEPN1 expression with various immune cells across pan-cancer using multiple TME algorithms.

Differential analyses of SEPN1 expression in cancer samples and adjacent non-cancerous samples across different cancer types showed upregulation of SEPN1 in cancers such as GBM, LGG, ESCA, STES, and PAAD. In contrast, SEPN1 was found to be downregulated in cancers including CESC, KIRP, and ACC (Fig 1A and S1 Table in S1 File). To assess the prognostic implications of SEPN1, we employed the CoxPHs and the log-rank tests. Our findings indicate that in UCEC, SEPN1 serves as a favorable prognostic factor for OS and DSS. Conversely, in LGG, SEPN1 emerges as a risk prognostic factor, impacting OS, DSS, and progression-free interval (PFI) (Fig 1B and 1C). Furthermore, we utilized the ESTIMATE algorithm to investigate the relationships between SEPN1 expression and immune, stromal, and estimate scores in pan-cancer. This analysis uncovered the significant involvement of SEPN1 in the TME of multiple cancers. Notably, in LGG, UVM, DLBC, and other tumors, SEPN1 expression exhibited a significant positive correlation with all three scores (Fig 1D–1F). Additionally, we leveraged the TIMER2.0 database and various immune algorithms to assess the associations between SEPN1 expression and immune cells in pan-cancer. Our analysis revealed close relationships between SEPN1 and various immune cells. For instance, in LGG, SEPN1 may regulate the activation of macrophages and neutrophils while suppressing B cells (Fig 1G). In summary, the pan-cancer analyses highlight the potential significance of SEPN1 in the initiation and progression of glioma. Further investigations will focus on comprehensive analyses of roles of SEPN1 in glioma.

### SEPN1 as an independent prognostic risk factor for glioma patients

**Fig 2:**
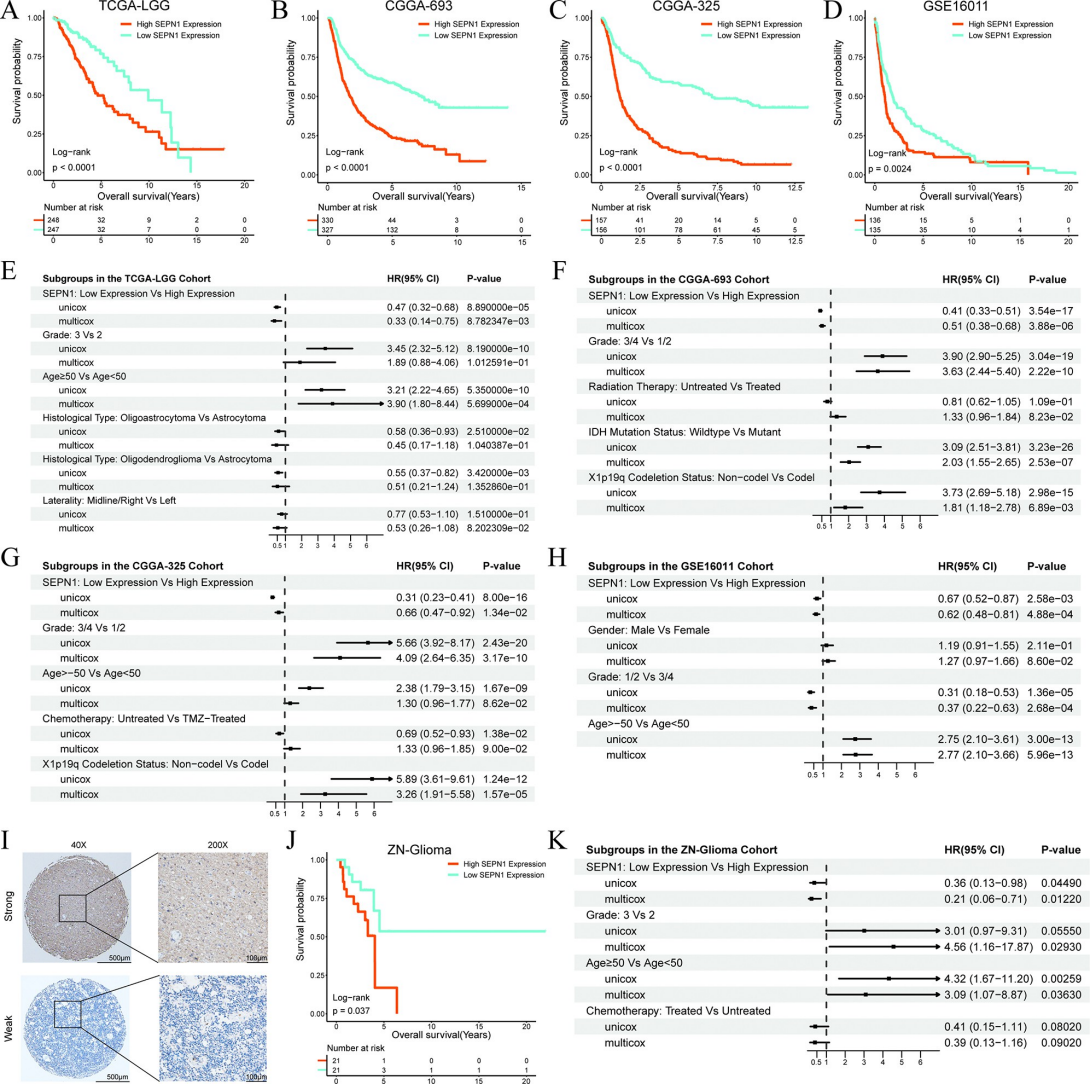
The role of SEPN1 in glioma prognosis. (A-D) KM curves of high and low SEPN1 expression groups in TCGA-LGG, CGGA-693, CGGA-325, and GSE16011 cohorts. (E-H) Forest plots of SEPN1 expression groups and other clinical indicators based on univariate and multivariate CoxPHs in TCGA-LGG, CGGA-693, CGGA-325, and GSE16011 cohorts. (I) Representative IHC staining images of high and low SEPN1 expression groups in the ZN-Glioma cohort. (J) KM curves of high and low SEPN1 expression groups in the ZN-Glioma cohort. (K) Forest plot of SEPN1 expression groups and other clinical indicators based on univariate and multivariate CoxPHs in the ZN-Glioma cohort.

Chi-square analyses in TCGA-LGG, CGGA-693, CGGA-325, and GSE16011 cohorts examined the distribution of clinicopathological features between high and low SEPN1 expression groups (S2-S5 Tables in S1 File). KM curves revealed that glioma patients with high SEPN1 expression experienced shorter OS time (Fig 2A–2D), which was further validated using RMST analyses (S6-S9 Tables in S1 File). Both univariate and multivariate CoxPHs supported high SEPN1 expression as an independent and significant prognostic risk factor for glioma patients (Fig 2E–2H). To strengthen our findings, we conducted analyses in an independent cohort, ZN-Glioma, which reaffirmed that high SEPN1 expression is an adverse prognostic factor for glioma patients (Fig 2I–2K and S10, S11 Tables in S1 File).

### SEPN1 exhibits high expression in malignant cells within glioma

**Fig 3:**
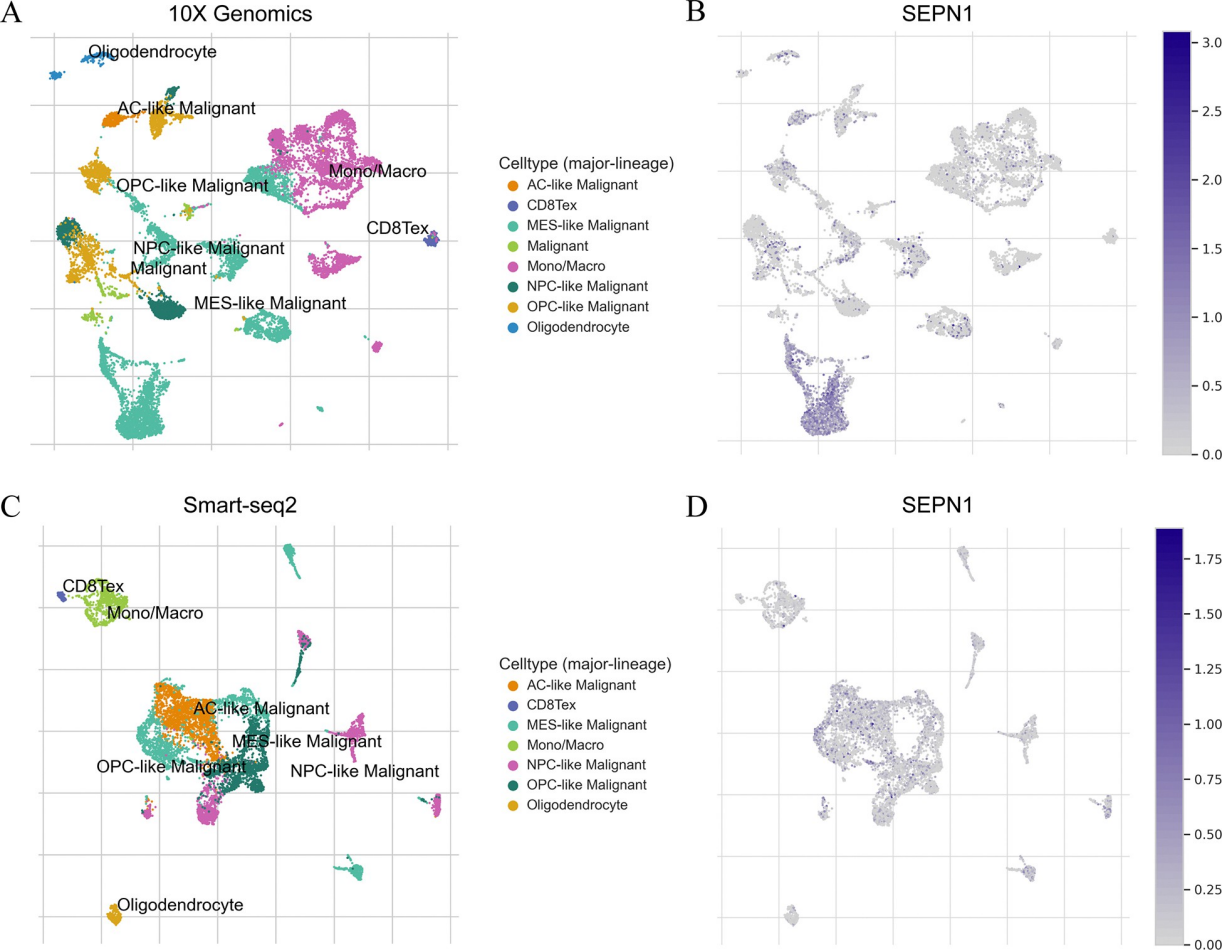
Expression characteristics of SEPN1 at the single-cell level in glioma. (A) Single-cell clustering in the GSE131928 cohort using 10X Genomics technology. (B) Expression of SEPN1 in cell clusters identified in the GSE131928 cohort using 10X Genomics technology. (C) Single-cell clustering in the GSE131928 cohort using Smart-seq2 technology. (D) Expression of SEPN1 in cell clusters identified in the GSE131928 cohort using Smart-seq2 technology.

Our pan-cancer analyses highlighted the upregulation of SEPN1 expression specifically in glioma. To further explore this observation, we analyzed the GSE131928 glioma scRNA-seq cohort which utilized both 10× Genomics and Smart-seq2 sequencing technologies. This analysis confirmed that SEPN1 is predominantly expressed in malignant cell populations within glioma tissues, suggesting a potential role in tumor aggressiveness and progression (Fig 3A–3D).

### SEPN1 in glioma exhibits multiple biological functions

**Fig 4:**
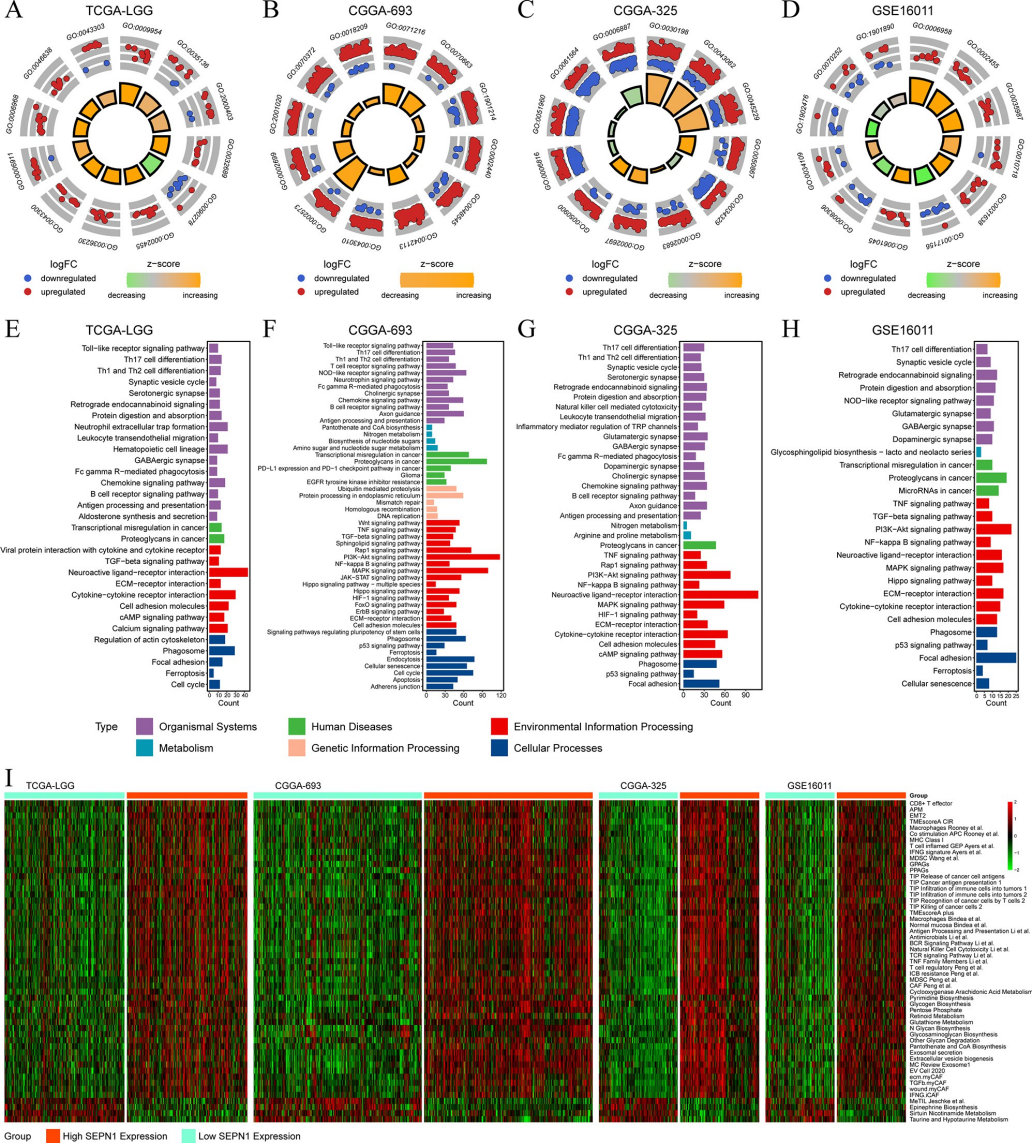
Functional enrichment analysis of SEPN1 in glioma. (A-D) Partial results of GO-based BP analysis in TCGA-LGG, CGGA-693, CGGA-325, and GSE16011 cohorts. (E-H) Partial results of KEGG-based analysis in CGGA-325, CGGA-693, GSE16011, and TCGA-LGG cohorts.

Enrichment analyses of DEGs between high and low SEPN1 expression groups provided insights into the potential biological functions of SEPN1 in glioma tissues. GO analyses of the BP module identified several processes associated with SEPN1, including negative regulation of type II interferon production, granulocyte activation, phagocytosis, positive regulation of cell activation, and negative regulation of immune system processes (Fig 4A–4D and S12-S15 Tables in S1 File). KEGG pathway enrichment analyses further unveiled the involvement of SEPN1 in various pathways, such as Th17 cell differentiation, Th1 and Th2 cell differentiation, neutrophil extracellular trap formation, chemokine signaling pathway, B cell receptor signaling pathway, transcriptional misregulation in cancer, proteoglycans in cancer, cell cycle, glioma, TNF signaling pathway, and apoptosis (Fig 4E–4H and S16-S19 Tables in S1 File). Moreover, utilizing ssGSEA with 255 published characteristic gene sets, we observed positive correlations between SEPN1 expression and macrophages, release of cancer cell antigens, infiltration of immune cells into tumors, and exosomal secretion, while negative correlations with sirtuin nicotinamide metabolism and taurine and hypotaurine metabolism (Fig 4I). These findings underscore the diverse biological functions associated with SEPN1 in glioma, encompassing immune-related processes, signaling pathways, and metabolic pathways, which could be pivotal in understanding tumor behavior and developing targeted therapies.

### High SEPN1 expression indicates high expression of multiple ICs in glioma

As shown in the S1-S8 Figs in S1 File, SEPN1 expression was positively correlated with multiple ICs. High IC expression implies that tumors can evade the immune system through the IC pathway, suggesting that IC inhibition therapies might be effective [46, 47]. Therefore, glioma patients exhibiting high SEPN1 expression were more likely to benefit from immunotherapy.

### SEPN1 deficiency suppresses glioblastoma (GBM) cell proliferation, and promotes G2/M cycle arrest and apoptosis

**Fig 5:**
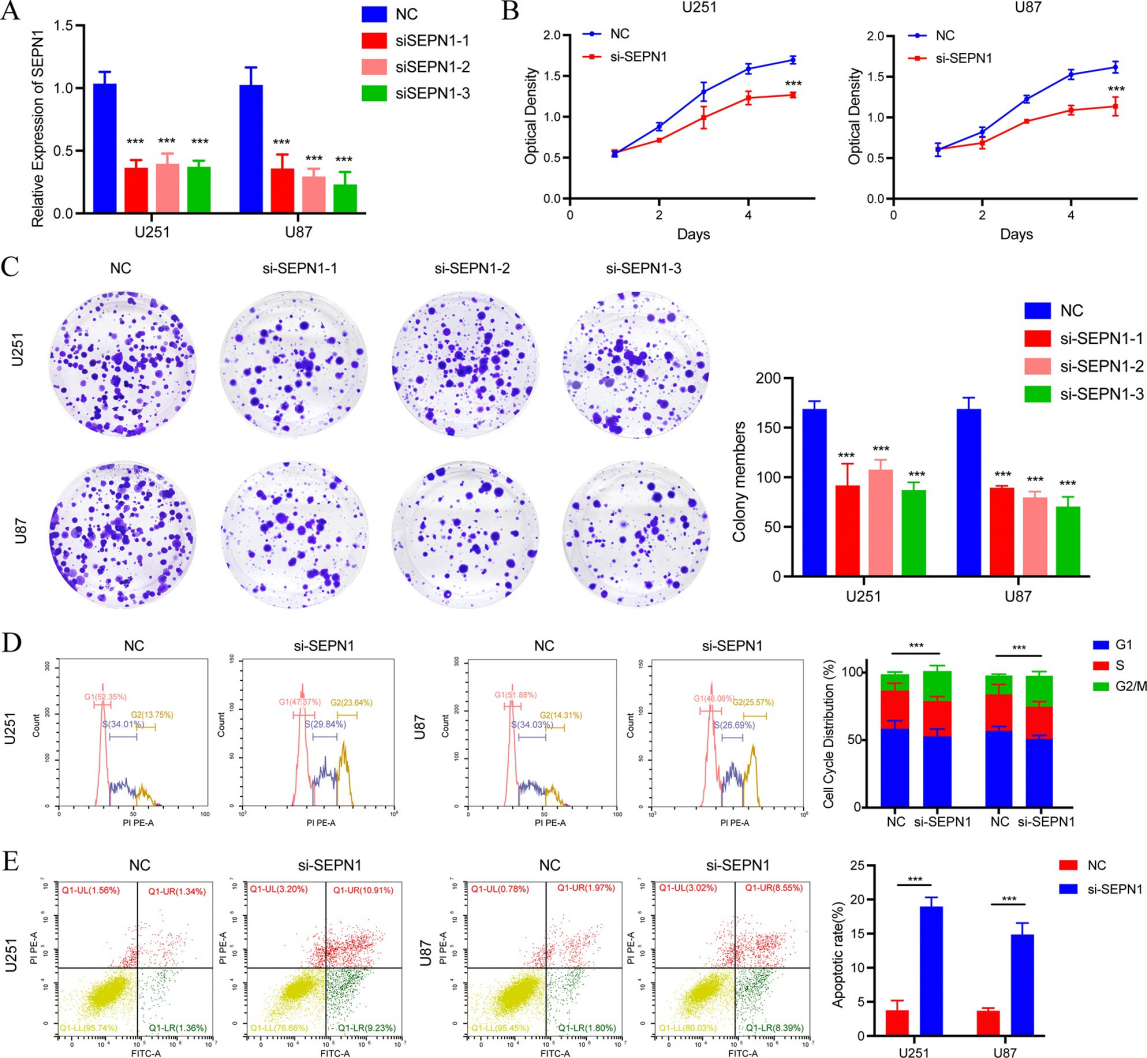
SEPN1 deficiency promotes glioblastoma cell proliferation, G2M cycle arrest and apoptosis. (A) The mRNA level of SEPN1 was weakened by si-SEPN1 transfection in U251 and U87 cell lines. (B) Knockdown of SEPN1 inhibited the proliferation of the U251 and U87 cell lines. (C) Clone formation assay showed the knockdown of SEPN1 could restrain the growth capacity of GBM cells. (D, E) The si-SEPN1 induced increased cell apoptosis and G2/M phase cycle arrest in U251 and U87 cell lines.

Given that SEPN1 might be associated with cell cycle and apoptosis in glioma, we decided to further assess the roles of SEPN1 in GBM cell biology. We initially verified the RNA knock-down efficiency of SEPN1 in U251 and U87 cell lines (Fig 5A). Subsequent MTT assay and clone formation assay confirmed that the deficiency of SEPN1 attenuated the proliferation capacity of both U251 and U87 cells (Fig 5B, 5C). To further elucidate the effects of SEPN1 on glioma cell cycle and apoptosis, we conducted flow cytometry analysis in GBM cells. Notably, compared to control group, the absence of SEPN1 significantly increased the cell proportion of G2/M cycle, as well as the numbers of apoptotic cells in U251 and U87 cells (Fig 5D and 5E). These findings collectively underscored the indispensable role of SEPN1 in regulating the in vitro proliferation, cell cycle and apoptosis behaviors of GBM cells.

### Development and validation of SRS and nomogram

**Fig 6:**
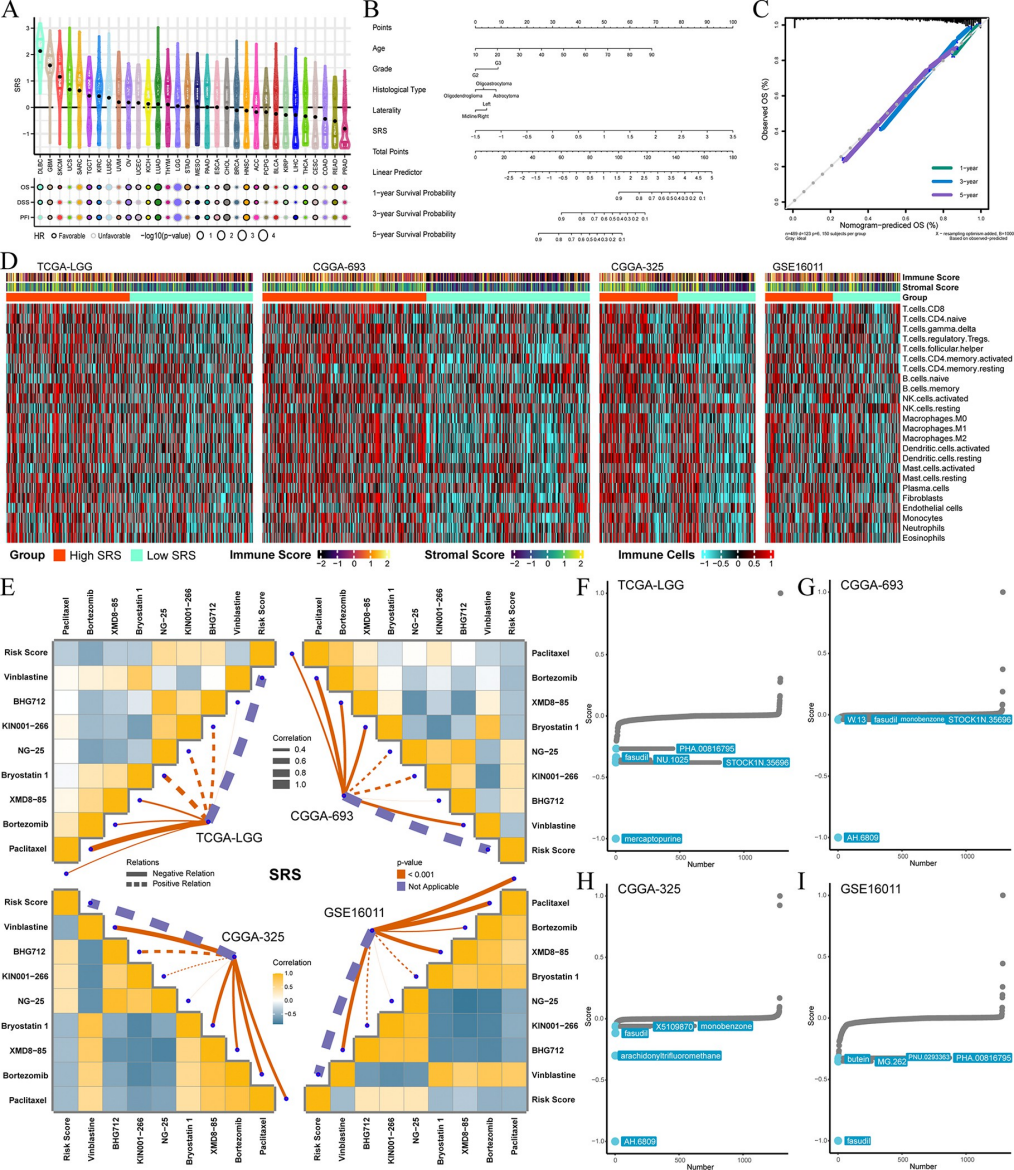
Applications based on SRS. (A) Association of SRS with prognosis in pan-cancer. (B) Construction of a nomogram based on SRS and other clinical indicators to comprehensively predict patient survival in the TCGA-LGG cohort. (C) Calibration curves plotted to evaluate the predictive performance of the nomogram. (D) Heatmaps showing the immune profiles in TCGA-LGG, CGGA-693, CGGA-325, and GSE16011 cohorts, including immune score, stromal score, and enrichment scores of 24 TME cell types. (E) Drugs with IC50s significantly correlated with SRS in the GDSC database for TCGA-LGG, CGGA-693, CGGA-325, and GSE16011 cohorts. (F-I) CMap analyses based on DEGs between high and low SRS groups, showing drugs with anti-glioma potential in TCGA-LGG, CGGA-693, CGGA-325, and GSE16011 cohorts.

In the TCGA-LGG cohort, we identified SEPN1-related lncRNAs and employed univariate CoxPHs to identify significant survival-related lncRNAs. Then, 19 lncRNAs with optimal lambda in the LASSO model were used to construct the SRS (S9A, S9B Fig & S20 Table in S1 File). Patients were divided into high and low SRS groups based on median SRS levels. High SRS groups exhibited a higher proportion of deceased patients across the training set, test set, and whole TCGA-LGG cohort (S9C-S9H Fig in S1 File). KM curves and RMST analyses confirmed that patients in high SRS groups had poorer prognosis (S9I-S9K Fig and S21-S23 Tables in S1 File). This finding was further validated in the CGGA-693, CGGA-325, and GSE16011 cohorts (S9L-S9N Fig and S24-S26 Tables in S1 File). Both univariate and multivariate CoxPHs demonstrated that high SRS is an independent prognostic factor for glioma patients (S9O-S9R Fig in S1 File). Additionally, we calculated the pan-cancer SRS using TCGA data and constructed univariate CoxPHs to analyze its associations with OS, DSS, and PFI (Fig 6A). The results revealed a significant correlation between SRS and the prognosis of LGG. Based on these findings, we developed a nomogram in the TCGA-LGG cohort, integrating SRS and clinical pathological information (Fig 6B). The nomogram score, which combines relevant factors, can predict the 1-year, 3-year, and 5-year survival rates. Calibration curves demonstrated good accuracy in predicting survival rates (Fig 6C).

### SRS and its relevance to glioma immunity and potential anti-glioma drugs

High SRS in glioma is associated with increased immune scores, stromal scores, and most immune cell scores, as indicated by heatmaps generated based on the ESTIMATE algorithm and curated immune signatures (Fig 6D). This suggests that patients with high SRS exhibit more substantial immune infiltration and stromal components within the TME. Analysis of the GDSC database revealed that SRS is negatively correlated with IC50s of certain drugs, including Vinblastine, BHG712, XMD8-85, Bortezomib, and Paclitaxel, suggesting that higher SRS expression may be associated with increased sensitivity to these drugs in glioma (Fig 6E). Furthermore, performing CMap analyses using DEGs between high and low SRS groups based on median SRS levels, we identified several potential anti-glioma drugs. Drugs with lower CMap scores are more likely to reverse the molecular features associated with high SRS, indicating their potential efficacy in treating glioma. Across the four cohorts, the CMap analyses highlighted drugs such as PHA.00816795, fasudil, NU.1025, STOCK1N.35696, mercaptopurine, W.13, monobenzone, AH.6809, X5109870, arachidonyltrifluoromethane, butein, PNU.0293363, and MG.262 as potential candidates (Fig 6F–6I). Notably, fasudil, STOCK1N.35696, monobenzone, and AH.6809 appeared in more than one cohort, underscoring their high potential for the treatment of glioma.

### Pan-cancer characteristics of selenoprotein family

**Fig 7:**
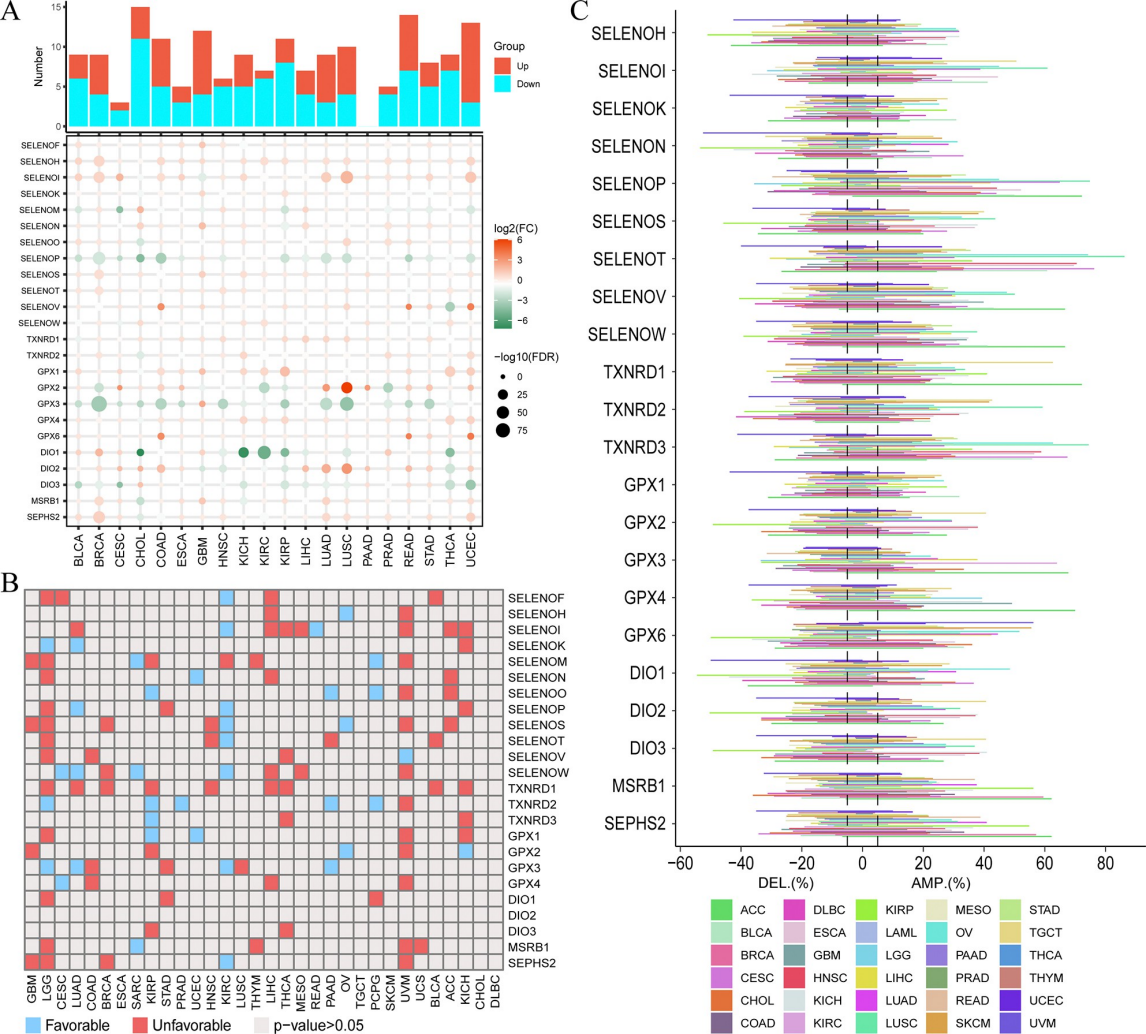
The pan-cancer analyses of selenoprotein family. (A) The heatmap displaying FC and false discovery rate (FDR) values illustrates the expression of the selenoprotein family in pan-cancer. The histogram illustrates the number of genes significantly differentially expressed. (B) Associations between selenoprotein family expression and pan-cancer prognosis were assessed using CoxPHs. A hazard ratio (HR)>1 indicates unfavorable associations, while an HR<1 indicates favorable associations. Grey indicates p-values>0.05. (C) Amplification and deletion rates of selenoprotein families in pan-cancer calculated using CNV data (threshold set at 0.05).

Finally, we performed a pan-cancer analysis of the selenoprotein family, revealing significant variations in their expression across different tumor types. For instance, SELENOI was mostly upregulated in tumor tissues, whereas GPX3 downregulated; the selenoprotein family tended to be downregulated in CHOL but upregulated in UCEC (Fig 7A). Univariate CoxPHs indicated that selenoproteins often acted as unfavorable prognostic factors in cancers such as LGG and UVM, but as protective factors in KIRC, and different selenoproteins could also exhibit varying prognostic roles within the same cancer type (Fig 7B). We also found that the selenoprotein family displays distinct CNV characteristics in pan-cancer, highlighting the heterogeneity of the selenoprotein family in pan-cancer. For example, SELENOP and SELENOT frequently showed amplification in multiple cancers (Fig 7C). Overall, our results provide a valuable reference for future studies investigating the relationship between selenoproteins and cancer.

## Discussion

Glioma originates from glial cells in the central nervous system and is the most common primary malignant brain tumor. Due to its invasiveness and high recurrence rate, it presents significant challenges for clinical treatment [48, 49]. Therefore, understanding the molecular drivers of gliomagenesis and identifying new therapeutic targets are crucial for improving patient prognosis. In this study, we found that SEPN1 plays an important role in glioma and is a promising biomarker.

SEPN1 is a protein that is critical in maintaining cellular redox balance and protecting cells from oxidative stress [50]. Previous studies have focused on the associations between SEPN1 and muscle diseases, such as the discovery of SEPN1 gene mutations linked to various congenital muscle diseases, including rigid spine muscular dystrophy and multiminicore disease [51, 52]. However, studies investigating the relationship between SEPN1 and cancer risk are scarce. Some studies have suggested that changes in SEPN1 expression might serve as a marker for the progression of colorectal adenomas to cancer [53], and SEPN1 was upregulated in the HepG2 liver cancer cell line compared to the normal liver cell line LO2 [54]. However, existing studies have not further explored the roles of SEPN1 in cancer, particularly its associations with pan-cancer and specifically glioma.

Therefore, our study conducted a pan-cancer analysis of SEPN1 expression, revealing its differential expression across various tumor types. Notably, SEPN1 was upregulated in GBM, LGG, ESCA, STES, and PAAD, while downregulated in CESC, KIRP, and ACC. These findings suggested that SEPN1 might play different roles in various cancers, thereby affecting tumor occurrence and development. Survival analysis further highlighted the prognostic significance of SEPN1, showing that it was a risk factor in LGG, negatively impacting OS, DSS, and PFI. Our pan-cancer TME analyses indicated that SEPN1 expression was significantly associated with immune, stromal, and ESTIMATE scores, as well as various immune cells in LGG and other tumors. These findings underscored the potential involvement of SEPN1 in modulating the TME and influencing tumor behaviors.

Based on the significant potential of SEPN1 in glioma prognosis as shown by pan-cancer analyses, we further focused on glioma. The TCGA-LGG, CGGA-693, CGGA-325, GSE16011, and ZN-GC cohorts all confirmed that high SEPN1 expression was associated with shorter OS in glioma patients, establishing it as an independent prognostic risk factor. ScRNA-seq analyses showed that SEPN1 was primarily expressed in the malignant cell populations within glioma tissues, suggesting its potential role in promoting tumor growth and progression. In recent years, the intricate interactions between tumor cells and the immune system have garnered significant attention. Our study found that SEPN1 was closely related to immune cell infiltration in glioma. Notably, SEPN1 expression was positively correlated with macrophages and neutrophils, and negatively correlated with B cells. These findings suggested that SEPN1 might regulate the activation and recruitment of immune cells in the TME, potentially affecting antitumor immune responses. Furthermore, to elucidate the functional roles of SEPN1 in glioma, we conducted enrichment analyses of DEGs between high and low SEPN1 expression groups. GO and KEGG analyses highlighted various biological functions associated with SEPN1 in glioma, including immune-related processes, signaling pathways, and metabolic pathways, which are crucial for understanding tumor behavior and developing targeted therapies. Notably, we found that SEPN1 was significantly associated with the cell cycle in glioma, and dysregulated cell proliferation was closely related to tumor progression [55]. Therefore, we conducted molecular assays which further confirmed that SEPN1 deficiency inhibited GBM cell proliferation, inducing G2/M cycle arrest and apoptosis.

To enhance the clinical applicability of our research findings, we developed and validated the SRS and associated nomogram for predicting glioma patient survival. The SRS constructed using SEPN1-related lncRNAs demonstrated strong prognostic value across multiple glioma cohorts. The nomogram integrating SRS and clinicopathological information showed good accuracy in predicting 1-year, 3-year, and 5-year survival rates, potentially aiding personalized risk assessment and treatment decision-making for glioma patients. Additionally, we explored the association between SRS and glioma immunity and identified potential anti-glioma drugs based on SRS. We found that high SRS was associated with increased immune infiltration and stromal components in the glioma TME. By analyzing drug sensitivity data from GDSC and CMap, we identified several potential anti-glioma drugs effective against high SRS, including the Rho kinase inhibitor fasudil, which has been reported to have anti-glioma effects [56]. Although the identified potential drugs require further validation, these results indicate the potential of leveraging SEPN1 for discovering and repurposing glioma drugs. Finally, we conducted a preliminary exploration of the characteristics of the selenoprotein family in pan-cancer to guide future related studies. Overall, these findings lay the foundation for future research on the immunological significance of SEPN1 in glioma and the development of SEPN1-based targeted therapies. Nonetheless, our study has some limitations. First, SEPN1 exhibited certain differences between LGG and GBM in the results and future studies could delve deeper to uncover the specific characteristics of SEPN1 across different grades of glioma. Second, further experiments are needed to validate the identified potential drugs.

## Conclusions

Our comprehensive pan-cancer analyses revealed the critical roles of SEPN1 in glioma prognosis and the TME. SEPN1 was significantly upregulated in glioma and served as an independent prognostic factor for glioma patients. ScRNA-seq analysis confirmed that SEPN1 was mainly expressed in malignant glioma cells. Enrichment analyses revealed multiple biological functions associated with SEPN1 in glioma, providing new insights into the complex mechanisms of glioma development and highlighting potential therapeutic targets. Our in vitro experiments validated the oncogenic effects of SEPN1 in glioma cells, showing that inhibition of SEPN1 expression suppressed cell proliferation and migration, induced apoptosis, and caused cell cycle arrest. Furthermore, we developed a robust SRS based on SEPN1-related lncRNAs, demonstrating strong prognostic value across multiple glioma cohorts. Using the SRS, we identified several potential anti-glioma drugs that warrant further investigation. Besides, the characteristics of selenoprotein family in pan-cancer were also explored. Overall, our study is the first to describe the roles of SEPN1 in glioma, establishing SEPN1 as a valuable biomarker for glioma and laying the foundation for future studies on the molecular mechanisms of SEPN1 in glioma pathogenesis and the development of SEPN1-targeted therapeutic strategies.

## Supporting information

S1 File Supplementary materials. (PDF)

S1 Graphical abstract (PNG)
