# Supplementary material for: Integrative analysis of SEPN1 in glioma: Prognostic roles, functional implications, and potential therapeutic interventions
Source: PLoS One. 2025 Feb 7;20(2):e0318501. doi: 10.1371/journal.pone.0318501 (PMC11805447; doi:10.1371/journal.pone.0318501)
Supplement: S1 File — (PDF) [file pone.0318501.s001.pdf]

Integrative Analysis of SEPN1 in Glioma: Prognostic Roles, Functional Implications, and Potential Therapeutic Interventions

Supplementary Materials

Content

Figure S1 3

Figure S2 4

Figure S3 5

Figure S4 6

Figure S5 7

Figure S6 8

Figure S7 9

Figure S8 10

Figure S9 11

Table S1 12

Table S2 13

Table S3 14

Table S4 15

Table S5 16

Table S6 17

Table S7 17

Table S8 18

Table S9 18

Table S10 19

Table S11 20

Table S12 21

Table S13 66

Table S14 121

Table S15 198

Table S16 227

Table S17 229

Table S18 232

Table S19 235

Table S20 237

Table S21 237

Table S22 238

Table S23 238

Table S24 239

Table S25 239

Table S26 240

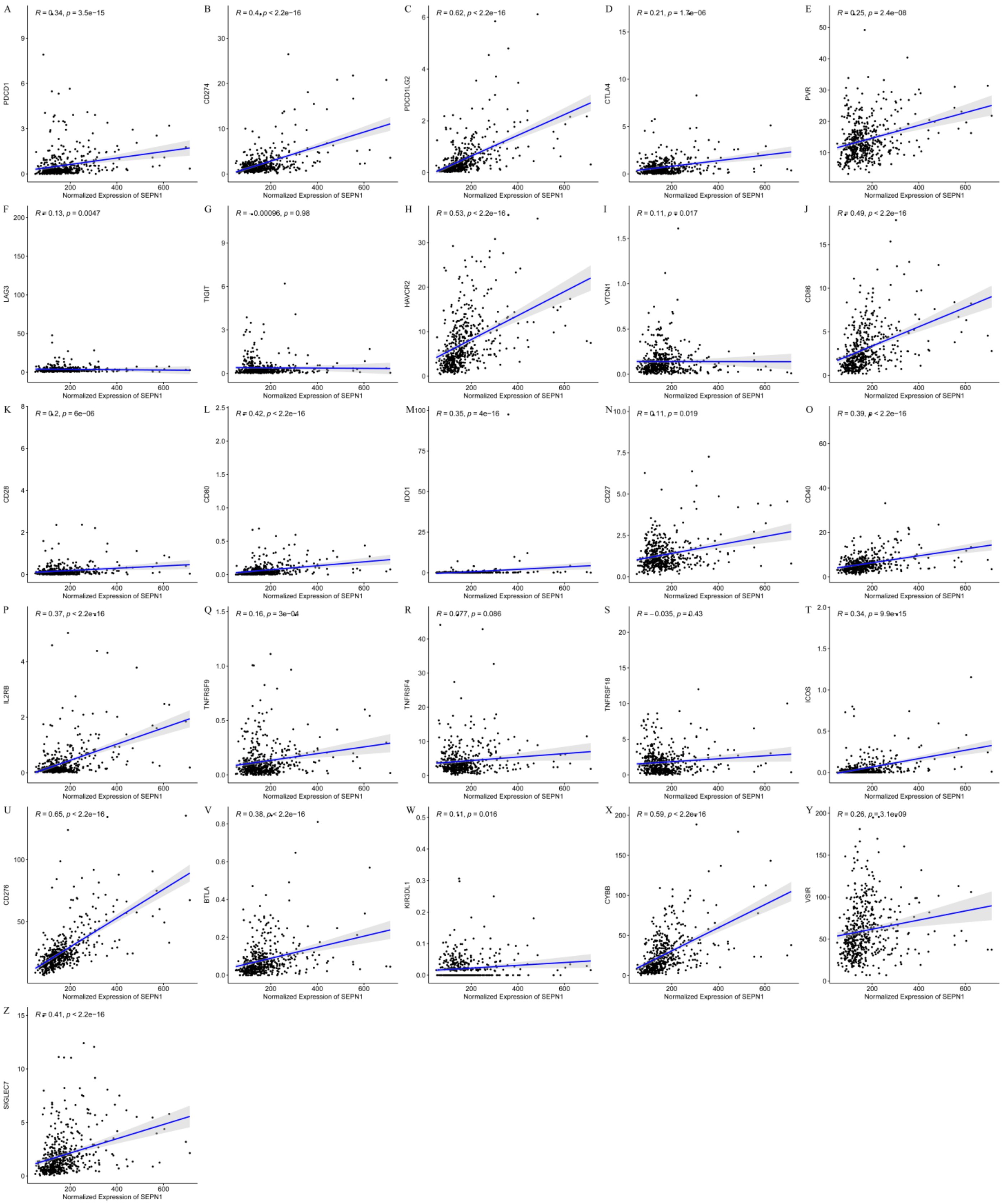

Figure S1 Scatter plots showing the correlations between expression of SEPN1 and immune checkpoints in TCGA-LGG.

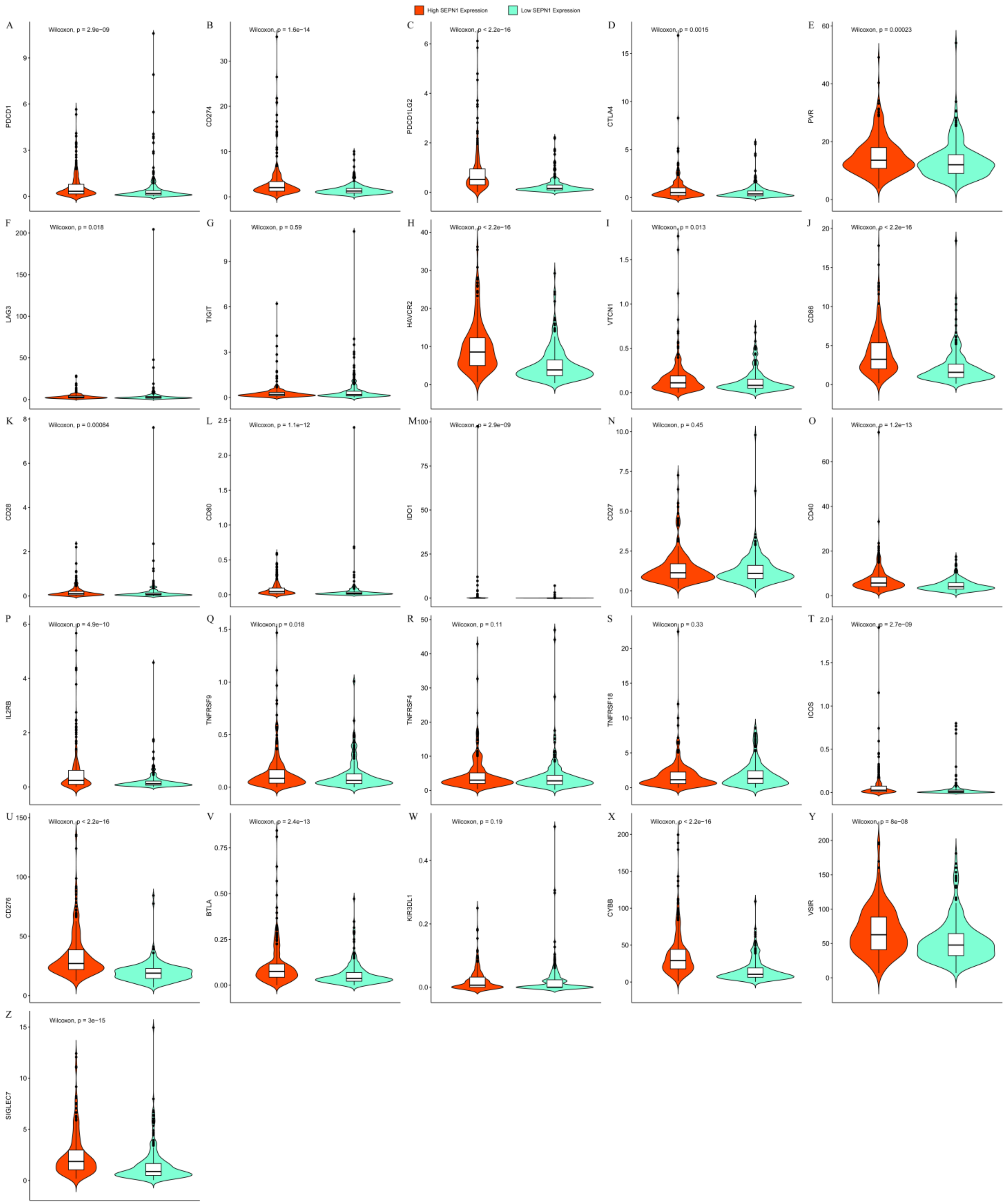

Figure S2 Violin plots showing the distributions of immune checkpoints expression between high and low SEPN1 expression groups in TCGA-LGG.

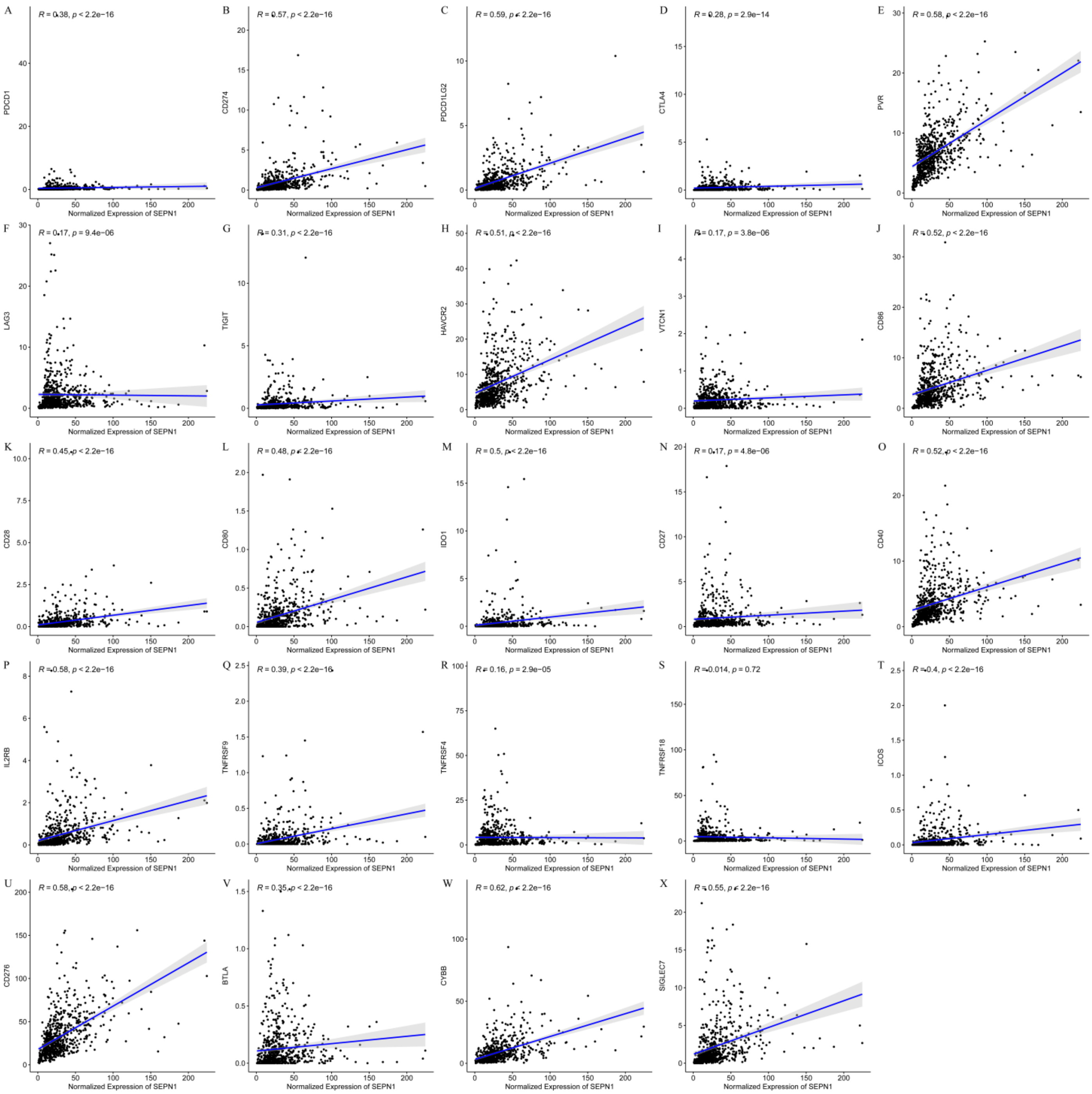

Figure S3 Scatter plots showing the correlations between expression of SEPN1 and immune checkpoints in CGGA-693.

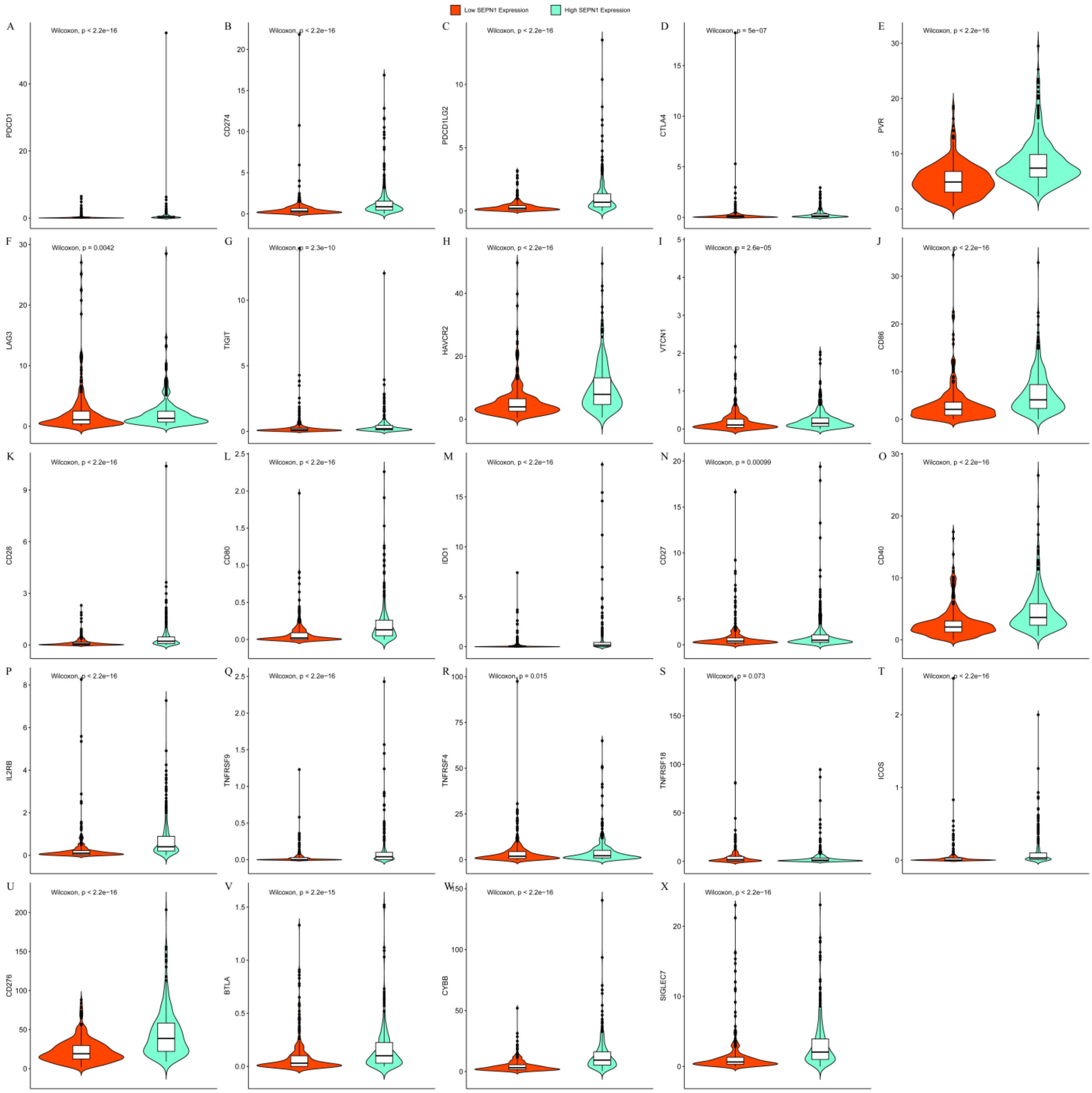

Figure S4 Violin plots showing the distributions of immune checkpoints expression between high and low SEPN1 expression groups in CGGA-693.

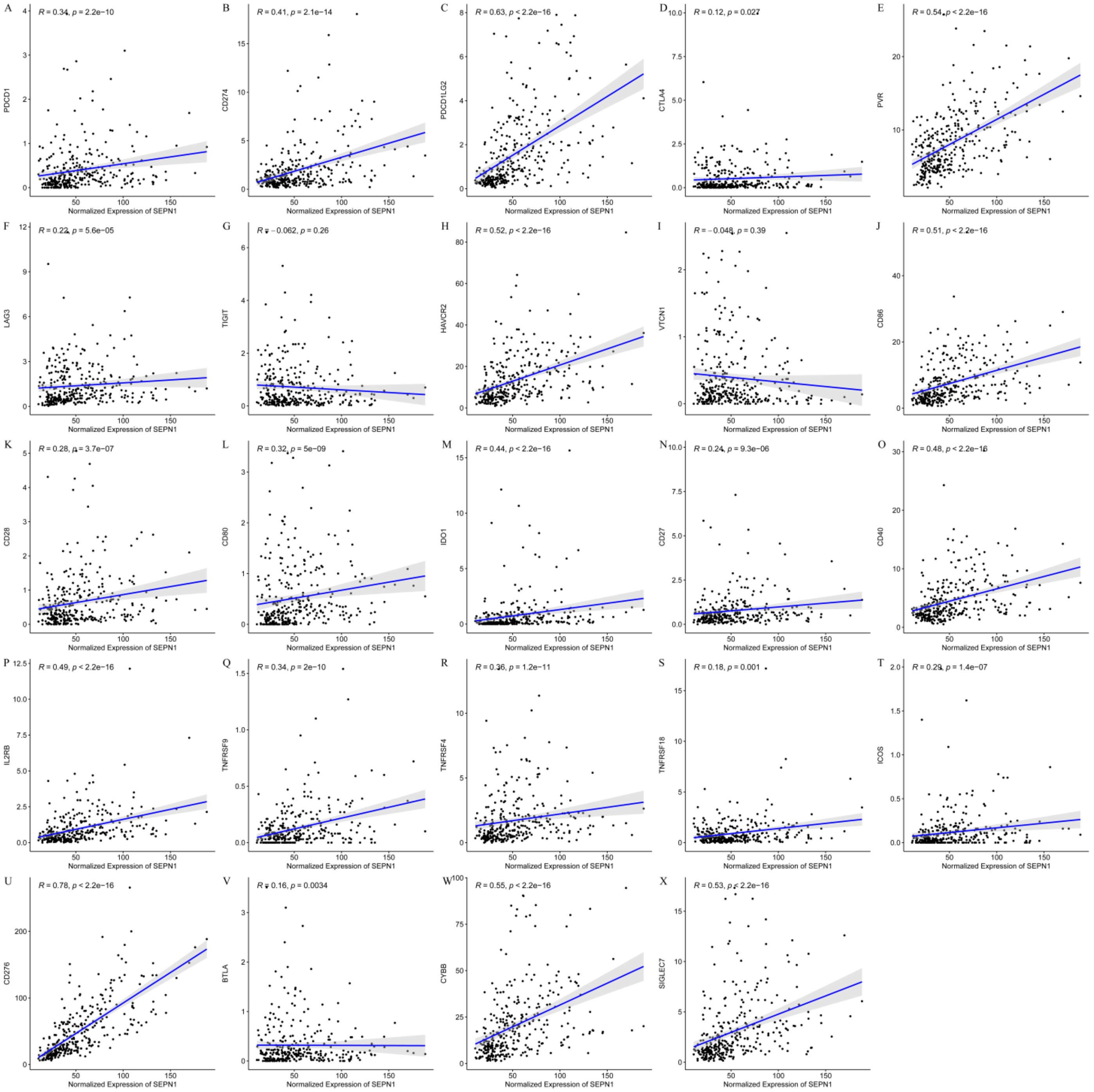

Figure S5 Scatter plots showing the correlations between expression of SEPN1 and immune checkpoints in CGGA-325.

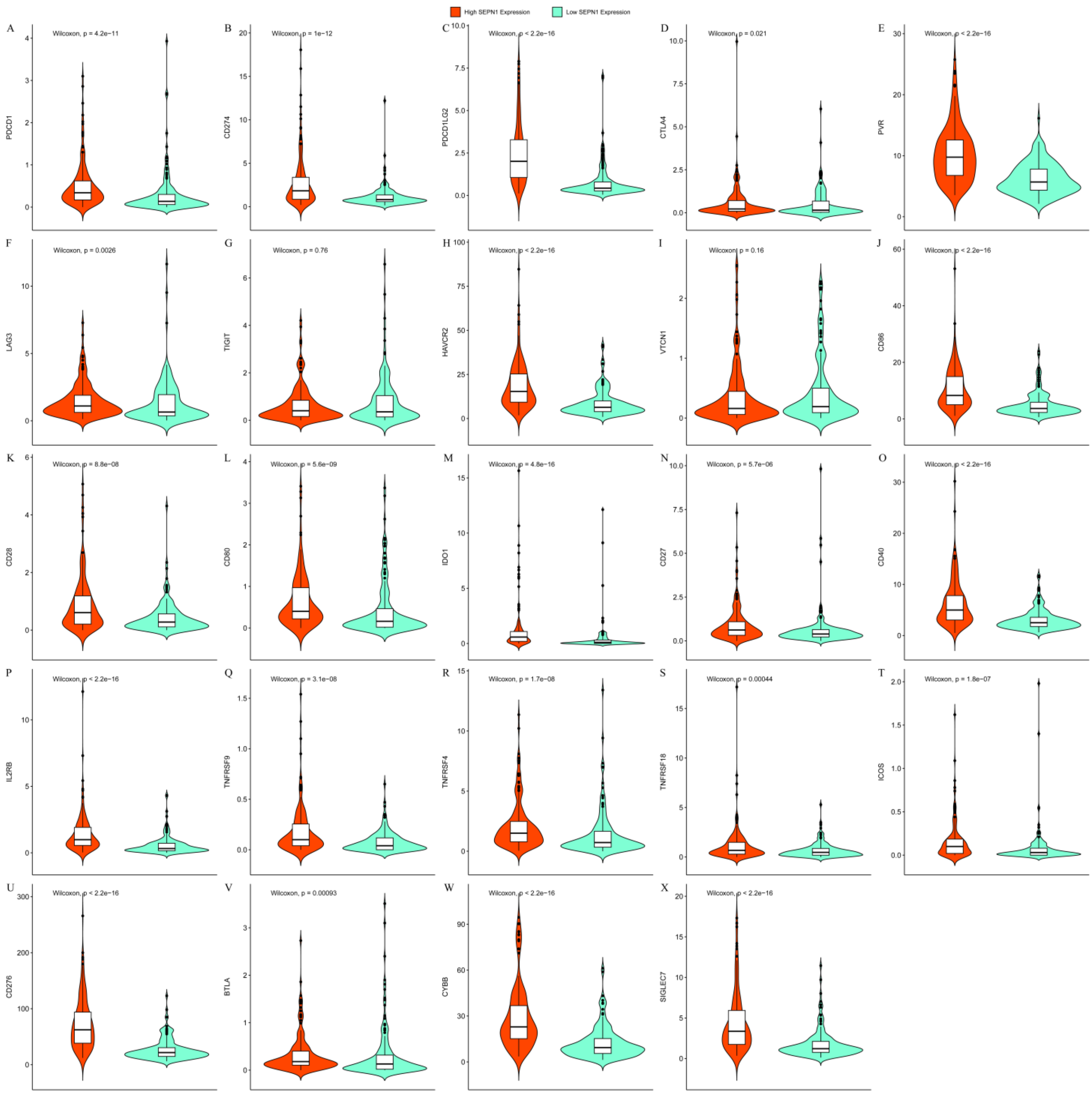

Figure S6 Violin plots showing the distributions of immune checkpoints expression between high and low SEPN1 expression groups in CGGA-325.

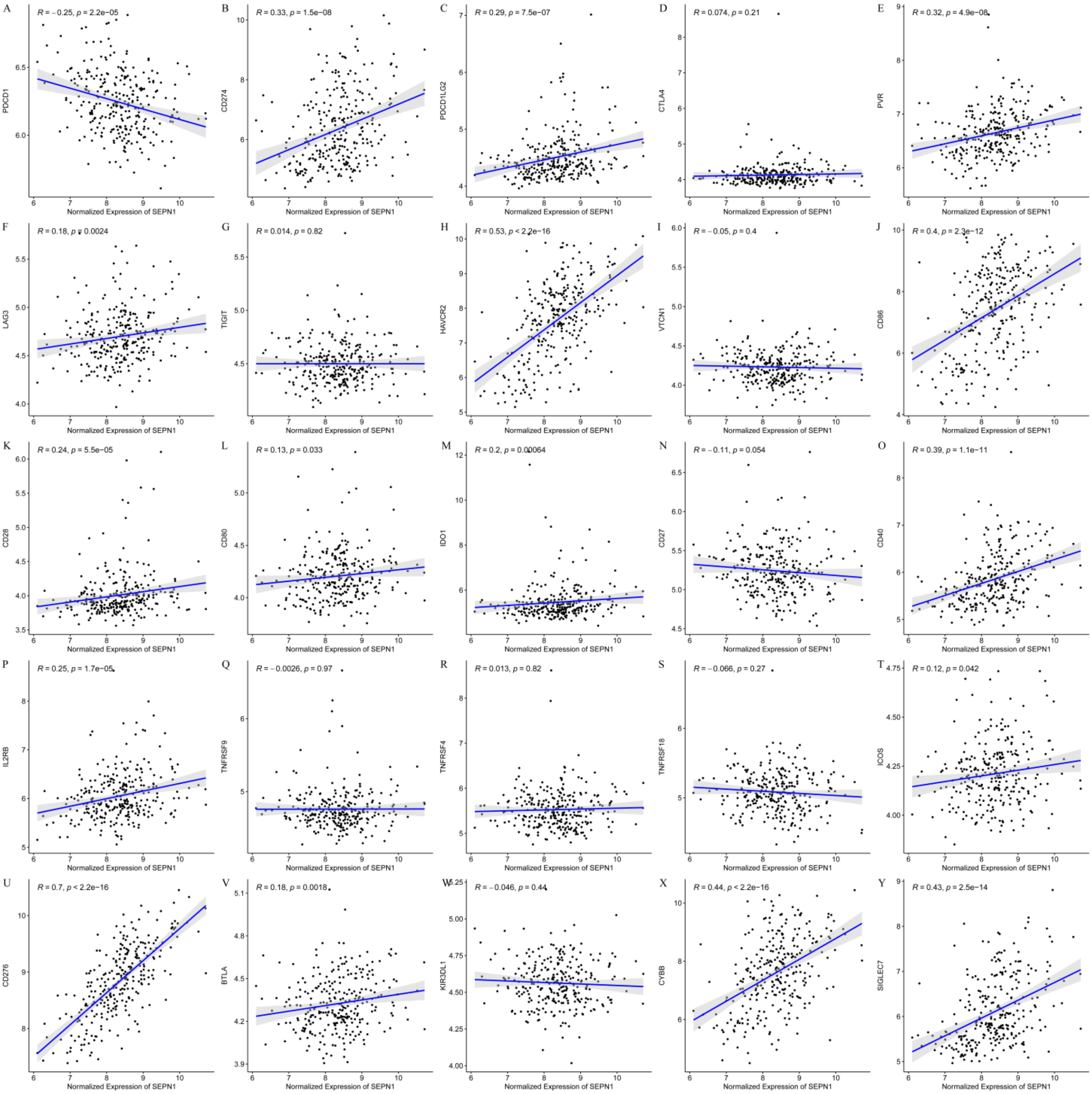

Figure S7 Scatter plots showing the correlations between expression of SEPN1 and immune checkpoints in GSE16011.

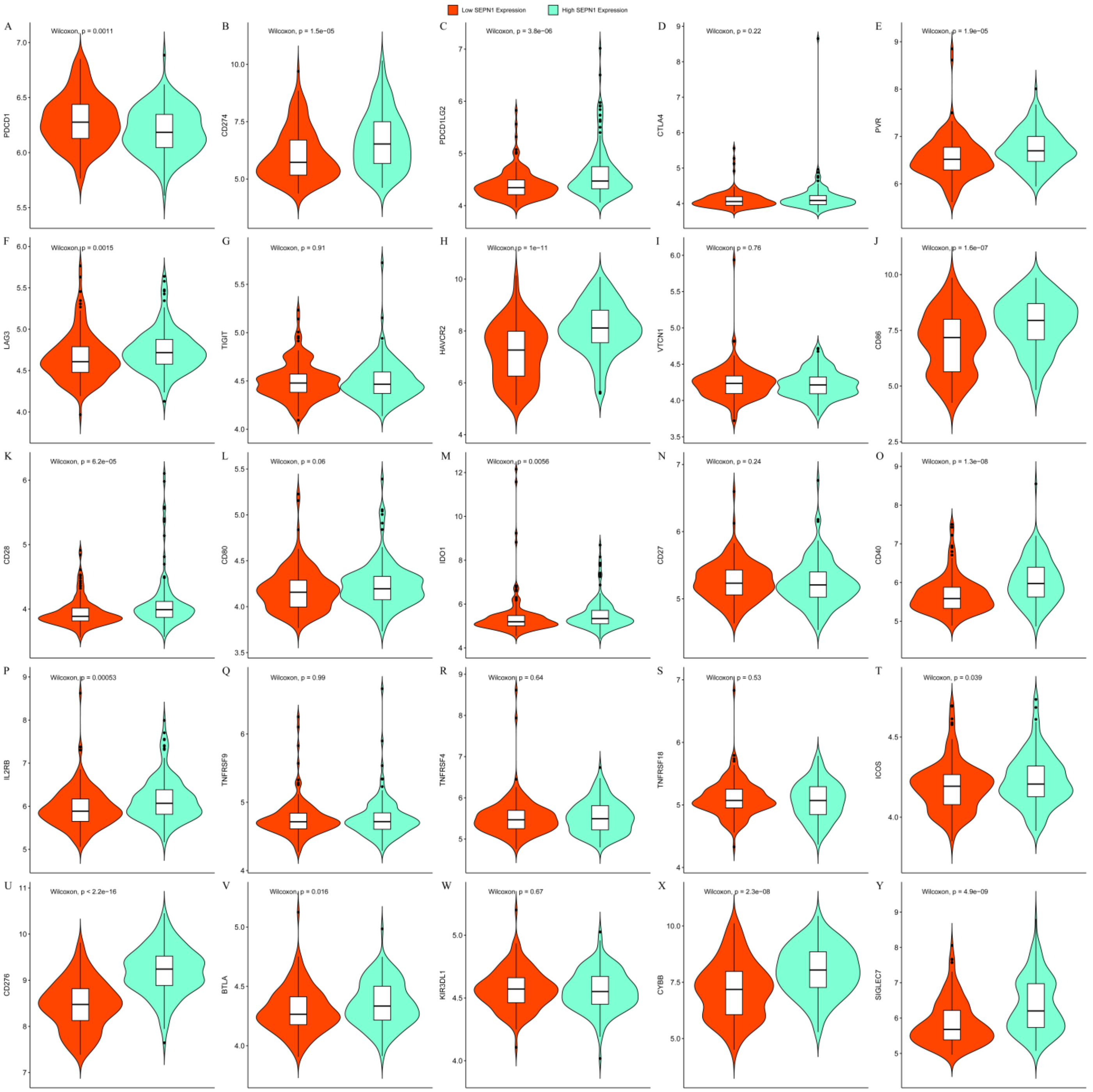

Figure S8 Violin plots showing the distributions of immune checkpoints expression between high and low SEPN1 expression groups in GSE16011.

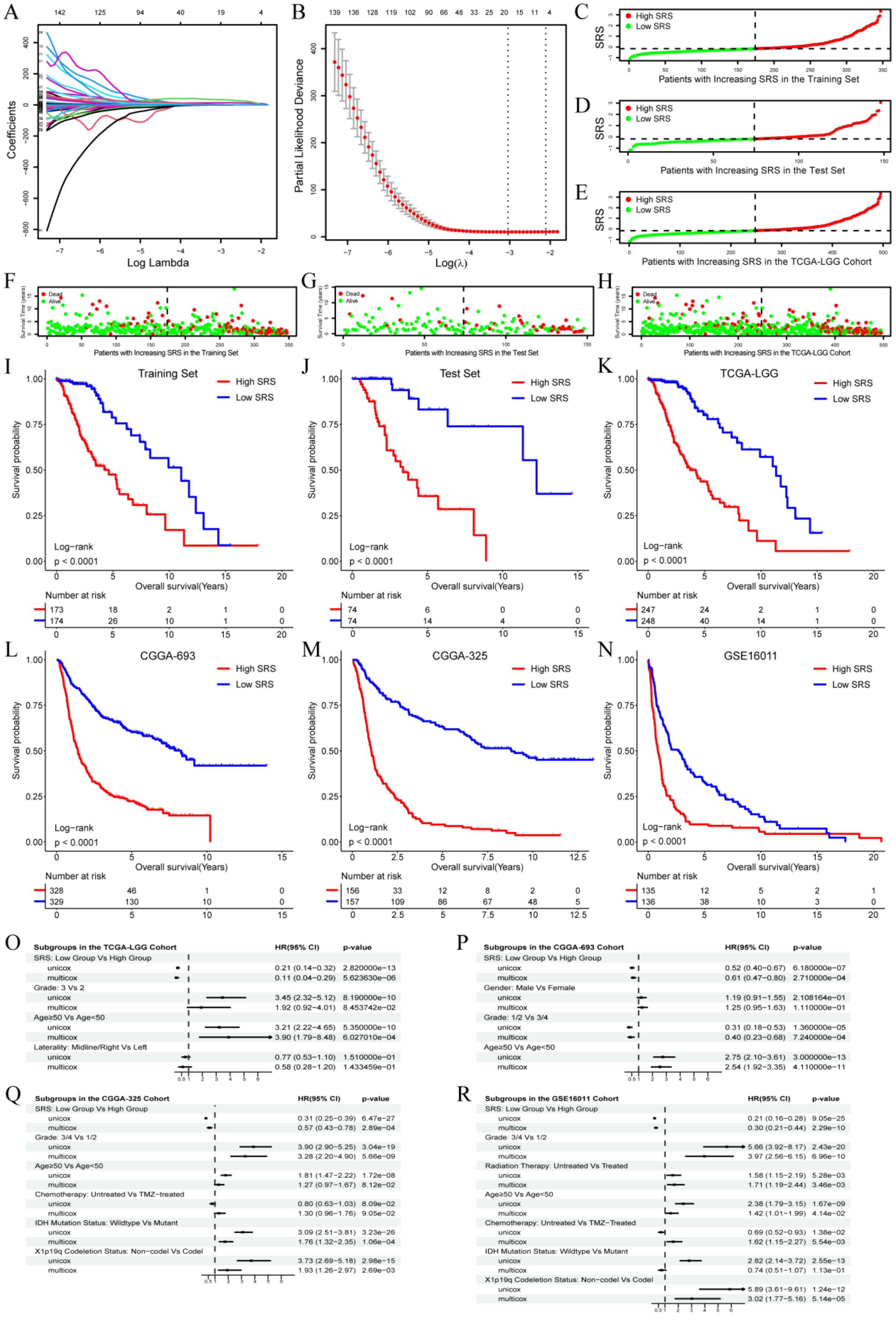

Figure S9 Development and validation of the SRS. (A) Tenfold cross-validation to select the best parameter (lambda) for the SRS. (B) Identification of 19 prognostic genes with the minimum λ value. (C-H) Distribution of SRS, survival time, and survival status of patients in the training set, testing set, and TCGA-LGG cohort; the dashed line represents the optimal cutoff value between high and low SRS groups. (I-N) KM curves of high and low SRS groups in the training set, testing set, TCGA-LGG, CGGA-693, CGGA-325, and GSE16011 cohorts. (O-R) Forest plots of SRS groups and other clinical indicators based on univariate and multivariate CoxPHs in TCGA-LGG, CGGA-693, CGGA-325, and GSE16011 cohorts.

Table S1 Pan-cancer cohorts and corresponding diseases**.**

Cohort	Disease
TCGA-ACC	Adrenocortical carcinoma
TCGA-BLCA	Bladder Urothelial Carcinoma
TCGA-BRCA	Breast invasive carcinoma
TCGA-CESC	Cervical squamous cell carcinoma and endocervical adenocarcinoma
TCGA-CHOL	Cholangiocarcinoma
TCGA-COAD	Colon adenocarcinoma
TCGA-COADREAD	Colon adenocarcinoma/ Rectum adenocarcinoma Esophageal carcinoma
TCGA-DLBC	Lymphoid Neoplasm Diffuse Large B-cell Lymphoma
TCGA-ESCA	Esophageal carcinoma
TCGA-GBM	Glioblastoma multiforme
TCGA-HNSC	Head and Neck squamous cell carcinoma
TCGA-KICH	Kidney Chromophobe
TCGA-KIPAN	Pan-kidney cohort (KICH+KIRC+KIRP)
TCGA-KIRC	Kidney renal clear cell carcinoma
TCGA-KIRP	Kidney renal papillary cell carcinoma
TCGA-LAML	Acute Myeloid Leukemia
TCGA-LGG	Brain Lower Grade Glioma
TCGA-LIHC	Liver hepatocellular carcinoma
TCGA-LUAD	Lung adenocarcinoma
TCGA-LUSC	Lung squamous cell carcinoma
TCGA-MESO	Mesothelioma
TCGA-OV	Ovarian serous cystadenocarcinoma
TCGA-PAAD	Pancreatic adenocarcinoma
TCGA-PCPG	Pheochromocytoma and Paraganglioma
TCGA-PRAD	Prostate adenocarcinoma
TCGA-READ	Rectum adenocarcinoma
TCGA-SARC	Sarcoma
TCGA-STAD	Stomach adenocarcinoma
TCGA-SKCM	Skin Cutaneous Melanoma
TCGA-STES	Stomach and Esophageal carcinoma
TCGA-TGCT	Testicular Germ Cell Tumors
TCGA-THCA	Thyroid carcinoma
TCGA-THYM	Thymoma
TCGA-UCEC	Uterine Corpus Endometrial Carcinoma
TCGA-UCS	Uterine Carcinosarcoma
TCGA-UVM	Uveal Melanoma
TARGET-OS	Osteosarcoma
TARGET-ALL	Acute Lymphoblastic Leukemia
TARGET-WT	High-Risk Wilms Tumor

Table S2 Clinicopathologic information of the patients in TCGA-LGG.

Variables	SEPN1 Expression Groups		P-value
	**High (N=248)**	**Low (N=247)**	
**Age (Years)**			
Mean (SD)	42.0 (13.5)	44.1 (13.3)	0.0777
Median [Min, Max]	40.5 [14.0, 74.0]	42.0 [18.0, 87.0]	
**Gender**			
Female	115 (46.4%)	106 (42.9%)	0.495
Male	133 (53.6%)	141 (57.1%)	
**Histological Type**			
Astrocytoma	131 (52.8%)	56 (22.7%)	
Oligoastrocytoma	65 (26.2%)	60 (24.3%)	<0.001
Oligodendroglioma	52 (21.0%)	131 (53.0%)	
**Race**			
American Indian or Alaska Native	0 (0%)	1 (0.4%)	
Asian	4 (1.6%)	4 (1.6%)	
Black or African American	9 (3.6%)	12 (4.9%)	0.661
White	233 (94.0%)	222 (89.9%)	
Missing	2 (0.8%)	8 (3.2%)	
**Radiation Therapy**			
No	43 (17.3%)	75 (30.4%)	
Yes	76 (30.6%)	63 (25.5%)	0.00517
Missing	129 (52.0%)	109 (44.1%)	
**Family History of Cancer**			
No	95 (38.3%)	103 (41.7%)	
Yes	63 (25.4%)	66 (26.7%)	0.969
Missing	90 (36.3%)	78 (31.6%)	
**Laterality**			
Left	120 (48.4%)	121 (49.0%)	
Midline	3 (1.2%)	3 (1.2%)	0.996
Right	122 (49.2%)	121 (49.0%)	
Missing	3 (1.2%)	2 (0.8%)	
**IDH1 Mutation Found**			
No	23 (9.3%)	10 (4.0%)	
Yes	31 (12.5%)	60 (24.3%)	<0.001
Missing	194 (78.2%)	177 (71.7%)	
**Grade**			
G2	101 (40.7%)	137 (55.5%)	
G3	146 (58.9%)	110 (44.5%)	0.00162
Missing	1 (0.4%)	0 (0%)	

Table S3 Clinicopathologic information of the patients in CGGA-693**.**

Variables	SEPN1 Expression Groups		P-value
	High (N=330)	Low (N=327)	
**Age (Years)**			
Mean (SD)	43.8 (13.0)	43.1 (11.8)	
Median [Min, Max]	43.0 [11.0, 73.0]	43.0 [13.0, 76.0]	0.529
Missing	0 (0%)	1 (0.3%)	
**Gender**			
Female	142 (43.0%)	141 (43.1%)	1
Male	188 (57.0%)	186 (56.9%)	
**Radiation Therapy**			
Treated	262 (79.4%)	239 (73.1%)	
Untreated	53 (16.1%)	78 (23.9%)	0.0206
Missing	15 (4.5%)	10 (3.1%)	
**Chemotherapy**			
TMZ-treated	247 (74.8%)	233 (71.3%)	
Untreated	72 (21.8%)	84 (25.7%)	0.29
Missing	11 (3.3%)	10 (3.1%)	
**IDH Mutation Status**			
Mutant	155 (47.0%)	178 (54.4%)	
Wildtype	170 (51.5%)	106 (32.4%)	<0.001
Missing	5 (1.5%)	43 (13.1%)	
**X1p19q Codeletion Status**			
Codel	35 (10.6%)	102 (31.2%)	
Non-codel	288 (87.3%)	166 (50.8%)	<0.001
Missing	7 (2.1%)	59 (18.0%)	
**Grade**			
2	65 (19.7%)	107 (32.7%)	
3	112 (33.9%)	136 (41.6%)	<0.001
4	153 (46.4%)	84 (25.7%)	

Table S4 Clinicopathologic information of the patients in CGGA-325**.**

Variables	SEPN1 Expression Groups		P-value
	High (N=157)	Low (N=156)	
**Age (Years)**			
Mean (SD)	45.7 (12.1)	40.6 (11.3)	<0.001
Median [Min, Max]	45.0 [11.0, 73.0]	40.0 [8.00, 79.0]	
**Gender**			
Female	56 (35.7%)	60 (38.5%)	0.693
Male	101 (64.3%)	96 (61.5%)	
**Radiation Therapy**			
Treated	112 (71.3%)	129 (82.7%)	
Untreated	37 (23.6%)	25 (16.0%)	0.0868
Missing	8 (5.1%)	2 (1.3%)	
**Chemotherapy**			
TMZ-treated	101 (64.3%)	89 (57.1%)	
Untreated	49 (31.2%)	61 (39.1%)	0.188
Missing	7 (4.5%)	6 (3.8%)	
**IDH Mutation Status**			
Mutant	53 (33.8%)	114 (73.1%)	
Wildtype	103 (65.6%)	42 (26.9%)	<0.001
Missing	1 (0.6%)	0 (0%)	
**X1p19q Codeletion Status**			
Codel	1 (0.6%)	61 (39.1%)	
Non-codel	150 (95.5%)	93 (59.6%)	<0.001
Missing	6 (3.8%)	2 (1.3%)	
**Grade**			
2	22 (14.0%)	76 (48.7%)	
3	36 (22.9%)	38 (24.4%)	<0.001
4	95 (60.5%)	42 (26.9%)	
Missing	4 (2.5%)	0 (0%)	

Table S5 Clinicopathologic information of the patients in GSE16011.

Variables	SEPN1 Expression Groups		P-value
	High (N=136)	Low (N=135)	
**Age (Years)**			
Mean (SD)	50.2 (15.3)	50.0 (14.2)	
Median [Min, Max]	51.6 [11.7, 80.7]	50.2 [15.0, 81.2]	0.875
Missing	1 (0.7%)	0 (0%)	
**Gender**			
Female	46 (33.8%)	51 (37.8%)	0.581
Male	90 (66.2%)	84 (62.2%)	
**Histological Type**			
A	14 (10.3%)	15 (11.1%)	
GBM	97 (71.3%)	58 (43.0%)	
OA	11 (8.1%)	17 (12.6%)	<0.001
OD	8 (5.9%)	43 (31.9%)	
PA	6 (4.4%)	2 (1.5%)	
**Type of Surgery**			
Completeresection	43 (31.6%)	43 (31.9%)	
Openbiopsy	3 (2.2%)	4 (3.0%)	
Partialresection	71 (52.2%)	72 (53.3%)	0.965
Stereotacticbiopsy	17 (12.5%)	15 (11.1%)	
Missing	2 (1.5%)	1 (0.7%)	
**Grade**			
1	6 (4.4%)	2 (1.5%)	
2	5 (3.7%)	18 (13.3%)	<0.001
3	28 (20.6%)	57 (42.2%)	
4	97 (71.3%)	58 (43.0%)	

Table S6 Comparing RMST between high and low SEPN1 expression groups in TCGA-LGG**.**

Year	High SEPN1 Expression RMST	Low SEPN1 Expression RMST	Estimation	LCI	UCI	P-value
1	11.604	11.809	-0.205	-0.464	0.054	0.120645425
2	21.873	23.121	-1.248	-2.071	-0.425	0.002965829
3	30.650	33.828	-3.178	-4.793	-1.562	0.000115504
4	38.111	44.010	-5.899	-8.474	-3.324	7.13E-06
5	44.389	53.576	-9.187	-12.888	-5.486	1.14E-06
6	49.855	62.463	-12.608	-17.604	-7.612	7.57E-07
7	54.634	70.697	-16.062	-22.500	-9.624	1.01E-06
8	59.105	78.263	-19.157	-27.198	-11.117	3.02E-06
9	63.034	84.712	-21.678	-31.388	-11.968	1.21E-05
10	66.433	91.053	-24.621	-36.166	-13.075	2.92E-05
11	69.606	96.658	-27.052	-40.521	-13.583	8.26E-05
12	71.917	101.651	-29.734	-45.050	-14.418	0.000141832
13	73.730	104.690	-30.960	-47.790	-14.131	0.000311411
14	75.544	105.907	-30.363	-48.387	-12.340	0.000960514
15	77.357	106.315	-28.958	-47.898	-10.019	0.002728138

Abbreviations: RMST, restricted mean survival time; LCI, lower limit of 95% confidence interval; UCI, upper limit of 95% confidence interval.

Table S7 Comparing RMST between high and low SEPN1 expression groups in CGGA-693**.**

Year	High SEPN1 Expression RMST	Low SEPN1 Expression RMST	Estimation	LCI	UCI	P-value
1	10.278	11.299	-1.021	-1.405	-0.637	1.88E-07
2	16.907	20.551	-3.644	-4.739	-2.549	6.98E-11
3	21.683	28.743	-7.061	-8.904	-5.217	6.11E-14
4	25.474	36.239	-10.765	-13.366	-8.163	5.10E-16
5	28.693	43.412	-14.718	-18.087	-11.349	1.10E-17
6	31.460	50.235	-18.775	-22.910	-14.639	5.69E-19
7	34.053	56.599	-22.546	-27.462	-17.630	2.50E-19
8	36.301	62.437	-26.136	-31.818	-20.453	1.97E-19
9	38.317	67.794	-29.477	-35.947	-23.006	4.31E-19
10	39.939	72.937	-32.998	-40.302	-25.695	8.34E-19
11	41.092	78.081	-36.989	-45.164	-28.814	7.43E-19
12	42.130	83.224	-41.094	-50.249	-31.939	1.40E-18

Abbreviations: RMST, restricted mean survival time; LCI, lower limit of 95% confidence interval; UCI, upper limit of 95% confidence interval.

Table S8 Comparing RMST between high and low SEPN1 expression groups in CGGA-325**.**

Year	High SEPN1 Expression RMST	Low SEPN1 Expression RMST	Estimation	LCI	UCI	P-value
1	9.960	11.002	-1.042	-1.668	-0.415	0.001125167
2	15.183	20.228	-5.045	-6.693	-3.396	2.01E-09
3	18.737	28.611	-9.874	-12.581	-7.168	8.59E-13
4	21.265	35.986	-14.721	-18.427	-11.016	6.90E-15
5	23.191	43.067	-19.875	-24.579	-15.171	1.22E-16
6	24.858	49.917	-25.060	-30.776	-19.344	8.45E-18
7	26.251	56.372	-30.121	-36.812	-23.429	1.12E-18
8	27.446	62.266	-34.820	-42.448	-27.192	3.66E-19
9	28.501	68.080	-39.579	-48.156	-31.001	1.51E-19
10	29.294	73.577	-44.283	-53.756	-34.810	5.09E-20
11	30.083	78.762	-48.679	-59.057	-38.301	3.81E-20
12	30.872	83.920	-53.047	-64.369	-41.726	4.19E-20

Abbreviations: RMST, restricted mean survival time; LCI, lower limit of 95% confidence interval; UCI, upper limit of 95% confidence interval.

Table S9 Comparing RMST between high and low SEPN1 expression groups in GSE16011**.**

Year	High SEPN1 Expression RMST	Low SEPN1 Expression RMST	Estimation	LCI	UCI	P-value
1	8.853	9.837	-0.985	-1.875	-0.094	0.030197554
2	13.224	16.664	-3.441	-5.474	-1.407	0.000914588
3	16.294	21.865	-5.571	-8.730	-2.413	0.000545127
4	18.336	26.198	-7.862	-12.019	-3.705	0.000209991
5	20.070	29.884	-9.814	-14.947	-4.682	0.000178384
6	21.708	33.118	-11.411	-17.509	-5.313	0.000244905
7	23.087	35.963	-12.876	-19.874	-5.877	0.000311152
8	24.445	38.390	-13.945	-21.820	-6.069	0.000519716
9	25.803	40.369	-14.566	-23.279	-5.853	0.001051115
10	27.091	41.957	-14.866	-24.357	-5.375	0.002141041
11	28.061	43.117	-15.055	-25.171	-4.939	0.003534342
12	29.031	43.978	-14.946	-25.655	-4.237	0.006228763

Abbreviations: RMST, restricted mean survival time; LCI, lower limit of 95% confidence interval; UCI, upper limit of 95% confidence interval.

Table S10 Clinicopathologic information of the patients in ZN-Glioma**.**

	SEPN1 Expression Groups		P-value
	High (N=21)	Low (N=21)	
**Age (Years)**			
Mean (SD)	48.1 (10.4)	44.2 (11.3)	0.252
Median [Min, Max]	49.0 [35.0, 69.0]	42.0 [28.0, 70.0]	
**Gender**			
Female	9 (42.9%)	7 (33.3%)	0.751
Male	12 (57.1%)	14 (66.7%)	
**Radiotherapy**			
Mean (SD)	0.762 (0.436)	0.810 (0.402)	0.715
Median [Min, Max]	1.00 [0, 1.00]	1.00 [0, 1.00]	
**Chemotherapy**			
Mean (SD)	0.524 (0.512)	0.524 (0.512)	1
Median [Min, Max]	1.00 [0, 1.00]	1.00 [0, 1.00]	
**Ki67**			
Mean (SD)	9.33 (6.54)	9.52 (6.59)	0.926
Median [Min, Max]	8.00 [0, 20.0]	8.00 [0, 30.0]	
**Grade**			
Mean (SD)	2.52 (0.512)	2.71 (0.463)	0.213
Median [Min, Max]	3.00 [2.00, 3.00]	3.00 [2.00, 3.00]	

Table S11 Comparing RMST between high and low SEPN1 expression groups in ZN-Glioma**.**

Year	High SEPN1 Expression RMST	Low SEPN1 Expression RMST	Estimation	LCI	UCI	P-value
1	11.224	11.938	-0.714	-1.482	0.053	0.068230846
2	20.310	22.795	-2.486	-5.601	0.629	0.117813113
3	28.442	32.797	-4.355	-10.182	1.473	0.143004992
4	34.903	42.333	-7.429	-16.084	1.226	0.092490948
5	37.116	49.618	-12.503	-23.678	-1.327	0.028323384
6	39.143	56.047	-16.904	-31.836	-1.973	0.026486405
7	39.886	62.476	-22.590	-40.718	-4.462	0.014590874
8	39.886	68.904	-29.019	-50.051	-7.986	0.006846792
9	39.886	75.333	-35.447	-59.662	-11.232	0.004116954
10	39.886	81.761	-41.876	-69.457	-14.295	0.002922463
11	39.886	88.190	-48.304	-79.374	-17.234	0.002310268
12	39.886	94.618	-54.733	-89.378	-20.088	0.001958928
13	39.886	101.047	-61.161	-99.443	-22.879	0.001740071
14	39.886	107.476	-67.590	-109.555	-25.625	0.001595199
15	39.886	113.904	-74.019	-119.701	-28.336	0.001494767
16	39.886	120.333	-80.447	-129.874	-31.020	0.001422597
17	39.886	126.761	-86.876	-140.068	-33.683	0.001369235
18	39.886	133.190	-93.304	-150.280	-36.329	0.00132886
19	39.886	139.618	-99.733	-160.505	-38.961	0.001297728
20	39.886	146.047	-106.161	-170.742	-41.581	0.001273344
21	39.886	152.476	-112.590	-180.988	-44.192	0.001253994
22	39.886	158.904	-119.019	-191.242	-46.795	0.001238467

Abbreviations: RMST, restricted mean survival time; LCI, lower limit of 95% confidence interval; UCI, upper limit of 95% confidence interval.

Table S12 GO terms based on DEGs between high and low SEPN1 expression groups in TCGA-LGG.

ONTOLOGY	ID	Description	GeneRatio	BgRatio	P-value	P-adjust	Q-value	Count
BP	GO:0002443	leukocyte mediated immunity	57/751	401/18614	8.77E-17	5.50E-13	4.13E-13	57
BP	GO:0050867	positive regulation of cell activation	56/751	394/18614	1.66E-16	5.50E-13	4.13E-13	56
BP	GO:0048706	embryonic skeletal system development	30/751	129/18614	3.86E-15	8.52E-12	6.41E-12	30
BP	GO:0007159	leukocyte cell-cell adhesion	55/751	415/18614	6.71E-15	9.65E-12	7.26E-12	55
BP	GO:0002696	positive regulation of leukocyte activation	52/751	377/18614	7.29E-15	9.65E-12	7.26E-12	52
BP	GO:0045785	positive regulation of cell adhesion	59/751	482/18614	2.47E-14	2.22E-11	1.67E-11	59
BP	GO:0002399	MHC class II protein complex assembly	12/751	16/18614	2.68E-14	2.22E-11	1.67E-11	12
BP	GO:0002503	peptide antigen assembly with MHC class II protein complex	12/751	16/18614	2.68E-14	2.22E-11	1.67E-11	12
BP	GO:0022409	positive regulation of cell-cell adhesion	46/751	321/18614	6.67E-14	4.91E-11	3.69E-11	46
BP	GO:0002460	adaptive immune response based on somatic recombination of immune receptors built from immunoglobulin superfamily domains	45/751	311/18614	8.82E-14	5.67E-11	4.26E-11	45
BP	GO:0002274	myeloid leukocyte activation	39/751	240/18614	9.42E-14	5.67E-11	4.26E-11	39
BP	GO:0016064	immunoglobulin mediated immune response	30/751	145/18614	1.08E-13	5.96E-11	4.48E-11	30
BP	GO:1903037	regulation of leukocyte cell-cell adhesion	50/751	378/18614	1.28E-13	6.52E-11	4.90E-11	50
BP	GO:0019724	B cell mediated immunity	30/751	148/18614	1.91E-13	9.03E-11	6.79E-11	30
BP	GO:1903039	positive regulation of leukocyte cell-cell adhesion	41/751	273/18614	3.27E-13	1.44E-10	1.08E-10	41
BP	GO:0002396	MHC protein complex assembly	12/751	20/18614	1.60E-12	6.11E-10	4.59E-10	12
BP	GO:0002501	peptide antigen assembly with MHC protein complex	12/751	20/18614	1.60E-12	6.11E-10	4.59E-10	12
BP	GO:0050863	regulation of T cell activation	48/751	377/18614	1.66E-12	6.11E-10	4.59E-10	48
BP	GO:0002449	lymphocyte mediated immunity	42/751	300/18614	1.85E-12	6.43E-10	4.84E-10	42
BP	GO:0048704	embryonic skeletal system morphogenesis	23/751	95/18614	2.58E-12	8.53E-10	6.42E-10	23

BP	GO:0070661	leukocyte proliferation	45/751	348/18614	4.90E-12	1.55E-09	1.16E-09	45
BP	GO:0042098	T cell proliferation	34/751	213/18614	6.20E-12	1.87E-09	1.40E-09	34
BP	GO:0022407	regulation of cell-cell adhesion	55/751	491/18614	7.06E-12	2.03E-09	1.53E-09	55
BP	GO:0050900	leukocyte migration	48/751	393/18614	7.57E-12	2.09E-09	1.57E-09	48
BP	GO:0051251	positive regulation of lymphocyte activation	43/751	327/18614	8.42E-12	2.23E-09	1.68E-09	43
BP	GO:0046651	lymphocyte proliferation	41/751	306/18614	1.42E-11	3.62E-09	2.72E-09	41
BP	GO:0002495	antigen processing and presentation of peptide antigen via MHC class II	14/751	35/18614	2.84E-11	6.39E-09	4.81E-09	14
BP	GO:0050870	positive regulation of T cell activation	36/751	249/18614	2.84E-11	6.39E-09	4.81E-09	36
BP	GO:0032943	mononuclear cell proliferation	41/751	313/18614	2.94E-11	6.39E-09	4.81E-09	41
BP	GO:0071706	tumor necrosis factor superfamily cytokine production	31/751	190/18614	2.99E-11	6.39E-09	4.81E-09	31
BP	GO:1903555	regulation of tumor necrosis factor superfamily cytokine production	31/751	190/18614	2.99E-11	6.39E-09	4.81E-09	31
BP	GO:0001819	positive regulation of cytokine production	53/751	489/18614	5.99E-11	1.24E-08	9.32E-09	53
BP	GO:0002504	antigen processing and presentation of peptide or polysaccharide antigen via MHC class II	14/751	37/18614	6.92E-11	1.34E-08	1.01E-08	14
BP	GO:0019886	antigen processing and presentation of exogenous peptide antigen via MHC class II	13/751	31/18614	7.13E-11	1.34E-08	1.01E-08	13
BP	GO:0032640	tumor necrosis factor production	30/751	185/18614	7.30E-11	1.34E-08	1.01E-08	30
BP	GO:0032680	regulation of tumor necrosis factor production	30/751	185/18614	7.30E-11	1.34E-08	1.01E-08	30
BP	GO:0002366	leukocyte activation involved in immune response	39/751	296/18614	7.52E-11	1.35E-08	1.01E-08	39
BP	GO:0009952	anterior/posterior pattern specification	32/751	209/18614	7.97E-11	1.39E-08	1.04E-08	32
BP	GO:0002263	cell activation involved in immune response	39/751	300/18614	1.13E-10	1.92E-08	1.44E-08	39
BP	GO:0002478	antigen processing and presentation of exogenous peptide antigen	14/751	40/18614	2.35E-10	3.89E-08	2.93E-08	14
BP	GO:0003002	regionalization	47/751	422/18614	3.03E-10	4.90E-08	3.68E-08	47

BP	GO:0048705	skeletal system morphogenesis	32/751	229/18614	8.68E-10	1.37E-07	1.03E-07	32
BP	GO:1903557	positive regulation of tumor necrosis factor superfamily cytokine production	21/751	109/18614	2.14E-09	3.29E-07	2.47E-07	21
BP	GO:0048002	antigen processing and presentation of peptide antigen	17/751	71/18614	2.23E-09	3.36E-07	2.53E-07	17
BP	GO:0002444	myeloid leukocyte mediated immunity	21/751	110/18614	2.54E-09	3.70E-07	2.78E-07	21
BP	GO:0050670	regulation of lymphocyte proliferation	32/751	239/18614	2.57E-09	3.70E-07	2.78E-07	32
BP	GO:0007389	pattern specification process	48/751	467/18614	2.88E-09	4.06E-07	3.06E-07	48
BP	GO:0070663	regulation of leukocyte proliferation	34/751	268/18614	3.38E-09	4.66E-07	3.50E-07	34
BP	GO:0032944	regulation of mononuclear cell proliferation	32/751	243/18614	3.89E-09	5.26E-07	3.96E-07	32
BP	GO:0019884	antigen processing and presentation of exogenous antigen	14/751	49/18614	4.88E-09	6.43E-07	4.83E-07	14
BP	GO:0019882	antigen processing and presentation	21/751	114/18614	4.99E-09	6.43E-07	4.83E-07	21
BP	GO:0002687	positive regulation of leukocyte migration	24/751	147/18614	5.05E-09	6.43E-07	4.83E-07	24
BP	GO:0042129	regulation of T cell proliferation	27/751	183/18614	5.49E-09	6.86E-07	5.16E-07	27
BP	GO:0032760	positive regulation of tumor necrosis factor production	20/751	105/18614	6.34E-09	7.77E-07	5.84E-07	20
BP	GO:0032609	type II interferon production	21/751	118/18614	9.49E-09	1.12E-06	8.44E-07	21
BP	GO:0032649	regulation of type II interferon production	21/751	118/18614	9.49E-09	1.12E-06	8.44E-07	21
BP	GO:0002275	myeloid cell activation involved in immune response	19/751	98/18614	1.12E-08	1.30E-06	9.75E-07	19
BP	GO:0002695	negative regulation of leukocyte activation	28/751	202/18614	1.18E-08	1.34E-06	1.01E-06	28
BP	GO:0030595	leukocyte chemotaxis	31/751	241/18614	1.20E-08	1.34E-06	1.01E-06	31
BP	GO:0032677	regulation of interleukin-8 production	19/751	103/18614	2.62E-08	2.89E-06	2.17E-06	19
BP	GO:0032637	interleukin-8 production	19/751	104/18614	3.08E-08	3.35E-06	2.52E-06	19
BP	GO:0050866	negative regulation of cell activation	29/751	225/18614	3.38E-08	3.61E-06	2.72E-06	29
BP	GO:0002697	regulation of immune effector process	40/751	384/18614	4.26E-08	4.48E-06	3.37E-06	40

BP	GO:0045055	regulated exocytosis	29/751	228/18614	4.55E-08	4.71E-06	3.54E-06	29
BP	GO:0071674	mononuclear cell migration	27/751	202/18614	4.67E-08	4.76E-06	3.58E-06	27
BP	GO:0002440	production of molecular mediator of immune response	36/751	328/18614	5.45E-08	5.47E-06	4.11E-06	36
BP	GO:0070665	positive regulation of leukocyte proliferation	24/751	166/18614	5.74E-08	5.68E-06	4.27E-06	24
BP	GO:0042116	macrophage activation	19/751	109/18614	6.78E-08	6.60E-06	4.97E-06	19
BP	GO:0050671	positive regulation of lymphocyte proliferation	22/751	145/18614	8.53E-08	8.18E-06	6.16E-06	22
BP	GO:0048562	embryonic organ morphogenesis	33/751	294/18614	1.15E-07	1.09E-05	8.20E-06	33
BP	GO:0043299	leukocyte degranulation	16/751	81/18614	1.22E-07	1.14E-05	8.54E-06	16
BP	GO:0032946	positive regulation of mononuclear cell proliferation	22/751	148/18614	1.24E-07	1.14E-05	8.56E-06	22
BP	GO:0002685	regulation of leukocyte migration	28/751	227/18614	1.49E-07	1.35E-05	1.02E-05	28
BP	GO:0060326	cell chemotaxis	34/751	312/18614	1.52E-07	1.35E-05	1.02E-05	34
BP	GO:0097529	myeloid leukocyte migration	29/751	241/18614	1.53E-07	1.35E-05	1.02E-05	29
BP	GO:0032623	interleukin-2 production	14/751	63/18614	1.60E-07	1.38E-05	1.03E-05	14
BP	GO:0032663	regulation of interleukin-2 production	14/751	63/18614	1.60E-07	1.38E-05	1.03E-05	14
BP	GO:0030198	extracellular matrix organization	34/751	314/18614	1.77E-07	1.50E-05	1.13E-05	34
BP	GO:0043062	extracellular structure organization	34/751	315/18614	1.91E-07	1.60E-05	1.20E-05	34
BP	GO:0045229	external encapsulating structure organization	34/751	317/18614	2.22E-07	1.83E-05	1.38E-05	34
BP	GO:0002381	immunoglobulin production involved in immunoglobulin-mediated immune response	15/751	75/18614	2.54E-07	2.07E-05	1.55E-05	15
BP	GO:1903131	mononuclear cell differentiation	44/751	474/18614	2.56E-07	2.07E-05	1.55E-05	44
BP	GO:0032612	interleukin-1 production	20/751	131/18614	2.96E-07	2.30E-05	1.73E-05	20
BP	GO:0032652	regulation of interleukin-1 production	20/751	131/18614	2.96E-07	2.30E-05	1.73E-05	20
BP	GO:0050868	negative regulation of T cell activation	20/751	131/18614	2.96E-07	2.30E-05	1.73E-05	20
BP	GO:0002764	immune response-regulating signaling pathway	42/751	450/18614	4.15E-07	3.19E-05	2.40E-05	42
BP	GO:0002699	positive regulation of immune effector process	30/751	267/18614	4.23E-07	3.22E-05	2.42E-05	30

BP	GO:0060078	regulation of postsynaptic membrane potential	21/751	148/18614	5.29E-07	3.98E-05	2.99E-05	21
BP	GO:0060079	excitatory postsynaptic potential	19/751	124/18614	5.50E-07	4.10E-05	3.08E-05	19
BP	GO:0051216	cartilage development	25/751	203/18614	7.08E-07	5.21E-05	3.92E-05	25
BP	GO:1902105	regulation of leukocyte differentiation	33/751	319/18614	7.61E-07	5.54E-05	4.17E-05	33
BP	GO:0002291	T cell activation via T cell receptor contact with antigen bound to MHC molecule on antigen presenting cell	6/751	10/18614	7.73E-07	5.56E-05	4.18E-05	6
BP	GO:0002237	response to molecule of bacterial origin	36/751	366/18614	8.15E-07	5.76E-05	4.33E-05	36
BP	GO:0032635	interleukin-6 production	23/751	178/18614	8.41E-07	5.76E-05	4.33E-05	23
BP	GO:0032675	regulation of interleukin-6 production	23/751	178/18614	8.41E-07	5.76E-05	4.33E-05	23
BP	GO:0006887	exocytosis	35/751	351/18614	8.42E-07	5.76E-05	4.33E-05	35
BP	GO:0002253	activation of immune response	44/751	495/18614	8.43E-07	5.76E-05	4.33E-05	44
BP	GO:0007188	adenylate cyclase-modulating G protein-coupled receptor signaling pathway	27/751	234/18614	9.45E-07	6.39E-05	4.80E-05	27
BP	GO:0042102	positive regulation of T cell proliferation	17/751	105/18614	9.79E-07	6.55E-05	4.93E-05	17
BP	GO:0042391	regulation of membrane potential	40/751	433/18614	1.02E-06	6.74E-05	5.07E-05	40
BP	GO:0050808	synapse organization	42/751	466/18614	1.05E-06	6.82E-05	5.13E-05	42
BP	GO:0071216	cellular response to biotic stimulus	29/751	264/18614	1.05E-06	6.82E-05	5.13E-05	29
BP	GO:0017157	regulation of exocytosis	24/751	194/18614	1.08E-06	6.92E-05	5.20E-05	24
BP	GO:0050766	positive regulation of phagocytosis	14/751	73/18614	1.09E-06	6.92E-05	5.20E-05	14
BP	GO:0032757	positive regulation of interleukin-8 production	13/751	63/18614	1.10E-06	6.92E-05	5.20E-05	13
BP	GO:0051250	negative regulation of lymphocyte activation	22/751	168/18614	1.15E-06	7.21E-05	5.42E-05	22
BP	GO:0071219	cellular response to molecule of bacterial origin	27/751	237/18614	1.21E-06	7.51E-05	5.65E-05	27
BP	GO:0048568	embryonic organ development	41/751	453/18614	1.26E-06	7.74E-05	5.82E-05	41
BP	GO:0002683	negative regulation of immune system process	43/751	487/18614	1.34E-06	8.15E-05	6.13E-05	43
BP	GO:0001906	cell killing	24/751	197/18614	1.43E-06	8.62E-05	6.48E-05	24
BP	GO:0099565	chemical synaptic transmission, postsynaptic	19/751	132/18614	1.46E-06	8.73E-05	6.57E-05	19

BP	GO:0030326	embryonic limb morphogenesis	18/751	120/18614	1.50E-06	8.78E-05	6.60E-05	18
BP	GO:0035113	embryonic appendage morphogenesis	18/751	120/18614	1.50E-06	8.78E-05	6.60E-05	18
BP	GO:0002768	immune response-regulating cell surface receptor signaling pathway	33/751	329/18614	1.52E-06	8.81E-05	6.63E-05	33
BP	GO:0015844	monoamine transport	15/751	86/18614	1.61E-06	9.25E-05	6.95E-05	15
BP	GO:0038063	collagen-activated tyrosine kinase receptor signaling pathway	6/751	11/18614	1.64E-06	9.38E-05	7.05E-05	6
BP	GO:0032613	interleukin-10 production	13/751	66/18614	1.92E-06	0.0001075	8.09E-05	13
BP	GO:0032653	regulation of interleukin-10 production	13/751	66/18614	1.92E-06	0.0001075	8.09E-05	13
BP	GO:0032729	positive regulation of type II interferon production	14/751	77/18614	2.13E-06	0.00011847	8.91E-05	14
BP	GO:0061448	connective tissue development	29/751	275/18614	2.41E-06	0.00013286	9.99E-05	29
BP	GO:0002703	regulation of leukocyte mediated immunity	27/751	246/18614	2.50E-06	0.00013665	0.00010277	27
BP	GO:1903038	negative regulation of leukocyte cell-cell adhesion	20/751	150/18614	2.65E-06	0.00014365	0.00010804	20
BP	GO:0050764	regulation of phagocytosis	16/751	101/18614	2.73E-06	0.00014707	0.00011061	16
BP	GO:0006959	humoral immune response	27/751	250/18614	3.40E-06	0.00018136	0.0001364	27
BP	GO:0006909	phagocytosis	26/751	236/18614	3.56E-06	0.00018867	0.0001419	26
BP	GO:0042060	wound healing	39/751	439/18614	3.59E-06	0.00018886	0.00014204	39
BP	GO:0072678	T cell migration	13/751	71/18614	4.52E-06	0.00023568	0.00017725	13
BP	GO:1902107	positive regulation of leukocyte differentiation	22/751	184/18614	5.30E-06	0.00027073	0.00020361	22
BP	GO:1903708	positive regulation of hemopoiesis	22/751	184/18614	5.30E-06	0.00027073	0.00020361	22
BP	GO:0071677	positive regulation of mononuclear cell migration	13/751	72/18614	5.31E-06	0.00027073	0.00020361	13
BP	GO:0071621	granulocyte chemotaxis	18/751	131/18614	5.40E-06	0.00027281	0.00020518	18
BP	GO:0002429	immune response-activating cell surface receptor signaling pathway	30/751	302/18614	5.48E-06	0.00027475	0.00020664	30
BP	GO:0060740	prostate gland epithelium morphogenesis	8/751	26/18614	5.53E-06	0.00027552	0.00020721	8
BP	GO:1904862	inhibitory synapse assembly	7/751	19/18614	5.57E-06	0.00027552	0.00020721	7

BP	GO:0009954	proximal/distal pattern formation	9/751	34/18614	5.71E-06	0.00027989	0.0002105	9
BP	GO:0002286	T cell activation involved in immune response	17/751	119/18614	5.75E-06	0.00027989	0.0002105	17
BP	GO:0023061	signal release	41/751	483/18614	6.25E-06	0.00030207	0.00022718	41
BP	GO:0035107	appendage morphogenesis	19/751	146/18614	6.71E-06	0.00031977	0.0002405	19
BP	GO:0035108	limb morphogenesis	19/751	146/18614	6.71E-06	0.00031977	0.0002405	19
BP	GO:0019722	calcium-mediated signaling	22/751	187/18614	6.90E-06	0.00032648	0.00024554	22
BP	GO:0002367	cytokine production involved in immune response	17/751	121/18614	7.23E-06	0.00033942	0.00025527	17
BP	GO:0010818	T cell chemotaxis	8/751	27/18614	7.59E-06	0.00035391	0.00026617	8
BP	GO:0002792	negative regulation of peptide secretion	10/751	44/18614	7.68E-06	0.00035589	0.00026766	10
BP	GO:0019932	second-messenger-mediated signaling	29/751	292/18614	7.86E-06	0.00036134	0.00027176	29
BP	GO:0071675	regulation of mononuclear cell migration	17/751	122/18614	8.09E-06	0.00036681	0.00027587	17
BP	GO:0072676	lymphocyte migration	17/751	122/18614	8.09E-06	0.00036681	0.00027587	17
BP	GO:0046631	alpha-beta T cell activation	21/751	175/18614	8.15E-06	0.0003672	0.00027616	21
BP	GO:0030098	lymphocyte differentiation	37/751	422/18614	8.58E-06	0.00038385	0.00028869	37
BP	GO:0002757	immune response-activating signaling pathway	37/751	423/18614	9.05E-06	0.00040213	0.00030243	37
BP	GO:0032602	chemokine production	15/751	99/18614	9.76E-06	0.00043083	0.00032402	15
BP	GO:0060512	prostate gland morphogenesis	8/751	28/18614	1.02E-05	0.00044953	0.00033809	8
BP	GO:0032611	interleukin-1 beta production	16/751	112/18614	1.08E-05	0.00046567	0.00035022	16
BP	GO:0032651	regulation of interleukin-1 beta production	16/751	112/18614	1.08E-05	0.00046567	0.00035022	16
BP	GO:0032496	response to lipopolysaccharide	32/751	345/18614	1.12E-05	0.00048141	0.00036206	32
BP	GO:0001655	urogenital system development	12/751	66/18614	1.13E-05	0.00048141	0.00036206	12
BP	GO:0034765	regulation of monoatomic ion transmembrane transport	40/751	478/18614	1.13E-05	0.00048141	0.00036206	40
BP	GO:0007204	positive regulation of cytosolic calcium ion concentration	21/751	179/18614	1.16E-05	0.00048898	0.00036776	21
BP	GO:0045580	regulation of T cell differentiation	21/751	180/18614	1.26E-05	0.00052963	0.00039833	21
BP	GO:0030217	T cell differentiation	29/751	300/18614	1.32E-05	0.0005495	0.00041327	29

BP	GO:0030900	forebrain development	35/751	397/18614	1.33E-05	0.00055048	0.00041401	35
BP	GO:0002526	acute inflammatory response	16/751	114/18614	1.35E-05	0.00055639	0.00041845	16
BP	GO:1903706	regulation of hemopoiesis	36/751	415/18614	1.43E-05	0.00058455	0.00043963	36
BP	GO:0050433	regulation of catecholamine secretion	11/751	57/18614	1.45E-05	0.00058881	0.00044283	11
BP	GO:0048736	appendage development	21/751	182/18614	1.50E-05	0.00060128	0.00045222	21
BP	GO:0060173	limb development	21/751	182/18614	1.50E-05	0.00060128	0.00045222	21
BP	GO:0048934	peripheral nervous system neuron differentiation	6/751	15/18614	1.55E-05	0.00061391	0.00046171	6
BP	GO:0048935	peripheral nervous system neuron development	6/751	15/18614	1.55E-05	0.00061391	0.00046171	6
BP	GO:0006813	potassium ion transport	25/751	241/18614	1.56E-05	0.00061643	0.00046361	25
BP	GO:0045619	regulation of lymphocyte differentiation	23/751	212/18614	1.65E-05	0.00064631	0.00048608	23
BP	GO:0030850	prostate gland development	10/751	48/18614	1.75E-05	0.00068108	0.00051223	10
BP	GO:0097530	granulocyte migration	19/751	156/18614	1.76E-05	0.00068108	0.00051223	19
BP	GO:0045621	positive regulation of lymphocyte differentiation	17/751	130/18614	1.90E-05	0.00072748	0.00054712	17
BP	GO:1903305	regulation of regulated secretory pathway	17/751	130/18614	1.90E-05	0.00072748	0.00054712	17
BP	GO:0006816	calcium ion transport	38/751	455/18614	1.96E-05	0.00074465	0.00056004	38
BP	GO:0050432	catecholamine secretion	11/751	59/18614	2.04E-05	0.000773	0.00058136	11
BP	GO:0043270	positive regulation of monoatomic ion transport	24/751	231/18614	2.24E-05	0.00084001	0.00063176	24
BP	GO:0044341	sodium-dependent phosphate transport	5/751	10/18614	2.24E-05	0.00084001	0.00063176	5
BP	GO:0048732	gland development	37/751	441/18614	2.27E-05	0.00084568	0.00063602	37
BP	GO:0002718	regulation of cytokine production involved in immune response	16/751	119/18614	2.34E-05	0.00086574	0.00065111	16
BP	GO:0038065	collagen-activated signaling pathway	6/751	16/18614	2.39E-05	0.00087255	0.00065623	6
BP	GO:0035136	forelimb morphogenesis	9/751	40/18614	2.40E-05	0.00087255	0.00065623	9
BP	GO:0071805	potassium ion transmembrane transport	23/751	217/18614	2.40E-05	0.00087255	0.00065623	23
BP	GO:0002886	regulation of myeloid leukocyte mediated immunity	11/751	60/18614	2.41E-05	0.00087255	0.00065623	11
BP	GO:0030593	neutrophil chemotaxis	15/751	107/18614	2.53E-05	0.00090906	0.00068369	15
BP	GO:0043410	positive regulation of MAPK cascade	40/751	495/18614	2.54E-05	0.00090906	0.00068369	40
BP	GO:0002700	regulation of production of molecular mediator of immune response	21/751	189/18614	2.66E-05	0.00094688	0.00071214	21

BP	GO:0042130	negative regulation of T cell proliferation	12/751	72/18614	2.83E-05	0.00099655	0.00074949	12
BP	GO:0046635	positive regulation of alpha-beta T cell activation	12/751	72/18614	2.83E-05	0.00099655	0.00074949	12
BP	GO:2000403	positive regulation of lymphocyte migration	9/751	41/18614	2.96E-05	0.00103762	0.00078038	9
BP	GO:0002690	positive regulation of leukocyte chemotaxis	14/751	96/18614	2.99E-05	0.00104139	0.00078321	14
BP	GO:0035115	embryonic forelimb morphogenesis	8/751	32/18614	3.01E-05	0.00104227	0.00078387	8
BP	GO:0002698	negative regulation of immune effector process	16/751	122/18614	3.20E-05	0.00110117	0.00082817	16
BP	GO:0098581	detection of external biotic stimulus	7/751	24/18614	3.21E-05	0.00110117	0.00082817	7
BP	GO:0010720	positive regulation of cell development	36/751	432/18614	3.37E-05	0.00115164	0.00086613	36
BP	GO:0050890	cognition	29/751	317/18614	3.69E-05	0.00123284	0.0009272	29
BP	GO:0051047	positive regulation of secretion	29/751	317/18614	3.69E-05	0.00123284	0.0009272	29
BP	GO:0001794	type IIa hypersensitivity	4/751	6/18614	3.70E-05	0.00123284	0.0009272	4
BP	GO:0002445	type II hypersensitivity	4/751	6/18614	3.70E-05	0.00123284	0.0009272	4
BP	GO:0002285	lymphocyte activation involved in immune response	22/751	208/18614	3.70E-05	0.00123284	0.0009272	22
BP	GO:0032642	regulation of chemokine production	14/751	98/18614	3.78E-05	0.0012429	0.00093477	14
BP	GO:1902476	chloride transmembrane transport	14/751	98/18614	3.78E-05	0.0012429	0.00093477	14
BP	GO:0015698	inorganic anion transport	20/751	179/18614	3.79E-05	0.0012429	0.00093477	20
BP	GO:0071222	cellular response to lipopolysaccharide	23/751	224/18614	3.98E-05	0.00127922	0.00096208	23
BP	GO:0032490	detection of molecule of bacterial origin	5/751	11/18614	3.98E-05	0.00127922	0.00096208	5
BP	GO:0051256	mitotic spindle midzone assembly	5/751	11/18614	3.98E-05	0.00127922	0.00096208	5
BP	GO:0060601	lateral sprouting from an epithelium	5/751	11/18614	3.98E-05	0.00127922	0.00096208	5
BP	GO:0032689	negative regulation of type II interferon production	9/751	43/18614	4.43E-05	0.00141187	0.00106184	9
BP	GO:0090278	negative regulation of peptide hormone secretion	9/751	43/18614	4.43E-05	0.00141187	0.00106184	9
BP	GO:0001909	leukocyte mediated cytotoxicity	17/751	139/18614	4.55E-05	0.00144322	0.00108542	17
BP	GO:0001818	negative regulation of cytokine production	32/751	371/18614	4.73E-05	0.00149101	0.00112136	32

BP	GO:0032743	positive regulation of interleukin-2 production	8/751	34/18614	4.83E-05	0.00150909	0.00113496	8
BP	GO:2000406	positive regulation of T cell migration	8/751	34/18614	4.83E-05	0.00150909	0.00113496	8
BP	GO:0006821	chloride transport	15/751	113/18614	4.86E-05	0.00151203	0.00113718	15
BP	GO:0002573	myeloid leukocyte differentiation	23/751	227/18614	4.90E-05	0.00151561	0.00113986	23
BP	GO:0098813	nuclear chromosome segregation	30/751	339/18614	5.06E-05	0.00155792	0.00117169	30
BP	GO:0010324	membrane invagination	11/751	65/18614	5.24E-05	0.00160742	0.00120891	11
BP	GO:0000070	mitotic sister chromatid segregation	21/751	199/18614	5.71E-05	0.00174389	0.00131156	21
BP	GO:1990266	neutrophil migration	16/751	128/18614	5.80E-05	0.00176114	0.00132452	16
BP	GO:0006584	catecholamine metabolic process	10/751	55/18614	6.07E-05	0.00182628	0.00137352	10
BP	GO:0009712	catechol-containing compound metabolic process	10/751	55/18614	6.07E-05	0.00182628	0.00137352	10
BP	GO:0009612	response to mechanical stimulus	22/751	215/18614	6.12E-05	0.00183415	0.00137944	22
BP	GO:0050921	positive regulation of chemotaxis	17/751	143/18614	6.54E-05	0.00194305	0.00146134	17
BP	GO:0070340	detection of bacterial lipopeptide	3/751	3/18614	6.54E-05	0.00194305	0.00146134	3
BP	GO:0000022	mitotic spindle elongation	5/751	12/18614	6.59E-05	0.00194668	0.00146407	5
BP	GO:0045582	positive regulation of T cell differentiation	15/751	116/18614	6.61E-05	0.00194668	0.00146407	15
BP	GO:0007162	negative regulation of cell adhesion	28/751	312/18614	6.99E-05	0.00204976	0.00154159	28
BP	GO:0030071	regulation of mitotic metaphase/anaphase transition	13/751	91/18614	7.10E-05	0.00206867	0.00155582	13
BP	GO:0007187	G protein-coupled receptor signaling pathway, coupled to cyclic nucleotide second messenger	10/751	56/18614	7.12E-05	0.00206867	0.00155582	10
BP	GO:0032930	positive regulation of superoxide anion generation	6/751	19/18614	7.31E-05	0.00208638	0.00156913	6
BP	GO:0055062	phosphate ion homeostasis	6/751	19/18614	7.31E-05	0.00208638	0.00156913	6
BP	GO:1903818	positive regulation of voltage-gated potassium channel activity	6/751	19/18614	7.31E-05	0.00208638	0.00156913	6
BP	GO:0009308	amine metabolic process	15/751	117/18614	7.31E-05	0.00208638	0.00156913	15
BP	GO:0051983	regulation of chromosome segregation	16/751	131/18614	7.68E-05	0.00218433	0.0016428	16

BP	GO:0002455	humoral immune response mediated by circulating immunoglobulin	9/751	46/18614	7.77E-05	0.00220034	0.00165484	9
BP	GO:0000819	sister chromatid segregation	24/751	250/18614	8.01E-05	0.00225724	0.00169764	24
BP	GO:0002704	negative regulation of leukocyte mediated immunity	11/751	68/18614	8.05E-05	0.00225773	0.00169801	11
BP	GO:0060638	mesenchymal-epithelial cell signaling	4/751	7/18614	8.35E-05	0.00231273	0.00173936	4
BP	GO:0110088	hippocampal neuron apoptotic process	4/751	7/18614	8.35E-05	0.00231273	0.00173936	4
BP	GO:0110089	regulation of hippocampal neuron apoptotic process	4/751	7/18614	8.35E-05	0.00231273	0.00173936	4
BP	GO:0042886	amide transport	30/751	349/18614	8.59E-05	0.00237115	0.00178331	30
BP	GO:0002819	regulation of adaptive immune response	21/751	205/18614	8.79E-05	0.00240299	0.00180725	21
BP	GO:0022408	negative regulation of cell-cell adhesion	21/751	205/18614	8.79E-05	0.00240299	0.00180725	21
BP	GO:0002822	regulation of adaptive immune response based on somatic recombination of immune receptors built from immunoglobulin superfamily domains	20/751	190/18614	8.82E-05	0.00240299	0.00180725	20
BP	GO:0007214	gamma-aminobutyric acid signaling pathway	7/751	28/18614	9.53E-05	0.00256698	0.00193058	7
BP	GO:0032703	negative regulation of interleukin-2 production	7/751	28/18614	9.53E-05	0.00256698	0.00193058	7
BP	GO:0036037	CD8-positive, alpha-beta T cell activation	7/751	28/18614	9.53E-05	0.00256698	0.00193058	7
BP	GO:0010965	regulation of mitotic sister chromatid separation	10/751	58/18614	9.70E-05	0.00259161	0.00194911	10
BP	GO:0099024	plasma membrane invagination	10/751	58/18614	9.70E-05	0.00259161	0.00194911	10
BP	GO:0070374	positive regulation of ERK1 and ERK2 cascade	22/751	222/18614	9.86E-05	0.00262144	0.00197154	22
BP	GO:1902099	regulation of metaphase/anaphase transition of cell cycle	13/751	94/18614	9.99E-05	0.00264671	0.00199055	13
BP	GO:0002702	positive regulation of production of molecular mediator of immune response	16/751	134/18614	0.00010091	0.00266276	0.00200262	16
BP	GO:0002524	hypersensitivity	5/751	13/18614	0.0001036	0.00272285	0.00204782	5

BP	GO:0032722	positive regulation of chemokine production	11/751	70/18614	0.00010554	0.00276284	0.00207789	11
BP	GO:0036230	granulocyte activation	9/751	48/18614	0.00011011	0.00285993	0.00215091	9
BP	GO:0043300	regulation of leukocyte degranulation	9/751	48/18614	0.00011011	0.00285993	0.00215091	9
BP	GO:0007091	metaphase/anaphase transition of mitotic cell cycle	13/751	95/18614	0.00011155	0.00288582	0.00217038	13
BP	GO:0098661	inorganic anion transmembrane transport	15/751	122/18614	0.00011829	0.0030485	0.00229273	15
BP	GO:0031644	regulation of nervous system process	17/751	150/18614	0.00011898	0.0030544	0.00229717	17
BP	GO:0003209	cardiac atrium morphogenesis	7/751	29/18614	0.00012134	0.00309086	0.00232459	7
BP	GO:1901018	positive regulation of potassium ion transmembrane transporter activity	7/751	29/18614	0.00012134	0.00309086	0.00232459	7
BP	GO:0009306	protein secretion	31/751	373/18614	0.0001223	0.00310348	0.00233408	31
BP	GO:0051960	regulation of nervous system development	36/751	461/18614	0.00012721	0.00321581	0.00241856	36
BP	GO:0035592	establishment of protein localization to extracellular region	31/751	374/18614	0.00012837	0.00323265	0.00243122	31
BP	GO:0002761	regulation of myeloid leukocyte differentiation	15/751	123/18614	0.0001298	0.00325196	0.00244575	15
BP	GO:0006911	phagocytosis, engulfment	9/751	49/18614	0.00013012	0.00325196	0.00244575	9
BP	GO:0002888	positive regulation of myeloid leukocyte mediated immunity	6/751	21/18614	0.00013635	0.00338212	0.00254364	6
BP	GO:0051315	attachment of mitotic spindle microtubules to kinetochore	6/751	21/18614	0.00013635	0.00338212	0.00254364	6
BP	GO:0051310	metaphase plate congression	11/751	72/18614	0.00013702	0.00338608	0.00254661	11
BP	GO:0002790	peptide secretion	23/751	243/18614	0.00013844	0.00340855	0.00256352	23
BP	GO:0018958	phenol-containing compound metabolic process	14/751	111/18614	0.00015037	0.00366218	0.00275426	14
BP	GO:0051306	mitotic sister chromatid separation	10/751	61/18614	0.0001504	0.00366218	0.00275426	10
BP	GO:1905517	macrophage migration	10/751	61/18614	0.0001504	0.00366218	0.00275426	10
BP	GO:0044784	metaphase/anaphase transition of cell cycle	13/751	98/18614	0.00015375	0.00368563	0.00277191	13
BP	GO:0051937	catecholamine transport	11/751	73/18614	0.00015554	0.00368563	0.00277191	11
BP	GO:0060415	muscle tissue morphogenesis	11/751	73/18614	0.00015554	0.00368563	0.00277191	11
BP	GO:0071260	cellular response to mechanical stimulus	11/751	73/18614	0.00015554	0.00368563	0.00277191	11

BP	GO:1905818	regulation of chromosome separation	11/751	73/18614	0.00015554	0.00368563	0.00277191	11
BP	GO:0098656	monoatomic anion transmembrane transport	15/751	125/18614	0.00015577	0.00368563	0.00277191	15
BP	GO:0051231	spindle elongation	5/751	14/18614	0.00015582	0.00368563	0.00277191	5
BP	GO:0051255	spindle midzone assembly	5/751	14/18614	0.00015582	0.00368563	0.00277191	5
BP	GO:0043313	regulation of neutrophil degranulation	4/751	8/18614	0.00016158	0.00378137	0.00284391	4
BP	GO:0051964	negative regulation of synapse assembly	4/751	8/18614	0.00016158	0.00378137	0.00284391	4
BP	GO:2000427	positive regulation of apoptotic cell clearance	4/751	8/18614	0.00016158	0.00378137	0.00284391	4
BP	GO:0032412	regulation of monoatomic ion transmembrane transporter activity	25/751	278/18614	0.00016277	0.0037959	0.00285484	25
BP	GO:0007229	integrin-mediated signaling pathway	14/751	112/18614	0.0001656	0.0038483	0.00289424	14
BP	GO:1990868	response to chemokine	13/751	99/18614	0.00017058	0.00393645	0.00296054	13
BP	GO:1990869	cellular response to chemokine	13/751	99/18614	0.00017058	0.00393645	0.00296054	13
BP	GO:0043030	regulation of macrophage activation	10/751	62/18614	0.00017294	0.00397691	0.00299097	10
BP	GO:0032732	positive regulation of interleukin-1 production	11/751	74/18614	0.00017614	0.00403666	0.00303591	11
BP	GO:0050804	modulation of chemical synaptic transmission	37/751	487/18614	0.00017882	0.00408386	0.00307141	37
BP	GO:0032928	regulation of superoxide anion generation	6/751	22/18614	0.00018111	0.00412206	0.00310013	6
BP	GO:0007200	phospholipase C-activating G protein-coupled receptor signaling pathway	14/751	113/18614	0.00018214	0.00413123	0.00310703	14
BP	GO:0099177	regulation of trans-synaptic signaling	37/751	488/18614	0.00018624	0.00420968	0.00316603	37
BP	GO:0071692	protein localization to extracellular region	31/751	382/18614	0.00018749	0.00422161	0.00317501	31
BP	GO:0033627	cell adhesion mediated by integrin	12/751	87/18614	0.00018804	0.00422161	0.00317501	12
BP	GO:0032755	positive regulation of interleukin-6 production	13/751	100/18614	0.00018897	0.00422813	0.00317991	13
BP	GO:1903531	negative regulation of secretion by cell	17/751	156/18614	0.00019226	0.00428733	0.00322444	17
BP	GO:0042417	dopamine metabolic process	8/751	41/18614	0.00019806	0.00438708	0.00329945	8
BP	GO:1901381	positive regulation of potassium ion transmembrane transport	8/751	41/18614	0.00019806	0.00438708	0.00329945	8
BP	GO:0009914	hormone transport	27/751	315/18614	0.00019999	0.0044026	0.00331113	27

BP	GO:0046634	regulation of alpha-beta T cell activation	14/751	114/18614	0.00020009	0.0044026	0.00331113	14
BP	GO:0006968	cellular defense response	9/751	52/18614	0.00020917	0.00457204	0.00343856	9
BP	GO:0046638	positive regulation of alpha-beta T cell differentiation	9/751	52/18614	0.00020917	0.00457204	0.00343856	9
BP	GO:0050672	negative regulation of lymphocyte proliferation	12/751	88/18614	0.00020988	0.00457245	0.00343886	12
BP	GO:0001763	morphogenesis of a branching structure	20/751	203/18614	0.00021743	0.00472152	0.00355098	20
BP	GO:2000027	regulation of animal organ morphogenesis	15/751	129/18614	0.00022148	0.00479362	0.0036052	15
BP	GO:0061138	morphogenesis of a branching epithelium	19/751	188/18614	0.00022343	0.0048099	0.00361745	19
BP	GO:0002468	dendritic cell antigen processing and presentation	5/751	15/18614	0.00022599	0.0048099	0.00361745	5
BP	GO:0007567	parturition	5/751	15/18614	0.00022599	0.0048099	0.00361745	5
BP	GO:0072498	embryonic skeletal joint development	5/751	15/18614	0.00022599	0.0048099	0.00361745	5
BP	GO:1902563	regulation of neutrophil activation	5/751	15/18614	0.00022599	0.0048099	0.00361745	5
BP	GO:0048247	lymphocyte chemotaxis	10/751	64/18614	0.00022659	0.0048099	0.00361745	10
BP	GO:0030316	osteoclast differentiation	13/751	102/18614	0.00023088	0.00488542	0.00367424	13
BP	GO:0032945	negative regulation of mononuclear cell proliferation	12/751	89/18614	0.00023384	0.0049323	0.0037095	12
BP	GO:0032733	positive regulation of interleukin-10 production	8/751	42/18614	0.00023611	0.00493292	0.00370997	8
BP	GO:0042119	neutrophil activation	8/751	42/18614	0.00023611	0.00493292	0.00370997	8
BP	GO:0045124	regulation of bone resorption	8/751	42/18614	0.00023611	0.00493292	0.00370997	8
BP	GO:0140014	mitotic nuclear division	25/751	286/18614	0.00025226	0.00520385	0.00391373	25
BP	GO:0021536	diencephalon development	11/751	77/18614	0.00025237	0.00520385	0.00391373	11
BP	GO:0042494	detection of bacterial lipoprotein	3/751	4/18614	0.00025381	0.00520385	0.00391373	3
BP	GO:0043315	positive regulation of neutrophil degranulation	3/751	4/18614	0.00025381	0.00520385	0.00391373	3
BP	GO:0051891	positive regulation of cardioblast differentiation	3/751	4/18614	0.00025381	0.00520385	0.00391373	3
BP	GO:0150062	complement-mediated synapse pruning	3/751	4/18614	0.00025381	0.00520385	0.00391373	3
BP	GO:0051783	regulation of nuclear division	16/751	145/18614	0.00025457	0.00520385	0.00391373	16
BP	GO:0034767	positive regulation of monoatomic ion transmembrane transport	18/751	175/18614	0.00026071	0.00531282	0.00399569	18

BP	GO:0007088	regulation of mitotic nuclear division	14/751	117/18614	0.00026336	0.00535045	0.00402399	14
BP	GO:0030072	peptide hormone secretion	22/751	238/18614	0.00026827	0.00543348	0.00408643	22
BP	GO:0050777	negative regulation of immune response	19/751	191/18614	0.00027384	0.00552935	0.00415854	19
BP	GO:0008608	attachment of spindle microtubules to kinetochore	8/751	43/18614	0.00027994	0.00553326	0.00416148	8
BP	GO:0033574	response to testosterone	8/751	43/18614	0.00027994	0.00553326	0.00416148	8
BP	GO:0042554	superoxide anion generation	8/751	43/18614	0.00027994	0.00553326	0.00416148	8
BP	GO:0033045	regulation of sister chromatid segregation	13/751	104/18614	0.00028051	0.00553326	0.00416148	13
BP	GO:1902850	microtubule cytoskeleton organization involved in mitosis	17/751	161/18614	0.00028072	0.00553326	0.00416148	17
BP	GO:0043303	mast cell degranulation	9/751	54/18614	0.00028137	0.00553326	0.00416148	9
BP	GO:0046645	positive regulation of gamma-delta T cell activation	4/751	9/18614	0.00028155	0.00553326	0.00416148	4
BP	GO:0060513	prostatic bud formation	4/751	9/18614	0.00028155	0.00553326	0.00416148	4
BP	GO:2001187	positive regulation of CD8-positive, alpha-beta T cell activation	4/751	9/18614	0.00028155	0.00553326	0.00416148	4
BP	GO:0046879	hormone secretion	26/751	305/18614	0.00028386	0.00556205	0.00418313	26
BP	GO:0006958	complement activation, classical pathway	7/751	33/18614	0.00028858	0.005593	0.00420641	7
BP	GO:0036336	dendritic cell migration	7/751	33/18614	0.00028858	0.005593	0.00420641	7
BP	GO:0048665	neuron fate specification	7/751	33/18614	0.00028858	0.005593	0.00420641	7
BP	GO:0070098	chemokine-mediated signaling pathway	12/751	91/18614	0.00028881	0.005593	0.00420641	12
BP	GO:0022898	regulation of transmembrane transporter activity	25/751	289/18614	0.00029567	0.00569245	0.0042812	25
BP	GO:1903532	positive regulation of secretion by cell	25/751	289/18614	0.00029567	0.00569245	0.0042812	25
BP	GO:0070371	ERK1 and ERK2 cascade	28/751	341/18614	0.00031195	0.00598862	0.00450394	28
BP	GO:0046632	alpha-beta T cell differentiation	14/751	119/18614	0.00031449	0.0060143	0.00452326	14
BP	GO:0051304	chromosome separation	11/751	79/18614	0.00031731	0.0060143	0.00452326	11
BP	GO:0006704	glucocorticoid biosynthetic process	5/751	16/18614	0.00031783	0.0060143	0.00452326	5
BP	GO:0010819	regulation of T cell chemotaxis	5/751	16/18614	0.00031783	0.0060143	0.00452326	5
BP	GO:0048172	regulation of short-term neuronal synaptic plasticity	5/751	16/18614	0.00031783	0.0060143	0.00452326	5

BP	GO:0048167	regulation of synaptic plasticity	20/751	209/18614	0.00031973	0.00602418	0.00453069	20
BP	GO:0050000	chromosome localization	12/751	92/18614	0.00032017	0.00602418	0.00453069	12
BP	GO:0031589	cell-substrate adhesion	29/751	359/18614	0.00032235	0.006048	0.00454861	29
BP	GO:0033674	positive regulation of kinase activity	33/751	430/18614	0.00032854	0.00614672	0.00462285	33
BP	GO:0070588	calcium ion transmembrane transport	29/751	360/18614	0.00033752	0.00629684	0.00473576	29
BP	GO:0015833	peptide transport	23/751	259/18614	0.00035096	0.00652602	0.00490811	23
BP	GO:0002720	positive regulation of cytokine production involved in immune response	11/751	80/18614	0.00035473	0.00652602	0.00490811	11
BP	GO:0007193	adenylate cyclase-inhibiting G protein-coupled receptor signaling pathway	11/751	80/18614	0.00035473	0.00652602	0.00490811	11
BP	GO:0045921	positive regulation of exocytosis	11/751	80/18614	0.00035473	0.00652602	0.00490811	11
BP	GO:0048644	muscle organ morphogenesis	11/751	80/18614	0.00035473	0.00652602	0.00490811	11
BP	GO:0000280	nuclear division	34/751	450/18614	0.00035708	0.00655106	0.00492695	34
BP	GO:0007611	learning or memory	24/751	276/18614	0.0003592	0.00657173	0.0049425	24
BP	GO:0051048	negative regulation of secretion	18/751	180/18614	0.00036843	0.00672203	0.00505553	18
BP	GO:0002279	mast cell activation involved in immune response	9/751	56/18614	0.00037307	0.00678799	0.00510514	9
BP	GO:2000401	regulation of lymphocyte migration	10/751	68/18614	0.00037627	0.00682749	0.00513485	10
BP	GO:0030890	positive regulation of B cell proliferation	8/751	45/18614	0.00038758	0.00697662	0.00524701	8
BP	GO:1903307	positive regulation of regulated secretory pathway	8/751	45/18614	0.00038758	0.00697662	0.00524701	8
BP	GO:0008211	glucocorticoid metabolic process	6/751	25/18614	0.00038765	0.00697662	0.00524701	6
BP	GO:0007059	chromosome segregation	33/751	435/18614	0.00040362	0.00724435	0.00544836	33
BP	GO:0030073	insulin secretion	19/751	197/18614	0.00040513	0.0072518	0.00545397	19
BP	GO:0002446	neutrophil mediated immunity	7/751	35/18614	0.00042349	0.00755601	0.00568276	7
BP	GO:0045576	mast cell activation	10/751	69/18614	0.00042441	0.00755601	0.00568276	10
BP	GO:0002448	mast cell mediated immunity	9/751	57/18614	0.0004274	0.00756868	0.00569228	9
BP	GO:0048168	regulation of neuronal synaptic plasticity	9/751	57/18614	0.0004274	0.00756868	0.00569228	9
BP	GO:0030100	regulation of endocytosis	20/751	214/18614	0.00043485	0.00766899	0.00576773	20

BP	GO:1901970	positive regulation of mitotic sister chromatid separation	5/751	17/18614	0.00043538	0.00766899	0.00576773	5
BP	GO:0022612	gland morphogenesis	14/751	123/18614	0.00044264	0.00771469	0.0058021	14
BP	GO:0045765	regulation of angiogenesis	28/751	349/18614	0.00045269	0.00771469	0.0058021	28
BP	GO:0007094	mitotic spindle assembly checkpoint signaling	8/751	46/18614	0.00045281	0.00771469	0.0058021	8
BP	GO:0071173	spindle assembly checkpoint signaling	8/751	46/18614	0.00045281	0.00771469	0.0058021	8
BP	GO:0071174	mitotic spindle checkpoint signaling	8/751	46/18614	0.00045281	0.00771469	0.0058021	8
BP	GO:0001820	serotonin secretion	4/751	10/18614	0.00045428	0.00771469	0.0058021	4
BP	GO:0021520	spinal cord motor neuron cell fate specification	4/751	10/18614	0.00045428	0.00771469	0.0058021	4
BP	GO:0070339	response to bacterial lipopeptide	4/751	10/18614	0.00045428	0.00771469	0.0058021	4
BP	GO:0071220	cellular response to bacterial lipoprotein	4/751	10/18614	0.00045428	0.00771469	0.0058021	4
BP	GO:0071221	cellular response to bacterial lipopeptide	4/751	10/18614	0.00045428	0.00771469	0.0058021	4
BP	GO:0098953	receptor diffusion trapping	4/751	10/18614	0.00045428	0.00771469	0.0058021	4
BP	GO:0098970	postsynaptic neurotransmitter receptor diffusion trapping	4/751	10/18614	0.00045428	0.00771469	0.0058021	4
BP	GO:0099628	neurotransmitter receptor diffusion trapping	4/751	10/18614	0.00045428	0.00771469	0.0058021	4
BP	GO:2000425	regulation of apoptotic cell clearance	4/751	10/18614	0.00045428	0.00771469	0.0058021	4
BP	GO:0070372	regulation of ERK1 and ERK2 cascade	26/751	315/18614	0.00046591	0.00789192	0.00593539	26
BP	GO:0070664	negative regulation of leukocyte proliferation	12/751	96/18614	0.00047607	0.00804339	0.00604931	12
BP	GO:0043627	response to estrogen	10/751	70/18614	0.00047755	0.00804786	0.00605267	10
BP	GO:0002706	regulation of lymphocyte mediated immunity	18/751	184/18614	0.00048055	0.00806279	0.0060639	18
BP	GO:0032411	positive regulation of transporter activity	14/751	124/18614	0.00048087	0.00806279	0.0060639	14
BP	GO:1903306	negative regulation of regulated secretory pathway	6/751	26/18614	0.00048689	0.00812265	0.00610891	6
BP	GO:1905523	positive regulation of macrophage migration	6/751	26/18614	0.00048689	0.00812265	0.00610891	6
BP	GO:0050871	positive regulation of B cell activation	11/751	83/18614	0.00048991	0.00815249	0.00613136	11
BP	GO:0050851	antigen receptor-mediated signaling pathway	19/751	201/18614	0.00052039	0.00863788	0.00649641	19

BP	GO:0006576	biogenic amine metabolic process	12/751	97/18614	0.00052374	0.0086719	0.006522	12
BP	GO:0031577	spindle checkpoint signaling	8/751	47/18614	0.00052666	0.00869849	0.006542	8
BP	GO:0003208	cardiac ventricle morphogenesis	10/751	71/18614	0.0005361	0.00883227	0.00664261	10
BP	GO:0007189	adenylate cyclase-activating G protein-coupled receptor signaling pathway	15/751	140/18614	0.00053827	0.008846	0.00665294	15
BP	GO:0051303	establishment of chromosome localization	11/751	84/18614	0.00054358	0.00891123	0.00670199	11
BP	GO:0030324	lung development	18/751	186/18614	0.0005469	0.00894358	0.00672633	18
BP	GO:0006836	neurotransmitter transport	19/751	202/18614	0.00055328	0.0090256	0.00678801	19
BP	GO:0008347	glial cell migration	9/751	59/18614	0.00055565	0.00904198	0.00680033	9
BP	GO:0002688	regulation of leukocyte chemotaxis	14/751	126/18614	0.00056581	0.0091623	0.00689082	14
BP	GO:0003206	cardiac chamber morphogenesis	14/751	126/18614	0.00056581	0.0091623	0.00689082	14
BP	GO:0051952	regulation of amine transport	12/751	98/18614	0.00057537	0.00929434	0.00699013	12
BP	GO:0007584	response to nutrient	16/751	156/18614	0.00057981	0.00934322	0.00702689	16
BP	GO:0071772	response to BMP	18/751	187/18614	0.00058294	0.00934828	0.00703069	18
BP	GO:0071773	cellular response to BMP stimulus	18/751	187/18614	0.00058294	0.00934828	0.00703069	18
BP	GO:0072001	renal system development	26/751	320/18614	0.00059057	0.00944772	0.00710548	26
BP	GO:1901342	regulation of vasculature development	28/751	355/18614	0.00059233	0.00945306	0.00710949	28
BP	GO:1904888	cranial skeletal system development	10/751	72/18614	0.00060047	0.00945701	0.00711246	10
BP	GO:0003230	cardiac atrium development	7/751	37/18614	0.00060453	0.00945701	0.00711246	7
BP	GO:0045920	negative regulation of exocytosis	7/751	37/18614	0.00060453	0.00945701	0.00711246	7
BP	GO:0001837	epithelial to mesenchymal transition	17/751	172/18614	0.00060678	0.00945701	0.00711246	17
BP	GO:0048015	phosphatidylinositol-mediated signaling	17/751	172/18614	0.00060678	0.00945701	0.00711246	17
BP	GO:0007520	myoblast fusion	8/751	48/18614	0.00060997	0.00945701	0.00711246	8
BP	GO:0033046	negative regulation of sister chromatid segregation	8/751	48/18614	0.00060997	0.00945701	0.00711246	8
BP	GO:0033048	negative regulation of mitotic sister chromatid segregation	8/751	48/18614	0.00060997	0.00945701	0.00711246	8
BP	GO:0043268	positive regulation of potassium ion transport	8/751	48/18614	0.00060997	0.00945701	0.00711246	8

BP	GO:0045841	negative regulation of mitotic metaphase/anaphase transition	8/751	48/18614	0.00060997	0.00945701	0.00711246	8
BP	GO:2000404	regulation of T cell migration	8/751	48/18614	0.00060997	0.00945701	0.00711246	8
BP	GO:2000816	negative regulation of mitotic sister chromatid separation	8/751	48/18614	0.00060997	0.00945701	0.00711246	8
BP	GO:0001788	antibody-dependent cellular cytotoxicity	3/751	5/18614	0.00061543	0.00945701	0.00711246	3
BP	GO:0030885	regulation of myeloid dendritic cell activation	3/751	5/18614	0.00061543	0.00945701	0.00711246	3
BP	GO:0043378	positive regulation of CD8-positive, alpha-beta T cell differentiation	3/751	5/18614	0.00061543	0.00945701	0.00711246	3
BP	GO:0048818	positive regulation of hair follicle maturation	3/751	5/18614	0.00061543	0.00945701	0.00711246	3
BP	GO:0032102	negative regulation of response to external stimulus	33/751	446/18614	0.00062453	0.00957467	0.00720096	33
BP	GO:0055008	cardiac muscle tissue morphogenesis	9/751	60/18614	0.0006307	0.00964549	0.00725422	9
BP	GO:0032414	positive regulation of ion transmembrane transporter activity	13/751	113/18614	0.00063206	0.00964549	0.00725422	13
BP	GO:0031214	biomineral tissue development	17/751	173/18614	0.00064827	0.00987013	0.00742316	17
BP	GO:0001503	ossification	32/751	429/18614	0.00065473	0.00993818	0.00747435	32
BP	GO:0043583	ear development	20/751	221/18614	0.00065574	0.00993818	0.00747435	20
BP	GO:0034103	regulation of tissue remodeling	11/751	86/18614	0.00066571	0.01006618	0.00757061	11
BP	GO:0046637	regulation of alpha-beta T cell differentiation	10/751	73/18614	0.00067109	0.01012441	0.00761441	10
BP	GO:0032493	response to bacterial lipoprotein	4/751	11/18614	0.00069114	0.01035616	0.0077887	4
BP	GO:0046643	regulation of gamma-delta T cell activation	4/751	11/18614	0.00069114	0.01035616	0.0077887	4
BP	GO:0060272	embryonic skeletal joint morphogenesis	4/751	11/18614	0.00069114	0.01035616	0.0077887	4
BP	GO:0030323	respiratory tube development	18/751	190/18614	0.0007036	0.01049544	0.00789345	18
BP	GO:0046850	regulation of bone remodeling	8/751	49/18614	0.0007036	0.01049544	0.00789345	8
BP	GO:0032409	regulation of transporter activity	26/751	324/18614	0.00071042	0.0105733	0.00795201	26
BP	GO:0009595	detection of biotic stimulus	7/751	38/18614	0.00071557	0.01060223	0.00797377	7

BP	GO:0046676	negative regulation of insulin secretion	7/751	38/18614	0.00071557	0.01060223	0.00797377	7
BP	GO:1905820	positive regulation of chromosome separation	6/751	28/18614	0.0007438	0.01099588	0.00826983	6
BP	GO:0045123	cellular extravasation	10/751	74/18614	0.00074842	0.01103966	0.00830275	10
BP	GO:0002283	neutrophil activation involved in immune response	5/751	19/18614	0.00076504	0.01118508	0.00841212	5
BP	GO:0030903	notochord development	5/751	19/18614	0.00076504	0.01118508	0.00841212	5
BP	GO:0098543	detection of other organism	5/751	19/18614	0.00076504	0.01118508	0.00841212	5
BP	GO:1901881	positive regulation of protein depolymerization	5/751	19/18614	0.00076504	0.01118508	0.00841212	5
BP	GO:0050864	regulation of B cell activation	14/751	130/18614	0.00077435	0.01129637	0.00849582	14
BP	GO:0048017	inositol lipid-mediated signaling	17/751	176/18614	0.00078766	0.0114652	0.00862279	17
BP	GO:1901654	response to ketone	19/751	208/18614	0.00079095	0.01148787	0.00863985	19
BP	GO:0000768	syncytium formation by plasma membrane fusion	9/751	62/18614	0.00080568	0.01160946	0.00873129	9
BP	GO:0140253	cell-cell fusion	9/751	62/18614	0.00080568	0.01160946	0.00873129	9
BP	GO:0051985	negative regulation of chromosome segregation	8/751	50/18614	0.00080847	0.01160946	0.00873129	8
BP	GO:1902100	negative regulation of metaphase/anaphase transition of cell cycle	8/751	50/18614	0.00080847	0.01160946	0.00873129	8
BP	GO:1905819	negative regulation of chromosome separation	8/751	50/18614	0.00080847	0.01160946	0.00873129	8
BP	GO:0021675	nerve development	11/751	88/18614	0.00080984	0.01160946	0.00873129	11
BP	GO:1901214	regulation of neuron death	26/751	327/18614	0.00081374	0.01164021	0.00875442	26
BP	GO:0001822	kidney development	25/751	310/18614	0.00083099	0.01186134	0.00892072	25
BP	GO:0042113	B cell activation	23/751	276/18614	0.00084986	0.01210459	0.00910367	23
BP	GO:0006820	monoatomic anion transport	15/751	147/18614	0.00089709	0.0127499	0.00958899	15
BP	GO:0019233	sensory perception of pain	12/751	103/18614	0.00090208	0.01279332	0.00962165	12
BP	GO:0001773	myeloid dendritic cell activation	6/751	29/18614	0.00090618	0.01280702	0.00963196	6
BP	GO:0032731	positive regulation of interleukin-1 beta production	9/751	63/18614	0.00090691	0.01280702	0.00963196	9
BP	GO:0060688	regulation of morphogenesis of a branching structure	8/751	51/18614	0.00092552	0.013042	0.00980868	8
BP	GO:0045860	positive regulation of protein kinase activity	28/751	366/18614	0.00094861	0.01333894	0.010032	28

BP	GO:0002705	positive regulation of leukocyte mediated immunity	15/751	148/18614	0.00096199	0.01349845	0.01015197	15
BP	GO:0015850	organic hydroxy compound transport	24/751	296/18614	0.00097142	0.0135946	0.01022428	24
BP	GO:0030099	myeloid cell differentiation	31/751	421/18614	0.00097457	0.0135946	0.01022428	31
BP	GO:0042092	type 2 immune response	7/751	40/18614	0.00098574	0.0135946	0.01022428	7
BP	GO:0071392	cellular response to estradiol stimulus	7/751	40/18614	0.00098574	0.0135946	0.01022428	7
BP	GO:0002544	chronic inflammatory response	5/751	20/18614	0.00098644	0.0135946	0.01022428	5
BP	GO:0002577	regulation of antigen processing and presentation	5/751	20/18614	0.00098644	0.0135946	0.01022428	5
BP	GO:2001185	regulation of CD8-positive, alpha-beta T cell activation	5/751	20/18614	0.00098644	0.0135946	0.01022428	5
BP	GO:0090068	positive regulation of cell cycle process	22/751	262/18614	0.00098786	0.0135946	0.01022428	22
BP	GO:0002377	immunoglobulin production	19/751	212/18614	0.00099418	0.0135946	0.01022428	19
BP	GO:0042742	defense response to bacterium	25/751	314/18614	0.00099722	0.0135946	0.01022428	25
BP	GO:0002887	negative regulation of myeloid leukocyte mediated immunity	4/751	12/18614	0.00100374	0.0135946	0.01022428	4
BP	GO:0034135	regulation of toll-like receptor 2 signaling pathway	4/751	12/18614	0.00100374	0.0135946	0.01022428	4
BP	GO:0043312	neutrophil degranulation	4/751	12/18614	0.00100374	0.0135946	0.01022428	4
BP	GO:0051798	positive regulation of hair follicle development	4/751	12/18614	0.00100374	0.0135946	0.01022428	4
BP	GO:0090232	positive regulation of spindle checkpoint	4/751	12/18614	0.00100374	0.0135946	0.01022428	4
BP	GO:0090267	positive regulation of mitotic cell cycle spindle assembly checkpoint	4/751	12/18614	0.00100374	0.0135946	0.01022428	4
BP	GO:1905809	negative regulation of synapse organization	4/751	12/18614	0.00100374	0.0135946	0.01022428	4
BP	GO:0050853	B cell receptor signaling pathway	10/751	77/18614	0.00102558	0.01386202	0.0104254	10
BP	GO:0010959	regulation of metal ion transport	31/751	423/18614	0.00105195	0.01418949	0.01067169	31
BP	GO:0007416	synapse assembly	18/751	197/18614	0.00107113	0.01441888	0.01084421	18
BP	GO:0035725	sodium ion transmembrane transport	17/751	181/18614	0.00107687	0.01443747	0.01085819	17
BP	GO:0048771	tissue remodeling	17/751	181/18614	0.00107687	0.01443747	0.01085819	17

BP	GO:0043032	positive regulation of macrophage activation	6/751	30/18614	0.00109452	0.01455622	0.0109475	6
BP	GO:0060142	regulation of syncytium formation by plasma membrane fusion	6/751	30/18614	0.00109452	0.01455622	0.0109475	6
BP	GO:0099505	regulation of presynaptic membrane potential	6/751	30/18614	0.00109452	0.01455622	0.0109475	6
BP	GO:0099590	neurotransmitter receptor internalization	6/751	30/18614	0.00109452	0.01455622	0.0109475	6
BP	GO:0045637	regulation of myeloid cell differentiation	19/751	214/18614	0.00111154	0.01475292	0.01109544	19
BP	GO:0003205	cardiac chamber development	16/751	166/18614	0.00113549	0.01495593	0.01124812	16
BP	GO:0031960	response to corticosteroid	16/751	166/18614	0.00113549	0.01495593	0.01124812	16
BP	GO:0017156	calcium-ion regulated exocytosis	9/751	65/18614	0.00114038	0.01495593	0.01124812	9
BP	GO:0030888	regulation of B cell proliferation	9/751	65/18614	0.00114038	0.01495593	0.01124812	9
BP	GO:0046888	negative regulation of hormone secretion	9/751	65/18614	0.00114038	0.01495593	0.01124812	9
BP	GO:1905330	regulation of morphogenesis of an epithelium	9/751	65/18614	0.00114038	0.01495593	0.01124812	9
BP	GO:0003278	apoptotic process involved in heart morphogenesis	3/751	6/18614	0.0011939	0.015474	0.01163775	3
BP	GO:0033239	negative regulation of amine metabolic process	3/751	6/18614	0.0011939	0.015474	0.01163775	3
BP	GO:0051890	regulation of cardioblast differentiation	3/751	6/18614	0.0011939	0.015474	0.01163775	3
BP	GO:0060685	regulation of prostatic bud formation	3/751	6/18614	0.0011939	0.015474	0.01163775	3
BP	GO:1902565	positive regulation of neutrophil activation	3/751	6/18614	0.0011939	0.015474	0.01163775	3
BP	GO:2001188	regulation of T cell activation via T cell receptor contact with antigen bound to MHC molecule on antigen presenting cell	3/751	6/18614	0.0011939	0.015474	0.01163775	3
BP	GO:0033047	regulation of mitotic sister chromatid segregation	8/751	53/18614	0.00120025	0.01552591	0.01167679	8
BP	GO:0030282	bone mineralization	13/751	121/18614	0.00120418	0.01554636	0.01169217	13
BP	GO:0048608	reproductive structure development	24/751	301/18614	0.00122229	0.01574948	0.01184493	24
BP	GO:0006837	serotonin transport	5/751	21/18614	0.0012521	0.01603995	0.01206339	5
BP	GO:0090026	positive regulation of monocyte chemotaxis	5/751	21/18614	0.0012521	0.01603995	0.01206339	5

BP	GO:1901739	regulation of myoblast fusion	5/751	21/18614	0.0012521	0.01603995	0.01206339	5
BP	GO:0015837	amine transport	12/751	107/18614	0.00126354	0.01615531	0.01215015	12
BP	GO:0006949	syncytium formation	9/751	66/18614	0.00127409	0.0162588	0.01222799	9
BP	GO:0046661	male sex differentiation	16/751	168/18614	0.00128872	0.01641377	0.01234454	16
BP	GO:0071385	cellular response to glucocorticoid stimulus	8/751	54/18614	0.00136006	0.01728916	0.0130029	8
BP	GO:0010721	negative regulation of cell development	23/751	286/18614	0.00136752	0.01730349	0.01301368	23
BP	GO:0045766	positive regulation of angiogenesis	17/751	185/18614	0.00136902	0.01730349	0.01301368	17
BP	GO:1904018	positive regulation of vasculature development	17/751	185/18614	0.00136902	0.01730349	0.01301368	17
BP	GO:0030500	regulation of bone mineralization	10/751	80/18614	0.00138156	0.01737824	0.0130699	10
BP	GO:1903556	negative regulation of tumor necrosis factor superfamily cytokine production	10/751	80/18614	0.00138156	0.01737824	0.0130699	10
BP	GO:0034764	positive regulation of transmembrane transport	20/751	235/18614	0.0013991	0.01737824	0.0130699	20
BP	GO:0002824	positive regulation of adaptive immune response based on somatic recombination of immune receptors built from immunoglobulin superfamily domains	13/751	123/18614	0.00140049	0.01737824	0.0130699	13
BP	GO:0002430	complement receptor mediated signaling pathway	4/751	13/18614	0.0014038	0.01737824	0.0130699	4
BP	GO:0032621	interleukin-18 production	4/751	13/18614	0.0014038	0.01737824	0.0130699	4
BP	GO:0032661	regulation of interleukin-18 production	4/751	13/18614	0.0014038	0.01737824	0.0130699	4
BP	GO:0033690	positive regulation of osteoblast proliferation	4/751	13/18614	0.0014038	0.01737824	0.0130699	4
BP	GO:0036006	cellular response to macrophage colony-stimulating factor stimulus	4/751	13/18614	0.0014038	0.01737824	0.0130699	4
BP	GO:0060100	positive regulation of phagocytosis, engulfment	4/751	13/18614	0.0014038	0.01737824	0.0130699	4
BP	GO:1905155	positive regulation of membrane invagination	4/751	13/18614	0.0014038	0.01737824	0.0130699	4
BP	GO:0043523	regulation of neuron apoptotic process	19/751	219/18614	0.00145785	0.01801365	0.01354778	19
BP	GO:0061458	reproductive system development	24/751	305/18614	0.00146141	0.01802409	0.01355563	24
BP	GO:0001708	cell fate specification	12/751	109/18614	0.00148493	0.01828011	0.01374818	12

BP	GO:0050727	regulation of inflammatory response	30/751	414/18614	0.00149786	0.01840504	0.01384214	30
BP	GO:0003229	ventricular cardiac muscle tissue development	8/751	55/18614	0.00153631	0.01872544	0.0140831	8
BP	GO:0043370	regulation of CD4-positive, alpha-beta T cell differentiation	8/751	55/18614	0.00153631	0.01872544	0.0140831	8
BP	GO:0099003	vesicle-mediated transport in synapse	19/751	220/18614	0.00153712	0.01872544	0.0140831	19
BP	GO:0003228	atrial cardiac muscle tissue development	5/751	22/18614	0.0015671	0.01872544	0.0140831	5
BP	GO:0009713	catechol-containing compound biosynthetic process	5/751	22/18614	0.0015671	0.01872544	0.0140831	5
BP	GO:0033630	positive regulation of cell adhesion mediated by integrin	5/751	22/18614	0.0015671	0.01872544	0.0140831	5
BP	GO:0042423	catecholamine biosynthetic process	5/751	22/18614	0.0015671	0.01872544	0.0140831	5
BP	GO:0051383	kinetochore organization	5/751	22/18614	0.0015671	0.01872544	0.0140831	5
BP	GO:0045670	regulation of osteoclast differentiation	9/751	68/18614	0.0015794	0.01872544	0.0140831	9
BP	GO:0048645	animal organ formation	9/751	68/18614	0.0015794	0.01872544	0.0140831	9
BP	GO:0031341	regulation of cell killing	12/751	110/18614	0.00160707	0.01872544	0.0140831	12
BP	GO:0002491	antigen processing and presentation of endogenous peptide antigen via MHC class II	2/751	2/18614	0.00162572	0.01872544	0.0140831	2
BP	GO:0032632	interleukin-3 production	2/751	2/18614	0.00162572	0.01872544	0.0140831	2
BP	GO:0032672	regulation of interleukin-3 production	2/751	2/18614	0.00162572	0.01872544	0.0140831	2
BP	GO:0032812	positive regulation of epinephrine secretion	2/751	2/18614	0.00162572	0.01872544	0.0140831	2
BP	GO:0035700	astrocyte chemotaxis	2/751	2/18614	0.00162572	0.01872544	0.0140831	2
BP	GO:0038124	toll-like receptor TLR6:TLR2 signaling pathway	2/751	2/18614	0.00162572	0.01872544	0.0140831	2
BP	GO:0042495	detection of triacyl bacterial lipopeptide	2/751	2/18614	0.00162572	0.01872544	0.0140831	2
BP	GO:0042496	detection of diacyl bacterial lipopeptide	2/751	2/18614	0.00162572	0.01872544	0.0140831	2
BP	GO:0043449	cellular alkene metabolic process	2/751	2/18614	0.00162572	0.01872544	0.0140831	2
BP	GO:0045914	negative regulation of catecholamine metabolic process	2/751	2/18614	0.00162572	0.01872544	0.0140831	2

BP	GO:0045963	negative regulation of dopamine metabolic process	2/751	2/18614	0.00162572	0.01872544	0.0140831	2
BP	GO:0060302	negative regulation of cytokine activity	2/751	2/18614	0.00162572	0.01872544	0.0140831	2
BP	GO:0060448	dichotomous subdivision of terminal units involved in lung branching	2/751	2/18614	0.00162572	0.01872544	0.0140831	2
BP	GO:0060599	lateral sprouting involved in mammary gland duct morphogenesis	2/751	2/18614	0.00162572	0.01872544	0.0140831	2
BP	GO:0060929	atrioventricular node cell fate commitment	2/751	2/18614	0.00162572	0.01872544	0.0140831	2
BP	GO:0062044	negative regulation of cardiac epithelial to mesenchymal transition	2/751	2/18614	0.00162572	0.01872544	0.0140831	2
BP	GO:0099607	lateral attachment of mitotic spindle microtubules to kinetochore	2/751	2/18614	0.00162572	0.01872544	0.0140831	2
BP	GO:1900673	olefin metabolic process	2/751	2/18614	0.00162572	0.01872544	0.0140831	2
BP	GO:1904093	negative regulation of autophagic cell death	2/751	2/18614	0.00162572	0.01872544	0.0140831	2
BP	GO:1905006	negative regulation of epithelial to mesenchymal transition involved in endocardial cushion formation	2/751	2/18614	0.00162572	0.01872544	0.0140831	2
BP	GO:1905443	regulation of clathrin coat assembly	2/751	2/18614	0.00162572	0.01872544	0.0140831	2
BP	GO:1905445	positive regulation of clathrin coat assembly	2/751	2/18614	0.00162572	0.01872544	0.0140831	2
BP	GO:2000458	regulation of astrocyte chemotaxis	2/751	2/18614	0.00162572	0.01872544	0.0140831	2
BP	GO:2000471	regulation of hematopoietic stem cell migration	2/751	2/18614	0.00162572	0.01872544	0.0140831	2
BP	GO:2000473	positive regulation of hematopoietic stem cell migration	2/751	2/18614	0.00162572	0.01872544	0.0140831	2
BP	GO:0006898	receptor-mediated endocytosis	21/751	256/18614	0.00170122	0.01956109	0.01471158	21
BP	GO:0050767	regulation of neurogenesis	28/751	381/18614	0.00172691	0.01982202	0.01490783	28
BP	GO:0045839	negative regulation of mitotic nuclear division	8/751	56/18614	0.00173019	0.0198253	0.01491029	8
BP	GO:0003231	cardiac ventricle development	13/751	126/18614	0.00174426	0.01993961	0.01499626	13
BP	GO:0030902	hindbrain development	15/751	157/18614	0.00174618	0.01993961	0.01499626	15

BP	GO:0051384	response to glucocorticoid	14/751	142/18614	0.00182506	0.02080441	0.01564667	14
BP	GO:0002828	regulation of type 2 immune response	6/751	33/18614	0.00184226	0.02096443	0.01576702	6
BP	GO:0001704	formation of primary germ layer	13/751	127/18614	0.00187328	0.02127765	0.01600258	13
BP	GO:1905954	positive regulation of lipid localization	12/751	112/18614	0.00187621	0.02127765	0.01600258	12
BP	GO:0003207	cardiac chamber formation	4/751	14/18614	0.00190299	0.0215077	0.0161756	4
BP	GO:0043301	negative regulation of leukocyte degranulation	4/751	14/18614	0.00190299	0.0215077	0.0161756	4
BP	GO:0060065	uterus development	5/751	23/18614	0.00193664	0.02184674	0.01643058	5
BP	GO:0002931	response to ischemia	8/751	57/18614	0.00194289	0.02184674	0.01643058	8
BP	GO:0007080	mitotic metaphase plate congression	8/751	57/18614	0.00194289	0.02184674	0.01643058	8
BP	GO:0048285	organelle fission	34/751	497/18614	0.00197676	0.02218993	0.01668869	34
BP	GO:0000281	mitotic cytokinesis	10/751	84/18614	0.00200618	0.02222362	0.01671403	10
BP	GO:0031349	positive regulation of defense response	31/751	441/18614	0.00202587	0.02222362	0.01671403	31
BP	GO:0007442	hindgut morphogenesis	3/751	7/18614	0.00202674	0.02222362	0.01671403	3
BP	GO:0010668	ectodermal cell differentiation	3/751	7/18614	0.00202674	0.02222362	0.01671403	3
BP	GO:0014060	regulation of epinephrine secretion	3/751	7/18614	0.00202674	0.02222362	0.01671403	3
BP	GO:0014062	regulation of serotonin secretion	3/751	7/18614	0.00202674	0.02222362	0.01671403	3
BP	GO:0021546	rhombomere development	3/751	7/18614	0.00202674	0.02222362	0.01671403	3
BP	GO:0030952	establishment or maintenance of cytoskeleton polarity	3/751	7/18614	0.00202674	0.02222362	0.01671403	3
BP	GO:0031580	membrane raft distribution	3/751	7/18614	0.00202674	0.02222362	0.01671403	3
BP	GO:0045588	positive regulation of gamma-delta T cell differentiation	3/751	7/18614	0.00202674	0.02222362	0.01671403	3
BP	GO:0048242	epinephrine secretion	3/751	7/18614	0.00202674	0.02222362	0.01671403	3
BP	GO:0061312	BMP signaling pathway involved in heart development	3/751	7/18614	0.00202674	0.02222362	0.01671403	3
BP	GO:0071420	cellular response to histamine	3/751	7/18614	0.00202674	0.02222362	0.01671403	3
BP	GO:1903490	positive regulation of mitotic cytokinesis	3/751	7/18614	0.00202674	0.02222362	0.01671403	3
BP	GO:0010038	response to metal ion	27/751	367/18614	0.00203763	0.02230619	0.01677613	27
BP	GO:0014065	phosphatidylinositol 3-kinase signaling	14/751	144/18614	0.00208208	0.02275518	0.01711381	14
BP	GO:0030509	BMP signaling pathway	16/751	176/18614	0.00208757	0.0227776	0.01713067	16

BP	GO:0002821	positive regulation of adaptive immune response	13/751	129/18614	0.002155	0.02335709	0.01756649	13
BP	GO:0048839	inner ear development	17/751	193/18614	0.00215718	0.02335709	0.01756649	17
BP	GO:0033238	regulation of amine metabolic process	6/751	34/18614	0.00216184	0.02335709	0.01756649	6
BP	GO:0043372	positive regulation of CD4-positive, alpha-beta T cell differentiation	6/751	34/18614	0.00216184	0.02335709	0.01756649	6
BP	GO:0090322	regulation of superoxide metabolic process	6/751	34/18614	0.00216184	0.02335709	0.01756649	6
BP	GO:0098664	G protein-coupled serotonin receptor signaling pathway	6/751	34/18614	0.00216184	0.02335709	0.01756649	6
BP	GO:0006956	complement activation	8/751	58/18614	0.00217565	0.02342985	0.01762121	8
BP	GO:0042733	embryonic digit morphogenesis	8/751	58/18614	0.00217565	0.02342985	0.01762121	8
BP	GO:0034331	cell junction maintenance	7/751	46/18614	0.00229608	0.02464652	0.01853626	7
BP	GO:0055010	ventricular cardiac muscle tissue morphogenesis	7/751	46/18614	0.00229608	0.02464652	0.01853626	7
BP	GO:0007052	mitotic spindle organization	13/751	130/18614	0.0023084	0.02471718	0.0185894	13
BP	GO:0060541	respiratory system development	18/751	211/18614	0.00231012	0.02471718	0.0185894	18
BP	GO:0051656	establishment of organelle localization	31/751	445/18614	0.0023258	0.02484485	0.01868541	31
BP	GO:0032715	negative regulation of interleukin-6 production	9/751	72/18614	0.00236528	0.02507243	0.01885658	9
BP	GO:0031338	regulation of vesicle fusion	5/751	24/18614	0.00236604	0.02507243	0.01885658	5
BP	GO:0060143	positive regulation of syncytium formation by plasma membrane fusion	5/751	24/18614	0.00236604	0.02507243	0.01885658	5
BP	GO:0060571	morphogenesis of an epithelial fold	5/751	24/18614	0.00236604	0.02507243	0.01885658	5
BP	GO:0060907	positive regulation of macrophage cytokine production	5/751	24/18614	0.00236604	0.02507243	0.01885658	5
BP	GO:0045165	cell fate commitment	22/751	281/18614	0.00240348	0.02542848	0.01912436	22
BP	GO:0002763	positive regulation of myeloid leukocyte differentiation	8/751	59/18614	0.00242977	0.02562481	0.01927201	8
BP	GO:1903053	regulation of extracellular matrix organization	8/751	59/18614	0.00242977	0.02562481	0.01927201	8
BP	GO:0001771	immunological synapse formation	4/751	15/18614	0.00251281	0.02615232	0.01966874	4
BP	GO:0010820	positive regulation of T cell chemotaxis	4/751	15/18614	0.00251281	0.02615232	0.01966874	4

BP	GO:0043374	CD8-positive, alpha-beta T cell differentiation	4/751	15/18614	0.00251281	0.02615232	0.01966874	4
BP	GO:0060099	regulation of phagocytosis, engulfment	4/751	15/18614	0.00251281	0.02615232	0.01966874	4
BP	GO:0060572	morphogenesis of an epithelial bud	4/751	15/18614	0.00251281	0.02615232	0.01966874	4
BP	GO:0098883	synapse pruning	4/751	15/18614	0.00251281	0.02615232	0.01966874	4
BP	GO:1905153	regulation of membrane invagination	4/751	15/18614	0.00251281	0.02615232	0.01966874	4
BP	GO:0032633	interleukin-4 production	6/751	35/18614	0.0025215	0.02615232	0.01966874	6
BP	GO:0032673	regulation of interleukin-4 production	6/751	35/18614	0.0025215	0.02615232	0.01966874	6
BP	GO:0061081	positive regulation of myeloid leukocyte cytokine production involved in immune response	6/751	35/18614	0.0025215	0.02615232	0.01966874	6
BP	GO:0045667	regulation of osteoblast differentiation	14/751	147/18614	0.00252323	0.02615232	0.01966874	14
BP	GO:0050920	regulation of chemotaxis	19/751	230/18614	0.00255205	0.02640978	0.01986237	19
BP	GO:0001774	microglial cell activation	7/751	47/18614	0.00260572	0.02683934	0.02018544	7
BP	GO:0002920	regulation of humoral immune response	7/751	47/18614	0.00260572	0.02683934	0.02018544	7
BP	GO:0043277	apoptotic cell clearance	7/751	47/18614	0.00260572	0.02683934	0.02018544	7
BP	GO:0002011	morphogenesis of an epithelial sheet	8/751	60/18614	0.00270655	0.02779147	0.02090152	8
BP	GO:0021545	cranial nerve development	8/751	60/18614	0.00270655	0.02779147	0.02090152	8
BP	GO:0021510	spinal cord development	11/751	102/18614	0.00271355	0.02782019	0.02092312	11
BP	GO:0099072	regulation of postsynaptic membrane neurotransmitter receptor levels	10/751	88/18614	0.00284071	0.02907883	0.02186972	10
BP	GO:0031579	membrane raft organization	5/751	25/18614	0.00286064	0.0291029	0.02188783	5
BP	GO:0043302	positive regulation of leukocyte degranulation	5/751	25/18614	0.00286064	0.0291029	0.02188783	5
BP	GO:0061339	establishment or maintenance of monopolar cell polarity	5/751	25/18614	0.00286064	0.0291029	0.02188783	5
BP	GO:2000311	regulation of AMPA receptor activity	5/751	25/18614	0.00286064	0.0291029	0.02188783	5
BP	GO:0001649	osteoblast differentiation	20/751	250/18614	0.00289624	0.02941995	0.02212627	20
BP	GO:0033687	osteoblast proliferation	6/751	36/18614	0.00292428	0.02962796	0.02228271	6
BP	GO:0042100	B cell proliferation	11/751	103/18614	0.00293014	0.02962796	0.02228271	11
BP	GO:0045639	positive regulation of myeloid cell differentiation	11/751	103/18614	0.00293014	0.02962796	0.02228271	11
BP	GO:0003012	muscle system process	31/751	452/18614	0.0029428	0.02965714	0.02230466	31

BP	GO:0006953	acute-phase response	7/751	48/18614	0.00294646	0.02965714	0.02230466	7
BP	GO:0046189	phenol-containing compound biosynthetic process	7/751	48/18614	0.00294646	0.02965714	0.02230466	7
BP	GO:0034113	heterotypic cell-cell adhesion	8/751	61/18614	0.00300734	0.03017821	0.02269655	8
BP	GO:0046456	icosanoid biosynthetic process	8/751	61/18614	0.00300734	0.03017821	0.02269655	8
BP	GO:0043367	CD4-positive, alpha-beta T cell differentiation	10/751	89/18614	0.0030875	0.03091222	0.02324858	10
BP	GO:0019221	cytokine-mediated signaling pathway	33/751	492/18614	0.00311155	0.03091222	0.02324858	33
BP	GO:0030278	regulation of ossification	12/751	119/18614	0.00312412	0.03091222	0.02324858	12
BP	GO:0045669	positive regulation of osteoblast differentiation	9/751	75/18614	0.00313686	0.03091222	0.02324858	9
BP	GO:0042421	norepinephrine biosynthetic process	3/751	8/18614	0.00314583	0.03091222	0.02324858	3
BP	GO:0043376	regulation of CD8-positive, alpha-beta T cell differentiation	3/751	8/18614	0.00314583	0.03091222	0.02324858	3
BP	GO:0048570	notochord morphogenesis	3/751	8/18614	0.00314583	0.03091222	0.02324858	3
BP	GO:0051665	membrane raft localization	3/751	8/18614	0.00314583	0.03091222	0.02324858	3
BP	GO:0060600	dichotomous subdivision of an epithelial terminal unit	3/751	8/18614	0.00314583	0.03091222	0.02324858	3
BP	GO:0061525	hindgut development	3/751	8/18614	0.00314583	0.03091222	0.02324858	3
BP	GO:0090091	positive regulation of extracellular matrix disassembly	3/751	8/18614	0.00314583	0.03091222	0.02324858	3
BP	GO:0098700	neurotransmitter loading into synaptic vesicle	3/751	8/18614	0.00314583	0.03091222	0.02324858	3
BP	GO:1902423	regulation of attachment of mitotic spindle microtubules to kinetochore	3/751	8/18614	0.00314583	0.03091222	0.02324858	3
BP	GO:1902425	positive regulation of attachment of mitotic spindle microtubules to kinetochore	3/751	8/18614	0.00314583	0.03091222	0.02324858	3
BP	GO:0060348	bone development	19/751	235/18614	0.00324073	0.03150752	0.02369631	19
BP	GO:0071356	cellular response to tumor necrosis factor	19/751	235/18614	0.00324073	0.03150752	0.02369631	19
BP	GO:0000212	meiotic spindle organization	4/751	16/18614	0.00324447	0.03150752	0.02369631	4
BP	GO:0016045	detection of bacterium	4/751	16/18614	0.00324447	0.03150752	0.02369631	4
BP	GO:0034134	toll-like receptor 2 signaling pathway	4/751	16/18614	0.00324447	0.03150752	0.02369631	4

BP	GO:0045579	positive regulation of B cell differentiation	4/751	16/18614	0.00324447	0.03150752	0.02369631	4
BP	GO:0045779	negative regulation of bone resorption	4/751	16/18614	0.00324447	0.03150752	0.02369631	4
BP	GO:1901741	positive regulation of myoblast fusion	4/751	16/18614	0.00324447	0.03150752	0.02369631	4
BP	GO:0033628	regulation of cell adhesion mediated by integrin	7/751	49/18614	0.00332027	0.03214932	0.02417899	7
BP	GO:0140895	cell surface toll-like receptor signaling pathway	7/751	49/18614	0.00332027	0.03214932	0.02417899	7
BP	GO:0032355	response to estradiol	12/751	120/18614	0.00334736	0.03236434	0.0243407	12
BP	GO:0010934	macrophage cytokine production	6/751	37/18614	0.00337326	0.03247256	0.0244221	6
BP	GO:0010935	regulation of macrophage cytokine production	6/751	37/18614	0.00337326	0.03247256	0.0244221	6
BP	GO:0014059	regulation of dopamine secretion	6/751	37/18614	0.00337326	0.03247256	0.0244221	6
BP	GO:0010466	negative regulation of peptidase activity	19/751	236/18614	0.00339555	0.03247893	0.02442689	19
BP	GO:0001911	negative regulation of leukocyte mediated cytotoxicity	5/751	26/18614	0.00342581	0.03247893	0.02442689	5
BP	GO:0002407	dendritic cell chemotaxis	5/751	26/18614	0.00342581	0.03247893	0.02442689	5
BP	GO:0002438	acute inflammatory response to antigenic stimulus	5/751	26/18614	0.00342581	0.03247893	0.02442689	5
BP	GO:0006817	phosphate ion transport	5/751	26/18614	0.00342581	0.03247893	0.02442689	5
BP	GO:0046629	gamma-delta T cell activation	5/751	26/18614	0.00342581	0.03247893	0.02442689	5
BP	GO:0099637	neurotransmitter receptor transport	5/751	26/18614	0.00342581	0.03247893	0.02442689	5
BP	GO:1901623	regulation of lymphocyte chemotaxis	5/751	26/18614	0.00342581	0.03247893	0.02442689	5
BP	GO:0007612	learning	14/751	152/18614	0.00342787	0.03247893	0.02442689	14
BP	GO:0072006	nephron development	14/751	152/18614	0.00342787	0.03247893	0.02442689	14
BP	GO:1904064	positive regulation of cation transmembrane transport	14/751	152/18614	0.00342787	0.03247893	0.02442689	14
BP	GO:0043588	skin development	23/751	308/18614	0.00351422	0.03324951	0.02500642	23
BP	GO:0002833	positive regulation of response to biotic stimulus	24/751	327/18614	0.00362082	0.03420925	0.02572823	24
BP	GO:0035270	endocrine system development	13/751	137/18614	0.00365186	0.03445336	0.02591182	13
BP	GO:1902074	response to salt	27/751	383/18614	0.00367469	0.03461949	0.02603677	27
BP	GO:0051784	negative regulation of nuclear division	8/751	63/18614	0.00368647	0.03463194	0.02604613	8

BP	GO:0060393	regulation of pathway-restricted SMAD protein phosphorylation	8/751	63/18614	0.00368647	0.03463194	0.02604613	8
BP	GO:0002269	leukocyte activation involved in inflammatory response	7/751	50/18614	0.00372917	0.03488456	0.02623612	7
BP	GO:0010862	positive regulation of pathway-restricted SMAD protein phosphorylation	7/751	50/18614	0.00372917	0.03488456	0.02623612	7
BP	GO:0090102	cochlea development	7/751	50/18614	0.00372917	0.03488456	0.02623612	7
BP	GO:0090100	positive regulation of transmembrane receptor protein serine/threonine kinase signaling pathway	12/751	122/18614	0.00383248	0.03580042	0.02692493	12
BP	GO:0050729	positive regulation of inflammatory response	14/751	154/18614	0.00385691	0.03597793	0.02705843	14
BP	GO:0014046	dopamine secretion	6/751	38/18614	0.00387158	0.03606397	0.02712314	6
BP	GO:0007160	cell-matrix adhesion	19/751	239/18614	0.00389741	0.03625358	0.02726574	19
BP	GO:0001656	metanephros development	10/751	92/18614	0.00393173	0.03647035	0.02742877	10
BP	GO:1901216	positive regulation of neuron death	10/751	92/18614	0.00393173	0.03647035	0.02742877	10
BP	GO:0034612	response to tumor necrosis factor	20/751	257/18614	0.00395956	0.03667717	0.02758431	20
BP	GO:0050708	regulation of protein secretion	21/751	275/18614	0.00398678	0.03687771	0.02773514	21
BP	GO:0032753	positive regulation of interleukin-4 production	5/751	27/18614	0.00406694	0.03722657	0.02799751	5
BP	GO:0034143	regulation of toll-like receptor 4 signaling pathway	5/751	27/18614	0.00406694	0.03722657	0.02799751	5
BP	GO:0030199	collagen fibril organization	8/751	64/18614	0.00406765	0.03722657	0.02799751	8
BP	GO:0032615	interleukin-12 production	8/751	64/18614	0.00406765	0.03722657	0.02799751	8
BP	GO:0032655	regulation of interleukin-12 production	8/751	64/18614	0.00406765	0.03722657	0.02799751	8
BP	GO:0045453	bone resorption	8/751	64/18614	0.00406765	0.03722657	0.02799751	8
BP	GO:0071384	cellular response to corticosteroid stimulus	8/751	64/18614	0.00406765	0.03722657	0.02799751	8
BP	GO:0050803	regulation of synapse structure or activity	19/751	240/18614	0.00407777	0.03722657	0.02799751	19
BP	GO:0002220	innate immune response activating cell surface receptor signaling pathway	9/751	78/18614	0.00409419	0.03722657	0.02799751	9
BP	GO:0002437	inflammatory response to antigenic stimulus	9/751	78/18614	0.00409419	0.03722657	0.02799751	9

BP	GO:0032720	negative regulation of tumor necrosis factor production	9/751	78/18614	0.00409419	0.03722657	0.02799751	9
BP	GO:0036005	response to macrophage colony-stimulating factor	4/751	17/18614	0.00410881	0.03722657	0.02799751	4
BP	GO:0051382	kinetochore assembly	4/751	17/18614	0.00410881	0.03722657	0.02799751	4
BP	GO:0060080	inhibitory postsynaptic potential	4/751	17/18614	0.00410881	0.03722657	0.02799751	4
BP	GO:0070206	protein trimerization	4/751	17/18614	0.00410881	0.03722657	0.02799751	4
BP	GO:0034341	response to type II interferon	13/751	139/18614	0.00413454	0.03740856	0.02813438	13
BP	GO:0045778	positive regulation of ossification	7/751	51/18614	0.0041752	0.0376735	0.02833364	7
BP	GO:0061082	myeloid leukocyte cytokine production	7/751	51/18614	0.0041752	0.0376735	0.02833364	7
BP	GO:0002831	regulation of response to biotic stimulus	31/751	463/18614	0.00419306	0.03778316	0.02841611	31
BP	GO:0051781	positive regulation of cell division	10/751	93/18614	0.00425044	0.0381963	0.02872682	10
BP	GO:2001251	negative regulation of chromosome organization	10/751	93/18614	0.00425044	0.0381963	0.02872682	10
BP	GO:0051051	negative regulation of transport	32/751	483/18614	0.0042823	0.03843047	0.02890295	32
BP	GO:0007210	serotonin receptor signaling pathway	6/751	39/18614	0.00442239	0.03942057	0.02964758	6
BP	GO:0017158	regulation of calcium ion-dependent exocytosis	6/751	39/18614	0.00442239	0.03942057	0.02964758	6
BP	GO:0042401	biogenic amine biosynthetic process	6/751	39/18614	0.00442239	0.03942057	0.02964758	6
BP	GO:0045730	respiratory burst	6/751	39/18614	0.00442239	0.03942057	0.02964758	6
BP	GO:0097242	amyloid-beta clearance	6/751	39/18614	0.00442239	0.03942057	0.02964758	6
BP	GO:1902106	negative regulation of leukocyte differentiation	11/751	109/18614	0.00453927	0.03959041	0.02977531	11
BP	GO:0070482	response to oxygen levels	24/751	333/18614	0.00454279	0.03959041	0.02977531	24
BP	GO:0002579	positive regulation of antigen processing and presentation	3/751	9/18614	0.00457797	0.03959041	0.02977531	3
BP	GO:0043615	astrocyte cell migration	3/751	9/18614	0.00457797	0.03959041	0.02977531	3
BP	GO:0044851	hair cycle phase	3/751	9/18614	0.00457797	0.03959041	0.02977531	3
BP	GO:0045586	regulation of gamma-delta T cell differentiation	3/751	9/18614	0.00457797	0.03959041	0.02977531	3
BP	GO:0048819	regulation of hair follicle maturation	3/751	9/18614	0.00457797	0.03959041	0.02977531	3
BP	GO:0060052	neurofilament cytoskeleton organization	3/751	9/18614	0.00457797	0.03959041	0.02977531	3
BP	GO:0060926	cardiac pacemaker cell development	3/751	9/18614	0.00457797	0.03959041	0.02977531	3
BP	GO:0070305	response to cGMP	3/751	9/18614	0.00457797	0.03959041	0.02977531	3

BP	GO:0090306	meiotic spindle assembly	3/751	9/18614	0.00457797	0.03959041	0.02977531	3
BP	GO:0032692	negative regulation of interleukin-1 production	7/751	52/18614	0.00466045	0.03959041	0.02977531	7
BP	GO:0006690	icosanoid metabolic process	12/751	125/18614	0.00466462	0.03959041	0.02977531	12
BP	GO:0031623	receptor internalization	12/751	125/18614	0.00466462	0.03959041	0.02977531	12
BP	GO:1903522	regulation of blood circulation	20/751	261/18614	0.00470074	0.03959041	0.02977531	20
BP	GO:0001798	positive regulation of type IIa hypersensitivity	2/751	3/18614	0.00474631	0.03959041	0.02977531	2
BP	GO:0002469	myeloid dendritic cell antigen processing and presentation	2/751	3/18614	0.00474631	0.03959041	0.02977531	2
BP	GO:0002538	arachidonic acid metabolite production involved in inflammatory response	2/751	3/18614	0.00474631	0.03959041	0.02977531	2
BP	GO:0002540	leukotriene production involved in inflammatory response	2/751	3/18614	0.00474631	0.03959041	0.02977531	2
BP	GO:0002894	positive regulation of type II hypersensitivity	2/751	3/18614	0.00474631	0.03959041	0.02977531	2
BP	GO:0003213	cardiac right atrium morphogenesis	2/751	3/18614	0.00474631	0.03959041	0.02977531	2
BP	GO:0021569	rhombomere 3 development	2/751	3/18614	0.00474631	0.03959041	0.02977531	2
BP	GO:0030886	negative regulation of myeloid dendritic cell activation	2/751	3/18614	0.00474631	0.03959041	0.02977531	2
BP	GO:0031444	slow-twitch skeletal muscle fiber contraction	2/751	3/18614	0.00474631	0.03959041	0.02977531	2
BP	GO:0033693	neurofilament bundle assembly	2/751	3/18614	0.00474631	0.03959041	0.02977531	2
BP	GO:0043309	regulation of eosinophil degranulation	2/751	3/18614	0.00474631	0.03959041	0.02977531	2
BP	GO:0045726	positive regulation of integrin biosynthetic process	2/751	3/18614	0.00474631	0.03959041	0.02977531	2
BP	GO:0048619	embryonic hindgut morphogenesis	2/751	3/18614	0.00474631	0.03959041	0.02977531	2
BP	GO:0051795	positive regulation of timing of catagen	2/751	3/18614	0.00474631	0.03959041	0.02977531	2
BP	GO:0060535	trachea cartilage morphogenesis	2/751	3/18614	0.00474631	0.03959041	0.02977531	2
BP	GO:0060927	cardiac pacemaker cell fate commitment	2/751	3/18614	0.00474631	0.03959041	0.02977531	2
BP	GO:0061110	dense core granule biogenesis	2/751	3/18614	0.00474631	0.03959041	0.02977531	2

BP	GO:0070093	negative regulation of glucagon secretion	2/751	3/18614	0.00474631	0.03959041	0.02977531	2
BP	GO:0071725	response to triacyl bacterial lipopeptide	2/751	3/18614	0.00474631	0.03959041	0.02977531	2
BP	GO:0071727	cellular response to triacyl bacterial lipopeptide	2/751	3/18614	0.00474631	0.03959041	0.02977531	2
BP	GO:0099185	postsynaptic intermediate filament cytoskeleton organization	2/751	3/18614	0.00474631	0.03959041	0.02977531	2
BP	GO:0150064	vertebrate eye-specific patterning	2/751	3/18614	0.00474631	0.03959041	0.02977531	2
BP	GO:0150102	negative regulation of monocyte activation	2/751	3/18614	0.00474631	0.03959041	0.02977531	2
BP	GO:1900159	positive regulation of bone mineralization involved in bone maturation	2/751	3/18614	0.00474631	0.03959041	0.02977531	2
BP	GO:1900623	regulation of monocyte aggregation	2/751	3/18614	0.00474631	0.03959041	0.02977531	2
BP	GO:1900625	positive regulation of monocyte aggregation	2/751	3/18614	0.00474631	0.03959041	0.02977531	2
BP	GO:1901079	positive regulation of relaxation of muscle	2/751	3/18614	0.00474631	0.03959041	0.02977531	2
BP	GO:1903615	positive regulation of protein tyrosine phosphatase activity	2/751	3/18614	0.00474631	0.03959041	0.02977531	2
BP	GO:1904092	regulation of autophagic cell death	2/751	3/18614	0.00474631	0.03959041	0.02977531	2
BP	GO:1905581	positive regulation of low-density lipoprotein particle clearance	2/751	3/18614	0.00474631	0.03959041	0.02977531	2
BP	GO:1990680	response to melanocyte-stimulating hormone	2/751	3/18614	0.00474631	0.03959041	0.02977531	2
BP	GO:2000361	regulation of prostaglandin-E synthase activity	2/751	3/18614	0.00474631	0.03959041	0.02977531	2
BP	GO:2000363	positive regulation of prostaglandin-E synthase activity	2/751	3/18614	0.00474631	0.03959041	0.02977531	2
BP	GO:2000705	regulation of dense core granule biogenesis	2/751	3/18614	0.00474631	0.03959041	0.02977531	2
BP	GO:2001055	positive regulation of mesenchymal cell apoptotic process	2/751	3/18614	0.00474631	0.03959041	0.02977531	2

BP	GO:2001189	negative regulation of T cell activation via T cell receptor contact with antigen bound to MHC molecule on antigen presenting cell	2/751	3/18614	0.00474631	0.03959041	0.02977531	2
BP	GO:0150146	cell junction disassembly	5/751	28/18614	0.00478936	0.03981414	0.02994358	5
BP	GO:1903055	positive regulation of extracellular matrix organization	5/751	28/18614	0.00478936	0.03981414	0.02994358	5
BP	GO:0007163	establishment or maintenance of cell polarity	18/751	226/18614	0.00479116	0.03981414	0.02994358	18
BP	GO:2001259	positive regulation of cation channel activity	8/751	66/18614	0.00492048	0.04083753	0.03071325	8
BP	GO:0001910	regulation of leukocyte mediated cytotoxicity	10/751	95/18614	0.00494881	0.04102121	0.0308514	10
BP	GO:0045471	response to ethanol	12/751	126/18614	0.00497203	0.04116219	0.03095742	12
BP	GO:0070997	neuron death	26/751	373/18614	0.0049927	0.0412817	0.03104731	26
BP	GO:0009309	amine biosynthetic process	6/751	40/18614	0.00502886	0.04152883	0.03123317	6
BP	GO:0002830	positive regulation of type 2 immune response	4/751	18/18614	0.0051162	0.04183281	0.03146179	4
BP	GO:0010755	regulation of plasminogen activation	4/751	18/18614	0.0051162	0.04183281	0.03146179	4
BP	GO:0034501	protein localization to kinetochore	4/751	18/18614	0.0051162	0.04183281	0.03146179	4
BP	GO:0046851	negative regulation of bone remodeling	4/751	18/18614	0.0051162	0.04183281	0.03146179	4
BP	GO:0061450	trophoblast cell migration	4/751	18/18614	0.0051162	0.04183281	0.03146179	4
BP	GO:0090594	inflammatory response to wounding	4/751	18/18614	0.0051162	0.04183281	0.03146179	4
BP	GO:1901978	positive regulation of cell cycle checkpoint	4/751	18/18614	0.0051162	0.04183281	0.03146179	4
BP	GO:1903083	protein localization to condensed chromosome	4/751	18/18614	0.0051162	0.04183281	0.03146179	4
BP	GO:0014902	myotube differentiation	12/751	127/18614	0.00529538	0.04324449	0.03252349	12
BP	GO:0045807	positive regulation of endocytosis	10/751	96/18614	0.00533017	0.04347501	0.03269686	10
BP	GO:0060389	pathway-restricted SMAD protein phosphorylation	8/751	67/18614	0.0053951	0.0439505	0.03305447	8
BP	GO:0010469	regulation of signaling receptor activity	15/751	177/18614	0.00547106	0.04451457	0.0334787	15
BP	GO:1901990	regulation of mitotic cell cycle phase transition	25/751	357/18614	0.00550637	0.04474684	0.03365338	25
BP	GO:0007548	sex differentiation	21/751	283/18614	0.00552296	0.04482664	0.0337134	21
BP	GO:0048880	sensory system development	27/751	395/18614	0.00553438	0.04486437	0.03374178	27

BP	GO:0098657	import into cell	20/751	265/18614	0.00555305	0.04488857	0.03375998	20
BP	GO:0008584	male gonad development	13/751	144/18614	0.0055691	0.04488857	0.03375998	13
BP	GO:0035249	synaptic transmission, glutamatergic	11/751	112/18614	0.0055721	0.04488857	0.03375998	11
BP	GO:0003156	regulation of animal organ formation	5/751	29/18614	0.00559836	0.04488857	0.03375998	5
BP	GO:0030878	thyroid gland development	5/751	29/18614	0.00559836	0.04488857	0.03375998	5
BP	GO:0031342	negative regulation of cell killing	5/751	29/18614	0.00559836	0.04488857	0.03375998	5
BP	GO:0060037	pharyngeal system development	5/751	29/18614	0.00559836	0.04488857	0.03375998	5
BP	GO:0090025	regulation of monocyte chemotaxis	5/751	29/18614	0.00559836	0.04488857	0.03375998	5
BP	GO:0099170	postsynaptic modulation of chemical synaptic transmission	5/751	29/18614	0.00559836	0.04488857	0.03375998	5
BP	GO:0071214	cellular response to abiotic stimulus	24/751	339/18614	0.00565305	0.0452176	0.03400743	24
BP	GO:0104004	cellular response to environmental stimulus	24/751	339/18614	0.00565305	0.0452176	0.03400743	24
BP	GO:0002791	regulation of peptide secretion	16/751	195/18614	0.00572373	0.04572769	0.03439106	16
BP	GO:0002709	regulation of T cell mediated immunity	10/751	97/18614	0.00573414	0.0457557	0.03441213	10
BP	GO:0046546	development of primary male sexual characteristics	13/751	145/18614	0.0058988	0.04694031	0.03530305	13
BP	GO:0061180	mammary gland epithelium development	8/751	68/18614	0.00590386	0.04694031	0.03530305	8
BP	GO:1902893	regulation of miRNA transcription	8/751	68/18614	0.00590386	0.04694031	0.03530305	8
BP	GO:0002062	chondrocyte differentiation	11/751	113/18614	0.00595474	0.04723143	0.035522	11
BP	GO:0044344	cellular response to fibroblast growth factor stimulus	11/751	113/18614	0.00595474	0.04723143	0.035522	11
BP	GO:0140013	meiotic nuclear division	16/751	196/18614	0.00600697	0.04758873	0.03579072	16
BP	GO:0002281	macrophage activation involved in immune response	4/751	19/18614	0.00627651	0.04932474	0.03709635	4
BP	GO:0010002	cardioblast differentiation	4/751	19/18614	0.00627651	0.04932474	0.03709635	4
BP	GO:0051797	regulation of hair follicle development	4/751	19/18614	0.00627651	0.04932474	0.03709635	4
BP	GO:0060039	pericardium development	4/751	19/18614	0.00627651	0.04932474	0.03709635	4
BP	GO:0090087	regulation of peptide transport	16/751	197/18614	0.00630145	0.04932474	0.03709635	16
BP	GO:0050878	regulation of body fluid levels	25/751	361/18614	0.00632097	0.04932474	0.03709635	25

BP	GO:0001915	negative regulation of T cell mediated cytotoxicity	3/751	10/18614	0.00634526	0.04932474	0.03709635	3
BP	GO:0003211	cardiac ventricle formation	3/751	10/18614	0.00634526	0.04932474	0.03709635	3
BP	GO:0019372	lipoxygenase pathway	3/751	10/18614	0.00634526	0.04932474	0.03709635	3
BP	GO:0035860	glial cell-derived neurotrophic factor receptor signaling pathway	3/751	10/18614	0.00634526	0.04932474	0.03709635	3
BP	GO:0048241	epinephrine transport	3/751	10/18614	0.00634526	0.04932474	0.03709635	3
BP	GO:0060536	cartilage morphogenesis	3/751	10/18614	0.00634526	0.04932474	0.03709635	3
BP	GO:0060920	cardiac pacemaker cell differentiation	3/751	10/18614	0.00634526	0.04932474	0.03709635	3
BP	GO:0098976	excitatory chemical synaptic transmission	3/751	10/18614	0.00634526	0.04932474	0.03709635	3
BP	GO:1902412	regulation of mitotic cytokinesis	3/751	10/18614	0.00634526	0.04932474	0.03709635	3
BP	GO:1990034	calcium ion export across plasma membrane	3/751	10/18614	0.00634526	0.04932474	0.03709635	3
BP	GO:0050796	regulation of insulin secretion	14/751	163/18614	0.00636063	0.04936248	0.03712474	14
BP	GO:0051703	biological process involved in intraspecies interaction between organisms	7/751	55/18614	0.00637248	0.04936248	0.03712474	7
BP	GO:0061900	glial cell activation	7/751	55/18614	0.00637248	0.04936248	0.03712474	7
BP	GO:0071542	dopaminergic neuron differentiation	6/751	42/18614	0.00642148	0.04968395	0.03736651	6
BP	GO:0061614	miRNA transcription	8/751	69/18614	0.00644828	0.04983311	0.03747869	8
CC	GO:0042613	MHC class II protein complex	13/789	17/19518	1.43E-15	8.45E-13	6.27E-13	13
CC	GO:0042611	MHC protein complex	13/789	25/19518	2.32E-12	6.84E-10	5.07E-10	13
CC	GO:0062023	collagen-containing extracellular matrix	48/789	415/19518	5.81E-11	1.14E-08	8.48E-09	48
CC	GO:0097060	synaptic membrane	43/789	390/19518	2.60E-09	3.83E-07	2.85E-07	43
CC	GO:0005604	basement membrane	18/789	84/19518	5.30E-09	6.26E-07	4.64E-07	18
CC	GO:0043025	neuronal cell body	49/789	500/19518	1.03E-08	1.01E-06	7.53E-07	49
CC	GO:0070821	tertiary granule membrane	16/789	73/19518	2.67E-08	1.91E-06	1.42E-06	16
CC	GO:0030667	secretory granule membrane	36/789	319/19518	2.85E-08	1.91E-06	1.42E-06	36
CC	GO:0045211	postsynaptic membrane	33/789	277/19518	2.92E-08	1.91E-06	1.42E-06	33
CC	GO:0034702	ion channel complex	34/789	299/19518	5.74E-08	3.39E-06	2.51E-06	34
CC	GO:0098553	lumenal side of endoplasmic reticulum membrane	10/789	29/19518	1.10E-07	5.88E-06	4.36E-06	10
CC	GO:0032590	dendrite membrane	11/789	41/19518	4.57E-07	2.25E-05	1.67E-05	11
CC	GO:0035579	specific granule membrane	16/789	91/19518	6.68E-07	3.03E-05	2.25E-05	16

CC	GO:0098644	complex of collagen trimers	8/789	21/19518	8.77E-07	3.69E-05	2.74E-05	8
CC	GO:0030658	transport vesicle membrane	27/789	234/19518	9.90E-07	3.90E-05	2.89E-05	27
CC	GO:0098576	lumenal side of membrane	10/789	36/19518	1.08E-06	3.97E-05	2.94E-05	10
CC	GO:0030666	endocytic vesicle membrane	24/789	196/19518	1.36E-06	4.73E-05	3.51E-05	24
CC	GO:0034707	chloride channel complex	12/789	55/19518	1.55E-06	5.08E-05	3.77E-05	12
CC	GO:0009897	external side of plasma membrane	39/789	426/19518	1.85E-06	5.73E-05	4.25E-05	39
CC	GO:0070820	tertiary granule	21/789	164/19518	3.01E-06	8.87E-05	6.58E-05	21
CC	GO:0032589	neuron projection membrane	12/789	60/19518	4.11E-06	0.00011534	8.56E-05	12
CC	GO:0043204	perikaryon	20/789	156/19518	5.05E-06	0.00013531	0.00010043	20
CC	GO:1902711	GABA-A receptor complex	7/789	19/19518	5.65E-06	0.00014499	0.00010761	7
CC	GO:0030669	clathrin-coated endocytic vesicle membrane	13/789	73/19518	6.38E-06	0.00015686	0.00011642	13
CC	GO:0042581	specific granule	20/789	160/19518	7.44E-06	0.00017549	0.00013025	20
CC	GO:1902495	transmembrane transporter complex	35/789	392/19518	1.07E-05	0.0002424	0.00017991	35
CC	GO:1902710	GABA receptor complex	7/789	21/19518	1.22E-05	0.00026551	0.00019706	7
CC	GO:1990351	transporter complex	36/789	418/19518	1.76E-05	0.00037159	0.00027579	36
CC	GO:0030133	transport vesicle	36/789	425/19518	2.52E-05	0.00051196	0.00037997	36
CC	GO:0030139	endocytic vesicle	31/789	347/19518	3.33E-05	0.00065476	0.00048596	31
CC	GO:0030665	clathrin-coated vesicle membrane	17/789	136/19518	3.53E-05	0.00067271	0.00049928	17
CC	GO:1904090	peptidase inhibitor complex	5/789	11/19518	4.02E-05	0.00074086	0.00054986	5
CC	GO:0031256	leading edge membrane	20/789	180/19518	4.24E-05	0.00075823	0.00056275	20
CC	GO:0032588	trans-Golgi network membrane	14/789	102/19518	6.08E-05	0.00105543	0.00078333	14
CC	GO:0045334	clathrin-coated endocytic vesicle	13/789	92/19518	8.15E-05	0.00137423	0.00101994	13
CC	GO:0097179	protease inhibitor complex	4/789	8/19518	0.00016285	0.0024636	0.00182847	4
CC	GO:0098642	network-forming collagen trimer	4/789	8/19518	0.00016285	0.0024636	0.00182847	4
CC	GO:0098645	collagen network	4/789	8/19518	0.00016285	0.0024636	0.00182847	4
CC	GO:0098651	basement membrane collagen trimer	4/789	8/19518	0.00016285	0.0024636	0.00182847	4
CC	GO:0005788	endoplasmic reticulum lumen	27/789	312/19518	0.00017777	0.00262211	0.00194612	27
CC	GO:0008328	ionotropic glutamate receptor complex	8/789	41/19518	0.00020092	0.00283335	0.00210289	8
CC	GO:0012507	ER to Golgi transport vesicle membrane	10/789	63/19518	0.0002017	0.00283335	0.00210289	10
CC	GO:0060076	excitatory synapse	10/789	64/19518	0.00023051	0.00316287	0.00234747	10

CC	GO:0099160	postsynaptic intermediate filament cytoskeleton	3/789	4/19518	0.00025532	0.00342355	0.00254094	3
CC	GO:0005883	neurofilament	4/789	9/19518	0.00028374	0.0037202	0.00276111	4
CC	GO:0098978	glutamatergic synapse	32/789	410/19518	0.00031218	0.00400408	0.0029718	32
CC	GO:0030134	COPII-coated ER to Golgi transport vesicle	12/789	93/19518	0.00036135	0.00453614	0.00336669	12
CC	GO:0150034	distal axon	24/789	277/19518	0.00039144	0.00481142	0.00357101	24
CC	GO:0098878	neurotransmitter receptor complex	8/789	46/19518	0.00045917	0.00549662	0.00407956	8
CC	GO:0031253	cell projection membrane	28/789	349/19518	0.00046967	0.00549662	0.00407956	28
CC	GO:0030136	clathrin-coated vesicle	20/789	215/19518	0.00047513	0.00549662	0.00407956	20
CC	GO:0032281	AMPA glutamate receptor complex	6/789	26/19518	0.00049225	0.00558518	0.00414529	6
CC	GO:0098982	GABA-ergic synapse	11/789	84/19518	0.00055337	0.00616018	0.00457205	11
CC	GO:0098802	plasma membrane signaling receptor complex	26/789	319/19518	0.00058321	0.00637212	0.00472935	26
CC	GO:0005581	collagen trimer	11/789	85/19518	0.00061286	0.00657431	0.00487942	11
CC	GO:0098981	cholinergic synapse	4/789	11/19518	0.00069643	0.00733734	0.00544574	4
CC	GO:0005583	fibrillar collagen trimer	4/789	12/19518	0.00101134	0.0102878	0.00763555	4
CC	GO:0098643	banded collagen fibril	4/789	12/19518	0.00101134	0.0102878	0.00763555	4
CC	GO:0031091	platelet alpha granule	11/789	91/19518	0.00109249	0.01092486	0.00810837	11
CC	GO:0005587	collagen type IV trimer	3/789	6/19518	0.00120085	0.01161476	0.00862041	3
CC	GO:0032133	chromosome passenger complex	3/789	6/19518	0.00120085	0.01161476	0.00862041	3
CC	GO:0000940	outer kinetochore	4/789	13/19518	0.00141434	0.01319119	0.00979043	4
CC	GO:0031093	platelet alpha granule lumen	9/789	67/19518	0.00144096	0.01319119	0.00979043	9
CC	GO:0045121	membrane raft	25/789	323/19518	0.00152646	0.01319119	0.00979043	25
CC	GO:0001772	immunological synapse	7/789	43/19518	0.00155284	0.01319119	0.00979043	7
CC	GO:0098857	membrane microdomain	25/789	324/19518	0.00159265	0.01319119	0.00979043	25
CC	GO:0034687	integrin alphaL-beta2 complex	2/789	2/19518	0.00163213	0.01319119	0.00979043	2
CC	GO:0034688	integrin alphaM-beta2 complex	2/789	2/19518	0.00163213	0.01319119	0.00979043	2
CC	GO:0035354	Toll-like receptor 1-Toll-like receptor 2 protein complex	2/789	2/19518	0.00163213	0.01319119	0.00979043	2
CC	GO:0035355	Toll-like receptor 2-Toll-like receptor 6 protein complex	2/789	2/19518	0.00163213	0.01319119	0.00979043	2
CC	GO:0035692	macrophage migration inhibitory factor receptor complex	2/789	2/19518	0.00163213	0.01319119	0.00979043	2
CC	GO:0070554	synaptobrevin 2-SNAP-25-syntaxin-3-complexin complex	2/789	2/19518	0.00163213	0.01319119	0.00979043	2
CC	GO:1990665	AnxA2-p11 complex	2/789	2/19518	0.00163213	0.01319119	0.00979043	2
CC	GO:0005802	trans-Golgi network	21/789	255/19518	0.00166605	0.01328334	0.00985882	21

CC	GO:0098636	protein complex involved in cell adhesion	8/789	57/19518	0.00196839	0.01548469	0.01149265	8
CC	GO:0031252	cell leading edge	30/789	422/19518	0.0020757	0.01611401	0.01195973	30
CC	GO:0099634	postsynaptic specialization membrane	13/789	129/19518	0.00219573	0.01682442	0.01248699	13
CC	GO:0001533	cornified envelope	8/789	59/19518	0.00246127	0.01861732	0.01381768	8
CC	GO:0032591	dendritic spine membrane	4/789	15/19518	0.00253132	0.01890478	0.01403102	4
CC	GO:0051233	spindle midzone	6/789	36/19518	0.0029542	0.02178722	0.01617035	6
CC	GO:0098690	glycinergic synapse	3/789	8/19518	0.00316372	0.02304439	0.01710342	3
CC	GO:0030662	coated vesicle membrane	17/789	202/19518	0.00354282	0.02549103	0.0189193	17
CC	GO:0099572	postsynaptic specialization	26/789	364/19518	0.00374005	0.02658592	0.01973192	26
CC	GO:0000779	condensed chromosome, centromeric region	15/789	170/19518	0.00384085	0.0269774	0.02002247	15
CC	GO:0030135	coated vesicle	23/789	312/19518	0.00422644	0.02933649	0.02177338	23
CC	GO:0030672	synaptic vesicle membrane	12/789	125/19518	0.00474307	0.03194553	0.0237098	12
CC	GO:0099501	exocytic vesicle membrane	12/789	125/19518	0.00474307	0.03194553	0.0237098	12
CC	GO:0031523	Myb complex	2/789	3/19518	0.00476476	0.03194553	0.0237098	2
CC	GO:0009898	cytoplasmic side of plasma membrane	14/789	159/19518	0.0052186	0.03459522	0.02567638	14
CC	GO:0032809	neuronal cell body membrane	5/789	29/19518	0.00564618	0.03701387	0.02747149	5
CC	GO:0044309	neuron spine	15/789	178/19518	0.0058708	0.03806344	0.02825047	15
CC	GO:0044306	neuron projection terminus	12/789	129/19518	0.00609087	0.03905603	0.02898717	12
CC	GO:0034774	secretory granule lumen	23/789	322/19518	0.00615629	0.03905603	0.02898717	23
CC	GO:0099571	postsynaptic cytoskeleton	3/789	10/19518	0.00638053	0.04004802	0.02972342	3
CC	GO:0043679	axon terminus	11/789	114/19518	0.00645555	0.04009234	0.02975631	11
CC	GO:0042734	presynaptic membrane	14/789	164/19518	0.00682678	0.0413773	0.03071001	14
CC	GO:0060205	cytoplasmic vesicle lumen	23/789	325/19518	0.00685999	0.0413773	0.03071001	23
CC	GO:0019898	extrinsic component of membrane	21/789	288/19518	0.00687284	0.0413773	0.03071001	21
CC	GO:0031983	vesicle lumen	23/789	327/19518	0.00736487	0.04389162	0.03257612	23
CC	GO:0008305	integrin complex	5/789	31/19518	0.00755971	0.04460229	0.03310357	5
MF	GO:0140375	immune receptor activity	31/763	141/18369	1.55E-14	1.84E-11	1.46E-11	31
MF	GO:0023026	MHC class II protein complex binding	13/763	27/18369	1.16E-11	6.86E-09	5.43E-09	13
MF	GO:0023023	MHC protein complex binding	14/763	36/18369	6.56E-11	2.59E-08	2.05E-08	14
MF	GO:0032395	MHC class II receptor activity	8/763	10/18369	3.57E-10	1.06E-07	8.37E-08	8
MF	GO:0005216	monoatomic ion channel activity	47/763	432/18369	1.69E-09	4.00E-07	3.16E-07	47
MF	GO:0030594	neurotransmitter receptor activity	20/763	98/18369	2.94E-09	5.81E-07	4.59E-07	20
MF	GO:0015267	channel activity	49/763	478/18369	5.51E-09	7.31E-07	5.78E-07	49

MF	GO:0022803	passive transmembrane transporter activity	49/763	479/18369	5.90E-09	7.31E-07	5.78E-07	49
MF	GO:0005201	extracellular matrix structural constituent	26/763	167/18369	6.01E-09	7.31E-07	5.78E-07	26
MF	GO:0022836	gated channel activity	37/763	308/18369	6.79E-09	7.31E-07	5.78E-07	37
MF	GO:0022839	monoatomic ion gated channel activity	37/763	308/18369	6.79E-09	7.31E-07	5.78E-07	37
MF	GO:0015276	ligand-gated monoatomic ion channel activity	23/763	140/18369	1.59E-08	1.45E-06	1.15E-06	23
MF	GO:0022834	ligand-gated channel activity	23/763	140/18369	1.59E-08	1.45E-06	1.15E-06	23
MF	GO:0046873	metal ion transmembrane transporter activity	44/763	435/18369	4.97E-08	4.21E-06	3.33E-06	44
MF	GO:0008528	G protein-coupled peptide receptor activity	21/763	142/18369	4.19E-07	3.18E-05	2.52E-05	21
MF	GO:0099095	ligand-gated monoatomic anion channel activity	8/763	19/18369	4.30E-07	3.18E-05	2.52E-05	8
MF	GO:0001653	peptide receptor activity	21/763	148/18369	8.45E-07	5.89E-05	4.66E-05	21
MF	GO:0005237	inhibitory extracellular ligand-gated monoatomic ion channel activity	7/763	15/18369	9.97E-07	6.22E-05	4.92E-05	7
MF	GO:0001228	DNA-binding transcription activator activity, RNA polymerase II-specific	43/763	468/18369	9.98E-07	6.22E-05	4.92E-05	43
MF	GO:0001216	DNA-binding transcription activator activity	43/763	472/18369	1.25E-06	7.40E-05	5.86E-05	43
MF	GO:0022824	transmitter-gated monoatomic ion channel activity	12/763	56/18369	2.51E-06	0.00013507	0.00010688	12
MF	GO:0022835	transmitter-gated channel activity	12/763	56/18369	2.51E-06	0.00013507	0.00010688	12
MF	GO:0005230	extracellular ligand-gated monoatomic ion channel activity	13/763	67/18369	3.15E-06	0.00016217	0.00012831	13
MF	GO:0042277	peptide binding	32/763	317/18369	3.49E-06	0.0001722	0.00013626	32
MF	GO:0030020	extracellular matrix structural constituent conferring tensile strength	10/763	41/18369	5.01E-06	0.0002374	0.00018784	10
MF	GO:0005261	monoatomic cation channel activity	32/763	324/18369	5.53E-06	0.00025164	0.00019911	32
MF	GO:0022851	GABA-gated chloride ion channel activity	6/763	13/18369	6.73E-06	0.00028542	0.00022584	6
MF	GO:0004890	GABA-A receptor activity	7/763	19/18369	6.75E-06	0.00028542	0.00022584	7

MF	GO:0015079	potassium ion transmembrane transporter activity	20/763	157/18369	8.25E-06	0.00033686	0.00026654	20
MF	GO:0005254	chloride channel activity	13/763	74/18369	9.94E-06	0.00039232	0.00031042	13
MF	GO:0048248	CXCR3 chemokine receptor binding	4/763	5/18369	1.43E-05	0.00054555	0.00043167	4
MF	GO:0016917	GABA receptor activity	7/763	22/18369	2.05E-05	0.00075781	0.00059962	7
MF	GO:0030246	carbohydrate binding	27/763	273/18369	2.87E-05	0.00102875	0.000814	27
MF	GO:0015108	chloride transmembrane transporter activity	15/763	107/18369	3.54E-05	0.00123296	0.00097558	15
MF	GO:0005539	glycosaminoglycan binding	24/763	232/18369	3.83E-05	0.00126342	0.00099968	24
MF	GO:0016702	oxidoreductase activity, acting on single donors with incorporation of molecular oxygen, incorporation of two atoms of oxygen	7/763	24/18369	3.87E-05	0.00126342	0.00099968	7
MF	GO:0005244	voltage-gated monoatomic ion channel activity	21/763	189/18369	4.05E-05	0.00126342	0.00099968	21
MF	GO:0022832	voltage-gated channel activity	21/763	189/18369	4.05E-05	0.00126342	0.00099968	21
MF	GO:0016701	oxidoreductase activity, acting on single donors with incorporation of molecular oxygen	7/763	25/18369	5.18E-05	0.00157156	0.0012435	7
MF	GO:0005253	monoatomic anion channel activity	13/763	87/18369	5.92E-05	0.0017513	0.00138572	13
MF	GO:0033218	amide binding	34/763	400/18369	6.60E-05	0.00190583	0.00150799	34
MF	GO:0022843	voltage-gated monoatomic cation channel activity	17/763	140/18369	7.14E-05	0.0019659	0.00155552	17
MF	GO:0140788	L-glutamate uniporter activity	3/763	3/18369	7.14E-05	0.0019659	0.00155552	3
MF	GO:0004857	enzyme inhibitor activity	31/763	359/18369	0.00010267	0.00276265	0.00218595	31
MF	GO:0035325	Toll-like receptor binding	5/763	13/18369	0.00011888	0.00312797	0.00247501	5
MF	GO:1902936	phosphatidylinositol bisphosphate binding	14/763	107/18369	0.00013684	0.00352211	0.00278687	14
MF	GO:0005267	potassium channel activity	15/763	121/18369	0.00014813	0.00373162	0.00295265	15
MF	GO:0099094	ligand-gated monoatomic cation channel activity	14/763	108/18369	0.00015133	0.00373271	0.00295351	14
MF	GO:1901981	phosphatidylinositol phosphate binding	19/763	179/18369	0.00017064	0.00412327	0.00326254	19
MF	GO:0008509	monoatomic anion transmembrane transporter activity	15/763	123/18369	0.00017816	0.00421884	0.00333816	15

MF	GO:0030414	peptidase inhibitor activity	19/763	180/18369	0.00018353	0.00426088	0.00337143	19
MF	GO:0004993	G protein-coupled serotonin receptor activity	6/763	22/18369	0.00021217	0.00473979	0.00375037	6
MF	GO:0019956	chemokine binding	6/763	22/18369	0.00021217	0.00473979	0.00375037	6
MF	GO:0015103	inorganic anion transmembrane transporter activity	16/763	141/18369	0.00025527	0.0055712	0.00440822	16
MF	GO:0032393	MHC class I receptor activity	5/763	15/18369	0.0002588	0.0055712	0.00440822	5
MF	GO:0005249	voltage-gated potassium channel activity	12/763	88/18369	0.00027384	0.00564898	0.00446977	12
MF	GO:0022810	membrane potential driven uniporter activity	3/763	4/18369	0.00027672	0.00564898	0.00446977	3
MF	GO:0035663	Toll-like receptor 2 binding	3/763	4/18369	0.00027672	0.00564898	0.00446977	3
MF	GO:0019865	immunoglobulin binding	6/763	24/18369	0.00035649	0.00715405	0.00566066	6
MF	GO:0004896	cytokine receptor activity	12/763	92/18369	0.00041595	0.00820801	0.00649461	12
MF	GO:0001637	G protein-coupled chemoattractant receptor activity	6/763	25/18369	0.00045272	0.00864557	0.00684082	6
MF	GO:0004950	chemokine receptor activity	6/763	25/18369	0.00045272	0.00864557	0.00684082	6
MF	GO:0032396	inhibitory MHC class I receptor activity	4/763	10/18369	0.00050745	0.0093879	0.00742819	4
MF	GO:0071723	lipopeptide binding	4/763	10/18369	0.00050745	0.0093879	0.00742819	4
MF	GO:0005546	phosphatidylinositol-4,5-bisphosphate binding	11/763	82/18369	0.00056195	0.01023618	0.0080994	11
MF	GO:0061134	peptidase regulator activity	21/763	230/18369	0.0006293	0.01128924	0.00893263	21
MF	GO:0008201	heparin binding	17/763	168/18369	0.00064132	0.01133316	0.00896738	17
MF	GO:0015172	acidic amino acid transmembrane transporter activity	5/763	18/18369	0.00066557	0.01133906	0.00897205	5
MF	GO:0004982	N-formyl peptide receptor activity	3/763	5/18369	0.00067038	0.01133906	0.00897205	3
MF	GO:0019834	phospholipase A2 inhibitor activity	3/763	5/18369	0.00067038	0.01133906	0.00897205	3
MF	GO:0099589	serotonin receptor activity	6/763	27/18369	0.00070494	0.01175556	0.00930161	6
MF	GO:0015081	sodium ion transmembrane transporter activity	16/763	155/18369	0.00073712	0.01212156	0.0095912	16
MF	GO:0001540	amyloid-beta binding	11/763	85/18369	0.00076517	0.01217602	0.0096343	11
MF	GO:0008503	benzodiazepine receptor activity	4/763	11/18369	0.00077128	0.01217602	0.0096343	4
MF	GO:0019864	IgG binding	4/763	11/18369	0.00077128	0.01217602	0.0096343	4
MF	GO:0005543	phospholipid binding	35/763	475/18369	0.00078252	0.01219086	0.00964604	35

MF	GO:0004866	endopeptidase inhibitor activity	17/763	173/18369	0.00089347	0.01373858	0.01087068	17
MF	GO:0015293	symporter activity	15/763	144/18369	0.00097134	0.0145965	0.01154951	15
MF	GO:0001786	phosphatidylserine binding	9/763	62/18369	0.00098928	0.0145965	0.01154951	9
MF	GO:0048306	calcium-dependent protein binding	9/763	62/18369	0.00098928	0.0145965	0.01154951	9
MF	GO:0042605	peptide antigen binding	7/763	39/18369	0.00099858	0.0145965	0.01154951	7
MF	GO:0004859	phospholipase inhibitor activity	4/763	12/18369	0.00111905	0.01596337	0.01263105	4
MF	GO:0004875	complement receptor activity	4/763	12/18369	0.00111905	0.01596337	0.01263105	4
MF	GO:0005544	calcium-dependent phospholipid binding	8/763	52/18369	0.00127205	0.01792983	0.01418701	8
MF	GO:0005436	sodium:phosphate symporter activity	4/763	13/18369	0.00156357	0.02177967	0.0172332	4
MF	GO:0004051	arachidonate 5-lipoxygenase activity	2/763	2/18369	0.00172319	0.02345117	0.01855578	2
MF	GO:0071791	chemokine (C-C motif) ligand 5 binding	2/763	2/18369	0.00172319	0.02345117	0.01855578	2
MF	GO:0033691	sialic acid binding	5/763	22/18369	0.00178215	0.02397804	0.01897267	5
MF	GO:0001227	DNA-binding transcription repressor activity, RNA polymerase II-specific	24/763	303/18369	0.00196202	0.02588307	0.02048003	24
MF	GO:0005125	cytokine activity	20/763	235/18369	0.00196746	0.02588307	0.02048003	20
MF	GO:0061135	endopeptidase regulator activity	17/763	187/18369	0.00208838	0.02667219	0.02110442	17
MF	GO:0005313	L-glutamate transmembrane transporter activity	4/763	14/18369	0.00211756	0.02667219	0.02110442	4
MF	GO:0044548	S100 protein binding	4/763	14/18369	0.00211756	0.02667219	0.02110442	4
MF	GO:0071837	HMG box domain binding	4/763	14/18369	0.00211756	0.02667219	0.02110442	4
MF	GO:0015292	uniporter activity	3/763	7/18369	0.0022037	0.02746512	0.02173182	3
MF	GO:0015085	calcium ion transmembrane transporter activity	14/763	142/18369	0.00238243	0.02938336	0.02324964	14
MF	GO:0001217	DNA-binding transcription repressor activity	24/763	308/18369	0.0024311	0.02967444	0.02347996	24
MF	GO:0030247	polysaccharide binding	5/763	25/18369	0.00324362	0.03918817	0.03100771	5
MF	GO:0099104	potassium channel activator activity	3/763	8/18369	0.00341742	0.04087101	0.03233926	3
MF	GO:0003953	NAD+ nucleosidase activity	4/763	16/18369	0.00360342	0.04142183	0.0327751	4
MF	GO:0050135	NAD(P)+ nucleosidase activity	4/763	16/18369	0.00360342	0.04142183	0.0327751	4
MF	GO:0055102	lipase inhibitor activity	4/763	16/18369	0.00360342	0.04142183	0.0327751	4

MF	GO:0061809	NAD+ nucleotidase, cyclic ADP-ribose generating	4/763	16/18369	0.00360342	0.04142183	0.0327751	4
MF	GO:0001848	complement binding	5/763	26/18369	0.00388066	0.04417977	0.03495732	5
MF	GO:0005178	integrin binding	14/763	151/18369	0.00417405	0.04706739	0.03724216	14
MF	GO:1901681	sulfur compound binding	21/763	269/18369	0.00430268	0.04806016	0.03802769	21
MF	GO:0140828	metal cation:monoatomic cation antiporter activity	6/763	38/18369	0.00446253	0.04937979	0.03907185	6
MF	GO:0042165	neurotransmitter binding	4/763	17/18369	0.00455906	0.04953866	0.03919755	4
MF	GO:0005242	inward rectifier potassium channel activity	5/763	27/18369	0.00460241	0.04953866	0.03919755	5
MF	GO:0038187	pattern recognition receptor activity	5/763	27/18369	0.00460241	0.04953866	0.03919755	5

Table S13 GO terms based on DEGs between high and low SEPN1 expression groups in CGGA-693.

ONTOLOGY	ID	Description	GeneRatio	BgRatio	P-value	P-adjust	Q-value	Count
BP	GO:0045785	positive regulation of cell adhesion	194/4071	482/18614	2.51E-20	2.87E-16	2.33E-16	194
BP	GO:0002696	positive regulation of leukocyte activation	156/4071	377/18614	6.77E-18	3.86E-14	3.14E-14	156
BP	GO:0051251	positive regulation of lymphocyte activation	139/4071	327/18614	2.89E-17	1.10E-13	8.95E-14	139
BP	GO:0048568	embryonic organ development	177/4071	453/18614	4.03E-17	1.15E-13	9.34E-14	177
BP	GO:0050867	positive regulation of cell activation	159/4071	394/18614	5.26E-17	1.17E-13	9.53E-14	159
BP	GO:1903706	regulation of hemopoiesis	165/4071	415/18614	7.12E-17	1.17E-13	9.53E-14	165
BP	GO:1903039	positive regulation of leukocyte cell-cell adhesion	121/4071	273/18614	7.19E-17	1.17E-13	9.53E-14	121
BP	GO:0043123	positive regulation of I-kappaB kinase/NF-kappaB signaling	93/4071	192/18614	3.05E-16	4.29E-13	3.48E-13	93
BP	GO:1902105	regulation of leukocyte differentiation	134/4071	319/18614	3.46E-16	4.29E-13	3.48E-13	134
BP	GO:0007059	chromosome segregation	169/4071	435/18614	3.94E-16	4.29E-13	3.48E-13	169
BP	GO:0007159	leukocyte cell-cell adhesion	163/4071	415/18614	4.13E-16	4.29E-13	3.48E-13	163
BP	GO:0044772	mitotic cell cycle phase transition	178/4071	467/18614	5.61E-16	5.33E-13	4.33E-13	178
BP	GO:0050870	positive regulation of T cell activation	111/4071	249/18614	8.31E-16	7.29E-13	5.92E-13	111
BP	GO:0022409	positive regulation of cell-cell adhesion	133/4071	321/18614	1.66E-15	1.35E-12	1.10E-12	133
BP	GO:1901987	regulation of cell cycle phase transition	173/4071	457/18614	3.05E-15	2.32E-12	1.89E-12	173
BP	GO:0000070	mitotic sister chromatid segregation	93/4071	199/18614	5.17E-15	3.63E-12	2.95E-12	93
BP	GO:1903037	regulation of leukocyte cell-cell adhesion	149/4071	378/18614	5.41E-15	3.63E-12	2.95E-12	149
BP	GO:0000819	sister chromatid segregation	109/4071	250/18614	9.78E-15	6.20E-12	5.04E-12	109
BP	GO:1903131	mononuclear cell differentiation	176/4071	474/18614	1.41E-14	8.49E-12	6.90E-12	176
BP	GO:0007249	I-kappaB kinase/NF-kappaB signaling	122/4071	293/18614	1.59E-14	9.07E-12	7.37E-12	122
BP	GO:0050863	regulation of T cell activation	147/4071	377/18614	2.36E-14	1.28E-11	1.04E-11	147
BP	GO:0030217	T cell differentiation	123/4071	300/18614	4.77E-14	2.47E-11	2.01E-11	123
BP	GO:1902107	positive regulation of leukocyte differentiation	86/4071	184/18614	5.40E-14	2.53E-11	2.05E-11	86
BP	GO:1903708	positive regulation of hemopoiesis	86/4071	184/18614	5.40E-14	2.53E-11	2.05E-11	86

BP	GO:0048562	embryonic organ morphogenesis	121/4071	294/18614	5.54E-14	2.53E-11	2.05E-11	121
BP	GO:0140014	mitotic nuclear division	118/4071	286/18614	9.24E-14	4.06E-11	3.30E-11	118
BP	GO:0006260	DNA replication	115/4071	278/18614	1.54E-13	6.51E-11	5.29E-11	115
BP	GO:0001819	positive regulation of cytokine production	177/4071	489/18614	1.72E-13	7.01E-11	5.70E-11	177
BP	GO:0098813	nuclear chromosome segregation	133/4071	339/18614	2.38E-13	9.37E-11	7.62E-11	133
BP	GO:0043122	regulation of I-kappaB kinase/NF-kappaB signaling	108/4071	258/18614	3.37E-13	1.28E-10	1.04E-10	108
BP	GO:0022407	regulation of cell-cell adhesion	176/4071	491/18614	5.41E-13	1.99E-10	1.62E-10	176
BP	GO:1901990	regulation of mitotic cell cycle phase transition	137/4071	357/18614	7.37E-13	2.63E-10	2.14E-10	137
BP	GO:0002573	myeloid leukocyte differentiation	97/4071	227/18614	1.24E-12	4.29E-10	3.48E-10	97
BP	GO:0030098	lymphocyte differentiation	154/4071	422/18614	3.15E-12	1.06E-09	8.59E-10	154
BP	GO:0016032	viral process	155/4071	426/18614	3.50E-12	1.14E-09	9.28E-10	155
BP	GO:0002460	adaptive immune response based on somatic recombination of immune receptors built from immunoglobulin superfamily domains	121/4071	311/18614	5.53E-12	1.75E-09	1.42E-09	121
BP	GO:0000075	cell cycle checkpoint signaling	83/4071	188/18614	6.59E-12	2.03E-09	1.65E-09	83
BP	GO:0030099	myeloid cell differentiation	152/4071	421/18614	1.12E-11	3.37E-09	2.74E-09	152
BP	GO:1901988	negative regulation of cell cycle phase transition	109/4071	274/18614	1.25E-11	3.66E-09	2.97E-09	109
BP	GO:0010948	negative regulation of cell cycle process	121/4071	317/18614	2.43E-11	6.92E-09	5.62E-09	121
BP	GO:0045786	negative regulation of cell cycle	146/4071	404/18614	2.65E-11	7.36E-09	5.98E-09	146
BP	GO:0051983	regulation of chromosome segregation	62/4071	131/18614	8.98E-11	2.44E-08	1.98E-08	62
BP	GO:0032943	mononuclear cell proliferation	118/4071	313/18614	1.07E-10	2.83E-08	2.30E-08	118
BP	GO:0001503	ossification	151/4071	429/18614	1.14E-10	2.96E-08	2.41E-08	151
BP	GO:0002237	response to molecule of bacterial origin	133/4071	366/18614	1.32E-10	3.35E-08	2.72E-08	133
BP	GO:0071559	response to transforming growth factor beta	108/4071	280/18614	1.38E-10	3.42E-08	2.78E-08	108
BP	GO:1905818	regulation of chromosome separation	41/4071	73/18614	1.86E-10	4.53E-08	3.68E-08	41
BP	GO:0042060	wound healing	153/4071	439/18614	1.97E-10	4.59E-08	3.73E-08	153

BP	GO:0045621	positive regulation of lymphocyte differentiation	61/4071	130/18614	1.97E-10	4.59E-08	3.73E-08	61
BP	GO:1902850	microtubule cytoskeleton organization involved in mitosis	71/4071	161/18614	2.28E-10	5.13E-08	4.17E-08	71
BP	GO:0046651	lymphocyte proliferation	115/4071	306/18614	2.29E-10	5.13E-08	4.17E-08	115
BP	GO:0090068	positive regulation of cell cycle process	102/4071	262/18614	2.42E-10	5.30E-08	4.31E-08	102
BP	GO:0002443	leukocyte mediated immunity	142/4071	401/18614	2.54E-10	5.47E-08	4.45E-08	142
BP	GO:0032496	response to lipopolysaccharide	126/4071	345/18614	2.79E-10	5.89E-08	4.78E-08	126
BP	GO:0042098	T cell proliferation	87/4071	213/18614	2.97E-10	6.10E-08	4.95E-08	87
BP	GO:0019221	cytokine-mediated signaling pathway	167/4071	492/18614	2.99E-10	6.10E-08	4.95E-08	167
BP	GO:0030199	collagen fibril organization	37/4071	64/18614	4.65E-10	9.20E-08	7.48E-08	37
BP	GO:0006261	DNA-templated DNA replication	70/4071	160/18614	4.68E-10	9.20E-08	7.48E-08	70
BP	GO:0061448	connective tissue development	105/4071	275/18614	4.88E-10	9.44E-08	7.67E-08	105
BP	GO:0001701	in utero embryonic development	140/4071	398/18614	5.70E-10	1.08E-07	8.81E-08	140
BP	GO:1903557	positive regulation of tumor necrosis factor superfamily cytokine production	53/4071	109/18614	5.97E-10	1.12E-07	9.07E-08	53
BP	GO:0030198	extracellular matrix organization	116/4071	314/18614	6.41E-10	1.17E-07	9.50E-08	116
BP	GO:0048705	skeletal system morphogenesis	91/4071	229/18614	6.45E-10	1.17E-07	9.50E-08	91
BP	GO:0048704	embryonic skeletal system morphogenesis	48/4071	95/18614	7.35E-10	1.31E-07	1.07E-07	48
BP	GO:0043062	extracellular structure organization	116/4071	315/18614	7.99E-10	1.40E-07	1.14E-07	116
BP	GO:0071560	cellular response to transforming growth factor beta stimulus	104/4071	274/18614	8.68E-10	1.50E-07	1.22E-07	104
BP	GO:1901991	negative regulation of mitotic cell cycle phase transition	80/4071	194/18614	8.91E-10	1.52E-07	1.23E-07	80
BP	GO:0045582	positive regulation of T cell differentiation	55/4071	116/18614	9.36E-10	1.57E-07	1.28E-07	55
BP	GO:0051304	chromosome separation	42/4071	79/18614	1.12E-09	1.86E-07	1.51E-07	42
BP	GO:0045229	external encapsulating structure organization	116/4071	317/18614	1.23E-09	2.01E-07	1.63E-07	116

BP	GO:0032760	positive regulation of tumor necrosis factor production	51/4071	105/18614	1.31E-09	2.11E-07	1.71E-07	51
BP	GO:0048706	embryonic skeletal system development	59/4071	129/18614	1.35E-09	2.13E-07	1.73E-07	59
BP	GO:0010720	positive regulation of cell development	148/4071	432/18614	1.50E-09	2.29E-07	1.86E-07	148
BP	GO:0071214	cellular response to abiotic stimulus	122/4071	339/18614	1.51E-09	2.29E-07	1.86E-07	122
BP	GO:0104004	cellular response to environmental stimulus	122/4071	339/18614	1.51E-09	2.29E-07	1.86E-07	122
BP	GO:0050670	regulation of lymphocyte proliferation	93/4071	239/18614	1.52E-09	2.29E-07	1.86E-07	93
BP	GO:0030071	regulation of mitotic metaphase/anaphase transition	46/4071	91/18614	1.62E-09	2.40E-07	1.95E-07	46
BP	GO:0002253	activation of immune response	165/4071	495/18614	1.74E-09	2.54E-07	2.07E-07	165
BP	GO:0032944	regulation of mononuclear cell proliferation	94/4071	243/18614	1.78E-09	2.54E-07	2.07E-07	94
BP	GO:0045930	negative regulation of mitotic cell cycle	94/4071	243/18614	1.78E-09	2.54E-07	2.07E-07	94
BP	GO:0007265	Ras protein signal transduction	120/4071	333/18614	1.87E-09	2.64E-07	2.14E-07	120
BP	GO:0048732	gland development	150/4071	441/18614	2.05E-09	2.85E-07	2.31E-07	150
BP	GO:0070661	leukocyte proliferation	124/4071	348/18614	2.24E-09	3.07E-07	2.50E-07	124
BP	GO:0019058	viral life cycle	116/4071	320/18614	2.34E-09	3.17E-07	2.58E-07	116
BP	GO:0007091	metaphase/anaphase transition of mitotic cell cycle	47/4071	95/18614	2.69E-09	3.61E-07	2.93E-07	47
BP	GO:0009615	response to virus	140/4071	408/18614	3.70E-09	4.90E-07	3.98E-07	140
BP	GO:0002761	regulation of myeloid leukocyte differentiation	56/4071	123/18614	4.30E-09	5.65E-07	4.59E-07	56
BP	GO:0045580	regulation of T cell differentiation	74/4071	180/18614	4.41E-09	5.72E-07	4.65E-07	74
BP	GO:0071216	cellular response to biotic stimulus	99/4071	264/18614	4.59E-09	5.89E-07	4.79E-07	99
BP	GO:0060562	epithelial tube morphogenesis	119/4071	334/18614	4.71E-09	5.98E-07	4.86E-07	119
BP	GO:0007264	small GTPase mediated signal transduction	160/4071	483/18614	5.02E-09	6.29E-07	5.11E-07	160
BP	GO:0000280	nuclear division	151/4071	450/18614	5.14E-09	6.37E-07	5.18E-07	151
BP	GO:0000082	G1/S transition of mitotic cell cycle	95/4071	251/18614	5.43E-09	6.61E-07	5.37E-07	95
BP	GO:0042770	signal transduction in response to DNA damage	75/4071	184/18614	5.45E-09	6.61E-07	5.37E-07	75
BP	GO:0007052	mitotic spindle organization	58/4071	130/18614	5.83E-09	7.00E-07	5.69E-07	58

BP	GO:1902099	regulation of metaphase/anaphase transition of cell cycle	46/4071	94/18614	6.17E-09	7.33E-07	5.96E-07	46
BP	GO:0048872	homeostasis of number of cells	110/4071	304/18614	6.75E-09	7.94E-07	6.45E-07	110
BP	GO:0010965	regulation of mitotic sister chromatid separation	33/4071	58/18614	7.05E-09	8.21E-07	6.67E-07	33
BP	GO:0007093	mitotic cell cycle checkpoint signaling	61/4071	140/18614	7.26E-09	8.37E-07	6.80E-07	61
BP	GO:0002757	immune response-activating signaling pathway	143/4071	423/18614	7.60E-09	8.67E-07	7.05E-07	143
BP	GO:0045619	regulation of lymphocyte differentiation	83/4071	212/18614	8.14E-09	9.20E-07	7.47E-07	83
BP	GO:0033046	negative regulation of sister chromatid segregation	29/4071	48/18614	8.75E-09	9.51E-07	7.73E-07	29
BP	GO:0033048	negative regulation of mitotic sister chromatid segregation	29/4071	48/18614	8.75E-09	9.51E-07	7.73E-07	29
BP	GO:0045841	negative regulation of mitotic metaphase/anaphase transition	29/4071	48/18614	8.75E-09	9.51E-07	7.73E-07	29
BP	GO:2000816	negative regulation of mitotic sister chromatid separation	29/4071	48/18614	8.75E-09	9.51E-07	7.73E-07	29
BP	GO:0044784	metaphase/anaphase transition of cell cycle	47/4071	98/18614	9.64E-09	1.04E-06	8.43E-07	47
BP	GO:0007369	gastrulation	78/4071	196/18614	9.73E-09	1.04E-06	8.43E-07	78
BP	GO:0072001	renal system development	114/4071	320/18614	9.94E-09	1.05E-06	8.53E-07	114
BP	GO:0001822	kidney development	111/4071	310/18614	1.13E-08	1.17E-06	9.54E-07	111
BP	GO:0070663	regulation of leukocyte proliferation	99/4071	268/18614	1.13E-08	1.17E-06	9.54E-07	99
BP	GO:0045637	regulation of myeloid cell differentiation	83/4071	214/18614	1.35E-08	1.39E-06	1.13E-06	83
BP	GO:0007088	regulation of mitotic nuclear division	53/4071	117/18614	1.37E-08	1.40E-06	1.14E-06	53
BP	GO:0034504	protein localization to nucleus	109/4071	304/18614	1.40E-08	1.41E-06	1.15E-06	109
BP	GO:0048285	organelle fission	162/4071	497/18614	1.42E-08	1.42E-06	1.16E-06	162
BP	GO:0006275	regulation of DNA replication	59/4071	136/18614	1.55E-08	1.53E-06	1.25E-06	59
BP	GO:0031589	cell-substrate adhesion	124/4071	359/18614	1.85E-08	1.82E-06	1.48E-06	124
BP	GO:0002367	cytokine production involved in immune response	54/4071	121/18614	1.91E-08	1.86E-06	1.51E-06	54

BP	GO:0044843	cell cycle G1/S phase transition	102/4071	281/18614	1.96E-08	1.89E-06	1.54E-06	102
BP	GO:0002702	positive regulation of production of molecular mediator of immune response	58/4071	134/18614	2.25E-08	2.16E-06	1.75E-06	58
BP	GO:0002274	myeloid leukocyte activation	90/4071	240/18614	2.27E-08	2.16E-06	1.75E-06	90
BP	GO:1900182	positive regulation of protein localization to nucleus	42/4071	86/18614	3.00E-08	2.83E-06	2.30E-06	42
BP	GO:0051098	regulation of binding	126/4071	369/18614	3.05E-08	2.85E-06	2.32E-06	126
BP	GO:0002764	immune response-regulating signaling pathway	148/4071	450/18614	3.16E-08	2.89E-06	2.35E-06	148
BP	GO:0051985	negative regulation of chromosome segregation	29/4071	50/18614	3.19E-08	2.89E-06	2.35E-06	29
BP	GO:1902100	negative regulation of metaphase/anaphase transition of cell cycle	29/4071	50/18614	3.19E-08	2.89E-06	2.35E-06	29
BP	GO:1905819	negative regulation of chromosome separation	29/4071	50/18614	3.19E-08	2.89E-06	2.35E-06	29
BP	GO:0007179	transforming growth factor beta receptor signaling pathway	83/4071	218/18614	3.60E-08	3.24E-06	2.63E-06	83
BP	GO:0051306	mitotic sister chromatid separation	33/4071	61/18614	3.82E-08	3.41E-06	2.77E-06	33
BP	GO:0002429	immune response-activating cell surface receptor signaling pathway	107/4071	302/18614	3.88E-08	3.43E-06	2.79E-06	107
BP	GO:0061138	morphogenesis of a branching epithelium	74/4071	188/18614	3.96E-08	3.48E-06	2.82E-06	74
BP	GO:0002699	positive regulation of immune effector process	97/4071	267/18614	4.12E-08	3.59E-06	2.91E-06	97
BP	GO:0050900	leukocyte migration	132/4071	393/18614	4.39E-08	3.80E-06	3.09E-06	132
BP	GO:0051607	defense response to virus	107/4071	303/18614	4.72E-08	4.05E-06	3.29E-06	107
BP	GO:0002449	lymphocyte mediated immunity	106/4071	300/18614	5.27E-08	4.49E-06	3.64E-06	106
BP	GO:0045787	positive regulation of cell cycle	121/4071	354/18614	5.34E-08	4.49E-06	3.64E-06	121
BP	GO:0002683	negative regulation of immune system process	157/4071	487/18614	5.35E-08	4.49E-06	3.64E-06	157
BP	GO:0071260	cellular response to mechanical stimulus	37/4071	73/18614	5.74E-08	4.74E-06	3.85E-06	37
BP	GO:0140546	defense response to symbiont	107/4071	304/18614	5.74E-08	4.74E-06	3.85E-06	107
BP	GO:0042129	regulation of T cell proliferation	72/4071	183/18614	6.13E-08	5.04E-06	4.09E-06	72

BP	GO:0048762	mesenchymal cell differentiation	93/4071	255/18614	6.30E-08	5.13E-06	4.17E-06	93
BP	GO:0071706	tumor necrosis factor superfamily cytokine production	74/4071	190/18614	6.62E-08	5.31E-06	4.31E-06	74
BP	GO:1903555	regulation of tumor necrosis factor superfamily cytokine production	74/4071	190/18614	6.62E-08	5.31E-06	4.31E-06	74
BP	GO:0007094	mitotic spindle assembly checkpoint signaling	27/4071	46/18614	6.74E-08	5.31E-06	4.31E-06	27
BP	GO:0071173	spindle assembly checkpoint signaling	27/4071	46/18614	6.74E-08	5.31E-06	4.31E-06	27
BP	GO:0071174	mitotic spindle checkpoint signaling	27/4071	46/18614	6.74E-08	5.31E-06	4.31E-06	27
BP	GO:0031349	positive regulation of defense response	144/4071	441/18614	7.95E-08	6.18E-06	5.02E-06	144
BP	GO:0072175	epithelial tube formation	58/4071	138/18614	7.97E-08	6.18E-06	5.02E-06	58
BP	GO:0002718	regulation of cytokine production involved in immune response	52/4071	119/18614	8.10E-08	6.25E-06	5.08E-06	52
BP	GO:0098727	maintenance of cell number	71/4071	181/18614	8.69E-08	6.66E-06	5.41E-06	71
BP	GO:0007051	spindle organization	76/4071	198/18614	9.02E-08	6.86E-06	5.58E-06	76
BP	GO:0033044	regulation of chromosome organization	91/4071	250/18614	9.63E-08	7.28E-06	5.91E-06	91
BP	GO:0033045	regulation of sister chromatid segregation	47/4071	104/18614	9.73E-08	7.30E-06	5.94E-06	47
BP	GO:0051784	negative regulation of nuclear division	33/4071	63/18614	1.07E-07	8.00E-06	6.50E-06	33
BP	GO:0002697	regulation of immune effector process	128/4071	384/18614	1.14E-07	8.45E-06	6.87E-06	128
BP	GO:0031577	spindle checkpoint signaling	27/4071	47/18614	1.25E-07	9.23E-06	7.50E-06	27
BP	GO:0042113	B cell activation	98/4071	276/18614	1.27E-07	9.25E-06	7.52E-06	98
BP	GO:0001763	morphogenesis of a branching structure	77/4071	203/18614	1.32E-07	9.57E-06	7.77E-06	77
BP	GO:0051216	cartilage development	77/4071	203/18614	1.32E-07	9.57E-06	7.77E-06	77
BP	GO:0002064	epithelial cell development	79/4071	210/18614	1.37E-07	9.85E-06	8.01E-06	79
BP	GO:0001704	formation of primary germ layer	54/4071	127/18614	1.39E-07	9.93E-06	8.07E-06	54
BP	GO:0090329	regulation of DNA-templated DNA replication	31/4071	58/18614	1.42E-07	1.01E-05	8.19E-06	31
BP	GO:0071496	cellular response to external stimulus	112/4071	327/18614	1.46E-07	1.03E-05	8.38E-06	112
BP	GO:0002520	immune system development	75/4071	197/18614	1.61E-07	1.13E-05	9.17E-06	75

BP	GO:1900180	regulation of protein localization to nucleus	56/4071	134/18614	1.67E-07	1.16E-05	9.42E-06	56
BP	GO:0019827	stem cell population maintenance	69/4071	177/18614	1.74E-07	1.20E-05	9.77E-06	69
BP	GO:0051225	spindle assembly	55/4071	131/18614	1.78E-07	1.22E-05	9.95E-06	55
BP	GO:0033047	regulation of mitotic sister chromatid segregation	29/4071	53/18614	1.83E-07	1.25E-05	1.02E-05	29
BP	GO:0035148	tube formation	62/4071	154/18614	1.89E-07	1.28E-05	1.04E-05	62
BP	GO:0009612	response to mechanical stimulus	80/4071	215/18614	1.96E-07	1.32E-05	1.08E-05	80
BP	GO:0002768	immune response-regulating cell surface receptor signaling pathway	112/4071	329/18614	2.09E-07	1.40E-05	1.14E-05	112
BP	GO:0032755	positive regulation of interleukin-6 production	45/4071	100/18614	2.11E-07	1.40E-05	1.14E-05	45
BP	GO:0045839	negative regulation of mitotic nuclear division	30/4071	56/18614	2.11E-07	1.40E-05	1.14E-05	30
BP	GO:0032946	positive regulation of mononuclear cell proliferation	60/4071	148/18614	2.22E-07	1.46E-05	1.19E-05	60
BP	GO:0032640	tumor necrosis factor production	71/4071	185/18614	2.37E-07	1.55E-05	1.26E-05	71
BP	GO:0032680	regulation of tumor necrosis factor production	71/4071	185/18614	2.37E-07	1.55E-05	1.26E-05	71
BP	GO:0050671	positive regulation of lymphocyte proliferation	59/4071	145/18614	2.40E-07	1.55E-05	1.26E-05	59
BP	GO:0051783	regulation of nuclear division	59/4071	145/18614	2.40E-07	1.55E-05	1.26E-05	59
BP	GO:2000027	regulation of animal organ morphogenesis	54/4071	129/18614	2.58E-07	1.65E-05	1.34E-05	54
BP	GO:0090596	sensory organ morphogenesis	95/4071	269/18614	2.61E-07	1.66E-05	1.35E-05	95
BP	GO:0001837	epithelial to mesenchymal transition	67/4071	172/18614	2.69E-07	1.71E-05	1.39E-05	67
BP	GO:0061082	myeloid leukocyte cytokine production	28/4071	51/18614	2.71E-07	1.71E-05	1.39E-05	28
BP	GO:0002700	regulation of production of molecular mediator of immune response	72/4071	189/18614	2.74E-07	1.72E-05	1.40E-05	72
BP	GO:0060485	mesenchyme development	109/4071	320/18614	2.93E-07	1.82E-05	1.48E-05	109
BP	GO:0002720	positive regulation of cytokine production involved in immune response	38/4071	80/18614	3.35E-07	2.08E-05	1.69E-05	38
BP	GO:0007492	endoderm development	39/4071	83/18614	3.37E-07	2.08E-05	1.69E-05	39
BP	GO:0001706	endoderm formation	30/4071	57/18614	3.52E-07	2.16E-05	1.75E-05	30

BP	GO:0021915	neural tube development	64/4071	163/18614	3.54E-07	2.16E-05	1.75E-05	64
BP	GO:0006909	phagocytosis	85/4071	236/18614	4.23E-07	2.56E-05	2.08E-05	85
BP	GO:0030316	osteoclast differentiation	45/4071	102/18614	4.24E-07	2.56E-05	2.08E-05	45
BP	GO:1903707	negative regulation of hemopoiesis	49/4071	115/18614	4.81E-07	2.86E-05	2.32E-05	49
BP	GO:0090307	mitotic spindle assembly	35/4071	72/18614	4.81E-07	2.86E-05	2.32E-05	35
BP	GO:0071103	DNA conformation change	41/4071	90/18614	4.81E-07	2.86E-05	2.32E-05	41
BP	GO:1902106	negative regulation of leukocyte differentiation	47/4071	109/18614	5.38E-07	3.18E-05	2.58E-05	47
BP	GO:0002244	hematopoietic progenitor cell differentiation	54/4071	132/18614	6.23E-07	3.66E-05	2.98E-05	54
BP	GO:0061029	eyelid development in camera-type eye	12/4071	14/18614	6.87E-07	4.02E-05	3.26E-05	12
BP	GO:0002366	leukocyte activation involved in immune response	101/4071	296/18614	7.17E-07	4.17E-05	3.39E-05	101
BP	GO:0051099	positive regulation of binding	67/4071	176/18614	7.23E-07	4.17E-05	3.39E-05	67
BP	GO:0001838	embryonic epithelial tube formation	52/4071	126/18614	7.23E-07	4.17E-05	3.39E-05	52
BP	GO:0051984	positive regulation of chromosome segregation	18/4071	27/18614	7.49E-07	4.29E-05	3.49E-05	18
BP	GO:2001233	regulation of apoptotic signaling pathway	124/4071	381/18614	7.54E-07	4.29E-05	3.49E-05	124
BP	GO:0070665	positive regulation of leukocyte proliferation	64/4071	166/18614	7.56E-07	4.29E-05	3.49E-05	64
BP	GO:0002263	cell activation involved in immune response	102/4071	300/18614	7.65E-07	4.32E-05	3.51E-05	102
BP	GO:0001649	osteoblast differentiation	88/4071	250/18614	8.32E-07	4.67E-05	3.80E-05	88
BP	GO:0010212	response to ionizing radiation	57/4071	143/18614	8.51E-07	4.76E-05	3.87E-05	57
BP	GO:0002819	regulation of adaptive immune response	75/4071	205/18614	9.94E-07	5.53E-05	4.50E-05	75
BP	GO:0035987	endodermal cell differentiation	26/4071	48/18614	1.02E-06	5.66E-05	4.60E-05	26
BP	GO:0045069	regulation of viral genome replication	39/4071	86/18614	1.05E-06	5.78E-05	4.69E-05	39
BP	GO:0071219	cellular response to molecule of bacterial origin	84/4071	237/18614	1.06E-06	5.82E-05	4.73E-05	84
BP	GO:0032392	DNA geometric change	38/4071	83/18614	1.07E-06	5.85E-05	4.75E-05	38
BP	GO:0032508	DNA duplex unwinding	36/4071	77/18614	1.10E-06	5.96E-05	4.84E-05	36
BP	GO:0002831	regulation of response to biotic stimulus	145/4071	463/18614	1.13E-06	6.11E-05	4.96E-05	145
BP	GO:0001841	neural tube formation	45/4071	105/18614	1.14E-06	6.15E-05	5.00E-05	45
BP	GO:0061081	positive regulation of myeloid leukocyte cytokine production	21/4071	35/18614	1.18E-06	6.32E-05	5.14E-05	21

		involved in immune response						
BP	GO:0045766	positive regulation of angiogenesis	69/4071	185/18614	1.19E-06	6.32E-05	5.14E-05	69
BP	GO:1904018	positive regulation of vasculature development	69/4071	185/18614	1.19E-06	6.32E-05	5.14E-05	69
BP	GO:0008608	attachment of spindle microtubules to kinetochore	24/4071	43/18614	1.29E-06	6.82E-05	5.54E-05	24
BP	GO:0007517	muscle organ development	115/4071	351/18614	1.30E-06	6.84E-05	5.56E-05	115
BP	GO:0019724	B cell mediated immunity	58/4071	148/18614	1.32E-06	6.91E-05	5.61E-05	58
BP	GO:0050792	regulation of viral process	63/4071	165/18614	1.36E-06	7.07E-05	5.75E-05	63
BP	GO:1902749	regulation of cell cycle G2/M phase transition	47/4071	112/18614	1.38E-06	7.16E-05	5.82E-05	47
BP	GO:2001251	negative regulation of chromosome organization	41/4071	93/18614	1.39E-06	7.19E-05	5.85E-05	41
BP	GO:0016064	immunoglobulin mediated immune response	57/4071	145/18614	1.44E-06	7.42E-05	6.03E-05	57
BP	GO:0050673	epithelial cell proliferation	151/4071	488/18614	1.49E-06	7.61E-05	6.18E-05	151
BP	GO:0033627	cell adhesion mediated by integrin	39/4071	87/18614	1.50E-06	7.64E-05	6.21E-05	39
BP	GO:0031570	DNA integrity checkpoint signaling	53/4071	132/18614	1.56E-06	7.93E-05	6.44E-05	53
BP	GO:0034612	response to tumor necrosis factor	89/4071	257/18614	1.59E-06	8.03E-05	6.53E-05	89
BP	GO:0048144	fibroblast proliferation	44/4071	103/18614	1.66E-06	8.37E-05	6.80E-05	44
BP	GO:0007162	negative regulation of cell adhesion	104/4071	312/18614	1.70E-06	8.52E-05	6.93E-05	104
BP	GO:0007178	transmembrane receptor protein serine/threonine kinase signaling pathway	127/4071	398/18614	1.78E-06	8.87E-05	7.21E-05	127
BP	GO:0002062	chondrocyte differentiation	47/4071	113/18614	1.87E-06	9.26E-05	7.52E-05	47
BP	GO:0002285	lymphocyte activation involved in immune response	75/4071	208/18614	1.88E-06	9.27E-05	7.54E-05	75
BP	GO:0010998	regulation of translational initiation by eIF2 alpha phosphorylation	10/4071	11/18614	2.19E-06	0.00010747	8.73E-05	10
BP	GO:0010718	positive regulation of epithelial to mesenchymal transition	29/4071	58/18614	2.19E-06	0.00010747	8.73E-05	29
BP	GO:0035329	hippo signaling	24/4071	44/18614	2.25E-06	0.00010974	8.92E-05	24
BP	GO:0048754	branching morphogenesis of an epithelial tube	60/4071	157/18614	2.31E-06	0.00011238	9.13E-05	60

BP	GO:0051054	positive regulation of DNA metabolic process	101/4071	303/18614	2.39E-06	0.00011544	9.38E-05	101
BP	GO:0002687	positive regulation of leukocyte migration	57/4071	147/18614	2.41E-06	0.00011593	9.42E-05	57
BP	GO:0002262	myeloid cell homeostasis	64/4071	171/18614	2.49E-06	0.00011942	9.70E-05	64
BP	GO:1901976	regulation of cell cycle checkpoint	25/4071	47/18614	2.57E-06	0.00012261	9.96E-05	25
BP	GO:0071222	cellular response to lipopolysaccharide	79/4071	224/18614	2.72E-06	0.00012933	0.00010509	79
BP	GO:0044839	cell cycle G2/M phase transition	58/4071	151/18614	2.81E-06	0.00013293	0.00010802	58
BP	GO:0043583	ear development	78/4071	221/18614	3.04E-06	0.00014317	0.00011634	78
BP	GO:0060071	Wnt signaling pathway, planar cell polarity pathway	27/4071	53/18614	3.08E-06	0.00014483	0.00011769	27
BP	GO:0045071	negative regulation of viral genome replication	28/4071	56/18614	3.27E-06	0.00015311	0.00012442	28
BP	GO:0002822	regulation of adaptive immune response based on somatic recombination of immune receptors built from immunoglobulin superfamily domains	69/4071	190/18614	3.57E-06	0.00016595	0.00013485	69
BP	GO:0016331	morphogenesis of embryonic epithelium	58/4071	152/18614	3.58E-06	0.00016595	0.00013485	58
BP	GO:0048863	stem cell differentiation	85/4071	247/18614	3.59E-06	0.00016595	0.00013485	85
BP	GO:0006986	response to unfolded protein	55/4071	142/18614	3.73E-06	0.00017178	0.00013959	55
BP	GO:0048483	autonomic nervous system development	24/4071	45/18614	3.82E-06	0.00017453	0.00014182	24
BP	GO:0048701	embryonic cranial skeleton morphogenesis	24/4071	45/18614	3.82E-06	0.00017453	0.00014182	24
BP	GO:0050851	antigen receptor-mediated signaling pathway	72/4071	201/18614	3.92E-06	0.00017822	0.00014482	72
BP	GO:0016055	Wnt signaling pathway	142/4071	461/18614	3.96E-06	0.0001794	0.00014578	142
BP	GO:0019079	viral genome replication	51/4071	129/18614	4.16E-06	0.00018758	0.00015242	51
BP	GO:0045165	cell fate commitment	94/4071	281/18614	4.45E-06	0.00019969	0.00016227	94
BP	GO:0045639	positive regulation of myeloid cell differentiation	43/4071	103/18614	4.49E-06	0.00020074	0.00016312	43
BP	GO:1903900	regulation of viral life cycle	55/4071	143/18614	4.79E-06	0.00021339	0.0001734	55
BP	GO:0097191	extrinsic apoptotic signaling pathway	79/4071	227/18614	4.84E-06	0.00021488	0.0001746	79
BP	GO:0000077	DNA damage checkpoint signaling	49/4071	123/18614	4.95E-06	0.00021898	0.00017794	49

BP	GO:0090175	regulation of establishment of planar polarity	28/4071	57/18614	5.10E-06	0.0002239	0.00018194	28
BP	GO:0198738	cell-cell signaling by wnt	142/4071	463/18614	5.11E-06	0.0002239	0.00018194	142
BP	GO:0014020	primary neural tube formation	41/4071	97/18614	5.12E-06	0.0002239	0.00018194	41
BP	GO:0062197	cellular response to chemical stress	112/4071	349/18614	5.29E-06	0.00023036	0.00018719	112
BP	GO:0032757	positive regulation of interleukin-8 production	30/4071	63/18614	5.31E-06	0.00023036	0.00018719	30
BP	GO:0060760	positive regulation of response to cytokine stimulus	31/4071	66/18614	5.33E-06	0.00023036	0.00018719	31
BP	GO:0050864	regulation of B cell activation	51/4071	130/18614	5.40E-06	0.00023262	0.00018903	51
BP	GO:0071356	cellular response to tumor necrosis factor	81/4071	235/18614	5.62E-06	0.00024096	0.0001958	81
BP	GO:0009411	response to UV	58/4071	154/18614	5.76E-06	0.00024564	0.0001996	58
BP	GO:0001843	neural tube closure	39/4071	91/18614	5.78E-06	0.00024564	0.0001996	39
BP	GO:0003158	endothelium development	53/4071	137/18614	5.79E-06	0.00024564	0.0001996	53
BP	GO:0051170	import into nucleus	62/4071	168/18614	6.02E-06	0.0002545	0.0002068	62
BP	GO:0046631	alpha-beta T cell activation	64/4071	175/18614	6.07E-06	0.00025577	0.00020783	64
BP	GO:0045088	regulation of innate immune response	118/4071	373/18614	6.44E-06	0.0002703	0.00021965	118
BP	GO:0010389	regulation of G2/M transition of mitotic cell cycle	42/4071	101/18614	6.49E-06	0.00027134	0.00022048	42
BP	GO:0001667	ameboidal-type cell migration	150/4071	496/18614	6.59E-06	0.00027434	0.00022292	150
BP	GO:0050868	negative regulation of T cell activation	51/4071	131/18614	6.98E-06	0.00028977	0.00023546	51
BP	GO:0034501	protein localization to kinetochore	13/4071	18/18614	7.15E-06	0.00029461	0.00023939	13
BP	GO:1903083	protein localization to condensed chromosome	13/4071	18/18614	7.15E-06	0.00029461	0.00023939	13
BP	GO:0043618	regulation of transcription from RNA polymerase II promoter in response to stress	21/4071	38/18614	7.35E-06	0.00030168	0.00024514	21
BP	GO:0003170	heart valve development	33/4071	73/18614	7.53E-06	0.00030616	0.00024878	33
BP	GO:0035567	non-canonical Wnt signaling pathway	33/4071	73/18614	7.53E-06	0.00030616	0.00024878	33
BP	GO:2001234	negative regulation of apoptotic signaling pathway	80/4071	233/18614	7.54E-06	0.00030616	0.00024878	80
BP	GO:0032609	type II interferon production	47/4071	118/18614	7.70E-06	0.00031015	0.00025203	47

BP	GO:0032649	regulation of type II interferon production	47/4071	118/18614	7.70E-06	0.00031015	0.00025203	47
BP	GO:0032722	positive regulation of chemokine production	32/4071	70/18614	7.72E-06	0.00031015	0.00025203	32
BP	GO:0030225	macrophage differentiation	28/4071	58/18614	7.81E-06	0.00031254	0.00025396	28
BP	GO:1905517	macrophage migration	29/4071	61/18614	7.91E-06	0.00031419	0.00025531	29
BP	GO:0050000	chromosome localization	39/4071	92/18614	7.93E-06	0.00031419	0.00025531	39
BP	GO:0060606	tube closure	39/4071	92/18614	7.93E-06	0.00031419	0.00025531	39
BP	GO:0072331	signal transduction by p53 class mediator	63/4071	173/18614	8.38E-06	0.0003307	0.00026872	63
BP	GO:0140467	integrated stress response signaling	22/4071	41/18614	8.49E-06	0.00033389	0.00027132	22
BP	GO:0006606	protein import into nucleus	60/4071	163/18614	9.26E-06	0.00036303	0.000295	60
BP	GO:0043410	positive regulation of MAPK cascade	149/4071	495/18614	9.31E-06	0.00036363	0.00029548	149
BP	GO:1901992	positive regulation of mitotic cell cycle phase transition	41/4071	99/18614	9.39E-06	0.00036554	0.00029703	41
BP	GO:0002821	positive regulation of adaptive immune response	50/4071	129/18614	9.88E-06	0.00038327	0.00031144	50
BP	GO:0060326	cell chemotaxis	101/4071	312/18614	9.97E-06	0.00038572	0.00031343	101
BP	GO:1905820	positive regulation of chromosome separation	17/4071	28/18614	1.00E-05	0.00038607	0.00031371	17
BP	GO:0046632	alpha-beta T cell differentiation	47/4071	119/18614	1.00E-05	0.00038607	0.00031371	47
BP	GO:1905314	semi-lunar valve development	24/4071	47/18614	1.03E-05	0.00039354	0.00031979	24
BP	GO:0007596	blood coagulation	77/4071	224/18614	1.06E-05	0.00040263	0.00032717	77
BP	GO:0032732	positive regulation of interleukin-1 production	33/4071	74/18614	1.08E-05	0.00040821	0.00033171	33
BP	GO:0042063	gliogenesis	103/4071	320/18614	1.08E-05	0.00040821	0.00033171	103
BP	GO:0048146	positive regulation of fibroblast proliferation	26/4071	53/18614	1.14E-05	0.0004291	0.00034868	26
BP	GO:0045670	regulation of osteoclast differentiation	31/4071	68/18614	1.14E-05	0.00043021	0.00034958	31
BP	GO:0035966	response to topologically incorrect protein	60/4071	164/18614	1.15E-05	0.00043152	0.00035065	60
BP	GO:0006913	nucleocytoplasmic transport	105/4071	328/18614	1.16E-05	0.00043152	0.00035065	105
BP	GO:0051169	nuclear transport	105/4071	328/18614	1.16E-05	0.00043152	0.00035065	105
BP	GO:0031663	lipopolysaccharide-mediated signaling pathway	30/4071	65/18614	1.16E-05	0.00043279	0.00035167	30
BP	GO:0001961	positive regulation of cytokine-mediated signaling pathway	28/4071	59/18614	1.18E-05	0.00043465	0.00035319	28

BP	GO:0002763	positive regulation of myeloid leukocyte differentiation	28/4071	59/18614	1.18E-05	0.00043465	0.00035319	28
BP	GO:0048145	regulation of fibroblast proliferation	37/4071	87/18614	1.23E-05	0.00045203	0.00036731	37
BP	GO:0034446	substrate adhesion-dependent cell spreading	41/4071	100/18614	1.26E-05	0.00046133	0.00037487	41
BP	GO:0042307	positive regulation of protein import into nucleus	21/4071	39/18614	1.26E-05	0.00046255	0.00037586	21
BP	GO:0051250	negative regulation of lymphocyte activation	61/4071	168/18614	1.28E-05	0.00046785	0.00038016	61
BP	GO:0051303	establishment of chromosome localization	36/4071	84/18614	1.30E-05	0.00047362	0.00038485	36
BP	GO:1902751	positive regulation of cell cycle G2/M phase transition	18/4071	31/18614	1.32E-05	0.00047739	0.00038792	18
BP	GO:0060541	respiratory system development	73/4071	211/18614	1.37E-05	0.00049585	0.00040292	73
BP	GO:2000045	regulation of G1/S transition of mitotic cell cycle	66/4071	186/18614	1.38E-05	0.0004977	0.00040442	66
BP	GO:0046822	regulation of nucleocytoplasmic transport	43/4071	107/18614	1.42E-05	0.00050948	0.000414	43
BP	GO:0001738	morphogenesis of a polarized epithelium	39/4071	94/18614	1.46E-05	0.00052183	0.00042403	39
BP	GO:0070371	ERK1 and ERK2 cascade	108/4071	341/18614	1.47E-05	0.00052558	0.00042708	108
BP	GO:0017015	regulation of transforming growth factor beta receptor signaling pathway	55/4071	148/18614	1.56E-05	0.0005524	0.00044887	55
BP	GO:0046634	regulation of alpha-beta T cell activation	45/4071	114/18614	1.56E-05	0.0005524	0.00044887	45
BP	GO:0045765	regulation of angiogenesis	110/4071	349/18614	1.56E-05	0.0005524	0.00044887	110
BP	GO:0051310	metaphase plate congression	32/4071	72/18614	1.58E-05	0.00055644	0.00045216	32
BP	GO:1904888	cranial skeletal system development	32/4071	72/18614	1.58E-05	0.00055644	0.00045216	32
BP	GO:0007599	hemostasis	78/4071	230/18614	1.61E-05	0.00056417	0.00045844	78
BP	GO:0097529	myeloid leukocyte migration	81/4071	241/18614	1.62E-05	0.00056683	0.00046059	81
BP	GO:0021675	nerve development	37/4071	88/18614	1.68E-05	0.00058301	0.00047375	37
BP	GO:1901989	positive regulation of cell cycle phase transition	47/4071	121/18614	1.69E-05	0.00058441	0.00047488	47
BP	GO:0001885	endothelial cell development	29/4071	63/18614	1.73E-05	0.00059533	0.00048376	29

BP	GO:0032731	positive regulation of interleukin-1 beta production	29/4071	63/18614	1.73E-05	0.00059533	0.00048376	29
BP	GO:0003179	heart valve morphogenesis	28/4071	60/18614	1.75E-05	0.00060063	0.00048806	28
BP	GO:0007080	mitotic metaphase plate congression	27/4071	57/18614	1.75E-05	0.00060063	0.00048806	27
BP	GO:0010721	negative regulation of cell development	93/4071	286/18614	1.80E-05	0.00061446	0.0004993	93
BP	GO:0002685	regulation of leukocyte migration	77/4071	227/18614	1.80E-05	0.00061446	0.0004993	77
BP	GO:0002695	negative regulation of leukocyte activation	70/4071	202/18614	1.92E-05	0.00064868	0.00052711	70
BP	GO:0060759	regulation of response to cytokine stimulus	63/4071	177/18614	1.92E-05	0.00064868	0.00052711	63
BP	GO:0010592	positive regulation of lamellipodium assembly	17/4071	29/18614	1.93E-05	0.00064868	0.00052711	17
BP	GO:0034508	centromere complex assembly	17/4071	29/18614	1.93E-05	0.00064868	0.00052711	17
BP	GO:0009101	glycoprotein biosynthetic process	103/4071	324/18614	1.93E-05	0.00064868	0.00052711	103
BP	GO:0043161	proteasome-mediated ubiquitin-dependent protein catabolic process	133/4071	439/18614	1.95E-05	0.00065356	0.00053107	133
BP	GO:2000736	regulation of stem cell differentiation	34/4071	79/18614	2.02E-05	0.00067245	0.00054642	34
BP	GO:0044403	biological process involved in symbiotic interaction	100/4071	313/18614	2.02E-05	0.00067285	0.00054675	100
BP	GO:0042102	positive regulation of T cell proliferation	42/4071	105/18614	2.03E-05	0.00067399	0.00054767	42
BP	GO:0043367	CD4-positive, alpha-beta T cell differentiation	37/4071	89/18614	2.27E-05	0.0007472	0.00060716	37
BP	GO:0140694	non-membrane-bounded organelle assembly	120/4071	390/18614	2.27E-05	0.0007472	0.00060716	120
BP	GO:0045931	positive regulation of mitotic cell cycle	49/4071	129/18614	2.27E-05	0.0007472	0.00060716	49
BP	GO:0044786	cell cycle DNA replication	22/4071	43/18614	2.30E-05	0.00075419	0.00061284	22
BP	GO:0002224	toll-like receptor signaling pathway	31/4071	70/18614	2.33E-05	0.0007632	0.00062016	31
BP	GO:0000086	G2/M transition of mitotic cell cycle	51/4071	136/18614	2.35E-05	0.00076434	0.00062109	51
BP	GO:0007098	centrosome cycle	51/4071	136/18614	2.35E-05	0.00076434	0.00062109	51
BP	GO:0032602	chemokine production	40/4071	99/18614	2.40E-05	0.00077689	0.00063129	40
BP	GO:0050921	positive regulation of chemotaxis	53/4071	143/18614	2.40E-05	0.00077689	0.00063129	53
BP	GO:0050817	coagulation	77/4071	229/18614	2.55E-05	0.00082225	0.00066815	77

BP	GO:0002762	negative regulation of myeloid leukocyte differentiation	25/4071	52/18614	2.59E-05	0.00083179	0.0006759	25
BP	GO:0042306	regulation of protein import into nucleus	27/4071	58/18614	2.60E-05	0.00083179	0.0006759	27
BP	GO:0001570	vasculogenesis	35/4071	83/18614	2.60E-05	0.00083179	0.0006759	35
BP	GO:1903320	regulation of protein modification by small protein conjugation or removal	84/4071	255/18614	2.65E-05	0.00084369	0.00068557	84
BP	GO:0018212	peptidyl-tyrosine modification	115/4071	372/18614	2.65E-05	0.00084369	0.00068557	115
BP	GO:1904019	epithelial cell apoptotic process	52/4071	140/18614	2.67E-05	0.00084522	0.00068681	52
BP	GO:0010810	regulation of cell-substrate adhesion	75/4071	222/18614	2.69E-05	0.00085058	0.00069116	75
BP	GO:0002218	activation of innate immune response	78/4071	233/18614	2.70E-05	0.00085125	0.00069172	78
BP	GO:0002824	positive regulation of adaptive immune response based on somatic recombination of immune receptors built from immunoglobulin superfamily domains	47/4071	123/18614	2.77E-05	0.00086817	0.00070546	47
BP	GO:0048844	artery morphogenesis	34/4071	80/18614	2.77E-05	0.00086817	0.00070546	34
BP	GO:0051402	neuron apoptotic process	85/4071	259/18614	2.78E-05	0.00086817	0.00070546	85
BP	GO:0006301	postreplication repair	19/4071	35/18614	2.81E-05	0.00087442	0.00071054	19
BP	GO:0048048	embryonic eye morphogenesis	19/4071	35/18614	2.81E-05	0.00087442	0.00071054	19
BP	GO:0034644	cellular response to UV	38/4071	93/18614	2.82E-05	0.00087472	0.00071079	38
BP	GO:0001776	leukocyte homeostasis	41/4071	103/18614	2.90E-05	0.00089628	0.00072831	41
BP	GO:0050678	regulation of epithelial cell proliferation	127/4071	419/18614	2.91E-05	0.00089628	0.00072831	127
BP	GO:0051383	kinetochore organization	14/4071	22/18614	2.94E-05	0.00090098	0.00073212	14
BP	GO:0032729	positive regulation of type II interferon production	33/4071	77/18614	2.95E-05	0.00090098	0.00073212	33
BP	GO:0050853	B cell receptor signaling pathway	33/4071	77/18614	2.95E-05	0.00090098	0.00073212	33
BP	GO:0002833	positive regulation of response to biotic stimulus	103/4071	327/18614	2.95E-05	0.00090098	0.00073212	103
BP	GO:0001959	regulation of cytokine-mediated signaling pathway	59/4071	165/18614	2.98E-05	0.00090555	0.00073584	59
BP	GO:1903844	regulation of cellular response to transforming	55/4071	151/18614	3.02E-05	0.00091492	0.00074345	55

		growth factor beta stimulus						
BP	GO:0070997	neuron death	115/4071	373/18614	3.02E-05	0.00091492	0.00074345	115
BP	GO:0043558	regulation of translational initiation in response to stress	11/4071	15/18614	3.04E-05	0.00091654	0.00074477	11
BP	GO:0030326	embryonic limb morphogenesis	46/4071	120/18614	3.05E-05	0.00091654	0.00074477	46
BP	GO:0035113	embryonic appendage morphogenesis	46/4071	120/18614	3.05E-05	0.00091654	0.00074477	46
BP	GO:0001736	establishment of planar polarity	32/4071	74/18614	3.12E-05	0.00093089	0.00075642	32
BP	GO:0007164	establishment of tissue polarity	32/4071	74/18614	3.12E-05	0.00093089	0.00075642	32
BP	GO:0090092	regulation of transmembrane receptor protein serine/threonine kinase signaling pathway	96/4071	301/18614	3.16E-05	0.00093779	0.00076204	96
BP	GO:0097193	intrinsic apoptotic signaling pathway	96/4071	301/18614	3.16E-05	0.00093779	0.00076204	96
BP	GO:0003180	aortic valve morphogenesis	20/4071	38/18614	3.16E-05	0.00093779	0.00076204	20
BP	GO:0018108	peptidyl-tyrosine phosphorylation	114/4071	370/18614	3.37E-05	0.00099626	0.00080955	114
BP	GO:0009100	glycoprotein metabolic process	122/4071	401/18614	3.44E-05	0.00101313	0.00082326	122
BP	GO:1901342	regulation of vasculature development	110/4071	355/18614	3.53E-05	0.00103669	0.0008424	110
BP	GO:0030888	regulation of B cell proliferation	29/4071	65/18614	3.58E-05	0.00104988	0.00085311	29
BP	GO:0071478	cellular response to radiation	66/4071	191/18614	3.62E-05	0.00105865	0.00086024	66
BP	GO:0006888	endoplasmic reticulum to Golgi vesicle-mediated transport	49/4071	131/18614	3.63E-05	0.0010607	0.00086191	49
BP	GO:0043620	regulation of DNA-templated transcription in response to stress	22/4071	44/18614	3.65E-05	0.00106345	0.00086414	22
BP	GO:0072006	nephron development	55/4071	152/18614	3.73E-05	0.00108426	0.00088105	55
BP	GO:0060348	bone development	78/4071	235/18614	3.77E-05	0.00109165	0.00088706	78
BP	GO:0030282	bone mineralization	46/4071	121/18614	3.89E-05	0.00112256	0.00091217	46
BP	GO:0043543	protein acylation	82/4071	250/18614	3.90E-05	0.00112256	0.00091217	82
BP	GO:0051256	mitotic spindle midzone assembly	9/4071	11/18614	4.04E-05	0.00116102	0.00094343	9
BP	GO:0060021	roof of mouth development	37/4071	91/18614	4.06E-05	0.00116474	0.00094645	37
BP	GO:0045342	MHC class II biosynthetic process	13/4071	20/18614	4.13E-05	0.0011811	0.00095975	13
BP	GO:0016050	vesicle organization	113/4071	368/18614	4.27E-05	0.00121846	0.0009901	113

BP	GO:0002065	columnar/cuboidal epithelial cell differentiation	45/4071	118/18614	4.30E-05	0.00122171	0.00099275	45
BP	GO:0002381	immunoglobulin production involved in immunoglobulin-mediated immune response	32/4071	75/18614	4.30E-05	0.00122171	0.00099275	32
BP	GO:0002456	T cell mediated immunity	47/4071	125/18614	4.46E-05	0.00126254	0.00102592	47
BP	GO:0050866	negative regulation of cell activation	75/4071	225/18614	4.48E-05	0.00126432	0.00102736	75
BP	GO:1902806	regulation of cell cycle G1/S phase transition	72/4071	214/18614	4.49E-05	0.00126432	0.00102736	72
BP	GO:0032642	regulation of chemokine production	39/4071	98/18614	4.53E-05	0.00126938	0.00103148	39
BP	GO:0044000	movement in host	64/4071	185/18614	4.53E-05	0.00126938	0.00103148	64
BP	GO:0044409	entry into host	57/4071	160/18614	4.54E-05	0.00126955	0.00103162	57
BP	GO:0006487	protein N-linked glycosylation	31/4071	72/18614	4.56E-05	0.00127018	0.00103213	31
BP	GO:0030330	DNA damage response, signal transduction by p53 class mediator	31/4071	72/18614	4.56E-05	0.00127018	0.00103213	31
BP	GO:0046718	viral entry into host cell	55/4071	153/18614	4.60E-05	0.00127794	0.00103843	55
BP	GO:0034341	response to type II interferon	51/4071	139/18614	4.62E-05	0.00128011	0.00104019	51
BP	GO:0050878	regulation of body fluid levels	111/4071	361/18614	4.67E-05	0.00128994	0.00104819	111
BP	GO:0045089	positive regulation of innate immune response	95/4071	300/18614	4.71E-05	0.00129762	0.00105443	95
BP	GO:0035710	CD4-positive, alpha-beta T cell activation	44/4071	115/18614	4.74E-05	0.00130343	0.00105914	44
BP	GO:0071900	regulation of protein serine/threonine kinase activity	113/4071	369/18614	4.85E-05	0.00133034	0.00108101	113
BP	GO:0048592	eye morphogenesis	56/4071	157/18614	5.07E-05	0.00138302	0.00112382	56
BP	GO:0046683	response to organophosphorus	48/4071	129/18614	5.07E-05	0.00138302	0.00112382	48
BP	GO:0061351	neural precursor cell proliferation	54/4071	150/18614	5.13E-05	0.00139756	0.00113563	54
BP	GO:0010971	positive regulation of G2/M transition of mitotic cell cycle	16/4071	28/18614	5.26E-05	0.00142852	0.00116079	16
BP	GO:0010639	negative regulation of organelle organization	111/4071	362/18614	5.31E-05	0.00143778	0.00116832	111
BP	GO:0002286	T cell activation involved in immune response	45/4071	119/18614	5.46E-05	0.0014708	0.00119515	45
BP	GO:0045446	endothelial cell differentiation	45/4071	119/18614	5.46E-05	0.0014708	0.00119515	45

BP	GO:0046824	positive regulation of nucleocytoplasmic transport	27/4071	60/18614	5.48E-05	0.0014708	0.00119515	27
BP	GO:2000630	positive regulation of miRNA metabolic process	27/4071	60/18614	5.48E-05	0.0014708	0.00119515	27
BP	GO:0006403	RNA localization	67/4071	197/18614	5.57E-05	0.00149214	0.00121249	67
BP	GO:0006022	aminoglycan metabolic process	47/4071	126/18614	5.62E-05	0.00149968	0.00121862	47
BP	GO:0006473	protein acetylation	70/4071	208/18614	5.63E-05	0.00149968	0.00121862	70
BP	GO:0050729	positive regulation of inflammatory response	55/4071	154/18614	5.65E-05	0.00149968	0.00121862	55
BP	GO:0009314	response to radiation	134/4071	452/18614	5.66E-05	0.00149968	0.00121862	134
BP	GO:0031295	T cell costimulation	22/4071	45/18614	5.68E-05	0.00149968	0.00121862	22
BP	GO:0062208	positive regulation of pattern recognition receptor signaling pathway	22/4071	45/18614	5.68E-05	0.00149968	0.00121862	22
BP	GO:0060840	artery development	42/4071	109/18614	5.76E-05	0.00151769	0.00123325	42
BP	GO:1902895	positive regulation of miRNA transcription	24/4071	51/18614	5.80E-05	0.00152549	0.00123959	24
BP	GO:0006310	DNA recombination	103/4071	332/18614	5.82E-05	0.0015274	0.00124114	103
BP	GO:0018394	peptidyl-lysine acetylation	60/4071	172/18614	5.87E-05	0.00153564	0.00124784	60
BP	GO:0042471	ear morphogenesis	46/4071	123/18614	6.23E-05	0.00162679	0.00132191	46
BP	GO:0030032	lamellipodium assembly	31/4071	73/18614	6.28E-05	0.00163328	0.00132718	31
BP	GO:0046637	regulation of alpha-beta T cell differentiation	31/4071	73/18614	6.28E-05	0.00163328	0.00132718	31
BP	GO:0022408	negative regulation of cell-cell adhesion	69/4071	205/18614	6.30E-05	0.00163393	0.0013277	69
BP	GO:1901653	cellular response to peptide	114/4071	375/18614	6.33E-05	0.00163652	0.00132981	114
BP	GO:0033077	T cell differentiation in thymus	35/4071	86/18614	6.34E-05	0.00163652	0.00132981	35
BP	GO:1903510	mucopolysaccharide metabolic process	38/4071	96/18614	6.47E-05	0.00166536	0.00135325	38
BP	GO:0003002	regionalization	126/4071	422/18614	6.58E-05	0.00169153	0.00137451	126
BP	GO:0006979	response to oxidative stress	129/4071	434/18614	6.78E-05	0.00173866	0.00141281	129
BP	GO:0050871	positive regulation of B cell activation	34/4071	83/18614	6.86E-05	0.00175553	0.00142652	34
BP	GO:0051101	regulation of DNA binding	45/4071	120/18614	6.90E-05	0.00176231	0.00143202	45
BP	GO:0043524	negative regulation of neuron apoptotic process	55/4071	155/18614	6.92E-05	0.00176231	0.00143202	55
BP	GO:1990823	response to leukemia inhibitory factor	40/4071	103/18614	6.97E-05	0.00177217	0.00144004	40
BP	GO:0032743	positive regulation of interleukin-2 production	18/4071	34/18614	7.10E-05	0.00179984	0.00146252	18

BP	GO:0007160	cell-matrix adhesion	78/4071	239/18614	7.16E-05	0.00181266	0.00147294	78
BP	GO:0010717	regulation of epithelial to mesenchymal transition	42/4071	110/18614	7.37E-05	0.00185958	0.00151106	42
BP	GO:2001020	regulation of response to DNA damage stimulus	97/4071	311/18614	7.54E-05	0.00189824	0.00154248	97
BP	GO:0045936	negative regulation of phosphate metabolic process	130/4071	439/18614	7.66E-05	0.00192433	0.00156368	130
BP	GO:0048525	negative regulation of viral process	36/4071	90/18614	7.71E-05	0.00193303	0.00157075	36
BP	GO:0010934	macrophage cytokine production	19/4071	37/18614	7.75E-05	0.00193435	0.00157182	19
BP	GO:0010935	regulation of macrophage cytokine production	19/4071	37/18614	7.75E-05	0.00193435	0.00157182	19
BP	GO:2000514	regulation of CD4-positive, alpha-beta T cell activation	32/4071	77/18614	7.98E-05	0.00198797	0.00161539	32
BP	GO:0048771	tissue remodeling	62/4071	181/18614	8.19E-05	0.00203559	0.00165409	62
BP	GO:0043370	regulation of CD4-positive, alpha-beta T cell differentiation	25/4071	55/18614	8.37E-05	0.00207492	0.00168605	25
BP	GO:2001236	regulation of extrinsic apoptotic signaling pathway	55/4071	156/18614	8.44E-05	0.0020888	0.00169732	55
BP	GO:0003176	aortic valve development	21/4071	43/18614	8.51E-05	0.00210068	0.00170698	21
BP	GO:0010563	negative regulation of phosphorus metabolic process	130/4071	440/18614	8.56E-05	0.00210942	0.00171408	130
BP	GO:0016574	histone ubiquitination	22/4071	46/18614	8.65E-05	0.00212615	0.00172767	22
BP	GO:0001889	liver development	51/4071	142/18614	8.76E-05	0.00214949	0.00174664	51
BP	GO:0051259	protein complex oligomerization	80/4071	248/18614	9.07E-05	0.00222047	0.00180432	80
BP	GO:0016573	histone acetylation	54/4071	153/18614	9.43E-05	0.0023042	0.00187236	54
BP	GO:0035107	appendage morphogenesis	52/4071	146/18614	9.63E-05	0.00234382	0.00190455	52
BP	GO:0035108	limb morphogenesis	52/4071	146/18614	9.63E-05	0.00234382	0.00190455	52
BP	GO:0034599	cellular response to oxidative stress	91/4071	290/18614	9.71E-05	0.00235202	0.00191122	91
BP	GO:0030595	leukocyte chemotaxis	78/4071	241/18614	9.76E-05	0.00235202	0.00191122	78
BP	GO:0034101	erythrocyte homeostasis	50/4071	139/18614	9.77E-05	0.00235202	0.00191122	50
BP	GO:0050684	regulation of mRNA processing	50/4071	139/18614	9.77E-05	0.00235202	0.00191122	50
BP	GO:1903322	positive regulation of protein modification by small protein conjugation or removal	50/4071	139/18614	9.77E-05	0.00235202	0.00191122	50
BP	GO:0050764	regulation of phagocytosis	39/4071	101/18614	9.87E-05	0.00237207	0.00192751	39

BP	GO:0045348	positive regulation of MHC class II biosynthetic process	10/4071	14/18614	0.00010289	0.00245595	0.00199567	10
BP	GO:0051231	spindle elongation	10/4071	14/18614	0.00010289	0.00245595	0.00199567	10
BP	GO:1902969	mitotic DNA replication	10/4071	14/18614	0.00010289	0.00245595	0.00199567	10
BP	GO:0030324	lung development	63/4071	186/18614	0.00010448	0.00248882	0.00202237	63
BP	GO:1903038	negative regulation of leukocyte cell-cell adhesion	53/4071	150/18614	0.00010539	0.0025051	0.0020356	53
BP	GO:0061196	fungiform papilla development	6/4071	6/18614	0.00010912	0.00258856	0.00210342	6
BP	GO:0008380	RNA splicing	136/4071	466/18614	0.00011025	0.00260993	0.00212079	136
BP	GO:0030323	respiratory tube development	64/4071	190/18614	0.00011103	0.0026186	0.00212783	64
BP	GO:0018393	internal peptidyl-lysine acetylation	56/4071	161/18614	0.00011108	0.0026186	0.00212783	56
BP	GO:0060907	positive regulation of macrophage cytokine production	14/4071	24/18614	0.0001149	0.00270318	0.00219656	14
BP	GO:0007229	integrin-mediated signaling pathway	42/4071	112/18614	0.00011857	0.00278367	0.00226196	42
BP	GO:0007389	pattern specification process	136/4071	467/18614	0.00012251	0.00287038	0.00233243	136
BP	GO:0009952	anterior/posterior pattern specification	69/4071	209/18614	0.000123	0.00287425	0.00233557	69
BP	GO:0033260	nuclear DNA replication	19/4071	38/18614	0.00012331	0.00287425	0.00233557	19
BP	GO:0016570	histone modification	138/4071	475/18614	0.00012343	0.00287425	0.00233557	138
BP	GO:0045346	regulation of MHC class II biosynthetic process	12/4071	19/18614	0.00012443	0.00289147	0.00234956	12
BP	GO:0030111	regulation of Wnt signaling pathway	103/4071	338/18614	0.00012589	0.00291951	0.00237234	103
BP	GO:1990830	cellular response to leukemia inhibitory factor	39/4071	102/18614	0.00012649	0.00292755	0.00237888	39
BP	GO:0048246	macrophage chemotaxis	20/4071	41/18614	0.00012753	0.00294559	0.00239354	20
BP	GO:0070372	regulation of ERK1 and ERK2 cascade	97/4071	315/18614	0.00012785	0.0029471	0.00239477	97
BP	GO:0031294	lymphocyte costimulation	22/4071	47/18614	0.00012917	0.00297137	0.00241449	22
BP	GO:0000022	mitotic spindle elongation	9/4071	12/18614	0.00012999	0.00298433	0.00242502	9
BP	GO:0001656	metanephros development	36/4071	92/18614	0.00013128	0.00300432	0.00244126	36
BP	GO:0042116	macrophage activation	41/4071	109/18614	0.00013139	0.00300432	0.00244126	41
BP	GO:0061614	miRNA transcription	29/4071	69/18614	0.00013364	0.00304958	0.00247804	29
BP	GO:0007266	Rho protein signal transduction	47/4071	130/18614	0.00013549	0.00308575	0.00250743	47
BP	GO:1901796	regulation of signal transduction by p53 class mediator	40/4071	106/18614	0.00014552	0.00330755	0.00268766	40

BP	GO:0051092	positive regulation of NF-kappaB transcription factor activity	55/4071	159/18614	0.00015006	0.00340386	0.00276592	55
BP	GO:0048593	camera-type eye morphogenesis	46/4071	127/18614	0.00015105	0.00341958	0.00277869	46
BP	GO:0032623	interleukin-2 production	27/4071	63/18614	0.00015204	0.00342697	0.0027847	27
BP	GO:0032663	regulation of interleukin-2 production	27/4071	63/18614	0.00015204	0.00342697	0.0027847	27
BP	GO:0033314	mitotic DNA replication checkpoint signaling	8/4071	10/18614	0.00015228	0.00342697	0.0027847	8
BP	GO:1905821	positive regulation of chromosome condensation	7/4071	8/18614	0.00015426	0.00346481	0.00281545	7
BP	GO:0001890	placenta development	53/4071	152/18614	0.00015556	0.00348027	0.00282801	53
BP	GO:0031023	microtubule organizing center organization	53/4071	152/18614	0.00015556	0.00348027	0.00282801	53
BP	GO:0021602	cranial nerve morphogenesis	16/4071	30/18614	0.0001598	0.00356638	0.00289798	16
BP	GO:0061008	hepaticobiliary system development	51/4071	145/18614	0.00016037	0.00356638	0.00289798	51
BP	GO:0021545	cranial nerve development	26/4071	60/18614	0.00016103	0.00356638	0.00289798	26
BP	GO:0032677	regulation of interleukin-8 production	39/4071	103/18614	0.00016108	0.00356638	0.00289798	39
BP	GO:0150115	cell-substrate junction organization	39/4071	103/18614	0.00016108	0.00356638	0.00289798	39
BP	GO:0006475	internal protein amino acid acetylation	56/4071	163/18614	0.00016128	0.00356638	0.00289798	56
BP	GO:0007405	neuroblast proliferation	30/4071	73/18614	0.00016761	0.00369205	0.0030001	30
BP	GO:0050766	positive regulation of phagocytosis	30/4071	73/18614	0.00016761	0.00369205	0.0030001	30
BP	GO:0030203	glycosaminoglycan metabolic process	43/4071	117/18614	0.00016802	0.00369386	0.00300157	43
BP	GO:0032069	regulation of nuclease activity	13/4071	22/18614	0.00016941	0.00370311	0.00300909	13
BP	GO:0043555	regulation of translation in response to stress	13/4071	22/18614	0.00016941	0.00370311	0.00300909	13
BP	GO:0048485	sympathetic nervous system development	13/4071	22/18614	0.00016941	0.00370311	0.00300909	13
BP	GO:0072332	intrinsic apoptotic signaling pathway by p53 class mediator	33/4071	83/18614	0.00017259	0.00376528	0.0030596	33
BP	GO:1903311	regulation of mRNA metabolic process	100/4071	329/18614	0.00017387	0.00378115	0.0030725	100
BP	GO:0002221	pattern recognition receptor signaling pathway	63/4071	189/18614	0.00017481	0.00378115	0.0030725	63
BP	GO:0032635	interleukin-6 production	60/4071	178/18614	0.00017486	0.00378115	0.0030725	60

BP	GO:0032675	regulation of interleukin-6 production	60/4071	178/18614	0.00017486	0.00378115	0.0030725	60
BP	GO:0002063	chondrocyte development	17/4071	33/18614	0.00017497	0.00378115	0.0030725	17
BP	GO:0003198	epithelial to mesenchymal transition involved in endocardial cushion formation	11/4071	17/18614	0.00017689	0.00380822	0.0030945	11
BP	GO:0033631	cell-cell adhesion mediated by integrin	11/4071	17/18614	0.00017689	0.00380822	0.0030945	11
BP	GO:0043434	response to peptide hormone	125/4071	427/18614	0.0001779	0.00380862	0.00309482	125
BP	GO:0030512	negative regulation of transforming growth factor beta receptor signaling pathway	38/4071	100/18614	0.00017819	0.00380862	0.00309482	38
BP	GO:0140888	interferon-mediated signaling pathway	38/4071	100/18614	0.00017819	0.00380862	0.00309482	38
BP	GO:0050920	regulation of chemotaxis	74/4071	230/18614	0.00017825	0.00380862	0.00309482	74
BP	GO:0051384	response to glucocorticoid	50/4071	142/18614	0.00017932	0.00382428	0.00310755	50
BP	GO:0048839	inner ear development	64/4071	193/18614	0.00018426	0.00391948	0.00318491	64
BP	GO:0090287	regulation of cellular response to growth factor stimulus	104/4071	345/18614	0.00018447	0.00391948	0.00318491	104
BP	GO:0001569	branching involved in blood vessel morphogenesis	18/4071	36/18614	0.00018535	0.00393097	0.00319425	18
BP	GO:0019882	antigen processing and presentation	42/4071	114/18614	0.0001869	0.00395656	0.00321504	42
BP	GO:2000142	regulation of DNA-templated transcription initiation	32/4071	80/18614	0.00018848	0.00398252	0.00323613	32
BP	GO:0030890	positive regulation of B cell proliferation	21/4071	45/18614	0.00019287	0.00406773	0.00330537	21
BP	GO:0010591	regulation of lamellipodium assembly	20/4071	42/18614	0.00019369	0.0040729	0.00330957	20
BP	GO:0042176	regulation of protein catabolic process	108/4071	361/18614	0.00019383	0.0040729	0.00330957	108
BP	GO:0072073	kidney epithelium development	51/4071	146/18614	0.00019474	0.00408449	0.00331899	51
BP	GO:0002709	regulation of T cell mediated immunity	37/4071	97/18614	0.00019698	0.00412386	0.00335098	37
BP	GO:0006302	double-strand break repair	93/4071	303/18614	0.00019762	0.00412983	0.00335583	93
BP	GO:0032637	interleukin-8 production	39/4071	104/18614	0.00020395	0.00425414	0.00345685	39
BP	GO:0002703	regulation of leukocyte mediated immunity	78/4071	246/18614	0.00020442	0.00425622	0.00345853	78
BP	GO:0010811	positive regulation of cell-substrate adhesion	45/4071	125/18614	0.00020801	0.00432303	0.00351282	45

BP	GO:2000679	positive regulation of transcription regulatory region DNA binding	14/4071	25/18614	0.00020864	0.0043284	0.00351719	14
BP	GO:0014074	response to purine-containing compound	50/4071	143/18614	0.00021789	0.00451205	0.00366642	50
BP	GO:1901214	regulation of neuron death	99/4071	327/18614	0.00021887	0.00452408	0.00367619	99
BP	GO:0002758	innate immune response-activating signaling pathway	68/4071	209/18614	0.00022207	0.004582	0.00372326	68
BP	GO:0030879	mammary gland development	48/4071	136/18614	0.00022419	0.00461572	0.00375066	48
BP	GO:0072594	establishment of protein localization to organelle	131/4071	453/18614	0.00022452	0.00461572	0.00375066	131
BP	GO:0003007	heart morphogenesis	82/4071	262/18614	0.00022931	0.00470581	0.00382387	82
BP	GO:0031396	regulation of protein ubiquitination	69/4071	213/18614	0.00023145	0.00474118	0.00385261	69
BP	GO:0048008	platelet-derived growth factor receptor signaling pathway	25/4071	58/18614	0.00023666	0.00483925	0.0039323	25
BP	GO:0097581	lamellipodium organization	35/4071	91/18614	0.00024014	0.00490162	0.00398297	35
BP	GO:0002440	production of molecular mediator of immune response	99/4071	328/18614	0.00024711	0.00503477	0.00409117	99
BP	GO:0043584	nose development	10/4071	15/18614	0.00024793	0.00503782	0.00409365	10
BP	GO:0051701	biological process involved in interaction with host	67/4071	206/18614	0.00024873	0.00503782	0.00409365	67
BP	GO:1900227	positive regulation of NLRP3 inflammasome complex assembly	12/4071	20/18614	0.00024911	0.00503782	0.00409365	12
BP	GO:2001185	regulation of CD8-positive, alpha-beta T cell activation	12/4071	20/18614	0.00024911	0.00503782	0.00409365	12
BP	GO:0050730	regulation of peptidyl-tyrosine phosphorylation	80/4071	255/18614	0.00024946	0.00503782	0.00409365	80
BP	GO:0018210	peptidyl-threonine modification	43/4071	119/18614	0.00025893	0.00521973	0.00424147	43
BP	GO:0048880	sensory system development	116/4071	395/18614	0.00026017	0.00523548	0.00425426	116
BP	GO:1902893	regulation of miRNA transcription	28/4071	68/18614	0.00026248	0.00524601	0.00426282	28
BP	GO:0046638	positive regulation of alpha-beta T cell differentiation	23/4071	52/18614	0.00026249	0.00524601	0.00426282	23
BP	GO:0001894	tissue homeostasis	82/4071	263/18614	0.00026279	0.00524601	0.00426282	82
BP	GO:0060249	anatomical structure homeostasis	82/4071	263/18614	0.00026279	0.00524601	0.00426282	82

BP	GO:0010390	histone monoubiquitination	16/4071	31/18614	0.00026345	0.00524601	0.00426282	16
BP	GO:0019886	antigen processing and presentation of exogenous peptide antigen via MHC class II	16/4071	31/18614	0.00026345	0.00524601	0.00426282	16
BP	GO:0033628	regulation of cell adhesion mediated by integrin	22/4071	49/18614	0.00027344	0.00542597	0.00440906	22
BP	GO:0061028	establishment of endothelial barrier	22/4071	49/18614	0.00027344	0.00542597	0.00440906	22
BP	GO:0031960	response to corticosteroid	56/4071	166/18614	0.00027568	0.00546103	0.00443754	56
BP	GO:0045638	negative regulation of myeloid cell differentiation	36/4071	95/18614	0.00027742	0.00548584	0.0044577	36
BP	GO:0001782	B cell homeostasis	17/4071	34/18614	0.00027896	0.00549736	0.00446706	17
BP	GO:0110110	positive regulation of animal organ morphogenesis	17/4071	34/18614	0.00027896	0.00549736	0.00446706	17
BP	GO:0030218	erythrocyte differentiation	46/4071	130/18614	0.00028028	0.00551373	0.00448037	46
BP	GO:0070482	response to oxygen levels	100/4071	333/18614	0.0002822	0.00554193	0.00450328	100
BP	GO:0002504	antigen processing and presentation of peptide or polysaccharide antigen via MHC class II	18/4071	37/18614	0.00028755	0.0056277	0.00457297	18
BP	GO:0006270	DNA replication initiation	18/4071	37/18614	0.00028755	0.0056277	0.00457297	18
BP	GO:1901532	regulation of hematopoietic progenitor cell differentiation	19/4071	40/18614	0.00029026	0.00567097	0.00460813	19
BP	GO:0071479	cellular response to ionizing radiation	30/4071	75/18614	0.00029533	0.00576015	0.0046806	30
BP	GO:0032481	positive regulation of type I interferon production	26/4071	62/18614	0.00030452	0.00592937	0.00481811	26
BP	GO:0051236	establishment of RNA localization	55/4071	163/18614	0.00030898	0.00600589	0.00488029	55
BP	GO:0032727	positive regulation of interferon-alpha production	13/4071	23/18614	0.00031173	0.00604897	0.00491529	13
BP	GO:0001654	eye development	113/4071	385/18614	0.00031745	0.00614955	0.00499702	113
BP	GO:0150063	visual system development	114/4071	389/18614	0.00031933	0.00617556	0.00501816	114
BP	GO:0046635	positive regulation of alpha-beta T cell activation	29/4071	72/18614	0.00032208	0.0062181	0.00505273	29
BP	GO:0031667	response to nutrient levels	136/4071	477/18614	0.00033559	0.00646813	0.0052559	136

BP	GO:0001892	embryonic placenta development	34/4071	89/18614	0.00033946	0.00652052	0.00529847	34
BP	GO:0002312	B cell activation involved in immune response	34/4071	89/18614	0.00033946	0.00652052	0.00529847	34
BP	GO:0032612	interleukin-1 production	46/4071	131/18614	0.00034157	0.00653116	0.00530711	46
BP	GO:0032652	regulation of interleukin-1 production	46/4071	131/18614	0.00034157	0.00653116	0.00530711	46
BP	GO:0048736	appendage development	60/4071	182/18614	0.0003423	0.00653116	0.00530711	60
BP	GO:0060173	limb development	60/4071	182/18614	0.0003423	0.00653116	0.00530711	60
BP	GO:0050657	nucleic acid transport	54/4071	160/18614	0.00034633	0.00658598	0.00535166	54
BP	GO:0050658	RNA transport	54/4071	160/18614	0.00034633	0.00658598	0.00535166	54
BP	GO:0018205	peptidyl-lysine modification	110/4071	374/18614	0.00034734	0.00659427	0.00535839	110
BP	GO:0042093	T-helper cell differentiation	28/4071	69/18614	0.00035047	0.00664256	0.00539763	28
BP	GO:0048534	hematopoietic or lymphoid organ development	36/4071	96/18614	0.00035151	0.00665137	0.00540479	36
BP	GO:0042100	B cell proliferation	38/4071	103/18614	0.00035783	0.00675973	0.00549285	38
BP	GO:0006929	substrate-dependent cell migration	14/4071	26/18614	0.00036131	0.00680296	0.00552797	14
BP	GO:1905523	positive regulation of macrophage migration	14/4071	26/18614	0.00036131	0.00680296	0.00552797	14
BP	GO:0000076	DNA replication checkpoint signaling	11/4071	18/18614	0.00036488	0.00682501	0.00554589	11
BP	GO:0007250	activation of NF-kappaB-inducing kinase activity	11/4071	18/18614	0.00036488	0.00682501	0.00554589	11
BP	GO:0051988	regulation of attachment of spindle microtubules to kinetochore	11/4071	18/18614	0.00036488	0.00682501	0.00554589	11
BP	GO:0090594	inflammatory response to wounding	11/4071	18/18614	0.00036488	0.00682501	0.00554589	11
BP	GO:0006457	protein folding	70/4071	220/18614	0.00037578	0.00701737	0.0057022	70
BP	GO:0032613	interleukin-10 production	27/4071	66/18614	0.00038039	0.00708038	0.0057534	27
BP	GO:0032653	regulation of interleukin-10 production	27/4071	66/18614	0.00038039	0.00708038	0.0057534	27
BP	GO:0010976	positive regulation of neuron projection development	53/4071	157/18614	0.00038822	0.0072032	0.0058532	53
BP	GO:0010862	positive regulation of pathway-restricted SMAD protein phosphorylation	22/4071	50/18614	0.00038825	0.0072032	0.0058532	22
BP	GO:0042326	negative regulation of phosphorylation	112/4071	383/18614	0.00039173	0.00722751	0.00587295	112
BP	GO:0031398	positive regulation of protein ubiquitination	43/4071	121/18614	0.00039173	0.00722751	0.00587295	43
BP	GO:0072089	stem cell proliferation	43/4071	121/18614	0.00039173	0.00722751	0.00587295	43
BP	GO:0001666	response to hypoxia	89/4071	293/18614	0.0003921	0.00722751	0.00587295	89

BP	GO:0010575	positive regulation of vascular endothelial growth factor production	15/4071	29/18614	0.00039725	0.00728719	0.00592145	15
BP	GO:0032607	interferon-alpha production	15/4071	29/18614	0.00039725	0.00728719	0.00592145	15
BP	GO:0032647	regulation of interferon-alpha production	15/4071	29/18614	0.00039725	0.00728719	0.00592145	15
BP	GO:0006984	ER-nucleus signaling pathway	21/4071	47/18614	0.0004059	0.00743385	0.00604063	21
BP	GO:0006298	mismatch repair	16/4071	32/18614	0.00042045	0.00764764	0.00621434	16
BP	GO:0045671	negative regulation of osteoclast differentiation	16/4071	32/18614	0.00042045	0.00764764	0.00621434	16
BP	GO:0060055	angiogenesis involved in wound healing	16/4071	32/18614	0.00042045	0.00764764	0.00621434	16
BP	GO:0042771	intrinsic apoptotic signaling pathway in response to DNA damage by p53 class mediator	20/4071	44/18614	0.00042049	0.00764764	0.00621434	20
BP	GO:0060070	canonical Wnt signaling pathway	94/4071	313/18614	0.00042092	0.00764764	0.00621434	94
BP	GO:0071674	mononuclear cell migration	65/4071	202/18614	0.0004239	0.00768952	0.00624838	65
BP	GO:0034976	response to endoplasmic reticulum stress	80/4071	259/18614	0.00042783	0.00774853	0.00629633	80
BP	GO:0001893	maternal placenta development	17/4071	35/18614	0.00043253	0.00780881	0.00634531	17
BP	GO:0071425	hematopoietic stem cell proliferation	17/4071	35/18614	0.00043253	0.00780881	0.00634531	17
BP	GO:0015931	nucleobase-containing compound transport	70/4071	221/18614	0.00043408	0.0078229	0.00635676	70
BP	GO:0032728	positive regulation of interferon-beta production	18/4071	38/18614	0.00043537	0.0078229	0.00635676	18
BP	GO:0140894	endolysosomal toll-like receptor signaling pathway	18/4071	38/18614	0.00043537	0.0078229	0.00635676	18
BP	GO:0006397	mRNA processing	141/4071	500/18614	0.00044168	0.00792386	0.0064388	141
BP	GO:0010586	miRNA metabolic process	36/4071	97/18614	0.00044272	0.00792997	0.00644377	36
BP	GO:0048193	Golgi vesicle transport	89/4071	294/18614	0.00044345	0.00793072	0.00644438	89
BP	GO:0050679	positive regulation of epithelial cell proliferation	71/4071	225/18614	0.00044741	0.00798896	0.0064917	71
BP	GO:1903829	positive regulation of protein localization	133/4071	468/18614	0.00044883	0.00799627	0.00649764	133
BP	GO:0046349	amino sugar biosynthetic process	8/4071	11/18614	0.00045062	0.00799627	0.00649764	8

BP	GO:0060623	regulation of chromosome condensation	8/4071	11/18614	0.00045062	0.00799627	0.00649764	8
BP	GO:0060900	embryonic camera-type eye formation	8/4071	11/18614	0.00045062	0.00799627	0.00649764	8
BP	GO:0001933	negative regulation of protein phosphorylation	101/4071	341/18614	0.00045286	0.00802346	0.00651973	101
BP	GO:0002292	T cell differentiation involved in immune response	31/4071	80/18614	0.00045649	0.00806277	0.00655167	31
BP	GO:0070227	lymphocyte apoptotic process	31/4071	80/18614	0.00045649	0.00806277	0.00655167	31
BP	GO:0007568	aging	56/4071	169/18614	0.00045876	0.00809037	0.0065741	56
BP	GO:0036293	response to decreased oxygen levels	92/4071	306/18614	0.00046229	0.00813743	0.00661234	92
BP	GO:0006268	DNA unwinding involved in DNA replication	12/4071	21/18614	0.00046571	0.00813743	0.00661234	12
BP	GO:0006490	oligosaccharide-lipid intermediate biosynthetic process	12/4071	21/18614	0.00046571	0.00813743	0.00661234	12
BP	GO:0014856	skeletal muscle cell proliferation	12/4071	21/18614	0.00046571	0.00813743	0.00661234	12
BP	GO:0030728	ovulation	12/4071	21/18614	0.00046571	0.00813743	0.00661234	12
BP	GO:0051315	attachment of mitotic spindle microtubules to kinetochore	12/4071	21/18614	0.00046571	0.00813743	0.00661234	12
BP	GO:0043388	positive regulation of DNA binding	24/4071	57/18614	0.00047713	0.00830229	0.0067463	24
BP	GO:0072132	mesenchyme morphogenesis	24/4071	57/18614	0.00047713	0.00830229	0.0067463	24
BP	GO:0071675	regulation of mononuclear cell migration	43/4071	122/18614	0.00047862	0.00830229	0.0067463	43
BP	GO:0090100	positive regulation of transmembrane receptor protein serine/threonine kinase signaling pathway	43/4071	122/18614	0.00047862	0.00830229	0.0067463	43
BP	GO:0007548	sex differentiation	86/4071	283/18614	0.00047878	0.00830229	0.0067463	86
BP	GO:0050852	T cell receptor signaling pathway	48/4071	140/18614	0.00048051	0.00831957	0.00676035	48
BP	GO:0007259	receptor signaling pathway via JAK-STAT	57/4071	173/18614	0.00048212	0.00832222	0.0067625	57
BP	GO:0031214	biomineral tissue development	57/4071	173/18614	0.00048212	0.00832222	0.0067625	57
BP	GO:0048024	regulation of mRNA splicing, via spliceosome	39/4071	108/18614	0.00049627	0.00849251	0.00690088	39
BP	GO:0051656	establishment of organelle localization	127/4071	445/18614	0.00049657	0.00849251	0.00690088	127

BP	GO:0010957	negative regulation of vitamin D biosynthetic process	5/4071	5/18614	0.00049943	0.00849251	0.00690088	5
BP	GO:0018057	peptidyl-lysine oxidation	5/4071	5/18614	0.00049943	0.00849251	0.00690088	5
BP	GO:0043378	positive regulation of CD8-positive, alpha-beta T cell differentiation	5/4071	5/18614	0.00049943	0.00849251	0.00690088	5
BP	GO:0060369	positive regulation of Fc receptor mediated stimulatory signaling pathway	5/4071	5/18614	0.00049943	0.00849251	0.00690088	5
BP	GO:0061197	fungiform papilla morphogenesis	5/4071	5/18614	0.00049943	0.00849251	0.00690088	5
BP	GO:0061550	cranial ganglion development	5/4071	5/18614	0.00049943	0.00849251	0.00690088	5
BP	GO:1903912	negative regulation of endoplasmic reticulum stress-induced eIF2 alpha phosphorylation	5/4071	5/18614	0.00049943	0.00849251	0.00690088	5
BP	GO:1905007	positive regulation of epithelial to mesenchymal transition involved in endocardial cushion formation	5/4071	5/18614	0.00049943	0.00849251	0.00690088	5
BP	GO:0034605	cellular response to heat	27/4071	67/18614	0.00050578	0.00858768	0.00697821	27
BP	GO:0003205	cardiac chamber development	55/4071	166/18614	0.00051437	0.00872065	0.00708626	55
BP	GO:0003206	cardiac chamber morphogenesis	44/4071	126/18614	0.00051992	0.00880158	0.00715202	44
BP	GO:0006470	protein dephosphorylation	77/4071	249/18614	0.00052162	0.00881733	0.00716482	77
BP	GO:0000281	mitotic cytokinesis	32/4071	84/18614	0.00053016	0.00891598	0.00724498	32
BP	GO:0002399	MHC class II protein complex assembly	10/4071	16/18614	0.00053136	0.00891598	0.00724498	10
BP	GO:0002503	peptide antigen assembly with MHC class II protein complex	10/4071	16/18614	0.00053136	0.00891598	0.00724498	10
BP	GO:0038065	collagen-activated signaling pathway	10/4071	16/18614	0.00053136	0.00891598	0.00724498	10
BP	GO:0045651	positive regulation of macrophage differentiation	10/4071	16/18614	0.00053136	0.00891598	0.00724498	10
BP	GO:0007498	mesoderm development	47/4071	137/18614	0.00053859	0.00902394	0.0073327	47
BP	GO:1903034	regulation of response to wounding	56/4071	170/18614	0.00054053	0.00904313	0.0073483	56
BP	GO:0002053	positive regulation of mesenchymal cell proliferation	13/4071	24/18614	0.00054434	0.00909351	0.00738923	13

BP	GO:0032611	interleukin-1 beta production	40/4071	112/18614	0.00054734	0.00910641	0.00739972	40
BP	GO:0032651	regulation of interleukin-1 beta production	40/4071	112/18614	0.00054734	0.00910641	0.00739972	40
BP	GO:0060537	muscle tissue development	122/4071	426/18614	0.0005476	0.00910641	0.00739972	122
BP	GO:0018209	peptidyl-serine modification	97/4071	327/18614	0.0005483	0.00910641	0.00739972	97
BP	GO:0043523	regulation of neuron apoptotic process	69/4071	219/18614	0.00055971	0.00926655	0.00752985	69
BP	GO:0002246	wound healing involved in inflammatory response	7/4071	9/18614	0.000562	0.00926655	0.00752985	7
BP	GO:0002578	negative regulation of antigen processing and presentation	7/4071	9/18614	0.000562	0.00926655	0.00752985	7
BP	GO:0060700	regulation of ribonuclease activity	7/4071	9/18614	0.000562	0.00926655	0.00752985	7
BP	GO:0061549	sympathetic ganglion development	7/4071	9/18614	0.000562	0.00926655	0.00752985	7
BP	GO:2000628	regulation of miRNA metabolic process	31/4071	81/18614	0.00058643	0.00965544	0.00784585	31
BP	GO:1902074	response to salt	111/4071	383/18614	0.00059479	0.00977893	0.0079462	111
BP	GO:0046639	negative regulation of alpha-beta T cell differentiation	14/4071	27/18614	0.00060001	0.00985055	0.0080044	14
BP	GO:0046636	negative regulation of alpha-beta T cell activation	20/4071	45/18614	0.00060258	0.00987852	0.00802712	20
BP	GO:0048565	digestive tract development	46/4071	134/18614	0.00060375	0.00988344	0.00803112	46
BP	GO:0002294	CD4-positive, alpha-beta T cell differentiation involved in immune response	28/4071	71/18614	0.0006074	0.00992903	0.00806817	28
BP	GO:0033209	tumor necrosis factor-mediated signaling pathway	39/4071	109/18614	0.00061184	0.00996032	0.00809359	39
BP	GO:0097530	granulocyte migration	52/4071	156/18614	0.00061378	0.00996032	0.00809359	52
BP	GO:0043484	regulation of RNA splicing	59/4071	182/18614	0.00061752	0.00996032	0.00809359	59
BP	GO:0042472	inner ear morphogenesis	37/4071	102/18614	0.00061836	0.00996032	0.00809359	37
BP	GO:0007442	hindgut morphogenesis	6/4071	7/18614	0.00062083	0.00996032	0.00809359	6
BP	GO:0021546	rhombomere development	6/4071	7/18614	0.00062083	0.00996032	0.00809359	6
BP	GO:0035723	interleukin-15-mediated signaling pathway	6/4071	7/18614	0.00062083	0.00996032	0.00809359	6
BP	GO:0036492	eiF2alpha phosphorylation in	6/4071	7/18614	0.00062083	0.00996032	0.00809359	6

		response to endoplasmic reticulum stress						
BP	GO:0046137	negative regulation of vitamin metabolic process	6/4071	7/18614	0.00062083	0.00996032	0.00809359	6
BP	GO:0071350	cellular response to interleukin-15	6/4071	7/18614	0.00062083	0.00996032	0.00809359	6
BP	GO:1902523	positive regulation of protein K63-linked ubiquitination	6/4071	7/18614	0.00062083	0.00996032	0.00809359	6
BP	GO:1903490	positive regulation of mitotic cytokinesis	6/4071	7/18614	0.00062083	0.00996032	0.00809359	6
BP	GO:1905462	regulation of DNA duplex unwinding	6/4071	7/18614	0.00062083	0.00996032	0.00809359	6
BP	GO:0010001	glial cell differentiation	73/4071	235/18614	0.00062154	0.00996032	0.00809359	73
BP	GO:0060338	regulation of type I interferon-mediated signaling pathway	19/4071	42/18614	0.00062666	0.01002837	0.00814889	19
BP	GO:0045740	positive regulation of DNA replication	18/4071	39/18614	0.00064449	0.01029915	0.00836892	18
BP	GO:0002200	somatic diversification of immune receptors	30/4071	78/18614	0.00064809	0.01034229	0.00840398	30
BP	GO:0036336	dendritic cell migration	16/4071	33/18614	0.00065148	0.01036306	0.00842085	16
BP	GO:0071711	basement membrane organization	16/4071	33/18614	0.00065148	0.01036306	0.00842085	16
BP	GO:0006906	vesicle fusion	42/4071	120/18614	0.00065212	0.01036306	0.00842085	42
BP	GO:0045589	regulation of regulatory T cell differentiation	17/4071	36/18614	0.00065369	0.01037356	0.00842939	17
BP	GO:0035019	somatic stem cell population maintenance	27/4071	68/18614	0.000666	0.01055424	0.0085762	27
BP	GO:0044774	mitotic DNA integrity checkpoint signaling	32/4071	85/18614	0.00067414	0.0106685	0.00866905	32
BP	GO:0030183	B cell differentiation	51/4071	153/18614	0.00068842	0.01087534	0.00883712	51
BP	GO:0060349	bone morphogenesis	36/4071	99/18614	0.00069007	0.01087534	0.00883712	36
BP	GO:2001243	negative regulation of intrinsic apoptotic signaling pathway	36/4071	99/18614	0.00069007	0.01087534	0.00883712	36
BP	GO:0007076	mitotic chromosome condensation	11/4071	19/18614	0.0006955	0.01093063	0.00888205	11
BP	GO:0010759	positive regulation of macrophage chemotaxis	11/4071	19/18614	0.0006955	0.01093063	0.00888205	11
BP	GO:0071897	DNA biosynthetic process	63/4071	198/18614	0.0007151	0.01122318	0.00911977	63
BP	GO:0030177	positive regulation of Wnt signaling pathway	49/4071	146/18614	0.00073033	0.01143182	0.00928931	49
BP	GO:0071887	leukocyte apoptotic process	41/4071	117/18614	0.00073039	0.01143182	0.00928931	41
BP	GO:0030900	forebrain development	114/4071	397/18614	0.0007335	0.01146473	0.00931606	114

BP	GO:0051091	positive regulation of DNA-binding transcription factor activity	82/4071	271/18614	0.00073704	0.01150435	0.00934825	82
BP	GO:0006968	cellular defense response	22/4071	52/18614	0.00074876	0.0116395	0.00945807	22
BP	GO:0060324	face development	22/4071	52/18614	0.00074876	0.0116395	0.00945807	22
BP	GO:2000677	regulation of transcription regulatory region DNA binding	22/4071	52/18614	0.00074876	0.0116395	0.00945807	22
BP	GO:0002444	myeloid leukocyte mediated immunity	39/4071	110/18614	0.0007506	0.01165219	0.00946838	39
BP	GO:0051591	response to cAMP	33/4071	89/18614	0.0007652	0.0117951	0.0095845	33
BP	GO:2000177	regulation of neural precursor cell proliferation	33/4071	89/18614	0.0007652	0.0117951	0.0095845	33
BP	GO:0006047	UDP-N-acetylglucosamine metabolic process	9/4071	14/18614	0.00076704	0.0117951	0.0095845	9
BP	GO:0014841	skeletal muscle satellite cell proliferation	9/4071	14/18614	0.00076704	0.0117951	0.0095845	9
BP	GO:0032486	Rap protein signal transduction	9/4071	14/18614	0.00076704	0.0117951	0.0095845	9
BP	GO:0051255	spindle midzone assembly	9/4071	14/18614	0.00076704	0.0117951	0.0095845	9
BP	GO:0060019	radial glial cell differentiation	9/4071	14/18614	0.00076704	0.0117951	0.0095845	9
BP	GO:0007044	cell-substrate junction assembly	35/4071	96/18614	0.00076997	0.01182416	0.00960812	35
BP	GO:0050777	negative regulation of immune response	61/4071	191/18614	0.00077428	0.01187445	0.00964898	61
BP	GO:0002287	alpha-beta T cell activation involved in immune response	28/4071	72/18614	0.00078897	0.01203502	0.00977946	28
BP	GO:0002293	alpha-beta T cell differentiation involved in immune response	28/4071	72/18614	0.00078897	0.01203502	0.00977946	28
BP	GO:0071677	positive regulation of mononuclear cell migration	28/4071	72/18614	0.00078897	0.01203502	0.00977946	28
BP	GO:2000036	regulation of stem cell population maintenance	28/4071	72/18614	0.00078897	0.01203502	0.00977946	28
BP	GO:0140895	cell surface toll-like receptor signaling pathway	21/4071	49/18614	0.00079974	0.01218298	0.00989969	21
BP	GO:0031532	actin cytoskeleton reorganization	40/4071	114/18614	0.00081811	0.01244614	0.01011353	40
BP	GO:0042026	protein refolding	12/4071	22/18614	0.00082126	0.01246091	0.01012553	12

BP	GO:0043371	negative regulation of CD4-positive, alpha-beta T cell differentiation	12/4071	22/18614	0.00082126	0.01246091	0.01012553	12
BP	GO:0032465	regulation of cytokinesis	34/4071	93/18614	0.00085894	0.01301523	0.01057597	34
BP	GO:0048545	response to steroid hormone	99/4071	339/18614	0.0008643	0.01305927	0.01061175	99
BP	GO:0051321	meiotic cell cycle	85/4071	284/18614	0.0008646	0.01305927	0.01061175	85
BP	GO:0006486	protein glycosylation	70/4071	226/18614	0.00086642	0.01305927	0.01061175	70
BP	GO:0043413	macromolecule glycosylation	70/4071	226/18614	0.00086642	0.01305927	0.01061175	70
BP	GO:0002711	positive regulation of T cell mediated immunity	27/4071	69/18614	0.00086884	0.01307848	0.01062736	27
BP	GO:0010038	response to metal ion	106/4071	367/18614	0.00088628	0.01332344	0.01082641	106
BP	GO:0090130	tissue migration	109/4071	379/18614	0.00089118	0.01337945	0.01087192	109
BP	GO:0016266	O-glycan processing	19/4071	43/18614	0.00089465	0.01339623	0.01088556	19
BP	GO:0031952	regulation of protein autophosphorylation	19/4071	43/18614	0.00089465	0.01339623	0.01088556	19
BP	GO:0031579	membrane raft organization	13/4071	25/18614	0.000908	0.0135606	0.01101912	13
BP	GO:2000001	regulation of DNA damage checkpoint	13/4071	25/18614	0.000908	0.0135606	0.01101912	13
BP	GO:2001252	positive regulation of chromosome organization	39/4071	111/18614	0.0009164	0.0136682	0.01110655	39
BP	GO:0060337	type I interferon-mediated signaling pathway	29/4071	76/18614	0.00091822	0.01367738	0.01111402	29
BP	GO:1903046	meiotic cell cycle process	67/4071	215/18614	0.00092336	0.01373607	0.01116171	67
BP	GO:1901215	negative regulation of neuron death	68/4071	219/18614	0.00094595	0.01403063	0.01140106	68
BP	GO:0014068	positive regulation of phosphatidylinositol 3-kinase signaling	31/4071	83/18614	0.00094685	0.01403063	0.01140106	31
BP	GO:0051851	modulation by host of symbiont process	31/4071	83/18614	0.00094685	0.01403063	0.01140106	31
BP	GO:0002698	negative regulation of immune effector process	42/4071	122/18614	0.00095432	0.01409214	0.01145104	42
BP	GO:0090174	organelle membrane fusion	42/4071	122/18614	0.00095432	0.01409214	0.01145104	42
BP	GO:0001657	ureteric bud development	35/4071	97/18614	0.00095471	0.01409214	0.01145104	35
BP	GO:0034063	stress granule assembly	14/4071	28/18614	0.00095988	0.01415013	0.01149817	14
BP	GO:0043516	regulation of DNA damage response, signal transduction by p53 class mediator	17/4071	37/18614	0.00096491	0.01418484	0.01152636	17
BP	GO:1902745	positive regulation of lamellipodium organization	17/4071	37/18614	0.00096491	0.01418484	0.01152636	17
BP	GO:0034329	cell junction assembly	125/4071	444/18614	0.00096596	0.01418484	0.01152636	125
BP	GO:0030168	platelet activation	45/4071	133/18614	0.00097165	0.01423782	0.01156942	45

BP	GO:0014065	phosphatidylinositol 3-kinase signaling	48/4071	144/18614	0.00097207	0.01423782	0.01156942	48
BP	GO:0060351	cartilage development involved in endochondral bone morphogenesis	16/4071	34/18614	0.00098261	0.01426607	0.01159238	16
BP	GO:0060563	neuroepithelial cell differentiation	16/4071	34/18614	0.00098261	0.01426607	0.01159238	16
BP	GO:1901797	negative regulation of signal transduction by p53 class mediator	16/4071	34/18614	0.00098261	0.01426607	0.01159238	16
BP	GO:0035335	peptidyl-tyrosine dephosphorylation	15/4071	31/18614	0.00098275	0.01426607	0.01159238	15
BP	GO:0061437	renal system vasculature development	15/4071	31/18614	0.00098275	0.01426607	0.01159238	15
BP	GO:0061440	kidney vasculature development	15/4071	31/18614	0.00098275	0.01426607	0.01159238	15
BP	GO:1900225	regulation of NLRP3 inflammasome complex assembly	15/4071	31/18614	0.00098275	0.01426607	0.01159238	15
BP	GO:0003231	cardiac ventricle development	43/4071	126/18614	0.00102248	0.01482397	0.01204572	43
BP	GO:0061041	regulation of wound healing	46/4071	137/18614	0.00103152	0.01493158	0.01213316	46
BP	GO:0031053	primary miRNA processing	10/4071	17/18614	0.00103775	0.01493158	0.01213316	10
BP	GO:0034138	toll-like receptor 3 signaling pathway	10/4071	17/18614	0.00103775	0.01493158	0.01213316	10
BP	GO:0051382	kinetochore assembly	10/4071	17/18614	0.00103775	0.01493158	0.01213316	10
BP	GO:0061548	ganglion development	10/4071	17/18614	0.00103775	0.01493158	0.01213316	10
BP	GO:1902916	positive regulation of protein polyubiquitination	10/4071	17/18614	0.00103775	0.01493158	0.01213316	10
BP	GO:0035904	aorta development	25/4071	63/18614	0.0010486	0.01506867	0.01224455	25
BP	GO:0048644	muscle organ morphogenesis	30/4071	80/18614	0.00105248	0.01508645	0.012259	30
BP	GO:0070972	protein localization to endoplasmic reticulum	30/4071	80/18614	0.00105248	0.01508645	0.012259	30
BP	GO:0030278	regulation of ossification	41/4071	119/18614	0.00107036	0.01532341	0.01245156	41
BP	GO:0032434	regulation of proteasomal ubiquitin-dependent protein catabolic process	47/4071	141/18614	0.00109084	0.0155148	0.01260707	47
BP	GO:0000727	double-strand break repair via break-induced replication	8/4071	12/18614	0.00109188	0.0155148	0.01260707	8
BP	GO:0014842	regulation of skeletal muscle satellite cell proliferation	8/4071	12/18614	0.00109188	0.0155148	0.01260707	8
BP	GO:0051095	regulation of helicase activity	8/4071	12/18614	0.00109188	0.0155148	0.01260707	8

BP	GO:0090232	positive regulation of spindle checkpoint	8/4071	12/18614	0.00109188	0.0155148	0.01260707	8
BP	GO:0090267	positive regulation of mitotic cell cycle spindle assembly checkpoint	8/4071	12/18614	0.00109188	0.0155148	0.01260707	8
BP	GO:0045581	negative regulation of T cell differentiation	21/4071	50/18614	0.00109772	0.01557828	0.01265866	21
BP	GO:0014066	regulation of phosphatidylinositol 3-kinase signaling	39/4071	112/18614	0.00111358	0.01578375	0.01282562	39
BP	GO:0050808	synapse organization	130/4071	466/18614	0.00113505	0.01605988	0.01304999	130
BP	GO:0000724	double-strand break repair via homologous recombination	55/4071	171/18614	0.00113587	0.01605988	0.01304999	55
BP	GO:0071357	cellular response to type I interferon	29/4071	77/18614	0.00116922	0.01649045	0.01339987	29
BP	GO:0034620	cellular response to unfolded protein	35/4071	98/18614	0.00117715	0.01649045	0.01339987	35
BP	GO:0072163	mesonephric epithelium development	35/4071	98/18614	0.00117715	0.01649045	0.01339987	35
BP	GO:0072164	mesonephric tubule development	35/4071	98/18614	0.00117715	0.01649045	0.01339987	35
BP	GO:0035196	miRNA processing	20/4071	47/18614	0.0011772	0.01649045	0.01339987	20
BP	GO:0045646	regulation of erythrocyte differentiation	20/4071	47/18614	0.0011772	0.01649045	0.01339987	20
BP	GO:1902459	positive regulation of stem cell population maintenance	20/4071	47/18614	0.0011772	0.01649045	0.01339987	20
BP	GO:0000725	recombinational repair	56/4071	175/18614	0.00117789	0.01649045	0.01339987	56
BP	GO:0043010	camera-type eye development	98/4071	338/18614	0.00118212	0.01652935	0.01343148	98
BP	GO:0001942	hair follicle development	33/4071	91/18614	0.00119187	0.01662493	0.01350914	33
BP	GO:0072091	regulation of stem cell proliferation	33/4071	91/18614	0.00119187	0.01662493	0.01350914	33
BP	GO:0072009	nephron epithelium development	40/4071	116/18614	0.00120065	0.01672694	0.01359204	40
BP	GO:0022613	ribonucleoprotein complex biogenesis	132/4071	475/18614	0.00122691	0.01707197	0.0138724	132
BP	GO:0002544	chronic inflammatory response	11/4071	20/18614	0.00124108	0.01720622	0.01398149	11
BP	GO:0002643	regulation of tolerance induction	11/4071	20/18614	0.00124108	0.01720622	0.01398149	11
BP	GO:0006488	dolichol-linked oligosaccharide biosynthetic process	11/4071	20/18614	0.00124108	0.01720622	0.01398149	11
BP	GO:1904035	regulation of epithelial cell apoptotic process	38/4071	109/18614	0.00124853	0.01728854	0.01404838	38
BP	GO:0009266	response to temperature stimulus	58/4071	183/18614	0.00125887	0.01741059	0.01414756	58

BP	GO:0043200	response to amino acid	41/4071	120/18614	0.00128742	0.01777607	0.01444455	41
BP	GO:0001823	mesonephros development	36/4071	102/18614	0.00128841	0.01777607	0.01444455	36
BP	GO:0007565	female pregnancy	59/4071	187/18614	0.00129776	0.01788334	0.01453171	59
BP	GO:0002753	cytosolic pattern recognition receptor signaling pathway	34/4071	95/18614	0.00131726	0.01810831	0.01471452	34
BP	GO:0014033	neural crest cell differentiation	34/4071	95/18614	0.00131726	0.01810831	0.01471452	34
BP	GO:0014855	striated muscle cell proliferation	30/4071	81/18614	0.00132663	0.01819329	0.01478357	30
BP	GO:0044773	mitotic DNA damage checkpoint signaling	30/4071	81/18614	0.00132663	0.01819329	0.01478357	30
BP	GO:0044344	cellular response to fibroblast growth factor stimulus	39/4071	113/18614	0.00134697	0.01845008	0.01499223	39
BP	GO:0030100	regulation of endocytosis	66/4071	214/18614	0.001349	0.0184558	0.01499688	66
BP	GO:0055123	digestive system development	48/4071	146/18614	0.00135472	0.01851183	0.01504241	48
BP	GO:0031098	stress-activated protein kinase signaling cascade	74/4071	245/18614	0.0013672	0.01862618	0.01513533	74
BP	GO:0070085	glycosylation	74/4071	245/18614	0.0013672	0.01862618	0.01513533	74
BP	GO:1902743	regulation of lamellipodium organization	22/4071	54/18614	0.00136799	0.01862618	0.01513533	22
BP	GO:0045591	positive regulation of regulatory T cell differentiation	12/4071	23/18614	0.00137712	0.01870589	0.0152001	12
BP	GO:0061042	vascular wound healing	12/4071	23/18614	0.00137712	0.01870589	0.0152001	12
BP	GO:0033002	muscle cell proliferation	75/4071	249/18614	0.00138634	0.01880867	0.01528362	75
BP	GO:0071375	cellular response to peptide hormone stimulus	90/4071	308/18614	0.00141362	0.0191561	0.01556593	90
BP	GO:0031668	cellular response to extracellular stimulus	78/4071	261/18614	0.00143844	0.01941715	0.01577806	78
BP	GO:0002562	somatic diversification of immune receptors via germline recombination within a single locus	27/4071	71/18614	0.00143996	0.01941715	0.01577806	27
BP	GO:0016444	somatic cell DNA recombination	27/4071	71/18614	0.00143996	0.01941715	0.01577806	27
BP	GO:0048002	antigen processing and presentation of peptide antigen	27/4071	71/18614	0.00143996	0.01941715	0.01577806	27
BP	GO:0002495	antigen processing and presentation of peptide antigen via MHC class II	16/4071	35/18614	0.00144583	0.01941715	0.01577806	16
BP	GO:0035633	maintenance of blood-brain barrier	16/4071	35/18614	0.00144583	0.01941715	0.01577806	16

BP	GO:1902253	regulation of intrinsic apoptotic signaling pathway by p53 class mediator	16/4071	35/18614	0.00144583	0.01941715	0.01577806	16
BP	GO:0051604	protein maturation	96/4071	332/18614	0.0014465	0.01941715	0.01577806	96
BP	GO:0097696	receptor signaling pathway via STAT	58/4071	184/18614	0.00145293	0.0194805	0.01582954	58
BP	GO:0048596	embryonic camera-type eye morphogenesis	13/4071	26/18614	0.00145469	0.01948122	0.01583012	13
BP	GO:0002431	Fc receptor mediated stimulatory signaling pathway	15/4071	32/18614	0.00147889	0.01975898	0.01605582	15
BP	GO:0010165	response to X-ray	15/4071	32/18614	0.00147889	0.01975898	0.01605582	15
BP	GO:0051090	regulation of DNA-binding transcription factor activity	129/4071	465/18614	0.00148266	0.01977106	0.01606564	129
BP	GO:0002052	positive regulation of neuroblast proliferation	14/4071	29/18614	0.001485	0.01977106	0.01606564	14
BP	GO:0090025	regulation of monocyte chemotaxis	14/4071	29/18614	0.001485	0.01977106	0.01606564	14
BP	GO:0051960	regulation of nervous system development	128/4071	461/18614	0.00149073	0.01982424	0.01610885	128
BP	GO:0043903	regulation of biological process involved in symbiotic interaction	24/4071	61/18614	0.00150598	0.02000374	0.01625471	24
BP	GO:0045445	myoblast differentiation	38/4071	110/18614	0.00151133	0.02002398	0.01627116	38
BP	GO:0008298	intracellular mRNA localization	7/4071	10/18614	0.00151803	0.02002398	0.01627116	7
BP	GO:0021561	facial nerve development	7/4071	10/18614	0.00151803	0.02002398	0.01627116	7
BP	GO:0021610	facial nerve morphogenesis	7/4071	10/18614	0.00151803	0.02002398	0.01627116	7
BP	GO:0060368	regulation of Fc receptor mediated stimulatory signaling pathway	7/4071	10/18614	0.00151803	0.02002398	0.01627116	7
BP	GO:1902412	regulation of mitotic cytokinesis	7/4071	10/18614	0.00151803	0.02002398	0.01627116	7
BP	GO:2001242	regulation of intrinsic apoptotic signaling pathway	55/4071	173/18614	0.00153	0.02015849	0.01638046	55
BP	GO:0045444	fat cell differentiation	74/4071	246/18614	0.00154378	0.02022543	0.01643486	74
BP	GO:0030174	regulation of DNA-templated DNA replication initiation	9/4071	15/18614	0.00154571	0.02022543	0.01643486	9
BP	GO:0043518	negative regulation of DNA damage response, signal transduction by p53 class mediator	9/4071	15/18614	0.00154571	0.02022543	0.01643486	9
BP	GO:0045176	apical protein localization	9/4071	15/18614	0.00154571	0.02022543	0.01643486	9

BP	GO:0048934	peripheral nervous system neuron differentiation	9/4071	15/18614	0.00154571	0.02022543	0.01643486	9
BP	GO:0048935	peripheral nervous system neuron development	9/4071	15/18614	0.00154571	0.02022543	0.01643486	9
BP	GO:0048608	reproductive structure development	88/4071	301/18614	0.00155851	0.02036949	0.01655192	88
BP	GO:0061458	reproductive system development	89/4071	305/18614	0.00156637	0.02044879	0.01661635	89
BP	GO:0008630	intrinsic apoptotic signaling pathway in response to DNA damage	36/4071	103/18614	0.00156997	0.02047243	0.01663556	36
BP	GO:0016445	somatic diversification of immunoglobulins	26/4071	68/18614	0.00159602	0.02076459	0.01687297	26
BP	GO:2000144	positive regulation of DNA-templated transcription initiation	26/4071	68/18614	0.00159602	0.02076459	0.01687297	26
BP	GO:0000375	RNA splicing, via transesterification reactions	95/4071	329/18614	0.00159967	0.02078841	0.01689232	95
BP	GO:0035850	epithelial cell differentiation involved in kidney development	20/4071	48/18614	0.00160793	0.02087202	0.01696026	20
BP	GO:0002690	positive regulation of leukocyte chemotaxis	34/4071	96/18614	0.00161738	0.02092328	0.01700191	34
BP	GO:0045807	positive regulation of endocytosis	34/4071	96/18614	0.00161738	0.02092328	0.01700191	34
BP	GO:0070664	negative regulation of leukocyte proliferation	34/4071	96/18614	0.00161738	0.02092328	0.01700191	34
BP	GO:0001707	mesoderm formation	28/4071	75/18614	0.00164509	0.0211856	0.01721507	28
BP	GO:0043536	positive regulation of blood vessel endothelial cell migration	28/4071	75/18614	0.00164509	0.0211856	0.01721507	28
BP	GO:0045669	positive regulation of osteoblast differentiation	28/4071	75/18614	0.00164509	0.0211856	0.01721507	28
BP	GO:0050818	regulation of coagulation	28/4071	75/18614	0.00164509	0.0211856	0.01721507	28
BP	GO:0061005	cell differentiation involved in kidney development	23/4071	58/18614	0.00165505	0.02128993	0.01729985	23
BP	GO:0006446	regulation of translational initiation	30/4071	82/18614	0.00166055	0.02131256	0.01731824	30
BP	GO:0006513	protein monoubiquitination	30/4071	82/18614	0.00166055	0.02131256	0.01731824	30
BP	GO:0051051	negative regulation of transport	133/4071	483/18614	0.00171475	0.02198347	0.0178634	133
BP	GO:0042254	ribosome biogenesis	87/4071	298/18614	0.00172722	0.02210252	0.01796015	87

BP	GO:0035967	cellular response to topologically incorrect protein	40/4071	118/18614	0.00173151	0.02210252	0.01796015	40
BP	GO:0051702	biological process involved in interaction with symbiont	40/4071	118/18614	0.00173151	0.02210252	0.01796015	40
BP	GO:1902692	regulation of neuroblast proliferation	19/4071	45/18614	0.00173178	0.02210252	0.01796015	19
BP	GO:0090132	epithelium migration	106/4071	374/18614	0.00176982	0.02256279	0.01833415	106
BP	GO:0060260	regulation of transcription initiation by RNA polymerase II	27/4071	72/18614	0.00183056	0.02331099	0.01894213	27
BP	GO:0008625	extrinsic apoptotic signaling pathway via death domain receptors	31/4071	86/18614	0.00184534	0.02344687	0.01905255	31
BP	GO:2000134	negative regulation of G1/S transition of mitotic cell cycle	31/4071	86/18614	0.00184534	0.02344687	0.01905255	31
BP	GO:0002705	positive regulation of leukocyte mediated immunity	48/4071	148/18614	0.00186376	0.02365462	0.01922136	48
BP	GO:0002371	dendritic cell cytokine production	10/4071	18/18614	0.00187884	0.02366181	0.0192272	10
BP	GO:0035455	response to interferon-alpha	10/4071	18/18614	0.00187884	0.02366181	0.0192272	10
BP	GO:0036003	positive regulation of transcription from RNA polymerase II promoter in response to stress	10/4071	18/18614	0.00187884	0.02366181	0.0192272	10
BP	GO:0036499	PERK-mediated unfolded protein response	10/4071	18/18614	0.00187884	0.02366181	0.0192272	10
BP	GO:0045064	T-helper 2 cell differentiation	10/4071	18/18614	0.00187884	0.02366181	0.0192272	10
BP	GO:0051770	positive regulation of nitric-oxide synthase biosynthetic process	10/4071	18/18614	0.00187884	0.02366181	0.0192272	10
BP	GO:1901978	positive regulation of cell cycle checkpoint	10/4071	18/18614	0.00187884	0.02366181	0.0192272	10
BP	GO:0035270	endocrine system development	45/4071	137/18614	0.00191625	0.0241063	0.01958839	45
BP	GO:0043405	regulation of MAP kinase activity	58/4071	186/18614	0.00192151	0.02414589	0.01962055	58
BP	GO:0032970	regulation of actin filament-based process	109/4071	387/18614	0.00193096	0.02423785	0.01969528	109
BP	GO:0002688	regulation of leukocyte chemotaxis	42/4071	126/18614	0.00194579	0.02439718	0.01982475	42
BP	GO:0010631	epithelial cell migration	105/4071	371/18614	0.00194952	0.02441711	0.01984094	105

BP	GO:0016447	somatic recombination of immunoglobulin gene segments	24/4071	62/18614	0.00195448	0.02445242	0.01986963	24
BP	GO:0031099	regeneration	59/4071	190/18614	0.00196885	0.02459608	0.01998637	59
BP	GO:0040001	establishment of mitotic spindle localization	17/4071	39/18614	0.00197243	0.02459608	0.01998637	17
BP	GO:0045066	regulatory T cell differentiation	17/4071	39/18614	0.00197243	0.02459608	0.01998637	17
BP	GO:0060425	lung morphogenesis	21/4071	52/18614	0.00198541	0.02473091	0.02009593	21
BP	GO:0006898	receptor-mediated endocytosis	76/4071	256/18614	0.00199804	0.02486113	0.02020174	76
BP	GO:0007584	response to nutrient	50/4071	156/18614	0.00201273	0.02492369	0.02025258	50
BP	GO:0048284	organelle fusion	50/4071	156/18614	0.00201273	0.02492369	0.02025258	50
BP	GO:0021612	facial nerve structural organization	6/4071	8/18614	0.00202054	0.02492369	0.02025258	6
BP	GO:0036491	regulation of translation initiation in response to endoplasmic reticulum stress	6/4071	8/18614	0.00202054	0.02492369	0.02025258	6
BP	GO:0043376	regulation of CD8-positive, alpha-beta T cell differentiation	6/4071	8/18614	0.00202054	0.02492369	0.02025258	6
BP	GO:0051665	membrane raft localization	6/4071	8/18614	0.00202054	0.02492369	0.02025258	6
BP	GO:0060839	endothelial cell fate commitment	6/4071	8/18614	0.00202054	0.02492369	0.02025258	6
BP	GO:0061525	hindgut development	6/4071	8/18614	0.00202054	0.02492369	0.02025258	6
BP	GO:0032868	response to insulin	80/4071	272/18614	0.00206319	0.02542225	0.0206577	80
BP	GO:0090026	positive regulation of monocyte chemotaxis	11/4071	21/18614	0.00209399	0.02569074	0.02087587	11
BP	GO:0090231	regulation of spindle checkpoint	11/4071	21/18614	0.00209399	0.02569074	0.02087587	11
BP	GO:0090266	regulation of mitotic cell cycle spindle assembly checkpoint	11/4071	21/18614	0.00209399	0.02569074	0.02087587	11
BP	GO:1903504	regulation of mitotic spindle checkpoint	11/4071	21/18614	0.00209399	0.02569074	0.02087587	11
BP	GO:0045137	development of primary sexual characteristics	70/4071	233/18614	0.00210465	0.02579384	0.02095965	70
BP	GO:0070228	regulation of lymphocyte apoptotic process	23/4071	59/18614	0.00215866	0.02642736	0.02147444	23
BP	GO:0032386	regulation of intracellular transport	95/4071	332/18614	0.00216641	0.02648982	0.02152519	95
BP	GO:1990776	response to angiotensin	15/4071	33/18614	0.0021684	0.02648982	0.02152519	15
BP	GO:0000079	regulation of cyclin-dependent protein serine/threonine kinase activity	38/4071	112/18614	0.0021843	0.02665542	0.02165975	38

BP	GO:0036120	cellular response to platelet-derived growth factor stimulus	12/4071	24/18614	0.00220991	0.02692175	0.02187617	12
BP	GO:1901888	regulation of cell junction assembly	65/4071	214/18614	0.00221499	0.02692175	0.02187617	65
BP	GO:0022404	molting cycle process	33/4071	94/18614	0.00221556	0.02692175	0.02187617	33
BP	GO:0022405	hair cycle process	33/4071	94/18614	0.00221556	0.02692175	0.02187617	33
BP	GO:0051291	protein heterooligomerization	13/4071	27/18614	0.00224832	0.02727552	0.02216364	13
BP	GO:0010517	regulation of phospholipase activity	25/4071	66/18614	0.00226225	0.02727552	0.02216364	25
BP	GO:0043254	regulation of protein-containing complex assembly	114/4071	409/18614	0.0022709	0.02727552	0.02216364	114
BP	GO:0002605	negative regulation of dendritic cell antigen processing and presentation	4/4071	4/18614	0.00228531	0.02727552	0.02216364	4
BP	GO:0007057	spindle assembly involved in female meiosis I	4/4071	4/18614	0.00228531	0.02727552	0.02216364	4
BP	GO:0007296	vitellogenesis	4/4071	4/18614	0.00228531	0.02727552	0.02216364	4
BP	GO:0038189	neuropilin signaling pathway	4/4071	4/18614	0.00228531	0.02727552	0.02216364	4
BP	GO:0045629	negative regulation of T-helper 2 cell differentiation	4/4071	4/18614	0.00228531	0.02727552	0.02216364	4
BP	GO:0061551	trigeminal ganglion development	4/4071	4/18614	0.00228531	0.02727552	0.02216364	4
BP	GO:0071284	cellular response to lead ion	4/4071	4/18614	0.00228531	0.02727552	0.02216364	4
BP	GO:0090500	endocardial cushion to mesenchymal transition	4/4071	4/18614	0.00228531	0.02727552	0.02216364	4
BP	GO:0097490	sympathetic neuron projection extension	4/4071	4/18614	0.00228531	0.02727552	0.02216364	4
BP	GO:0097491	sympathetic neuron projection guidance	4/4071	4/18614	0.00228531	0.02727552	0.02216364	4
BP	GO:1900127	positive regulation of hyaluronan biosynthetic process	4/4071	4/18614	0.00228531	0.02727552	0.02216364	4
BP	GO:1901407	regulation of phosphorylation of RNA polymerase II C-terminal domain	4/4071	4/18614	0.00228531	0.02727552	0.02216364	4
BP	GO:1904580	regulation of intracellular mRNA localization	4/4071	4/18614	0.00228531	0.02727552	0.02216364	4
BP	GO:1990441	negative regulation of transcription from RNA polymerase II promoter	4/4071	4/18614	0.00228531	0.02727552	0.02216364	4

		in response to endoplasmic reticulum stress						
BP	GO:0042117	monocyte activation	8/4071	13/18614	0.00229476	0.02730259	0.02218564	8
BP	GO:0070486	leukocyte aggregation	8/4071	13/18614	0.00229476	0.02730259	0.02218564	8
BP	GO:0071696	ectodermal placode development	8/4071	13/18614	0.00229476	0.02730259	0.02218564	8
BP	GO:0034502	protein localization to chromosome	39/4071	116/18614	0.00232138	0.02756189	0.02239634	39
BP	GO:0071346	cellular response to type II interferon	39/4071	116/18614	0.00232138	0.02756189	0.02239634	39
BP	GO:0031669	cellular response to nutrient levels	69/4071	230/18614	0.00234387	0.02779595	0.02258653	69
BP	GO:0010634	positive regulation of epithelial cell migration	55/4071	176/18614	0.00234597	0.02779595	0.02258653	55
BP	GO:0032467	positive regulation of cytokinesis	19/4071	46/18614	0.00235301	0.02782161	0.02260738	19
BP	GO:0042088	T-helper 1 type immune response	19/4071	46/18614	0.00235301	0.02782161	0.02260738	19
BP	GO:0032608	interferon-beta production	22/4071	56/18614	0.00238036	0.02804219	0.02278662	22
BP	GO:0032648	regulation of interferon-beta production	22/4071	56/18614	0.00238036	0.02804219	0.02278662	22
BP	GO:0045005	DNA-templated DNA replication maintenance of fidelity	22/4071	56/18614	0.00238036	0.02804219	0.02278662	22
BP	GO:0018105	peptidyl-serine phosphorylation	89/4071	309/18614	0.0023815	0.02804219	0.02278662	89
BP	GO:0035265	organ growth	56/4071	180/18614	0.00240555	0.02829619	0.02299302	56
BP	GO:0045598	regulation of fat cell differentiation	47/4071	146/18614	0.00244145	0.02854303	0.02319359	47
BP	GO:0010032	meiotic chromosome condensation	5/4071	6/18614	0.00245095	0.02854303	0.02319359	5
BP	GO:0031584	activation of phospholipase D activity	5/4071	6/18614	0.00245095	0.02854303	0.02319359	5
BP	GO:0072008	glomerular mesangial cell differentiation	5/4071	6/18614	0.00245095	0.02854303	0.02319359	5
BP	GO:1901166	neural crest cell migration involved in autonomic nervous system development	5/4071	6/18614	0.00245095	0.02854303	0.02319359	5
BP	GO:1901534	positive regulation of hematopoietic progenitor cell differentiation	5/4071	6/18614	0.00245095	0.02854303	0.02319359	5
BP	GO:1902747	negative regulation of lens fiber cell differentiation	5/4071	6/18614	0.00245095	0.02854303	0.02319359	5
BP	GO:1905005	regulation of epithelial to mesenchymal transition	5/4071	6/18614	0.00245095	0.02854303	0.02319359	5

		involved in endocardial cushion formation						
BP	GO:0001708	cell fate specification	37/4071	109/18614	0.00245405	0.02854303	0.02319359	37
BP	GO:0018107	peptidyl-threonine phosphorylation	37/4071	109/18614	0.00245405	0.02854303	0.02319359	37
BP	GO:0045132	meiotic chromosome segregation	37/4071	109/18614	0.00245405	0.02854303	0.02319359	37
BP	GO:0046578	regulation of Ras protein signal transduction	57/4071	184/18614	0.00246273	0.02861484	0.02325195	57
BP	GO:0016485	protein processing	74/4071	250/18614	0.00246812	0.02864822	0.02327907	74
BP	GO:0007519	skeletal muscle tissue development	52/4071	165/18614	0.00248682	0.02883597	0.02343164	52
BP	GO:0060393	regulation of pathway-restricted SMAD protein phosphorylation	24/4071	63/18614	0.00251194	0.02909774	0.02364435	24
BP	GO:0042476	odontogenesis	44/4071	135/18614	0.00252768	0.02925038	0.02376838	44
BP	GO:0034142	toll-like receptor 4 signaling pathway	18/4071	43/18614	0.00254584	0.02937111	0.02386648	18
BP	GO:0048713	regulation of oligodendrocyte differentiation	18/4071	43/18614	0.00254584	0.02937111	0.02386648	18
BP	GO:0071604	transforming growth factor beta production	18/4071	43/18614	0.00254584	0.02937111	0.02386648	18
BP	GO:0030193	regulation of blood coagulation	26/4071	70/18614	0.00257482	0.02964549	0.02408944	26
BP	GO:0043627	response to estrogen	26/4071	70/18614	0.00257482	0.02964549	0.02408944	26
BP	GO:0048332	mesoderm morphogenesis	28/4071	77/18614	0.00258331	0.02971326	0.0241445	28
BP	GO:0001909	leukocyte mediated cytotoxicity	45/4071	139/18614	0.0026355	0.030283	0.02460747	45
BP	GO:0006029	proteoglycan metabolic process	33/4071	95/18614	0.00269369	0.03092049	0.02512548	33
BP	GO:0043281	regulation of cysteine-type endopeptidase activity involved in apoptotic process	62/4071	204/18614	0.00271302	0.03109867	0.02527027	62
BP	GO:1990266	neutrophil migration	42/4071	128/18614	0.00271466	0.03109867	0.02527027	42
BP	GO:0071634	regulation of transforming growth factor beta production	17/4071	40/18614	0.00273973	0.03135442	0.02547808	17
BP	GO:0033273	response to vitamin	31/4071	88/18614	0.00278908	0.03182337	0.02585915	31
BP	GO:0048864	stem cell development	31/4071	88/18614	0.00278908	0.03182337	0.02585915	31
BP	GO:0050672	negative regulation of lymphocyte proliferation	31/4071	88/18614	0.00278908	0.03182337	0.02585915	31
BP	GO:0014857	regulation of skeletal muscle cell proliferation	9/4071	16/18614	0.00284987	0.03229102	0.02623916	9
BP	GO:0021783	preganglionic parasympathetic fiber development	9/4071	16/18614	0.00284987	0.03229102	0.02623916	9

BP	GO:0034134	toll-like receptor 2 signaling pathway	9/4071	16/18614	0.00284987	0.03229102	0.02623916	9
BP	GO:0042249	establishment of planar polarity of embryonic epithelium	9/4071	16/18614	0.00284987	0.03229102	0.02623916	9
BP	GO:0048532	anatomical structure arrangement	9/4071	16/18614	0.00284987	0.03229102	0.02623916	9
BP	GO:0060576	intestinal epithelial cell development	9/4071	16/18614	0.00284987	0.03229102	0.02623916	9
BP	GO:1902033	regulation of hematopoietic stem cell proliferation	9/4071	16/18614	0.00284987	0.03229102	0.02623916	9
BP	GO:0051348	negative regulation of transferase activity	81/4071	279/18614	0.00287076	0.0324954	0.02640523	81
BP	GO:0090102	cochlea development	20/4071	50/18614	0.00287703	0.0325019	0.02641051	20
BP	GO:1902041	regulation of extrinsic apoptotic signaling pathway via death domain receptors	20/4071	50/18614	0.00287703	0.0325019	0.02641051	20
BP	GO:0006023	aminoglycan biosynthetic process	27/4071	74/18614	0.00288911	0.03254167	0.02644283	27
BP	GO:0051298	centrosome duplication	27/4071	74/18614	0.00288911	0.03254167	0.02644283	27
BP	GO:2000573	positive regulation of DNA biosynthetic process	27/4071	74/18614	0.00288911	0.03254167	0.02644283	27
BP	GO:2001237	negative regulation of extrinsic apoptotic signaling pathway	34/4071	99/18614	0.00289861	0.03261647	0.02650361	34
BP	GO:0030522	intracellular receptor signaling pathway	86/4071	299/18614	0.00290655	0.03267366	0.02655008	86
BP	GO:0009225	nucleotide-sugar metabolic process	16/4071	37/18614	0.00292878	0.03279667	0.02665004	16
BP	GO:0031076	embryonic camera-type eye development	16/4071	37/18614	0.00292878	0.03279667	0.02665004	16
BP	GO:0035909	aorta morphogenesis	16/4071	37/18614	0.00292878	0.03279667	0.02665004	16
BP	GO:0009408	response to heat	37/4071	110/18614	0.00293187	0.03279667	0.02665004	37
BP	GO:0014812	muscle cell migration	37/4071	110/18614	0.00293187	0.03279667	0.02665004	37
BP	GO:0008406	gonad development	68/4071	228/18614	0.00293843	0.03283792	0.02668355	68
BP	GO:0050803	regulation of synapse structure or activity	71/4071	240/18614	0.00302296	0.03374212	0.0274183	71
BP	GO:0009791	post-embryonic development	32/4071	92/18614	0.00302526	0.03374212	0.0274183	32
BP	GO:0000302	response to reactive oxygen species	62/4071	205/18614	0.00307415	0.03425397	0.02783421	62
BP	GO:0060538	skeletal muscle organ development	55/4071	178/18614	0.00308081	0.03429467	0.02786729	55
BP	GO:0007566	embryo implantation	22/4071	57/18614	0.00308758	0.03433649	0.02790126	22

BP	GO:0043372	positive regulation of CD4-positive, alpha-beta T cell differentiation	15/4071	34/18614	0.00310456	0.03435794	0.0279187	15
BP	GO:0044546	NLRP3 inflammasome complex assembly	15/4071	34/18614	0.00310456	0.03435794	0.0279187	15
BP	GO:0050869	negative regulation of B cell activation	15/4071	34/18614	0.00310456	0.03435794	0.0279187	15
BP	GO:0060317	cardiac epithelial to mesenchymal transition	15/4071	34/18614	0.00310456	0.03435794	0.0279187	15
BP	GO:2000515	negative regulation of CD4-positive, alpha-beta T cell activation	15/4071	34/18614	0.00310456	0.03435794	0.0279187	15
BP	GO:0060828	regulation of canonical Wnt signaling pathway	76/4071	260/18614	0.00312655	0.03456779	0.02808922	76
BP	GO:0035050	embryonic heart tube development	30/4071	85/18614	0.00312987	0.03457102	0.02809184	30
BP	GO:0046633	alpha-beta T cell proliferation	19/4071	47/18614	0.00315088	0.03470226	0.02819849	19
BP	GO:0061383	trabecula morphogenesis	19/4071	47/18614	0.00315088	0.03470226	0.02819849	19
BP	GO:0070849	response to epidermal growth factor	19/4071	47/18614	0.00315088	0.03470226	0.02819849	19
BP	GO:0002755	MyD88-dependent toll-like receptor signaling pathway	10/4071	19/18614	0.00319367	0.03510577	0.02852637	10
BP	GO:0030903	notochord development	10/4071	19/18614	0.00319367	0.03510577	0.02852637	10
BP	GO:0050918	positive chemotaxis	24/4071	64/18614	0.00319841	0.035124	0.02854119	24
BP	GO:0070373	negative regulation of ERK1 and ERK2 cascade	28/4071	78/18614	0.00320249	0.03513496	0.02855009	28
BP	GO:0051100	negative regulation of binding	51/4071	163/18614	0.0032087	0.0351693	0.02857799	51
BP	GO:0003208	cardiac ventricle morphogenesis	26/4071	71/18614	0.00323051	0.03537442	0.02874467	26
BP	GO:0001945	lymph vessel development	14/4071	31/18614	0.00325534	0.03557795	0.02891006	14
BP	GO:0016556	mRNA modification	14/4071	31/18614	0.00325534	0.03557795	0.02891006	14
BP	GO:0003279	cardiac septum development	36/4071	107/18614	0.00329604	0.03598831	0.02924351	36
BP	GO:0061136	regulation of proteasomal protein catabolic process	59/4071	194/18614	0.00332489	0.03626864	0.02947131	59
BP	GO:0051403	stress-activated MAPK cascade	70/4071	237/18614	0.00335976	0.03639854	0.02957686	70
BP	GO:0003272	endocardial cushion formation	13/4071	28/18614	0.00336497	0.03639854	0.02957686	13
BP	GO:0010758	regulation of macrophage chemotaxis	13/4071	28/18614	0.00336497	0.03639854	0.02957686	13
BP	GO:0031664	regulation of lipopolysaccharide-mediated signaling pathway	13/4071	28/18614	0.00336497	0.03639854	0.02957686	13

BP	GO:0036037	CD8-positive, alpha-beta T cell activation	13/4071	28/18614	0.00336497	0.03639854	0.02957686	13
BP	GO:0150146	cell junction disassembly	13/4071	28/18614	0.00336497	0.03639854	0.02957686	13
BP	GO:0010042	response to manganese ion	11/4071	22/18614	0.00336671	0.03639854	0.02957686	11
BP	GO:2001044	regulation of integrin-mediated signaling pathway	11/4071	22/18614	0.00336671	0.03639854	0.02957686	11
BP	GO:0034975	protein folding in endoplasmic reticulum	7/4071	11/18614	0.003386	0.03639854	0.02957686	7
BP	GO:0036490	regulation of translation in response to endoplasmic reticulum stress	7/4071	11/18614	0.003386	0.03639854	0.02957686	7
BP	GO:0045628	regulation of T-helper 2 cell differentiation	7/4071	11/18614	0.003386	0.03639854	0.02957686	7
BP	GO:0070934	CRD-mediated mRNA stabilization	7/4071	11/18614	0.003386	0.03639854	0.02957686	7
BP	GO:0071609	chemokine (C-C motif) ligand 5 production	7/4071	11/18614	0.003386	0.03639854	0.02957686	7
BP	GO:0071649	regulation of chemokine (C-C motif) ligand 5 production	7/4071	11/18614	0.003386	0.03639854	0.02957686	7
BP	GO:0032945	negative regulation of mononuclear cell proliferation	31/4071	89/18614	0.00339827	0.03639854	0.02957686	31
BP	GO:0071229	cellular response to acid chemical	31/4071	89/18614	0.00339827	0.03639854	0.02957686	31
BP	GO:0034614	cellular response to reactive oxygen species	48/4071	152/18614	0.00339944	0.03639854	0.02957686	48
BP	GO:0033674	positive regulation of kinase activity	118/4071	430/18614	0.00340138	0.03639854	0.02957686	118
BP	GO:0045649	regulation of macrophage differentiation	12/4071	25/18614	0.00341167	0.03639854	0.02957686	12
BP	GO:0046697	decidualization	12/4071	25/18614	0.00341167	0.03639854	0.02957686	12
BP	GO:0060706	cell differentiation involved in embryonic placenta development	12/4071	25/18614	0.00341167	0.03639854	0.02957686	12
BP	GO:0030857	negative regulation of epithelial cell differentiation	21/4071	54/18614	0.00341655	0.03639854	0.02957686	21
BP	GO:0038093	Fc receptor signaling pathway	21/4071	54/18614	0.00341655	0.03639854	0.02957686	21
BP	GO:0071385	cellular response to glucocorticoid stimulus	21/4071	54/18614	0.00341655	0.03639854	0.02957686	21
BP	GO:2000179	positive regulation of neural precursor cell proliferation	21/4071	54/18614	0.00341655	0.03639854	0.02957686	21

BP	GO:0006509	membrane protein ectodomain proteolysis	18/4071	44/18614	0.00343949	0.03654059	0.02969228	18
BP	GO:1905521	regulation of macrophage migration	18/4071	44/18614	0.00343949	0.03654059	0.02969228	18
BP	GO:2000516	positive regulation of CD4-positive, alpha-beta T cell activation	18/4071	44/18614	0.00343949	0.03654059	0.02969228	18
BP	GO:0003151	outflow tract morphogenesis	29/4071	82/18614	0.00351274	0.03724938	0.03026823	29
BP	GO:0014032	neural crest cell development	29/4071	82/18614	0.00351274	0.03724938	0.03026823	29
BP	GO:0051653	spindle localization	23/4071	61/18614	0.00356157	0.03773213	0.03066051	23
BP	GO:0043409	negative regulation of MAPK cascade	56/4071	183/18614	0.00358592	0.03795489	0.03084152	56
BP	GO:0051781	positive regulation of cell division	32/4071	93/18614	0.00366158	0.03871979	0.03146306	32
BP	GO:0050807	regulation of synapse organization	69/4071	234/18614	0.00373361	0.03940837	0.0320226	69
BP	GO:2000116	regulation of cysteine-type endopeptidase activity	69/4071	234/18614	0.00373361	0.03940837	0.0320226	69
BP	GO:0044818	mitotic G2/M transition checkpoint	20/4071	51/18614	0.00377447	0.03976615	0.03231332	20
BP	GO:2001238	positive regulation of extrinsic apoptotic signaling pathway	20/4071	51/18614	0.00377447	0.03976615	0.03231332	20
BP	GO:0051592	response to calcium ion	47/4071	149/18614	0.00381166	0.04012088	0.03260157	47
BP	GO:0048041	focal adhesion assembly	30/4071	86/18614	0.00381802	0.04015078	0.03262586	30
BP	GO:0060993	kidney morphogenesis	33/4071	97/18614	0.00391873	0.04110544	0.0334016	33
BP	GO:1902807	negative regulation of cell cycle G1/S phase transition	33/4071	97/18614	0.00391873	0.04110544	0.0334016	33
BP	GO:0014013	regulation of gliogenesis	36/4071	108/18614	0.00392164	0.04110544	0.0334016	36
BP	GO:0044703	multi-organism reproductive process	62/4071	207/18614	0.00392321	0.04110544	0.0334016	62
BP	GO:0099024	plasma membrane invagination	22/4071	58/18614	0.003963	0.0414843	0.03370946	22
BP	GO:0002260	lymphocyte homeostasis	26/4071	72/18614	0.00402179	0.04195106	0.03408874	26
BP	GO:0006024	glycosaminoglycan biosynthetic process	26/4071	72/18614	0.00402179	0.04195106	0.03408874	26
BP	GO:1900046	regulation of hemostasis	26/4071	72/18614	0.00402179	0.04195106	0.03408874	26
BP	GO:0006413	translational initiation	40/4071	123/18614	0.00402598	0.04195106	0.03408874	40
BP	GO:0022612	gland morphogenesis	40/4071	123/18614	0.00402598	0.04195106	0.03408874	40
BP	GO:0010324	membrane invagination	24/4071	65/18614	0.00403618	0.04201904	0.03414398	24
BP	GO:0002335	mature B cell differentiation	16/4071	38/18614	0.00404528	0.04207537	0.03418976	16
BP	GO:0021543	pallium development	56/4071	184/18614	0.00407732	0.04236994	0.03442912	56
BP	GO:2000106	regulation of leukocyte apoptotic process	31/4071	90/18614	0.00411682	0.04271493	0.03470945	31

BP	GO:0046777	protein autophosphorylation	67/4071	227/18614	0.004118	0.04271493	0.03470945	67
BP	GO:0038061	NIK/NF-kappaB signaling	45/4071	142/18614	0.0041475	0.04298178	0.03492629	45
BP	GO:0048538	thymus development	19/4071	48/18614	0.00416178	0.04309063	0.03501474	19
BP	GO:0070167	regulation of biomineral tissue development	34/4071	101/18614	0.00416884	0.04312466	0.03504239	34
BP	GO:0071482	cellular response to light stimulus	41/4071	127/18614	0.00419291	0.04333428	0.03521273	41
BP	GO:0031330	negative regulation of cellular catabolic process	73/4071	251/18614	0.00426323	0.04399644	0.03575079	73
BP	GO:0070302	regulation of stress-activated protein kinase signaling cascade	59/4071	196/18614	0.00426469	0.04399644	0.03575079	59
BP	GO:0034340	response to type I interferon	29/4071	83/18614	0.00429048	0.04422255	0.03593452	29
BP	GO:1904029	regulation of cyclin-dependent protein kinase activity	38/4071	116/18614	0.00433137	0.04448679	0.03614924	38
BP	GO:0007100	mitotic centrosome separation	8/4071	14/18614	0.00433171	0.04448679	0.03614924	8
BP	GO:0018158	protein oxidation	8/4071	14/18614	0.00433171	0.04448679	0.03614924	8
BP	GO:0070816	phosphorylation of RNA polymerase II C-terminal domain	8/4071	14/18614	0.00433171	0.04448679	0.03614924	8
BP	GO:0031128	developmental induction	15/4071	35/18614	0.00434861	0.0446202	0.03625764	15
BP	GO:0071621	granulocyte chemotaxis	42/4071	131/18614	0.00435322	0.04462735	0.03626345	42
BP	GO:2000377	regulation of reactive oxygen species metabolic process	47/4071	150/18614	0.00439545	0.04501983	0.03658237	47
BP	GO:1900407	regulation of cellular response to oxidative stress	32/4071	94/18614	0.00440792	0.04510055	0.03664797	32
BP	GO:0070828	heterochromatin organization	35/4071	105/18614	0.00441124	0.04510055	0.03664797	35
BP	GO:1901654	response to ketone	62/4071	208/18614	0.00441883	0.04513777	0.03667821	62
BP	GO:1903036	positive regulation of response to wounding	27/4071	76/18614	0.0044263	0.04517355	0.03670729	27
BP	GO:0032835	glomerulus development	25/4071	69/18614	0.00450676	0.04589793	0.0372959	25
BP	GO:0090303	positive regulation of wound healing	23/4071	62/18614	0.00450934	0.04589793	0.0372959	23
BP	GO:1904036	negative regulation of epithelial cell apoptotic process	23/4071	62/18614	0.00450934	0.04589793	0.0372959	23
BP	GO:0071774	response to fibroblast growth factor	39/4071	120/18614	0.00452516	0.0460179	0.03739339	39
BP	GO:0023061	signal release	130/4071	483/18614	0.0045571	0.04630142	0.03762377	130
BP	GO:0032092	positive regulation of protein binding	30/4071	87/18614	0.00462983	0.04690454	0.03811386	30

BP	GO:0008156	negative regulation of DNA replication	14/4071	32/18614	0.00463701	0.04690454	0.03811386	14
BP	GO:0035115	embryonic forelimb morphogenesis	14/4071	32/18614	0.00463701	0.04690454	0.03811386	14
BP	GO:0046685	response to arsenic-containing substance	14/4071	32/18614	0.00463701	0.04690454	0.03811386	14
BP	GO:0061037	negative regulation of cartilage development	14/4071	32/18614	0.00463701	0.04690454	0.03811386	14
BP	GO:0000209	protein polyubiquitination	71/4071	244/18614	0.00470774	0.04757776	0.03866091	71
BP	GO:0050767	regulation of neurogenesis	105/4071	381/18614	0.00477862	0.04825135	0.03920826	105
BP	GO:0071230	cellular response to amino acid stimulus	28/4071	80/18614	0.00482242	0.0486506	0.03953268	28
BP	GO:0022411	cellular component disassembly	131/4071	488/18614	0.00486344	0.04877778	0.03963602	131
BP	GO:0002730	regulation of dendritic cell cytokine production	9/4071	17/18614	0.00488851	0.04877778	0.03963602	9
BP	GO:0034112	positive regulation of homotypic cell-cell adhesion	9/4071	17/18614	0.00488851	0.04877778	0.03963602	9
BP	GO:0044320	cellular response to leptin stimulus	9/4071	17/18614	0.00488851	0.04877778	0.03963602	9
BP	GO:1901970	positive regulation of mitotic sister chromatid separation	9/4071	17/18614	0.00488851	0.04877778	0.03963602	9
BP	GO:1902036	regulation of hematopoietic stem cell differentiation	9/4071	17/18614	0.00488851	0.04877778	0.03963602	9
BP	GO:0003156	regulation of animal organ formation	13/4071	29/18614	0.00489244	0.04877778	0.03963602	13
BP	GO:0003209	cardiac atrium morphogenesis	13/4071	29/18614	0.00489244	0.04877778	0.03963602	13
BP	GO:0060441	epithelial tube branching involved in lung morphogenesis	13/4071	29/18614	0.00489244	0.04877778	0.03963602	13
BP	GO:0072012	glomerulus vasculature development	13/4071	29/18614	0.00489244	0.04877778	0.03963602	13
BP	GO:0048013	ephrin receptor signaling pathway	20/4071	52/18614	0.00489264	0.04877778	0.03963602	20
BP	GO:0072599	establishment of protein localization to endoplasmic reticulum	20/4071	52/18614	0.00489264	0.04877778	0.03963602	20
BP	GO:0050727	regulation of inflammatory response	113/4071	414/18614	0.00489906	0.04877778	0.03963602	113
BP	GO:0045667	regulation of osteoblast differentiation	46/4071	147/18614	0.00492722	0.04877778	0.03963602	46
BP	GO:0002317	plasma cell differentiation	6/4071	9/18614	0.00493763	0.04877778	0.03963602	6

BP	GO:0002840	regulation of T cell mediated immune response to tumor cell	6/4071	9/18614	0.00493763	0.04877778	0.03963602	6
BP	GO:0006048	UDP-N-acetylglucosamine biosynthetic process	6/4071	9/18614	0.00493763	0.04877778	0.03963602	6
BP	GO:0006930	substrate-dependent cell migration, cell extension	6/4071	9/18614	0.00493763	0.04877778	0.03963602	6
BP	GO:0034139	regulation of toll-like receptor 3 signaling pathway	6/4071	9/18614	0.00493763	0.04877778	0.03963602	6
BP	GO:0062043	positive regulation of cardiac epithelial to mesenchymal transition	6/4071	9/18614	0.00493763	0.04877778	0.03963602	6
BP	GO:0070672	response to interleukin-15	6/4071	9/18614	0.00493763	0.04877778	0.03963602	6
BP	GO:0071281	cellular response to iron ion	6/4071	9/18614	0.00493763	0.04877778	0.03963602	6
BP	GO:1902746	regulation of lens fiber cell differentiation	6/4071	9/18614	0.00493763	0.04877778	0.03963602	6
BP	GO:2001187	positive regulation of CD8-positive, alpha-beta T cell activation	6/4071	9/18614	0.00493763	0.04877778	0.03963602	6
BP	GO:0061035	regulation of cartilage development	26/4071	73/18614	0.00496953	0.04905042	0.03985757	26
BP	GO:0032733	positive regulation of interleukin-10 production	17/4071	42/18614	0.00502112	0.04951685	0.04023658	17
BP	GO:0001913	T cell mediated cytotoxicity	22/4071	59/18614	0.00503586	0.04957649	0.04028504	22
BP	GO:1903053	regulation of extracellular matrix organization	22/4071	59/18614	0.00503586	0.04957649	0.04028504	22
BP	GO:0031100	animal organ regeneration	24/4071	66/18614	0.00504986	0.0496287	0.04032747	24
BP	GO:0060675	ureteric bud morphogenesis	24/4071	66/18614	0.00504986	0.0496287	0.04032747	24
BP	GO:0036119	response to platelet-derived growth factor	12/4071	26/18614	0.00508907	0.0496718	0.04036249	12
BP	GO:0044346	fibroblast apoptotic process	12/4071	26/18614	0.00508907	0.0496718	0.04036249	12
BP	GO:0048745	smooth muscle tissue development	12/4071	26/18614	0.00508907	0.0496718	0.04036249	12
BP	GO:0060575	intestinal epithelial cell differentiation	12/4071	26/18614	0.00508907	0.0496718	0.04036249	12
BP	GO:0062009	secondary palate development	12/4071	26/18614	0.00508907	0.0496718	0.04036249	12
BP	GO:0070102	interleukin-6-mediated signaling pathway	12/4071	26/18614	0.00508907	0.0496718	0.04036249	12
BP	GO:1900151	regulation of nuclear-transcribed mRNA catabolic process,	12/4071	26/18614	0.00508907	0.0496718	0.04036249	12

		deadenylation-dependent decay						
BP	GO:1903306	negative regulation of regulated secretory pathway	12/4071	26/18614	0.00508907	0.0496718	0.04036249	12
CC	GO:0005925	focal adhesion	180/4225	422/19518	1.11E-22	8.79E-20	7.32E-20	180
CC	GO:0030055	cell-substrate junction	183/4225	432/19518	1.25E-22	8.79E-20	7.32E-20	183
CC	GO:0000775	chromosome, centromeric region	104/4225	249/19518	5.65E-13	2.65E-10	2.21E-10	104
CC	GO:0098687	chromosomal region	144/4225	390/19518	2.69E-12	9.48E-10	7.89E-10	144
CC	GO:0005819	spindle	152/4225	421/19518	5.04E-12	1.42E-09	1.18E-09	152
CC	GO:0000793	condensed chromosome	107/4225	270/19518	1.33E-11	2.88E-09	2.40E-09	107
CC	GO:0000779	condensed chromosome, centromeric region	76/4225	170/19518	1.43E-11	2.88E-09	2.40E-09	76
CC	GO:0062023	collagen-containing extracellular matrix	147/4225	415/19518	5.66E-11	9.97E-09	8.30E-09	147
CC	GO:0005788	endoplasmic reticulum lumen	117/4225	312/19518	9.76E-11	1.53E-08	1.27E-08	117
CC	GO:0000776	kinetochore	70/4225	159/19518	2.09E-10	2.95E-08	2.45E-08	70
CC	GO:0090575	RNA polymerase II transcription regulator complex	98/4225	254/19518	5.41E-10	6.94E-08	5.77E-08	98
CC	GO:0030667	secretory granule membrane	115/4225	319/19518	2.15E-09	2.52E-07	2.10E-07	115
CC	GO:0000228	nuclear chromosome	87/4225	223/19518	2.63E-09	2.85E-07	2.37E-07	87
CC	GO:0072686	mitotic spindle	73/4225	180/19518	7.12E-09	7.17E-07	5.97E-07	73
CC	GO:0005769	early endosome	140/4225	423/19518	2.52E-08	2.37E-06	1.97E-06	140
CC	GO:0034774	secretory granule lumen	112/4225	322/19518	3.33E-08	2.93E-06	2.44E-06	112
CC	GO:0016607	nuclear speck	137/4225	415/19518	4.23E-08	3.39E-06	2.82E-06	137
CC	GO:0031983	vesicle lumen	113/4225	327/19518	4.32E-08	3.39E-06	2.82E-06	113
CC	GO:0098857	membrane microdomain	112/4225	324/19518	4.86E-08	3.60E-06	3.00E-06	112
CC	GO:0060205	cytoplasmic vesicle lumen	112/4225	325/19518	5.85E-08	4.12E-06	3.43E-06	112
CC	GO:0045121	membrane raft	111/4225	323/19518	7.90E-08	5.31E-06	4.42E-06	111
CC	GO:0031252	cell leading edge	137/4225	422/19518	1.29E-07	8.27E-06	6.89E-06	137
CC	GO:0000922	spindle pole	67/4225	171/19518	1.40E-07	8.58E-06	7.14E-06	67
CC	GO:0051233	spindle midzone	22/4225	36/19518	3.47E-07	2.04E-05	1.70E-05	22
CC	GO:0042470	melanosome	47/4225	112/19518	1.02E-06	5.53E-05	4.60E-05	47
CC	GO:0048770	pigment granule	47/4225	112/19518	1.02E-06	5.53E-05	4.60E-05	47
CC	GO:0030139	endocytic vesicle	113/4225	347/19518	1.34E-06	6.98E-05	5.81E-05	113
CC	GO:1904813	ficolin-1-rich granule lumen	50/4225	124/19518	1.93E-06	9.74E-05	8.11E-05	50
CC	GO:0000940	outer kinetochore	11/4225	13/19518	2.43E-06	0.00011811	9.83E-05	11
CC	GO:0005604	basement membrane	37/4225	84/19518	3.57E-06	0.00016785	0.00013972	37
CC	GO:0017053	transcription repressor complex	35/4225	78/19518	3.89E-06	0.00017684	0.0001472	35
CC	GO:0016363	nuclear matrix	50/4225	127/19518	4.37E-06	0.00019263	0.00016034	50
CC	GO:0030135	coated vesicle	101/4225	312/19518	6.35E-06	0.00027113	0.00022569	101
CC	GO:0101002	ficolin-1-rich granule	66/4225	185/19518	7.97E-06	0.00033066	0.00027524	66

CC	GO:0070820	tertiary granule	60/4225	164/19518	8.28E-06	0.00033368	0.00027776	60
CC	GO:0030133	transport vesicle	130/4225	425/19518	8.68E-06	0.00033978	0.00028283	130
CC	GO:0034399	nuclear periphery	55/4225	147/19518	9.09E-06	0.00034621	0.00028819	55
CC	GO:0031091	platelet alpha granule	38/4225	91/19518	1.22E-05	0.00045434	0.00037819	38
CC	GO:0030027	lamellipodium	70/4225	202/19518	1.35E-05	0.00048678	0.0004052	70
CC	GO:0005774	vacuolar membrane	138/4225	460/19518	1.39E-05	0.00049121	0.00040889	138
CC	GO:0001726	ruffle	64/4225	181/19518	1.50E-05	0.00051689	0.00043026	64
CC	GO:0030496	midbody	70/4225	203/19518	1.62E-05	0.00054553	0.0004541	70
CC	GO:0030666	endocytic vesicle membrane	68/4225	196/19518	1.70E-05	0.00055713	0.00046376	68
CC	GO:0031901	early endosome membrane	65/4225	187/19518	2.41E-05	0.00077182	0.00064246	65
CC	GO:0045335	phagocytic vesicle	52/4225	141/19518	2.50E-05	0.00078198	0.00065092	52
CC	GO:0042581	specific granule	57/4225	160/19518	3.35E-05	0.00102785	0.00085558	57
CC	GO:0009897	external side of plasma membrane	127/4225	426/19518	4.13E-05	0.00121898	0.00101468	127
CC	GO:0005657	replication fork	29/4225	66/19518	4.15E-05	0.00121898	0.00101468	29
CC	GO:0005765	lysosomal membrane	124/4225	415/19518	4.53E-05	0.00127809	0.00106389	124
CC	GO:0098852	lytic vacuole membrane	124/4225	415/19518	4.53E-05	0.00127809	0.00106389	124
CC	GO:0005681	spliceosomal complex	66/4225	197/19518	7.64E-05	0.00211207	0.00175809	66
CC	GO:0000792	heterochromatin	33/4225	81/19518	8.03E-05	0.00213667	0.00177856	33
CC	GO:0005876	spindle microtubule	33/4225	81/19518	8.03E-05	0.00213667	0.00177856	33
CC	GO:0005775	vacuolar lumen	60/4225	176/19518	9.12E-05	0.00238135	0.00198224	60
CC	GO:0140534	endoplasmic reticulum protein-containing complex	46/4225	126/19518	9.45E-05	0.00242198	0.00201606	46
CC	GO:0005766	primary lysosome	54/4225	155/19518	0.00010522	0.00260285	0.00216661	54
CC	GO:0042582	azurophil granule	54/4225	155/19518	0.00010522	0.00260285	0.00216661	54
CC	GO:0030134	COPII-coated ER to Golgi transport vesicle	36/4225	93/19518	0.00013665	0.00332209	0.00276531	36
CC	GO:0000796	condensin complex	7/4225	8/19518	0.0001439	0.00343905	0.00286266	7
CC	GO:0001650	fibrillar center	51/4225	146/19518	0.00014943	0.00347174	0.00288987	51
CC	GO:0032154	cleavage furrow	24/4225	54/19518	0.0001502	0.00347174	0.00288987	24
CC	GO:0042613	MHC class II protein complex	11/4225	17/19518	0.00016041	0.00362688	0.00301902	11
CC	GO:0031093	platelet alpha granule lumen	28/4225	67/19518	0.00016205	0.00362688	0.00301902	28
CC	GO:0030662	coated vesicle membrane	66/4225	202/19518	0.00017554	0.00386744	0.00321926	66
CC	GO:0042611	MHC protein complex	14/4225	25/19518	0.0001859	0.00403258	0.00335672	14
CC	GO:0035770	ribonucleoprotein granule	84/4225	271/19518	0.00019452	0.0041557	0.00345921	84
CC	GO:0035861	site of double-strand break	31/4225	78/19518	0.00022299	0.00469279	0.00390628	31
CC	GO:0036464	cytoplasmic ribonucleoprotein granule	79/4225	253/19518	0.00022908	0.00475004	0.00395393	79
CC	GO:0001772	immunological synapse	20/4225	43/19518	0.00024929	0.0050942	0.00424041	20
CC	GO:0090734	site of DNA damage	40/4225	110/19518	0.00028782	0.00579757	0.0048259	40
CC	GO:0043596	nuclear replication fork	17/4225	35/19518	0.00038066	0.00755956	0.00629258	17
CC	GO:0030175	filopodium	39/4225	108/19518	0.00040051	0.00781479	0.00650503	39

CC	GO:0098636	protein complex involved in cell adhesion	24/4225	57/19518	0.00040696	0.00781479	0.00650503	24
CC	GO:0071162	CMG complex	8/4225	11/19518	0.00041812	0.00781479	0.00650503	8
CC	GO:1904090	peptidase inhibitor complex	8/4225	11/19518	0.00041812	0.00781479	0.00650503	8
CC	GO:0098644	complex of collagen trimers	12/4225	21/19518	0.00042122	0.00781479	0.00650503	12
CC	GO:0005798	Golgi-associated vesicle	34/4225	91/19518	0.00044945	0.00823026	0.00685087	34
CC	GO:0030670	phagocytic vesicle membrane	30/4225	78/19518	0.00054138	0.00972425	0.00809446	30
CC	GO:0035577	azurophil granule membrane	24/4225	58/19518	0.00055314	0.00972425	0.00809446	24
CC	GO:0005581	collagen trimer	32/4225	85/19518	0.00055929	0.00972425	0.00809446	32
CC	GO:0002102	podosome	15/4225	30/19518	0.00056552	0.00972425	0.00809446	15
CC	GO:0005721	pericentric heterochromatin	15/4225	30/19518	0.00056552	0.00972425	0.00809446	15
CC	GO:1990023	mitotic spindle midzone	9/4225	14/19518	0.00070799	0.01202724	0.01001148	9
CC	GO:0005635	nuclear envelope	136/4225	490/19518	0.00072368	0.01214756	0.01011163	136
CC	GO:0031258	lamellipodium membrane	12/4225	22/19518	0.00074464	0.01235234	0.01028209	12
CC	GO:0030669	clathrin-coated endocytic vesicle membrane	28/4225	73/19518	0.00085905	0.01392257	0.01158915	28
CC	GO:0070821	tertiary granule membrane	28/4225	73/19518	0.00085905	0.01392257	0.01158915	28
CC	GO:0012507	ER to Golgi transport vesicle membrane	25/4225	63/19518	0.00089692	0.01437104	0.01196245	25
CC	GO:0015629	actin cytoskeleton	135/4225	489/19518	0.00094955	0.01504336	0.0125221	135
CC	GO:0005583	fibrillar collagen trimer	8/4225	12/19518	0.00101556	0.01556455	0.01295593	8
CC	GO:0031261	DNA replication preinitiation complex	8/4225	12/19518	0.00101556	0.01556455	0.01295593	8
CC	GO:0098643	banded collagen fibril	8/4225	12/19518	0.00101556	0.01556455	0.01295593	8
CC	GO:0005793	endoplasmic reticulum-Golgi intermediate compartment	44/4225	132/19518	0.00124496	0.01873456	0.01559465	44
CC	GO:0010494	cytoplasmic stress granule	31/4225	85/19518	0.00124897	0.01873456	0.01559465	31
CC	GO:0005802	trans-Golgi network	76/4225	255/19518	0.00132948	0.01973227	0.01642515	76
CC	GO:0071735	IgG immunoglobulin complex	7/4225	10/19518	0.00142284	0.02089795	0.01739546	7
CC	GO:0030140	trans-Golgi network transport vesicle	17/4225	39/19518	0.00175327	0.02548564	0.02121425	17
CC	GO:0030658	transport vesicle membrane	70/4225	234/19518	0.00180696	0.02599812	0.02164084	70
CC	GO:0031371	ubiquitin conjugating enzyme complex	6/4225	8/19518	0.00190861	0.026645	0.0221793	6
CC	GO:0042555	MCM complex	6/4225	8/19518	0.00190861	0.026645	0.0221793	6
CC	GO:0097179	protease inhibitor complex	6/4225	8/19518	0.00190861	0.026645	0.0221793	6

CC	GO:0061695	transferase complex, transferring phosphorus-containing groups	88/4225	306/19518	0.00193638	0.02676765	0.0222814	88
CC	GO:0000932	P-body	34/4225	98/19518	0.0020069	0.027209	0.02264877	34
CC	GO:0043202	lysosomal lumen	34/4225	98/19518	0.0020069	0.027209	0.02264877	34
CC	GO:0035579	specific granule membrane	32/4225	91/19518	0.00209373	0.02811578	0.02340358	32
CC	GO:0000151	ubiquitin ligase complex	88/4225	307/19518	0.0021477	0.02837101	0.02361603	88
CC	GO:0008623	CHRAC	4/4225	4/19518	0.00219322	0.02837101	0.02361603	4
CC	GO:0033597	mitotic checkpoint complex	4/4225	4/19518	0.00219322	0.02837101	0.02361603	4
CC	GO:0070532	BRCA1-B complex	4/4225	4/19518	0.00219322	0.02837101	0.02361603	4
CC	GO:0032133	chromosome passenger complex	5/4225	6/19518	0.00233345	0.02991061	0.0248976	5
CC	GO:0000307	cyclin-dependent protein kinase holoenzyme complex	20/4225	50/19518	0.00253298	0.03216078	0.02677064	20
CC	GO:0045334	clathrin-coated endocytic vesicle	32/4225	92/19518	0.00255462	0.03216078	0.02677064	32
CC	GO:0000803	sex chromosome	14/4225	31/19518	0.00294379	0.0367322	0.03057589	14
CC	GO:1990391	DNA repair complex	11/4225	22/19518	0.00309056	0.03822539	0.03181882	11
CC	GO:0030136	clathrin-coated vesicle	64/4225	215/19518	0.00313087	0.03838723	0.03195354	64
CC	GO:0034663	endoplasmic reticulum chaperone complex	7/4225	11/19518	0.0031811	0.03866678	0.03218623	7
CC	GO:0097431	mitotic spindle pole	16/4225	38/19518	0.00363119	0.04376044	0.0364262	16
CC	GO:1904724	tertiary granule lumen	21/4225	55/19518	0.00387944	0.04635602	0.03858675	21
CC	GO:0035580	specific granule lumen	23/4225	62/19518	0.00394275	0.04671661	0.03888692	23
CC	GO:0008250	oligosaccharyltransferase complex	8/4225	14/19518	0.00404802	0.04717109	0.03926522	8
CC	GO:0071564	npBAF complex	8/4225	14/19518	0.00404802	0.04717109	0.03926522	8
CC	GO:0032153	cell division site	26/4225	73/19518	0.0043052	0.0497568	0.04141757	26
MF	GO:0001228	DNA-binding transcription activator activity, RNA polymerase II-specific	177/4252	468/18369	3.82E-13	6.24E-10	5.96E-10	177
MF	GO:0001216	DNA-binding transcription activator activity	178/4252	472/18369	4.41E-13	6.24E-10	5.96E-10	178
MF	GO:0019003	GDP binding	42/4252	73/18369	2.60E-10	2.46E-07	2.35E-07	42
MF	GO:0019838	growth factor binding	59/4252	132/18369	3.59E-08	2.55E-05	2.43E-05	59
MF	GO:0005201	extracellular matrix structural constituent	70/4252	167/18369	5.01E-08	2.84E-05	2.71E-05	70
MF	GO:0003697	single-stranded DNA binding	53/4252	119/18369	2.01E-07	9.50E-05	9.07E-05	53
MF	GO:0003725	double-stranded RNA binding	35/4252	70/18369	8.50E-07	0.00030481	0.00029096	35
MF	GO:0003924	GTPase activity	114/4252	327/18369	8.60E-07	0.00030481	0.00029096	114
MF	GO:0005518	collagen binding	33/4252	66/18369	1.74E-06	0.00054679	0.00052195	33
MF	GO:0005525	GTP binding	128/4252	382/18369	2.09E-06	0.0005494	0.00052445	128

MF	GO:0003925	G protein activity	24/4252	42/18369	2.13E-06	0.0005494	0.00052445	24
MF	GO:0140297	DNA-binding transcription factor binding	154/4252	478/18369	2.75E-06	0.0006504	0.00062085	154
MF	GO:0045296	cadherin binding	112/4252	333/18369	7.29E-06	0.00158869	0.00151653	112
MF	GO:0044389	ubiquitin-like protein ligase binding	108/4252	320/18369	8.86E-06	0.00179319	0.00171173	108
MF	GO:0019001	guanyl nucleotide binding	130/4252	404/18369	1.71E-05	0.0030259	0.00288845	130
MF	GO:0032561	guanyl ribonucleotide binding	130/4252	404/18369	1.71E-05	0.0030259	0.00288845	130
MF	GO:0061629	RNA polymerase II-specific DNA-binding transcription factor binding	114/4252	348/18369	2.31E-05	0.00385652	0.00368134	114
MF	GO:0031625	ubiquitin protein ligase binding	100/4252	301/18369	3.83E-05	0.00603396	0.00575986	100
MF	GO:0019956	chemokine binding	14/4252	22/18369	5.78E-05	0.00861732	0.00822587	14
MF	GO:0140097	catalytic activity, acting on DNA	83/4252	245/18369	7.93E-05	0.01123831	0.0107278	83
MF	GO:0043394	proteoglycan binding	19/4252	36/18369	0.0001078	0.01454798	0.01388712	19
MF	GO:0008094	ATP-dependent activity, acting on DNA	48/4252	126/18369	0.00011327	0.01459175	0.01392891	48
MF	GO:0019955	cytokine binding	49/4252	130/18369	0.0001313	0.01617809	0.01544319	49
MF	GO:0015026	coreceptor activity	23/4252	48/18369	0.00014587	0.01722525	0.01644278	23
MF	GO:0035925	mRNA 3'-UTR AU-rich region binding	15/4252	26/18369	0.00015477	0.01754514	0.01674814	15
MF	GO:0051219	phosphoprotein binding	37/4252	92/18369	0.00018741	0.01983268	0.01893176	37
MF	GO:0004860	protein kinase inhibitor activity	20/4252	40/18369	0.00018895	0.01983268	0.01893176	20
MF	GO:0046935	1-phosphatidylinositol-3-kinase regulator activity	12/4252	19/18369	0.00022199	0.02108926	0.02013127	12
MF	GO:0042393	histone binding	80/4252	241/18369	0.00022323	0.02108926	0.02013127	80
MF	GO:0017147	Wnt-protein binding	17/4252	32/18369	0.00022325	0.02108926	0.02013127	17
MF	GO:0019210	kinase inhibitor activity	21/4252	44/18369	0.0003009	0.02750828	0.0262587	21
MF	GO:0140375	immune receptor activity	51/4252	141/18369	0.00031536	0.0279291	0.0266604	51
MF	GO:0035325	Toll-like receptor binding	9/4252	13/18369	0.00053555	0.0455947	0.04352352	9
MF	GO:0017116	single-stranded DNA helicase activity	13/4252	23/18369	0.0005631	0.0455947	0.04352352	13
MF	GO:0050431	transforming growth factor beta binding	13/4252	23/18369	0.0005631	0.0455947	0.04352352	13
MF	GO:0004720	protein-lysine 6-oxidase activity	5/4252	5/18369	0.00066337	0.04820462	0.04601488	5
MF	GO:0038064	collagen receptor activity	5/4252	5/18369	0.00066337	0.04820462	0.04601488	5
MF	GO:0045545	syndecan binding	5/4252	5/18369	0.00066337	0.04820462	0.04601488	5
MF	GO:0045569	TRAIL binding	5/4252	5/18369	0.00066337	0.04820462	0.04601488	5
MF	GO:0048407	platelet-derived growth factor binding	8/4252	11/18369	0.00068043	0.04820819	0.04601829	8

Table S14 GO terms based on DEGs between high and low SEPN1 expression groups in CGGA-325.

ONTOLOGY	ID	Description	GeneRatio	BgRatio	P-value	P-adjust	Q-value	Count
BP	GO:0099177	regulation of trans-synaptic signaling	140/2016	488/18614	2.26E-28	2.08E-24	1.59E-24	140
BP	GO:0050804	modulation of chemical synaptic transmission	139/2016	487/18614	6.27E-28	2.89E-24	2.21E-24	139
BP	GO:0050808	synapse organization	131/2016	466/18614	1.10E-25	2.65E-22	2.03E-22	131
BP	GO:0002443	leukocyte mediated immunity	119/2016	401/18614	1.15E-25	2.65E-22	2.03E-22	119
BP	GO:0030198	extracellular matrix organization	99/2016	314/18614	1.04E-23	1.91E-20	1.46E-20	99
BP	GO:0043062	extracellular structure organization	99/2016	315/18614	1.36E-23	2.04E-20	1.56E-20	99
BP	GO:0034765	regulation of monoatomic ion transmembrane transport	129/2016	478/18614	1.55E-23	2.04E-20	1.56E-20	129
BP	GO:0045229	external encapsulating structure organization	99/2016	317/18614	2.31E-23	2.66E-20	2.04E-20	99
BP	GO:0042391	regulation of membrane potential	120/2016	433/18614	4.99E-23	5.10E-20	3.91E-20	120
BP	GO:0002460	adaptive immune response based on somatic recombination of immune receptors built from immunoglobulin superfamily domains	93/2016	311/18614	1.48E-20	1.36E-17	1.04E-17	93
BP	GO:0002449	lymphocyte mediated immunity	89/2016	300/18614	1.78E-19	1.49E-16	1.14E-16	89
BP	GO:0045785	positive regulation of cell adhesion	119/2016	482/18614	2.04E-18	1.57E-15	1.20E-15	119
BP	GO:0050890	cognition	88/2016	317/18614	2.98E-17	2.11E-14	1.61E-14	88
BP	GO:0006887	exocytosis	94/2016	351/18614	3.20E-17	2.11E-14	1.61E-14	94
BP	GO:0045055	regulated exocytosis	71/2016	228/18614	4.84E-17	2.97E-14	2.27E-14	71
BP	GO:0023061	signal release	116/2016	483/18614	5.19E-17	2.98E-14	2.28E-14	116
BP	GO:0007611	learning or memory	80/2016	276/18614	5.52E-17	2.99E-14	2.29E-14	80
BP	GO:0017157	regulation of exocytosis	64/2016	194/18614	7.10E-17	3.63E-14	2.78E-14	64
BP	GO:0006813	potassium ion transport	73/2016	241/18614	9.45E-17	4.58E-14	3.50E-14	73
BP	GO:0071805	potassium ion transmembrane transport	68/2016	217/18614	1.55E-16	7.14E-14	5.47E-14	68
BP	GO:0031644	regulation of nervous system process	54/2016	150/18614	2.63E-16	1.15E-13	8.83E-14	54
BP	GO:0042060	wound healing	107/2016	439/18614	2.94E-16	1.23E-13	9.41E-14	107
BP	GO:0050867	positive regulation of cell activation	99/2016	394/18614	4.66E-16	1.87E-13	1.43E-13	99
BP	GO:0022407	regulation of cell-cell adhesion	115/2016	491/18614	5.05E-16	1.94E-13	1.48E-13	115
BP	GO:0060078	regulation of postsynaptic membrane potential	53/2016	148/18614	6.44E-16	2.37E-13	1.81E-13	53

BP	GO:0007159	leukocyte cell-cell adhesion	102/2016	415/18614	8.16E-16	2.89E-13	2.21E-13	102
BP	GO:0002274	myeloid leukocyte activation	71/2016	240/18614	9.86E-16	3.36E-13	2.57E-13	71
BP	GO:0048167	regulation of synaptic plasticity	64/2016	209/18614	4.18E-15	1.37E-12	1.05E-12	64
BP	GO:0002696	positive regulation of leukocyte activation	94/2016	377/18614	4.36E-15	1.39E-12	1.06E-12	94
BP	GO:0002697	regulation of immune effector process	95/2016	384/18614	5.26E-15	1.61E-12	1.24E-12	95
BP	GO:0006836	neurotransmitter transport	62/2016	202/18614	9.94E-15	2.95E-12	2.26E-12	62
BP	GO:1903037	regulation of leukocyte cell-cell adhesion	93/2016	378/18614	1.47E-14	4.22E-12	3.23E-12	93
BP	GO:0050900	leukocyte migration	95/2016	393/18614	2.44E-14	6.81E-12	5.21E-12	95
BP	GO:1903039	positive regulation of leukocyte cell-cell adhesion	74/2016	273/18614	3.94E-14	1.07E-11	8.16E-12	74
BP	GO:0022409	positive regulation of cell-cell adhesion	82/2016	321/18614	6.02E-14	1.58E-11	1.21E-11	82
BP	GO:0051251	positive regulation of lymphocyte activation	83/2016	327/18614	6.20E-14	1.59E-11	1.21E-11	83
BP	GO:0001505	regulation of neurotransmitter levels	62/2016	211/18614	9.13E-14	2.23E-11	1.71E-11	62
BP	GO:1902074	response to salt	92/2016	383/18614	9.40E-14	2.23E-11	1.71E-11	92
BP	GO:0050863	regulation of T cell activation	91/2016	377/18614	9.47E-14	2.23E-11	1.71E-11	91
BP	GO:0035249	synaptic transmission, glutamatergic	42/2016	112/18614	1.07E-13	2.45E-11	1.88E-11	42
BP	GO:0030199	collagen fibril organization	30/2016	64/18614	3.59E-13	8.06E-11	6.17E-11	30
BP	GO:0001819	positive regulation of cytokine production	107/2016	489/18614	6.12E-13	1.34E-10	1.03E-10	107
BP	GO:0007612	learning	49/2016	152/18614	8.03E-13	1.72E-10	1.31E-10	49
BP	GO:0070661	leukocyte proliferation	84/2016	348/18614	8.55E-13	1.79E-10	1.37E-10	84
BP	GO:0019882	antigen processing and presentation	41/2016	114/18614	1.05E-12	2.15E-10	1.64E-10	41
BP	GO:0050670	regulation of lymphocyte proliferation	65/2016	239/18614	1.19E-12	2.37E-10	1.82E-10	65
BP	GO:0060079	excitatory postsynaptic potential	43/2016	124/18614	1.25E-12	2.44E-10	1.87E-10	43
BP	GO:1903305	regulation of regulated secretory pathway	44/2016	130/18614	1.78E-12	3.36E-10	2.57E-10	44
BP	GO:0097529	myeloid leukocyte migration	65/2016	241/18614	1.79E-12	3.36E-10	2.57E-10	65
BP	GO:0046651	lymphocyte proliferation	76/2016	306/18614	2.32E-12	4.27E-10	3.26E-10	76
BP	GO:0032944	regulation of mononuclear cell proliferation	65/2016	243/18614	2.68E-12	4.84E-10	3.71E-10	65

BP	GO:0050870	positive regulation of T cell activation	66/2016	249/18614	2.86E-12	5.06E-10	3.88E-10	66
BP	GO:0099565	chemical synaptic transmission, postsynaptic	44/2016	132/18614	3.24E-12	5.44E-10	4.16E-10	44
BP	GO:0002429	immune response-activating cell surface receptor signaling pathway	75/2016	302/18614	3.25E-12	5.44E-10	4.16E-10	75
BP	GO:0002253	activation of immune response	106/2016	495/18614	3.25E-12	5.44E-10	4.16E-10	106
BP	GO:0070663	regulation of leukocyte proliferation	69/2016	268/18614	4.02E-12	6.60E-10	5.05E-10	69
BP	GO:0050803	regulation of synapse structure or activity	64/2016	240/18614	4.58E-12	7.39E-10	5.66E-10	64
BP	GO:0002703	regulation of leukocyte mediated immunity	65/2016	246/18614	4.87E-12	7.73E-10	5.91E-10	65
BP	GO:0035418	protein localization to synapse	32/2016	78/18614	5.32E-12	8.30E-10	6.36E-10	32
BP	GO:0032943	mononuclear cell proliferation	76/2016	313/18614	7.70E-12	1.18E-09	9.04E-10	76
BP	GO:0048002	antigen processing and presentation of peptide antigen	30/2016	71/18614	9.92E-12	1.50E-09	1.15E-09	30
BP	GO:0002768	immune response-regulating cell surface receptor signaling pathway	78/2016	329/18614	1.46E-11	2.17E-09	1.66E-09	78
BP	GO:0019724	B cell mediated immunity	46/2016	148/18614	1.67E-11	2.43E-09	1.86E-09	46
BP	GO:0030595	leukocyte chemotaxis	63/2016	241/18614	1.71E-11	2.43E-09	1.86E-09	63
BP	GO:0010038	response to metal ion	84/2016	367/18614	1.72E-11	2.43E-09	1.86E-09	84
BP	GO:0034329	cell junction assembly	96/2016	444/18614	1.97E-11	2.75E-09	2.10E-09	96
BP	GO:0022898	regulation of transmembrane transporter activity	71/2016	289/18614	2.11E-11	2.89E-09	2.22E-09	71
BP	GO:0015844	monoamine transport	33/2016	86/18614	2.20E-11	2.98E-09	2.28E-09	33
BP	GO:0032412	regulation of monoatomic ion transmembrane transporter activity	69/2016	278/18614	2.46E-11	3.28E-09	2.51E-09	69
BP	GO:0002366	leukocyte activation involved in immune response	72/2016	296/18614	2.51E-11	3.29E-09	2.52E-09	72
BP	GO:0050433	regulation of catecholamine secretion	26/2016	57/18614	2.91E-11	3.75E-09	2.87E-09	26
BP	GO:0016064	immunoglobulin mediated immune response	45/2016	145/18614	2.93E-11	3.75E-09	2.87E-09	45

BP	GO:1904062	regulation of monoatomic cation transmembrane transport	80/2016	346/18614	3.06E-11	3.86E-09	2.95E-09	80
BP	GO:0033627	cell adhesion mediated by integrin	33/2016	87/18614	3.19E-11	3.96E-09	3.03E-09	33
BP	GO:0007269	neurotransmitter secretion	44/2016	141/18614	3.96E-11	4.79E-09	3.67E-09	44
BP	GO:0099643	signal release from synapse	44/2016	141/18614	3.96E-11	4.79E-09	3.67E-09	44
BP	GO:0007188	adenylate cyclase-modulating G protein-coupled receptor signaling pathway	61/2016	234/18614	4.04E-11	4.83E-09	3.70E-09	61
BP	GO:0007162	negative regulation of cell adhesion	74/2016	312/18614	4.77E-11	5.63E-09	4.31E-09	74
BP	GO:0002263	cell activation involved in immune response	72/2016	300/18614	4.87E-11	5.68E-09	4.35E-09	72
BP	GO:0007204	positive regulation of cytosolic calcium ion concentration	51/2016	179/18614	4.98E-11	5.73E-09	4.39E-09	51
BP	GO:0050866	negative regulation of cell activation	59/2016	225/18614	6.51E-11	7.40E-09	5.67E-09	59
BP	GO:0051966	regulation of synaptic transmission, glutamatergic	31/2016	80/18614	6.60E-11	7.41E-09	5.67E-09	31
BP	GO:0032496	response to lipopolysaccharide	79/2016	345/18614	6.73E-11	7.46E-09	5.71E-09	79
BP	GO:0050432	catecholamine secretion	26/2016	59/18614	7.58E-11	8.31E-09	6.36E-09	26
BP	GO:0002699	positive regulation of immune effector process	66/2016	267/18614	8.04E-11	8.70E-09	6.66E-09	66
BP	GO:0001909	leukocyte mediated cytotoxicity	43/2016	139/18614	9.01E-11	9.59E-09	7.34E-09	43
BP	GO:0048791	calcium ion-regulated exocytosis of neurotransmitter	15/2016	21/18614	9.06E-11	9.59E-09	7.34E-09	15
BP	GO:0002237	response to molecule of bacterial origin	82/2016	366/18614	9.31E-11	9.74E-09	7.45E-09	82
BP	GO:0002504	antigen processing and presentation of peptide or polysaccharide antigen via MHC class II	20/2016	37/18614	1.16E-10	1.20E-08	9.15E-09	20
BP	GO:0050807	regulation of synapse organization	60/2016	234/18614	1.20E-10	1.23E-08	9.42E-09	60
BP	GO:0060326	cell chemotaxis	73/2016	312/18614	1.26E-10	1.27E-08	9.72E-09	73
BP	GO:0017156	calcium-ion regulated exocytosis	27/2016	65/18614	1.72E-10	1.72E-08	1.32E-08	27
BP	GO:0042098	T cell proliferation	56/2016	213/18614	1.78E-10	1.76E-08	1.35E-08	56
BP	GO:0098742	cell-cell adhesion via plasma-membrane adhesion molecules	67/2016	278/18614	1.88E-10	1.82E-08	1.40E-08	67

BP	GO:0006816	calcium ion transport	95/2016	455/18614	1.88E-10	1.82E-08	1.40E-08	95
BP	GO:0002695	negative regulation of leukocyte activation	54/2016	202/18614	1.92E-10	1.84E-08	1.41E-08	54
BP	GO:0043410	positive regulation of MAPK cascade	101/2016	495/18614	1.96E-10	1.86E-08	1.42E-08	101
BP	GO:0019886	antigen processing and presentation of exogenous peptide antigen via MHC class II	18/2016	31/18614	2.01E-10	1.89E-08	1.45E-08	18
BP	GO:0007416	synapse assembly	53/2016	197/18614	2.18E-10	2.03E-08	1.55E-08	53
BP	GO:0099003	vesicle-mediated transport in synapse	57/2016	220/18614	2.26E-10	2.08E-08	1.59E-08	57
BP	GO:0046631	alpha-beta T cell activation	49/2016	175/18614	2.31E-10	2.11E-08	1.61E-08	49
BP	GO:0050806	positive regulation of synaptic transmission	48/2016	170/18614	2.57E-10	2.32E-08	1.78E-08	48
BP	GO:0099504	synaptic vesicle cycle	53/2016	198/18614	2.67E-10	2.39E-08	1.83E-08	53
BP	GO:0051952	regulation of amine transport	34/2016	98/18614	2.73E-10	2.41E-08	1.85E-08	34
BP	GO:0032963	collagen metabolic process	36/2016	108/18614	2.97E-10	2.60E-08	1.99E-08	36
BP	GO:0032409	regulation of transporter activity	74/2016	324/18614	3.05E-10	2.64E-08	2.02E-08	74
BP	GO:0002495	antigen processing and presentation of peptide antigen via MHC class II	19/2016	35/18614	3.07E-10	2.64E-08	2.02E-08	19
BP	GO:0051960	regulation of nervous system development	95/2016	461/18614	3.93E-10	3.35E-08	2.56E-08	95
BP	GO:0018108	peptidyl-tyrosine phosphorylation	81/2016	370/18614	3.97E-10	3.36E-08	2.57E-08	81
BP	GO:0097530	granulocyte migration	45/2016	156/18614	4.33E-10	3.62E-08	2.77E-08	45
BP	GO:0002757	immune response-activating signaling pathway	89/2016	423/18614	4.71E-10	3.87E-08	2.96E-08	89
BP	GO:0010959	regulation of metal ion transport	89/2016	423/18614	4.71E-10	3.87E-08	2.96E-08	89
BP	GO:0018212	peptidyl-tyrosine modification	81/2016	372/18614	5.22E-10	4.25E-08	3.25E-08	81
BP	GO:0031341	regulation of cell killing	36/2016	110/18614	5.30E-10	4.28E-08	3.28E-08	36
BP	GO:0051937	catecholamine transport	28/2016	73/18614	7.25E-10	5.80E-08	4.44E-08	28
BP	GO:0042886	amide transport	77/2016	349/18614	7.38E-10	5.86E-08	4.48E-08	77
BP	GO:0009612	response to mechanical stimulus	55/2016	215/18614	7.76E-10	6.11E-08	4.67E-08	55
BP	GO:0002685	regulation of leukocyte migration	57/2016	227/18614	8.21E-10	6.40E-08	4.90E-08	57
BP	GO:0015837	amine transport	35/2016	107/18614	9.33E-10	7.21E-08	5.52E-08	35
BP	GO:0002819	regulation of adaptive immune response	53/2016	205/18614	1.05E-09	8.02E-08	6.14E-08	53
BP	GO:0042063	gliogenesis	72/2016	320/18614	1.07E-09	8.11E-08	6.21E-08	72

BP	GO:0002764	immune response-regulating signaling pathway	92/2016	450/18614	1.14E-09	8.61E-08	6.59E-08	92
BP	GO:0010001	glial cell differentiation	58/2016	235/18614	1.19E-09	8.87E-08	6.79E-08	58
BP	GO:0042129	regulation of T cell proliferation	49/2016	183/18614	1.24E-09	9.18E-08	7.02E-08	49
BP	GO:0070371	ERK1 and ERK2 cascade	75/2016	341/18614	1.42E-09	1.05E-07	8.01E-08	75
BP	GO:0007613	memory	38/2016	124/18614	1.49E-09	1.08E-07	8.30E-08	38
BP	GO:0021782	glial cell development	37/2016	119/18614	1.55E-09	1.13E-07	8.61E-08	37
BP	GO:0002822	regulation of adaptive immune response based on somatic recombination of immune receptors built from immunoglobulin superfamily domains	50/2016	190/18614	1.60E-09	1.15E-07	8.83E-08	50
BP	GO:0099173	postsynapse organization	49/2016	185/18614	1.84E-09	1.31E-07	1.01E-07	49
BP	GO:0031349	positive regulation of defense response	90/2016	441/18614	1.90E-09	1.34E-07	1.03E-07	90
BP	GO:0001906	cell killing	51/2016	197/18614	2.04E-09	1.44E-07	1.10E-07	51
BP	GO:0001910	regulation of leukocyte mediated cytotoxicity	32/2016	95/18614	2.12E-09	1.48E-07	1.13E-07	32
BP	GO:0008306	associative learning	30/2016	86/18614	2.61E-09	1.80E-07	1.38E-07	30
BP	GO:0061564	axon development	95/2016	479/18614	3.17E-09	2.17E-07	1.66E-07	95
BP	GO:0002440	production of molecular mediator of immune response	72/2016	328/18614	3.30E-09	2.25E-07	1.72E-07	72
BP	GO:0002683	negative regulation of immune system process	96/2016	487/18614	3.62E-09	2.45E-07	1.88E-07	96
BP	GO:0007229	integrin-mediated signaling pathway	35/2016	112/18614	3.67E-09	2.47E-07	1.89E-07	35
BP	GO:0016079	synaptic vesicle exocytosis	32/2016	97/18614	3.82E-09	2.55E-07	1.95E-07	32
BP	GO:1903131	mononuclear cell differentiation	94/2016	474/18614	3.85E-09	2.55E-07	1.95E-07	94
BP	GO:1990266	neutrophil migration	38/2016	128/18614	4.01E-09	2.64E-07	2.02E-07	38
BP	GO:0043270	positive regulation of monoatomic ion transport	56/2016	231/18614	4.59E-09	2.99E-07	2.29E-07	56
BP	GO:0006821	chloride transport	35/2016	113/18614	4.77E-09	3.09E-07	2.37E-07	35
BP	GO:0007215	glutamate receptor signaling pathway	22/2016	52/18614	5.54E-09	3.57E-07	2.73E-07	22
BP	GO:0002478	antigen processing and presentation of exogenous peptide antigen	19/2016	40/18614	5.82E-09	3.72E-07	2.85E-07	19
BP	GO:0051048	negative regulation of secretion	47/2016	180/18614	6.45E-09	4.09E-07	3.13E-07	47
BP	GO:0051588	regulation of neurotransmitter transport	32/2016	99/18614	6.74E-09	4.25E-07	3.25E-07	32
BP	GO:0002456	T cell mediated immunity	37/2016	125/18614	6.99E-09	4.37E-07	3.35E-07	37

BP	GO:0070588	calcium ion transmembrane transport	76/2016	360/18614	7.51E-09	4.66E-07	3.56E-07	76
BP	GO:0048168	regulation of neuronal synaptic plasticity	23/2016	57/18614	7.54E-09	4.66E-07	3.56E-07	23
BP	GO:0070372	regulation of ERK1 and ERK2 cascade	69/2016	315/18614	7.66E-09	4.70E-07	3.60E-07	69
BP	GO:0035725	sodium ion transmembrane transport	47/2016	181/18614	7.81E-09	4.76E-07	3.64E-07	47
BP	GO:0007193	adenylate cyclase-inhibiting G protein-coupled receptor signaling pathway	28/2016	80/18614	8.06E-09	4.87E-07	3.73E-07	28
BP	GO:0050868	negative regulation of T cell activation	38/2016	131/18614	8.15E-09	4.87E-07	3.73E-07	38
BP	GO:0071621	granulocyte chemotaxis	38/2016	131/18614	8.15E-09	4.87E-07	3.73E-07	38
BP	GO:0022408	negative regulation of cell-cell adhesion	51/2016	205/18614	8.79E-09	5.22E-07	4.00E-07	51
BP	GO:0014059	regulation of dopamine secretion	18/2016	37/18614	9.08E-09	5.36E-07	4.10E-07	18
BP	GO:0006814	sodium ion transport	59/2016	254/18614	9.84E-09	5.77E-07	4.41E-07	59
BP	GO:0006909	phagocytosis	56/2016	236/18614	1.04E-08	6.07E-07	4.64E-07	56
BP	GO:0007596	blood coagulation	54/2016	224/18614	1.06E-08	6.14E-07	4.70E-07	54
BP	GO:0043299	leukocyte degranulation	28/2016	81/18614	1.11E-08	6.36E-07	4.87E-07	28
BP	GO:0046879	hormone secretion	67/2016	305/18614	1.12E-08	6.39E-07	4.89E-07	67
BP	GO:0051592	response to calcium ion	41/2016	149/18614	1.16E-08	6.58E-07	5.04E-07	41
BP	GO:0030217	T cell differentiation	66/2016	300/18614	1.35E-08	7.62E-07	5.83E-07	66
BP	GO:0030593	neutrophil chemotaxis	33/2016	107/18614	1.47E-08	8.19E-07	6.27E-07	33
BP	GO:1902414	protein localization to cell junction	33/2016	107/18614	1.47E-08	8.19E-07	6.27E-07	33
BP	GO:0031589	cell-substrate adhesion	75/2016	359/18614	1.51E-08	8.35E-07	6.39E-07	75
BP	GO:1903531	negative regulation of secretion by cell	42/2016	156/18614	1.55E-08	8.50E-07	6.50E-07	42
BP	GO:0014046	dopamine secretion	18/2016	38/18614	1.55E-08	8.50E-07	6.50E-07	18
BP	GO:0051250	negative regulation of lymphocyte activation	44/2016	168/18614	1.76E-08	9.58E-07	7.33E-07	44
BP	GO:0009914	hormone transport	68/2016	315/18614	1.82E-08	9.85E-07	7.54E-07	68
BP	GO:0050878	regulation of body fluid levels	75/2016	361/18614	1.93E-08	1.04E-06	7.95E-07	75
BP	GO:1902476	chloride transmembrane transport	31/2016	98/18614	2.05E-08	1.10E-06	8.40E-07	31
BP	GO:0071277	cellular response to calcium ion	29/2016	88/18614	2.09E-08	1.11E-06	8.49E-07	29
BP	GO:0034767	positive regulation of monoatomic ion transmembrane transport	45/2016	175/18614	2.23E-08	1.18E-06	9.01E-07	45
BP	GO:0046634	regulation of alpha-beta T cell activation	34/2016	114/18614	2.29E-08	1.20E-06	9.20E-07	34
BP	GO:0050817	coagulation	54/2016	229/18614	2.39E-08	1.25E-06	9.55E-07	54

BP	GO:0031343	positive regulation of cell killing	26/2016	74/18614	2.50E-08	1.30E-06	9.93E-07	26
BP	GO:0042554	superoxide anion generation	19/2016	43/18614	2.57E-08	1.33E-06	1.01E-06	19
BP	GO:1903034	regulation of response to wounding	44/2016	170/18614	2.58E-08	1.33E-06	1.01E-06	44
BP	GO:0007599	hemostasis	54/2016	230/18614	2.79E-08	1.43E-06	1.09E-06	54
BP	GO:1903522	regulation of blood circulation	59/2016	261/18614	2.81E-08	1.43E-06	1.10E-06	59
BP	GO:0007187	G protein-coupled receptor signaling pathway, coupled to cyclic nucleotide second messenger	22/2016	56/18614	2.86E-08	1.44E-06	1.11E-06	22
BP	GO:0071248	cellular response to metal ion	49/2016	200/18614	2.90E-08	1.46E-06	1.12E-06	49
BP	GO:0002705	positive regulation of leukocyte mediated immunity	40/2016	148/18614	3.01E-08	1.51E-06	1.15E-06	40
BP	GO:1902105	regulation of leukocyte differentiation	68/2016	319/18614	3.08E-08	1.53E-06	1.17E-06	68
BP	GO:0010720	positive regulation of cell development	85/2016	432/18614	3.11E-08	1.54E-06	1.18E-06	85
BP	GO:0009266	response to temperature stimulus	46/2016	183/18614	3.31E-08	1.63E-06	1.25E-06	46
BP	GO:0015800	acidic amino acid transport	23/2016	61/18614	3.46E-08	1.70E-06	1.30E-06	23
BP	GO:0070665	positive regulation of leukocyte proliferation	43/2016	166/18614	3.61E-08	1.76E-06	1.34E-06	43
BP	GO:0007409	axonogenesis	84/2016	427/18614	3.78E-08	1.83E-06	1.40E-06	84
BP	GO:0002698	negative regulation of immune effector process	35/2016	122/18614	4.26E-08	2.05E-06	1.57E-06	35
BP	GO:0050764	regulation of phagocytosis	31/2016	101/18614	4.51E-08	2.16E-06	1.66E-06	31
BP	GO:0002831	regulation of response to biotic stimulus	89/2016	463/18614	4.60E-08	2.19E-06	1.68E-06	89
BP	GO:0050671	positive regulation of lymphocyte proliferation	39/2016	145/18614	5.15E-08	2.44E-06	1.87E-06	39
BP	GO:0051047	positive regulation of secretion	67/2016	317/18614	5.49E-08	2.59E-06	1.98E-06	67
BP	GO:1902075	cellular response to salt	49/2016	204/18614	5.67E-08	2.64E-06	2.02E-06	49
BP	GO:0019884	antigen processing and presentation of exogenous antigen	20/2016	49/18614	5.68E-08	2.64E-06	2.02E-06	20
BP	GO:0033628	regulation of cell adhesion mediated by integrin	20/2016	49/18614	5.68E-08	2.64E-06	2.02E-06	20
BP	GO:0002399	MHC class II protein complex assembly	11/2016	16/18614	6.10E-08	2.80E-06	2.14E-06	11

BP	GO:0002503	peptide antigen assembly with MHC class II protein complex	11/2016	16/18614	6.10E-08	2.80E-06	2.14E-06	11
BP	GO:0010975	regulation of neuron projection development	87/2016	452/18614	6.11E-08	2.80E-06	2.14E-06	87
BP	GO:0014003	oligodendrocyte development	19/2016	45/18614	6.33E-08	2.89E-06	2.21E-06	19
BP	GO:0007200	phospholipase C-activating G protein-coupled receptor signaling pathway	33/2016	113/18614	6.43E-08	2.92E-06	2.23E-06	33
BP	GO:0050920	regulation of chemotaxis	53/2016	230/18614	7.24E-08	3.26E-06	2.50E-06	53
BP	GO:0050730	regulation of peptidyl-tyrosine phosphorylation	57/2016	255/18614	7.26E-08	3.26E-06	2.50E-06	57
BP	GO:0071219	cellular response to molecule of bacterial origin	54/2016	237/18614	8.15E-08	3.64E-06	2.79E-06	54
BP	GO:0071241	cellular response to inorganic substance	53/2016	231/18614	8.42E-08	3.74E-06	2.86E-06	53
BP	GO:0021675	nerve development	28/2016	88/18614	8.52E-08	3.77E-06	2.89E-06	28
BP	GO:0042742	defense response to bacterium	66/2016	314/18614	8.57E-08	3.77E-06	2.89E-06	66
BP	GO:1903053	regulation of extracellular matrix organization	22/2016	59/18614	8.66E-08	3.79E-06	2.90E-06	22
BP	GO:0001508	action potential	38/2016	142/18614	8.79E-08	3.83E-06	2.94E-06	38
BP	GO:0032946	positive regulation of mononuclear cell proliferation	39/2016	148/18614	9.36E-08	4.07E-06	3.11E-06	39
BP	GO:0099505	regulation of presynaptic membrane potential	15/2016	30/18614	9.99E-08	4.31E-06	3.29E-06	15
BP	GO:0061041	regulation of wound healing	37/2016	137/18614	1.00E-07	4.31E-06	3.29E-06	37
BP	GO:0042119	neutrophil activation	18/2016	42/18614	1.07E-07	4.56E-06	3.49E-06	18
BP	GO:0002444	myeloid leukocyte mediated immunity	32/2016	110/18614	1.12E-07	4.77E-06	3.65E-06	32
BP	GO:0003012	muscle system process	86/2016	452/18614	1.24E-07	5.26E-06	4.02E-06	86
BP	GO:0015872	dopamine transport	20/2016	51/18614	1.26E-07	5.31E-06	4.06E-06	20
BP	GO:0015698	inorganic anion transport	44/2016	179/18614	1.31E-07	5.48E-06	4.19E-06	44
BP	GO:0050777	negative regulation of immune response	46/2016	191/18614	1.31E-07	5.48E-06	4.19E-06	46
BP	GO:0060560	developmental growth involved in morphogenesis	53/2016	234/18614	1.32E-07	5.48E-06	4.19E-06	53
BP	GO:0032102	negative regulation of response to external stimulus	85/2016	446/18614	1.36E-07	5.64E-06	4.31E-06	85
BP	GO:0002544	chronic inflammatory response	12/2016	20/18614	1.38E-07	5.68E-06	4.34E-06	12

BP	GO:2001185	regulation of CD8-positive, alpha-beta T cell activation	12/2016	20/18614	1.38E-07	5.68E-06	4.34E-06	12
BP	GO:0019932	second-messenger-mediated signaling	62/2016	292/18614	1.43E-07	5.85E-06	4.47E-06	62
BP	GO:0098661	inorganic anion transmembrane transport	34/2016	122/18614	1.44E-07	5.85E-06	4.47E-06	34
BP	GO:0045921	positive regulation of exocytosis	26/2016	80/18614	1.52E-07	6.16E-06	4.72E-06	26
BP	GO:0036293	response to decreased oxygen levels	64/2016	306/18614	1.62E-07	6.54E-06	5.00E-06	64
BP	GO:0001666	response to hypoxia	62/2016	293/18614	1.63E-07	6.55E-06	5.01E-06	62
BP	GO:0097120	receptor localization to synapse	22/2016	61/18614	1.72E-07	6.90E-06	5.28E-06	22
BP	GO:0070482	response to oxygen levels	68/2016	333/18614	1.75E-07	6.96E-06	5.32E-06	68
BP	GO:0045730	respiratory burst	17/2016	39/18614	1.78E-07	7.08E-06	5.42E-06	17
BP	GO:0051968	positive regulation of synaptic transmission, glutamatergic	16/2016	35/18614	1.83E-07	7.23E-06	5.53E-06	16
BP	GO:1901214	regulation of neuron death	67/2016	327/18614	1.88E-07	7.40E-06	5.67E-06	67
BP	GO:0071222	cellular response to lipopolysaccharide	51/2016	224/18614	1.90E-07	7.43E-06	5.69E-06	51
BP	GO:0019221	cytokine-mediated signaling pathway	91/2016	492/18614	2.05E-07	7.98E-06	6.11E-06	91
BP	GO:0006959	humoral immune response	55/2016	250/18614	2.13E-07	8.28E-06	6.34E-06	55
BP	GO:0036230	granulocyte activation	19/2016	48/18614	2.18E-07	8.41E-06	6.44E-06	19
BP	GO:1903532	positive regulation of secretion by cell	61/2016	289/18614	2.24E-07	8.64E-06	6.61E-06	61
BP	GO:0002709	regulation of T cell mediated immunity	29/2016	97/18614	2.28E-07	8.75E-06	6.70E-06	29
BP	GO:0002687	positive regulation of leukocyte migration	38/2016	147/18614	2.33E-07	8.87E-06	6.79E-06	38
BP	GO:0006820	monoatomic anion transport	38/2016	147/18614	2.33E-07	8.87E-06	6.79E-06	38
BP	GO:0006936	muscle contraction	70/2016	349/18614	2.38E-07	9.00E-06	6.89E-06	70
BP	GO:0002286	T cell activation involved in immune response	33/2016	119/18614	2.47E-07	9.33E-06	7.14E-06	33
BP	GO:1903539	protein localization to postsynaptic membrane	18/2016	44/18614	2.51E-07	9.42E-06	7.21E-06	18
BP	GO:0001912	positive regulation of leukocyte mediated cytotoxicity	23/2016	67/18614	2.57E-07	9.61E-06	7.36E-06	23
BP	GO:0046928	regulation of neurotransmitter secretion	26/2016	82/18614	2.64E-07	9.84E-06	7.53E-06	26
BP	GO:0050853	B cell receptor signaling pathway	25/2016	77/18614	2.68E-07	9.91E-06	7.59E-06	25

BP	GO:0019233	sensory perception of pain	30/2016	103/18614	2.68E-07	9.91E-06	7.59E-06	30
BP	GO:0098656	monoatomic anion transmembrane transport	34/2016	125/18614	2.71E-07	9.98E-06	7.64E-06	34
BP	GO:0002526	acute inflammatory response	32/2016	114/18614	2.77E-07	1.01E-05	7.76E-06	32
BP	GO:2001257	regulation of cation channel activity	39/2016	154/18614	2.91E-07	1.06E-05	8.10E-06	39
BP	GO:0002275	myeloid cell activation involved in immune response	29/2016	98/18614	2.91E-07	1.06E-05	8.10E-06	29
BP	GO:0002706	regulation of lymphocyte mediated immunity	44/2016	184/18614	3.03E-07	1.10E-05	8.40E-06	44
BP	GO:1990138	neuron projection extension	42/2016	172/18614	3.04E-07	1.10E-05	8.40E-06	42
BP	GO:0042116	macrophage activation	31/2016	109/18614	3.07E-07	1.10E-05	8.45E-06	31
BP	GO:0035710	CD4-positive, alpha-beta T cell activation	32/2016	115/18614	3.44E-07	1.23E-05	9.42E-06	32
BP	GO:0062237	protein localization to postsynapse	18/2016	45/18614	3.76E-07	1.34E-05	1.02E-05	18
BP	GO:0098815	modulation of excitatory postsynaptic potential	18/2016	45/18614	3.76E-07	1.34E-05	1.02E-05	18
BP	GO:0030098	lymphocyte differentiation	80/2016	422/18614	3.96E-07	1.40E-05	1.07E-05	80
BP	GO:1903038	negative regulation of leukocyte cell-cell adhesion	38/2016	150/18614	4.07E-07	1.44E-05	1.10E-05	38
BP	GO:0031646	positive regulation of nervous system process	16/2016	37/18614	4.70E-07	1.64E-05	1.26E-05	16
BP	GO:0002790	peptide secretion	53/2016	243/18614	4.70E-07	1.64E-05	1.26E-05	53
BP	GO:0006801	superoxide metabolic process	24/2016	74/18614	4.73E-07	1.65E-05	1.26E-05	24
BP	GO:0009308	amine metabolic process	32/2016	117/18614	5.26E-07	1.83E-05	1.40E-05	32
BP	GO:1904064	positive regulation of cation transmembrane transport	38/2016	152/18614	5.83E-07	2.02E-05	1.54E-05	38
BP	GO:0071216	cellular response to biotic stimulus	56/2016	264/18614	5.86E-07	2.02E-05	1.54E-05	56
BP	GO:0048709	oligodendrocyte differentiation	29/2016	101/18614	5.88E-07	2.02E-05	1.54E-05	29
BP	GO:0044703	multi-organism reproductive process	47/2016	207/18614	6.14E-07	2.10E-05	1.61E-05	47
BP	GO:0019226	transmission of nerve impulse	22/2016	65/18614	6.16E-07	2.10E-05	1.61E-05	22
BP	GO:0051051	negative regulation of transport	88/2016	483/18614	6.34E-07	2.15E-05	1.65E-05	88
BP	GO:0050851	antigen receptor-mediated signaling pathway	46/2016	201/18614	6.35E-07	2.15E-05	1.65E-05	46

BP	GO:0098976	excitatory chemical synaptic transmission	8/2016	10/18614	6.88E-07	2.32E-05	1.77E-05	8
BP	GO:0050727	regulation of inflammatory response	78/2016	414/18614	7.11E-07	2.38E-05	1.82E-05	78
BP	GO:0002285	lymphocyte activation involved in immune response	47/2016	208/18614	7.12E-07	2.38E-05	1.82E-05	47
BP	GO:0002573	myeloid leukocyte differentiation	50/2016	227/18614	7.20E-07	2.40E-05	1.84E-05	50
BP	GO:0050864	regulation of B cell activation	34/2016	130/18614	7.38E-07	2.45E-05	1.88E-05	34
BP	GO:0070997	neuron death	72/2016	373/18614	7.42E-07	2.46E-05	1.88E-05	72
BP	GO:0001503	ossification	80/2016	429/18614	7.88E-07	2.60E-05	1.99E-05	80
BP	GO:0043266	regulation of potassium ion transport	30/2016	108/18614	8.24E-07	2.71E-05	2.07E-05	30
BP	GO:0032613	interleukin-10 production	22/2016	66/18614	8.30E-07	2.71E-05	2.07E-05	22
BP	GO:0032653	regulation of interleukin-10 production	22/2016	66/18614	8.30E-07	2.71E-05	2.07E-05	22
BP	GO:0044706	multi-multicellular organism process	48/2016	216/18614	9.10E-07	2.96E-05	2.27E-05	48
BP	GO:0006968	cellular defense response	19/2016	52/18614	9.40E-07	3.05E-05	2.33E-05	19
BP	GO:0032729	positive regulation of type II interferon production	24/2016	77/18614	1.07E-06	3.45E-05	2.64E-05	24
BP	GO:0022617	extracellular matrix disassembly	21/2016	62/18614	1.09E-06	3.51E-05	2.68E-05	21
BP	GO:0017158	regulation of calcium ion-dependent exocytosis	16/2016	39/18614	1.11E-06	3.57E-05	2.73E-05	16
BP	GO:0015850	organic hydroxy compound transport	60/2016	296/18614	1.17E-06	3.75E-05	2.87E-05	60
BP	GO:0002367	cytokine production involved in immune response	32/2016	121/18614	1.19E-06	3.78E-05	2.89E-05	32
BP	GO:0050672	negative regulation of lymphocyte proliferation	26/2016	88/18614	1.22E-06	3.87E-05	2.96E-05	26
BP	GO:0045619	regulation of lymphocyte differentiation	47/2016	212/18614	1.26E-06	4.00E-05	3.06E-05	47
BP	GO:0007565	female pregnancy	43/2016	187/18614	1.27E-06	4.01E-05	3.07E-05	43
BP	GO:2000463	positive regulation of excitatory postsynaptic potential	14/2016	31/18614	1.30E-06	4.07E-05	3.12E-05	14
BP	GO:0050731	positive regulation of peptidyl-tyrosine phosphorylation	42/2016	181/18614	1.31E-06	4.09E-05	3.13E-05	42
BP	GO:0030072	peptide hormone secretion	51/2016	238/18614	1.34E-06	4.18E-05	3.20E-05	51
BP	GO:0042102	positive regulation of T cell proliferation	29/2016	105/18614	1.42E-06	4.42E-05	3.38E-05	29

BP	GO:0050766	positive regulation of phagocytosis	23/2016	73/18614	1.45E-06	4.48E-05	3.43E-05	23
BP	GO:0008016	regulation of heart contraction	47/2016	213/18614	1.45E-06	4.49E-05	3.44E-05	47
BP	GO:0002704	negative regulation of leukocyte mediated immunity	22/2016	68/18614	1.47E-06	4.54E-05	3.47E-05	22
BP	GO:0032945	negative regulation of mononuclear cell proliferation	26/2016	89/18614	1.55E-06	4.71E-05	3.60E-05	26
BP	GO:0043367	CD4-positive, alpha-beta T cell differentiation	26/2016	89/18614	1.55E-06	4.71E-05	3.60E-05	26
BP	GO:0002396	MHC protein complex assembly	11/2016	20/18614	1.55E-06	4.71E-05	3.60E-05	11
BP	GO:0002501	peptide antigen assembly with MHC protein complex	11/2016	20/18614	1.55E-06	4.71E-05	3.60E-05	11
BP	GO:0032755	positive regulation of interleukin-6 production	28/2016	100/18614	1.59E-06	4.82E-05	3.69E-05	28
BP	GO:0015833	peptide transport	54/2016	259/18614	1.62E-06	4.89E-05	3.74E-05	54
BP	GO:0072006	nephron development	37/2016	152/18614	1.64E-06	4.92E-05	3.76E-05	37
BP	GO:0002824	positive regulation of adaptive immune response based on somatic recombination of immune receptors built from immunoglobulin superfamily domains	32/2016	123/18614	1.75E-06	5.25E-05	4.02E-05	32
BP	GO:0051962	positive regulation of nervous system development	57/2016	280/18614	1.87E-06	5.59E-05	4.28E-05	57
BP	GO:0001913	T cell mediated cytotoxicity	20/2016	59/18614	1.93E-06	5.74E-05	4.39E-05	20
BP	GO:0033555	multicellular organismal response to stress	26/2016	90/18614	1.95E-06	5.79E-05	4.43E-05	26
BP	GO:0031338	regulation of vesicle fusion	12/2016	24/18614	1.96E-06	5.79E-05	4.43E-05	12
BP	GO:0071706	tumor necrosis factor superfamily cytokine production	43/2016	190/18614	1.99E-06	5.85E-05	4.48E-05	43
BP	GO:1903555	regulation of tumor necrosis factor superfamily cytokine production	43/2016	190/18614	1.99E-06	5.85E-05	4.48E-05	43
BP	GO:0030574	collagen catabolic process	17/2016	45/18614	2.04E-06	5.95E-05	4.55E-05	17
BP	GO:0031295	T cell costimulation	17/2016	45/18614	2.04E-06	5.95E-05	4.55E-05	17
BP	GO:1903307	positive regulation of regulated secretory pathway	17/2016	45/18614	2.04E-06	5.95E-05	4.55E-05	17

BP	GO:0033631	cell-cell adhesion mediated by integrin	10/2016	17/18614	2.06E-06	5.95E-05	4.55E-05	10
BP	GO:1902107	positive regulation of leukocyte differentiation	42/2016	184/18614	2.06E-06	5.95E-05	4.55E-05	42
BP	GO:1903708	positive regulation of hemopoiesis	42/2016	184/18614	2.06E-06	5.95E-05	4.55E-05	42
BP	GO:0070374	positive regulation of ERK1 and ERK2 cascade	48/2016	222/18614	2.08E-06	5.98E-05	4.58E-05	48
BP	GO:0036037	CD8-positive, alpha-beta T cell activation	13/2016	28/18614	2.12E-06	6.06E-05	4.64E-05	13
BP	GO:0070664	negative regulation of leukocyte proliferation	27/2016	96/18614	2.21E-06	6.33E-05	4.84E-05	27
BP	GO:0061448	connective tissue development	56/2016	275/18614	2.27E-06	6.47E-05	4.95E-05	56
BP	GO:0050729	positive regulation of inflammatory response	37/2016	154/18614	2.28E-06	6.49E-05	4.97E-05	37
BP	GO:0050818	regulation of coagulation	23/2016	75/18614	2.44E-06	6.91E-05	5.29E-05	23
BP	GO:0046632	alpha-beta T cell differentiation	31/2016	119/18614	2.45E-06	6.92E-05	5.30E-05	31
BP	GO:0042417	dopamine metabolic process	16/2016	41/18614	2.46E-06	6.93E-05	5.31E-05	16
BP	GO:0006584	catecholamine metabolic process	19/2016	55/18614	2.51E-06	7.00E-05	5.35E-05	19
BP	GO:0009712	catechol-containing compound metabolic process	19/2016	55/18614	2.51E-06	7.00E-05	5.35E-05	19
BP	GO:0050805	negative regulation of synaptic transmission	19/2016	55/18614	2.51E-06	7.00E-05	5.35E-05	19
BP	GO:0030193	regulation of blood coagulation	22/2016	70/18614	2.55E-06	7.09E-05	5.42E-05	22
BP	GO:0030534	adult behavior	35/2016	143/18614	2.71E-06	7.48E-05	5.72E-05	35
BP	GO:0050921	positive regulation of chemotaxis	35/2016	143/18614	2.71E-06	7.48E-05	5.72E-05	35
BP	GO:0006576	biogenic amine metabolic process	27/2016	97/18614	2.75E-06	7.59E-05	5.81E-05	27
BP	GO:0051963	regulation of synapse assembly	30/2016	114/18614	2.83E-06	7.77E-05	5.94E-05	30
BP	GO:0045580	regulation of T cell differentiation	41/2016	180/18614	2.87E-06	7.87E-05	6.02E-05	41
BP	GO:0008038	neuron recognition	17/2016	46/18614	2.91E-06	7.95E-05	6.09E-05	17
BP	GO:0007156	homophilic cell adhesion via plasma membrane adhesion molecules	39/2016	168/18614	3.05E-06	8.30E-05	6.35E-05	39
BP	GO:0007411	axon guidance	48/2016	225/18614	3.09E-06	8.37E-05	6.40E-05	48
BP	GO:0097485	neuron projection guidance	48/2016	225/18614	3.09E-06	8.37E-05	6.40E-05	48
BP	GO:0042552	myelination	35/2016	144/18614	3.21E-06	8.66E-05	6.62E-05	35
BP	GO:1903557	positive regulation of tumor necrosis factor	29/2016	109/18614	3.25E-06	8.73E-05	6.69E-05	29

		superfamily cytokine production						
BP	GO:0050767	regulation of neurogenesis	71/2016	381/18614	3.32E-06	8.91E-05	6.82E-05	71
BP	GO:0006949	syncytium formation	21/2016	66/18614	3.44E-06	9.19E-05	7.04E-05	21
BP	GO:0035640	exploration behavior	13/2016	29/18614	3.45E-06	9.21E-05	7.05E-05	13
BP	GO:0007160	cell-matrix adhesion	50/2016	239/18614	3.50E-06	9.32E-05	7.14E-05	50
BP	GO:0003015	heart process	53/2016	259/18614	3.61E-06	9.58E-05	7.33E-05	53
BP	GO:0010721	negative regulation of cell development	57/2016	286/18614	3.73E-06	9.87E-05	7.56E-05	57
BP	GO:0002833	positive regulation of response to biotic stimulus	63/2016	327/18614	3.83E-06	0.00010091	7.72E-05	63
BP	GO:0007626	locomotory behavior	44/2016	201/18614	3.88E-06	0.00010191	7.80E-05	44
BP	GO:0031294	lymphocyte costimulation	17/2016	47/18614	4.10E-06	0.00010733	8.21E-05	17
BP	GO:0042220	response to cocaine	17/2016	47/18614	4.10E-06	0.00010733	8.21E-05	17
BP	GO:0046635	positive regulation of alpha-beta T cell activation	22/2016	72/18614	4.29E-06	0.00011162	8.54E-05	22
BP	GO:1900046	regulation of hemostasis	22/2016	72/18614	4.29E-06	0.00011162	8.54E-05	22
BP	GO:0045765	regulation of angiogenesis	66/2016	349/18614	4.37E-06	0.00011341	8.68E-05	66
BP	GO:0007272	ensheathment of neurons	35/2016	146/18614	4.48E-06	0.00011541	8.83E-05	35
BP	GO:0008366	axon ensheathment	35/2016	146/18614	4.48E-06	0.00011541	8.83E-05	35
BP	GO:0032760	positive regulation of tumor necrosis factor production	28/2016	105/18614	4.54E-06	0.00011671	8.93E-05	28
BP	GO:0051932	synaptic transmission, GABAergic	19/2016	57/18614	4.60E-06	0.00011756	9.00E-05	19
BP	GO:0000768	syncytium formation by plasma membrane fusion	20/2016	62/18614	4.61E-06	0.00011756	9.00E-05	20
BP	GO:0140253	cell-cell fusion	20/2016	62/18614	4.61E-06	0.00011756	9.00E-05	20
BP	GO:0010469	regulation of signaling receptor activity	40/2016	177/18614	4.64E-06	0.00011794	9.03E-05	40
BP	GO:0006835	dicarboxylic acid transport	26/2016	94/18614	4.75E-06	0.00012056	9.23E-05	26
BP	GO:0034764	positive regulation of transmembrane transport	49/2016	235/18614	4.82E-06	0.00012177	9.32E-05	49
BP	GO:0031099	regeneration	42/2016	190/18614	4.92E-06	0.00012394	9.49E-05	42
BP	GO:0090322	regulation of superoxide metabolic process	14/2016	34/18614	4.97E-06	0.00012492	9.56E-05	14
BP	GO:0001763	morphogenesis of a branching structure	44/2016	203/18614	5.09E-06	0.00012724	9.74E-05	44
BP	GO:0051216	cartilage development	44/2016	203/18614	5.09E-06	0.00012724	9.74E-05	44
BP	GO:0002578	negative regulation of antigen processing and presentation	7/2016	9/18614	5.11E-06	0.00012754	9.76E-05	7
BP	GO:0014002	astrocyte development	16/2016	43/18614	5.13E-06	0.00012761	9.77E-05	16

BP	GO:0002821	positive regulation of adaptive immune response	32/2016	129/18614	5.27E-06	0.00013063	1.00E-04	32
BP	GO:0032928	regulation of superoxide anion generation	11/2016	22/18614	5.30E-06	0.00013071	0.00010004	11
BP	GO:2001044	regulation of integrin-mediated signaling pathway	11/2016	22/18614	5.30E-06	0.00013071	0.00010004	11
BP	GO:0007517	muscle organ development	66/2016	351/18614	5.33E-06	0.00013127	0.00010047	66
BP	GO:1902563	regulation of neutrophil activation	9/2016	15/18614	5.48E-06	0.00013456	0.00010298	9
BP	GO:0042113	B cell activation	55/2016	276/18614	5.51E-06	0.0001348	0.00010317	55
BP	GO:0071260	cellular response to mechanical stimulus	22/2016	73/18614	5.52E-06	0.00013481	0.00010317	22
BP	GO:0022010	central nervous system myelination	12/2016	26/18614	5.67E-06	0.00013745	0.0001052	12
BP	GO:0032291	axon ensheathment in central nervous system	12/2016	26/18614	5.67E-06	0.00013745	0.0001052	12
BP	GO:0099637	neurotransmitter receptor transport	12/2016	26/18614	5.67E-06	0.00013745	0.0001052	12
BP	GO:0043300	regulation of leukocyte degranulation	17/2016	48/18614	5.72E-06	0.00013812	0.00010571	17
BP	GO:0045088	regulation of innate immune response	69/2016	373/18614	5.90E-06	0.00014211	0.00010876	69
BP	GO:0032640	tumor necrosis factor production	41/2016	185/18614	5.93E-06	0.00014211	0.00010876	41
BP	GO:0032680	regulation of tumor necrosis factor production	41/2016	185/18614	5.93E-06	0.00014211	0.00010876	41
BP	GO:0032609	type II interferon production	30/2016	118/18614	6.04E-06	0.000144	0.00011021	30
BP	GO:0032649	regulation of type II interferon production	30/2016	118/18614	6.04E-06	0.000144	0.00011021	30
BP	GO:0031630	regulation of synaptic vesicle fusion to presynaptic active zone membrane	8/2016	12/18614	6.19E-06	0.0001468	0.00011235	8
BP	GO:1901632	regulation of synaptic vesicle membrane organization	8/2016	12/18614	6.19E-06	0.0001468	0.00011235	8
BP	GO:0045621	positive regulation of lymphocyte differentiation	32/2016	130/18614	6.27E-06	0.00014833	0.00011352	32
BP	GO:0002718	regulation of cytokine production involved in immune response	30/2016	119/18614	7.25E-06	0.00017033	0.00013036	30
BP	GO:0002347	response to tumor cell	16/2016	44/18614	7.25E-06	0.00017033	0.00013036	16

BP	GO:0099084	postsynaptic specialization organization	16/2016	44/18614	7.25E-06	0.00017033	0.00013036	16
BP	GO:0009410	response to xenobiotic stimulus	78/2016	440/18614	7.76E-06	0.00018163	0.00013901	78
BP	GO:0019722	calcium-mediated signaling	41/2016	187/18614	7.84E-06	0.00018309	0.00014013	41
BP	GO:1901342	regulation of vasculature development	66/2016	355/18614	7.87E-06	0.00018337	0.00014034	66
BP	GO:0032930	positive regulation of superoxide anion generation	10/2016	19/18614	7.97E-06	0.00018534	0.00014185	10
BP	GO:0002708	positive regulation of lymphocyte mediated immunity	31/2016	126/18614	8.79E-06	0.00020378	0.00015596	31
BP	GO:0034109	homotypic cell-cell adhesion	26/2016	97/18614	8.88E-06	0.00020545	0.00015724	26
BP	GO:1903861	positive regulation of dendrite extension	11/2016	23/18614	9.16E-06	0.00021035	0.00016099	11
BP	GO:0097553	calcium ion transmembrane import into cytosol	43/2016	201/18614	9.18E-06	0.00021035	0.00016099	43
BP	GO:1901888	regulation of cell junction assembly	45/2016	214/18614	9.19E-06	0.00021035	0.00016099	45
BP	GO:0046883	regulation of hormone secretion	50/2016	247/18614	9.21E-06	0.00021035	0.00016099	50
BP	GO:0060047	heart contraction	50/2016	247/18614	9.21E-06	0.00021035	0.00016099	50
BP	GO:0030902	hindbrain development	36/2016	157/18614	9.65E-06	0.00021996	0.00016835	36
BP	GO:0097028	dendritic cell differentiation	16/2016	45/18614	1.01E-05	0.00023023	0.0001762	16
BP	GO:0099011	neuronal dense core vesicle exocytosis	6/2016	7/18614	1.02E-05	0.00023087	0.0001767	6
BP	GO:0002700	regulation of production of molecular mediator of immune response	41/2016	189/18614	1.03E-05	0.0002326	0.00017802	41
BP	GO:0031663	lipopolysaccharide-mediated signaling pathway	20/2016	65/18614	1.03E-05	0.0002326	0.00017802	20
BP	GO:1903706	regulation of hemopoiesis	74/2016	415/18614	1.07E-05	0.00023996	0.00018365	74
BP	GO:0001914	regulation of T cell mediated cytotoxicity	17/2016	50/18614	1.07E-05	0.00024055	0.0001841	17
BP	GO:0002886	regulation of myeloid leukocyte mediated immunity	19/2016	60/18614	1.07E-05	0.00024055	0.0001841	19
BP	GO:0043370	regulation of CD4-positive, alpha-beta T cell differentiation	18/2016	55/18614	1.09E-05	0.00024378	0.00018658	18

BP	GO:0002486	antigen processing and presentation of endogenous peptide antigen via MHC class I via ER pathway, TAP-independent	9/2016	16/18614	1.13E-05	0.00025171	0.00019264	9
BP	GO:0048172	regulation of short-term neuronal synaptic plasticity	9/2016	16/18614	1.13E-05	0.00025171	0.00019264	9
BP	GO:0042267	natural killer cell mediated cytotoxicity	22/2016	76/18614	1.14E-05	0.00025186	0.00019276	22
BP	GO:0048708	astrocyte differentiation	24/2016	87/18614	1.14E-05	0.00025328	0.00019384	24
BP	GO:0030073	insulin secretion	42/2016	197/18614	1.27E-05	0.00027961	0.000214	42
BP	GO:0032635	interleukin-6 production	39/2016	178/18614	1.31E-05	0.00028845	0.00022076	39
BP	GO:0032675	regulation of interleukin-6 production	39/2016	178/18614	1.31E-05	0.00028845	0.00022076	39
BP	GO:0052547	regulation of peptidase activity	75/2016	425/18614	1.36E-05	0.00029708	0.00022737	75
BP	GO:0072593	reactive oxygen species metabolic process	48/2016	237/18614	1.36E-05	0.00029708	0.00022737	48
BP	GO:0099072	regulation of postsynaptic membrane neurotransmitter receptor levels	24/2016	88/18614	1.41E-05	0.00030842	0.00023605	24
BP	GO:0045766	positive regulation of angiogenesis	40/2016	185/18614	1.43E-05	0.00030943	0.00023682	40
BP	GO:1904018	positive regulation of vasculature development	40/2016	185/18614	1.43E-05	0.00030943	0.00023682	40
BP	GO:2000514	regulation of CD4-positive, alpha-beta T cell activation	22/2016	77/18614	1.43E-05	0.00030943	0.00023682	22
BP	GO:0002577	regulation of antigen processing and presentation	10/2016	20/18614	1.44E-05	0.00030962	0.00023697	10
BP	GO:0042053	regulation of dopamine metabolic process	10/2016	20/18614	1.44E-05	0.00030962	0.00023697	10
BP	GO:0042069	regulation of catecholamine metabolic process	10/2016	20/18614	1.44E-05	0.00030962	0.00023697	10
BP	GO:0002418	immune response to tumor cell	12/2016	28/18614	1.45E-05	0.0003101	0.00023733	12
BP	GO:0007214	gamma-aminobutyric acid signaling pathway	12/2016	28/18614	1.45E-05	0.0003101	0.00023733	12
BP	GO:0098698	postsynaptic specialization assembly	12/2016	28/18614	1.45E-05	0.0003101	0.00023733	12
BP	GO:2001046	positive regulation of integrin-mediated signaling pathway	8/2016	13/18614	1.46E-05	0.0003101	0.00023733	8

BP	GO:0048588	developmental cell growth	47/2016	231/18614	1.47E-05	0.000312	0.00023878	47
BP	GO:0018057	peptidyl-lysine oxidation	5/2016	5/18614	1.48E-05	0.00031464	0.00024081	5
BP	GO:1901379	regulation of potassium ion transmembrane transport	25/2016	94/18614	1.52E-05	0.00032186	0.00024633	25
BP	GO:0060143	positive regulation of syncytium formation by plasma membrane fusion	11/2016	24/18614	1.53E-05	0.00032199	0.00024644	11
BP	GO:0072001	renal system development	60/2016	320/18614	1.53E-05	0.00032266	0.00024694	60
BP	GO:0090066	regulation of anatomical structure size	84/2016	492/18614	1.54E-05	0.00032266	0.00024694	84
BP	GO:0048251	elastic fiber assembly	7/2016	10/18614	1.54E-05	0.00032369	0.00024774	7
BP	GO:0042130	negative regulation of T cell proliferation	21/2016	72/18614	1.57E-05	0.00032866	0.00025154	21
BP	GO:0140029	exocytic process	23/2016	83/18614	1.60E-05	0.00033333	0.00025511	23
BP	GO:0043523	regulation of neuron apoptotic process	45/2016	219/18614	1.71E-05	0.00035515	0.00027181	45
BP	GO:0051591	response to cAMP	24/2016	89/18614	1.74E-05	0.00036142	0.00027661	24
BP	GO:0001706	endoderm formation	18/2016	57/18614	1.90E-05	0.00039421	0.0003017	18
BP	GO:0071711	basement membrane organization	13/2016	33/18614	1.92E-05	0.00039763	0.00030432	13
BP	GO:0061138	morphogenesis of a branching epithelium	40/2016	188/18614	2.12E-05	0.00043729	0.00033468	40
BP	GO:0002476	antigen processing and presentation of endogenous peptide antigen via MHC class Ib	9/2016	17/18614	2.17E-05	0.00044667	0.00034185	9
BP	GO:0002484	antigen processing and presentation of endogenous peptide antigen via MHC class I via ER pathway	9/2016	17/18614	2.17E-05	0.00044667	0.00034185	9
BP	GO:0007568	aging	37/2016	169/18614	2.20E-05	0.00045124	0.00034535	37
BP	GO:0002228	natural killer cell mediated immunity	22/2016	79/18614	2.23E-05	0.00045467	0.00034798	22
BP	GO:0086001	cardiac muscle cell action potential	22/2016	79/18614	2.23E-05	0.00045467	0.00034798	22
BP	GO:0032623	interleukin-2 production	19/2016	63/18614	2.34E-05	0.00047485	0.00036343	19
BP	GO:0032663	regulation of interleukin-2 production	19/2016	63/18614	2.34E-05	0.00047485	0.00036343	19
BP	GO:0099601	regulation of neurotransmitter receptor activity	19/2016	63/18614	2.34E-05	0.00047485	0.00036343	19
BP	GO:0002688	regulation of leukocyte chemotaxis	30/2016	126/18614	2.41E-05	0.00048811	0.00037357	30
BP	GO:0002483	antigen processing and presentation of	11/2016	25/18614	2.46E-05	0.00049601	0.00037962	11

		endogenous peptide antigen						
BP	GO:0051954	positive regulation of amine transport	15/2016	43/18614	2.48E-05	0.00050041	0.00038299	15
BP	GO:0006898	receptor-mediated endocytosis	50/2016	256/18614	2.53E-05	0.00050794	0.00038875	50
BP	GO:0035987	endodermal cell differentiation	16/2016	48/18614	2.58E-05	0.00051648	0.00039529	16
BP	GO:0030900	forebrain development	70/2016	397/18614	2.67E-05	0.00053411	0.00040878	70
BP	GO:0002711	positive regulation of T cell mediated immunity	20/2016	69/18614	2.75E-05	0.00054885	0.00042006	20
BP	GO:0030168	platelet activation	31/2016	133/18614	2.78E-05	0.00055406	0.00042404	31
BP	GO:0033238	regulation of amine metabolic process	13/2016	34/18614	2.81E-05	0.00055763	0.00042677	13
BP	GO:0072073	kidney epithelium development	33/2016	146/18614	3.04E-05	0.00060345	0.00046185	33
BP	GO:0045956	positive regulation of calcium ion-dependent exocytosis	8/2016	14/18614	3.07E-05	0.00060815	0.00046545	8
BP	GO:0098657	import into cell	51/2016	265/18614	3.18E-05	0.00062828	0.00048085	51
BP	GO:0045823	positive regulation of heart contraction	14/2016	39/18614	3.19E-05	0.0006289	0.00048132	14
BP	GO:0060142	regulation of syncytium formation by plasma membrane fusion	12/2016	30/18614	3.36E-05	0.00066021	0.00050529	12
BP	GO:0009409	response to cold	17/2016	54/18614	3.37E-05	0.00066021	0.00050529	17
BP	GO:0046718	viral entry into host cell	34/2016	153/18614	3.37E-05	0.00066021	0.00050529	34
BP	GO:0045582	positive regulation of T cell differentiation	28/2016	116/18614	3.41E-05	0.00066655	0.00051014	28
BP	GO:2000379	positive regulation of reactive oxygen species metabolic process	20/2016	70/18614	3.46E-05	0.00067482	0.00051647	20
BP	GO:0051402	neuron apoptotic process	50/2016	259/18614	3.48E-05	0.00067691	0.00051807	50
BP	GO:0006865	amino acid transport	33/2016	147/18614	3.52E-05	0.00068372	0.00052328	33
BP	GO:0044409	entry into host	35/2016	160/18614	3.69E-05	0.0007142	0.00054661	35
BP	GO:0033674	positive regulation of kinase activity	74/2016	430/18614	3.69E-05	0.0007142	0.00054661	74
BP	GO:0030888	regulation of B cell proliferation	19/2016	65/18614	3.81E-05	0.0007347	0.00056229	19
BP	GO:1903035	negative regulation of response to wounding	24/2016	93/18614	3.83E-05	0.00073697	0.00056403	24
BP	GO:0035235	ionotropic glutamate receptor signaling pathway	11/2016	26/18614	3.84E-05	0.00073697	0.00056403	11
BP	GO:1903859	regulation of dendrite extension	11/2016	26/18614	3.84E-05	0.00073697	0.00056403	11
BP	GO:0046683	response to organophosphorus	30/2016	129/18614	3.89E-05	0.00074464	0.00056991	30

BP	GO:0002428	antigen processing and presentation of peptide antigen via MHC class Ib	9/2016	18/18614	3.93E-05	0.00074732	0.00057195	9
BP	GO:0010755	regulation of plasminogen activation	9/2016	18/18614	3.93E-05	0.00074732	0.00057195	9
BP	GO:0090594	inflammatory response to wounding	9/2016	18/18614	3.93E-05	0.00074732	0.00057195	9
BP	GO:0021549	cerebellum development	26/2016	105/18614	4.02E-05	0.00076264	0.00058368	26
BP	GO:0007088	regulation of mitotic nuclear division	28/2016	117/18614	4.03E-05	0.00076264	0.00058368	28
BP	GO:0031629	synaptic vesicle fusion to presynaptic active zone membrane	10/2016	22/18614	4.11E-05	0.00077646	0.00059426	10
BP	GO:0051346	negative regulation of hydrolase activity	62/2016	345/18614	4.24E-05	0.00079896	0.00061148	62
BP	GO:0008217	regulation of blood pressure	39/2016	187/18614	4.28E-05	0.00080532	0.00061635	39
BP	GO:0001822	kidney development	57/2016	310/18614	4.40E-05	0.00082689	0.00063286	57
BP	GO:0099068	postsynapse assembly	14/2016	40/18614	4.42E-05	0.00082734	0.0006332	14
BP	GO:1903524	positive regulation of blood circulation	14/2016	40/18614	4.42E-05	0.00082734	0.0006332	14
BP	GO:0002269	leukocyte activation involved in inflammatory response	16/2016	50/18614	4.56E-05	0.00085084	0.00065119	16
BP	GO:0045685	regulation of glial cell differentiation	21/2016	77/18614	4.77E-05	0.00088901	0.0006804	21
BP	GO:0060537	muscle tissue development	73/2016	426/18614	4.79E-05	0.00089135	0.00068219	73
BP	GO:0060292	long-term synaptic depression	12/2016	31/18614	4.94E-05	0.00091691	0.00070175	12
BP	GO:0002791	regulation of peptide secretion	40/2016	195/18614	5.09E-05	0.00094229	0.00072117	40
BP	GO:0006941	striated muscle contraction	38/2016	182/18614	5.19E-05	0.00096014	0.00073483	38
BP	GO:0021700	developmental maturation	58/2016	319/18614	5.27E-05	0.00096786	0.00074074	58
BP	GO:1905517	macrophage migration	18/2016	61/18614	5.27E-05	0.00096786	0.00074074	18
BP	GO:0007178	transmembrane receptor protein serine/threonine kinase signaling pathway	69/2016	398/18614	5.27E-05	0.00096786	0.00074074	69
BP	GO:0071674	mononuclear cell migration	41/2016	202/18614	5.31E-05	0.00097407	0.0007455	41
BP	GO:0010466	negative regulation of peptidase activity	46/2016	236/18614	5.51E-05	0.00100756	0.00077113	46
BP	GO:0045071	negative regulation of viral genome replication	17/2016	56/18614	5.67E-05	0.00103419	0.00079151	17
BP	GO:0097484	dendrite extension	13/2016	36/18614	5.67E-05	0.00103419	0.00079151	13
BP	GO:0034612	response to tumor necrosis factor	49/2016	257/18614	5.76E-05	0.00104852	0.00080247	49
BP	GO:0019228	neuronal action potential	11/2016	27/18614	5.85E-05	0.00105543	0.00080777	11

BP	GO:0042730	fibrinolysis	11/2016	27/18614	5.85E-05	0.00105543	0.00080777	11
BP	GO:0048499	synaptic vesicle membrane organization	11/2016	27/18614	5.85E-05	0.00105543	0.00080777	11
BP	GO:0060384	innervation	11/2016	27/18614	5.85E-05	0.00105543	0.00080777	11
BP	GO:0002220	innate immune response activating cell surface receptor signaling pathway	21/2016	78/18614	5.87E-05	0.00105543	0.00080777	21
BP	GO:0050905	neuromuscular process	34/2016	157/18614	5.87E-05	0.00105543	0.00080777	34
BP	GO:0002468	dendritic cell antigen processing and presentation	8/2016	15/18614	5.96E-05	0.00106469	0.00081485	8
BP	GO:0098883	synapse pruning	8/2016	15/18614	5.96E-05	0.00106469	0.00081485	8
BP	GO:1900426	positive regulation of defense response to bacterium	8/2016	15/18614	5.96E-05	0.00106469	0.00081485	8
BP	GO:0032964	collagen biosynthetic process	16/2016	51/18614	5.98E-05	0.00106638	0.00081615	16
BP	GO:0002455	humoral immune response mediated by circulating immunoglobulin	15/2016	46/18614	6.13E-05	0.00108878	0.00083329	15
BP	GO:0042088	T-helper 1 type immune response	15/2016	46/18614	6.13E-05	0.00108878	0.00083329	15
BP	GO:0003018	vascular process in circulatory system	50/2016	265/18614	6.43E-05	0.00114044	0.00087283	50
BP	GO:0090087	regulation of peptide transport	40/2016	197/18614	6.46E-05	0.00114301	0.00087479	40
BP	GO:0051783	regulation of nuclear division	32/2016	145/18614	6.52E-05	0.0011524	0.00088198	32
BP	GO:0099500	vesicle fusion to plasma membrane	10/2016	23/18614	6.56E-05	0.00115741	0.00088582	10
BP	GO:0046637	regulation of alpha-beta T cell differentiation	20/2016	73/18614	6.68E-05	0.00117582	0.0008999	20
BP	GO:0002283	neutrophil activation involved in immune response	9/2016	19/18614	6.75E-05	0.00117871	0.00090212	9
BP	GO:0016082	synaptic vesicle priming	9/2016	19/18614	6.75E-05	0.00117871	0.00090212	9
BP	GO:0031643	positive regulation of myelination	9/2016	19/18614	6.75E-05	0.00117871	0.00090212	9
BP	GO:0032026	response to magnesium ion	9/2016	19/18614	6.75E-05	0.00117871	0.00090212	9
BP	GO:0019883	antigen processing and presentation of endogenous antigen	12/2016	32/18614	7.13E-05	0.00124291	0.00095125	12
BP	GO:0048880	sensory system development	68/2016	395/18614	7.42E-05	0.00129184	0.0009887	68
BP	GO:0098739	import across plasma membrane	42/2016	212/18614	7.75E-05	0.00134645	0.0010305	42

BP	GO:0046638	positive regulation of alpha-beta T cell differentiation	16/2016	52/18614	7.77E-05	0.0013476	0.00103138	16
BP	GO:0022037	metencephalon development	27/2016	115/18614	7.81E-05	0.00135057	0.00103365	27
BP	GO:0045165	cell fate commitment	52/2016	281/18614	7.85E-05	0.00135621	0.00103796	52
BP	GO:1990778	protein localization to cell periphery	61/2016	345/18614	7.89E-05	0.00135935	0.00104037	61
BP	GO:0045112	integrin biosynthetic process	5/2016	6/18614	8.10E-05	0.00138671	0.00106131	5
BP	GO:0099525	presynaptic dense core vesicle exocytosis	5/2016	6/18614	8.10E-05	0.00138671	0.00106131	5
BP	GO:0001774	microglial cell activation	15/2016	47/18614	8.11E-05	0.00138671	0.00106131	15
BP	GO:1990573	potassium ion import across plasma membrane	15/2016	47/18614	8.11E-05	0.00138671	0.00106131	15
BP	GO:0031960	response to corticosteroid	35/2016	166/18614	8.19E-05	0.00139896	0.00107068	35
BP	GO:0150063	visual system development	67/2016	389/18614	8.23E-05	0.00140303	0.0010738	67
BP	GO:0031638	zymogen activation	18/2016	63/18614	8.41E-05	0.00142583	0.00109125	18
BP	GO:0032757	positive regulation of interleukin-8 production	18/2016	63/18614	8.41E-05	0.00142583	0.00109125	18
BP	GO:0051965	positive regulation of synapse assembly	18/2016	63/18614	8.41E-05	0.00142583	0.00109125	18
BP	GO:0006937	regulation of muscle contraction	36/2016	173/18614	8.68E-05	0.00146254	0.00111934	36
BP	GO:0030194	positive regulation of blood coagulation	11/2016	28/18614	8.70E-05	0.00146254	0.00111934	11
BP	GO:0050951	sensory perception of temperature stimulus	11/2016	28/18614	8.70E-05	0.00146254	0.00111934	11
BP	GO:1900048	positive regulation of hemostasis	11/2016	28/18614	8.70E-05	0.00146254	0.00111934	11
BP	GO:0071214	cellular response to abiotic stimulus	60/2016	339/18614	8.73E-05	0.00146254	0.00111934	60
BP	GO:0104004	cellular response to environmental stimulus	60/2016	339/18614	8.73E-05	0.00146254	0.00111934	60
BP	GO:0010951	negative regulation of endopeptidase activity	34/2016	160/18614	8.74E-05	0.00146254	0.00111934	34
BP	GO:0002292	T cell differentiation involved in immune response	21/2016	80/18614	8.77E-05	0.00146416	0.00112059	21
BP	GO:0016358	dendrite development	45/2016	234/18614	9.19E-05	0.00153307	0.00117332	45
BP	GO:0042093	T-helper cell differentiation	19/2016	69/18614	9.36E-05	0.00155737	0.00119192	19
BP	GO:0071868	cellular response to monoamine stimulus	25/2016	104/18614	9.44E-05	0.00156581	0.00119838	25
BP	GO:0071870	cellular response to catecholamine stimulus	25/2016	104/18614	9.44E-05	0.00156581	0.00119838	25
BP	GO:0030100	regulation of endocytosis	42/2016	214/18614	9.67E-05	0.00160114	0.00122542	42
BP	GO:0099172	presynapse organization	16/2016	53/18614	0.00010027	0.00164802	0.0012613	16

BP	GO:0007196	adenylate cyclase-inhibiting G protein-coupled glutamate receptor signaling pathway	6/2016	9/18614	0.00010069	0.00164802	0.0012613	6
BP	GO:0060385	axonogenesis involved in innervation	6/2016	9/18614	0.00010069	0.00164802	0.0012613	6
BP	GO:0099640	axo-dendritic protein transport	6/2016	9/18614	0.00010069	0.00164802	0.0012613	6
BP	GO:0099641	anterograde axonal protein transport	6/2016	9/18614	0.00010069	0.00164802	0.0012613	6
BP	GO:2001187	positive regulation of CD8-positive, alpha-beta T cell activation	6/2016	9/18614	0.00010069	0.00164802	0.0012613	6
BP	GO:0002381	immunoglobulin production involved in immunoglobulin-mediated immune response	20/2016	75/18614	0.00010099	0.00164802	0.0012613	20
BP	GO:0045669	positive regulation of osteoblast differentiation	20/2016	75/18614	0.00010099	0.00164802	0.0012613	20
BP	GO:0006213	pyrimidine nucleoside metabolic process	10/2016	24/18614	0.00010163	0.00165565	0.00126714	10
BP	GO:0071356	cellular response to tumor necrosis factor	45/2016	235/18614	0.00010201	0.00165891	0.00126964	45
BP	GO:0043524	negative regulation of neuron apoptotic process	33/2016	155/18614	0.00010605	0.00171777	0.00131468	33
BP	GO:0006953	acute-phase response	15/2016	48/18614	0.00010619	0.00171777	0.00131468	15
BP	GO:0031641	regulation of myelination	15/2016	48/18614	0.00010619	0.00171777	0.00131468	15
BP	GO:0055074	calcium ion homeostasis	58/2016	327/18614	0.0001069	0.00172608	0.00132104	58
BP	GO:0038065	collagen-activated signaling pathway	8/2016	16/18614	0.00010783	0.00172958	0.00132372	8
BP	GO:0042448	progesterone metabolic process	8/2016	16/18614	0.00010783	0.00172958	0.00132372	8
BP	GO:0046135	pyrimidine nucleoside catabolic process	8/2016	16/18614	0.00010783	0.00172958	0.00132372	8
BP	GO:0001662	behavioral fear response	13/2016	38/18614	0.00010805	0.00172958	0.00132372	13
BP	GO:0097106	postsynaptic density organization	13/2016	38/18614	0.00010805	0.00172958	0.00132372	13
BP	GO:0045687	positive regulation of glial cell differentiation	14/2016	43/18614	0.00010925	0.0017458	0.00133613	14
BP	GO:0098659	inorganic cation import across plasma membrane	30/2016	136/18614	0.0001097	0.00174688	0.00133696	30
BP	GO:0099587	inorganic ion import across plasma membrane	30/2016	136/18614	0.0001097	0.00174688	0.00133696	30
BP	GO:0002643	regulation of tolerance induction	9/2016	20/18614	0.00011093	0.00176337	0.00134958	9
BP	GO:0001649	osteoblast differentiation	47/2016	250/18614	0.00011483	0.00182229	0.00139468	47

BP	GO:0048638	regulation of developmental growth	58/2016	328/18614	0.00011644	0.00184459	0.00141175	58
BP	GO:0031102	neuron projection regeneration	17/2016	59/18614	0.00011712	0.00185214	0.00141752	17
BP	GO:0014074	response to purine-containing compound	31/2016	143/18614	0.00011947	0.00188384	0.00144178	31
BP	GO:0048568	embryonic organ development	75/2016	453/18614	0.00011953	0.00188384	0.00144178	75
BP	GO:0032411	positive regulation of transporter activity	28/2016	124/18614	0.00011991	0.00188657	0.00144388	28
BP	GO:0045089	positive regulation of innate immune response	54/2016	300/18614	0.00012298	0.00193157	0.00147832	54
BP	GO:0150076	neuroinflammatory response	20/2016	76/18614	0.00012327	0.00193282	0.00147927	20
BP	GO:0052548	regulation of endopeptidase activity	59/2016	336/18614	0.00012452	0.0019491	0.00149173	59
BP	GO:0001773	myeloid dendritic cell activation	11/2016	29/18614	0.0001265	0.00197347	0.00151038	11
BP	GO:0015813	L-glutamate transmembrane transport	11/2016	29/18614	0.0001265	0.00197347	0.00151038	11
BP	GO:0071320	cellular response to cAMP	16/2016	54/18614	0.0001283	0.00199472	0.00152664	16
BP	GO:1904645	response to amyloid-beta	16/2016	54/18614	0.0001283	0.00199472	0.00152664	16
BP	GO:1901890	positive regulation of cell junction assembly	25/2016	106/18614	0.00013095	0.00203247	0.00155554	25
BP	GO:0009306	protein secretion	64/2016	373/18614	0.00013144	0.00203661	0.00155871	64
BP	GO:0002605	negative regulation of dendritic cell antigen processing and presentation	4/2016	4/18614	0.00013723	0.00211215	0.00161652	4
BP	GO:0046958	nonassociative learning	4/2016	4/18614	0.00013723	0.00211215	0.00161652	4
BP	GO:0060300	regulation of cytokine activity	4/2016	4/18614	0.00013723	0.00211215	0.00161652	4
BP	GO:0150062	complement-mediated synapse pruning	4/2016	4/18614	0.00013723	0.00211215	0.00161652	4
BP	GO:0006911	phagocytosis, engulfment	15/2016	49/18614	0.00013786	0.00211472	0.00161848	15
BP	GO:0099054	presynapse assembly	15/2016	49/18614	0.00013786	0.00211472	0.00161848	15
BP	GO:0032743	positive regulation of interleukin-2 production	12/2016	34/18614	0.00014088	0.00213973	0.00163763	12
BP	GO:0043372	positive regulation of CD4-positive, alpha-beta T cell differentiation	12/2016	34/18614	0.00014088	0.00213973	0.00163763	12
BP	GO:0060074	synapse maturation	12/2016	34/18614	0.00014088	0.00213973	0.00163763	12
BP	GO:0060317	cardiac epithelial to mesenchymal transition	12/2016	34/18614	0.00014088	0.00213973	0.00163763	12
BP	GO:0098664	G protein-coupled serotonin receptor signaling pathway	12/2016	34/18614	0.00014088	0.00213973	0.00163763	12

BP	GO:0099171	presynaptic modulation of chemical synaptic transmission	12/2016	34/18614	0.00014088	0.00213973	0.00163763	12
BP	GO:0002294	CD4-positive, alpha-beta T cell differentiation involved in immune response	19/2016	71/18614	0.00014192	0.00215202	0.00164703	19
BP	GO:0035592	establishment of protein localization to extracellular region	64/2016	374/18614	0.00014217	0.00215218	0.00164715	64
BP	GO:0042596	fear response	14/2016	44/18614	0.0001444	0.00217876	0.0016675	14
BP	GO:2000516	positive regulation of CD4-positive, alpha-beta T cell activation	14/2016	44/18614	0.0001444	0.00217876	0.0016675	14
BP	GO:0060048	cardiac muscle contraction	30/2016	138/18614	0.00014475	0.00218046	0.0016688	30
BP	GO:0002209	behavioral defense response	13/2016	39/18614	0.00014612	0.00219399	0.00167916	13
BP	GO:0007210	serotonin receptor signaling pathway	13/2016	39/18614	0.00014612	0.00219399	0.00167916	13
BP	GO:0097696	receptor signaling pathway via STAT	37/2016	184/18614	0.00014668	0.00219882	0.00168285	37
BP	GO:0021545	cranial nerve development	17/2016	60/18614	0.0001471	0.00220153	0.00168492	17
BP	GO:0032414	positive regulation of ion transmembrane transporter activity	26/2016	113/18614	0.00014948	0.00223325	0.0017092	26
BP	GO:0071692	protein localization to extracellular region	65/2016	382/18614	0.00014971	0.00223325	0.0017092	65
BP	GO:0051260	protein homooligomerization	38/2016	191/18614	0.00015156	0.00225727	0.00172758	38
BP	GO:0016048	detection of temperature stimulus	10/2016	25/18614	0.00015301	0.00227509	0.00174122	10
BP	GO:0071867	response to monoamine	25/2016	107/18614	0.00015355	0.00227585	0.0017418	25
BP	GO:0071869	response to catecholamine	25/2016	107/18614	0.00015355	0.00227585	0.0017418	25
BP	GO:0061337	cardiac conduction	24/2016	101/18614	0.00015644	0.00231485	0.00177165	24
BP	GO:0060538	skeletal muscle organ development	36/2016	178/18614	0.00015883	0.00234643	0.00179582	36
BP	GO:0006874	intracellular calcium ion homeostasis	54/2016	303/18614	0.00016017	0.00235698	0.0018039	54
BP	GO:0006022	aminoglycan metabolic process	28/2016	126/18614	0.00016031	0.00235698	0.0018039	28
BP	GO:0045471	response to ethanol	28/2016	126/18614	0.00016031	0.00235698	0.0018039	28
BP	GO:0061900	glial cell activation	16/2016	55/18614	0.00016293	0.0023917	0.00183047	16
BP	GO:0033605	positive regulation of catecholamine secretion	7/2016	13/18614	0.00016427	0.00239612	0.00183385	7
BP	GO:0070486	leukocyte aggregation	7/2016	13/18614	0.00016427	0.00239612	0.00183385	7

BP	GO:0070493	thrombin-activated receptor signaling pathway	7/2016	13/18614	0.00016427	0.00239612	0.00183385	7
BP	GO:1903054	negative regulation of extracellular matrix organization	7/2016	13/18614	0.00016427	0.00239612	0.00183385	7
BP	GO:0032355	response to estradiol	27/2016	120/18614	0.00016749	0.00243924	0.00186686	27
BP	GO:0090257	regulation of muscle system process	47/2016	254/18614	0.00016948	0.00246202	0.00188429	47
BP	GO:0090276	regulation of peptide hormone secretion	38/2016	192/18614	0.00016959	0.00246202	0.00188429	38
BP	GO:0002287	alpha-beta T cell activation involved in immune response	19/2016	72/18614	0.00017347	0.00251035	0.00192128	19
BP	GO:0002293	alpha-beta T cell differentiation involved in immune response	19/2016	72/18614	0.00017347	0.00251035	0.00192128	19
BP	GO:0060004	reflex	9/2016	21/18614	0.00017551	0.00253599	0.0019409	9
BP	GO:1902041	regulation of extrinsic apoptotic signaling pathway via death domain receptors	15/2016	50/18614	0.00017741	0.00255932	0.00195875	15
BP	GO:0002507	tolerance induction	11/2016	30/18614	0.0001803	0.00258891	0.0019814	11
BP	GO:0050820	positive regulation of coagulation	11/2016	30/18614	0.0001803	0.00258891	0.0019814	11
BP	GO:0051938	L-glutamate import	11/2016	30/18614	0.0001803	0.00258891	0.0019814	11
BP	GO:0061045	negative regulation of wound healing	20/2016	78/18614	0.00018118	0.00259753	0.001988	20
BP	GO:0019229	regulation of vasoconstriction	17/2016	61/18614	0.00018359	0.00261434	0.00200086	17
BP	GO:0034113	heterotypic cell-cell adhesion	17/2016	61/18614	0.00018359	0.00261434	0.00200086	17
BP	GO:0030316	osteoclast differentiation	24/2016	102/18614	0.00018387	0.00261434	0.00200086	24
BP	GO:0034112	positive regulation of homotypic cell-cell adhesion	8/2016	17/18614	0.00018434	0.00261434	0.00200086	8
BP	GO:0034138	toll-like receptor 3 signaling pathway	8/2016	17/18614	0.00018434	0.00261434	0.00200086	8
BP	GO:0098712	L-glutamate import across plasma membrane	8/2016	17/18614	0.00018434	0.00261434	0.00200086	8
BP	GO:0098877	neurotransmitter receptor transport to plasma membrane	8/2016	17/18614	0.00018434	0.00261434	0.00200086	8
BP	GO:0001704	formation of primary germ layer	28/2016	127/18614	0.00018474	0.00261585	0.00200202	28
BP	GO:0002690	positive regulation of leukocyte chemotaxis	23/2016	96/18614	0.00018659	0.00263493	0.00201663	23
BP	GO:0051259	protein complex oligomerization	46/2016	248/18614	0.00018666	0.00263493	0.00201663	46

BP	GO:1903351	cellular response to dopamine	22/2016	90/18614	0.00018733	0.00264036	0.00202078	22
BP	GO:0048813	dendrite morphogenesis	30/2016	140/18614	0.00018949	0.00266678	0.002041	30
BP	GO:0007259	receptor signaling pathway via JAK-STAT	35/2016	173/18614	0.00019324	0.00271351	0.00207676	35
BP	GO:1990806	ligand-gated ion channel signaling pathway	12/2016	35/18614	0.0001934	0.00271351	0.00207676	12
BP	GO:0001916	positive regulation of T cell mediated cytotoxicity	13/2016	40/18614	0.00019519	0.00273437	0.00209273	13
BP	GO:0002702	positive regulation of production of molecular mediator of immune response	29/2016	134/18614	0.00020101	0.00281176	0.00215196	29
BP	GO:0033002	muscle cell proliferation	46/2016	249/18614	0.0002054	0.00286869	0.00219553	46
BP	GO:0003170	heart valve development	19/2016	73/18614	0.00021099	0.00293793	0.00224852	19
BP	GO:0070527	platelet aggregation	19/2016	73/18614	0.00021099	0.00293793	0.00224852	19
BP	GO:0032677	regulation of interleukin-8 production	24/2016	103/18614	0.00021548	0.00299591	0.0022929	24
BP	GO:0042310	vasoconstriction	20/2016	79/18614	0.00021823	0.00302955	0.00231865	20
BP	GO:0060993	kidney morphogenesis	23/2016	97/18614	0.00021988	0.0030433	0.00232916	23
BP	GO:0099175	regulation of postsynapse organization	23/2016	97/18614	0.00021988	0.0030433	0.00232916	23
BP	GO:1903350	response to dopamine	22/2016	91/18614	0.00022214	0.00306999	0.00234959	22
BP	GO:0010810	regulation of cell-substrate adhesion	42/2016	222/18614	0.00022419	0.00309023	0.00236508	42
BP	GO:0001911	negative regulation of leukocyte mediated cytotoxicity	10/2016	26/18614	0.00022462	0.00309023	0.00236508	10
BP	GO:0046629	gamma-delta T cell activation	10/2016	26/18614	0.00022462	0.00309023	0.00236508	10
BP	GO:0090303	positive regulation of wound healing	17/2016	62/18614	0.00022772	0.00311207	0.0023818	17
BP	GO:0002138	retinoic acid biosynthetic process	6/2016	10/18614	0.00022857	0.00311207	0.0023818	6
BP	GO:0002291	T cell activation via T cell receptor contact with antigen bound to MHC molecule on antigen presenting cell	6/2016	10/18614	0.00022857	0.00311207	0.0023818	6
BP	GO:0044341	sodium-dependent phosphate transport	6/2016	10/18614	0.00022857	0.00311207	0.0023818	6
BP	GO:0051933	amino acid neurotransmitter reuptake	6/2016	10/18614	0.00022857	0.00311207	0.0023818	6
BP	GO:0060075	regulation of resting membrane potential	6/2016	10/18614	0.00022857	0.00311207	0.0023818	6
BP	GO:1990504	dense core granule exocytosis	6/2016	10/18614	0.00022857	0.00311207	0.0023818	6
BP	GO:0072009	nephron epithelium development	26/2016	116/18614	0.00023398	0.00318102	0.00243457	26

BP	GO:0008037	cell recognition	32/2016	155/18614	0.00024284	0.00329658	0.00252301	32
BP	GO:0048706	embryonic skeletal system development	28/2016	129/18614	0.00024374	0.00330393	0.00252864	28
BP	GO:2000300	regulation of synaptic vesicle exocytosis	14/2016	46/18614	0.00024474	0.00331262	0.00253529	14
BP	GO:0009582	detection of abiotic stimulus	30/2016	142/18614	0.00024618	0.00332235	0.00254274	30
BP	GO:0051384	response to glucocorticoid	30/2016	142/18614	0.00024618	0.00332235	0.00254274	30
BP	GO:0048732	gland development	72/2016	441/18614	0.00024868	0.00335111	0.00256475	72
BP	GO:0046942	carboxylic acid transport	60/2016	352/18614	0.00025143	0.00337511	0.00258311	60
BP	GO:0032637	interleukin-8 production	24/2016	104/18614	0.0002518	0.00337511	0.00258311	24
BP	GO:0060291	long-term synaptic potentiation	24/2016	104/18614	0.0002518	0.00337511	0.00258311	24
BP	GO:0086011	membrane repolarization during action potential	11/2016	31/18614	0.00025229	0.00337511	0.00258311	11
BP	GO:0097205	renal filtration	11/2016	31/18614	0.00025229	0.00337511	0.00258311	11
BP	GO:0045123	cellular extravasation	19/2016	74/18614	0.00025543	0.00341222	0.00261152	19
BP	GO:0002691	regulation of cellular extravasation	13/2016	41/18614	0.00025772	0.00342079	0.00261807	13
BP	GO:1901381	positive regulation of potassium ion transmembrane transport	13/2016	41/18614	0.00025772	0.00342079	0.00261807	13
BP	GO:0014060	regulation of epinephrine secretion	5/2016	7/18614	0.00025805	0.00342079	0.00261807	5
BP	GO:0048242	epinephrine secretion	5/2016	7/18614	0.00025805	0.00342079	0.00261807	5
BP	GO:0098914	membrane repolarization during atrial cardiac muscle cell action potential	5/2016	7/18614	0.00025805	0.00342079	0.00261807	5
BP	GO:0043279	response to alkaloid	23/2016	98/18614	0.00025831	0.00342079	0.00261807	23
BP	GO:0002474	antigen processing and presentation of peptide antigen via MHC class I	12/2016	36/18614	0.00026173	0.00345619	0.00264517	12
BP	GO:1905144	response to acetylcholine	12/2016	36/18614	0.00026173	0.00345619	0.00264517	12
BP	GO:0002028	regulation of sodium ion transport	22/2016	92/18614	0.00026253	0.00345689	0.0026457	22
BP	GO:1901216	positive regulation of neuron death	22/2016	92/18614	0.00026253	0.00345689	0.0026457	22
BP	GO:0033630	positive regulation of cell adhesion mediated by integrin	9/2016	22/18614	0.00026858	0.0035264	0.0026989	9
BP	GO:0097107	postsynaptic density assembly	9/2016	22/18614	0.00026858	0.0035264	0.0026989	9
BP	GO:0015849	organic acid transport	60/2016	353/18614	0.00027163	0.0035614	0.00272569	60
BP	GO:0051961	negative regulation of nervous system development	32/2016	156/18614	0.00027435	0.00359186	0.002749	32

BP	GO:0050673	epithelial cell proliferation	78/2016	488/18614	0.00027759	0.00362922	0.0027776	78
BP	GO:0010977	negative regulation of neuron projection development	30/2016	143/18614	0.00027982	0.00364797	0.00279194	30
BP	GO:1903900	regulation of viral life cycle	30/2016	143/18614	0.00027982	0.00364797	0.00279194	30
BP	GO:0001885	endothelial cell development	17/2016	63/18614	0.00028079	0.00365539	0.00279763	17
BP	GO:0050796	regulation of insulin secretion	33/2016	163/18614	0.00028622	0.00372088	0.00284774	33
BP	GO:0048013	ephrin receptor signaling pathway	15/2016	52/18614	0.00028669	0.00372166	0.00284835	15
BP	GO:0014015	positive regulation of gliogenesis	18/2016	69/18614	0.00029747	0.00382302	0.00292592	18
BP	GO:0043301	negative regulation of leukocyte degranulation	7/2016	14/18614	0.00029782	0.00382302	0.00292592	7
BP	GO:0046131	pyrimidine ribonucleoside metabolic process	7/2016	14/18614	0.00029782	0.00382302	0.00292592	7
BP	GO:0046133	pyrimidine ribonucleoside catabolic process	7/2016	14/18614	0.00029782	0.00382302	0.00292592	7
BP	GO:0086070	SA node cell to atrial cardiac muscle cell communication	7/2016	14/18614	0.00029782	0.00382302	0.00292592	7
BP	GO:0098814	spontaneous synaptic transmission	7/2016	14/18614	0.00029782	0.00382302	0.00292592	7
BP	GO:0098840	protein transport along microtubule	7/2016	14/18614	0.00029782	0.00382302	0.00292592	7
BP	GO:0099118	microtubule-based protein transport	7/2016	14/18614	0.00029782	0.00382302	0.00292592	7
BP	GO:0032602	chemokine production	23/2016	99/18614	0.00030252	0.00387258	0.00296385	23
BP	GO:0048661	positive regulation of smooth muscle cell proliferation	23/2016	99/18614	0.00030252	0.00387258	0.00296385	23
BP	GO:0048754	branching morphogenesis of an epithelial tube	32/2016	157/18614	0.00030944	0.00395567	0.00302744	32
BP	GO:1905314	semi-lunar valve development	14/2016	47/18614	0.00031418	0.0040107	0.00306956	14
BP	GO:0032612	interleukin-1 production	28/2016	131/18614	0.0003189	0.00405525	0.00310365	28
BP	GO:0032652	regulation of interleukin-1 production	28/2016	131/18614	0.0003189	0.00405525	0.00310365	28
BP	GO:0006956	complement activation	16/2016	58/18614	0.00031987	0.00405525	0.00310365	16
BP	GO:0099024	plasma membrane invagination	16/2016	58/18614	0.00031987	0.00405525	0.00310365	16
BP	GO:2000649	regulation of sodium ion transmembrane transporter activity	16/2016	58/18614	0.00031987	0.00405525	0.00310365	16

BP	GO:0001654	eye development	64/2016	385/18614	0.00032547	0.00412057	0.00315365	64
BP	GO:0007264	small GTPase mediated signal transduction	77/2016	483/18614	0.00032828	0.00415037	0.00317645	77
BP	GO:0002377	immunoglobulin production	40/2016	212/18614	0.00033072	0.00417547	0.00319566	40
BP	GO:0032733	positive regulation of interleukin-10 production	13/2016	42/18614	0.00033659	0.00423794	0.00324348	13
BP	GO:0086091	regulation of heart rate by cardiac conduction	13/2016	42/18614	0.00033659	0.00423794	0.00324348	13
BP	GO:0051482	positive regulation of cytosolic calcium ion concentration involved in phospholipase C-activating G protein-coupled signaling pathway	11/2016	32/18614	0.00034709	0.00436427	0.00334016	11
BP	GO:0034110	regulation of homotypic cell-cell adhesion	12/2016	37/18614	0.00034951	0.00437077	0.00334514	12
BP	GO:0044319	wound healing, spreading of cells	12/2016	37/18614	0.00034951	0.00437077	0.00334514	12
BP	GO:0045920	negative regulation of exocytosis	12/2016	37/18614	0.00034951	0.00437077	0.00334514	12
BP	GO:0090505	epiboly involved in wound healing	12/2016	37/18614	0.00034951	0.00437077	0.00334514	12
BP	GO:0061326	renal tubule development	23/2016	100/18614	0.00035325	0.00441151	0.00337631	23
BP	GO:0019058	viral life cycle	55/2016	320/18614	0.00036086	0.00449434	0.00343971	55
BP	GO:0060485	mesenchyme development	55/2016	320/18614	0.00036086	0.00449434	0.00343971	55
BP	GO:0086003	cardiac muscle cell contraction	19/2016	76/18614	0.00036936	0.00458514	0.0035092	19
BP	GO:1903036	positive regulation of response to wounding	19/2016	76/18614	0.00036936	0.00458514	0.0035092	19
BP	GO:0033273	response to vitamin	21/2016	88/18614	0.00036964	0.00458514	0.0035092	21
BP	GO:0050848	regulation of calcium-mediated signaling	20/2016	82/18614	0.00037207	0.00460907	0.00352752	20
BP	GO:0050954	sensory perception of mechanical stimulus	35/2016	179/18614	0.00038115	0.00471516	0.00360871	35
BP	GO:0009581	detection of external stimulus	29/2016	139/18614	0.00038628	0.0047722	0.00365236	29
BP	GO:0019885	antigen processing and presentation of endogenous peptide antigen via MHC class I	9/2016	23/18614	0.00039904	0.00490008	0.00375024	9
BP	GO:0072243	metanephric nephron epithelium development	9/2016	23/18614	0.00039904	0.00490008	0.00375024	9
BP	GO:1900120	regulation of receptor binding	9/2016	23/18614	0.00039904	0.00490008	0.00375024	9
BP	GO:0007520	myoblast fusion	14/2016	48/18614	0.00039982	0.00490008	0.00375024	14
BP	GO:0035094	response to nicotine	14/2016	48/18614	0.00039982	0.00490008	0.00375024	14

BP	GO:0043268	positive regulation of potassium ion transport	14/2016	48/18614	0.00039982	0.00490008	0.00375024	14
BP	GO:0048857	neural nucleus development	17/2016	65/18614	0.00041976	0.00513764	0.00393206	17
BP	GO:0002548	monocyte chemotaxis	18/2016	71/18614	0.00043465	0.00531221	0.00406566	18
BP	GO:0060191	regulation of lipase activity	21/2016	89/18614	0.00043518	0.00531221	0.00406566	21
BP	GO:0014068	positive regulation of phosphatidylinositol 3-kinase signaling	20/2016	83/18614	0.00044103	0.0053694	0.00410943	20
BP	GO:0050871	positive regulation of B cell activation	20/2016	83/18614	0.00044103	0.0053694	0.00410943	20
BP	GO:1901654	response to ketone	39/2016	208/18614	0.00044381	0.00539603	0.00412981	39
BP	GO:0050819	negative regulation of coagulation	15/2016	54/18614	0.00044949	0.00545075	0.00417169	15
BP	GO:0086002	cardiac muscle cell action potential involved in contraction	15/2016	54/18614	0.00044949	0.00545075	0.00417169	15
BP	GO:0060402	calcium ion transport into cytosol	10/2016	28/18614	0.00045305	0.00547321	0.00418888	10
BP	GO:0150146	cell junction disassembly	10/2016	28/18614	0.00045305	0.00547321	0.00418888	10
BP	GO:0002604	regulation of dendritic cell antigen processing and presentation	6/2016	11/18614	0.0004567	0.00547321	0.00418888	6
BP	GO:0007614	short-term memory	6/2016	11/18614	0.0004567	0.00547321	0.00418888	6
BP	GO:0016102	diterpenoid biosynthetic process	6/2016	11/18614	0.0004567	0.00547321	0.00418888	6
BP	GO:0031340	positive regulation of vesicle fusion	6/2016	11/18614	0.0004567	0.00547321	0.00418888	6
BP	GO:0060081	membrane hyperpolarization	6/2016	11/18614	0.0004567	0.00547321	0.00418888	6
BP	GO:0099624	atrial cardiac muscle cell membrane repolarization	6/2016	11/18614	0.0004567	0.00547321	0.00418888	6
BP	GO:1905383	protein localization to presynapse	6/2016	11/18614	0.0004567	0.00547321	0.00418888	6
BP	GO:0002335	mature B cell differentiation	12/2016	38/18614	0.00046095	0.0054956	0.00420601	12
BP	GO:0002369	T cell cytokine production	12/2016	38/18614	0.00046095	0.0054956	0.00420601	12
BP	GO:0002724	regulation of T cell cytokine production	12/2016	38/18614	0.00046095	0.0054956	0.00420601	12
BP	GO:0090504	epiboly	12/2016	38/18614	0.00046095	0.0054956	0.00420601	12
BP	GO:0014048	regulation of glutamate secretion	8/2016	19/18614	0.00046976	0.00555442	0.00425103	8
BP	GO:0086014	atrial cardiac muscle cell action potential	8/2016	19/18614	0.00046976	0.00555442	0.00425103	8
BP	GO:0086026	atrial cardiac muscle cell to AV node cell signaling	8/2016	19/18614	0.00046976	0.00555442	0.00425103	8

BP	GO:0086066	atrial cardiac muscle cell to AV node cell communication	8/2016	19/18614	0.00046976	0.00555442	0.00425103	8
BP	GO:1903818	positive regulation of voltage-gated potassium channel activity	8/2016	19/18614	0.00046976	0.00555442	0.00425103	8
BP	GO:1904862	inhibitory synapse assembly	8/2016	19/18614	0.00046976	0.00555442	0.00425103	8
BP	GO:0006958	complement activation, classical pathway	11/2016	33/18614	0.00047011	0.00555442	0.00425103	11
BP	GO:0031345	negative regulation of cell projection organization	37/2016	195/18614	0.00048477	0.00572028	0.00437797	37
BP	GO:0002758	innate immune response-activating signaling pathway	39/2016	209/18614	0.00048975	0.00577169	0.00441732	39
BP	GO:0140895	cell surface toll-like receptor signaling pathway	14/2016	49/18614	0.00050461	0.00591345	0.00452581	14
BP	GO:0007567	parturition	7/2016	15/18614	0.00050628	0.00591345	0.00452581	7
BP	GO:0043374	CD8-positive, alpha-beta T cell differentiation	7/2016	15/18614	0.00050628	0.00591345	0.00452581	7
BP	GO:0048934	peripheral nervous system neuron differentiation	7/2016	15/18614	0.00050628	0.00591345	0.00452581	7
BP	GO:0048935	peripheral nervous system neuron development	7/2016	15/18614	0.00050628	0.00591345	0.00452581	7
BP	GO:0060099	regulation of phagocytosis, engulfment	7/2016	15/18614	0.00050628	0.00591345	0.00452581	7
BP	GO:1905153	regulation of membrane invagination	7/2016	15/18614	0.00050628	0.00591345	0.00452581	7
BP	GO:0010517	regulation of phospholipase activity	17/2016	66/18614	0.00050913	0.00593923	0.00454554	17
BP	GO:0097305	response to alcohol	45/2016	252/18614	0.00051707	0.00602418	0.00461056	45
BP	GO:0060041	retina development in camera-type eye	31/2016	155/18614	0.00053583	0.00623487	0.00477181	31
BP	GO:0042100	B cell proliferation	23/2016	103/18614	0.00055281	0.00642437	0.00491685	23
BP	GO:0051955	regulation of amino acid transport	13/2016	44/18614	0.000557	0.00646487	0.00494784	13
BP	GO:2000027	regulation of animal organ morphogenesis	27/2016	129/18614	0.00057223	0.00663322	0.00507669	27
BP	GO:0048660	regulation of smooth muscle cell proliferation	34/2016	176/18614	0.00057556	0.00666347	0.00509983	34
BP	GO:0050769	positive regulation of neurogenesis	42/2016	232/18614	0.00058482	0.00676214	0.00517535	42
BP	GO:0043903	regulation of biological process involved in symbiotic interaction	16/2016	61/18614	0.00059295	0.00683899	0.00523417	16
BP	GO:0086065	cell communication involved in cardiac conduction	16/2016	61/18614	0.00059295	0.00683899	0.00523417	16

BP	GO:0007584	response to nutrient	31/2016	156/18614	0.00060068	0.00691946	0.00529576	31
BP	GO:1902305	regulation of sodium ion transmembrane transport	17/2016	67/18614	0.00061438	0.00705266	0.0053977	17
BP	GO:0003094	glomerular filtration	10/2016	29/18614	0.00062491	0.00705266	0.0053977	10
BP	GO:0010996	response to auditory stimulus	10/2016	29/18614	0.00062491	0.00705266	0.0053977	10
BP	GO:0031342	negative regulation of cell killing	10/2016	29/18614	0.00062491	0.00705266	0.0053977	10
BP	GO:0007197	adenylate cyclase-inhibiting G protein-coupled acetylcholine receptor signaling pathway	5/2016	8/18614	0.00062651	0.00705266	0.0053977	5
BP	GO:0010757	negative regulation of plasminogen activation	5/2016	8/18614	0.00062651	0.00705266	0.0053977	5
BP	GO:0043313	regulation of neutrophil degranulation	5/2016	8/18614	0.00062651	0.00705266	0.0053977	5
BP	GO:0043376	regulation of CD8-positive, alpha-beta T cell differentiation	5/2016	8/18614	0.00062651	0.00705266	0.0053977	5
BP	GO:0060600	dichotomous subdivision of an epithelial terminal unit	5/2016	8/18614	0.00062651	0.00705266	0.0053977	5
BP	GO:0061669	spontaneous neurotransmitter secretion	5/2016	8/18614	0.00062651	0.00705266	0.0053977	5
BP	GO:0071314	cellular response to cocaine	5/2016	8/18614	0.00062651	0.00705266	0.0053977	5
BP	GO:1904026	regulation of collagen fibril organization	5/2016	8/18614	0.00062651	0.00705266	0.0053977	5
BP	GO:0002238	response to molecule of fungal origin	4/2016	5/18614	0.00062681	0.00705266	0.0053977	4
BP	GO:0002774	Fc receptor mediated inhibitory signaling pathway	4/2016	5/18614	0.00062681	0.00705266	0.0053977	4
BP	GO:0002835	negative regulation of response to tumor cell	4/2016	5/18614	0.00062681	0.00705266	0.0053977	4
BP	GO:0002838	negative regulation of immune response to tumor cell	4/2016	5/18614	0.00062681	0.00705266	0.0053977	4
BP	GO:0043378	positive regulation of CD8-positive, alpha-beta T cell differentiation	4/2016	5/18614	0.00062681	0.00705266	0.0053977	4
BP	GO:0045113	regulation of integrin biosynthetic process	4/2016	5/18614	0.00062681	0.00705266	0.0053977	4
BP	GO:0099174	regulation of presynapse organization	11/2016	34/18614	0.00062757	0.00705266	0.0053977	11
BP	GO:1905606	regulation of presynapse assembly	11/2016	34/18614	0.00062757	0.00705266	0.0053977	11

BP	GO:0030195	negative regulation of blood coagulation	14/2016	50/18614	0.00063185	0.00709217	0.00542794	14
BP	GO:0070252	actin-mediated cell contraction	23/2016	104/18614	0.00063829	0.00715566	0.00547653	23
BP	GO:0002218	activation of innate immune response	42/2016	233/18614	0.00064028	0.00716929	0.00548696	42
BP	GO:0046578	regulation of Ras protein signal transduction	35/2016	184/18614	0.00064717	0.00723758	0.00553923	35
BP	GO:0030203	glycosaminoglycan metabolic process	25/2016	117/18614	0.00065365	0.00730123	0.00558794	25
BP	GO:0015711	organic anion transport	69/2016	433/18614	0.00066014	0.00736474	0.00563654	69
BP	GO:0032642	regulation of chemokine production	22/2016	98/18614	0.00066887	0.00745309	0.00570416	22
BP	GO:0048592	eye morphogenesis	31/2016	157/18614	0.00067233	0.00748265	0.00572679	31
BP	GO:0002707	negative regulation of lymphocyte mediated immunity	15/2016	56/18614	0.00068556	0.0076115	0.00582541	15
BP	GO:0038066	p38MAPK cascade	15/2016	56/18614	0.00068556	0.0076115	0.00582541	15
BP	GO:0018958	phenol-containing compound metabolic process	24/2016	111/18614	0.00069472	0.00770383	0.00589607	24
BP	GO:0021537	telencephalon development	47/2016	270/18614	0.0007022	0.00776834	0.00594544	47
BP	GO:0045860	positive regulation of protein kinase activity	60/2016	366/18614	0.00070521	0.00776834	0.00594544	60
BP	GO:0001504	neurotransmitter uptake	13/2016	45/18614	0.00070654	0.00776834	0.00594544	13
BP	GO:1900744	regulation of p38MAPK cascade	13/2016	45/18614	0.00070654	0.00776834	0.00594544	13
BP	GO:1902622	regulation of neutrophil migration	13/2016	45/18614	0.00070654	0.00776834	0.00594544	13
BP	GO:0097191	extrinsic apoptotic signaling pathway	41/2016	227/18614	0.00070795	0.00776834	0.00594544	41
BP	GO:0043011	myeloid dendritic cell differentiation	8/2016	20/18614	0.00070897	0.00776834	0.00594544	8
BP	GO:0055093	response to hyperoxia	8/2016	20/18614	0.00070897	0.00776834	0.00594544	8
BP	GO:0072234	metanephric nephron tubule development	8/2016	20/18614	0.00070897	0.00776834	0.00594544	8
BP	GO:0097623	potassium ion export across plasma membrane	8/2016	20/18614	0.00070897	0.00776834	0.00594544	8
BP	GO:0001894	tissue homeostasis	46/2016	263/18614	0.00071651	0.00782531	0.00598904	46
BP	GO:0060249	anatomical structure homeostasis	46/2016	263/18614	0.00071651	0.00782531	0.00598904	46
BP	GO:0044000	movement in host	35/2016	185/18614	0.00071673	0.00782531	0.00598904	35
BP	GO:0045069	regulation of viral genome replication	20/2016	86/18614	0.0007186	0.00783647	0.00599758	20
BP	GO:0002720	positive regulation of cytokine production involved in immune response	19/2016	80/18614	0.00073449	0.00798138	0.00610849	19

BP	GO:0032890	regulation of organic acid transport	19/2016	80/18614	0.00073449	0.00798138	0.00610849	19
BP	GO:0097061	dendritic spine organization	19/2016	80/18614	0.00073449	0.00798138	0.00610849	19
BP	GO:0032732	positive regulation of interleukin-1 production	18/2016	74/18614	0.00074169	0.00805009	0.00616107	18
BP	GO:0048639	positive regulation of developmental growth	32/2016	165/18614	0.00076687	0.00830386	0.00635529	32
BP	GO:0050792	regulation of viral process	32/2016	165/18614	0.00076687	0.00830386	0.00635529	32
BP	GO:0042092	type 2 immune response	12/2016	40/18614	0.00077474	0.00837924	0.00641299	12
BP	GO:0001837	epithelial to mesenchymal transition	33/2016	172/18614	0.00077876	0.00841282	0.00643869	33
BP	GO:0043583	ear development	40/2016	221/18614	0.00078266	0.00844506	0.00646336	40
BP	GO:1900047	negative regulation of hemostasis	14/2016	51/18614	0.00078526	0.00846318	0.00647723	14
BP	GO:0010811	positive regulation of cell-substrate adhesion	26/2016	125/18614	0.00079077	0.00851262	0.00651507	26
BP	GO:0050886	endocrine process	21/2016	93/18614	0.00080897	0.0086453	0.00661662	21
BP	GO:0009074	aromatic amino acid family catabolic process	7/2016	16/18614	0.00081619	0.0086453	0.00661662	7
BP	GO:0034134	toll-like receptor 2 signaling pathway	7/2016	16/18614	0.00081619	0.0086453	0.00661662	7
BP	GO:0045579	positive regulation of B cell differentiation	7/2016	16/18614	0.00081619	0.0086453	0.00661662	7
BP	GO:0097091	synaptic vesicle clustering	7/2016	16/18614	0.00081619	0.0086453	0.00661662	7
BP	GO:0098969	neurotransmitter receptor transport to postsynaptic membrane	7/2016	16/18614	0.00081619	0.0086453	0.00661662	7
BP	GO:1901741	positive regulation of myoblast fusion	7/2016	16/18614	0.00081619	0.0086453	0.00661662	7
BP	GO:0002475	antigen processing and presentation via MHC class Ib	9/2016	25/18614	0.00081625	0.0086453	0.00661662	9
BP	GO:0009164	nucleoside catabolic process	9/2016	25/18614	0.00081625	0.0086453	0.00661662	9
BP	GO:0031579	membrane raft organization	9/2016	25/18614	0.00081625	0.0086453	0.00661662	9
BP	GO:0032693	negative regulation of interleukin-10 production	9/2016	25/18614	0.00081625	0.0086453	0.00661662	9
BP	GO:0099633	protein localization to postsynaptic specialization membrane	9/2016	25/18614	0.00081625	0.0086453	0.00661662	9
BP	GO:0099645	neurotransmitter receptor localization to postsynaptic specialization membrane	9/2016	25/18614	0.00081625	0.0086453	0.00661662	9
BP	GO:2000311	regulation of AMPA receptor activity	9/2016	25/18614	0.00081625	0.0086453	0.00661662	9

BP	GO:0002446	neutrophil mediated immunity	11/2016	35/18614	0.00082656	0.00866844	0.00663432	11
BP	GO:0018149	peptide cross-linking	11/2016	35/18614	0.00082656	0.00866844	0.00663432	11
BP	GO:0035633	maintenance of blood-brain barrier	11/2016	35/18614	0.00082656	0.00866844	0.00663432	11
BP	GO:0050974	detection of mechanical stimulus involved in sensory perception	11/2016	35/18614	0.00082656	0.00866844	0.00663432	11
BP	GO:0002645	positive regulation of tolerance induction	6/2016	12/18614	0.00082974	0.00866844	0.00663432	6
BP	GO:0002887	negative regulation of myeloid leukocyte mediated immunity	6/2016	12/18614	0.00082974	0.00866844	0.00663432	6
BP	GO:0016554	cytidine to uridine editing	6/2016	12/18614	0.00082974	0.00866844	0.00663432	6
BP	GO:0034135	regulation of toll-like receptor 2 signaling pathway	6/2016	12/18614	0.00082974	0.00866844	0.00663432	6
BP	GO:0043312	neutrophil degranulation	6/2016	12/18614	0.00082974	0.00866844	0.00663432	6
BP	GO:0070778	L-aspartate transmembrane transport	6/2016	12/18614	0.00082974	0.00866844	0.00663432	6
BP	GO:0086015	SA node cell action potential	6/2016	12/18614	0.00082974	0.00866844	0.00663432	6
BP	GO:0086018	SA node cell to atrial cardiac muscle cell signaling	6/2016	12/18614	0.00082974	0.00866844	0.00663432	6
BP	GO:0002448	mast cell mediated immunity	15/2016	57/18614	0.00083856	0.00875067	0.00669725	15
BP	GO:0048678	response to axon injury	20/2016	87/18614	0.00083971	0.00875273	0.00669884	20
BP	GO:0002027	regulation of heart rate	23/2016	106/18614	0.00084422	0.00878978	0.00672719	23
BP	GO:0002825	regulation of T-helper 1 type immune response	10/2016	30/18614	0.00084722	0.00881109	0.0067435	10
BP	GO:0036465	synaptic vesicle recycling	19/2016	81/18614	0.00086375	0.00895861	0.0068564	19
BP	GO:0034341	response to type II interferon	28/2016	139/18614	0.00086407	0.00895861	0.0068564	28
BP	GO:0031214	biomineral tissue development	33/2016	173/18614	0.00086432	0.00895861	0.0068564	33
BP	GO:0048659	smooth muscle cell proliferation	34/2016	180/18614	0.00087173	0.00902521	0.00690737	34
BP	GO:0071772	response to BMP	35/2016	187/18614	0.00087585	0.00904751	0.00692445	35
BP	GO:0071773	cellular response to BMP stimulus	35/2016	187/18614	0.00087585	0.00904751	0.00692445	35
BP	GO:0042698	ovulation cycle	18/2016	75/18614	0.00087869	0.00906667	0.00693911	18
BP	GO:0032835	glomerulus development	17/2016	69/18614	0.00088169	0.00906717	0.00693949	17
BP	GO:0045576	mast cell activation	17/2016	69/18614	0.00088169	0.00906717	0.00693949	17
BP	GO:1901016	regulation of potassium ion transmembrane transporter activity	17/2016	69/18614	0.00088169	0.00906717	0.00693949	17
BP	GO:0010712	regulation of collagen metabolic process	13/2016	46/18614	0.00088846	0.00911641	0.00697717	13

BP	GO:2000273	positive regulation of signaling receptor activity	13/2016	46/18614	0.00088846	0.00911641	0.00697717	13
BP	GO:0030101	natural killer cell activation	22/2016	100/18614	0.00089208	0.00914334	0.00699778	22
BP	GO:0019730	antimicrobial humoral response	26/2016	126/18614	0.00089566	0.00916981	0.00701804	26
BP	GO:0002062	chondrocyte differentiation	24/2016	113/18614	0.00090768	0.00928255	0.00710433	24
BP	GO:0007265	Ras protein signal transduction	55/2016	333/18614	0.00096208	0.00982799	0.00752178	55
BP	GO:0048771	tissue remodeling	34/2016	181/18614	0.00096398	0.00983649	0.00752828	34
BP	GO:0031103	axon regeneration	14/2016	52/18614	0.00096895	0.00986527	0.00755031	14
BP	GO:0086009	membrane repolarization	14/2016	52/18614	0.00096895	0.00986527	0.00755031	14
BP	GO:1904019	epithelial cell apoptotic process	28/2016	140/18614	0.00097066	0.00987177	0.00755529	28
BP	GO:0048246	macrophage chemotaxis	12/2016	41/18614	0.00098869	0.010044	0.0076871	12
BP	GO:0010718	positive regulation of epithelial to mesenchymal transition	15/2016	58/18614	0.00101949	0.01034557	0.0079179	15
BP	GO:0002837	regulation of immune response to tumor cell	8/2016	21/18614	0.00103726	0.01047961	0.00802049	8
BP	GO:0002888	positive regulation of myeloid leukocyte mediated immunity	8/2016	21/18614	0.00103726	0.01047961	0.00802049	8
BP	GO:0098962	regulation of postsynaptic neurotransmitter receptor activity	8/2016	21/18614	0.00103726	0.01047961	0.00802049	8
BP	GO:1901739	regulation of myoblast fusion	8/2016	21/18614	0.00103726	0.01047961	0.00802049	8
BP	GO:0019731	antibacterial humoral response	16/2016	64/18614	0.00104459	0.01054207	0.00806829	16
BP	GO:0002064	epithelial cell development	38/2016	210/18614	0.00104813	0.01056233	0.0080838	38
BP	GO:0048488	synaptic vesicle endocytosis	17/2016	70/18614	0.00104889	0.01056233	0.0080838	17
BP	GO:0014013	regulation of gliogenesis	23/2016	108/18614	0.00110528	0.01111808	0.00850914	23
BP	GO:0002920	regulation of humoral immune response	13/2016	47/18614	0.00110804	0.01112151	0.00851176	13
BP	GO:0046633	alpha-beta T cell proliferation	13/2016	47/18614	0.00110804	0.01112151	0.00851176	13
BP	GO:0002026	regulation of the force of heart contraction	9/2016	26/18614	0.00112947	0.01124899	0.00860933	9
BP	GO:0002407	dendritic cell chemotaxis	9/2016	26/18614	0.00112947	0.01124899	0.00860933	9
BP	GO:0010460	positive regulation of heart rate	9/2016	26/18614	0.00112947	0.01124899	0.00860933	9
BP	GO:0031639	plasminogen activation	9/2016	26/18614	0.00112947	0.01124899	0.00860933	9
BP	GO:0086013	membrane repolarization during cardiac muscle cell action potential	9/2016	26/18614	0.00112947	0.01124899	0.00860933	9

BP	GO:0095500	acetylcholine receptor signaling pathway	10/2016	31/18614	0.00113052	0.01124899	0.00860933	10
BP	GO:1900745	positive regulation of p38MAPK cascade	10/2016	31/18614	0.00113052	0.01124899	0.00860933	10
BP	GO:1902042	negative regulation of extrinsic apoptotic signaling pathway via death domain receptors	10/2016	31/18614	0.00113052	0.01124899	0.00860933	10
BP	GO:0048017	inositol lipid-mediated signaling	33/2016	176/18614	0.00117238	0.01165293	0.00891848	33
BP	GO:0021761	limbic system development	24/2016	115/18614	0.00117497	0.01165348	0.0089189	24
BP	GO:1903707	negative regulation of hemopoiesis	24/2016	115/18614	0.00117497	0.01165348	0.0089189	24
BP	GO:0007492	endoderm development	19/2016	83/18614	0.00118155	0.01170609	0.00895916	19
BP	GO:0042476	odontogenesis	27/2016	135/18614	0.00118416	0.01171938	0.00896934	27
BP	GO:0048146	positive regulation of fibroblast proliferation	14/2016	53/18614	0.00118745	0.01173928	0.00898457	14
BP	GO:0031348	negative regulation of defense response	48/2016	284/18614	0.00119431	0.01179447	0.00902681	48
BP	GO:0051651	maintenance of location in cell	41/2016	233/18614	0.00119943	0.01183237	0.00905581	41
BP	GO:0003407	neural retina development	18/2016	77/18614	0.00121843	0.0119828	0.00917095	18
BP	GO:0035296	regulation of tube diameter	28/2016	142/18614	0.00121859	0.0119828	0.00917095	28
BP	GO:0097746	blood vessel diameter maintenance	28/2016	142/18614	0.00121859	0.0119828	0.00917095	28
BP	GO:0048762	mesenchymal cell differentiation	44/2016	255/18614	0.00122465	0.01202954	0.00920671	44
BP	GO:0072080	nephron tubule development	21/2016	96/18614	0.00124696	0.01213885	0.00929037	21
BP	GO:0010324	membrane invagination	16/2016	65/18614	0.00124861	0.01213885	0.00929037	16
BP	GO:0046888	negative regulation of hormone secretion	16/2016	65/18614	0.00124861	0.01213885	0.00929037	16
BP	GO:0045622	regulation of T-helper cell differentiation	12/2016	42/18614	0.00124953	0.01213885	0.00929037	12
BP	GO:0085029	extracellular matrix assembly	12/2016	42/18614	0.00124953	0.01213885	0.00929037	12
BP	GO:1904037	positive regulation of epithelial cell apoptotic process	12/2016	42/18614	0.00124953	0.01213885	0.00929037	12
BP	GO:0003198	epithelial to mesenchymal transition involved in endocardial cushion formation	7/2016	17/18614	0.00125848	0.01213885	0.00929037	7
BP	GO:0016114	terpenoid biosynthetic process	7/2016	17/18614	0.00125848	0.01213885	0.00929037	7
BP	GO:0060080	inhibitory postsynaptic potential	7/2016	17/18614	0.00125848	0.01213885	0.00929037	7

BP	GO:0070206	protein trimerization	7/2016	17/18614	0.00125848	0.01213885	0.00929037	7
BP	GO:1903540	establishment of protein localization to postsynaptic membrane	7/2016	17/18614	0.00125848	0.01213885	0.00929037	7
BP	GO:0001661	conditioned taste aversion	3/2016	3/18614	0.00126875	0.01213885	0.00929037	3
BP	GO:0002436	immune complex clearance by monocytes and macrophages	3/2016	3/18614	0.00126875	0.01213885	0.00929037	3
BP	GO:0002538	arachidonic acid metabolite production involved in inflammatory response	3/2016	3/18614	0.00126875	0.01213885	0.00929037	3
BP	GO:0002540	leukotriene production involved in inflammatory response	3/2016	3/18614	0.00126875	0.01213885	0.00929037	3
BP	GO:0033693	neurofilament bundle assembly	3/2016	3/18614	0.00126875	0.01213885	0.00929037	3
BP	GO:0071725	response to triacyl bacterial lipopeptide	3/2016	3/18614	0.00126875	0.01213885	0.00929037	3
BP	GO:0071727	cellular response to triacyl bacterial lipopeptide	3/2016	3/18614	0.00126875	0.01213885	0.00929037	3
BP	GO:0090264	regulation of immune complex clearance by monocytes and macrophages	3/2016	3/18614	0.00126875	0.01213885	0.00929037	3
BP	GO:0099185	postsynaptic intermediate filament cytoskeleton organization	3/2016	3/18614	0.00126875	0.01213885	0.00929037	3
BP	GO:0150064	vertebrate eye-specific patterning	3/2016	3/18614	0.00126875	0.01213885	0.00929037	3
BP	GO:1902336	positive regulation of retinal ganglion cell axon guidance	3/2016	3/18614	0.00126875	0.01213885	0.00929037	3
BP	GO:1904612	response to 2,3,7,8-tetrachlorodibenzodioxine	3/2016	3/18614	0.00126875	0.01213885	0.00929037	3
BP	GO:1905604	negative regulation of blood-brain barrier permeability	3/2016	3/18614	0.00126875	0.01213885	0.00929037	3
BP	GO:2001189	negative regulation of T cell activation via T cell receptor contact with antigen bound to MHC molecule on antigen presenting cell	3/2016	3/18614	0.00126875	0.01213885	0.00929037	3
BP	GO:0002246	wound healing involved in inflammatory response	5/2016	9/18614	0.00128378	0.01220649	0.00934214	5
BP	GO:0006569	tryptophan catabolic process	5/2016	9/18614	0.00128378	0.01220649	0.00934214	5

BP	GO:0042436	indole-containing compound catabolic process	5/2016	9/18614	0.00128378	0.01220649	0.00934214	5
BP	GO:0070383	DNA cytosine deamination	5/2016	9/18614	0.00128378	0.01220649	0.00934214	5
BP	GO:0070672	response to interleukin-15	5/2016	9/18614	0.00128378	0.01220649	0.00934214	5
BP	GO:0090129	positive regulation of synapse maturation	5/2016	9/18614	0.00128378	0.01220649	0.00934214	5
BP	GO:0048525	negative regulation of viral process	20/2016	90/18614	0.00131377	0.0124659	0.00954068	20
BP	GO:0106027	neuron projection organization	20/2016	90/18614	0.00131377	0.0124659	0.00954068	20
BP	GO:0090287	regulation of cellular response to growth factor stimulus	56/2016	345/18614	0.00131759	0.01248934	0.00955862	56
BP	GO:1904892	regulation of receptor signaling pathway via STAT	24/2016	116/18614	0.00133233	0.01261604	0.00965559	24
BP	GO:0035150	regulation of tube size	28/2016	143/18614	0.00136192	0.01288297	0.00985988	28
BP	GO:0014075	response to amine	13/2016	48/18614	0.0013711	0.01294314	0.00990593	13
BP	GO:0042269	regulation of natural killer cell mediated cytotoxicity	13/2016	48/18614	0.0013711	0.01294314	0.00990593	13
BP	GO:0086019	cell-cell signaling involved in cardiac conduction	11/2016	37/18614	0.00138206	0.01303326	0.00997491	11
BP	GO:0050768	negative regulation of neurogenesis	29/2016	150/18614	0.00138802	0.01306276	0.00999748	29
BP	GO:2000377	regulation of reactive oxygen species metabolic process	29/2016	150/18614	0.00138802	0.01306276	0.00999748	29
BP	GO:0022612	gland morphogenesis	25/2016	123/18614	0.00139392	0.0130831	0.01001305	25
BP	GO:0002664	regulation of T cell tolerance induction	6/2016	13/18614	0.00140014	0.0130831	0.01001305	6
BP	GO:0021681	cerebellar granular layer development	6/2016	13/18614	0.00140014	0.0130831	0.01001305	6
BP	GO:0033604	negative regulation of catecholamine secretion	6/2016	13/18614	0.00140014	0.0130831	0.01001305	6
BP	GO:0060100	positive regulation of phagocytosis, engulfment	6/2016	13/18614	0.00140014	0.0130831	0.01001305	6
BP	GO:1900452	regulation of long-term synaptic depression	6/2016	13/18614	0.00140014	0.0130831	0.01001305	6
BP	GO:1905155	positive regulation of membrane invagination	6/2016	13/18614	0.00140014	0.0130831	0.01001305	6
BP	GO:0002437	inflammatory response to antigenic stimulus	18/2016	78/18614	0.00142644	0.01331537	0.01019081	18
BP	GO:0001657	ureteric bud development	21/2016	97/18614	0.00143217	0.01334175	0.01021101	21
BP	GO:0006942	regulation of striated muscle contraction	21/2016	97/18614	0.00143217	0.01334175	0.01021101	21

BP	GO:0007632	visual behavior	14/2016	54/18614	0.00144573	0.01344094	0.01028692	14
BP	GO:0043303	mast cell degranulation	14/2016	54/18614	0.00144573	0.01344094	0.01028692	14
BP	GO:0032535	regulation of cellular component size	57/2016	354/18614	0.00145291	0.01349406	0.01032758	57
BP	GO:0006024	glycosaminoglycan biosynthetic process	17/2016	72/18614	0.00146497	0.01357865	0.01039232	17
BP	GO:0140238	presynaptic endocytosis	17/2016	72/18614	0.00146497	0.01357865	0.01039232	17
BP	GO:0016485	protein processing	43/2016	250/18614	0.00147337	0.01358974	0.0104008	43
BP	GO:0002223	stimulatory C-type lectin receptor signaling pathway	8/2016	22/18614	0.0014765	0.01358974	0.0104008	8
BP	GO:0002834	regulation of response to tumor cell	8/2016	22/18614	0.0014765	0.01358974	0.0104008	8
BP	GO:0050961	detection of temperature stimulus involved in sensory perception	8/2016	22/18614	0.0014765	0.01358974	0.0104008	8
BP	GO:1900424	regulation of defense response to bacterium	8/2016	22/18614	0.0014765	0.01358974	0.0104008	8
BP	GO:1990840	response to lectin	8/2016	22/18614	0.0014765	0.01358974	0.0104008	8
BP	GO:1990858	cellular response to lectin	8/2016	22/18614	0.0014765	0.01358974	0.0104008	8
BP	GO:0050771	negative regulation of axonogenesis	16/2016	66/18614	0.00148524	0.01364196	0.01044077	16
BP	GO:0002431	Fc receptor mediated stimulatory signaling pathway	10/2016	32/18614	0.00148662	0.01364196	0.01044077	10
BP	GO:0002862	negative regulation of inflammatory response to antigenic stimulus	10/2016	32/18614	0.00148662	0.01364196	0.01044077	10
BP	GO:0014065	phosphatidylinositol 3-kinase signaling	28/2016	144/18614	0.0015196	0.01393066	0.01066172	28
BP	GO:0002710	negative regulation of T cell mediated immunity	9/2016	27/18614	0.00153305	0.01398429	0.01070277	9
BP	GO:0032753	positive regulation of interleukin-4 production	9/2016	27/18614	0.00153305	0.01398429	0.01070277	9
BP	GO:0051953	negative regulation of amine transport	9/2016	27/18614	0.00153305	0.01398429	0.01070277	9
BP	GO:0062149	detection of stimulus involved in sensory perception of pain	9/2016	27/18614	0.00153305	0.01398429	0.01070277	9
BP	GO:0072207	metanephric epithelium development	9/2016	27/18614	0.00153305	0.01398429	0.01070277	9
BP	GO:0003176	aortic valve development	12/2016	43/18614	0.00156479	0.01420347	0.01087051	12
BP	GO:0019098	reproductive behavior	12/2016	43/18614	0.00156479	0.01420347	0.01087051	12
BP	GO:0034142	toll-like receptor 4 signaling pathway	12/2016	43/18614	0.00156479	0.01420347	0.01087051	12
BP	GO:0048713	regulation of oligodendrocyte differentiation	12/2016	43/18614	0.00156479	0.01420347	0.01087051	12

BP	GO:0061001	regulation of dendritic spine morphogenesis	12/2016	43/18614	0.00156479	0.01420347	0.01087051	12
BP	GO:0007519	skeletal muscle tissue development	31/2016	165/18614	0.00157051	0.01424139	0.01089954	31
BP	GO:0001659	temperature homeostasis	33/2016	179/18614	0.0015721	0.01424175	0.01089981	33
BP	GO:0048015	phosphatidylinositol-mediated signaling	32/2016	172/18614	0.0015741	0.01424587	0.01090297	32
BP	GO:0051983	regulation of chromosome segregation	26/2016	131/18614	0.00162118	0.01465754	0.01121804	26
BP	GO:0072163	mesonephric epithelium development	21/2016	98/18614	0.00164034	0.01478714	0.01131722	21
BP	GO:0072164	mesonephric tubule development	21/2016	98/18614	0.00164034	0.01478714	0.01131722	21
BP	GO:0120162	positive regulation of cold-induced thermogenesis	21/2016	98/18614	0.00164034	0.01478714	0.01131722	21
BP	GO:0061028	establishment of endothelial barrier	13/2016	49/18614	0.00168399	0.01516583	0.01160705	13
BP	GO:0001794	type IIa hypersensitivity	4/2016	6/18614	0.00171841	0.01528141	0.01169551	4
BP	GO:0002445	type II hypersensitivity	4/2016	6/18614	0.00171841	0.01528141	0.01169551	4
BP	GO:0006682	galactosylceramide biosynthetic process	4/2016	6/18614	0.00171841	0.01528141	0.01169551	4
BP	GO:0019375	galactolipid biosynthetic process	4/2016	6/18614	0.00171841	0.01528141	0.01169551	4
BP	GO:0033591	response to L-ascorbic acid	4/2016	6/18614	0.00171841	0.01528141	0.01169551	4
BP	GO:0038110	interleukin-2-mediated signaling pathway	4/2016	6/18614	0.00171841	0.01528141	0.01169551	4
BP	GO:0051935	glutamate reuptake	4/2016	6/18614	0.00171841	0.01528141	0.01169551	4
BP	GO:0071352	cellular response to interleukin-2	4/2016	6/18614	0.00171841	0.01528141	0.01169551	4
BP	GO:0140009	L-aspartate import across plasma membrane	4/2016	6/18614	0.00171841	0.01528141	0.01169551	4
BP	GO:1900454	positive regulation of long-term synaptic depression	4/2016	6/18614	0.00171841	0.01528141	0.01169551	4
BP	GO:1901166	neural crest cell migration involved in autonomic nervous system development	4/2016	6/18614	0.00171841	0.01528141	0.01169551	4
BP	GO:1902564	negative regulation of neutrophil activation	4/2016	6/18614	0.00171841	0.01528141	0.01169551	4
BP	GO:2001188	regulation of T cell activation via T cell receptor contact with antigen bound to MHC molecule on antigen presenting cell	4/2016	6/18614	0.00171841	0.01528141	0.01169551	4

BP	GO:0046425	regulation of receptor signaling pathway via JAK-STAT	22/2016	105/18614	0.00174861	0.01551042	0.01187078	22
BP	GO:0010518	positive regulation of phospholipase activity	14/2016	55/18614	0.00174922	0.01551042	0.01187078	14
BP	GO:0051703	biological process involved in intraspecies interaction between organisms	14/2016	55/18614	0.00174922	0.01551042	0.01187078	14
BP	GO:0009595	detection of biotic stimulus	11/2016	38/18614	0.00175732	0.01554735	0.01189904	11
BP	GO:0140894	endolysosomal toll-like receptor signaling pathway	11/2016	38/18614	0.00175732	0.01554735	0.01189904	11
BP	GO:0060193	positive regulation of lipase activity	16/2016	67/18614	0.00175845	0.01554735	0.01189904	16
BP	GO:0002820	negative regulation of adaptive immune response	15/2016	61/18614	0.00177083	0.01562673	0.0119598	15
BP	GO:0098900	regulation of action potential	15/2016	61/18614	0.00177083	0.01562673	0.0119598	15
BP	GO:0071496	cellular response to external stimulus	53/2016	327/18614	0.00180353	0.01590008	0.012169	53
BP	GO:0014066	regulation of phosphatidylinositol 3-kinase signaling	23/2016	112/18614	0.0018408	0.01617934	0.01238273	23
BP	GO:0032611	interleukin-1 beta production	23/2016	112/18614	0.0018408	0.01617934	0.01238273	23
BP	GO:0032651	regulation of interleukin-1 beta production	23/2016	112/18614	0.0018408	0.01617934	0.01238273	23
BP	GO:0008625	extrinsic apoptotic signaling pathway via death domain receptors	19/2016	86/18614	0.00184224	0.01617934	0.01238273	19
BP	GO:0050708	regulation of protein secretion	46/2016	275/18614	0.00184802	0.01621462	0.01240974	46
BP	GO:0002693	positive regulation of cellular extravasation	7/2016	18/18614	0.00186818	0.01628287	0.01246197	7
BP	GO:0016264	gap junction assembly	7/2016	18/18614	0.00186818	0.01628287	0.01246197	7
BP	GO:0021554	optic nerve development	7/2016	18/18614	0.00186818	0.01628287	0.01246197	7
BP	GO:0045064	T-helper 2 cell differentiation	7/2016	18/18614	0.00186818	0.01628287	0.01246197	7
BP	GO:0050965	detection of temperature stimulus involved in sensory perception of pain	7/2016	18/18614	0.00186818	0.01628287	0.01246197	7
BP	GO:0051917	regulation of fibrinolysis	7/2016	18/18614	0.00186818	0.01628287	0.01246197	7
BP	GO:0060263	regulation of respiratory burst	7/2016	18/18614	0.00186818	0.01628287	0.01246197	7

BP	GO:0045446	endothelial cell differentiation	24/2016	119/18614	0.00191759	0.01669773	0.01277948	24
BP	GO:0036336	dendritic cell migration	10/2016	33/18614	0.00192859	0.01673018	0.01280431	10
BP	GO:0038094	Fc-gamma receptor signaling pathway	10/2016	33/18614	0.00192859	0.01673018	0.01280431	10
BP	GO:1904861	excitatory synapse assembly	10/2016	33/18614	0.00192859	0.01673018	0.01280431	10
BP	GO:1905145	cellular response to acetylcholine	10/2016	33/18614	0.00192859	0.01673018	0.01280431	10
BP	GO:0002792	negative regulation of peptide secretion	12/2016	44/18614	0.00194271	0.01680516	0.0128617	12
BP	GO:0046640	regulation of alpha-beta T cell proliferation	12/2016	44/18614	0.00194271	0.01680516	0.0128617	12
BP	GO:1905521	regulation of macrophage migration	12/2016	44/18614	0.00194271	0.01680516	0.0128617	12
BP	GO:0006023	aminoglycan biosynthetic process	17/2016	74/18614	0.00201236	0.0173913	0.0133103	17
BP	GO:0002675	positive regulation of acute inflammatory response	9/2016	28/18614	0.00204462	0.01763703	0.01349837	9
BP	GO:1903055	positive regulation of extracellular matrix organization	9/2016	28/18614	0.00204462	0.01763703	0.01349837	9
BP	GO:0019835	cytolysis	8/2016	23/18614	0.00205115	0.01764886	0.01350742	8
BP	GO:0072170	metanephric tubule development	8/2016	23/18614	0.00205115	0.01764886	0.01350742	8
BP	GO:0090596	sensory organ morphogenesis	45/2016	269/18614	0.00205325	0.01764886	0.01350742	45
BP	GO:1904894	positive regulation of receptor signaling pathway via STAT	13/2016	50/18614	0.00205366	0.01764886	0.01350742	13
BP	GO:0050852	T cell receptor signaling pathway	27/2016	140/18614	0.00206528	0.01773214	0.01357115	27
BP	GO:0009314	response to radiation	69/2016	452/18614	0.00207609	0.01780829	0.01362944	69
BP	GO:0006939	smooth muscle contraction	23/2016	113/18614	0.00207926	0.01781895	0.01363759	23
BP	GO:0045667	regulation of osteoblast differentiation	28/2016	147/18614	0.00209057	0.01789916	0.01369898	28
BP	GO:1905475	regulation of protein localization to membrane	32/2016	175/18614	0.0021005	0.01796207	0.01374713	32
BP	GO:0002279	mast cell activation involved in immune response	14/2016	56/18614	0.00210377	0.01796207	0.01374713	14
BP	GO:0002752	cell surface pattern recognition receptor signaling pathway	14/2016	56/18614	0.00210377	0.01796207	0.01374713	14
BP	GO:0043030	regulation of macrophage activation	15/2016	62/18614	0.0021062	0.01796613	0.01375024	15

BP	GO:0034446	substrate adhesion-dependent cell spreading	21/2016	100/18614	0.00213465	0.01819192	0.01392305	21
BP	GO:0045861	negative regulation of proteolysis	53/2016	330/18614	0.00220647	0.01878664	0.01437821	53
BP	GO:0044060	regulation of endocrine process	11/2016	39/18614	0.00221164	0.01881327	0.01439859	11
BP	GO:0007216	G protein-coupled glutamate receptor signaling pathway	6/2016	14/18614	0.00222687	0.01883838	0.01441781	6
BP	GO:0009159	deoxyribonucleoside monophosphate catabolic process	6/2016	14/18614	0.00222687	0.01883838	0.01441781	6
BP	GO:0046598	positive regulation of viral entry into host cell	6/2016	14/18614	0.00222687	0.01883838	0.01441781	6
BP	GO:0070593	dendrite self-avoidance	6/2016	14/18614	0.00222687	0.01883838	0.01441781	6
BP	GO:0075294	positive regulation by symbiont of entry into host	6/2016	14/18614	0.00222687	0.01883838	0.01441781	6
BP	GO:0099150	regulation of postsynaptic specialization assembly	6/2016	14/18614	0.00222687	0.01883838	0.01441781	6
BP	GO:1901215	negative regulation of neuron death	38/2016	219/18614	0.00229058	0.01935951	0.01481665	38
BP	GO:0030509	BMP signaling pathway	32/2016	176/18614	0.00230675	0.01947827	0.01490754	32
BP	GO:0001765	membrane raft assembly	5/2016	10/18614	0.00233898	0.01966028	0.01504684	5
BP	GO:0001820	serotonin secretion	5/2016	10/18614	0.00233898	0.01966028	0.01504684	5
BP	GO:0001915	negative regulation of T cell mediated cytotoxicity	5/2016	10/18614	0.00233898	0.01966028	0.01504684	5
BP	GO:0002676	regulation of chronic inflammatory response	5/2016	10/18614	0.00233898	0.01966028	0.01504684	5
BP	GO:0048241	epinephrine transport	5/2016	10/18614	0.00233898	0.01966028	0.01504684	5
BP	GO:0030890	positive regulation of B cell proliferation	12/2016	45/18614	0.00239222	0.02005619	0.01534985	12
BP	GO:0046636	negative regulation of alpha-beta T cell activation	12/2016	45/18614	0.00239222	0.02005619	0.01534985	12
BP	GO:0090092	regulation of transmembrane receptor protein serine/threonine kinase signaling pathway	49/2016	301/18614	0.00239262	0.02005619	0.01534985	49
BP	GO:0030282	bone mineralization	24/2016	121/18614	0.00241928	0.02024698	0.01549587	24
BP	GO:0048545	response to steroid hormone	54/2016	339/18614	0.00241978	0.02024698	0.01549587	54
BP	GO:0045577	regulation of B cell differentiation	10/2016	34/18614	0.00247068	0.02061662	0.01577877	10
BP	GO:0050869	negative regulation of B cell activation	10/2016	34/18614	0.00247068	0.02061662	0.01577877	10
BP	GO:1901658	glycosyl compound catabolic process	10/2016	34/18614	0.00247068	0.02061662	0.01577877	10
BP	GO:0051235	maintenance of location	55/2016	347/18614	0.00247972	0.02067329	0.01582214	55

BP	GO:0043113	receptor clustering	13/2016	51/18614	0.00248759	0.02068274	0.01582938	13
BP	GO:0050885	neuromuscular process controlling balance	13/2016	51/18614	0.00248759	0.02068274	0.01582938	13
BP	GO:1902895	positive regulation of miRNA transcription	13/2016	51/18614	0.00248759	0.02068274	0.01582938	13
BP	GO:0032731	positive regulation of interleukin-1 beta production	15/2016	63/18614	0.00249267	0.02070623	0.01584735	15
BP	GO:0007566	embryo implantation	14/2016	57/18614	0.0025157	0.02085992	0.01596498	14
BP	GO:0060997	dendritic spine morphogenesis	14/2016	57/18614	0.0025157	0.02085992	0.01596498	14
BP	GO:2001236	regulation of extrinsic apoptotic signaling pathway	29/2016	156/18614	0.00257074	0.02129712	0.01629959	29
BP	GO:0051701	biological process involved in interaction with host	36/2016	206/18614	0.00259722	0.02149715	0.01645268	36
BP	GO:0006029	proteoglycan metabolic process	20/2016	95/18614	0.00260723	0.02152195	0.01647166	20
BP	GO:0007091	metaphase/anaphase transition of mitotic cell cycle	20/2016	95/18614	0.00260723	0.02152195	0.01647166	20
BP	GO:0048704	embryonic skeletal system morphogenesis	20/2016	95/18614	0.00260723	0.02152195	0.01647166	20
BP	GO:0030431	sleep	9/2016	29/18614	0.00268349	0.02197761	0.01682039	9
BP	GO:0090025	regulation of monocyte chemotaxis	9/2016	29/18614	0.00268349	0.02197761	0.01682039	9
BP	GO:0099170	postsynaptic modulation of chemical synaptic transmission	9/2016	29/18614	0.00268349	0.02197761	0.01682039	9
BP	GO:0002281	macrophage activation involved in immune response	7/2016	19/18614	0.00268392	0.02197761	0.01682039	7
BP	GO:0016553	base conversion or substitution editing	7/2016	19/18614	0.00268392	0.02197761	0.01682039	7
BP	GO:0045091	regulation of single stranded viral RNA replication via double stranded DNA intermediate	7/2016	19/18614	0.00268392	0.02197761	0.01682039	7
BP	GO:0051580	regulation of neurotransmitter uptake	7/2016	19/18614	0.00268392	0.02197761	0.01682039	7
BP	GO:0071625	vocalization behavior	7/2016	19/18614	0.00268392	0.02197761	0.01682039	7
BP	GO:1901881	positive regulation of protein depolymerization	7/2016	19/18614	0.00268392	0.02197761	0.01682039	7
BP	GO:0043123	positive regulation of I-kappaB kinase/NF-kappaB signaling	34/2016	192/18614	0.00269434	0.02204333	0.01687069	34

BP	GO:0071675	regulation of mononuclear cell migration	24/2016	122/18614	0.00270928	0.02214585	0.01694916	24
BP	GO:0007422	peripheral nervous system development	17/2016	76/18614	0.00272149	0.02222587	0.0170104	17
BP	GO:0032970	regulation of actin filament-based process	60/2016	387/18614	0.00274016	0.02235857	0.01711196	60
BP	GO:0001823	mesonephros development	21/2016	102/18614	0.00274945	0.02239402	0.01713909	21
BP	GO:0032570	response to progesterone	11/2016	40/18614	0.00275667	0.02239402	0.01713909	11
BP	GO:0071392	cellular response to estradiol stimulus	11/2016	40/18614	0.00275667	0.02239402	0.01713909	11
BP	GO:0140448	signaling receptor ligand precursor processing	11/2016	40/18614	0.00275667	0.02239402	0.01713909	11
BP	GO:1903115	regulation of actin filament-based movement	11/2016	40/18614	0.00275667	0.02239402	0.01713909	11
BP	GO:0045216	cell-cell junction organization	37/2016	214/18614	0.00276172	0.02241521	0.01715531	37
BP	GO:0042430	indole-containing compound metabolic process	8/2016	24/18614	0.00278799	0.02258857	0.01728799	8
BP	GO:0098581	detection of external biotic stimulus	8/2016	24/18614	0.00278799	0.02258857	0.01728799	8
BP	GO:0032722	positive regulation of chemokine production	16/2016	70/18614	0.00284182	0.02300447	0.01760629	16
BP	GO:0051924	regulation of calcium ion transport	44/2016	266/18614	0.00284562	0.02301499	0.01761435	44
BP	GO:0007631	feeding behavior	22/2016	109/18614	0.00286571	0.02313683	0.01770759	22
BP	GO:1902106	negative regulation of leukocyte differentiation	22/2016	109/18614	0.00286571	0.02313683	0.01770759	22
BP	GO:0007094	mitotic spindle assembly checkpoint signaling	12/2016	46/18614	0.00292296	0.02349603	0.01798251	12
BP	GO:0046427	positive regulation of receptor signaling pathway via JAK-STAT	12/2016	46/18614	0.00292296	0.02349603	0.01798251	12
BP	GO:0071173	spindle assembly checkpoint signaling	12/2016	46/18614	0.00292296	0.02349603	0.01798251	12
BP	GO:0071174	mitotic spindle checkpoint signaling	12/2016	46/18614	0.00292296	0.02349603	0.01798251	12
BP	GO:2001222	regulation of neuron migration	12/2016	46/18614	0.00292296	0.02349603	0.01798251	12
BP	GO:0045807	positive regulation of endocytosis	20/2016	96/18614	0.0029652	0.02381475	0.01822643	20
BP	GO:0035023	regulation of Rho protein signal transduction	18/2016	83/18614	0.0029728	0.02385494	0.01825719	18
BP	GO:0002715	regulation of natural killer cell mediated immunity	13/2016	52/18614	0.00299381	0.02398171	0.01835422	13

BP	GO:0010543	regulation of platelet activation	13/2016	52/18614	0.00299381	0.02398171	0.01835422	13
BP	GO:0007389	pattern specification process	70/2016	467/18614	0.00307834	0.02461741	0.01884075	70
BP	GO:0007266	Rho protein signal transduction	25/2016	130/18614	0.00307851	0.02461741	0.01884075	25
BP	GO:0003333	amino acid transmembrane transport	21/2016	103/18614	0.0031088	0.02481644	0.01899307	21
BP	GO:0048144	fibroblast proliferation	21/2016	103/18614	0.0031088	0.02481644	0.01899307	21
BP	GO:0002719	negative regulation of cytokine production involved in immune response	10/2016	35/18614	0.00312833	0.02485936	0.01902592	10
BP	GO:0032633	interleukin-4 production	10/2016	35/18614	0.00312833	0.02485936	0.01902592	10
BP	GO:0032673	regulation of interleukin-4 production	10/2016	35/18614	0.00312833	0.02485936	0.01902592	10
BP	GO:0035025	positive regulation of Rho protein signal transduction	10/2016	35/18614	0.00312833	0.02485936	0.01902592	10
BP	GO:0098810	neurotransmitter reuptake	10/2016	35/18614	0.00312833	0.02485936	0.01902592	10
BP	GO:0044839	cell cycle G2/M phase transition	28/2016	151/18614	0.00313038	0.02485936	0.01902592	28
BP	GO:0022406	membrane docking	19/2016	90/18614	0.00318623	0.02528105	0.01934866	19
BP	GO:0045664	regulation of neuron differentiation	34/2016	194/18614	0.00320081	0.02537489	0.01942048	34
BP	GO:0051056	regulation of small GTPase mediated signal transduction	48/2016	298/18614	0.00326131	0.02583231	0.01977056	48
BP	GO:0002517	T cell tolerance induction	6/2016	15/18614	0.00337394	0.02660988	0.02036567	6
BP	GO:0002679	respiratory burst involved in defense response	6/2016	15/18614	0.00337394	0.02660988	0.02036567	6
BP	GO:0030201	heparan sulfate proteoglycan metabolic process	6/2016	15/18614	0.00337394	0.02660988	0.02036567	6
BP	GO:0035641	locomotory exploration behavior	6/2016	15/18614	0.00337394	0.02660988	0.02036567	6
BP	GO:0043518	negative regulation of DNA damage response, signal transduction by p53 class mediator	6/2016	15/18614	0.00337394	0.02660988	0.02036567	6
BP	GO:0030856	regulation of epithelial cell differentiation	30/2016	166/18614	0.0034015	0.02680427	0.02051444	30
BP	GO:0030048	actin filament-based movement	25/2016	131/18614	0.00342222	0.02692149	0.02060416	25
BP	GO:0070304	positive regulation of stress-activated protein kinase signaling cascade	25/2016	131/18614	0.00342222	0.02692149	0.02060416	25
BP	GO:0003002	regionalization	64/2016	422/18614	0.00343593	0.02700627	0.02066904	64

BP	GO:0021602	cranial nerve morphogenesis	9/2016	30/18614	0.00347047	0.02720798	0.02082342	9
BP	GO:0034656	nucleobase-containing small molecule catabolic process	9/2016	30/18614	0.00347047	0.02720798	0.02082342	9
BP	GO:0090330	regulation of platelet aggregation	9/2016	30/18614	0.00347047	0.02720798	0.02082342	9
BP	GO:0002701	negative regulation of production of molecular mediator of immune response	12/2016	47/18614	0.00354522	0.02772322	0.02121775	12
BP	GO:0002861	regulation of inflammatory response to antigenic stimulus	12/2016	47/18614	0.00354522	0.02772322	0.02121775	12
BP	GO:0031577	spindle checkpoint signaling	12/2016	47/18614	0.00354522	0.02772322	0.02121775	12
BP	GO:0035176	social behavior	13/2016	53/18614	0.00358087	0.02797822	0.02141292	13
BP	GO:0030071	regulation of mitotic metaphase/anaphase transition	19/2016	91/18614	0.00362708	0.0282325	0.02160753	19
BP	GO:0060996	dendritic spine development	19/2016	91/18614	0.00362708	0.0282325	0.02160753	19
BP	GO:0002248	connective tissue replacement involved in inflammatory response wound healing	4/2016	7/18614	0.00366556	0.0282325	0.02160753	4
BP	GO:0014062	regulation of serotonin secretion	4/2016	7/18614	0.00366556	0.0282325	0.02160753	4
BP	GO:0032289	central nervous system myelin formation	4/2016	7/18614	0.00366556	0.0282325	0.02160753	4
BP	GO:0035723	interleukin-15-mediated signaling pathway	4/2016	7/18614	0.00366556	0.0282325	0.02160753	4
BP	GO:0046137	negative regulation of vitamin metabolic process	4/2016	7/18614	0.00366556	0.0282325	0.02160753	4
BP	GO:0071286	cellular response to magnesium ion	4/2016	7/18614	0.00366556	0.0282325	0.02160753	4
BP	GO:0071350	cellular response to interleukin-15	4/2016	7/18614	0.00366556	0.0282325	0.02160753	4
BP	GO:0071420	cellular response to histamine	4/2016	7/18614	0.00366556	0.0282325	0.02160753	4
BP	GO:0097187	dentinogenesis	4/2016	7/18614	0.00366556	0.0282325	0.02160753	4
BP	GO:0098828	modulation of inhibitory postsynaptic potential	4/2016	7/18614	0.00366556	0.0282325	0.02160753	4
BP	GO:0098907	regulation of SA node cell action potential	4/2016	7/18614	0.00366556	0.0282325	0.02160753	4
BP	GO:0110088	hippocampal neuron apoptotic process	4/2016	7/18614	0.00366556	0.0282325	0.02160753	4

BP	GO:0110089	regulation of hippocampal neuron apoptotic process	4/2016	7/18614	0.00366556	0.0282325	0.02160753	4
BP	GO:1905684	regulation of plasma membrane repair	4/2016	7/18614	0.00366556	0.0282325	0.02160753	4
BP	GO:2001186	negative regulation of CD8-positive, alpha-beta T cell activation	4/2016	7/18614	0.00366556	0.0282325	0.02160753	4
BP	GO:0043010	camera-type eye development	53/2016	338/18614	0.00368653	0.02837025	0.02171295	53
BP	GO:0050830	defense response to Gram-positive bacterium	23/2016	118/18614	0.00370281	0.02842957	0.02175836	23
BP	GO:0014047	glutamate secretion	8/2016	25/18614	0.00371586	0.02842957	0.02175836	8
BP	GO:0017121	plasma membrane phospholipid scrambling	8/2016	25/18614	0.00371586	0.02842957	0.02175836	8
BP	GO:0043302	positive regulation of leukocyte degranulation	8/2016	25/18614	0.00371586	0.02842957	0.02175836	8
BP	GO:0046641	positive regulation of alpha-beta T cell proliferation	8/2016	25/18614	0.00371586	0.02842957	0.02175836	8
BP	GO:0048714	positive regulation of oligodendrocyte differentiation	8/2016	25/18614	0.00371586	0.02842957	0.02175836	8
BP	GO:0051957	positive regulation of amino acid transport	8/2016	25/18614	0.00371586	0.02842957	0.02175836	8
BP	GO:0039692	single stranded viral RNA replication via double stranded DNA intermediate	7/2016	20/18614	0.00374736	0.02859924	0.02188821	7
BP	GO:0042454	ribonucleoside catabolic process	7/2016	20/18614	0.00374736	0.02859924	0.02188821	7
BP	GO:0090128	regulation of synapse maturation	7/2016	20/18614	0.00374736	0.02859924	0.02188821	7
BP	GO:0031623	receptor internalization	24/2016	125/18614	0.00376149	0.02868334	0.02195257	24
BP	GO:0007369	gastrulation	34/2016	196/18614	0.00378641	0.02882561	0.02206146	34
BP	GO:0070302	regulation of stress-activated protein kinase signaling cascade	34/2016	196/18614	0.00378641	0.02882561	0.02206146	34
BP	GO:0044784	metaphase/anaphase transition of cell cycle	20/2016	98/18614	0.0038052	0.02894467	0.02215258	20
BP	GO:0030330	DNA damage response, signal transduction by p53 class mediator	16/2016	72/18614	0.00383348	0.02913569	0.02229878	16
BP	GO:0016049	cell growth	73/2016	495/18614	0.00385024	0.02923896	0.02237781	73
BP	GO:0006568	tryptophan metabolic process	5/2016	11/18614	0.00390756	0.02943138	0.02252508	5
BP	GO:0033632	regulation of cell-cell adhesion mediated by integrin	5/2016	11/18614	0.00390756	0.02943138	0.02252508	5

BP	GO:0035821	modulation of process of another organism	5/2016	11/18614	0.00390756	0.02943138	0.02252508	5
BP	GO:0038063	collagen-activated tyrosine kinase receptor signaling pathway	5/2016	11/18614	0.00390756	0.02943138	0.02252508	5
BP	GO:0045628	regulation of T-helper 2 cell differentiation	5/2016	11/18614	0.00390756	0.02943138	0.02252508	5
BP	GO:0051256	mitotic spindle midzone assembly	5/2016	11/18614	0.00390756	0.02943138	0.02252508	5
BP	GO:0099566	regulation of postsynaptic cytosolic calcium ion concentration	5/2016	11/18614	0.00390756	0.02943138	0.02252508	5
BP	GO:0110011	regulation of basement membrane organization	5/2016	11/18614	0.00390756	0.02943138	0.02252508	5
BP	GO:1904238	pericyte cell differentiation	5/2016	11/18614	0.00390756	0.02943138	0.02252508	5
BP	GO:2000833	positive regulation of steroid hormone secretion	5/2016	11/18614	0.00390756	0.02943138	0.02252508	5
BP	GO:0051930	regulation of sensory perception of pain	10/2016	36/18614	0.00391804	0.02943804	0.02253018	10
BP	GO:0051931	regulation of sensory perception	10/2016	36/18614	0.00391804	0.02943804	0.02253018	10
BP	GO:0086005	ventricular cardiac muscle cell action potential	10/2016	36/18614	0.00391804	0.02943804	0.02253018	10
BP	GO:0061387	regulation of extent of cell growth	21/2016	105/18614	0.00394618	0.02962535	0.02267354	21
BP	GO:2001259	positive regulation of cation channel activity	15/2016	66/18614	0.00401773	0.03013785	0.02306577	15
BP	GO:0048675	axon extension	23/2016	119/18614	0.00413052	0.03095869	0.023694	23
BP	GO:0048562	embryonic organ morphogenesis	47/2016	294/18614	0.00413888	0.03099617	0.02372268	47
BP	GO:0061333	renal tubule morphogenesis	17/2016	79/18614	0.00416525	0.03114483	0.02383646	17
BP	GO:0003179	heart valve morphogenesis	14/2016	60/18614	0.0041655	0.03114483	0.02383646	14
BP	GO:0009743	response to carbohydrate	39/2016	234/18614	0.00417416	0.03115891	0.02384723	39
BP	GO:2000116	regulation of cysteine-type endopeptidase activity	39/2016	234/18614	0.00417416	0.03115891	0.02384723	39
BP	GO:0007189	adenylate cyclase-activating G protein-coupled receptor signaling pathway	26/2016	140/18614	0.00421291	0.03142269	0.02404912	26
BP	GO:0097479	synaptic vesicle localization	13/2016	54/18614	0.00425787	0.03161742	0.02419815	13
BP	GO:0098703	calcium ion import across plasma membrane	13/2016	54/18614	0.00425787	0.03161742	0.02419815	13
BP	GO:0008542	visual learning	12/2016	48/18614	0.00426993	0.03161742	0.02419815	12

BP	GO:0033046	negative regulation of sister chromatid segregation	12/2016	48/18614	0.00426993	0.03161742	0.02419815	12
BP	GO:0033048	negative regulation of mitotic sister chromatid segregation	12/2016	48/18614	0.00426993	0.03161742	0.02419815	12
BP	GO:0035850	epithelial cell differentiation involved in kidney development	12/2016	48/18614	0.00426993	0.03161742	0.02419815	12
BP	GO:0044331	cell-cell adhesion mediated by cadherin	12/2016	48/18614	0.00426993	0.03161742	0.02419815	12
BP	GO:0045841	negative regulation of mitotic metaphase/anaphase transition	12/2016	48/18614	0.00426993	0.03161742	0.02419815	12
BP	GO:2000816	negative regulation of mitotic sister chromatid separation	12/2016	48/18614	0.00426993	0.03161742	0.02419815	12
BP	GO:2001237	negative regulation of extrinsic apoptotic signaling pathway	20/2016	99/18614	0.00429418	0.03177144	0.02431603	20
BP	GO:1905818	regulation of chromosome separation	16/2016	73/18614	0.00442661	0.0326806	0.02501184	16
BP	GO:0061437	renal system vasculature development	9/2016	31/18614	0.00442772	0.0326806	0.02501184	9
BP	GO:0061440	kidney vasculature development	9/2016	31/18614	0.00442772	0.0326806	0.02501184	9
BP	GO:0015807	L-amino acid transport	18/2016	86/18614	0.00443985	0.03274391	0.0250603	18
BP	GO:0050770	regulation of axonogenesis	28/2016	155/18614	0.00457932	0.03350592	0.0256435	28
BP	GO:0006906	vesicle fusion	23/2016	120/18614	0.00459869	0.03350592	0.0256435	23
BP	GO:0060562	epithelial tube morphogenesis	52/2016	334/18614	0.00462324	0.03350592	0.0256435	52
BP	GO:0048593	camera-type eye morphogenesis	24/2016	127/18614	0.00463825	0.03350592	0.0256435	24
BP	GO:0001550	ovarian cumulus expansion	3/2016	4/18614	0.00466331	0.03350592	0.0256435	3
BP	GO:0007057	spindle assembly involved in female meiosis I	3/2016	4/18614	0.00466331	0.03350592	0.0256435	3
BP	GO:0009753	response to jasmonic acid	3/2016	4/18614	0.00466331	0.03350592	0.0256435	3
BP	GO:0019747	regulation of isoprenoid metabolic process	3/2016	4/18614	0.00466331	0.03350592	0.0256435	3
BP	GO:0033366	protein localization to secretory granule	3/2016	4/18614	0.00466331	0.03350592	0.0256435	3
BP	GO:0038156	interleukin-3-mediated signaling pathway	3/2016	4/18614	0.00466331	0.03350592	0.0256435	3
BP	GO:0043315	positive regulation of neutrophil degranulation	3/2016	4/18614	0.00466331	0.03350592	0.0256435	3

BP	GO:0043316	cytotoxic T cell degranulation	3/2016	4/18614	0.00466331	0.03350592	0.0256435	3
BP	GO:0045163	clustering of voltage-gated potassium channels	3/2016	4/18614	0.00466331	0.03350592	0.0256435	3
BP	GO:0048642	negative regulation of skeletal muscle tissue development	3/2016	4/18614	0.00466331	0.03350592	0.0256435	3
BP	GO:0050975	sensory perception of touch	3/2016	4/18614	0.00466331	0.03350592	0.0256435	3
BP	GO:0051941	regulation of amino acid uptake involved in synaptic transmission	3/2016	4/18614	0.00466331	0.03350592	0.0256435	3
BP	GO:0051946	regulation of glutamate uptake involved in transmission of nerve impulse	3/2016	4/18614	0.00466331	0.03350592	0.0256435	3
BP	GO:0061590	calcium activated phosphatidylcholine scrambling	3/2016	4/18614	0.00466331	0.03350592	0.0256435	3
BP	GO:0071226	cellular response to molecule of fungal origin	3/2016	4/18614	0.00466331	0.03350592	0.0256435	3
BP	GO:0071395	cellular response to jasmonic acid stimulus	3/2016	4/18614	0.00466331	0.03350592	0.0256435	3
BP	GO:0072268	pattern specification involved in metanephros development	3/2016	4/18614	0.00466331	0.03350592	0.0256435	3
BP	GO:0097490	sympathetic neuron projection extension	3/2016	4/18614	0.00466331	0.03350592	0.0256435	3
BP	GO:0097491	sympathetic neuron projection guidance	3/2016	4/18614	0.00466331	0.03350592	0.0256435	3
BP	GO:0098967	exocytic insertion of neurotransmitter receptor to postsynaptic membrane	3/2016	4/18614	0.00466331	0.03350592	0.0256435	3
BP	GO:1902308	regulation of peptidyl-serine dephosphorylation	3/2016	4/18614	0.00466331	0.03350592	0.0256435	3
BP	GO:1902566	regulation of eosinophil activation	3/2016	4/18614	0.00466331	0.03350592	0.0256435	3
BP	GO:1902669	positive regulation of axon guidance	3/2016	4/18614	0.00466331	0.03350592	0.0256435	3
BP	GO:1903766	positive regulation of potassium ion export across plasma membrane	3/2016	4/18614	0.00466331	0.03350592	0.0256435	3
BP	GO:1904151	positive regulation of microglial cell mediated cytotoxicity	3/2016	4/18614	0.00466331	0.03350592	0.0256435	3
BP	GO:1905686	positive regulation of plasma membrane repair	3/2016	4/18614	0.00466331	0.03350592	0.0256435	3
BP	GO:1905702	regulation of inhibitory synapse assembly	3/2016	4/18614	0.00466331	0.03350592	0.0256435	3

BP	GO:1990708	conditioned place preference	3/2016	4/18614	0.00466331	0.03350592	0.0256435	3
BP	GO:2000097	regulation of smooth muscle cell-matrix adhesion	3/2016	4/18614	0.00466331	0.03350592	0.0256435	3
BP	GO:2000241	regulation of reproductive process	35/2016	206/18614	0.00470501	0.03377919	0.02585264	35
BP	GO:0070227	lymphocyte apoptotic process	17/2016	80/18614	0.00476746	0.03417419	0.02615495	17
BP	GO:0072028	nephron morphogenesis	17/2016	80/18614	0.00476746	0.03417419	0.02615495	17
BP	GO:0007617	mating behavior	10/2016	37/18614	0.00485729	0.03460653	0.02648584	10
BP	GO:0008207	C21-steroid hormone metabolic process	10/2016	37/18614	0.00485729	0.03460653	0.02648584	10
BP	GO:0016486	peptide hormone processing	10/2016	37/18614	0.00485729	0.03460653	0.02648584	10
BP	GO:0002433	immune response-regulating cell surface receptor signaling pathway involved in phagocytosis	8/2016	26/18614	0.00486537	0.03460653	0.02648584	8
BP	GO:0002726	positive regulation of T cell cytokine production	8/2016	26/18614	0.00486537	0.03460653	0.02648584	8
BP	GO:0006817	phosphate ion transport	8/2016	26/18614	0.00486537	0.03460653	0.02648584	8
BP	GO:0009435	NAD biosynthetic process	8/2016	26/18614	0.00486537	0.03460653	0.02648584	8
BP	GO:0038096	Fc-gamma receptor signaling pathway involved in phagocytosis	8/2016	26/18614	0.00486537	0.03460653	0.02648584	8
BP	GO:1903306	negative regulation of regulated secretory pathway	8/2016	26/18614	0.00486537	0.03460653	0.02648584	8
BP	GO:1905523	positive regulation of macrophage migration	8/2016	26/18614	0.00486537	0.03460653	0.02648584	8
BP	GO:0051480	regulation of cytosolic calcium ion concentration	14/2016	61/18614	0.00487881	0.03464859	0.02651803	14
BP	GO:0060042	retina morphogenesis in camera-type eye	14/2016	61/18614	0.00487881	0.03464859	0.02651803	14
BP	GO:0010715	regulation of extracellular matrix disassembly	6/2016	16/18614	0.00490859	0.03472609	0.02657735	6
BP	GO:0045869	negative regulation of single stranded viral RNA replication via double stranded DNA intermediate	6/2016	16/18614	0.00490859	0.03472609	0.02657735	6
BP	GO:0048532	anatomical structure arrangement	6/2016	16/18614	0.00490859	0.03472609	0.02657735	6
BP	GO:0072578	neurotransmitter-gated ion channel clustering	6/2016	16/18614	0.00490859	0.03472609	0.02657735	6

BP	GO:1902285	semaphorin-plexin signaling pathway involved in neuron projection guidance	6/2016	16/18614	0.00490859	0.03472609	0.02657735	6
BP	GO:0048705	skeletal system morphogenesis	38/2016	229/18614	0.00500033	0.03534795	0.02705328	38
BP	GO:0007605	sensory perception of sound	28/2016	156/18614	0.0050187	0.03545059	0.02713184	28
BP	GO:0009116	nucleoside metabolic process	13/2016	55/18614	0.00503435	0.03550666	0.02717475	13
BP	GO:0052372	modulation by symbiont of entry into host	13/2016	55/18614	0.00503435	0.03550666	0.02717475	13
BP	GO:0048145	regulation of fibroblast proliferation	18/2016	87/18614	0.00504442	0.03555039	0.02720822	18
BP	GO:0006904	vesicle docking involved in exocytosis	11/2016	43/18614	0.00506522	0.03564239	0.02727863	11
BP	GO:0090278	negative regulation of peptide hormone secretion	11/2016	43/18614	0.00506522	0.03564239	0.02727863	11
BP	GO:0006837	serotonin transport	7/2016	21/18614	0.00510241	0.03567503	0.02730361	7
BP	GO:0007213	G protein-coupled acetylcholine receptor signaling pathway	7/2016	21/18614	0.00510241	0.03567503	0.02730361	7
BP	GO:0030728	ovulation	7/2016	21/18614	0.00510241	0.03567503	0.02730361	7
BP	GO:0051590	positive regulation of neurotransmitter transport	7/2016	21/18614	0.00510241	0.03567503	0.02730361	7
BP	GO:0090026	positive regulation of monocyte chemotaxis	7/2016	21/18614	0.00510241	0.03567503	0.02730361	7
BP	GO:0097049	motor neuron apoptotic process	7/2016	21/18614	0.00510241	0.03567503	0.02730361	7
BP	GO:1902259	regulation of delayed rectifier potassium channel activity	7/2016	21/18614	0.00510241	0.03567503	0.02730361	7
BP	GO:0032892	positive regulation of organic acid transport	12/2016	49/18614	0.00510861	0.03567503	0.02730361	12
BP	GO:0043114	regulation of vascular permeability	12/2016	49/18614	0.00510861	0.03567503	0.02730361	12
BP	GO:0045933	positive regulation of muscle contraction	12/2016	49/18614	0.00510861	0.03567503	0.02730361	12
BP	GO:0008361	regulation of cell size	31/2016	178/18614	0.0051975	0.03626825	0.02775762	31
BP	GO:1902099	regulation of metaphase/anaphase transition of cell cycle	19/2016	94/18614	0.00526289	0.03669668	0.02808553	19
BP	GO:0032872	regulation of stress-activated MAPK cascade	33/2016	193/18614	0.00537727	0.03746584	0.02867419	33
BP	GO:0048663	neuron fate commitment	15/2016	68/18614	0.00540442	0.03759808	0.0287754	15
BP	GO:0051057	positive regulation of small GTPase mediated signal transduction	15/2016	68/18614	0.00540442	0.03759808	0.0287754	15

BP	GO:0010976	positive regulation of neuron projection development	28/2016	157/18614	0.00549281	0.03818418	0.02922397	28
BP	GO:0001975	response to amphetamine	9/2016	32/18614	0.00557858	0.03869272	0.02961318	9
BP	GO:0002313	mature B cell differentiation involved in immune response	9/2016	32/18614	0.00557858	0.03869272	0.02961318	9
BP	GO:0010644	cell communication by electrical coupling	9/2016	32/18614	0.00557858	0.03869272	0.02961318	9
BP	GO:0090174	organelle membrane fusion	23/2016	122/18614	0.00566797	0.0392831	0.03006502	23
BP	GO:0032874	positive regulation of stress-activated MAPK cascade	24/2016	129/18614	0.00567904	0.03930066	0.03007846	24
BP	GO:0051928	positive regulation of calcium ion transport	24/2016	129/18614	0.00567904	0.03930066	0.03007846	24
BP	GO:0002823	negative regulation of adaptive immune response based on somatic recombination of immune receptors built from immunoglobulin superfamily domains	13/2016	56/18614	0.00592034	0.04087835	0.03128593	13
BP	GO:0045839	negative regulation of mitotic nuclear division	13/2016	56/18614	0.00592034	0.04087835	0.03128593	13
BP	GO:0060986	endocrine hormone secretion	13/2016	56/18614	0.00592034	0.04087835	0.03128593	13
BP	GO:0040036	regulation of fibroblast growth factor receptor signaling pathway	10/2016	38/18614	0.00596442	0.04115179	0.0314952	10
BP	GO:0050728	negative regulation of inflammatory response	32/2016	187/18614	0.0059825	0.04121476	0.0315434	32
BP	GO:0051302	regulation of cell division	32/2016	187/18614	0.0059825	0.04121476	0.0315434	32
BP	GO:0045581	negative regulation of T cell differentiation	12/2016	50/18614	0.00607334	0.04132361	0.0316267	12
BP	GO:0051985	negative regulation of chromosome segregation	12/2016	50/18614	0.00607334	0.04132361	0.0316267	12
BP	GO:1902100	negative regulation of metaphase/anaphase transition of cell cycle	12/2016	50/18614	0.00607334	0.04132361	0.0316267	12
BP	GO:1905819	negative regulation of chromosome separation	12/2016	50/18614	0.00607334	0.04132361	0.0316267	12
BP	GO:0000022	mitotic spindle elongation	5/2016	12/18614	0.006106	0.04132361	0.0316267	5
BP	GO:0002424	T cell mediated immune response to tumor cell	5/2016	12/18614	0.006106	0.04132361	0.0316267	5
BP	GO:0006216	cytidine catabolic process	5/2016	12/18614	0.006106	0.04132361	0.0316267	5
BP	GO:0006971	hypotonic response	5/2016	12/18614	0.006106	0.04132361	0.0316267	5
BP	GO:0009972	cytidine deamination	5/2016	12/18614	0.006106	0.04132361	0.0316267	5

BP	GO:0010454	negative regulation of cell fate commitment	5/2016	12/18614	0.006106	0.04132361	0.0316267	5
BP	GO:0030656	regulation of vitamin metabolic process	5/2016	12/18614	0.006106	0.04132361	0.0316267	5
BP	GO:0031953	negative regulation of protein autophosphorylation	5/2016	12/18614	0.006106	0.04132361	0.0316267	5
BP	GO:0032252	secretory granule localization	5/2016	12/18614	0.006106	0.04132361	0.0316267	5
BP	GO:0034145	positive regulation of toll-like receptor 4 signaling pathway	5/2016	12/18614	0.006106	0.04132361	0.0316267	5
BP	GO:0034154	toll-like receptor 7 signaling pathway	5/2016	12/18614	0.006106	0.04132361	0.0316267	5
BP	GO:0046087	cytidine metabolic process	5/2016	12/18614	0.006106	0.04132361	0.0316267	5
BP	GO:0097084	vascular associated smooth muscle cell development	5/2016	12/18614	0.006106	0.04132361	0.0316267	5
BP	GO:0099550	trans-synaptic signaling, modulating synaptic transmission	5/2016	12/18614	0.006106	0.04132361	0.0316267	5
BP	GO:1901731	positive regulation of platelet aggregation	5/2016	12/18614	0.006106	0.04132361	0.0316267	5
BP	GO:1902667	regulation of axon guidance	5/2016	12/18614	0.006106	0.04132361	0.0316267	5
BP	GO:1905809	negative regulation of synapse organization	5/2016	12/18614	0.006106	0.04132361	0.0316267	5
BP	GO:2001198	regulation of dendritic cell differentiation	5/2016	12/18614	0.006106	0.04132361	0.0316267	5
BP	GO:0045214	sarcomere organization	11/2016	44/18614	0.00610605	0.04132361	0.0316267	11
BP	GO:1904646	cellular response to amyloid-beta	11/2016	44/18614	0.00610605	0.04132361	0.0316267	11
BP	GO:1901657	glycosyl compound metabolic process	17/2016	82/18614	0.00618564	0.04178531	0.03198006	17
BP	GO:0007218	neuropeptide signaling pathway	21/2016	109/18614	0.00618789	0.04178531	0.03198006	21
BP	GO:0060840	artery development	21/2016	109/18614	0.00618789	0.04178531	0.03198006	21
BP	GO:0003158	endothelium development	25/2016	137/18614	0.00623311	0.04202099	0.03216044	25
BP	GO:0001964	startle response	8/2016	27/18614	0.00626845	0.04202099	0.03216044	8
BP	GO:0010818	T cell chemotaxis	8/2016	27/18614	0.00626845	0.04202099	0.03216044	8
BP	GO:0032740	positive regulation of interleukin-17 production	8/2016	27/18614	0.00626845	0.04202099	0.03216044	8
BP	GO:0034143	regulation of toll-like receptor 4 signaling pathway	8/2016	27/18614	0.00626845	0.04202099	0.03216044	8
BP	GO:0035929	steroid hormone secretion	8/2016	27/18614	0.00626845	0.04202099	0.03216044	8
BP	GO:0036296	response to increased oxygen levels	8/2016	27/18614	0.00626845	0.04202099	0.03216044	8

BP	GO:0042133	neurotransmitter metabolic process	8/2016	27/18614	0.00626845	0.04202099	0.03216044	8
BP	GO:0042832	defense response to protozoan	8/2016	27/18614	0.00626845	0.04202099	0.03216044	8
BP	GO:0099560	synaptic membrane adhesion	8/2016	27/18614	0.00626845	0.04202099	0.03216044	8
BP	GO:0002761	regulation of myeloid leukocyte differentiation	23/2016	123/18614	0.00627512	0.0420351	0.03217124	23
BP	GO:0042692	muscle cell differentiation	61/2016	410/18614	0.00641087	0.04291318	0.03284327	61
BP	GO:0050679	positive regulation of epithelial cell proliferation	37/2016	225/18614	0.00643785	0.04306246	0.03295752	37
BP	GO:0051784	negative regulation of nuclear division	14/2016	63/18614	0.0066003	0.04404661	0.03371073	14
BP	GO:0140014	mitotic nuclear division	45/2016	286/18614	0.00661114	0.04404661	0.03371073	45
BP	GO:0007044	cell-substrate junction assembly	19/2016	96/18614	0.00665759	0.04404661	0.03371073	19
BP	GO:1903510	mucopolysaccharide metabolic process	19/2016	96/18614	0.00665759	0.04404661	0.03371073	19
BP	GO:0002576	platelet degranulation	4/2016	8/18614	0.00670462	0.04404661	0.03371073	4
BP	GO:0006681	galactosylceramide metabolic process	4/2016	8/18614	0.00670462	0.04404661	0.03371073	4
BP	GO:0009131	pyrimidine nucleoside monophosphate catabolic process	4/2016	8/18614	0.00670462	0.04404661	0.03371073	4
BP	GO:0009178	pyrimidine deoxyribonucleoside monophosphate catabolic process	4/2016	8/18614	0.00670462	0.04404661	0.03371073	4
BP	GO:0009448	gamma-aminobutyric acid metabolic process	4/2016	8/18614	0.00670462	0.04404661	0.03371073	4
BP	GO:0010807	regulation of synaptic vesicle priming	4/2016	8/18614	0.00670462	0.04404661	0.03371073	4
BP	GO:0021612	facial nerve structural organization	4/2016	8/18614	0.00670462	0.04404661	0.03371073	4
BP	GO:0032119	sequestering of zinc ion	4/2016	8/18614	0.00670462	0.04404661	0.03371073	4
BP	GO:0035744	T-helper 1 cell cytokine production	4/2016	8/18614	0.00670462	0.04404661	0.03371073	4
BP	GO:0046476	glycosylceramide biosynthetic process	4/2016	8/18614	0.00670462	0.04404661	0.03371073	4
BP	GO:0051964	negative regulation of synapse assembly	4/2016	8/18614	0.00670462	0.04404661	0.03371073	4
BP	GO:0060013	righting reflex	4/2016	8/18614	0.00670462	0.04404661	0.03371073	4
BP	GO:0060372	regulation of atrial cardiac muscle cell membrane repolarization	4/2016	8/18614	0.00670462	0.04404661	0.03371073	4
BP	GO:0070669	response to interleukin-2	4/2016	8/18614	0.00670462	0.04404661	0.03371073	4
BP	GO:0098700	neurotransmitter loading into synaptic vesicle	4/2016	8/18614	0.00670462	0.04404661	0.03371073	4

BP	GO:1900122	positive regulation of receptor binding	4/2016	8/18614	0.00670462	0.04404661	0.03371073	4
BP	GO:1903764	regulation of potassium ion export across plasma membrane	4/2016	8/18614	0.00670462	0.04404661	0.03371073	4
BP	GO:2000427	positive regulation of apoptotic cell clearance	4/2016	8/18614	0.00670462	0.04404661	0.03371073	4
BP	GO:2000554	regulation of T-helper 1 cell cytokine production	4/2016	8/18614	0.00670462	0.04404661	0.03371073	4
BP	GO:2000556	positive regulation of T-helper 1 cell cytokine production	4/2016	8/18614	0.00670462	0.04404661	0.03371073	4
BP	GO:2001225	regulation of chloride transport	4/2016	8/18614	0.00670462	0.04404661	0.03371073	4
BP	GO:0003228	atrial cardiac muscle tissue development	7/2016	22/18614	0.0067945	0.04447833	0.03404115	7
BP	GO:0030449	regulation of complement activation	7/2016	22/18614	0.0067945	0.04447833	0.03404115	7
BP	GO:0051383	kinetochore organization	7/2016	22/18614	0.0067945	0.04447833	0.03404115	7
BP	GO:2000310	regulation of NMDA receptor activity	7/2016	22/18614	0.0067945	0.04447833	0.03404115	7
BP	GO:2000831	regulation of steroid hormone secretion	7/2016	22/18614	0.0067945	0.04447833	0.03404115	7
BP	GO:0030178	negative regulation of Wnt signaling pathway	30/2016	174/18614	0.00682817	0.04466699	0.03418554	30
BP	GO:0009408	response to heat	21/2016	110/18614	0.00688727	0.04497417	0.03442063	21
BP	GO:0042445	hormone metabolic process	39/2016	241/18614	0.00689297	0.04497417	0.03442063	39
BP	GO:0010763	positive regulation of fibroblast migration	6/2016	17/18614	0.00689956	0.04497417	0.03442063	6
BP	GO:0030889	negative regulation of B cell proliferation	6/2016	17/18614	0.00689956	0.04497417	0.03442063	6
BP	GO:0051969	regulation of transmission of nerve impulse	6/2016	17/18614	0.00689956	0.04497417	0.03442063	6
BP	GO:0072132	mesenchyme morphogenesis	13/2016	57/18614	0.00692626	0.04511185	0.03452601	13
BP	GO:0071887	leukocyte apoptotic process	22/2016	117/18614	0.00693048	0.04511185	0.03452601	22
BP	GO:0002828	regulation of type 2 immune response	9/2016	33/18614	0.00694733	0.04515762	0.03456103	9
BP	GO:0072677	eosinophil migration	9/2016	33/18614	0.00694733	0.04515762	0.03456103	9
BP	GO:0001570	vasculogenesis	17/2016	83/18614	0.00701319	0.04555355	0.03486406	17
BP	GO:0045058	T cell selection	12/2016	51/18614	0.00717664	0.04654955	0.03562634	12
BP	GO:0045761	regulation of adenylate cyclase activity	12/2016	51/18614	0.00717664	0.04654955	0.03562634	12
BP	GO:0098926	postsynaptic signal transduction	10/2016	39/18614	0.00725848	0.04704719	0.0360072	10
BP	GO:0030516	regulation of axon extension	18/2016	90/18614	0.00727348	0.04707814	0.03603089	18

BP	GO:0050829	defense response to Gram-negative bacterium	18/2016	90/18614	0.00727348	0.04707814	0.03603089	18
BP	GO:0048483	autonomic nervous system development	11/2016	45/18614	0.00730758	0.04723245	0.03614899	11
BP	GO:0062208	positive regulation of pattern recognition receptor signaling pathway	11/2016	45/18614	0.00730758	0.04723245	0.03614899	11
BP	GO:0071559	response to transforming growth factor beta	44/2016	280/18614	0.00734278	0.04742661	0.03629759	44
BP	GO:0030099	myeloid cell differentiation	62/2016	421/18614	0.00751719	0.04851909	0.03713371	62
BP	GO:0033045	regulation of sister chromatid segregation	20/2016	104/18614	0.00758253	0.0489065	0.03743021	20
BP	GO:0072088	nephron epithelium morphogenesis	16/2016	77/18614	0.00759251	0.04893662	0.03745327	16
BP	GO:0030166	proteoglycan biosynthetic process	14/2016	64/18614	0.00762631	0.04894879	0.03746258	14
BP	GO:0032615	interleukin-12 production	14/2016	64/18614	0.00762631	0.04894879	0.03746258	14
BP	GO:0032655	regulation of interleukin-12 production	14/2016	64/18614	0.00762631	0.04894879	0.03746258	14
BP	GO:0048247	lymphocyte chemotaxis	14/2016	64/18614	0.00762631	0.04894879	0.03746258	14
BP	GO:0048278	vesicle docking	14/2016	64/18614	0.00762631	0.04894879	0.03746258	14
BP	GO:0050918	positive chemotaxis	14/2016	64/18614	0.00762631	0.04894879	0.03746258	14
BP	GO:0002832	negative regulation of response to biotic stimulus	23/2016	125/18614	0.00765039	0.04906914	0.03755469	23
BP	GO:0030510	regulation of BMP signaling pathway	22/2016	118/18614	0.00767016	0.04914653	0.03761391	22
BP	GO:0000302	response to reactive oxygen species	34/2016	205/18614	0.00767314	0.04914653	0.03761391	34
BP	GO:0043409	negative regulation of MAPK cascade	31/2016	183/18614	0.00780863	0.04997958	0.03825148	31
CC	GO:0097060	synaptic membrane	142/2125	390/19518	2.83E-41	2.60E-38	1.81E-38	142
CC	GO:0062023	collagen-containing extracellular matrix	141/2125	415/19518	4.41E-37	2.03E-34	1.41E-34	141
CC	GO:0045211	postsynaptic membrane	103/2125	277/19518	4.54E-31	1.39E-28	9.70E-29	103
CC	GO:0034702	ion channel complex	100/2125	299/19518	5.78E-26	1.33E-23	9.25E-24	100
CC	GO:0043025	neuronal cell body	137/2125	500/19518	2.27E-25	4.18E-23	2.91E-23	137
CC	GO:0098978	glutamatergic synapse	113/2125	410/19518	2.51E-21	3.84E-19	2.68E-19	113
CC	GO:0042734	presynaptic membrane	62/2125	164/19518	1.23E-19	1.62E-17	1.13E-17	62
CC	GO:0009897	external side of plasma membrane	110/2125	426/19518	1.86E-18	2.14E-16	1.49E-16	110
CC	GO:1902495	transmembrane transporter complex	102/2125	392/19518	1.88E-17	1.92E-15	1.34E-15	102
CC	GO:0099572	postsynaptic specialization	96/2125	364/19518	6.50E-17	5.97E-15	4.16E-15	96
CC	GO:0098984	neuron to neuron synapse	99/2125	382/19518	7.54E-17	6.30E-15	4.39E-15	99
CC	GO:1990351	transporter complex	104/2125	418/19518	2.59E-16	1.98E-14	1.38E-14	104

CC	GO:0043204	perikaryon	54/2125	156/19518	2.34E-15	1.65E-13	1.15E-13	54
CC	GO:0032279	asymmetric synapse	88/2125	353/19518	4.69E-14	3.08E-12	2.14E-12	88
CC	GO:0034703	cation channel complex	59/2125	192/19518	5.31E-14	3.26E-12	2.27E-12	59
CC	GO:0005581	collagen trimer	36/2125	85/19518	9.27E-14	5.32E-12	3.71E-12	36
CC	GO:0150034	distal axon	74/2125	277/19518	1.19E-13	6.43E-12	4.48E-12	74
CC	GO:0014069	postsynaptic density	83/2125	339/19518	7.05E-13	3.60E-11	2.51E-11	83
CC	GO:0005788	endoplasmic reticulum lumen	78/2125	312/19518	1.11E-12	5.38E-11	3.75E-11	78
CC	GO:0030667	secretory granule membrane	79/2125	319/19518	1.35E-12	6.19E-11	4.31E-11	79
CC	GO:0099634	postsynaptic specialization membrane	44/2125	129/19518	1.59E-12	6.98E-11	4.86E-11	44
CC	GO:0005604	basement membrane	34/2125	84/19518	2.15E-12	8.98E-11	6.26E-11	34
CC	GO:0034705	potassium channel complex	35/2125	89/19518	2.78E-12	1.11E-10	7.74E-11	35
CC	GO:0044309	neuron spine	53/2125	178/19518	4.00E-12	1.53E-10	1.07E-10	53
CC	GO:0043197	dendritic spine	52/2125	175/19518	6.94E-12	2.55E-10	1.78E-10	52
CC	GO:0098982	GABA-ergic synapse	33/2125	84/19518	1.20E-11	4.24E-10	2.96E-10	33
CC	GO:0045121	membrane raft	77/2125	323/19518	1.96E-11	6.58E-10	4.58E-10	77
CC	GO:0060076	excitatory synapse	28/2125	64/19518	2.00E-11	6.58E-10	4.58E-10	28
CC	GO:0098857	membrane microdomain	77/2125	324/19518	2.30E-11	7.29E-10	5.08E-10	77
CC	GO:0072562	blood microparticle	45/2125	144/19518	2.72E-11	8.34E-10	5.81E-10	45
CC	GO:0008076	voltage-gated potassium channel complex	31/2125	78/19518	3.52E-11	1.04E-09	7.26E-10	31
CC	GO:0044306	neuron projection terminus	41/2125	129/19518	1.14E-10	3.27E-09	2.28E-09	41
CC	GO:0030666	endocytic vesicle membrane	53/2125	196/19518	2.19E-10	6.03E-09	4.20E-09	53
CC	GO:0098636	protein complex involved in cell adhesion	25/2125	57/19518	2.23E-10	6.03E-09	4.20E-09	25
CC	GO:0042611	MHC protein complex	16/2125	25/19518	2.89E-10	7.58E-09	5.28E-09	16
CC	GO:0043679	axon terminus	37/2125	114/19518	4.68E-10	1.19E-08	8.31E-09	37
CC	GO:0030672	synaptic vesicle membrane	39/2125	125/19518	5.93E-10	1.43E-08	9.99E-09	39
CC	GO:0099501	exocytic vesicle membrane	39/2125	125/19518	5.93E-10	1.43E-08	9.99E-09	39
CC	GO:0031256	leading edge membrane	48/2125	180/19518	2.53E-09	5.96E-08	4.15E-08	48
CC	GO:0030658	transport vesicle membrane	57/2125	234/19518	3.40E-09	7.82E-08	5.45E-08	57
CC	GO:0030139	endocytic vesicle	75/2125	347/19518	4.11E-09	9.22E-08	6.42E-08	75
CC	GO:0042613	MHC class II protein complex	12/2125	17/19518	9.87E-09	2.11E-07	1.47E-07	12
CC	GO:0098637	protein complex involved in cell-matrix adhesion	12/2125	17/19518	9.87E-09	2.11E-07	1.47E-07	12
CC	GO:0031091	platelet alpha granule	30/2125	91/19518	1.34E-08	2.80E-07	1.95E-07	30
CC	GO:0048786	presynaptic active zone	28/2125	82/19518	1.70E-08	3.47E-07	2.41E-07	28
CC	GO:0045177	apical part of cell	88/2125	446/19518	2.03E-08	4.05E-07	2.82E-07	88
CC	GO:0032589	neuron projection membrane	23/2125	60/19518	2.66E-08	5.20E-07	3.62E-07	23

CC	GO:0016324	apical plasma membrane	77/2125	381/19518	5.46E-08	1.05E-06	7.28E-07	77
CC	GO:0031093	platelet alpha granule lumen	24/2125	67/19518	6.19E-08	1.16E-06	8.08E-07	24
CC	GO:0098839	postsynaptic density membrane	31/2125	102/19518	6.59E-08	1.21E-06	8.44E-07	31
CC	GO:0008328	ionotropic glutamate receptor complex	18/2125	41/19518	7.37E-08	1.33E-06	9.24E-07	18
CC	GO:0098878	neurotransmitter receptor complex	19/2125	46/19518	1.06E-07	1.87E-06	1.30E-06	19
CC	GO:0034707	chloride channel complex	21/2125	55/19518	1.16E-07	2.00E-06	1.40E-06	21
CC	GO:0034774	secretory granule lumen	66/2125	322/19518	2.80E-07	4.77E-06	3.32E-06	66
CC	GO:1902710	GABA receptor complex	12/2125	21/19518	3.08E-07	5.15E-06	3.58E-06	12
CC	GO:0060205	cytoplasmic vesicle lumen	66/2125	325/19518	3.99E-07	6.55E-06	4.56E-06	66
CC	GO:0032590	dendrite membrane	17/2125	41/19518	4.63E-07	7.47E-06	5.20E-06	17
CC	GO:0044304	main axon	22/2125	64/19518	4.98E-07	7.70E-06	5.36E-06	22
CC	GO:0032809	neuronal cell body membrane	14/2125	29/19518	5.00E-07	7.70E-06	5.36E-06	14
CC	GO:0031983	vesicle lumen	66/2125	327/19518	5.03E-07	7.70E-06	5.36E-06	66
CC	GO:0071735	IgG immunoglobulin complex	8/2125	10/19518	7.17E-07	1.08E-05	7.52E-06	8
CC	GO:0097386	glial cell projection	16/2125	38/19518	7.85E-07	1.16E-05	8.10E-06	16
CC	GO:0098688	parallel fiber to Purkinje cell synapse	11/2125	19/19518	8.17E-07	1.19E-05	8.30E-06	11
CC	GO:0042383	sarcolemma	35/2125	136/19518	8.77E-07	1.26E-05	8.77E-06	35
CC	GO:0031253	cell projection membrane	68/2125	349/19518	1.29E-06	1.80E-05	1.25E-05	68
CC	GO:0030133	transport vesicle	79/2125	425/19518	1.31E-06	1.80E-05	1.25E-05	79
CC	GO:0030055	cell-substrate junction	80/2125	432/19518	1.31E-06	1.80E-05	1.25E-05	80
CC	GO:0070820	tertiary granule	39/2125	164/19518	1.85E-06	2.50E-05	1.74E-05	39
CC	GO:0005925	focal adhesion	78/2125	422/19518	1.91E-06	2.54E-05	1.77E-05	78
CC	GO:0044298	cell body membrane	14/2125	32/19518	2.21E-06	2.90E-05	2.02E-05	14
CC	GO:1904090	peptidase inhibitor complex	8/2125	11/19518	2.38E-06	3.08E-05	2.14E-05	8
CC	GO:0008021	synaptic vesicle	46/2125	210/19518	2.69E-06	3.43E-05	2.39E-05	46
CC	GO:0031252	cell leading edge	77/2125	422/19518	3.65E-06	4.60E-05	3.20E-05	77
CC	GO:0098802	plasma membrane signaling receptor complex	62/2125	319/19518	4.05E-06	5.03E-05	3.51E-05	62
CC	GO:0098685	Schaffer collateral - CA1 synapse	26/2125	94/19518	5.25E-06	6.41E-05	4.46E-05	26
CC	GO:0005883	neurofilament	7/2125	9/19518	5.30E-06	6.41E-05	4.46E-05	7
CC	GO:0030669	clathrin-coated endocytic vesicle membrane	22/2125	73/19518	6.03E-06	7.19E-05	5.01E-05	22
CC	GO:0042581	specific granule	37/2125	160/19518	6.73E-06	7.93E-05	5.52E-05	37
CC	GO:0070382	exocytic vesicle	47/2125	225/19518	8.20E-06	9.54E-05	6.64E-05	47
CC	GO:1902711	GABA-A receptor complex	10/2125	19/19518	8.37E-06	9.61E-05	6.69E-05	10
CC	GO:0045334	clathrin-coated endocytic vesicle	25/2125	92/19518	1.11E-05	0.00012614	8.78E-05	25

CC	GO:0030426	growth cone	37/2125	167/19518	1.88E-05	0.00021056	0.00014664	37
CC	GO:0031594	neuromuscular junction	21/2125	73/19518	2.15E-05	0.00023536	0.00016391	21
CC	GO:0070821	tertiary granule membrane	21/2125	73/19518	2.15E-05	0.00023536	0.00016391	21
CC	GO:0098553	lumenal side of endoplasmic reticulum membrane	12/2125	29/19518	2.36E-05	0.00025503	0.00017761	12
CC	GO:0043083	synaptic cleft	10/2125	21/19518	2.60E-05	0.00027474	0.00019133	10
CC	GO:0098644	complex of collagen trimers	10/2125	21/19518	2.60E-05	0.00027474	0.00019133	10
CC	GO:0030427	site of polarized growth	37/2125	172/19518	3.72E-05	0.00038818	0.00027033	37
CC	GO:0048788	cytoskeleton of presynaptic active zone	6/2125	8/19518	3.81E-05	0.00038928	0.0002711	6
CC	GO:0097179	protease inhibitor complex	6/2125	8/19518	3.81E-05	0.00038928	0.0002711	6
CC	GO:0098981	cholinergic synapse	7/2125	11/19518	3.98E-05	0.00039788	0.00027709	7
CC	GO:0099569	presynaptic cytoskeleton	7/2125	11/19518	3.98E-05	0.00039788	0.00027709	7
CC	GO:0032281	AMPA glutamate receptor complex	11/2125	26/19518	4.04E-05	0.00039924	0.00027803	11
CC	GO:0098831	presynaptic active zone cytoplasmic component	9/2125	18/19518	4.10E-05	0.00040097	0.00027924	9
CC	GO:0030665	clathrin-coated vesicle membrane	31/2125	136/19518	4.88E-05	0.00047215	0.00032881	31
CC	GO:0008305	integrin complex	12/2125	31/19518	5.21E-05	0.00049873	0.00034732	12
CC	GO:0032432	actin filament bundle	21/2125	78/19518	6.35E-05	0.00060162	0.00041897	21
CC	GO:0031045	dense core granule	14/2125	41/19518	6.42E-05	0.00060182	0.00041911	14
CC	GO:0045178	basal part of cell	53/2125	285/19518	6.80E-05	0.00063168	0.00043991	53
CC	GO:0044305	calyx of Held	9/2125	19/19518	7.04E-05	0.00064052	0.00044607	9
CC	GO:0097449	astrocyte projection	9/2125	19/19518	7.04E-05	0.00064052	0.00044607	9
CC	GO:0044853	plasma membrane raft	27/2125	114/19518	7.30E-05	0.00065731	0.00045776	27
CC	GO:0005583	fibrillar collagen trimer	7/2125	12/19518	8.66E-05	0.00076502	0.00053277	7
CC	GO:0098643	banded collagen fibril	7/2125	12/19518	8.66E-05	0.00076502	0.00053277	7
CC	GO:0019898	extrinsic component of membrane	53/2125	288/19518	9.04E-05	0.00079086	0.00055076	53
CC	GO:0001725	stress fiber	19/2125	69/19518	0.00010055	0.00086364	0.00060144	19
CC	GO:0097517	contractile actin filament bundle	19/2125	69/19518	0.00010055	0.00086364	0.00060144	19
CC	GO:0001772	immunological synapse	14/2125	43/19518	0.00011575	0.00098493	0.00068592	14
CC	GO:0030136	clathrin-coated vesicle	42/2125	215/19518	0.00012171	0.00102614	0.00071461	42
CC	GO:0099160	postsynaptic intermediate filament cytoskeleton	4/2125	4/19518	0.00014015	0.00117091	0.00081543	4
CC	GO:0099738	cell cortex region	13/2125	39/19518	0.00015426	0.00127713	0.00088941	13
CC	GO:0000940	outer kinetochore	7/2125	13/19518	0.00016991	0.00139419	0.00097093	7
CC	GO:0005912	adherens junction	37/2125	185/19518	0.00018355	0.00149275	0.00103956	37
CC	GO:0071745	IgA immunoglobulin complex	6/2125	10/19518	0.00023543	0.00189788	0.0013217	6
CC	GO:0035579	specific granule membrane	22/2125	91/19518	0.00023983	0.00191654	0.0013347	22
CC	GO:0030018	Z disc	28/2125	129/19518	0.00026653	0.00211153	0.00147049	28

CC	GO:0098686	hippocampal mossy fiber to CA3 synapse	13/2125	41/19518	0.00027174	0.00213445	0.00148645	13
CC	GO:0098576	lumenal side of membrane	12/2125	36/19518	0.00027515	0.00214294	0.00149236	12
CC	GO:0031258	lamellipodium membrane	9/2125	22/19518	0.00027966	0.00215975	0.00150407	9
CC	GO:0009925	basal plasma membrane	48/2125	266/19518	0.00030367	0.0023256	0.00161957	48
CC	GO:0030673	axolemma	7/2125	14/19518	0.00030787	0.00233832	0.00162843	7
CC	GO:0043020	NADPH oxidase complex	8/2125	18/19518	0.00031165	0.00234761	0.0016349	8
CC	GO:0005871	kinesin complex	14/2125	47/19518	0.00033207	0.00248109	0.00172785	14
CC	GO:0042641	actomyosin	19/2125	76/19518	0.00039526	0.00292936	0.00204003	19
CC	GO:0016323	basolateral plasma membrane	43/2125	234/19518	0.00041017	0.00301554	0.00210005	43
CC	GO:0019814	immunoglobulin complex	24/2125	108/19518	0.00049392	0.00357413	0.00248906	24
CC	GO:0030175	filopodium	24/2125	108/19518	0.00049392	0.00357413	0.00248906	24
CC	GO:0019897	extrinsic component of plasma membrane	30/2125	147/19518	0.00050218	0.00360548	0.00251089	30
CC	GO:0031674	I band	29/2125	141/19518	0.00054058	0.00385114	0.00268197	29
CC	GO:0015629	actin cytoskeleton	77/2125	489/19518	0.00056326	0.00398184	0.00277299	77
CC	GO:0005954	calcium- and calmodulin-dependent protein kinase complex	4/2125	5/19518	0.00063983	0.0043399	0.00302234	4
CC	GO:0035976	transcription factor AP-1 complex	4/2125	5/19518	0.00063983	0.0043399	0.00302234	4
CC	GO:0044327	dendritic spine head	5/2125	8/19518	0.00064225	0.0043399	0.00302234	5
CC	GO:0061200	clathrin-sculpted gamma-aminobutyric acid transport vesicle	5/2125	8/19518	0.00064225	0.0043399	0.00302234	5
CC	GO:0061202	clathrin-sculpted gamma-aminobutyric acid transport vesicle membrane	5/2125	8/19518	0.00064225	0.0043399	0.00302234	5
CC	GO:0071753	IgM immunoglobulin complex	5/2125	8/19518	0.00064225	0.0043399	0.00302234	5
CC	GO:0030135	coated vesicle	53/2125	312/19518	0.00068971	0.00462662	0.00322202	53
CC	GO:0060198	clathrin-sculpted vesicle	6/2125	12/19518	0.00085372	0.00568525	0.00395926	6
CC	GO:0043292	contractile fiber	43/2125	243/19518	0.00091797	0.00606915	0.00422661	43
CC	GO:0030662	coated vesicle membrane	37/2125	202/19518	0.00106184	0.00697025	0.00485415	37
CC	GO:0045335	phagocytic vesicle	28/2125	141/19518	0.00118157	0.00770116	0.00536316	28
CC	GO:0005775	vacuolar lumen	33/2125	176/19518	0.00128344	0.00805791	0.0056116	33
CC	GO:0005602	complement component C1 complex	3/2125	3/19518	0.00128891	0.00805791	0.0056116	3
CC	GO:0030485	smooth muscle contractile fiber	3/2125	3/19518	0.00128891	0.00805791	0.0056116	3
CC	GO:0034666	integrin alpha2-beta1 complex	3/2125	3/19518	0.00128891	0.00805791	0.0056116	3
CC	GO:0034668	integrin alpha4-beta1 complex	3/2125	3/19518	0.00128891	0.00805791	0.0056116	3
CC	GO:0062167	complement component C1q complex	3/2125	3/19518	0.00128891	0.00805791	0.0056116	3

CC	GO:0017146	NMDA selective glutamate receptor complex	5/2125	9/19518	0.00131535	0.0081676	0.00568799	5
CC	GO:0001527	microfibril	6/2125	13/19518	0.00143983	0.00888058	0.00618452	6
CC	GO:0032133	chromosome passenger complex	4/2125	6/19518	0.00175324	0.01060017	0.00738206	4
CC	GO:0042571	immunoglobulin complex, circulating	4/2125	6/19518	0.00175324	0.01060017	0.00738206	4
CC	GO:0097180	serine protease inhibitor complex	4/2125	6/19518	0.00175324	0.01060017	0.00738206	4
CC	GO:0043198	dendritic shaft	11/2125	38/19518	0.00183463	0.01101976	0.00767426	11
CC	GO:0005796	Golgi lumen	22/2125	106/19518	0.00212436	0.01267717	0.00882849	22
CC	GO:0014704	intercalated disc	13/2125	50/19518	0.00215425	0.01277262	0.00889497	13
CC	GO:0098858	actin-based cell projection	39/2125	225/19518	0.00227177	0.01338304	0.00932007	39
CC	GO:0099571	postsynaptic cytoskeleton	5/2125	10/19518	0.00239527	0.0140207	0.00976414	5
CC	GO:0048787	presynaptic active zone membrane	10/2125	34/19518	0.00257028	0.01494994	0.01041128	10
CC	GO:0043195	terminal bouton	12/2125	47/19518	0.00370379	0.02133676	0.01485912	12
CC	GO:0030863	cortical cytoskeleton	21/2125	104/19518	0.00373455	0.02133676	0.01485912	21
CC	GO:0043203	axon hillock	4/2125	7/19518	0.003738	0.02133676	0.01485912	4
CC	GO:0030670	phagocytic vesicle membrane	17/2125	78/19518	0.00383162	0.02173616	0.01513726	17
CC	GO:0060077	inhibitory synapse	7/2125	20/19518	0.00386137	0.02177054	0.0151612	7
CC	GO:0005614	interstitial matrix	5/2125	11/19518	0.00399953	0.02241202	0.01560794	5
CC	GO:0044291	cell-cell contact zone	16/2125	72/19518	0.00404041	0.02250386	0.01567189	16
CC	GO:0030016	myofibril	39/2125	233/19518	0.00424372	0.02349387	0.01636135	39
CC	GO:0034704	calcium channel complex	17/2125	79/19518	0.00439777	0.0242009	0.01685373	17
CC	GO:0005938	cell cortex	48/2125	302/19518	0.00470593	0.02515405	0.01751751	48
CC	GO:0033269	internode region of axon	3/2125	4/19518	0.0047352	0.02515405	0.01751751	3
CC	GO:0060199	clathrin-sculpted glutamate transport vesicle	3/2125	4/19518	0.0047352	0.02515405	0.01751751	3
CC	GO:0060203	clathrin-sculpted glutamate transport vesicle membrane	3/2125	4/19518	0.0047352	0.02515405	0.01751751	3
CC	GO:0071748	monomeric IgA immunoglobulin complex	3/2125	4/19518	0.0047352	0.02515405	0.01751751	3
CC	GO:0098855	HCN channel complex	3/2125	4/19518	0.0047352	0.02515405	0.01751751	3
CC	GO:0005892	acetylcholine-gated channel complex	6/2125	16/19518	0.00503981	0.02661829	0.01853722	6
CC	GO:0101003	ficolin-1-rich granule membrane	14/2125	61/19518	0.00511535	0.0268629	0.01870757	14
CC	GO:0033162	melanosome membrane	7/2125	21/19518	0.00525486	0.02703641	0.01882841	7
CC	GO:0045009	chitosome	7/2125	21/19518	0.00525486	0.02703641	0.01882841	7
CC	GO:0090741	pigment granule membrane	7/2125	21/19518	0.00525486	0.02703641	0.01882841	7
CC	GO:0005891	voltage-gated calcium channel complex	13/2125	55/19518	0.00526607	0.02703641	0.01882841	13

CC	GO:0035580	specific granule lumen	14/2125	62/19518	0.00595989	0.03042854	0.02119071	14
CC	GO:0036454	growth factor complex	4/2125	8/19518	0.00683374	0.03450666	0.02403075	4
CC	GO:0098651	basement membrane collagen trimer	4/2125	8/19518	0.00683374	0.03450666	0.02403075	4
CC	GO:0012507	ER to Golgi transport vesicle membrane	14/2125	63/19518	0.00691254	0.03471381	0.02417501	14
CC	GO:0098992	neuronal dense core vesicle	8/2125	28/19518	0.00821175	0.04101414	0.02856262	8
CC	GO:0043202	lysosomal lumen	19/2125	98/19518	0.00880989	0.04376374	0.03047747	19
CC	GO:0042470	melanosome	21/2125	112/19518	0.00898932	0.04417744	0.03076557	21
CC	GO:0048770	pigment granule	21/2125	112/19518	0.00898932	0.04417744	0.03076557	21
CC	GO:0042583	chromaffin granule	5/2125	13/19518	0.00925074	0.04457844	0.03104483	5
CC	GO:0044194	cytolytic granule	5/2125	13/19518	0.00925074	0.04457844	0.03104483	5
CC	GO:0005766	primary lysosome	27/2125	155/19518	0.00926494	0.04457844	0.03104483	27
CC	GO:0042582	azurophil granule	27/2125	155/19518	0.00926494	0.04457844	0.03104483	27
CC	GO:0043034	costamere	6/2125	18/19518	0.00965695	0.04598309	0.03202304	6
CC	GO:1905286	serine-type peptidase complex	6/2125	18/19518	0.00965695	0.04598309	0.03202304	6
CC	GO:0101002	ficolin-1-rich granule	31/2125	185/19518	0.00981423	0.0464911	0.03237683	31
CC	GO:0030134	COPII-coated ER to Golgi transport vesicle	18/2125	93/19518	0.01078581	0.04693454	0.03268565	18
CC	GO:0005592	collagen type XI trimer	3/2125	5/19518	0.01087826	0.04693454	0.03268565	3
CC	GO:0016942	insulin-like growth factor binding protein complex	3/2125	5/19518	0.01087826	0.04693454	0.03268565	3
CC	GO:0030895	apolipoprotein B mRNA editing enzyme complex	3/2125	5/19518	0.01087826	0.04693454	0.03268565	3
CC	GO:0036021	endolysosome lumen	3/2125	5/19518	0.01087826	0.04693454	0.03268565	3
CC	GO:0071746	IgA immunoglobulin complex, circulating	3/2125	5/19518	0.01087826	0.04693454	0.03268565	3
CC	GO:0071749	polymeric IgA immunoglobulin complex	3/2125	5/19518	0.01087826	0.04693454	0.03268565	3
CC	GO:0071751	secretory IgA immunoglobulin complex	3/2125	5/19518	0.01087826	0.04693454	0.03268565	3
CC	GO:0071953	elastic fiber	3/2125	5/19518	0.01087826	0.04693454	0.03268565	3
CC	GO:0097418	neurofibrillary tangle	3/2125	5/19518	0.01087826	0.04693454	0.03268565	3
CC	GO:0098843	postsynaptic endocytic zone	3/2125	5/19518	0.01087826	0.04693454	0.03268565	3
CC	GO:0098966	perisynaptic extracellular matrix	3/2125	5/19518	0.01087826	0.04693454	0.03268565	3
CC	GO:0099012	neuronal dense core vesicle membrane	3/2125	5/19518	0.01087826	0.04693454	0.03268565	3
CC	GO:0042612	MHC class I protein complex	4/2125	9/19518	0.01124846	0.04693454	0.03268565	4
CC	GO:0043256	laminin complex	4/2125	9/19518	0.01124846	0.04693454	0.03268565	4
CC	GO:0045179	apical cortex	4/2125	9/19518	0.01124846	0.04693454	0.03268565	4
CC	GO:0031234	extrinsic component of cytoplasmic side of plasma membrane	16/2125	80/19518	0.01153203	0.04693454	0.03268565	16

CC	GO:0002095	caveolar macromolecular signaling complex	2/2125	2/19518	0.01184855	0.04693454	0.03268565	2
CC	GO:0005584	collagen type I trimer	2/2125	2/19518	0.01184855	0.04693454	0.03268565	2
CC	GO:0005589	collagen type VI trimer	2/2125	2/19518	0.01184855	0.04693454	0.03268565	2
CC	GO:0005607	laminin-2 complex	2/2125	2/19518	0.01184855	0.04693454	0.03268565	2
CC	GO:0005893	interleukin-2 receptor complex	2/2125	2/19518	0.01184855	0.04693454	0.03268565	2
CC	GO:0034665	integrin alpha1-beta1 complex	2/2125	2/19518	0.01184855	0.04693454	0.03268565	2
CC	GO:0034667	integrin alpha3-beta1 complex	2/2125	2/19518	0.01184855	0.04693454	0.03268565	2
CC	GO:0034674	integrin alpha5-beta1 complex	2/2125	2/19518	0.01184855	0.04693454	0.03268565	2
CC	GO:0034681	integrin alpha11-beta1 complex	2/2125	2/19518	0.01184855	0.04693454	0.03268565	2
CC	GO:0034688	integrin alphaM-beta2 complex	2/2125	2/19518	0.01184855	0.04693454	0.03268565	2
CC	GO:0035354	Toll-like receptor 1-Toll-like receptor 2 protein complex	2/2125	2/19518	0.01184855	0.04693454	0.03268565	2
CC	GO:0035692	macrophage migration inhibitory factor receptor complex	2/2125	2/19518	0.01184855	0.04693454	0.03268565	2
CC	GO:0036284	tubulobulbar complex	2/2125	2/19518	0.01184855	0.04693454	0.03268565	2
CC	GO:0070554	synaptobrevin 2-SNAP-25-syntaxin-3-complexin complex	2/2125	2/19518	0.01184855	0.04693454	0.03268565	2
CC	GO:0097058	CRLF-CLCF1 complex	2/2125	2/19518	0.01184855	0.04693454	0.03268565	2
CC	GO:0097059	CNTFR-CLCF1 complex	2/2125	2/19518	0.01184855	0.04693454	0.03268565	2
CC	GO:0098647	collagen beaded filament	2/2125	2/19518	0.01184855	0.04693454	0.03268565	2
CC	GO:1902712	G protein-coupled GABA receptor complex	2/2125	2/19518	0.01184855	0.04693454	0.03268565	2
CC	GO:1990665	AnxA2-p11 complex	2/2125	2/19518	0.01184855	0.04693454	0.03268565	2
CC	GO:1990794	basolateral part of cell	2/2125	2/19518	0.01184855	0.04693454	0.03268565	2
CC	GO:1990971	EMILIN complex	2/2125	2/19518	0.01184855	0.04693454	0.03268565	2
CC	GO:0005771	multivesicular body	14/2125	67/19518	0.01199482	0.04731006	0.03294716	14
CC	GO:0002102	podosome	8/2125	30/19518	0.01270955	0.04991485	0.03476116	8
MF	GO:0015267	channel activity	141/2054	478/18369	1.63E-28	1.98E-25	1.66E-25	141
MF	GO:0022803	passive transmembrane transporter activity	141/2054	479/18369	2.07E-28	1.98E-25	1.66E-25	141
MF	GO:0005216	monoatomic ion channel activity	131/2054	432/18369	7.95E-28	5.09E-25	4.25E-25	131
MF	GO:0022836	gated channel activity	105/2054	308/18369	4.60E-27	1.77E-24	1.47E-24	105
MF	GO:0022839	monoatomic ion gated channel activity	105/2054	308/18369	4.60E-27	1.77E-24	1.47E-24	105
MF	GO:0005201	extracellular matrix structural constituent	65/2054	167/18369	1.04E-20	3.32E-18	2.77E-18	65

MF	GO:0005244	voltage-gated monoatomic ion channel activity	69/2054	189/18369	4.44E-20	1.07E-17	8.89E-18	69
MF	GO:0022832	voltage-gated channel activity	69/2054	189/18369	4.44E-20	1.07E-17	8.89E-18	69
MF	GO:0005261	monoatomic cation channel activity	96/2054	324/18369	6.75E-20	1.44E-17	1.20E-17	96
MF	GO:0030594	neurotransmitter receptor activity	46/2054	98/18369	6.32E-19	1.21E-16	1.01E-16	46
MF	GO:0022843	voltage-gated monoatomic cation channel activity	56/2054	140/18369	1.10E-18	1.93E-16	1.61E-16	56
MF	GO:0046873	metal ion transmembrane transporter activity	112/2054	435/18369	7.93E-18	1.27E-15	1.06E-15	112
MF	GO:0005249	voltage-gated potassium channel activity	42/2054	88/18369	9.77E-18	1.44E-15	1.20E-15	42
MF	GO:0015079	potassium ion transmembrane transporter activity	58/2054	157/18369	2.25E-17	3.08E-15	2.57E-15	58
MF	GO:0005267	potassium channel activity	48/2054	121/18369	4.81E-16	6.16E-14	5.14E-14	48
MF	GO:0015276	ligand-gated monoatomic ion channel activity	51/2054	140/18369	3.70E-15	4.18E-13	3.49E-13	51
MF	GO:0022834	ligand-gated channel activity	51/2054	140/18369	3.70E-15	4.18E-13	3.49E-13	51
MF	GO:0005518	collagen binding	29/2054	66/18369	1.48E-11	1.58E-09	1.32E-09	29
MF	GO:0140375	immune receptor activity	45/2054	141/18369	2.97E-11	3.00E-09	2.51E-09	45
MF	GO:0003823	antigen binding	40/2054	118/18369	4.59E-11	4.40E-09	3.68E-09	40
MF	GO:0098960	postsynaptic neurotransmitter receptor activity	24/2054	52/18369	2.40E-10	2.06E-08	1.72E-08	24
MF	GO:0022824	transmitter-gated monoatomic ion channel activity	25/2054	56/18369	2.47E-10	2.06E-08	1.72E-08	25
MF	GO:0022835	transmitter-gated channel activity	25/2054	56/18369	2.47E-10	2.06E-08	1.72E-08	25
MF	GO:0019838	growth factor binding	41/2054	132/18369	5.64E-10	4.51E-08	3.76E-08	41
MF	GO:0005230	extracellular ligand-gated monoatomic ion channel activity	27/2054	67/18369	8.02E-10	6.16E-08	5.14E-08	27
MF	GO:0005178	integrin binding	44/2054	151/18369	1.29E-09	9.56E-08	7.98E-08	44
MF	GO:0005539	glycosaminoglycan binding	58/2054	232/18369	2.33E-09	1.66E-07	1.39E-07	58
MF	GO:0099094	ligand-gated monoatomic cation channel activity	35/2054	108/18369	2.89E-09	1.98E-07	1.65E-07	35
MF	GO:0005516	calmodulin binding	52/2054	200/18369	3.66E-09	2.43E-07	2.02E-07	52
MF	GO:0005254	chloride channel activity	27/2054	74/18369	1.05E-08	6.71E-07	5.60E-07	27
MF	GO:0019955	cytokine binding	38/2054	130/18369	1.54E-08	9.33E-07	7.79E-07	38
MF	GO:0042277	peptide binding	70/2054	317/18369	1.56E-08	9.33E-07	7.79E-07	70

MF	GO:0030020	extracellular matrix structural constituent conferring tensile strength	19/2054	41/18369	1.65E-08	9.60E-07	8.01E-07	19
MF	GO:0008066	glutamate receptor activity	15/2054	27/18369	2.38E-08	1.34E-06	1.12E-06	15
MF	GO:0015108	chloride transmembrane transporter activity	33/2054	107/18369	3.20E-08	1.74E-06	1.45E-06	33
MF	GO:0001968	fibronectin binding	16/2054	31/18369	3.27E-08	1.74E-06	1.45E-06	16
MF	GO:0001540	amyloid-beta binding	28/2054	85/18369	7.29E-08	3.78E-06	3.15E-06	28
MF	GO:0005253	monoatomic anion channel activity	28/2054	87/18369	1.27E-07	6.43E-06	5.37E-06	28
MF	GO:0033218	amide binding	79/2054	400/18369	3.02E-07	1.49E-05	1.24E-05	79
MF	GO:0003779	actin binding	84/2054	434/18369	3.16E-07	1.52E-05	1.26E-05	84
MF	GO:0016247	channel regulator activity	39/2054	152/18369	4.59E-07	2.10E-05	1.75E-05	39
MF	GO:0023023	MHC protein complex binding	16/2054	36/18369	4.60E-07	2.10E-05	1.75E-05	16
MF	GO:0015103	inorganic anion transmembrane transporter activity	37/2054	141/18369	4.92E-07	2.20E-05	1.83E-05	37
MF	GO:0008528	G protein-coupled peptide receptor activity	37/2054	142/18369	5.95E-07	2.60E-05	2.17E-05	37
MF	GO:0005543	phospholipid binding	89/2054	475/18369	6.20E-07	2.64E-05	2.21E-05	89
MF	GO:0004993	G protein-coupled serotonin receptor activity	12/2054	22/18369	8.15E-07	3.33E-05	2.78E-05	12
MF	GO:0016917	GABA receptor activity	12/2054	22/18369	8.15E-07	3.33E-05	2.78E-05	12
MF	GO:0099095	ligand-gated monoatomic anion channel activity	11/2054	19/18369	1.07E-06	4.28E-05	3.57E-05	11
MF	GO:0008509	monoatomic anion transmembrane transporter activity	33/2054	123/18369	1.17E-06	4.59E-05	3.83E-05	33
MF	GO:1902936	phosphatidylinositol bisphosphate binding	30/2054	107/18369	1.30E-06	5.00E-05	4.17E-05	30
MF	GO:0099106	ion channel regulator activity	37/2054	147/18369	1.48E-06	5.57E-05	4.65E-05	37
MF	GO:0005544	calcium-dependent phospholipid binding	19/2054	52/18369	1.53E-06	5.65E-05	4.71E-05	19
MF	GO:0015085	calcium ion transmembrane transporter activity	36/2054	142/18369	1.70E-06	6.14E-05	5.12E-05	36
MF	GO:0001653	peptide receptor activity	37/2054	148/18369	1.76E-06	6.14E-05	5.12E-05	37
MF	GO:0023026	MHC class II protein complex binding	13/2054	27/18369	1.81E-06	6.14E-05	5.12E-05	13
MF	GO:0099589	serotonin receptor activity	13/2054	27/18369	1.81E-06	6.14E-05	5.12E-05	13
MF	GO:0098631	cell adhesion mediator activity	21/2054	62/18369	1.83E-06	6.14E-05	5.12E-05	21
MF	GO:0008236	serine-type peptidase activity	44/2054	190/18369	1.85E-06	6.14E-05	5.12E-05	44
MF	GO:0048018	receptor ligand activity	90/2054	497/18369	2.40E-06	7.80E-05	6.51E-05	90
MF	GO:0008201	heparin binding	40/2054	168/18369	2.58E-06	8.26E-05	6.89E-05	40

MF	GO:0043394	proteoglycan binding	15/2054	36/18369	2.85E-06	8.98E-05	7.49E-05	15
MF	GO:0017171	serine hydrolase activity	44/2054	194/18369	3.33E-06	0.00010327	8.62E-05	44
MF	GO:0050840	extracellular matrix binding	19/2054	55/18369	4.04E-06	0.00012306	0.00010268	19
MF	GO:0099529	neurotransmitter receptor activity involved in regulation of postsynaptic membrane potential	15/2054	37/18369	4.30E-06	0.0001291	0.00010772	15
MF	GO:0004857	enzyme inhibitor activity	69/2054	359/18369	4.50E-06	0.00013299	0.00011097	69
MF	GO:0005251	delayed rectifier potassium channel activity	13/2054	29/18369	4.94E-06	0.00014376	0.00011996	13
MF	GO:0004866	endopeptidase inhibitor activity	40/2054	173/18369	5.57E-06	0.00015968	0.00013324	40
MF	GO:0005262	calcium channel activity	32/2054	126/18369	6.03E-06	0.00017033	0.00014213	32
MF	GO:0061134	peptidase regulator activity	49/2054	230/18369	6.21E-06	0.00017285	0.00014423	49
MF	GO:0005237	inhibitory extracellular ligand-gated monoatomic ion channel activity	9/2054	15/18369	7.16E-06	0.00019634	0.00016383	9
MF	GO:0001786	phosphatidylserine binding	20/2054	62/18369	7.50E-06	0.00020272	0.00016915	20
MF	GO:0004252	serine-type endopeptidase activity	39/2054	170/18369	8.84E-06	0.00023561	0.0001966	39
MF	GO:0004890	GABA-A receptor activity	10/2054	19/18369	1.06E-05	0.00027975	0.00023343	10
MF	GO:1904315	transmitter-gated monoatomic ion channel activity involved in regulation of postsynaptic membrane potential	14/2054	35/18369	1.08E-05	0.00028068	0.00023421	14
MF	GO:0098634	cell-matrix adhesion mediator activity	6/2054	7/18369	1.23E-05	0.00031111	0.0002596	6
MF	GO:0000149	SNARE binding	28/2054	107/18369	1.23E-05	0.00031111	0.0002596	28
MF	GO:0005242	inward rectifier potassium channel activity	12/2054	27/18369	1.28E-05	0.00031947	0.00026658	12
MF	GO:0030414	peptidase inhibitor activity	40/2054	180/18369	1.52E-05	0.00037531	0.00031317	40
MF	GO:0061135	endopeptidase regulator activity	41/2054	187/18369	1.69E-05	0.00041003	0.00034214	41
MF	GO:0004720	protein-lysine 6-oxidase activity	5/2054	5/18369	1.74E-05	0.00041687	0.00034785	5
MF	GO:0004896	cytokine receptor activity	25/2054	92/18369	1.76E-05	0.00041687	0.00034785	25
MF	GO:0022851	GABA-gated chloride ion channel activity	8/2054	13/18369	1.85E-05	0.00042737	0.00035661	8
MF	GO:0035325	Toll-like receptor binding	8/2054	13/18369	1.85E-05	0.00042737	0.00035661	8
MF	GO:0032395	MHC class II receptor activity	7/2054	10/18369	1.91E-05	0.00043689	0.00036455	7
MF	GO:0030246	carbohydrate binding	54/2054	273/18369	2.07E-05	0.00046766	0.00039023	54

MF	GO:1901681	sulfur compound binding	53/2054	269/18369	2.75E-05	0.00061424	0.00051254	53
MF	GO:1901981	phosphatidylinositol phosphate binding	39/2054	179/18369	3.11E-05	0.00068688	0.00057316	39
MF	GO:0015464	acetylcholine receptor activity	10/2054	21/18369	3.29E-05	0.00071724	0.00059848	10
MF	GO:0015081	sodium ion transmembrane transporter activity	35/2054	155/18369	3.56E-05	0.00076725	0.00064021	35
MF	GO:0008227	G protein-coupled amine receptor activity	14/2054	39/18369	4.56E-05	0.00097382	0.00081258	14
MF	GO:0019911	structural constituent of myelin sheath	7/2054	11/18369	4.75E-05	0.00099051	0.00082652	7
MF	GO:0099528	G protein-coupled neurotransmitter receptor activity	7/2054	11/18369	4.75E-05	0.00099051	0.00082652	7
MF	GO:0015172	acidic amino acid transmembrane transporter activity	9/2054	18/18369	5.08E-05	0.0010485	0.0008749	9
MF	GO:0005546	phosphatidylinositol-4,5-bisphosphate binding	22/2054	82/18369	6.79E-05	0.00138749	0.00115776	22
MF	GO:0044325	transmembrane transporter binding	30/2054	129/18369	7.04E-05	0.00142362	0.00118791	30
MF	GO:0097110	scaffold protein binding	19/2054	66/18369	7.44E-05	0.00148847	0.00124203	19
MF	GO:0004970	ionotropic glutamate receptor activity	9/2054	19/18369	8.69E-05	0.00172051	0.00143565	9
MF	GO:0005125	cytokine activity	46/2054	235/18369	0.00010627	0.00208205	0.00173733	46
MF	GO:0019905	syntaxin binding	19/2054	68/18369	0.00011582	0.00224616	0.00187426	19
MF	GO:0001640	adenylate cyclase inhibiting G protein-coupled glutamate receptor activity	6/2054	9/18369	0.00012076	0.00228542	0.00190702	6
MF	GO:0098988	G protein-coupled glutamate receptor activity	6/2054	9/18369	0.00012076	0.00228542	0.00190702	6
MF	GO:0072341	modified amino acid binding	22/2054	85/18369	0.00012141	0.00228542	0.00190702	22
MF	GO:0016641	oxidoreductase activity, acting on the CH-NH2 group of donors, oxygen as acceptor	8/2054	16/18369	0.00013548	0.00252544	0.00210731	8
MF	GO:0016638	oxidoreductase activity, acting on the CH-NH2 group of donors	9/2054	20/18369	0.00014238	0.00262858	0.00219337	9
MF	GO:0004222	metalloendopeptidase activity	28/2054	122/18369	0.00015388	0.00281384	0.00234795	28
MF	GO:0022810	membrane potential driven uniporter activity	4/2054	4/18369	0.00015593	0.00282441	0.00235677	4
MF	GO:0043325	phosphatidylinositol-3,4-bisphosphate binding	11/2054	29/18369	0.00016879	0.00302876	0.00252729	11

MF	GO:0005035	death receptor activity	7/2054	13/18369	0.00020134	0.00357938	0.00298674	7
MF	GO:0004713	protein tyrosine kinase activity	30/2054	137/18369	0.00022182	0.0039072	0.00326028	30
MF	GO:0035254	glutamate receptor binding	14/2054	45/18369	0.00026461	0.0046186	0.0038539	14
MF	GO:0005031	tumor necrosis factor receptor activity	6/2054	10/18369	0.00027324	0.00472627	0.00394374	6
MF	GO:0098918	structural constituent of synapse	10/2054	26/18369	0.00029246	0.00501359	0.00418349	10
MF	GO:0008467	[heparan sulfate]-glucosamine 3-sulfotransferase 1 activity	5/2054	7/18369	0.0003008	0.00506608	0.00422729	5
MF	GO:0016907	G protein-coupled acetylcholine receptor activity	5/2054	7/18369	0.0003008	0.00506608	0.00422729	5
MF	GO:0098632	cell-cell adhesion mediator activity	15/2054	51/18369	0.00032153	0.0053681	0.0044793	15
MF	GO:0019956	chemokine binding	9/2054	22/18369	0.0003424	0.00566729	0.00472895	9
MF	GO:0005313	L-glutamate transmembrane transporter activity	7/2054	14/18369	0.00036381	0.00597026	0.00498177	7
MF	GO:0004867	serine-type endopeptidase inhibitor activity	22/2054	92/18369	0.00041104	0.00668817	0.0055808	22
MF	GO:0004629	phospholipase C activity	10/2054	27/18369	0.00041824	0.00674808	0.0056308	10
MF	GO:0004175	endopeptidase activity	71/2054	428/18369	0.00042787	0.00684599	0.0057125	71
MF	GO:0051015	actin filament binding	39/2054	202/18369	0.00045629	0.00724024	0.00604147	39
MF	GO:0008503	benzodiazepine receptor activity	6/2054	11/18369	0.00054417	0.00835842	0.00697452	6
MF	GO:0042608	T cell receptor binding	6/2054	11/18369	0.00054417	0.00835842	0.00697452	6
MF	GO:0048407	platelet-derived growth factor binding	6/2054	11/18369	0.00054417	0.00835842	0.00697452	6
MF	GO:0099508	voltage-gated monoatomic ion channel activity involved in regulation of presynaptic membrane potential	6/2054	11/18369	0.00054417	0.00835842	0.00697452	6
MF	GO:0002020	protease binding	29/2054	138/18369	0.00057595	0.00877631	0.00732321	29
MF	GO:0030552	cAMP binding	8/2054	19/18369	0.00058432	0.00883387	0.00737124	8
MF	GO:0034483	heparan sulfate sulfotransferase activity	7/2054	15/18369	0.00061643	0.00924647	0.00771553	7
MF	GO:0008017	microtubule binding	48/2054	268/18369	0.00065438	0.00973966	0.00812706	48
MF	GO:0019834	phospholipase A2 inhibitor activity	4/2054	5/18369	0.00071003	0.01002863	0.00836819	4
MF	GO:0022849	glutamate-gated calcium ion channel activity	4/2054	5/18369	0.00071003	0.01002863	0.00836819	4
MF	GO:0038064	collagen receptor activity	4/2054	5/18369	0.00071003	0.01002863	0.00836819	4
MF	GO:0098639	collagen binding involved in cell-matrix adhesion	4/2054	5/18369	0.00071003	0.01002863	0.00836819	4

MF	GO:0004972	NMDA glutamate receptor activity	5/2054	8/18369	0.00072798	0.01002863	0.00836819	5
MF	GO:0031995	insulin-like growth factor II binding	5/2054	8/18369	0.00072798	0.01002863	0.00836819	5
MF	GO:0046703	natural killer cell lectin-like receptor binding	5/2054	8/18369	0.00072798	0.01002863	0.00836819	5
MF	GO:0047023	androsterone dehydrogenase activity	5/2054	8/18369	0.00072798	0.01002863	0.00836819	5
MF	GO:0004435	phosphatidylinositol phospholipase C activity	9/2054	24/18369	0.00073125	0.01002863	0.00836819	9
MF	GO:0005540	hyaluronic acid binding	9/2054	24/18369	0.00073125	0.01002863	0.00836819	9
MF	GO:0016702	oxidoreductase activity, acting on single donors with incorporation of molecular oxygen, incorporation of two atoms of oxygen	9/2054	24/18369	0.00073125	0.01002863	0.00836819	9
MF	GO:0042562	hormone binding	20/2054	84/18369	0.00078292	0.01066104	0.00889589	20
MF	GO:0003774	cytoskeletal motor activity	25/2054	115/18369	0.00079852	0.01076719	0.00898447	25
MF	GO:0042605	peptide antigen binding	12/2054	39/18369	0.00080193	0.01076719	0.00898447	12
MF	GO:0003777	microtubule motor activity	18/2054	73/18369	0.00090919	0.01212248	0.01011536	18
MF	GO:0004859	phospholipase inhibitor activity	6/2054	12/18369	0.00098546	0.01293721	0.01079519	6
MF	GO:0055102	lipase inhibitor activity	7/2054	16/18369	0.00099051	0.01293721	0.01079519	7
MF	GO:0086008	voltage-gated potassium channel activity involved in cardiac muscle cell action potential repolarization	7/2054	16/18369	0.00099051	0.01293721	0.01079519	7
MF	GO:0016701	oxidoreductase activity, acting on single donors with incorporation of molecular oxygen	9/2054	25/18369	0.00103014	0.01327427	0.01107645	9
MF	GO:0030247	polysaccharide binding	9/2054	25/18369	0.00103014	0.01327427	0.01107645	9
MF	GO:0008237	metallopeptidase activity	37/2054	197/18369	0.00107076	0.01370576	0.0114365	37
MF	GO:0035091	phosphatidylinositol binding	48/2054	275/18369	0.00116189	0.01477367	0.01232759	48
MF	GO:0099186	structural constituent of postsynapse	8/2054	21/18369	0.00128168	0.01618969	0.01350916	8
MF	GO:0005272	sodium channel activity	12/2054	41/18369	0.00131053	0.01644584	0.0137229	12
MF	GO:0005245	voltage-gated calcium channel activity	14/2054	52/18369	0.00132479	0.01649719	0.01376574	14
MF	GO:0004911	interleukin-2 receptor activity	3/2054	3/18369	0.00139631	0.01649719	0.01376574	3
MF	GO:0004999	vasoactive intestinal polypeptide receptor activity	3/2054	3/18369	0.00139631	0.01649719	0.01376574	3

MF	GO:0018636	phenanthrene 9,10-monooxygenase activity	3/2054	3/18369	0.00139631	0.01649719	0.01376574	3
MF	GO:0019976	interleukin-2 binding	3/2054	3/18369	0.00139631	0.01649719	0.01376574	3
MF	GO:0038131	neuregulin receptor activity	3/2054	3/18369	0.00139631	0.01649719	0.01376574	3
MF	GO:0042010	interleukin-15 receptor activity	3/2054	3/18369	0.00139631	0.01649719	0.01376574	3
MF	GO:0099184	structural constituent of postsynaptic intermediate filament cytoskeleton	3/2054	3/18369	0.00139631	0.01649719	0.01376574	3
MF	GO:0140788	L-glutamate uniporter activity	3/2054	3/18369	0.00139631	0.01649719	0.01376574	3
MF	GO:0042805	actinin binding	11/2054	36/18369	0.00140054	0.01649719	0.01376574	11
MF	GO:0005154	epidermal growth factor receptor binding	10/2054	31/18369	0.00144726	0.01684086	0.01405252	10
MF	GO:0005231	excitatory extracellular ligand-gated monoatomic ion channel activity	10/2054	31/18369	0.00144726	0.01684086	0.01405252	10
MF	GO:0005222	intracellular cAMP-activated cation channel activity	5/2054	9/18369	0.001487	0.01699433	0.01418057	5
MF	GO:0047044	androstan-3-alpha,17-beta-diol dehydrogenase activity	5/2054	9/18369	0.001487	0.01699433	0.01418057	5
MF	GO:0047844	deoxycytidine deaminase activity	5/2054	9/18369	0.001487	0.01699433	0.01418057	5
MF	GO:0031420	alkali metal ion binding	7/2054	17/18369	0.00152227	0.01719267	0.01434608	7
MF	GO:0099507	ligand-gated monoatomic ion channel activity involved in regulation of presynaptic membrane potential	7/2054	17/18369	0.00152227	0.01719267	0.01434608	7
MF	GO:0015459	potassium channel regulator activity	14/2054	53/18369	0.00161797	0.01816668	0.01515882	14
MF	GO:0001618	virus receptor activity	18/2054	77/18369	0.00175243	0.01956201	0.01632312	18
MF	GO:0030021	extracellular matrix structural constituent conferring compression resistance	8/2054	22/18369	0.00181842	0.01992094	0.01662263	8
MF	GO:0031681	G-protein beta-subunit binding	8/2054	22/18369	0.00181842	0.01992094	0.01662263	8
MF	GO:0035014	phosphatidylinositol 3-kinase regulator activity	8/2054	22/18369	0.00181842	0.01992094	0.01662263	8
MF	GO:0005044	scavenger receptor activity	13/2054	48/18369	0.00183646	0.01992094	0.01662263	13
MF	GO:0015026	coreceptor activity	13/2054	48/18369	0.00183646	0.01992094	0.01662263	13
MF	GO:0001875	lipopolysaccharide immune receptor activity	4/2054	6/18369	0.00194064	0.02081585	0.01736937	4

MF	GO:0097200	cysteine-type endopeptidase activity involved in execution phase of apoptosis	4/2054	6/18369	0.00194064	0.02081585	0.01736937	4
MF	GO:0005246	calcium channel regulator activity	14/2054	54/18369	0.00196317	0.0209405	0.01747337	14
MF	GO:0140272	exogenous protein binding	18/2054	78/18369	0.00204452	0.02168774	0.0180969	18
MF	GO:0098641	cadherin binding involved in cell-cell adhesion	7/2054	18/18369	0.00225242	0.02376175	0.01982751	7
MF	GO:0030551	cyclic nucleotide binding	10/2054	33/18369	0.00245244	0.0257305	0.02147029	10
MF	GO:0005416	amino acid:monoatomic cation symporter activity	9/2054	28/18369	0.00255474	0.02651403	0.0221241	9
MF	GO:0043236	laminin binding	9/2054	28/18369	0.00255474	0.02651403	0.0221241	9
MF	GO:0015271	outward rectifier potassium channel activity	6/2054	14/18369	0.00262788	0.02686776	0.02241926	6
MF	GO:0005432	calcium:sodium antiporter activity	5/2054	10/18369	0.00270077	0.02686776	0.02241926	5
MF	GO:0015183	L-aspartate transmembrane transporter activity	5/2054	10/18369	0.00270077	0.02686776	0.02241926	5
MF	GO:0016176	superoxide-generating NADPH oxidase activator activity	5/2054	10/18369	0.00270077	0.02686776	0.02241926	5
MF	GO:0030023	extracellular matrix constituent conferring elasticity	5/2054	10/18369	0.00270077	0.02686776	0.02241926	5
MF	GO:0032052	bile acid binding	5/2054	10/18369	0.00270077	0.02686776	0.02241926	5
MF	GO:0034987	immunoglobulin receptor binding	5/2054	10/18369	0.00270077	0.02686776	0.02241926	5
MF	GO:0051378	serotonin binding	5/2054	10/18369	0.00270077	0.02686776	0.02241926	5
MF	GO:0005507	copper ion binding	15/2054	62/18369	0.00287949	0.02835187	0.02365765	15
MF	GO:0048306	calcium-dependent protein binding	15/2054	62/18369	0.00287949	0.02835187	0.02365765	15
MF	GO:0005248	voltage-gated sodium channel activity	7/2054	19/18369	0.00322547	0.03112011	0.02596755	7
MF	GO:0005326	neurotransmitter transmembrane transporter activity	7/2054	19/18369	0.00322547	0.03112011	0.02596755	7
MF	GO:0046935	1-phosphatidylinositol-3-kinase regulator activity	7/2054	19/18369	0.00322547	0.03112011	0.02596755	7
MF	GO:0061778	intracellular chloride channel activity	7/2054	19/18369	0.00322547	0.03112011	0.02596755	7
MF	GO:0004089	carbonate dehydratase activity	6/2054	15/18369	0.00396885	0.03791138	0.03163439	6
MF	GO:0005005	transmembrane-ephrin receptor activity	6/2054	15/18369	0.00396885	0.03791138	0.03163439	6
MF	GO:0015292	uniporter activity	4/2054	7/18369	0.00412711	0.03922793	0.03273296	4

MF	GO:0015171	amino acid transmembrane transporter activity	18/2054	83/18369	0.00418827	0.039419	0.03289239	18
MF	GO:0038024	cargo receptor activity	18/2054	83/18369	0.00418827	0.039419	0.03289239	18
MF	GO:0005221	intracellular cyclic nucleotide activated monoatomic cation channel activity	5/2054	11/18369	0.00449794	0.04132076	0.03447928	5
MF	GO:0015368	calcium:monoatomic cation antiporter activity	5/2054	11/18369	0.00449794	0.04132076	0.03447928	5
MF	GO:0019864	IgG binding	5/2054	11/18369	0.00449794	0.04132076	0.03447928	5
MF	GO:0043176	amine binding	5/2054	11/18369	0.00449794	0.04132076	0.03447928	5
MF	GO:0043855	cyclic nucleotide-gated monoatomic ion channel activity	5/2054	11/18369	0.00449794	0.04132076	0.03447928	5
MF	GO:0035255	ionotropic glutamate receptor binding	8/2054	25/18369	0.0045316	0.0414318	0.03457193	8
MF	GO:0070851	growth factor receptor binding	26/2054	137/18369	0.00476931	0.04339843	0.03621294	26
MF	GO:0015293	symporter activity	27/2054	144/18369	0.00484071	0.04384041	0.03658174	27
MF	GO:0035662	Toll-like receptor 4 binding	3/2054	4/18369	0.00511744	0.04569991	0.03813337	3
MF	GO:0086089	voltage-gated potassium channel activity involved in atrial cardiac muscle cell action potential repolarization	3/2054	4/18369	0.00511744	0.04569991	0.03813337	3
MF	GO:0099530	G protein-coupled receptor activity involved in regulation of postsynaptic membrane potential	3/2054	4/18369	0.00511744	0.04569991	0.03813337	3
MF	GO:0004714	transmembrane receptor protein tyrosine kinase activity	14/2054	60/18369	0.00554246	0.04926634	0.04110931	14

Table S15 GO terms based on DEGs between high and low SEPN1 expression groups in GSE16011.

ONTOLOGY	ID	Description	GeneRatio	BgRatio	P-value	P-adjust	Q-value	Count
BP	GO:0030198	extracellular matrix organization	36/396	314/18614	1.61E-16	3.77E-13	2.83E-13	36
BP	GO:0043062	extracellular structure organization	36/396	315/18614	1.79E-16	3.77E-13	2.83E-13	36
BP	GO:0045229	external encapsulating structure organization	36/396	317/18614	2.19E-16	3.77E-13	2.83E-13	36
BP	GO:0042060	wound healing	41/396	439/18614	1.69E-15	2.18E-12	1.64E-12	41
BP	GO:0050808	synapse organization	40/396	466/18614	6.32E-14	6.52E-11	4.90E-11	40
BP	GO:0030199	collagen fibril organization	16/396	64/18614	2.51E-13	2.16E-10	1.62E-10	16
BP	GO:0031589	cell-substrate adhesion	31/396	359/18614	4.11E-11	3.03E-08	2.28E-08	31
BP	GO:1903034	regulation of response to wounding	21/396	170/18614	9.30E-11	6.00E-08	4.51E-08	21
BP	GO:0071559	response to transforming growth factor beta	26/396	280/18614	3.37E-10	1.93E-07	1.45E-07	26
BP	GO:0007178	transmembrane receptor protein serine/threonine kinase signaling pathway	31/396	398/18614	5.40E-10	2.79E-07	2.10E-07	31
BP	GO:0071560	cellular response to transforming growth factor beta stimulus	25/396	274/18614	1.07E-09	5.03E-07	3.78E-07	25
BP	GO:0001503	ossification	31/396	429/18614	3.31E-09	1.42E-06	1.07E-06	31
BP	GO:0001649	osteoblast differentiation	23/396	250/18614	4.30E-09	1.71E-06	1.29E-06	23
BP	GO:0061041	regulation of wound healing	17/396	137/18614	5.46E-09	2.01E-06	1.51E-06	17
BP	GO:1903036	positive regulation of response to wounding	13/396	76/18614	6.83E-09	2.35E-06	1.77E-06	13
BP	GO:0007179	transforming growth factor beta receptor signaling pathway	21/396	218/18614	9.09E-09	2.93E-06	2.21E-06	21
BP	GO:0045785	positive regulation of cell adhesion	32/396	482/18614	1.41E-08	4.29E-06	3.23E-06	32
BP	GO:0099177	regulation of trans-synaptic signaling	32/396	488/18614	1.89E-08	5.43E-06	4.09E-06	32
BP	GO:0006958	complement activation, classical pathway	9/396	33/18614	2.00E-08	5.44E-06	4.09E-06	9
BP	GO:0030193	regulation of blood coagulation	12/396	70/18614	2.55E-08	6.57E-06	4.94E-06	12
BP	GO:1900046	regulation of hemostasis	12/396	72/18614	3.54E-08	8.70E-06	6.55E-06	12
BP	GO:0097530	granulocyte migration	17/396	156/18614	3.92E-08	9.19E-06	6.91E-06	17
BP	GO:0007160	cell-matrix adhesion	21/396	239/18614	4.56E-08	1.02E-05	7.70E-06	21
BP	GO:0010755	regulation of plasminogen activation	7/396	18/18614	4.86E-08	1.05E-05	7.87E-06	7
BP	GO:0050818	regulation of coagulation	12/396	75/18614	5.68E-08	1.17E-05	8.83E-06	12
BP	GO:0050804	modulation of chemical synaptic transmission	31/396	487/18614	6.25E-08	1.24E-05	9.33E-06	31

BP	GO:0090303	positive regulation of wound healing	11/396	62/18614	6.76E-08	1.29E-05	9.72E-06	11
BP	GO:0007596	blood coagulation	20/396	224/18614	7.34E-08	1.35E-05	1.02E-05	20
BP	GO:0050890	cognition	24/396	317/18614	8.66E-08	1.54E-05	1.16E-05	24
BP	GO:0002685	regulation of leukocyte migration	20/396	227/18614	9.15E-08	1.57E-05	1.18E-05	20
BP	GO:0050900	leukocyte migration	27/396	393/18614	9.92E-08	1.65E-05	1.24E-05	27
BP	GO:0050817	coagulation	20/396	229/18614	1.06E-07	1.71E-05	1.28E-05	20
BP	GO:0007599	hemostasis	20/396	230/18614	1.14E-07	1.78E-05	1.34E-05	20
BP	GO:0007611	learning or memory	22/396	276/18614	1.23E-07	1.87E-05	1.41E-05	22
BP	GO:0097529	myeloid leukocyte migration	20/396	241/18614	2.43E-07	3.58E-05	2.69E-05	20
BP	GO:0001706	endoderm formation	10/396	57/18614	3.00E-07	4.22E-05	3.17E-05	10
BP	GO:0010810	regulation of cell-substrate adhesion	19/396	222/18614	3.02E-07	4.22E-05	3.17E-05	19
BP	GO:0006956	complement activation	10/396	58/18614	3.56E-07	4.84E-05	3.64E-05	10
BP	GO:0042063	gliogenesis	23/396	320/18614	4.07E-07	5.39E-05	4.05E-05	23
BP	GO:1903053	regulation of extracellular matrix organization	10/396	59/18614	4.21E-07	5.43E-05	4.08E-05	10
BP	GO:0060395	SMAD protein signal transduction	12/396	90/18614	4.47E-07	5.50E-05	4.14E-05	12
BP	GO:0002455	humoral immune response mediated by circulating immunoglobulin	9/396	46/18614	4.48E-07	5.50E-05	4.14E-05	9
BP	GO:0016064	immunoglobulin mediated immune response	15/396	145/18614	4.90E-07	5.88E-05	4.42E-05	15
BP	GO:0019724	B cell mediated immunity	15/396	148/18614	6.38E-07	7.31E-05	5.50E-05	15
BP	GO:1903035	negative regulation of response to wounding	12/396	93/18614	6.42E-07	7.31E-05	5.50E-05	12
BP	GO:0035987	endodermal cell differentiation	9/396	48/18614	6.56E-07	7.31E-05	5.50E-05	9
BP	GO:0034612	response to tumor necrosis factor	20/396	257/18614	6.78E-07	7.31E-05	5.50E-05	20
BP	GO:0042391	regulation of membrane potential	27/396	433/18614	6.80E-07	7.31E-05	5.50E-05	27
BP	GO:0071621	granulocyte chemotaxis	14/396	131/18614	7.93E-07	8.35E-05	6.28E-05	14
BP	GO:0070482	response to oxygen levels	23/396	333/18614	8.16E-07	8.42E-05	6.34E-05	23
BP	GO:0031639	plasminogen activation	7/396	26/18614	8.68E-07	8.78E-05	6.61E-05	7
BP	GO:0007612	learning	15/396	152/18614	8.99E-07	8.93E-05	6.71E-05	15
BP	GO:0048251	elastic fiber assembly	5/396	10/18614	9.81E-07	9.55E-05	7.19E-05	5
BP	GO:0034329	cell junction assembly	27/396	444/18614	1.10E-06	0.00010541	7.93E-05	27
BP	GO:0042730	fibrinolysis	7/396	27/18614	1.15E-06	0.00010638	8.00E-05	7
BP	GO:0008037	cell recognition	15/396	155/18614	1.15E-06	0.00010638	8.00E-05	15
BP	GO:0030194	positive regulation of blood coagulation	7/396	28/18614	1.51E-06	0.00013401	0.00010081	7
BP	GO:1900048	positive regulation of hemostasis	7/396	28/18614	1.51E-06	0.00013401	0.00010081	7

BP	GO:0045766	positive regulation of angiogenesis	16/396	185/18614	2.28E-06	0.0001959	0.00014737	16
BP	GO:1904018	positive regulation of vasculature development	16/396	185/18614	2.28E-06	0.0001959	0.00014737	16
BP	GO:0050820	positive regulation of coagulation	7/396	30/18614	2.50E-06	0.00021118	0.00015886	7
BP	GO:0006898	receptor-mediated endocytosis	19/396	256/18614	2.61E-06	0.00021729	0.00016346	19
BP	GO:0001704	formation of primary germ layer	13/396	127/18614	3.19E-06	0.00025993	0.00019553	13
BP	GO:0050878	regulation of body fluid levels	23/396	361/18614	3.22E-06	0.00025993	0.00019553	23
BP	GO:0010718	positive regulation of epithelial to mesenchymal transition	9/396	58/18614	3.45E-06	0.00027436	0.00020639	9
BP	GO:0060326	cell chemotaxis	21/396	312/18614	3.70E-06	0.00028906	0.00021745	21
BP	GO:0017157	regulation of exocytosis	16/396	194/18614	4.22E-06	0.0003255	0.00024486	16
BP	GO:0007229	integrin-mediated signaling pathway	12/396	112/18614	4.74E-06	0.00035958	0.0002705	12
BP	GO:0007416	synapse assembly	16/396	197/18614	5.15E-06	0.00038361	0.00028857	16
BP	GO:0051346	negative regulation of hydrolase activity	22/396	345/18614	5.20E-06	0.00038361	0.00028857	22
BP	GO:0045765	regulation of angiogenesis	22/396	349/18614	6.25E-06	0.00045455	0.00034194	22
BP	GO:0071420	cellular response to histamine	4/396	7/18614	6.71E-06	0.00048127	0.00036204	4
BP	GO:0051960	regulation of nervous system development	26/396	461/18614	6.85E-06	0.00048407	0.00036414	26
BP	GO:0031638	zymogen activation	9/396	63/18614	6.99E-06	0.0004877	0.00036687	9
BP	GO:0022407	regulation of cell-cell adhesion	27/396	491/18614	7.23E-06	0.00049751	0.00037426	27
BP	GO:0061448	connective tissue development	19/396	275/18614	7.39E-06	0.00050167	0.00037739	19
BP	GO:0051216	cartilage development	16/396	203/18614	7.55E-06	0.00050627	0.00038085	16
BP	GO:1901342	regulation of vasculature development	22/396	355/18614	8.19E-06	0.00054204	0.00040775	22
BP	GO:0017156	calcium-ion regulated exocytosis	9/396	65/18614	9.10E-06	0.00059433	0.00044709	9
BP	GO:0030195	negative regulation of blood coagulation	8/396	50/18614	9.61E-06	0.00061972	0.00046619	8
BP	GO:0036293	response to decreased oxygen levels	20/396	306/18614	9.72E-06	0.00061972	0.00046619	20
BP	GO:0007492	endoderm development	10/396	83/18614	1.03E-05	0.00065143	0.00049005	10
BP	GO:0098883	synapse pruning	5/396	15/18614	1.07E-05	0.00066565	0.00050074	5
BP	GO:1900047	negative regulation of hemostasis	8/396	51/18614	1.12E-05	0.00068734	0.00051706	8
BP	GO:0010001	glial cell differentiation	17/396	235/18614	1.23E-05	0.00073703	0.00055444	17
BP	GO:0071356	cellular response to tumor necrosis factor	17/396	235/18614	1.23E-05	0.00073703	0.00055444	17

BP	GO:0010466	negative regulation of peptidase activity	17/396	236/18614	1.30E-05	0.00077009	0.00057931	17
BP	GO:0010757	negative regulation of plasminogen activation	4/396	8/18614	1.32E-05	0.00077429	0.00058246	4
BP	GO:0007613	memory	12/396	124/18614	1.36E-05	0.00078794	0.00059274	12
BP	GO:0010038	response to metal ion	22/396	367/18614	1.38E-05	0.00079044	0.00059462	22
BP	GO:0031099	regeneration	15/396	190/18614	1.42E-05	0.00080487	0.00060547	15
BP	GO:0010975	regulation of neuron projection development	25/396	452/18614	1.43E-05	0.00080487	0.00060547	25
BP	GO:0010811	positive regulation of cell-substrate adhesion	12/396	125/18614	1.47E-05	0.00080968	0.00060909	12
BP	GO:0031623	receptor internalization	12/396	125/18614	1.47E-05	0.00080968	0.00060909	12
BP	GO:0052547	regulation of peptidase activity	24/396	425/18614	1.52E-05	0.00082437	0.00062014	24
BP	GO:0033627	cell adhesion mediated by integrin	10/396	87/18614	1.58E-05	0.00083924	0.00063132	10
BP	GO:0048708	astrocyte differentiation	10/396	87/18614	1.58E-05	0.00083924	0.00063132	10
BP	GO:0045667	regulation of osteoblast differentiation	13/396	147/18614	1.59E-05	0.00083924	0.00063132	13
BP	GO:0030595	leukocyte chemotaxis	17/396	241/18614	1.70E-05	0.00088861	0.00066846	17
BP	GO:0050819	negative regulation of coagulation	8/396	54/18614	1.73E-05	0.00089237	0.00067129	8
BP	GO:0001666	response to hypoxia	19/396	293/18614	1.81E-05	0.00092457	0.00069552	19
BP	GO:0051592	response to calcium ion	13/396	149/18614	1.84E-05	0.00093213	0.0007012	13
BP	GO:0048246	macrophage chemotaxis	7/396	41/18614	2.25E-05	0.00111879	0.00084162	7
BP	GO:0007214	gamma-aminobutyric acid signaling pathway	6/396	28/18614	2.26E-05	0.00111879	0.00084162	6
BP	GO:0010717	regulation of epithelial to mesenchymal transition	11/396	110/18614	2.28E-05	0.00111879	0.00084162	11
BP	GO:0099504	synaptic vesicle cycle	15/396	198/18614	2.32E-05	0.00112927	0.00084951	15
BP	GO:0051932	synaptic transmission, GABAergic	8/396	57/18614	2.60E-05	0.0012456	0.00093701	8
BP	GO:0090092	regulation of transmembrane receptor protein serine/threonine kinase signaling pathway	19/396	301/18614	2.63E-05	0.0012456	0.00093701	19
BP	GO:1902074	response to salt	22/396	383/18614	2.65E-05	0.0012456	0.00093701	22
BP	GO:0085029	extracellular matrix assembly	7/396	42/18614	2.65E-05	0.0012456	0.00093701	7
BP	GO:0035249	synaptic transmission, glutamatergic	11/396	112/18614	2.70E-05	0.00125423	0.0009435	11
BP	GO:0031349	positive regulation of defense response	24/396	441/18614	2.76E-05	0.00127435	0.00095864	24
BP	GO:0035640	exploration behavior	6/396	29/18614	2.80E-05	0.00127945	0.00096248	6
BP	GO:0051917	regulation of fibrinolysis	5/396	18/18614	2.90E-05	0.00130033	0.00097819	5
BP	GO:0090594	inflammatory response to wounding	5/396	18/18614	2.90E-05	0.00130033	0.00097819	5

BP	GO:0032102	negative regulation of response to external stimulus	24/396	446/18614	3.31E-05	0.00147398	0.00110882	24
BP	GO:0043588	skin development	19/396	308/18614	3.60E-05	0.00158771	0.00119437	19
BP	GO:0061564	axon development	25/396	479/18614	3.76E-05	0.00163206	0.00122773	25
BP	GO:2000097	regulation of smooth muscle cell-matrix adhesion	3/396	4/18614	3.76E-05	0.00163206	0.00122773	3
BP	GO:0010759	positive regulation of macrophage chemotaxis	5/396	19/18614	3.86E-05	0.00164804	0.00123975	5
BP	GO:1904862	inhibitory synapse assembly	5/396	19/18614	3.86E-05	0.00164804	0.00123975	5
BP	GO:0010951	negative regulation of endopeptidase activity	13/396	160/18614	3.91E-05	0.00165607	0.00124579	13
BP	GO:0061045	negative regulation of wound healing	9/396	78/18614	4.07E-05	0.0017076	0.00128455	9
BP	GO:0002460	adaptive immune response based on somatic recombination of immune receptors built from immunoglobulin superfamily domains	19/396	311/18614	4.11E-05	0.00170891	0.00128554	19
BP	GO:1905517	macrophage migration	8/396	61/18614	4.30E-05	0.00177488	0.00133517	8
BP	GO:0001505	regulation of neurotransmitter levels	15/396	211/18614	4.85E-05	0.0019878	0.00149534	15
BP	GO:0008038	neuron recognition	7/396	46/18614	4.89E-05	0.00198953	0.00149664	7
BP	GO:0050673	epithelial cell proliferation	25/396	488/18614	5.09E-05	0.00205204	0.00154366	25
BP	GO:0002443	leukocyte mediated immunity	22/396	401/18614	5.29E-05	0.00211512	0.00159111	22
BP	GO:0060393	regulation of pathway-restricted SMAD protein phosphorylation	8/396	63/18614	5.45E-05	0.00216294	0.00162709	8
BP	GO:0050921	positive regulation of chemotaxis	12/396	143/18614	5.64E-05	0.00222393	0.00167297	12
BP	GO:0034776	response to histamine	4/396	11/18614	5.91E-05	0.00231292	0.00173991	4
BP	GO:0009612	response to mechanical stimulus	15/396	215/18614	6.01E-05	0.00233385	0.00175566	15
BP	GO:0071711	basement membrane organization	6/396	33/18614	6.08E-05	0.00234119	0.00176118	6
BP	GO:0002253	activation of immune response	25/396	495/18614	6.40E-05	0.00244608	0.00184008	25
BP	GO:0048791	calcium ion-regulated exocytosis of neurotransmitter	5/396	21/18614	6.53E-05	0.00247752	0.00186373	5
BP	GO:0007568	aging	13/396	169/18614	6.90E-05	0.00259032	0.00194859	13
BP	GO:0060079	excitatory postsynaptic potential	11/396	124/18614	6.92E-05	0.00259032	0.00194859	11

BP	GO:0050767	regulation of neurogenesis	21/396	381/18614	7.30E-05	0.00271127	0.00203957	21
BP	GO:0002687	positive regulation of leukocyte migration	12/396	147/18614	7.38E-05	0.0027213	0.00204712	12
BP	GO:0099003	vesicle-mediated transport in synapse	15/396	220/18614	7.80E-05	0.00284471	0.00213996	15
BP	GO:0017015	regulation of transforming growth factor beta receptor signaling pathway	12/396	148/18614	7.88E-05	0.00284471	0.00213996	12
BP	GO:0060078	regulation of postsynaptic membrane potential	12/396	148/18614	7.88E-05	0.00284471	0.00213996	12
BP	GO:0002449	lymphocyte mediated immunity	18/396	300/18614	8.21E-05	0.0029347	0.00220766	18
BP	GO:0001837	epithelial to mesenchymal transition	13/396	172/18614	8.26E-05	0.0029347	0.00220766	13
BP	GO:2001044	regulation of integrin-mediated signaling pathway	5/396	22/18614	8.30E-05	0.0029347	0.00220766	5
BP	GO:1902041	regulation of extrinsic apoptotic signaling pathway via death domain receptors	7/396	50/18614	8.49E-05	0.0029516	0.00222037	7
BP	GO:0060389	pathway-restricted SMAD protein phosphorylation	8/396	67/18614	8.52E-05	0.0029516	0.00222037	8
BP	GO:0070613	regulation of protein processing	8/396	67/18614	8.52E-05	0.0029516	0.00222037	8
BP	GO:0018149	peptide cross-linking	6/396	35/18614	8.59E-05	0.00295699	0.00222442	6
BP	GO:0008306	associative learning	9/396	86/18614	8.84E-05	0.00302091	0.00227251	9
BP	GO:0045861	negative regulation of proteolysis	19/396	330/18614	9.06E-05	0.00307779	0.00231529	19
BP	GO:1990266	neutrophil migration	11/396	128/18614	9.24E-05	0.00310297	0.00233424	11
BP	GO:0018057	peptidyl-lysine oxidation	3/396	5/18614	9.26E-05	0.00310297	0.00233424	3
BP	GO:1903844	regulation of cellular response to transforming growth factor beta stimulus	12/396	151/18614	9.56E-05	0.0031781	0.00239075	12
BP	GO:0006959	humoral immune response	16/396	250/18614	9.60E-05	0.0031781	0.00239075	16
BP	GO:0150063	visual system development	21/396	389/18614	9.79E-05	0.00320547	0.00241134	21
BP	GO:0051604	protein maturation	19/396	332/18614	9.81E-05	0.00320547	0.00241134	19
BP	GO:0097484	dendrite extension	6/396	36/18614	0.00010128	0.00328801	0.00247344	6
BP	GO:1903861	positive regulation of dendrite extension	5/396	23/18614	0.00010422	0.00336236	0.00252936	5
BP	GO:0007215	glutamate receptor signaling pathway	7/396	52/18614	0.00010965	0.00351573	0.00264474	7

BP	GO:0052548	regulation of endopeptidase activity	19/396	336/18614	0.00011474	0.0036538	0.0027486	19
BP	GO:0050729	positive regulation of inflammatory response	12/396	154/18614	0.00011538	0.0036538	0.0027486	12
BP	GO:0044319	wound healing, spreading of cells	6/396	37/18614	0.00011873	0.00371448	0.00279425	6
BP	GO:0090505	epiboly involved in wound healing	6/396	37/18614	0.00011873	0.00371448	0.00279425	6
BP	GO:0048880	sensory system development	21/396	395/18614	0.00012117	0.00374344	0.00281604	21
BP	GO:0002237	response to molecule of bacterial origin	20/396	366/18614	0.00012134	0.00374344	0.00281604	20
BP	GO:0099565	chemical synaptic transmission, postsynaptic	11/396	132/18614	0.00012183	0.00374344	0.00281604	11
BP	GO:0033690	positive regulation of osteoblast proliferation	4/396	13/18614	0.00012389	0.00374493	0.00281716	4
BP	GO:0051918	negative regulation of fibrinolysis	4/396	13/18614	0.00012389	0.00374493	0.00281716	4
BP	GO:0071622	regulation of granulocyte chemotaxis	7/396	53/18614	0.00012406	0.00374493	0.00281716	7
BP	GO:0060537	muscle tissue development	22/396	426/18614	0.00012748	0.00378249	0.00284541	22
BP	GO:0050920	regulation of chemotaxis	15/396	230/18614	0.00012819	0.00378249	0.00284541	15
BP	GO:0048545	response to steroid hormone	19/396	339/18614	0.0001288	0.00378249	0.00284541	19
BP	GO:1903317	regulation of protein maturation	8/396	71/18614	0.00012908	0.00378249	0.00284541	8
BP	GO:0031338	regulation of vesicle fusion	5/396	24/18614	0.00012936	0.00378249	0.00284541	5
BP	GO:0002683	negative regulation of immune system process	24/396	487/18614	0.00013004	0.00378249	0.00284541	24
BP	GO:0007584	response to nutrient	12/396	156/18614	0.00013043	0.00378249	0.00284541	12
BP	GO:0007162	negative regulation of cell adhesion	18/396	312/18614	0.00013466	0.0038834	0.00292132	18
BP	GO:0001819	positive regulation of cytokine production	24/396	489/18614	0.00013834	0.0039206	0.0029493	24
BP	GO:0090504	epiboly	6/396	38/18614	0.00013849	0.0039206	0.0029493	6
BP	GO:0010976	positive regulation of neuron projection development	12/396	157/18614	0.00013857	0.0039206	0.0029493	12
BP	GO:0070371	ERK1 and ERK2 cascade	19/396	341/18614	0.00013899	0.0039206	0.0029493	19
BP	GO:0086002	cardiac muscle cell action potential involved in contraction	7/396	54/18614	0.00013996	0.00392646	0.00295371	7
BP	GO:0002062	chondrocyte differentiation	10/396	113/18614	0.0001502	0.00416857	0.00313584	10

BP	GO:0044344	cellular response to fibroblast growth factor stimulus	10/396	113/18614	0.0001502	0.00416857	0.00313584	10
BP	GO:0060560	developmental growth involved in morphogenesis	15/396	234/18614	0.00015502	0.00427932	0.00321915	15
BP	GO:0070997	neuron death	20/396	373/18614	0.00015635	0.00429273	0.00322924	20
BP	GO:0060415	muscle tissue morphogenesis	8/396	73/18614	0.00015717	0.00429273	0.00322924	8
BP	GO:0032496	response to lipopolysaccharide	19/396	345/18614	0.00016153	0.00434278	0.00326689	19
BP	GO:0090287	regulation of cellular response to growth factor stimulus	19/396	345/18614	0.00016153	0.00434278	0.00326689	19
BP	GO:1990778	protein localization to cell periphery	19/396	345/18614	0.00016153	0.00434278	0.00326689	19
BP	GO:0099173	postsynapse organization	13/396	185/18614	0.00017188	0.00459724	0.00345831	13
BP	GO:0021700	developmental maturation	18/396	319/18614	0.00017737	0.00471942	0.00355023	18
BP	GO:0045112	integrin biosynthetic process	3/396	6/18614	0.00018222	0.00479903	0.00361011	3
BP	GO:0061302	smooth muscle cell-matrix adhesion	3/396	6/18614	0.00018222	0.00479903	0.00361011	3
BP	GO:0045669	positive regulation of osteoblast differentiation	8/396	75/18614	0.00019012	0.00498176	0.00374757	8
BP	GO:0090090	negative regulation of canonical Wnt signaling pathway	11/396	139/18614	0.00019267	0.00498945	0.00375336	11
BP	GO:1903859	regulation of dendrite extension	5/396	26/18614	0.00019331	0.00498945	0.00375336	5
BP	GO:1905523	positive regulation of macrophage migration	5/396	26/18614	0.00019331	0.00498945	0.00375336	5
BP	GO:0006887	exocytosis	19/396	351/18614	0.00020136	0.00514567	0.00387087	19
BP	GO:0007517	muscle organ development	19/396	351/18614	0.00020136	0.00514567	0.00387087	19
BP	GO:0086003	cardiac muscle cell contraction	8/396	76/18614	0.00020861	0.00530456	0.0039904	8
BP	GO:0002691	regulation of cellular extravasation	6/396	41/18614	0.00021376	0.00540892	0.0040689	6
BP	GO:0034109	homotypic cell-cell adhesion	9/396	97/18614	0.00022374	0.00563386	0.00423812	9
BP	GO:0050727	regulation of inflammatory response	21/396	414/18614	0.00023064	0.00577941	0.00434761	21
BP	GO:0051384	response to glucocorticoid	11/396	142/18614	0.00023231	0.00579304	0.00435787	11
BP	GO:0031960	response to corticosteroid	12/396	166/18614	0.00023356	0.00579643	0.00436042	12
BP	GO:0007159	leukocyte cell-cell adhesion	21/396	415/18614	0.00023827	0.00588497	0.00442701	21

BP	GO:1901214	regulation of neuron death	18/396	327/18614	0.0002403	0.00590682	0.00444345	18
BP	GO:1902476	chloride transmembrane transport	9/396	98/18614	0.00024182	0.00591605	0.0044504	9
BP	GO:0031102	neuron projection regeneration	7/396	59/18614	0.00024614	0.00596748	0.00448909	7
BP	GO:0071774	response to fibroblast growth factor	10/396	120/18614	0.00024624	0.00596748	0.00448909	10
BP	GO:0022604	regulation of cell morphogenesis	15/396	245/18614	0.00025538	0.00616022	0.00463408	15
BP	GO:0086001	cardiac muscle cell action potential	8/396	79/18614	0.0002731	0.00655697	0.00493254	8
BP	GO:0071695	anatomical structure maturation	15/396	247/18614	0.00027866	0.00656127	0.00493577	15
BP	GO:0010758	regulation of macrophage chemotaxis	5/396	28/18614	0.0002789	0.00656127	0.00493577	5
BP	GO:0150146	cell junction disassembly	5/396	28/18614	0.0002789	0.00656127	0.00493577	5
BP	GO:1903055	positive regulation of extracellular matrix organization	5/396	28/18614	0.0002789	0.00656127	0.00493577	5
BP	GO:0014002	astrocyte development	6/396	43/18614	0.00027964	0.00656127	0.00493577	6
BP	GO:0007009	plasma membrane organization	12/396	170/18614	0.00029095	0.00679593	0.0051123	12
BP	GO:0051051	negative regulation of transport	23/396	483/18614	0.00029283	0.00680884	0.00512201	23
BP	GO:0048644	muscle organ morphogenesis	8/396	80/18614	0.00029791	0.00686518	0.00516439	8
BP	GO:0051966	regulation of synaptic transmission, glutamatergic	8/396	80/18614	0.00029791	0.00686518	0.00516439	8
BP	GO:0007369	gastrulation	13/396	196/18614	0.00030277	0.00690209	0.00519215	13
BP	GO:0043903	regulation of biological process involved in symbiotic interaction	7/396	61/18614	0.00030352	0.00690209	0.00519215	7
BP	GO:0098900	regulation of action potential	7/396	61/18614	0.00030352	0.00690209	0.00519215	7
BP	GO:1903224	regulation of endodermal cell differentiation	3/396	7/18614	0.00031385	0.00704382	0.00529877	3
BP	GO:2000391	positive regulation of neutrophil extravasation	3/396	7/18614	0.00031385	0.00704382	0.00529877	3
BP	GO:2000535	regulation of entry of bacterium into host cell	3/396	7/18614	0.00031385	0.00704382	0.00529877	3
BP	GO:0016485	protein processing	15/396	250/18614	0.00031701	0.0070766	0.00532343	15
BP	GO:1905521	regulation of macrophage migration	6/396	44/18614	0.00031805	0.0070766	0.00532343	6
BP	GO:0010955	negative regulation of protein processing	5/396	29/18614	0.00033117	0.00730563	0.00549572	5
BP	GO:1903318	negative regulation of protein maturation	5/396	29/18614	0.00033117	0.00730563	0.00549572	5

BP	GO:0007411	axon guidance	14/396	225/18614	0.0003456	0.0075592	0.00568647	14
BP	GO:0097485	neuron projection guidance	14/396	225/18614	0.0003456	0.0075592	0.00568647	14
BP	GO:1902622	regulation of neutrophil migration	6/396	45/18614	0.00036048	0.00785142	0.0059063	6
BP	GO:0002688	regulation of leukocyte chemotaxis	10/396	126/18614	0.00036526	0.00792206	0.00595944	10
BP	GO:0031644	regulation of nervous system process	11/396	150/18614	0.00037308	0.00805801	0.00606171	11
BP	GO:0070252	actin-mediated cell contraction	9/396	104/18614	0.00037763	0.00808721	0.00608367	9
BP	GO:0097191	extrinsic apoptotic signaling pathway	14/396	227/18614	0.00037793	0.00808721	0.00608367	14
BP	GO:1905475	regulation of protein localization to membrane	12/396	175/18614	0.00037914	0.00808721	0.00608367	12
BP	GO:0034112	positive regulation of homotypic cell-cell adhesion	4/396	17/18614	0.00038546	0.00818834	0.00615975	4
BP	GO:0048762	mesenchymal cell differentiation	15/396	255/18614	0.00039104	0.00827267	0.00622319	15
BP	GO:0006836	neurotransmitter transport	13/396	202/18614	0.00040471	0.00852699	0.0064145	13
BP	GO:0001763	morphogenesis of a branching structure	13/396	203/18614	0.00042426	0.00890247	0.00669696	13
BP	GO:1901890	positive regulation of cell junction assembly	9/396	106/18614	0.00043489	0.00908864	0.006837	9
BP	GO:0070372	regulation of ERK1 and ERK2 cascade	17/396	315/18614	0.00044567	0.00927635	0.00697821	17
BP	GO:0048588	developmental cell growth	14/396	231/18614	0.0004504	0.00928497	0.0069847	14
BP	GO:0062044	negative regulation of cardiac epithelial to mesenchymal transition	2/396	2/18614	0.00045148	0.00928497	0.0069847	2
BP	GO:1905006	negative regulation of epithelial to mesenchymal transition involved in endocardial cushion formation	2/396	2/18614	0.00045148	0.00928497	0.0069847	2
BP	GO:0017145	stem cell division	5/396	31/18614	0.00045758	0.00937311	0.007051	5
BP	GO:0030593	neutrophil chemotaxis	9/396	107/18614	0.00046609	0.0095097	0.00715375	9
BP	GO:0002693	positive regulation of cellular extravasation	4/396	18/18614	0.00048732	0.00988473	0.00743587	4
BP	GO:0008625	extrinsic apoptotic signaling pathway via death domain receptors	8/396	86/18614	0.0004883	0.00988473	0.00743587	8
BP	GO:1904026	regulation of collagen fibril organization	3/396	8/18614	0.00049424	0.00992702	0.00746768	3
BP	GO:2000389	regulation of neutrophil extravasation	3/396	8/18614	0.00049424	0.00992702	0.00746768	3

BP	GO:1903037	regulation of leukocyte cell-cell adhesion	19/396	378/18614	0.00050662	0.01013627	0.00762509	19
BP	GO:0048678	response to axon injury	8/396	87/18614	0.00052795	0.01052234	0.00791552	8
BP	GO:0060485	mesenchyme development	17/396	320/18614	0.0005328	0.01052988	0.00792119	17
BP	GO:0060840	artery development	9/396	109/18614	0.00053402	0.01052988	0.00792119	9
BP	GO:1901654	response to ketone	13/396	208/18614	0.00053445	0.01052988	0.00792119	13
BP	GO:0022409	positive regulation of cell-cell adhesion	17/396	321/18614	0.00055188	0.01083192	0.0081484	17
BP	GO:0006909	phagocytosis	14/396	236/18614	0.00055738	0.01089838	0.0081984	14
BP	GO:0033273	response to vitamin	8/396	88/18614	0.00057017	0.01107908	0.00833433	8
BP	GO:0014812	muscle cell migration	9/396	110/18614	0.00057091	0.01107908	0.00833433	9
BP	GO:1901522	positive regulation of transcription from RNA polymerase II promoter involved in cellular response to chemical stimulus	4/396	19/18614	0.00060697	0.01173477	0.00882758	4
BP	GO:1905207	regulation of cardiocyte differentiation	5/396	33/18614	0.00061726	0.01188909	0.00894367	5
BP	GO:0001654	eye development	19/396	385/18614	0.00063259	0.01213922	0.00913183	19
BP	GO:1905954	positive regulation of lipid localization	9/396	112/18614	0.00065096	0.0124454	0.00936215	9
BP	GO:0030324	lung development	12/396	186/18614	0.00065494	0.01247533	0.00938467	12
BP	GO:0002274	myeloid leukocyte activation	14/396	240/18614	0.00065783	0.01248435	0.00939145	14
BP	GO:0006821	chloride transport	9/396	113/18614	0.00069429	0.0130979	0.00985301	9
BP	GO:0030100	regulation of endocytosis	13/396	214/18614	0.00069778	0.0130979	0.00985301	13
BP	GO:1901888	regulation of cell junction assembly	13/396	214/18614	0.00069778	0.0130979	0.00985301	13
BP	GO:1902624	positive regulation of neutrophil migration	5/396	34/18614	0.00071118	0.01330117	0.01000591	5
BP	GO:0032964	collagen biosynthetic process	6/396	51/18614	0.00071609	0.0133181	0.01001866	6
BP	GO:0001558	regulation of cell growth	20/396	420/18614	0.00071744	0.0133181	0.01001866	20
BP	GO:0061138	morphogenesis of a branching epithelium	12/396	188/18614	0.00071983	0.0133181	0.01001866	12
BP	GO:0002246	wound healing involved in inflammatory response	3/396	9/18614	0.00072967	0.01340417	0.0100834	3
BP	GO:0035635	entry of bacterium into host cell	3/396	9/18614	0.00072967	0.01340417	0.0100834	3
BP	GO:2000146	negative regulation of cell motility	19/396	390/18614	0.00073836	0.013497	0.01015323	19
BP	GO:0002526	acute inflammatory response	9/396	114/18614	0.00073996	0.013497	0.01015323	9
BP	GO:0055093	response to hyperoxia	4/396	20/18614	0.00074606	0.01356044	0.01020095	4
BP	GO:1901216	positive regulation of neuron death	8/396	92/18614	0.00076718	0.01389543	0.01045295	8

BP	GO:0030323	respiratory tube development	12/396	190/18614	0.00079001	0.01425883	0.01072632	12
BP	GO:0031103	axon regeneration	6/396	52/18614	0.00079519	0.01430232	0.01075904	6
BP	GO:0071346	cellular response to type II interferon	9/396	116/18614	0.00083863	0.01503126	0.01130739	9
BP	GO:0030856	regulation of epithelial cell differentiation	11/396	166/18614	0.00087354	0.0155786	0.01171914	11
BP	GO:0007409	axonogenesis	20/396	427/18614	0.00087897	0.0155786	0.01171914	20
BP	GO:0040013	negative regulation of locomotion	20/396	427/18614	0.00087897	0.0155786	0.01171914	20
BP	GO:0007269	neurotransmitter secretion	10/396	141/18614	0.00088426	0.0155786	0.01171914	10
BP	GO:0099643	signal release from synapse	10/396	141/18614	0.00088426	0.0155786	0.01171914	10
BP	GO:0050766	positive regulation of phagocytosis	7/396	73/18614	0.00091169	0.0160073	0.01204163	7
BP	GO:0001508	action potential	10/396	142/18614	0.00093365	0.01633723	0.01228982	10
BP	GO:0016049	cell growth	22/396	495/18614	0.00098372	0.01709755	0.01286177	22
BP	GO:0043410	positive regulation of MAPK cascade	22/396	495/18614	0.00098372	0.01709755	0.01286177	22
BP	GO:0045123	cellular extravasation	7/396	74/18614	0.00098887	0.0171293	0.01288566	7
BP	GO:0021782	glial cell development	9/396	119/18614	0.00100647	0.01737596	0.01307121	9
BP	GO:0002690	positive regulation of leukocyte chemotaxis	8/396	96/18614	0.00101557	0.01747454	0.01314537	8
BP	GO:0051962	positive regulation of nervous system development	15/396	280/18614	0.00102431	0.01747898	0.01314871	15
BP	GO:0001765	membrane raft assembly	3/396	10/18614	0.00102598	0.01747898	0.01314871	3
BP	GO:0098917	retrograde trans-synaptic signaling	3/396	10/18614	0.00102598	0.01747898	0.01314871	3
BP	GO:0002504	antigen processing and presentation of peptide or polysaccharide antigen via MHC class II	5/396	37/18614	0.00105742	0.0178718	0.01344421	5
BP	GO:0014059	regulation of dopamine secretion	5/396	37/18614	0.00105742	0.0178718	0.01344421	5
BP	GO:0050806	positive regulation of synaptic transmission	11/396	170/18614	0.00106152	0.0178718	0.01344421	11
BP	GO:0071222	cellular response to lipopolysaccharide	13/396	224/18614	0.00106289	0.0178718	0.01344421	13
BP	GO:0052372	modulation by symbiont of entry into host	6/396	55/18614	0.0010734	0.01798987	0.01353303	6
BP	GO:2000725	regulation of cardiac muscle cell differentiation	4/396	22/18614	0.00108919	0.01819544	0.01368767	4
BP	GO:1990138	neuron projection extension	11/396	172/18614	0.00116733	0.01943791	0.01462233	11
BP	GO:0001954	positive regulation of cell-matrix adhesion	6/396	56/18614	0.001181	0.01948843	0.01466033	6

BP	GO:0030336	negative regulation of cell migration	18/396	375/18614	0.00119356	0.01948843	0.01466033	18
BP	GO:0014046	dopamine secretion	5/396	38/18614	0.00119665	0.01948843	0.01466033	5
BP	GO:0040036	regulation of fibroblast growth factor receptor signaling pathway	5/396	38/18614	0.00119665	0.01948843	0.01466033	5
BP	GO:0002573	myeloid leukocyte differentiation	13/396	227/18614	0.00119942	0.01948843	0.01466033	13
BP	GO:0071675	regulation of mononuclear cell migration	9/396	122/18614	0.00120057	0.01948843	0.01466033	9
BP	GO:0090100	positive regulation of transmembrane receptor protein serine/threonine kinase signaling pathway	9/396	122/18614	0.00120057	0.01948843	0.01466033	9
BP	GO:0098661	inorganic anion transmembrane transport	9/396	122/18614	0.00120057	0.01948843	0.01466033	9
BP	GO:1902903	regulation of supramolecular fiber organization	18/396	376/18614	0.0012297	0.01989878	0.01496902	18
BP	GO:0045055	regulated exocytosis	13/396	228/18614	0.00124805	0.02013266	0.01514496	13
BP	GO:0030178	negative regulation of Wnt signaling pathway	11/396	174/18614	0.00128168	0.02061067	0.01550455	11
BP	GO:0050433	regulation of catecholamine secretion	6/396	57/18614	0.00129667	0.02063746	0.0155247	6
BP	GO:0051047	positive regulation of secretion	16/396	317/18614	0.00131731	0.02063746	0.0155247	16
BP	GO:0034446	substrate adhesion-dependent cell spreading	8/396	100/18614	0.00132446	0.02063746	0.0155247	8
BP	GO:0002538	arachidonic acid metabolite production involved in inflammatory response	2/396	3/18614	0.00133532	0.02063746	0.0155247	2
BP	GO:0002540	leukotriene production involved in inflammatory response	2/396	3/18614	0.00133532	0.02063746	0.0155247	2
BP	GO:0038195	urokinase plasminogen activator signaling pathway	2/396	3/18614	0.00133532	0.02063746	0.0155247	2
BP	GO:0048677	axon extension involved in regeneration	2/396	3/18614	0.00133532	0.02063746	0.0155247	2
BP	GO:0048682	sprouting of injured axon	2/396	3/18614	0.00133532	0.02063746	0.0155247	2
BP	GO:0099183	trans-synaptic signaling by BDNF, modulating synaptic transmission	2/396	3/18614	0.00133532	0.02063746	0.0155247	2
BP	GO:0150064	vertebrate eye-specific patterning	2/396	3/18614	0.00133532	0.02063746	0.0155247	2
BP	GO:1900159	positive regulation of bone mineralization	2/396	3/18614	0.00133532	0.02063746	0.0155247	2

		involved in bone maturation						
BP	GO:1903225	negative regulation of endodermal cell differentiation	2/396	3/18614	0.00133532	0.02063746	0.0155247	2
BP	GO:1905604	negative regulation of blood-brain barrier permeability	2/396	3/18614	0.00133532	0.02063746	0.0155247	2
BP	GO:0071674	mononuclear cell migration	12/396	202/18614	0.0013419	0.02067722	0.01555461	12
BP	GO:0003015	heart process	14/396	259/18614	0.00137011	0.02098674	0.01578745	14
BP	GO:0051402	neuron apoptotic process	14/396	259/18614	0.00137011	0.02098674	0.01578745	14
BP	GO:0031340	positive regulation of vesicle fusion	3/396	11/18614	0.00138855	0.02108144	0.01585869	3
BP	GO:0038063	collagen-activated tyrosine kinase receptor signaling pathway	3/396	11/18614	0.00138855	0.02108144	0.01585869	3
BP	GO:2000726	negative regulation of cardiac muscle cell differentiation	3/396	11/18614	0.00138855	0.02108144	0.01585869	3
BP	GO:0001764	neuron migration	11/396	176/18614	0.00140508	0.02120767	0.01595365	11
BP	GO:0010634	positive regulation of epithelial cell migration	11/396	176/18614	0.00140508	0.02120767	0.01595365	11
BP	GO:0060828	regulation of canonical Wnt signaling pathway	14/396	260/18614	0.0014208	0.02130368	0.01602587	14
BP	GO:0001952	regulation of cell-matrix adhesion	9/396	125/18614	0.00142382	0.02130368	0.01602587	9
BP	GO:0098656	monoatomic anion transmembrane transport	9/396	125/18614	0.00142382	0.02130368	0.01602587	9
BP	GO:0034765	regulation of monoatomic ion transmembrane transport	21/396	478/18614	0.00146771	0.02189686	0.01647209	21
BP	GO:0030316	osteoclast differentiation	8/396	102/18614	0.00150467	0.02238364	0.01683828	8
BP	GO:0032570	response to progesterone	5/396	40/18614	0.00151516	0.02247494	0.01690696	5
BP	GO:0050432	catecholamine secretion	6/396	59/18614	0.00155377	0.02298156	0.01728807	6
BP	GO:0045921	positive regulation of exocytosis	7/396	80/18614	0.00156603	0.02309677	0.01737474	7
BP	GO:0050807	regulation of synapse organization	13/396	234/18614	0.00157566	0.0231725	0.01743171	13
BP	GO:0015698	inorganic anion transport	11/396	179/18614	0.00160832	0.02358563	0.01774249	11
BP	GO:0060348	bone development	13/396	235/18614	0.00163663	0.02390716	0.01798436	13
BP	GO:0071216	cellular response to biotic stimulus	14/396	264/18614	0.00163951	0.02390716	0.01798436	14
BP	GO:0042417	dopamine metabolic process	5/396	41/18614	0.00169592	0.02452325	0.01844782	5
BP	GO:0002011	morphogenesis of an epithelial sheet	6/396	60/18614	0.00169601	0.02452325	0.01844782	6
BP	GO:0055008	cardiac muscle tissue morphogenesis	6/396	60/18614	0.00169601	0.02452325	0.01844782	6

BP	GO:0048771	tissue remodeling	11/396	181/18614	0.00175671	0.02533002	0.01905472	11
BP	GO:0071219	cellular response to molecule of bacterial origin	13/396	237/18614	0.00176445	0.02537079	0.01908539	13
BP	GO:2000027	regulation of animal organ morphogenesis	9/396	129/18614	0.00177222	0.0254117	0.01911616	9
BP	GO:0048167	regulation of synaptic plasticity	12/396	209/18614	0.00179006	0.02542386	0.01912531	12
BP	GO:0043567	regulation of insulin-like growth factor receptor signaling pathway	4/396	25/18614	0.00179115	0.02542386	0.01912531	4
BP	GO:0030099	myeloid cell differentiation	19/396	421/18614	0.00179667	0.02542386	0.01912531	19
BP	GO:0046928	regulation of neurotransmitter secretion	7/396	82/18614	0.00180765	0.02542386	0.01912531	7
BP	GO:0060041	retina development in camera-type eye	10/396	155/18614	0.0018088	0.02542386	0.01912531	10
BP	GO:0048103	somatic stem cell division	3/396	12/18614	0.00182232	0.02542386	0.01912531	3
BP	GO:0060394	negative regulation of pathway-restricted SMAD protein phosphorylation	3/396	12/18614	0.00182232	0.02542386	0.01912531	3
BP	GO:0097084	vascular associated smooth muscle cell development	3/396	12/18614	0.00182232	0.02542386	0.01912531	3
BP	GO:0099550	trans-synaptic signaling, modulating synaptic transmission	3/396	12/18614	0.00182232	0.02542386	0.01912531	3
BP	GO:1901731	positive regulation of platelet aggregation	3/396	12/18614	0.00182232	0.02542386	0.01912531	3
BP	GO:0022029	telencephalon cell migration	6/396	61/18614	0.00184792	0.02550521	0.01918651	6
BP	GO:0045599	negative regulation of fat cell differentiation	6/396	61/18614	0.00184792	0.02550521	0.01918651	6
BP	GO:0046456	icosanoid biosynthetic process	6/396	61/18614	0.00184792	0.02550521	0.01918651	6
BP	GO:1900024	regulation of substrate adhesion-dependent cell spreading	6/396	61/18614	0.00184792	0.02550521	0.01918651	6
BP	GO:0048638	regulation of developmental growth	16/396	328/18614	0.0018665	0.02566151	0.01930409	16
BP	GO:1903305	regulation of regulated secretory pathway	9/396	130/18614	0.00186918	0.02566151	0.01930409	9
BP	GO:0048284	organelle fusion	10/396	156/18614	0.00189703	0.02590603	0.01948803	10
BP	GO:1903531	negative regulation of secretion by cell	10/396	156/18614	0.00189703	0.02590603	0.01948803	10
BP	GO:0009266	response to temperature stimulus	11/396	183/18614	0.0019161	0.02609732	0.01963193	11

BP	GO:0060541	respiratory system development	12/396	211/18614	0.00193857	0.02633397	0.01980995	12
BP	GO:0030048	actin filament-based movement	9/396	131/18614	0.00197034	0.02664221	0.02004182	9
BP	GO:0050803	regulation of synapse structure or activity	13/396	240/18614	0.00197159	0.02664221	0.02004182	13
BP	GO:0048754	branching morphogenesis of an epithelial tube	10/396	157/18614	0.00198871	0.02680342	0.0201631	10
BP	GO:0050867	positive regulation of cell activation	18/396	394/18614	0.00205801	0.02766518	0.02081136	18
BP	GO:0019221	cytokine-mediated signaling pathway	21/396	492/18614	0.00208018	0.02776629	0.02088742	21
BP	GO:0035235	ionotropic glutamate receptor signaling pathway	4/396	26/18614	0.00208166	0.02776629	0.02088742	4
BP	GO:0099637	neurotransmitter receptor transport	4/396	26/18614	0.00208166	0.02776629	0.02088742	4
BP	GO:0045687	positive regulation of glial cell differentiation	5/396	43/18614	0.00210414	0.02785021	0.02095056	5
BP	GO:0051954	positive regulation of amine transport	5/396	43/18614	0.00210414	0.02785021	0.02095056	5
BP	GO:0071604	transforming growth factor beta production	5/396	43/18614	0.00210414	0.02785021	0.02095056	5
BP	GO:0032963	collagen metabolic process	8/396	108/18614	0.00216431	0.02857328	0.02149449	8
BP	GO:0032757	positive regulation of interleukin-8 production	6/396	63/18614	0.00218241	0.02873875	0.02161897	6
BP	GO:0060562	epithelial tube morphogenesis	16/396	334/18614	0.00223937	0.02941384	0.02212681	16
BP	GO:0007565	female pregnancy	11/396	187/18614	0.00227023	0.02959323	0.02226176	11
BP	GO:0071772	response to BMP	11/396	187/18614	0.00227023	0.02959323	0.02226176	11
BP	GO:0071773	cellular response to BMP stimulus	11/396	187/18614	0.00227023	0.02959323	0.02226176	11
BP	GO:0001667	ameboidal-type cell migration	21/396	496/18614	0.00229064	0.02978411	0.02240534	21
BP	GO:0033604	negative regulation of catecholamine secretion	3/396	13/18614	0.00233185	0.03009257	0.02263739	3
BP	GO:1903054	negative regulation of extracellular matrix organization	3/396	13/18614	0.00233185	0.03009257	0.02263739	3
BP	GO:2001046	positive regulation of integrin-mediated signaling pathway	3/396	13/18614	0.00233185	0.03009257	0.02263739	3
BP	GO:0021885	forebrain cell migration	6/396	64/18614	0.00236584	0.03037924	0.02285304	6
BP	GO:0050918	positive chemotaxis	6/396	64/18614	0.00236584	0.03037924	0.02285304	6
BP	GO:0015844	monoamine transport	7/396	86/18614	0.00237689	0.03044537	0.02290278	7
BP	GO:0051017	actin filament bundle assembly	10/396	161/18614	0.00239181	0.03056076	0.02298959	10

BP	GO:0036296	response to increased oxygen levels	4/396	27/18614	0.00240315	0.03062975	0.02304148	4
BP	GO:0042476	odontogenesis	9/396	135/18614	0.00241952	0.03076243	0.0231413	9
BP	GO:0043010	camera-type eye development	16/396	338/18614	0.00252095	0.03197332	0.0240522	16
BP	GO:0060047	heart contraction	13/396	247/18614	0.00253374	0.03205675	0.02411496	13
BP	GO:0031663	lipopolysaccharide-mediated signaling pathway	6/396	65/18614	0.00256063	0.03231777	0.02431132	6
BP	GO:0098815	modulation of excitatory postsynaptic potential	5/396	45/18614	0.00257971	0.03241263	0.02438267	5
BP	GO:0001550	ovarian cumulus expansion	2/396	4/18614	0.00263301	0.03241263	0.02438267	2
BP	GO:0014839	myoblast migration involved in skeletal muscle regeneration	2/396	4/18614	0.00263301	0.03241263	0.02438267	2
BP	GO:0098967	exocytic insertion of neurotransmitter receptor to postsynaptic membrane	2/396	4/18614	0.00263301	0.03241263	0.02438267	2
BP	GO:0099191	trans-synaptic signaling by BDNF	2/396	4/18614	0.00263301	0.03241263	0.02438267	2
BP	GO:0150062	complement-mediated synapse pruning	2/396	4/18614	0.00263301	0.03241263	0.02438267	2
BP	GO:1900084	regulation of peptidyl-tyrosine autophosphorylation	2/396	4/18614	0.00263301	0.03241263	0.02438267	2
BP	GO:1902723	negative regulation of skeletal muscle satellite cell proliferation	2/396	4/18614	0.00263301	0.03241263	0.02438267	2
BP	GO:1902725	negative regulation of satellite cell differentiation	2/396	4/18614	0.00263301	0.03241263	0.02438267	2
BP	GO:1905686	positive regulation of plasma membrane repair	2/396	4/18614	0.00263301	0.03241263	0.02438267	2
BP	GO:0043523	regulation of neuron apoptotic process	12/396	219/18614	0.00263721	0.03241263	0.02438267	12
BP	GO:0033002	muscle cell proliferation	13/396	249/18614	0.00271651	0.0333079	0.02505615	13
BP	GO:0002675	positive regulation of acute inflammatory response	4/396	28/18614	0.00275719	0.03364682	0.0253111	4
BP	GO:0031664	regulation of lipopolysaccharide-mediated signaling pathway	4/396	28/18614	0.00275719	0.03364682	0.0253111	4
BP	GO:0043123	positive regulation of I-kappaB kinase/NF-kappaB signaling	11/396	192/18614	0.00278568	0.03391431	0.02551233	11

BP	GO:0060048	cardiac muscle contraction	9/396	138/18614	0.00280693	0.03409269	0.02564652	9
BP	GO:0055010	ventricular cardiac muscle tissue morphogenesis	5/396	46/18614	0.00284465	0.03438894	0.02586937	5
BP	GO:2000300	regulation of synaptic vesicle exocytosis	5/396	46/18614	0.00284465	0.03438894	0.02586937	5
BP	GO:0061572	actin filament bundle organization	10/396	165/18614	0.00285805	0.03447024	0.02593053	10
BP	GO:0018158	protein oxidation	3/396	14/18614	0.0029213	0.03498786	0.02631992	3
BP	GO:0045956	positive regulation of calcium ion-dependent exocytosis	3/396	14/18614	0.0029213	0.03498786	0.02631992	3
BP	GO:0072672	neutrophil extravasation	3/396	14/18614	0.0029213	0.03498786	0.02631992	3
BP	GO:0034341	response to type II interferon	9/396	139/18614	0.00294644	0.03520719	0.02648491	9
BP	GO:0009620	response to fungus	6/396	67/18614	0.00298604	0.03559798	0.02677888	6
BP	GO:0051963	regulation of synapse assembly	8/396	114/18614	0.00303241	0.03606756	0.02713212	8
BP	GO:0043254	regulation of protein-containing complex assembly	18/396	409/18614	0.00306809	0.036408	0.02738822	18
BP	GO:0022406	membrane docking	7/396	90/18614	0.00307514	0.036408	0.02738822	7
BP	GO:0002696	positive regulation of leukocyte activation	17/396	377/18614	0.00309562	0.03648308	0.02744471	17
BP	GO:0050863	regulation of T cell activation	17/396	377/18614	0.00309562	0.03648308	0.02744471	17
BP	GO:0043277	apoptotic cell clearance	5/396	47/18614	0.00312871	0.03670546	0.02761199	5
BP	GO:1905314	semi-lunar valve development	5/396	47/18614	0.00312871	0.03670546	0.02761199	5
BP	GO:0031345	negative regulation of cell projection organization	11/396	195/18614	0.00313771	0.03672755	0.0276286	11
BP	GO:0006910	phagocytosis, recognition	4/396	29/18614	0.00314535	0.03673371	0.02763324	4
BP	GO:0048259	regulation of receptor-mediated endocytosis	8/396	115/18614	0.00320022	0.03720613	0.02798863	8
BP	GO:0051261	protein depolymerization	8/396	115/18614	0.00320022	0.03720613	0.02798863	8
BP	GO:0035914	skeletal muscle cell differentiation	6/396	68/18614	0.00321753	0.03732338	0.02807683	6
BP	GO:0070555	response to interleukin-1	9/396	141/18614	0.00324184	0.03752105	0.02822552	9
BP	GO:0090101	negative regulation of transmembrane receptor protein serine/threonine kinase signaling pathway	10/396	169/18614	0.00339421	0.0391967	0.02948605	10
BP	GO:0010721	negative regulation of cell development	14/396	286/18614	0.00340316	0.0392123	0.02949778	14
BP	GO:0006953	acute-phase response	5/396	48/18614	0.00343266	0.03946409	0.0296872	5
BP	GO:0014015	positive regulation of gliogenesis	6/396	69/18614	0.00346215	0.03971466	0.02987569	6

BP	GO:0010977	negative regulation of neuron projection development	9/396	143/18614	0.00356009	0.0407476	0.03065272	9
BP	GO:0001771	immunological synapse formation	3/396	15/18614	0.00359447	0.04086923	0.03074422	3
BP	GO:0032905	transforming growth factor beta1 production	3/396	15/18614	0.00359447	0.04086923	0.03074422	3
BP	GO:1905208	negative regulation of cardiocyte differentiation	3/396	15/18614	0.00359447	0.04086923	0.03074422	3
BP	GO:0048488	synaptic vesicle endocytosis	6/396	70/18614	0.00372032	0.04220728	0.03175078	6
BP	GO:1903532	positive regulation of secretion by cell	14/396	289/18614	0.00373344	0.04225477	0.03178651	14
BP	GO:0006911	phagocytosis, engulfment	5/396	49/18614	0.00375725	0.04225477	0.03178651	5
BP	GO:0033628	regulation of cell adhesion mediated by integrin	5/396	49/18614	0.00375725	0.04225477	0.03178651	5
BP	GO:0043114	regulation of vascular permeability	5/396	49/18614	0.00375725	0.04225477	0.03178651	5
BP	GO:0048705	skeletal system morphogenesis	12/396	229/18614	0.00378529	0.0424775	0.03195406	12
BP	GO:0031346	positive regulation of cell projection organization	16/396	353/18614	0.00385209	0.04313337	0.03244744	16
BP	GO:0030278	regulation of ossification	8/396	119/18614	0.00394474	0.04392546	0.03304329	8
BP	GO:0045446	endothelial cell differentiation	8/396	119/18614	0.00394474	0.04392546	0.03304329	8
BP	GO:0050678	regulation of epithelial cell proliferation	18/396	419/18614	0.00394836	0.04392546	0.03304329	18
BP	GO:0031214	biomineral tissue development	10/396	173/18614	0.00400742	0.04416902	0.03322652	10
BP	GO:0032970	regulation of actin filament-based process	17/396	387/18614	0.00402828	0.04416902	0.03322652	17
BP	GO:0019886	antigen processing and presentation of exogenous peptide antigen via MHC class II	4/396	31/18614	0.00403015	0.04416902	0.03322652	4
BP	GO:0033688	regulation of osteoblast proliferation	4/396	31/18614	0.00403015	0.04416902	0.03322652	4
BP	GO:0061311	cell surface receptor signaling pathway involved in heart development	4/396	31/18614	0.00403015	0.04416902	0.03322652	4
BP	GO:0086011	membrane repolarization during action potential	4/396	31/18614	0.00403015	0.04416902	0.03322652	4
BP	GO:2000463	positive regulation of excitatory postsynaptic potential	4/396	31/18614	0.00403015	0.04416902	0.03322652	4
BP	GO:0072073	kidney epithelium development	9/396	146/18614	0.00408288	0.04465221	0.03359	9

BP	GO:0010862	positive regulation of pathway-restricted SMAD protein phosphorylation	5/396	50/18614	0.00410326	0.04468569	0.03361518	5
BP	GO:0048009	insulin-like growth factor receptor signaling pathway	5/396	50/18614	0.00410326	0.04468569	0.03361518	5
BP	GO:0006906	vesicle fusion	8/396	120/18614	0.00415021	0.04510185	0.03392825	8
BP	GO:0050769	positive regulation of neurogenesis	12/396	232/18614	0.00419885	0.04541286	0.0341622	12
BP	GO:0006816	calcium ion transport	19/396	455/18614	0.00421698	0.04541286	0.0341622	19
BP	GO:0006820	monoatomic anion transport	9/396	147/18614	0.00426987	0.04541286	0.0341622	9
BP	GO:0140238	presynaptic endocytosis	6/396	72/18614	0.00427917	0.04541286	0.0341622	6
BP	GO:0009996	negative regulation of cell fate specification	2/396	5/18614	0.00432664	0.04541286	0.0341622	2
BP	GO:0014063	negative regulation of serotonin secretion	2/396	5/18614	0.00432664	0.04541286	0.0341622	2
BP	GO:0014859	negative regulation of skeletal muscle cell proliferation	2/396	5/18614	0.00432664	0.04541286	0.0341622	2
BP	GO:0019732	antifungal humoral response	2/396	5/18614	0.00432664	0.04541286	0.0341622	2
BP	GO:0022614	membrane to membrane docking	2/396	5/18614	0.00432664	0.04541286	0.0341622	2
BP	GO:0033602	negative regulation of dopamine secretion	2/396	5/18614	0.00432664	0.04541286	0.0341622	2
BP	GO:0035905	ascending aorta development	2/396	5/18614	0.00432664	0.04541286	0.0341622	2
BP	GO:0045113	regulation of integrin biosynthetic process	2/396	5/18614	0.00432664	0.04541286	0.0341622	2
BP	GO:0071455	cellular response to hyperoxia	2/396	5/18614	0.00432664	0.04541286	0.0341622	2
BP	GO:0098920	retrograde trans-synaptic signaling by lipid	2/396	5/18614	0.00432664	0.04541286	0.0341622	2
BP	GO:0098921	retrograde trans-synaptic signaling by endocannabinoid	2/396	5/18614	0.00432664	0.04541286	0.0341622	2
BP	GO:2001015	negative regulation of skeletal muscle cell differentiation	2/396	5/18614	0.00432664	0.04541286	0.0341622	2
BP	GO:0002399	MHC class II protein complex assembly	3/396	16/18614	0.00435478	0.04541286	0.0341622	3
BP	GO:0002503	peptide antigen assembly with MHC class II protein complex	3/396	16/18614	0.00435478	0.04541286	0.0341622	3
BP	GO:0007158	neuron cell-cell adhesion	3/396	16/18614	0.00435478	0.04541286	0.0341622	3
BP	GO:0038065	collagen-activated signaling pathway	3/396	16/18614	0.00435478	0.04541286	0.0341622	3

BP	GO:0098773	skin epidermis development	8/396	121/18614	0.00436383	0.04541553	0.03416422	8
BP	GO:0043281	regulation of cysteine-type endopeptidase activity involved in apoptotic process	11/396	204/18614	0.00441298	0.04583458	0.03447945	11
BP	GO:0015872	dopamine transport	5/396	51/18614	0.00447143	0.04616302	0.03472652	5
BP	GO:0043113	receptor clustering	5/396	51/18614	0.00447143	0.04616302	0.03472652	5
BP	GO:1902895	positive regulation of miRNA transcription	5/396	51/18614	0.00447143	0.04616302	0.03472652	5
BP	GO:2000116	regulation of cysteine-type endopeptidase activity	12/396	234/18614	0.00449416	0.04630513	0.03483343	12
BP	GO:0030509	BMP signaling pathway	10/396	176/18614	0.00452234	0.04650263	0.03498199	10
BP	GO:0022408	negative regulation of cell-cell adhesion	11/396	205/18614	0.00457695	0.04650685	0.03498517	11
BP	GO:0003170	heart valve development	6/396	73/18614	0.00458073	0.04650685	0.03498517	6
BP	GO:0042246	tissue regeneration	6/396	73/18614	0.00458073	0.04650685	0.03498517	6
BP	GO:0051937	catecholamine transport	6/396	73/18614	0.00458073	0.04650685	0.03498517	6
BP	GO:0061035	regulation of cartilage development	6/396	73/18614	0.00458073	0.04650685	0.03498517	6
BP	GO:0071260	cellular response to mechanical stimulus	6/396	73/18614	0.00458073	0.04650685	0.03498517	6
BP	GO:0090174	organelle membrane fusion	8/396	122/18614	0.00458582	0.04650685	0.03498517	8
BP	GO:0015850	organic hydroxy compound transport	14/396	296/18614	0.00460645	0.04662454	0.0350737	14
BP	GO:0070588	calcium ion transmembrane transport	16/396	360/18614	0.00464657	0.04681768	0.035219	16
BP	GO:0001657	ureteric bud development	7/396	97/18614	0.00466181	0.04681768	0.035219	7
BP	GO:0014909	smooth muscle cell migration	7/396	97/18614	0.00466181	0.04681768	0.035219	7
BP	GO:0016079	synaptic vesicle exocytosis	7/396	97/18614	0.00466181	0.04681768	0.035219	7
BP	GO:0002761	regulation of myeloid leukocyte differentiation	8/396	123/18614	0.00481637	0.04818238	0.0362456	8
BP	GO:0022612	gland morphogenesis	8/396	123/18614	0.00481637	0.04818238	0.0362456	8
BP	GO:0010543	regulation of platelet activation	5/396	52/18614	0.00486252	0.04854993	0.0365221	5
BP	GO:0044703	multi-organism reproductive process	11/396	207/18614	0.00491942	0.04885613	0.03675244	11
BP	GO:0051952	regulation of amine transport	7/396	98/18614	0.00493104	0.04885613	0.03675244	7
BP	GO:0072163	mesonephric epithelium development	7/396	98/18614	0.00493104	0.04885613	0.03675244	7
BP	GO:0072164	mesonephric tubule development	7/396	98/18614	0.00493104	0.04885613	0.03675244	7
BP	GO:0010876	lipid localization	20/396	497/18614	0.00506699	0.04993939	0.03756733	20

BP	GO:0061036	positive regulation of cartilage development	4/396	33/18614	0.0050694	0.04993939	0.03756733	4
BP	GO:0072677	eosinophil migration	4/396	33/18614	0.0050694	0.04993939	0.03756733	4
CC	GO:0062023	collagen-containing extracellular matrix	61/409	415/19518	6.62E-34	3.29E-31	2.13E-31	61
CC	GO:0005788	endoplasmic reticulum lumen	37/409	312/19518	1.26E-17	3.14E-15	2.03E-15	37
CC	GO:0005581	collagen trimer	20/409	85/19518	6.77E-16	1.12E-13	7.27E-14	20
CC	GO:0098644	complex of collagen trimers	10/409	21/19518	4.20E-12	5.23E-10	3.38E-10	10
CC	GO:0098636	protein complex involved in cell adhesion	13/409	57/19518	1.32E-10	1.31E-08	8.50E-09	13
CC	GO:0005604	basement membrane	15/409	84/19518	2.03E-10	1.66E-08	1.07E-08	15
CC	GO:0097060	synaptic membrane	31/409	390/19518	2.33E-10	1.66E-08	1.07E-08	31
CC	GO:0098637	protein complex involved in cell-matrix adhesion	8/409	17/19518	7.16E-10	4.46E-08	2.88E-08	8
CC	GO:0005583	fibrillar collagen trimer	7/409	12/19518	1.22E-09	6.07E-08	3.93E-08	7
CC	GO:0098643	banded collagen fibril	7/409	12/19518	1.22E-09	6.07E-08	3.93E-08	7
CC	GO:0034774	secretory granule lumen	25/409	322/19518	2.15E-08	9.72E-07	6.29E-07	25
CC	GO:0060205	cytoplasmic vesicle lumen	25/409	325/19518	2.58E-08	1.07E-06	6.92E-07	25
CC	GO:0031983	vesicle lumen	25/409	327/19518	2.91E-08	1.11E-06	7.21E-07	25
CC	GO:1904090	peptidase inhibitor complex	6/409	11/19518	3.45E-08	1.23E-06	7.94E-07	6
CC	GO:0098978	glutamatergic synapse	28/409	410/19518	4.82E-08	1.60E-06	1.04E-06	28
CC	GO:0031091	platelet alpha granule	13/409	91/19518	5.42E-08	1.69E-06	1.09E-06	13
CC	GO:0031093	platelet alpha granule lumen	11/409	67/19518	1.34E-07	3.93E-06	2.54E-06	11
CC	GO:0097179	protease inhibitor complex	5/409	8/19518	2.10E-07	5.80E-06	3.75E-06	5
CC	GO:0043025	neuronal cell body	30/409	500/19518	2.71E-07	7.12E-06	4.60E-06	30
CC	GO:0045211	postsynaptic membrane	21/409	277/19518	4.35E-07	1.08E-05	7.00E-06	21
CC	GO:0099572	postsynaptic specialization	23/409	364/19518	2.92E-06	6.92E-05	4.48E-05	23
CC	GO:1902710	GABA receptor complex	6/409	21/19518	3.39E-06	7.40E-05	4.79E-05	6
CC	GO:0005925	focal adhesion	25/409	422/19518	3.43E-06	7.40E-05	4.79E-05	25
CC	GO:0030427	site of polarized growth	15/409	172/19518	3.57E-06	7.40E-05	4.79E-05	15
CC	GO:0030667	secretory granule membrane	21/409	319/19518	4.18E-06	8.33E-05	5.39E-05	21
CC	GO:0045121	membrane raft	21/409	323/19518	5.08E-06	9.47E-05	6.13E-05	21
CC	GO:0030055	cell-substrate junction	25/409	432/19518	5.17E-06	9.47E-05	6.13E-05	25
CC	GO:0098857	membrane microdomain	21/409	324/19518	5.33E-06	9.47E-05	6.13E-05	21
CC	GO:0005602	complement component C1 complex	3/409	3/19518	9.14E-06	0.00015165	9.81E-05	3
CC	GO:0062167	complement component C1q complex	3/409	3/19518	9.14E-06	0.00015165	9.81E-05	3
CC	GO:0042734	presynaptic membrane	14/409	164/19518	9.62E-06	0.00015459	1.00E-04	14
CC	GO:0098982	GABA-ergic synapse	10/409	84/19518	1.01E-05	0.00015791	0.00010214	10
CC	GO:0072562	blood microparticle	13/409	144/19518	1.09E-05	0.0001649	0.00010665	13

CC	GO:0030426	growth cone	14/409	167/19518	1.18E-05	0.00017346	0.0001122	14
CC	GO:0150034	distal axon	18/409	277/19518	2.41E-05	0.00034299	0.00022185	18
CC	GO:0031252	cell leading edge	23/409	422/19518	3.18E-05	0.00043953	0.00028429	23
CC	GO:1902711	GABA-A receptor complex	5/409	19/19518	3.60E-05	0.00048422	0.00031319	5
CC	GO:0005912	adherens junction	14/409	185/19518	3.74E-05	0.00048951	0.00031661	14
CC	GO:0032279	asymmetric synapse	20/409	353/19518	6.09E-05	0.00076889	0.00049732	20
CC	GO:0098984	neuron to neuron synapse	21/409	382/19518	6.18E-05	0.00076889	0.00049732	21
CC	GO:0034702	ion channel complex	18/409	299/19518	6.54E-05	0.00079492	0.00051415	18
CC	GO:0005592	collagen type XI trimer	3/409	5/19518	8.85E-05	0.0010497	0.00067894	3
CC	GO:0014069	postsynaptic density	19/409	339/19518	0.00010675	0.00123629	0.00079963	19
CC	GO:0098685	Schaffer collateral - CA1 synapse	9/409	94/19518	0.0001577	0.00178486	0.00115444	9
CC	GO:0097180	serine protease inhibitor complex	3/409	6/19518	0.0001743	0.00192893	0.00124763	3
CC	GO:0032590	dendrite membrane	6/409	41/19518	0.00019726	0.0021355	0.00138124	6
CC	GO:0032589	neuron projection membrane	7/409	60/19518	0.00025005	0.00264946	0.00171366	7
CC	GO:0048786	presynaptic active zone	8/409	82/19518	0.0003198	0.00331788	0.002146	8
CC	GO:0043197	dendritic spine	12/409	175/19518	0.00033225	0.00337671	0.00218405	12
CC	GO:0044309	neuron spine	12/409	178/19518	0.0003878	0.0038344	0.00248008	12
CC	GO:0099634	postsynaptic specialization membrane	10/409	129/19518	0.00039268	0.0038344	0.00248008	10
CC	GO:0008305	integrin complex	5/409	31/19518	0.00042744	0.00386142	0.00249756	5
CC	GO:0031256	leading edge membrane	12/409	180/19518	0.00042904	0.00386142	0.00249756	12
CC	GO:0005584	collagen type I trimer	2/409	2/19518	0.00043806	0.00386142	0.00249756	2
CC	GO:1990665	AnxA2-p11 complex	2/409	2/19518	0.00043806	0.00386142	0.00249756	2
CC	GO:1990971	EMILIN complex	2/409	2/19518	0.00043806	0.00386142	0.00249756	2
CC	GO:0043204	perikaryon	11/409	156/19518	0.00046049	0.00386142	0.00249756	11
CC	GO:0036454	growth factor complex	3/409	8/19518	0.00047299	0.00386142	0.00249756	3
CC	GO:0098642	network-forming collagen trimer	3/409	8/19518	0.00047299	0.00386142	0.00249756	3
CC	GO:0098645	collagen network	3/409	8/19518	0.00047299	0.00386142	0.00249756	3
CC	GO:0098651	basement membrane collagen trimer	3/409	8/19518	0.00047299	0.00386142	0.00249756	3
CC	GO:0042383	sarcolemma	10/409	136/19518	0.00059657	0.00479183	0.00309934	10
CC	GO:0034707	chloride channel complex	6/409	55/19518	0.00099412	0.00785829	0.00508273	6
CC	GO:0030672	synaptic vesicle membrane	9/409	125/19518	0.00128563	0.00977825	0.00632455	9
CC	GO:0099501	exocytic vesicle membrane	9/409	125/19518	0.00128563	0.00977825	0.00632455	9
CC	GO:0099544	perisynaptic space	2/409	3/19518	0.00129591	0.00977825	0.00632455	2
CC	GO:1990351	transporter complex	19/409	418/19518	0.00140249	0.01042445	0.00674251	19
CC	GO:0008021	synaptic vesicle	12/409	210/19518	0.00164798	0.01198445	0.00775152	12
CC	GO:1902495	transmembrane transporter complex	18/409	392/19518	0.0016605	0.01198445	0.00775152	18
CC	GO:0042581	specific granule	10/409	160/19518	0.0020529	0.01460492	0.00944643	10
CC	GO:0070820	tertiary granule	10/409	164/19518	0.00245956	0.01674757	0.01083229	10
CC	GO:0005588	collagen type V trimer	2/409	4/19518	0.00255585	0.01674757	0.01083229	2

CC	GO:0042567	insulin-like growth factor ternary complex	2/409	4/19518	0.00255585	0.01674757	0.01083229	2
CC	GO:0072557	IPAF inflammasome complex	2/409	4/19518	0.00255585	0.01674757	0.01083229	2
CC	GO:0090733	tenascin complex	2/409	4/19518	0.00255585	0.01674757	0.01083229	2
CC	GO:0097169	AIM2 inflammasome complex	2/409	4/19518	0.00255585	0.01674757	0.01083229	2
CC	GO:0030139	endocytic vesicle	16/409	347/19518	0.00282815	0.0178702	0.0115584	16
CC	GO:0016528	sarcoplasm	7/409	90/19518	0.00283221	0.0178702	0.0115584	7
CC	GO:0045177	apical part of cell	19/409	446/19518	0.00290235	0.0178702	0.0115584	19
CC	GO:0030666	endocytic vesicle membrane	11/409	196/19518	0.00291665	0.0178702	0.0115584	11
CC	GO:0070382	exocytic vesicle	12/409	225/19518	0.00291774	0.0178702	0.0115584	12
CC	GO:0031253	cell projection membrane	16/409	349/19518	0.00299212	0.0178702	0.0115584	16
CC	GO:0035578	azurophil granule lumen	7/409	91/19518	0.00301425	0.0178702	0.0115584	7
CC	GO:0035579	specific granule membrane	7/409	91/19518	0.00301425	0.0178702	0.0115584	7
CC	GO:0014704	intercalated disc	5/409	50/19518	0.0038514	0.02225497	0.01439446	5
CC	GO:0030133	transport vesicle	18/409	425/19518	0.00393124	0.02225497	0.01439446	18
CC	GO:0044291	cell-cell contact zone	6/409	72/19518	0.00398036	0.02225497	0.01439446	6
CC	GO:0030658	transport vesicle membrane	12/409	234/19518	0.00400051	0.02225497	0.01439446	12
CC	GO:0005775	vacuolar lumen	10/409	176/19518	0.00407938	0.02225497	0.01439446	10
CC	GO:0016942	insulin-like growth factor binding protein complex	2/409	5/19518	0.00420074	0.02225497	0.01439446	2
CC	GO:0046696	lipopolysaccharide receptor complex	2/409	5/19518	0.00420074	0.02225497	0.01439446	2
CC	GO:0071953	elastic fiber	2/409	5/19518	0.00420074	0.02225497	0.01439446	2
CC	GO:0098843	postsynaptic endocytic zone	2/409	5/19518	0.00420074	0.02225497	0.01439446	2
CC	GO:0098966	perisynaptic extracellular matrix	2/409	5/19518	0.00420074	0.02225497	0.01439446	2
CC	GO:0042613	MHC class II protein complex	3/409	17/19518	0.00499201	0.02616864	0.01692581	3
CC	GO:0005891	voltage-gated calcium channel complex	5/409	55/19518	0.00580858	0.02967025	0.01919065	5
CC	GO:0032432	actin filament bundle	6/409	78/19518	0.0058951	0.02967025	0.01919065	6
CC	GO:0031527	filopodium membrane	3/409	18/19518	0.0058983	0.02967025	0.01919065	3
CC	GO:1905286	serine-type peptidase complex	3/409	18/19518	0.0058983	0.02967025	0.01919065	3
CC	GO:0005587	collagen type IV trimer	2/409	6/19518	0.00621396	0.03063911	0.01981731	2
CC	GO:0099535	synapse-associated extracellular matrix	2/409	6/19518	0.00621396	0.03063911	0.01981731	2
CC	GO:0098802	plasma membrane signaling receptor complex	14/409	319/19518	0.00769652	0.03757715	0.02430481	14
CC	GO:0097386	glial cell projection	4/409	38/19518	0.00799197	0.0386408	0.02499278	4
CC	GO:1990635	proximal dendrite	2/409	7/19518	0.00857943	0.04108225	0.0265719	2
CC	GO:0043083	synaptic cleft	3/409	21/19518	0.00917792	0.04331699	0.02801733	3

CC	GO:0042470	melanosome	7/409	112/19518	0.00930706	0.04331699	0.02801733	7
CC	GO:0048770	pigment granule	7/409	112/19518	0.00930706	0.04331699	0.02801733	7
CC	GO:0009925	basal plasma membrane	12/409	266/19518	0.01068511	0.04881876	0.03157587	12
CC	GO:0016323	basolateral plasma membrane	11/409	234/19518	0.01068523	0.04881876	0.03157587	11
CC	GO:0060076	excitatory synapse	5/409	64/19518	0.01093254	0.04928266	0.03187591	5
CC	GO:0048788	cytoskeleton of presynaptic active zone	2/409	8/19518	0.01128157	0.04928266	0.03187591	2
CC	GO:0061200	clathrin-sculpted gamma-aminobutyric acid transport vesicle	2/409	8/19518	0.01128157	0.04928266	0.03187591	2
CC	GO:0061202	clathrin-sculpted gamma-aminobutyric acid transport vesicle membrane	2/409	8/19518	0.01128157	0.04928266	0.03187591	2
CC	GO:0072559	NLRP3 inflammasome complex	2/409	8/19518	0.01128157	0.04928266	0.03187591	2
MF	GO:0005201	extracellular matrix structural constituent	28/402	167/18369	4.19E-17	3.54E-14	2.67E-14	28
MF	GO:0030020	extracellular matrix structural constituent conferring tensile strength	11/402	41/18369	8.43E-10	3.57E-07	2.69E-07	11
MF	GO:0050840	extracellular matrix binding	12/402	55/18369	1.92E-09	5.43E-07	4.09E-07	12
MF	GO:0005178	integrin binding	18/402	151/18369	5.75E-09	1.22E-06	9.17E-07	18
MF	GO:0002020	protease binding	17/402	138/18369	9.26E-09	1.57E-06	1.18E-06	17
MF	GO:0005518	collagen binding	11/402	66/18369	1.75E-07	2.47E-05	1.86E-05	11
MF	GO:0019838	growth factor binding	15/402	132/18369	2.04E-07	2.47E-05	1.86E-05	15
MF	GO:0098641	cadherin binding involved in cell-cell adhesion	6/402	18/18369	1.57E-06	0.00016627	0.00012537	6
MF	GO:0048407	platelet-derived growth factor binding	5/402	11/18369	2.03E-06	0.00019079	0.00014386	5
MF	GO:0061134	peptidase regulator activity	18/402	230/18369	3.36E-06	0.00026871	0.00020261	18
MF	GO:0005539	glycosaminoglycan binding	18/402	232/18369	3.79E-06	0.00026871	0.00020261	18
MF	GO:0001968	fibronectin binding	7/402	31/18369	3.81E-06	0.00026871	0.00020261	7
MF	GO:0004857	enzyme inhibitor activity	23/402	359/18369	4.68E-06	0.00030453	0.00022962	23
MF	GO:0022851	GABA-gated chloride ion channel activity	5/402	13/18369	5.45E-06	0.0003295	0.00024845	5
MF	GO:0016917	GABA receptor activity	6/402	22/18369	5.87E-06	0.00033091	0.00024951	6
MF	GO:0004866	endopeptidase inhibitor activity	15/402	173/18369	6.41E-06	0.00033894	0.00025557	15
MF	GO:0030414	peptidase inhibitor activity	15/402	180/18369	1.04E-05	0.00051617	0.0003892	15
MF	GO:0061135	endopeptidase regulator activity	15/402	187/18369	1.64E-05	0.00076966	0.00058033	15

MF	GO:0043236	laminin binding	6/402	28/18369	2.65E-05	0.00112059	0.00084494	6
MF	GO:0022824	transmitter-gated monoatomic ion channel activity	8/402	56/18369	2.78E-05	0.00112059	0.00084494	8
MF	GO:0022835	transmitter-gated channel activity	8/402	56/18369	2.78E-05	0.00112059	0.00084494	8
MF	GO:0004890	GABA-A receptor activity	5/402	19/18369	4.42E-05	0.00161889	0.00122066	5
MF	GO:0099095	ligand-gated monoatomic anion channel activity	5/402	19/18369	4.42E-05	0.00161889	0.00122066	5
MF	GO:0022836	gated channel activity	19/402	308/18369	5.25E-05	0.00161889	0.00122066	19
MF	GO:0022839	monoatomic ion gated channel activity	19/402	308/18369	5.25E-05	0.00161889	0.00122066	19
MF	GO:0030594	neurotransmitter receptor activity	10/402	98/18369	5.69E-05	0.00161889	0.00122066	10
MF	GO:0015267	channel activity	25/402	478/18369	5.74E-05	0.00161889	0.00122066	25
MF	GO:0001786	phosphatidylserine binding	8/402	62/18369	5.91E-05	0.00161889	0.00122066	8
MF	GO:0048306	calcium-dependent protein binding	8/402	62/18369	5.91E-05	0.00161889	0.00122066	8
MF	GO:0098631	cell adhesion mediator activity	8/402	62/18369	5.91E-05	0.00161889	0.00122066	8
MF	GO:0022803	passive transmembrane transporter activity	25/402	479/18369	5.93E-05	0.00161889	0.00122066	25
MF	GO:0008201	heparin binding	13/402	168/18369	8.62E-05	0.00222493	0.00167762	13
MF	GO:0005216	monoatomic ion channel activity	23/402	432/18369	8.68E-05	0.00222493	0.00167762	23
MF	GO:0004720	protein-lysine 6-oxidase activity	3/402	5/18369	0.00010069	0.0024338	0.00183512	3
MF	GO:0019834	phospholipase A2 inhibitor activity	3/402	5/18369	0.00010069	0.0024338	0.00183512	3
MF	GO:0005230	extracellular ligand-gated monoatomic ion channel activity	8/402	67/18369	0.00010359	0.0024344	0.00183557	8
MF	GO:0098632	cell-cell adhesion mediator activity	7/402	51/18369	0.00011516	0.0025655	0.00193442	7
MF	GO:0030246	carbohydrate binding	17/402	273/18369	0.00011524	0.0025655	0.00193442	17
MF	GO:0044325	transmembrane transporter binding	11/402	129/18369	0.00012698	0.00275445	0.00207689	11
MF	GO:0004867	serine-type endopeptidase inhibitor activity	9/402	92/18369	0.00018429	0.00389769	0.00293891	9
MF	GO:0005237	inhibitory extracellular ligand-gated monoatomic ion channel activity	4/402	15/18369	0.00025473	0.00525613	0.00396319	4
MF	GO:0016641	oxidoreductase activity, acting on the CH-NH2 group of donors, oxygen as acceptor	4/402	16/18369	0.0003338	0.00672362	0.00506969	4

MF	GO:0005546	phosphatidylinositol-4,5-bisphosphate binding	8/402	82/18369	0.00042587	0.00824849	0.00621947	8
MF	GO:0030169	low-density lipoprotein particle binding	4/402	17/18369	0.000429	0.00824849	0.00621947	4
MF	GO:0001872	(1->3)-beta-D-glucan binding	2/402	2/18369	0.00047777	0.00898216	0.00677266	2
MF	GO:0031995	insulin-like growth factor II binding	3/402	8/18369	0.00053683	0.00977917	0.00737362	3
MF	GO:0072341	modified amino acid binding	8/402	85/18369	0.00054329	0.00977917	0.00737362	8
MF	GO:0015108	chloride transmembrane transporter activity	9/402	107/18369	0.00057081	0.01006057	0.0075858	9
MF	GO:0019955	cytokine binding	10/402	130/18369	0.00058323	0.01006964	0.00759263	10
MF	GO:0003779	actin binding	21/402	434/18369	0.00062244	0.01053175	0.00794107	21
MF	GO:0008083	growth factor activity	11/402	160/18369	0.00081117	0.01325519	0.00999458	11
MF	GO:0016638	oxidoreductase activity, acting on the CH-NH2 group of donors	4/402	20/18369	0.00082912	0.01325519	0.00999458	4
MF	GO:0005543	phospholipid binding	22/402	475/18369	0.00083041	0.01325519	0.00999458	22
MF	GO:0005544	calcium-dependent phospholipid binding	6/402	52/18369	0.00092054	0.01442183	0.01087424	6
MF	GO:0099186	structural constituent of postsynapse	4/402	21/18369	0.00100665	0.01548407	0.01167518	4
MF	GO:0043394	proteoglycan binding	5/402	36/18369	0.00105564	0.01594777	0.01202482	5
MF	GO:0015103	inorganic anion transmembrane transporter activity	10/402	141/18369	0.00109406	0.01596482	0.01203767	10
MF	GO:0005031	tumor necrosis factor receptor activity	3/402	10/18369	0.00111339	0.01596482	0.01203767	3
MF	GO:0030023	extracellular matrix constituent conferring elasticity	3/402	10/18369	0.00111339	0.01596482	0.01203767	3
MF	GO:0004252	serine-type endopeptidase activity	11/402	170/18369	0.00133025	0.01875646	0.01414261	11
MF	GO:0034714	type III transforming growth factor beta receptor binding	2/402	3/18369	0.00141251	0.01958994	0.01477107	2
MF	GO:0046332	SMAD binding	7/402	77/18369	0.00147145	0.0196031	0.01478099	7
MF	GO:0048018	receptor ligand activity	22/402	497/18369	0.00148308	0.0196031	0.01478099	22
MF	GO:0008503	benzodiazepine receptor activity	3/402	11/18369	0.00150615	0.0196031	0.01478099	3
MF	GO:0046790	virion binding	3/402	11/18369	0.00150615	0.0196031	0.01478099	3
MF	GO:0008509	monoatomic anion transmembrane transporter activity	9/402	123/18369	0.00154418	0.01979363	0.01492465	9
MF	GO:0005540	hyaluronic acid binding	4/402	24/18369	0.00169704	0.02142831	0.01615722	4
MF	GO:0004859	phospholipase inhibitor activity	3/402	12/18369	0.00197576	0.02422452	0.01826559	3

MF	GO:0097493	structural molecule activity conferring elasticity	3/402	12/18369	0.00197576	0.02422452	0.01826559	3
MF	GO:0098918	structural constituent of synapse	4/402	26/18369	0.00230674	0.02758413	0.02079878	4
MF	GO:0005507	copper ion binding	6/402	62/18369	0.00231498	0.02758413	0.02079878	6
MF	GO:1902936	phosphatidylinositol bisphosphate binding	8/402	107/18369	0.00242997	0.02855218	0.02152871	8
MF	GO:0005035	death receptor activity	3/402	13/18369	0.00252704	0.02889024	0.02178361	3
MF	GO:0031994	insulin-like growth factor I binding	3/402	13/18369	0.00252704	0.02889024	0.02178361	3
MF	GO:0001540	amyloid-beta binding	7/402	85/18369	0.00260297	0.02936156	0.02213899	7
MF	GO:0002054	nucleobase binding	2/402	4/18369	0.00278409	0.02981438	0.02248042	2
MF	GO:0004522	ribonuclease A activity	2/402	4/18369	0.00278409	0.02981438	0.02248042	2
MF	GO:0004971	AMPA glutamate receptor activity	2/402	4/18369	0.00278409	0.02981438	0.02248042	2
MF	GO:1904399	heparan sulfate binding	2/402	4/18369	0.00278409	0.02981438	0.02248042	2
MF	GO:0035254	glutamate receptor binding	5/402	45/18369	0.00291342	0.03080939	0.02323067	5
MF	GO:0005253	monoatomic anion channel activity	7/402	87/18369	0.00296934	0.03081311	0.02323348	7
MF	GO:0019208	phosphatase regulator activity	8/402	111/18369	0.00305328	0.03081311	0.02323348	8
MF	GO:0005244	voltage-gated monoatomic ion channel activity	11/402	189/18369	0.00305946	0.03081311	0.02323348	11
MF	GO:0022832	voltage-gated channel activity	11/402	189/18369	0.00305946	0.03081311	0.02323348	11
MF	GO:0044548	S100 protein binding	3/402	14/18369	0.0031644	0.03133698	0.02362848	3
MF	GO:0008236	serine-type peptidase activity	11/402	190/18369	0.00318556	0.03133698	0.02362848	11
MF	GO:0015276	ligand-gated monoatomic ion channel activity	9/402	140/18369	0.0037208	0.03508407	0.02645384	9
MF	GO:0022834	ligand-gated channel activity	9/402	140/18369	0.0037208	0.03508407	0.02645384	9
MF	GO:0022843	voltage-gated monoatomic cation channel activity	9/402	140/18369	0.0037208	0.03508407	0.02645384	9
MF	GO:0017171	serine hydrolase activity	11/402	194/18369	0.00373235	0.03508407	0.02645384	11
MF	GO:0005044	scavenger receptor activity	5/402	48/18369	0.00387099	0.03552782	0.02678843	5
MF	GO:0140375	immune receptor activity	9/402	141/18369	0.00389989	0.03552782	0.02678843	9
MF	GO:0071813	lipoprotein particle binding	4/402	30/18369	0.00394754	0.03552782	0.02678843	4
MF	GO:0071814	protein-lipid complex binding	4/402	30/18369	0.00394754	0.03552782	0.02678843	4
MF	GO:0015085	calcium ion transmembrane transporter activity	9/402	142/18369	0.00408567	0.03638392	0.02743394	9

MF	GO:0001847	opsonin receptor activity	2/402	5/18369	0.00457302	0.03868771	0.02917103	2
MF	GO:0005314	high-affinity L-glutamate transmembrane transporter activity	2/402	5/18369	0.00457302	0.03868771	0.02917103	2
MF	GO:0015501	glutamate:sodium symporter activity	2/402	5/18369	0.00457302	0.03868771	0.02917103	2
MF	GO:0019863	IgE binding	2/402	5/18369	0.00457302	0.03868771	0.02917103	2
MF	GO:0045545	syndecan binding	2/402	5/18369	0.00457302	0.03868771	0.02917103	2
MF	GO:0055102	lipase inhibitor activity	3/402	16/18369	0.00471289	0.03947633	0.02976565	3
MF	GO:0005245	voltage-gated calcium channel activity	5/402	52/18369	0.00547271	0.04539131	0.03422562	5
MF	GO:0005254	chloride channel activity	6/402	74/18369	0.0056072	0.04605528	0.03472627	6
MF	GO:0005125	cytokine activity	12/402	235/18369	0.00578679	0.04707329	0.03549386	12

Table S16 KEGG pathways based on DEGs between high and low SEPN1 expression groups in TCGA-LGG.

ID	Description	GeneRatio	BgRatio	P-value	P-adjust	Q-value	Count
hsa05150	Staphylococcus aureus infection	30/383	100/8842	7.54E-18	2.06E-15	1.66E-15	30
hsa05140	Leishmaniasis	24/383	77/8842	6.21E-15	8.48E-13	6.83E-13	24
hsa05310	Asthma	14/383	31/8842	8.77E-12	7.98E-10	6.43E-10	14
hsa04145	Phagosome	29/383	157/8842	2.17E-11	1.48E-09	1.20E-09	29
hsa05152	Tuberculosis	30/383	180/8842	1.41E-10	6.77E-09	5.46E-09	30
hsa05321	Inflammatory bowel disease	18/383	65/8842	1.49E-10	6.77E-09	5.46E-09	18
hsa04080	Neuroactive ligand-receptor interaction	44/383	368/8842	5.89E-10	2.30E-08	1.85E-08	44
hsa05033	Nicotine addiction	14/383	41/8842	7.84E-10	2.67E-08	2.15E-08	14
hsa04640	Hematopoietic cell lineage	21/383	99/8842	9.79E-10	2.97E-08	2.39E-08	21
hsa04672	Intestinal immune network for IgA production	15/383	49/8842	1.11E-09	3.02E-08	2.43E-08	15
hsa04940	Type I diabetes mellitus	14/383	43/8842	1.61E-09	3.99E-08	3.22E-08	14
hsa05330	Allograft rejection	13/383	38/8842	3.12E-09	7.10E-08	5.72E-08	13
hsa05332	Graft-versus-host disease	13/383	44/8842	2.36E-08	4.95E-07	3.99E-07	13
hsa05323	Rheumatoid arthritis	18/383	94/8842	8.45E-08	1.65E-06	1.33E-06	18
hsa05416	Viral myocarditis	15/383	69/8842	1.80E-07	3.27E-06	2.64E-06	15
hsa05145	Toxoplasmosis	19/383	111/8842	2.38E-07	4.07E-06	3.28E-06	19
hsa05320	Autoimmune thyroid disease	13/383	53/8842	2.68E-07	4.30E-06	3.47E-06	13
hsa05322	Systemic lupus erythematosus	21/383	139/8842	4.92E-07	7.47E-06	6.02E-06	21
hsa04610	Complement and coagulation cascades	16/383	88/8842	9.31E-07	1.34E-05	1.08E-05	16
hsa04514	Cell adhesion molecules	22/383	157/8842	9.89E-07	1.35E-05	1.09E-05	22
hsa04380	Osteoclast differentiation	19/383	142/8842	1.08E-05	0.00014083	0.00011349	19
hsa04060	Cytokine-cytokine receptor interaction	30/383	298/8842	1.33E-05	0.00016541	0.0001333	30
hsa04612	Antigen processing and presentation	13/383	80/8842	3.46E-05	0.00041098	0.00033119	13
hsa04613	Neutrophil extracellular trap formation	21/383	192/8842	8.27E-05	0.00094027	0.00075773	21
hsa04512	ECM-receptor interaction	13/383	89/8842	0.00010849	0.00118467	0.00095468	13
hsa04658	Th1 and Th2 cell differentiation	13/383	92/8842	0.00015315	0.00160813	0.00129593	13
hsa04974	Protein digestion and absorption	14/383	105/8842	0.00016244	0.00164244	0.00132357	14
hsa04659	Th17 cell differentiation	14/383	108/8842	0.00021987	0.00214372	0.00172754	14
hsa05166	Human T-cell leukemia virus 1 infection	22/383	223/8842	0.00025609	0.00241074	0.00194272	22
hsa04061	Viral protein interaction with cytokine and cytokine receptor	13/383	100/8842	0.00035652	0.00324429	0.00261445	13
hsa05169	Epstein-Barr virus infection	19/383	203/8842	0.00125994	0.01109559	0.0089415	19
hsa04062	Chemokine signaling pathway	18/383	193/8842	0.00174661	0.01490075	0.01200793	18
hsa05164	Influenza A	16/383	172/8842	0.00317086	0.02623165	0.02113906	16
hsa04020	Calcium signaling pathway	21/383	254/8842	0.00332647	0.0267096	0.02152422	21
hsa04726	Serotonergic synapse	12/383	115/8842	0.00402988	0.03143303	0.02533065	12
hsa05146	Amoebiasis	11/383	103/8842	0.00485676	0.03683045	0.02968022	11
hsa05144	Malaria	7/383	50/8842	0.00540995	0.03991668	0.03216729	7
hsa04662	B cell receptor signaling pathway	10/383	91/8842	0.00581204	0.04068426	0.03278585	10
hsa05032	Morphine addiction	10/383	91/8842	0.00581204	0.04068426	0.03278585	10
hsa05133	Pertussis	9/383	78/8842	0.00633526	0.04323813	0.03484391	9
hsa04350	TGF-beta signaling pathway	11/383	108/8842	0.00693432	0.04617245	0.03720857	11
hsa05205	Proteoglycans in cancer	17/383	204/8842	0.00729026	0.04738667	0.03818706	17

hsa04810	Regulation of actin cytoskeleton	18/383	230/8842	0.01093215	0.06940644	0.05593193	18
hsa04933	AGE-RAGE signaling pathway in diabetic complications	10/383	101/8842	0.01188019	0.07371118	0.05940095	10
hsa04727	GABAergic synapse	9/383	89/8842	0.01459295	0.08853054	0.0713433	9
hsa04024	cAMP signaling pathway	17/383	226/8842	0.01881433	0.11165894	0.08998157	17
hsa04620	Toll-like receptor signaling pathway	10/383	109/8842	0.01947687	0.11313159	0.09116831	10
hsa04721	Synaptic vesicle cycle	8/383	79/8842	0.02056486	0.11523886	0.09286648	8
hsa05143	African trypanosomiasis	5/383	37/8842	0.0206839	0.11523886	0.09286648	5
hsa04666	Fc gamma R-mediated phagocytosis	9/383	98/8842	0.02579553	0.13808197	0.11127485	9
hsa04925	Aldosterone synthesis and secretion	9/383	98/8842	0.02579553	0.13808197	0.11127485	9
hsa05410	Hypertrophic cardiomyopathy	9/383	99/8842	0.02733579	0.1435129	0.11565142	9
hsa04723	Retrograde endocannabinoid signaling	12/383	149/8842	0.0280862	0.14467044	0.11658424	12
hsa04670	Leukocyte transendothelial migration	10/383	116/8842	0.02862346	0.14470749	0.1166141	10
hsa04510	Focal adhesion	15/383	203/8842	0.03026344	0.15021672	0.12105377	15
hsa04742	Taste transduction	8/383	86/8842	0.03230016	0.15470076	0.12466728	8
hsa04911	Insulin secretion	8/383	86/8842	0.03230016	0.15470076	0.12466728	8
hsa05134	Legionellosis	6/383	56/8842	0.03326814	0.15658969	0.12618949	6
hsa04216	Ferroptosis	5/383	42/8842	0.03390053	0.15686179	0.12640877	5
hsa04261	Adrenergic signaling in cardiomyocytes	12/383	154/8842	0.03502295	0.1593544	0.12841747	12
hsa05414	Dilated cardiomyopathy	9/383	105/8842	0.03795485	0.1678187	0.13523851	9
hsa04148	Efferocytosis	12/383	156/8842	0.03811267	0.1678187	0.13523851	12
hsa05202	Transcriptional misregulation in cancer	14/383	193/8842	0.04058385	0.17586334	0.14172137	14
hsa04110	Cell cycle	12/383	158/8842	0.04138997	0.17655409	0.14227802	12

Table S17 KEGG pathways based on DEGs between high and low SEPN1 expression groups in CGGA-693.

ID	Description	GeneRatio	BgRatio	P-value	P-adjust	Q-value	Count
hsa05205	Proteoglycans in cancer	97/2147	203/8773	2.69E-13	9.15E-11	6.32E-11	97
hsa04110	Cell cycle	75/2147	157/8773	1.43E-10	2.43E-08	1.68E-08	75
hsa05145	Toxoplasmosis	57/2147	111/8773	7.61E-10	8.62E-08	5.95E-08	57
hsa04510	Focal adhesion	87/2147	202/8773	3.58E-09	3.04E-07	2.10E-07	87
hsa05164	Influenza A	74/2147	171/8773	4.16E-08	2.83E-06	1.95E-06	74
hsa05132	Salmonella infection	98/2147	247/8773	6.07E-08	3.44E-06	2.37E-06	98
hsa05161	Hepatitis B	70/2147	162/8773	1.04E-07	5.03E-06	3.48E-06	70
hsa05140	Leishmaniasis	40/2147	77/8773	1.72E-07	7.32E-06	5.05E-06	40
hsa05152	Tuberculosis	75/2147	180/8773	2.22E-07	8.39E-06	5.79E-06	75
hsa04933	AGE-RAGE signaling pathway in diabetic complications	48/2147	100/8773	2.47E-07	8.39E-06	5.79E-06	48
hsa05169	Epstein-Barr virus infection	81/2147	202/8773	5.09E-07	1.57E-05	1.09E-05	81
hsa05166	Human T-cell leukemia virus 1 infection	87/2147	222/8773	6.29E-07	1.78E-05	1.23E-05	87
hsa05130	Pathogenic Escherichia coli infection	79/2147	198/8773	8.90E-07	2.33E-05	1.61E-05	79
hsa04218	Cellular senescence	65/2147	156/8773	1.41E-06	3.43E-05	2.37E-05	65
hsa04145	Phagosome	63/2147	152/8773	2.53E-06	5.74E-05	3.96E-05	63
hsa05167	Kaposi sarcoma-associated herpesvirus infection	76/2147	194/8773	3.25E-06	6.90E-05	4.76E-05	76
hsa04520	Adherens junction	43/2147	93/8773	3.60E-06	7.20E-05	4.97E-05	43
hsa04380	Osteoclast differentiation	58/2147	141/8773	8.29E-06	0.00015257	0.00010533	58
hsa05162	Measles	57/2147	138/8773	8.53E-06	0.00015257	0.00010533	57
hsa04810	Regulation of actin cytoskeleton	85/2147	229/8773	1.08E-05	0.00018216	0.00012577	85
hsa05212	Pancreatic cancer	36/2147	76/8773	1.13E-05	0.00018216	0.00012577	36
hsa05210	Colorectal cancer	39/2147	86/8773	1.78E-05	0.00026763	0.00018478	39
hsa04512	ECM-receptor interaction	40/2147	89/8773	1.81E-05	0.00026763	0.00018478	40
hsa04659	Th17 cell differentiation	46/2147	108/8773	2.44E-05	0.00034617	0.000239	46
hsa05220	Chronic myeloid leukemia	35/2147	76/8773	3.16E-05	0.00043042	0.00029716	35
hsa05222	Small cell lung cancer	40/2147	92/8773	4.63E-05	0.00058461	0.00040362	40
hsa05235	PD-L1 expression and PD-1 checkpoint pathway in cancer	39/2147	89/8773	4.64E-05	0.00058461	0.00040362	39
hsa04668	TNF signaling pathway	47/2147	114/8773	5.43E-05	0.00065921	0.00045512	47
hsa05416	Viral myocarditis	32/2147	69/8773	5.77E-05	0.00067598	0.00046669	32
hsa05417	Lipid and atherosclerosis	78/2147	215/8773	6.11E-05	0.00069087	0.00047698	78
hsa04148	Efferocytosis	60/2147	156/8773	6.44E-05	0.00069087	0.00047698	60
hsa05170	Human immunodeficiency virus 1 infection	77/2147	212/8773	6.50E-05	0.00069087	0.00047698	77
hsa05135	Yersinia infection	54/2147	137/8773	6.86E-05	0.00070336	0.0004856	54
hsa04670	Leukocyte transendothelial migration	47/2147	115/8773	7.03E-05	0.00070336	0.0004856	47
hsa05160	Hepatitis C	60/2147	158/8773	9.85E-05	0.00095689	0.00066064	60
hsa05163	Human cytomegalovirus infection	80/2147	225/8773	0.0001085	0.00102476	0.0007075	80
hsa04151	PI3K-Akt signaling pathway	118/2147	359/8773	0.00015326	0.0014083	0.00097229	118
hsa05215	Prostate cancer	40/2147	97/8773	0.0001892	0.00169284	0.00116874	40
hsa05225	Hepatocellular carcinoma	62/2147	168/8773	0.00019543	0.00170371	0.00117624	62
hsa04350	TGF-beta signaling pathway	43/2147	108/8773	0.00028092	0.0023296	0.00160836	43
hsa04620	Toll-like receptor signaling pathway	43/2147	108/8773	0.00028092	0.0023296	0.00160836	43
hsa04660	T cell receptor signaling pathway	47/2147	121/8773	0.00029551	0.00239226	0.00165162	47
hsa04010	MAPK signaling pathway	99/2147	299/8773	0.0003817	0.00301805	0.00208367	99
hsa05202	Transcriptional misregulation in cancer	68/2147	193/8773	0.00046474	0.00359115	0.00247934	68

hsa05133	Pertussis	32/2147	76/8773	0.00051958	0.00392574	0.00271034	32
hsa04820	Cytoskeleton in muscle cells	78/2147	229/8773	0.00060539	0.00442304	0.00305368	78
hsa04014	Ras signaling pathway	80/2147	236/8773	0.00061142	0.00442304	0.00305368	80
hsa05131	Shigellosis	83/2147	247/8773	0.00066256	0.00469316	0.00324017	83
hsa04210	Apoptosis	50/2147	135/8773	0.00071131	0.00493565	0.00340759	50
hsa04662	B cell receptor signaling pathway	36/2147	90/8773	0.00077071	0.00520386	0.00359276	36
hsa04015	Rap1 signaling pathway	72/2147	210/8773	0.00078397	0.00520386	0.00359276	72
hsa03030	DNA replication	18/2147	36/8773	0.00079588	0.00520386	0.00359276	18
hsa05165	Human papillomavirus infection	106/2147	331/8773	0.00093055	0.00596955	0.00412139	106
hsa05211	Renal cell carcinoma	29/2147	69/8773	0.00096649	0.0060853	0.00420131	29
hsa01521	EGFR tyrosine kinase inhibitor resistance	32/2147	79/8773	0.00115181	0.00712026	0.00491584	32
hsa04068	FoxO signaling pathway	48/2147	131/8773	0.0011776	0.00714969	0.00493616	48
hsa04658	Th1 and Th2 cell differentiation	36/2147	92/8773	0.0012366	0.0073762	0.00509255	36
hsa05142	Chagas disease	39/2147	102/8773	0.00132705	0.00777309	0.00536656	39
hsa04621	NOD-like receptor signaling pathway	64/2147	186/8773	0.00134886	0.00777309	0.00536656	64
hsa04392	Hippo signaling pathway - multiple species	15/2147	29/8773	0.00138951	0.0078739	0.00543616	15
hsa05418	Fluid shear stress and atherosclerosis	50/2147	139/8773	0.00149911	0.00828491	0.00571992	50
hsa04926	Relaxin signaling pathway	47/2147	129/8773	0.00151078	0.00828491	0.00571992	47
hsa04141	Protein processing in endoplasmic reticulum	59/2147	170/8773	0.00161857	0.00873514	0.00603077	59
hsa05321	Inflammatory bowel disease	27/2147	65/8773	0.00175833	0.00934115	0.00644915	27
hsa04722	Neurotrophin signaling pathway	43/2147	119/8773	0.0028095	0.01464392	0.0101102	43
hsa04917	Prolactin signaling pathway	28/2147	70/8773	0.00284264	0.01464392	0.0101102	28
hsa05323	Rheumatoid arthritis	35/2147	93/8773	0.00311591	0.01581206	0.01091668	35
hsa04672	Intestinal immune network for IgA production	21/2147	49/8773	0.00352798	0.01763988	0.01217862	21
hsa04940	Type I diabetes mellitus	19/2147	43/8773	0.0035866	0.01767312	0.01220157	19
hsa04611	Platelet activation	44/2147	124/8773	0.00374935	0.01821111	0.01257299	44
hsa03430	Mismatch repair	12/2147	23/8773	0.00380497	0.018221	0.01257983	12
hsa04630	JAK-STAT signaling pathway	56/2147	166/8773	0.00423857	0.01991662	0.01375048	56
hsa04115	p53 signaling pathway	29/2147	75/8773	0.00433479	0.01991662	0.01375048	29
hsa05214	Glioma	29/2147	75/8773	0.00433479	0.01991662	0.01375048	29
hsa05223	Non-small cell lung cancer	28/2147	72/8773	0.00456293	0.02047535	0.01413623	28
hsa00510	N-Glycan biosynthesis	22/2147	53/8773	0.00457684	0.02047535	0.01413623	22
hsa00514	Other types of O-glycan biosynthesis	20/2147	47/8773	0.00480942	0.02123641	0.01466167	20
hsa05330	Allograft rejection	17/2147	38/8773	0.00492813	0.02148159	0.01483094	17
hsa04390	Hippo signaling pathway	53/2147	157/8773	0.0052184	0.02245893	0.0155057	53
hsa04640	Hematopoietic cell lineage	36/2147	99/8773	0.00528504	0.02246143	0.01550743	36
hsa05224	Breast cancer	50/2147	147/8773	0.00554579	0.02317605	0.0160008	50
hsa04360	Axon guidance	60/2147	182/8773	0.00558952	0.02317605	0.0160008	60
hsa03083	Polycomb repressive complex	31/2147	83/8773	0.005935	0.02431203	0.01678509	31
hsa04657	IL-17 signaling pathway	34/2147	94/8773	0.00724	0.02930476	0.02023208	34
hsa04120	Ubiquitin mediated proteolysis	48/2147	142/8773	0.00739089	0.02936594	0.02027432	48
hsa05226	Gastric cancer	50/2147	149/8773	0.00742786	0.02936594	0.02027432	50
hsa04612	Antigen processing and presentation	29/2147	78/8773	0.00820166	0.03205247	0.02212911	29
hsa04064	NF-kappa B signaling pathway	37/2147	105/8773	0.0084435	0.0326226	0.02252273	37
hsa04550	Signaling pathways regulating pluripotency of stem cells	48/2147	143/8773	0.00854412	0.03264046	0.02253506	48
hsa05218	Melanoma	27/2147	72/8773	0.00926039	0.03498371	0.02415284	27

hsa04610	Complement and coagulation cascades	31/2147	86/8773	0.01059913	0.03935879	0.02717341	31
hsa00910	Nitrogen metabolism	9/2147	17/8773	0.01065003	0.03935879	0.02717341	9
hsa03440	Homologous recombination	17/2147	41/8773	0.01213676	0.0438989	0.03030791	17
hsa05219	Bladder cancer	17/2147	41/8773	0.01213676	0.0438989	0.03030791	17
hsa04666	Fc gamma R-mediated phagocytosis	34/2147	97/8773	0.0122829	0.04395985	0.03034999	34
hsa04144	Endocytosis	77/2147	250/8773	0.01248433	0.04421535	0.03052639	77
hsa05332	Graft-versus-host disease	17/2147	42/8773	0.01584181	0.05498407	0.03796114	17
hsa05146	Amoebiasis	35/2147	102/8773	0.01584835	0.05498407	0.03796114	35
hsa05203	Viral carcinogenesis	63/2147	204/8773	0.02098865	0.07208224	0.04976575	63
hsa04625	C-type lectin receptor signaling pathway	35/2147	104/8773	0.02149382	0.07307899	0.05045392	35
hsa05144	Malaria	19/2147	50/8773	0.02293447	0.07707848	0.05321517	19
hsa04066	HIF-1 signaling pathway	36/2147	108/8773	0.02312354	0.07707848	0.05321517	36
hsa05221	Acute myeloid leukemia	24/2147	67/8773	0.02458444	0.08115253	0.05602791	24
hsa04216	Ferroptosis	16/2147	41/8773	0.02731861	0.08882304	0.06132365	16
hsa04062	Chemokine signaling pathway	59/2147	192/8773	0.02743065	0.08882304	0.06132365	59
hsa05412	Arrhythmogenic right ventricular cardiomyopathy	29/2147	86/8773	0.03326494	0.10669888	0.07366517	29
hsa00513	Various types of N-glycan biosynthesis	16/2147	42/8773	0.03438125	0.10924883	0.07542566	16
hsa00520	Amino sugar and nucleotide sugar metabolism	18/2147	49/8773	0.03724052	0.11723867	0.08094187	18
hsa00512	Mucin type O-glycan biosynthesis	14/2147	36/8773	0.03881234	0.12106603	0.08358428	14
hsa03250	Viral life cycle - HIV-1	22/2147	63/8773	0.0404794	0.12511814	0.08638187	22
hsa04310	Wnt signaling pathway	53/2147	174/8773	0.04098726	0.12554658	0.08667767	53
hsa04725	Cholinergic synapse	36/2147	113/8773	0.04484152	0.13612605	0.09398176	36
hsa04514	Cell adhesion molecules	48/2147	157/8773	0.04695979	0.14129493	0.09755037	48
hsa04012	ErbB signaling pathway	28/2147	85/8773	0.04795882	0.14303508	0.09875178	28
hsa01250	Biosynthesis of nucleotide sugars	14/2147	37/8773	0.04880495	0.14378616	0.09927032	14
hsa04071	Sphingolipid signaling pathway	38/2147	121/8773	0.04925983	0.14378616	0.09927032	38
hsa00770	Pantothenate and CoA biosynthesis	9/2147	21/8773	0.04947936	0.14378616	0.09927032	9

Table S18 KEGG pathways based on DEGs between high and low SEPN1 expression groups in CGGA-325.

ID	Description	GeneRatio	BgRatio	P-value	P-adjust	Q-value	Count
hsa04080	Neuroactive ligand-receptor interaction	108/1052	366/8773	2.76E-20	8.90E-18	6.60E-18	108
hsa05033	Nicotine addiction	26/1052	40/8773	3.70E-15	5.96E-13	4.42E-13	26
hsa05140	Leishmaniasis	34/1052	77/8773	1.39E-12	1.49E-10	1.11E-10	34
hsa04512	ECM-receptor interaction	35/1052	89/8773	3.68E-11	2.96E-09	2.20E-09	35
hsa04940	Type I diabetes mellitus	23/1052	43/8773	4.64E-11	2.99E-09	2.22E-09	23
hsa04610	Complement and coagulation cascades	34/1052	86/8773	5.90E-11	3.17E-09	2.35E-09	34
hsa04145	Phagosome	48/1052	152/8773	9.20E-11	3.88E-09	2.88E-09	48
hsa05150	Staphylococcus aureus infection	36/1052	96/8773	9.64E-11	3.88E-09	2.88E-09	36
hsa05323	Rheumatoid arthritis	35/1052	93/8773	1.57E-10	5.60E-09	4.16E-09	35
hsa05332	Graft-versus-host disease	21/1052	42/8773	1.68E-09	5.40E-08	4.01E-08	21
hsa05145	Toxoplasmosis	37/1052	111/8773	2.52E-09	7.38E-08	5.48E-08	37
hsa04514	Cell adhesion molecules	46/1052	157/8773	3.50E-09	9.39E-08	6.97E-08	46
hsa04672	Intestinal immune network for IgA production	22/1052	49/8773	8.90E-09	2.20E-07	1.64E-07	22
hsa05330	Allograft rejection	19/1052	38/8773	1.01E-08	2.33E-07	1.73E-07	19
hsa04727	GABAergic synapse	31/1052	89/8773	1.53E-08	3.28E-07	2.44E-07	31
hsa04640	Hematopoietic cell lineage	33/1052	99/8773	1.84E-08	3.70E-07	2.74E-07	33
hsa04510	Focal adhesion	52/1052	202/8773	4.41E-08	8.36E-07	6.20E-07	52
hsa04024	cAMP signaling pathway	56/1052	225/8773	4.76E-08	8.51E-07	6.31E-07	56
hsa04974	Protein digestion and absorption	33/1052	103/8773	5.49E-08	9.30E-07	6.90E-07	33
hsa04820	Cytoskeleton in muscle cells	56/1052	229/8773	9.11E-08	1.42E-06	1.06E-06	56
hsa04724	Glutamatergic synapse	35/1052	115/8773	9.29E-08	1.42E-06	1.06E-06	35
hsa04020	Calcium signaling pathway	60/1052	253/8773	1.02E-07	1.50E-06	1.11E-06	60
hsa05416	Viral myocarditis	25/1052	69/8773	1.57E-07	2.20E-06	1.63E-06	25
hsa05133	Pertussis	26/1052	76/8773	3.33E-07	4.31E-06	3.20E-06	26
hsa05152	Tuberculosis	46/1052	180/8773	3.34E-07	4.31E-06	3.20E-06	46
hsa05032	Morphine addiction	29/1052	91/8773	3.97E-07	4.92E-06	3.65E-06	29
hsa04721	Synaptic vesicle cycle	26/1052	78/8773	5.93E-07	7.08E-06	5.25E-06	26
hsa04060	Cytokine-cytokine receptor interaction	64/1052	297/8773	1.51E-06	1.73E-05	1.29E-05	64
hsa05143	African trypanosomiasis	16/1052	37/8773	1.80E-06	2.00E-05	1.48E-05	16
hsa04612	Antigen processing and presentation	25/1052	78/8773	2.22E-06	2.38E-05	1.77E-05	25
hsa04670	Leukocyte transendothelial migration	32/1052	115/8773	2.95E-06	3.06E-05	2.27E-05	32
hsa05321	Inflammatory bowel disease	22/1052	65/8773	3.26E-06	3.28E-05	2.43E-05	22
hsa05310	Asthma	14/1052	31/8773	4.29E-06	4.19E-05	3.11E-05	14
hsa05205	Proteoglycans in cancer	47/1052	203/8773	5.06E-06	4.79E-05	3.55E-05	47
hsa05146	Amoebiasis	29/1052	102/8773	5.36E-06	4.93E-05	3.66E-05	29
hsa04725	Cholinergic synapse	31/1052	113/8773	5.80E-06	4.96E-05	3.68E-05	31
hsa05320	Autoimmune thyroid disease	19/1052	53/8773	5.80E-06	4.96E-05	3.68E-05	19
hsa05166	Human T-cell leukemia virus 1 infection	50/1052	222/8773	5.85E-06	4.96E-05	3.68E-05	50
hsa04659	Th17 cell differentiation	30/1052	108/8773	6.21E-06	5.13E-05	3.81E-05	30
hsa04380	Osteoclast differentiation	36/1052	141/8773	6.44E-06	5.19E-05	3.85E-05	36
hsa04061	Viral protein interaction with cytokine and cytokine receptor	28/1052	100/8773	1.07E-05	8.38E-05	6.22E-05	28
hsa04726	Serotonergic synapse	30/1052	115/8773	2.39E-05	0.0001836	0.00013625	30
hsa04713	Circadian entrainment	26/1052	97/8773	5.00E-05	0.00037413	0.00027763	26
hsa04658	Th1 and Th2 cell differentiation	25/1052	92/8773	5.42E-05	0.00039666	0.00029435	25
hsa04010	MAPK signaling pathway	59/1052	299/8773	6.25E-05	0.00044699	0.0003317	59

hsa04151	PI3K-Akt signaling pathway	68/1052	359/8773	6.75E-05	0.00047225	0.00035044	68
hsa05031	Amphetamine addiction	20/1052	69/8773	0.00011262	0.00077158	0.00057257	20
hsa05030	Cocaine addiction	16/1052	49/8773	0.00011543	0.00077435	0.00057463	16
hsa04723	Retrograde endocannabinoid signaling	34/1052	148/8773	0.00011827	0.00077717	0.00057672	34
hsa05144	Malaria	16/1052	50/8773	0.00015112	0.00097323	0.00072221	16
hsa05410	Hypertrophic cardiomyopathy	25/1052	99/8773	0.00019583	0.00123644	0.00091753	25
hsa05169	Epstein-Barr virus infection	42/1052	202/8773	0.00021895	0.00135578	0.00100609	42
hsa04933	AGE-RAGE signaling pathway in diabetic complications	25/1052	100/8773	0.000232	0.00140953	0.00104597	25
hsa05142	Chagas disease	25/1052	102/8773	0.00032248	0.00192291	0.00142694	25
hsa04728	Dopaminergic synapse	30/1052	132/8773	0.00035521	0.0020796	0.00154321	30
hsa04911	Insulin secretion	22/1052	86/8773	0.00038233	0.00219841	0.00163138	22
hsa05414	Dilated cardiomyopathy	25/1052	105/8773	0.00051635	0.00291695	0.00216459	25
hsa04814	Motor proteins	38/1052	193/8773	0.00127332	0.00706911	0.00524579	38
hsa04668	TNF signaling pathway	25/1052	114/8773	0.00182404	0.00995491	0.00738727	25
hsa05412	Arrhythmogenic right ventricular cardiomyopathy	20/1052	86/8773	0.00243983	0.01309374	0.0097165	20
hsa04064	NF-kappa B signaling pathway	23/1052	105/8773	0.00276861	0.01461464	0.01084513	23
hsa04650	Natural killer cell mediated cytotoxicity	27/1052	132/8773	0.0035189	0.01827559	0.01356181	27
hsa04261	Adrenergic signaling in cardiomyocytes	30/1052	154/8773	0.00463654	0.02369785	0.01758553	30
hsa04720	Long-term potentiation	16/1052	67/8773	0.00483786	0.02434048	0.01806241	16
hsa05134	Legionellosis	14/1052	56/8773	0.00528824	0.02605156	0.01933215	14
hsa04360	Axon guidance	34/1052	182/8773	0.00535991	0.02605156	0.01933215	34
hsa04750	Inflammatory mediator regulation of TRP channels	21/1052	98/8773	0.00542067	0.02605156	0.01933215	21
hsa05170	Human immunodeficiency virus 1 infection	38/1052	212/8773	0.00685376	0.03245457	0.02408365	38
hsa04929	GnRH secretion	15/1052	64/8773	0.00751665	0.03507769	0.02603019	15
hsa04921	Oxytocin signaling pathway	29/1052	154/8773	0.00862359	0.03915397	0.02905509	29
hsa05417	Lipid and atherosclerosis	38/1052	215/8773	0.00863333	0.03915397	0.02905509	38
hsa04926	Relaxin signaling pathway	25/1052	129/8773	0.00985526	0.04407492	0.03270679	25
hsa04978	Mineral absorption	14/1052	60/8773	0.01003071	0.04424504	0.03283303	14
hsa04148	Efferocytosis	29/1052	156/8773	0.01030221	0.04482852	0.03326602	29
hsa05322	Systemic lupus erythematosus	26/1052	137/8773	0.0112689	0.04838115	0.03590233	26
hsa05222	Small cell lung cancer	19/1052	92/8773	0.01180767	0.05002724	0.03712384	19
hsa04062	Chemokine signaling pathway	34/1052	192/8773	0.01216779	0.05088349	0.03775924	34
hsa05418	Fluid shear stress and atherosclerosis	26/1052	139/8773	0.01352036	0.0558148	0.04141863	26
hsa05167	Kaposi sarcoma-associated herpesvirus infection	34/1052	194/8773	0.01414585	0.05756032	0.04271393	34
hsa04810	Regulation of actin cytoskeleton	39/1052	229/8773	0.0143007	0.05756032	0.04271393	39
hsa04971	Gastric acid secretion	16/1052	76/8773	0.01665739	0.06621828	0.04913877	16
hsa04930	Type II diabetes mellitus	11/1052	46/8773	0.01775362	0.06971542	0.0517339	11
hsa05164	Influenza A	30/1052	171/8773	0.02002453	0.07768553	0.0576483	30
hsa04961	Endocrine and other factor-regulated calcium reabsorption	12/1052	53/8773	0.02075781	0.0795716	0.0590479	12
hsa05165	Human papillomavirus infection	52/1052	331/8773	0.02377067	0.09004888	0.0668228	52
hsa04742	Taste transduction	17/1052	86/8773	0.02500356	0.09361797	0.06947133	17
hsa04066	HIF-1 signaling pathway	20/1052	108/8773	0.03075971	0.11284465	0.08373892	20
hsa04115	p53 signaling pathway	15/1052	75/8773	0.03083953	0.11284465	0.08373892	15
hsa00330	Arginine and proline metabolism	11/1052	50/8773	0.03194004	0.11555835	0.08575268	11

hsa04662	B cell receptor signaling pathway	17/1052	90/8773	0.0372521	0.13304682	0.09873039	17
hsa04611	Platelet activation	22/1052	124/8773	0.03760019	0.13304682	0.09873039	22
hsa04666	Fc gamma R-mediated phagocytosis	18/1052	97/8773	0.03831914	0.13411698	0.09952453	18
hsa04015	Rap1 signaling pathway	34/1052	210/8773	0.04092495	0.14169715	0.10514957	34
hsa00910	Nitrogen metabolism	5/1052	17/8773	0.04430363	0.15176349	0.11261952	5
hsa05130	Pathogenic Escherichia coli infection	32/1052	198/8773	0.04723052	0.16008659	0.11879587	32

Table S19 KEGG pathways based on DEGs between high and low SEPN1 expression groups in GSE16011.

ID	Description	GeneRatio	BgRatio	P-value	P-adjust	Q-value	Count
hsa04610	Complement and coagulation cascades	19/226	86/8773	3.58E-13	9.95E-11	7.42E-11	19
hsa04510	Focal adhesion	25/226	202/8773	5.40E-11	7.21E-09	5.38E-09	25
hsa04512	ECM-receptor interaction	17/226	89/8773	7.78E-11	7.21E-09	5.38E-09	17
hsa05133	Pertussis	14/226	76/8773	6.33E-09	4.40E-07	3.28E-07	14
hsa04933	AGE-RAGE signaling pathway in diabetic complications	15/226	100/8773	3.34E-08	1.74E-06	1.30E-06	15
hsa05142	Chagas disease	15/226	102/8773	4.39E-08	1.74E-06	1.30E-06	15
hsa05146	Amoebiasis	15/226	102/8773	4.39E-08	1.74E-06	1.30E-06	15
hsa05150	Staphylococcus aureus infection	13/226	96/8773	9.49E-07	3.30E-05	2.46E-05	13
hsa05205	Proteoglycans in cancer	19/226	203/8773	1.09E-06	3.36E-05	2.50E-05	19
hsa05323	Rheumatoid arthritis	12/226	93/8773	4.21E-06	0.00010758	8.02E-05	12
hsa05144	Malaria	9/226	50/8773	4.26E-06	0.00010758	8.02E-05	9
hsa04820	Cytoskeleton in muscle cells	19/226	229/8773	6.55E-06	0.00015172	0.00011317	19
hsa04974	Protein digestion and absorption	12/226	103/8773	1.23E-05	0.00026357	0.00019661	12
hsa05165	Human papillomavirus infection	23/226	331/8773	1.35E-05	0.00026743	0.00019949	23
hsa04727	GABAergic synapse	11/226	89/8773	1.62E-05	0.00030053	0.00022417	11
hsa04926	Relaxin signaling pathway	13/226	129/8773	2.61E-05	0.00045371	0.00033843	13
hsa05033	Nicotine addiction	7/226	40/8773	6.20E-05	0.00101334	0.00075588	7
hsa04723	Retrograde endocannabinoid signaling	13/226	148/8773	0.00010989	0.00169724	0.00126602	13
hsa05145	Toxoplasmosis	11/226	111/8773	0.00012786	0.00187083	0.00139551	11
hsa04151	PI3K-Akt signaling pathway	22/226	359/8773	0.0001378	0.00190677	0.00142232	22
hsa04145	Phagosome	13/226	152/8773	0.00014404	0.00190677	0.00142232	13
hsa04721	Synaptic vesicle cycle	9/226	78/8773	0.00016573	0.00209426	0.00156217	9
hsa04514	Cell adhesion molecules	13/226	157/8773	0.00019926	0.00240846	0.00179654	13
hsa05418	Fluid shear stress and atherosclerosis	12/226	139/8773	0.00023759	0.00275209	0.00205286	12
hsa04350	TGF-beta signaling pathway	10/226	108/8773	0.00044956	0.00499915	0.00372901	10
hsa04148	Efferocytosis	12/226	156/8773	0.00068241	0.00729658	0.00544273	12
hsa05152	Tuberculosis	13/226	180/8773	0.00074792	0.00770084	0.00574428	13
hsa05140	Leishmaniasis	8/226	77/8773	0.00078091	0.00775335	0.00578345	8
hsa04936	Alcoholic liver disease	11/226	142/8773	0.00107201	0.01027649	0.00766554	11
hsa04064	NF-kappa B signaling pathway	9/226	105/8773	0.00149876	0.01388854	0.01035987	9
hsa04010	MAPK signaling pathway	17/226	299/8773	0.00182293	0.01634761	0.01219416	17
hsa04728	Dopaminergic synapse	10/226	132/8773	0.00211968	0.01841474	0.0137361	10
hsa05032	Morphine addiction	8/226	91/8773	0.00231903	0.01953609	0.01457255	8
hsa05171	Coronavirus disease - COVID-19	14/226	233/8773	0.00276392	0.02170728	0.0161921	14
hsa05322	Systemic lupus erythematosus	10/226	137/8773	0.00278643	0.02170728	0.0161921	10
hsa04724	Glutamatergic synapse	9/226	115/8773	0.00281101	0.02170728	0.0161921	9
hsa05134	Legionellosis	6/226	56/8773	0.00302931	0.02252556	0.01680248	6
hsa04115	p53 signaling pathway	7/226	75/8773	0.00307903	0.02252556	0.01680248	7
hsa05417	Lipid and atherosclerosis	13/226	215/8773	0.00367684	0.02620927	0.01955027	13
hsa05164	Influenza A	11/226	171/8773	0.00463194	0.03115114	0.02323656	11
hsa05132	Salmonella infection	14/226	247/8773	0.00466351	0.03115114	0.02323656	14
hsa04940	Type I diabetes mellitus	5/226	43/8773	0.00470629	0.03115114	0.02323656	5
hsa04390	Hippo signaling pathway	10/226	157/8773	0.00729307	0.04715055	0.03517099	10
hsa05310	Asthma	4/226	31/8773	0.00781589	0.04938223	0.03683566	4
hsa04672	Intestinal immune network for IgA production	5/226	49/8773	0.00823278	0.05079083	0.03788638	5

hsa05416	Viral myocarditis	6/226	69/8773	0.0084875	0.05079083	0.03788638	6
hsa04621	NOD-like receptor signaling pathway	11/226	186/8773	0.00858694	0.05079083	0.03788638	11
hsa04668	TNF signaling pathway	8/226	114/8773	0.00908686	0.05262808	0.03925684	8
hsa05222	Small cell lung cancer	7/226	92/8773	0.00941948	0.05313183	0.0396326	7
hsa04726	Serotonergic synapse	8/226	115/8773	0.00955608	0.05313183	0.0396326	8
hsa04060	Cytokine-cytokine receptor interaction	15/226	297/8773	0.00977195	0.0532667	0.03973321	15
hsa04380	Osteoclast differentiation	9/226	141/8773	0.0105463	0.05638216	0.04205712	9
hsa05131	Shigellosis	13/226	247/8773	0.01140914	0.05954296	0.04441485	13
hsa05167	Kaposi sarcoma-associated herpesvirus infection	11/226	194/8773	0.0115659	0.05954296	0.04441485	11
hsa04713	Circadian entrainment	7/226	97/8773	0.01240688	0.06271112	0.04677808	7
hsa05212	Pancreatic cancer	6/226	76/8773	0.01335166	0.06555423	0.04889884	6
hsa05163	Human cytomegalovirus infection	12/226	225/8773	0.01344098	0.06555423	0.04889884	12
hsa04640	Hematopoietic cell lineage	7/226	99/8773	0.0137746	0.0660231	0.04924859	7
hsa05100	Bacterial invasion of epithelial cells	6/226	77/8773	0.01417806	0.0668051	0.0498319	6
hsa05169	Epstein-Barr virus infection	11/226	202/8773	0.0152784	0.07078992	0.0528043	11
hsa05330	Allograft rejection	4/226	38/8773	0.01594616	0.07267266	0.05420868	4
hsa04216	Ferroptosis	4/226	41/8773	0.02061227	0.09095573	0.06784657	4
hsa05219	Bladder cancer	4/226	41/8773	0.02061227	0.09095573	0.06784657	4
hsa04659	Th17 cell differentiation	7/226	108/8773	0.02128386	0.09245178	0.06896252	7
hsa05332	Graft-versus-host disease	4/226	42/8773	0.022335	0.09552507	0.07125497	4
hsa04911	Insulin secretion	6/226	86/8773	0.02327068	0.09801893	0.07311522	6
hsa05321	Inflammatory bowel disease	5/226	65/8773	0.0256598	0.10646903	0.0794184	5
hsa04080	Neuroactive ligand-receptor interaction	16/226	366/8773	0.0269357	0.11011949	0.08214138	16
hsa05202	Transcriptional misregulation in cancer	10/226	193/8773	0.02745795	0.11062769	0.08252046	10
hsa05166	Human T-cell leukemia virus 1 infection	11/226	222/8773	0.0284264	0.11242791	0.0838633	11
hsa04670	Leukocyte transendothelial migration	7/226	115/8773	0.02881142	0.11242791	0.0838633	7
hsa05206	MicroRNAs in cancer	14/226	310/8773	0.02934466	0.11242791	0.0838633	14
hsa05225	Hepatocellular carcinoma	9/226	168/8773	0.02952244	0.11242791	0.0838633	9
hsa05130	Pathogenic Escherichia coli infection	10/226	198/8773	0.03197056	0.11769051	0.08778883	10
hsa05031	Amphetamine addiction	5/226	69/8773	0.03217438	0.11769051	0.08778883	5
hsa05211	Renal cell carcinoma	5/226	69/8773	0.03217438	0.11769051	0.08778883	5
hsa00601	Glycosphingolipid biosynthesis - lacto and neolacto series	3/226	28/8773	0.03441135	0.12423837	0.09267307	3
hsa05410	Hypertrophic cardiomyopathy	6/226	99/8773	0.04227842	0.15068464	0.11240013	6
hsa05220	Chronic myeloid leukemia	5/226	76/8773	0.04587286	0.161426	0.12041243	5
hsa05320	Autoimmune thyroid disease	4/226	53/8773	0.04704848	0.16349346	0.12195461	4
hsa04218	Cellular senescence	8/226	156/8773	0.04833356	0.16588555	0.12373894	8

Table S20 LncRNAs and corresponding coefficients for calculating SRS.

LncRNA	Coefficient
C1RL-AS1	0.055871473
CRNDE	0.121255726
FAM181A-AS1	0.000650303
GLIS2-AS1	-0.06490602
GNAS-AS1	0.170592242
LINC00929	0.778957079
LINC01503	0.070169231
LINC01841	13.59035357
LINC01842	0.040480769
LINC01948	0.805276213
LINC02044	-0.567101044
LINC02454	0.054868318
MIR3681HG	33.86229365
PKN2-AS1	2.086251859
SFTA3	-0.126458348
SLC16A1-AS1	0.200101543
TGFB2-AS1	0.002034415
TRBV11-2	0.025235325
WAC-AS1	-0.006529102

Table S21 Comparing RMST between high and low SRS groups in the train cohort**.**

Year	High SRS RMST	Low SRS RMST	Estimation	LCI	UCI	P-value
1	11.420	11.868	-0.448	-0.797	-0.100	0.011678028
2	21.111	23.601	-2.490	-3.518	-1.463	2.04E-06
3	28.932	35.217	-6.285	-8.202	-4.369	1.29E-10
4	35.490	46.230	-10.741	-13.724	-7.757	1.73E-12
5	41.451	56.081	-14.630	-18.969	-10.292	3.85E-11
6	46.351	65.251	-18.900	-24.761	-13.039	2.60E-10
7	50.457	73.863	-23.406	-30.959	-15.853	1.25E-09
8	54.155	81.817	-27.662	-37.063	-18.261	8.05E-09
9	57.244	88.776	-31.533	-42.818	-20.248	4.34E-08
10	59.961	95.516	-35.556	-48.877	-22.235	1.68E-07
11	62.020	101.689	-39.669	-55.050	-24.288	4.31E-07
12	63.358	106.756	-43.398	-60.607	-26.189	7.70E-07
13	64.388	110.298	-45.910	-64.818	-27.002	1.95E-06
14	65.417	112.458	-47.041	-67.559	-26.523	7.01E-06
15	66.447	113.887	-47.440	-69.499	-25.380	2.50E-05

Abbreviations: RMST, restricted mean survival time; LCI, lower limit of 95% confidence interval; UCI, upper limit of 95% confidence interval.

Table S22 Comparing RMST between high and low SRS groups in the test cohort**.**

Year	High SRS RMST	Low SRS RMST	Estimation	LCI	UCI	P-value
1	11.689	12.000	-0.311	-0.584	-0.038	0.025674964
2	21.564	24.000	-2.436	-3.637	-1.236	6.92E-05
3	29.080	35.720	-6.640	-9.137	-4.143	1.87E-07
4	34.993	46.842	-11.849	-16.019	-7.679	2.56E-08
5	39.649	57.114	-17.465	-23.549	-11.382	1.83E-08
6	43.682	67.099	-23.417	-31.749	-15.084	3.63E-08
7	47.115	76.350	-29.235	-39.982	-18.488	9.73E-08
8	50.547	85.225	-34.678	-48.259	-21.097	5.60E-07
9	52.202	94.101	-41.899	-57.899	-25.899	2.86E-07
10	52.202	102.976	-50.774	-68.578	-32.971	2.27E-08
11	52.202	111.851	-59.650	-79.449	-39.850	3.53E-09
12	52.202	119.272	-67.070	-88.789	-45.352	1.43E-09
13	52.202	124.277	-72.075	-95.652	-48.498	2.08E-09
14	52.202	128.714	-76.513	-102.453	-50.573	7.42E-09

Abbreviations: RMST, restricted mean survival time; LCI, lower limit of 95% confidence interval; UCI, upper limit of 95% confidence interval.

Table S23 Comparing RMST between high and low SRS groups in the whole TCGA-LGG cohort**.**

Year	High SRS RMST	Low SRS RMST	Estimation	LCI	UCI	P-value
1	11.501	11.907	-0.406	-0.665	-0.147	0.002090455
2	21.242	23.719	-2.476	-3.282	-1.670	1.74E-09
3	28.977	35.367	-6.390	-7.928	-4.852	3.82E-16
4	35.342	46.422	-11.079	-13.512	-8.647	4.36E-19
5	40.885	56.397	-15.512	-19.054	-11.971	9.09E-18
6	45.499	65.815	-20.317	-25.127	-15.507	1.25E-16
7	49.373	74.631	-25.258	-31.459	-19.057	1.42E-15
8	52.943	82.858	-29.915	-37.662	-22.169	3.77E-14
9	55.584	90.345	-34.762	-44.031	-25.493	1.97E-13
10	57.353	97.663	-40.310	-51.143	-29.478	3.02E-13
11	58.694	104.530	-45.837	-58.310	-33.363	5.93E-13
12	59.565	110.218	-50.652	-64.605	-36.699	1.12E-12
13	60.236	114.152	-53.916	-69.221	-38.612	5.03E-12
14	60.906	116.991	-56.084	-72.768	-39.401	4.44E-11
15	61.577	119.191	-57.614	-75.687	-39.542	4.15E-10

Abbreviations: RMST, restricted mean survival time; LCI, lower limit of 95% confidence interval; UCI, upper limit of 95% confidence interval.

Table S24 Comparing RMST between high and low SRS groups in CGGA-693.

Year	High SRS RMST	Low SRS RMST	Estimation	LCI	UCI	P-value
1	10.182	11.389	-1.207	-1.589	-0.826	5.71E-10
2	16.053	21.373	-5.320	-6.376	-4.265	5.27E-23
3	20.105	30.244	-10.139	-11.898	-8.380	1.35E-29
4	23.306	38.317	-15.011	-17.488	-12.534	1.55E-32
5	26.153	45.884	-19.731	-22.948	-16.513	2.80E-33
6	28.646	53.024	-24.379	-28.340	-20.417	1.69E-33
7	30.786	59.861	-29.076	-33.782	-24.369	9.62E-34
8	32.631	66.138	-33.507	-38.950	-28.065	1.58E-33
9	34.385	71.824	-37.439	-43.653	-31.225	3.54E-32
10	36.139	76.920	-40.781	-47.846	-33.715	1.14E-29
11	36.524	81.952	-45.428	-53.216	-37.640	2.86E-30
12	36.524	86.985	-50.460	-59.010	-41.910	6.04E-31
13	36.524	92.017	-55.493	-64.891	-46.094	5.68E-31

Abbreviations: RMST, restricted mean survival time; LCI, lower limit of 95% confidence interval; UCI, upper limit of 95% confidence interval.

Table S25 Comparing RMST between high and low SRS groups in CGGA-325.

Year	High SRS RMST	Low SRS RMST	Estimation	LCI	UCI	P-value
1	9.513	11.444	-1.931	-2.532	-1.329	3.18E-10
2	14.009	21.408	-7.399	-8.935	-5.863	3.68E-21
3	16.879	30.508	-13.628	-16.124	-11.133	9.89E-27
4	18.522	38.782	-20.261	-23.622	-16.899	3.29E-32
5	19.705	46.590	-26.885	-31.125	-22.644	1.89E-35
6	20.772	54.018	-33.246	-38.406	-28.086	1.49E-36
7	21.701	60.907	-39.206	-45.273	-33.139	9.15E-37
8	22.532	67.137	-44.605	-51.561	-37.649	3.16E-36
9	23.217	73.270	-50.053	-57.907	-42.199	8.40E-36
10	23.674	79.011	-55.337	-64.033	-46.641	1.06E-35
11	24.127	84.450	-60.323	-69.880	-50.767	3.71E-35

Abbreviations: RMST, restricted mean survival time; LCI, lower limit of 95% confidence interval; UCI, upper limit of 95% confidence interval.

Table S26 Comparing RMST between high and low SRS groups in GSE16011**.**

Year	High SRS RMST	Low SRS RMST	Estimation	LCI	UCI	P-value
1	8.377	10.303	-1.926	-2.795	-1.057	1.40E-05
2	12.089	17.790	-5.701	-7.662	-3.741	1.20E-08
3	14.347	23.804	-9.457	-12.479	-6.435	8.62E-10
4	15.773	28.750	-12.977	-16.945	-9.009	1.45E-10
5	16.939	32.993	-16.054	-20.952	-11.155	1.33E-10
6	18.040	36.737	-18.697	-24.523	-12.871	3.18E-10
7	19.109	39.869	-20.760	-27.470	-14.049	1.34E-09
8	20.111	42.590	-22.479	-30.044	-14.914	5.75E-09
9	21.061	44.862	-23.801	-32.171	-15.431	2.50E-08
10	21.962	46.685	-24.723	-33.838	-15.608	1.06E-07
11	22.551	48.127	-25.575	-35.290	-15.861	2.47E-07
12	23.094	49.257	-26.162	-36.423	-15.901	5.81E-07
13	23.637	50.146	-26.509	-37.291	-15.727	1.44E-06
14	24.180	51.036	-26.856	-38.202	-15.510	3.50E-06
15	24.723	51.925	-27.203	-39.150	-15.255	8.10E-06
16	25.266	52.762	-27.496	-40.053	-14.939	1.77E-05
17	25.808	53.073	-27.265	-40.217	-14.312	3.70E-05
18	26.351	53.218	-26.867	-40.150	-13.584	7.36E-05
19	26.818	53.218	-26.400	-39.910	-12.890	0.000128128
20	27.090	53.218	-26.129	-39.782	-12.475	0.000176276

Abbreviations: RMST, restricted mean survival time; LCI, lower limit of 95% confidence interval; UCI, upper limit of 95% confidence interval.
