# Supplementary figures and images for: Integrative analysis of SEPN1 in glioma: Prognostic roles, functional implications, and potential therapeutic interventions
Source: PLoS One. 2025 Feb 7;20(2):e0318501. doi: 10.1371/journal.pone.0318501 (PMC11805447; doi:10.1371/journal.pone.0318501)

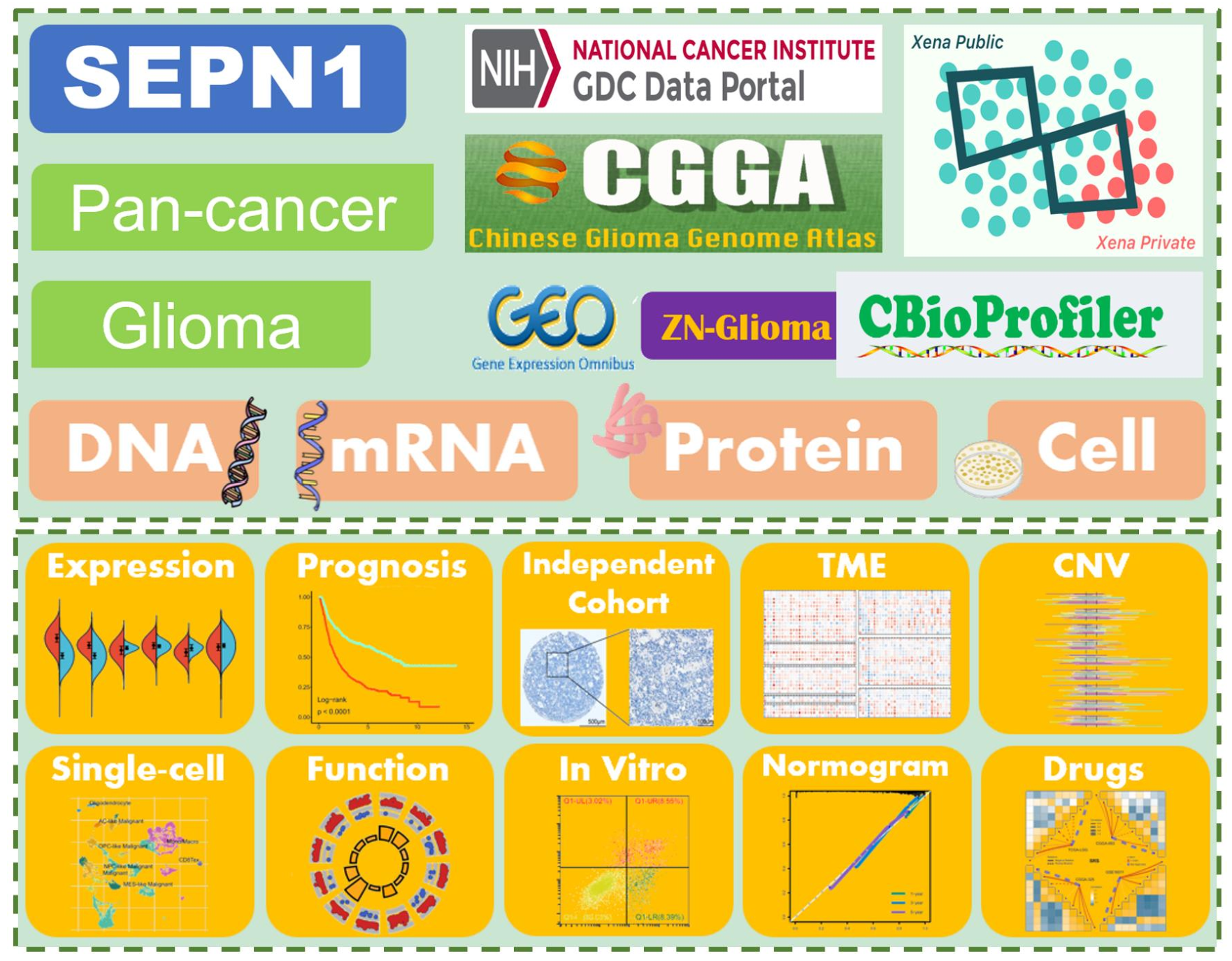

Supplement: S1 Graphical abstract — (PNG) [file pone.0318501.s002.png]
